# Carbon-to-nitrogen atom swap enables direct access to benzimidazoles from drug-like indoles

**DOI:** 10.1038/s41557-025-01904-x

**Published:** 2025-09-02

**Authors:** Ann-Sophie K. Paschke, Yannick Brägger, Bence B. Botlik, Erich Staudinger, Ori Green, Bill Morandi

**Affiliations:** Laboratorium für Organische Chemie, ETH Zürich, Zurich, Switzerland

**Keywords:** Synthetic chemistry methodology, Synthetic chemistry methodology

## Abstract

The ability to selectively edit organic molecules at the atomic level has the potential to streamline lead discovery and optimization in the pharmaceutical and agrochemical industry. While numerous atom insertion and deletion reactions have recently been reported, examples of single atom swaps remain scarce due to the challenge of orchestrating the selective cleavage and formation of multiple chemical bonds around the same atom. Here we report a method for the carbon-to-nitrogen atom swap in *N-*alkyl indoles, allowing the direct conversion of indoles to the corresponding benzimidazoles. The reaction leverages the innate reactivity of the indole scaffold to engage in an initial oxidative cleavage step, followed by oxidative amidation, Hofmann-type rearrangement and cyclization. This complex sequence of steps is mediated by the simple combination of commercially available phenyliodine(III) diacetate and ammonium carbamate as the nitrogen atom source. The reaction tolerates a wide range of functional groups, which is demonstrated by the interconversion of 15 drug-like molecules, implying its immediate applicability across a wide range of discovery programmes.

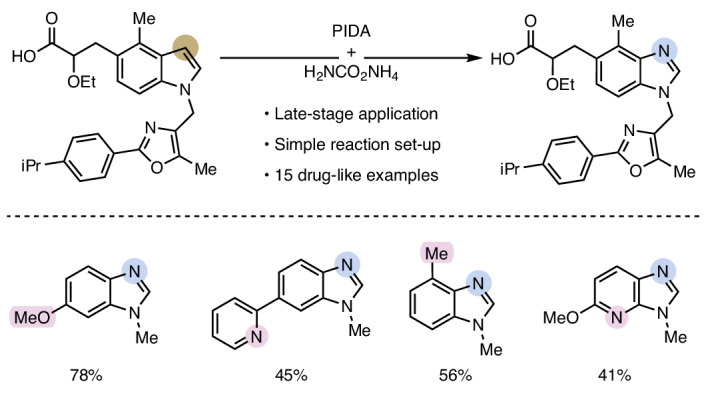

## Abstract (web summary)

Underexplored atom swap reactions offer the opportunity to selectively edit organic molecules and to streamline lead discovery and optimization in the pharmaceutical and agrochemical industry. Now it has been shown that benzimidazoles can be accessed from indoles via C-to-N atom swap. The method was applied to 15 drug-like indoles.

## Main

**Fig. 1:**
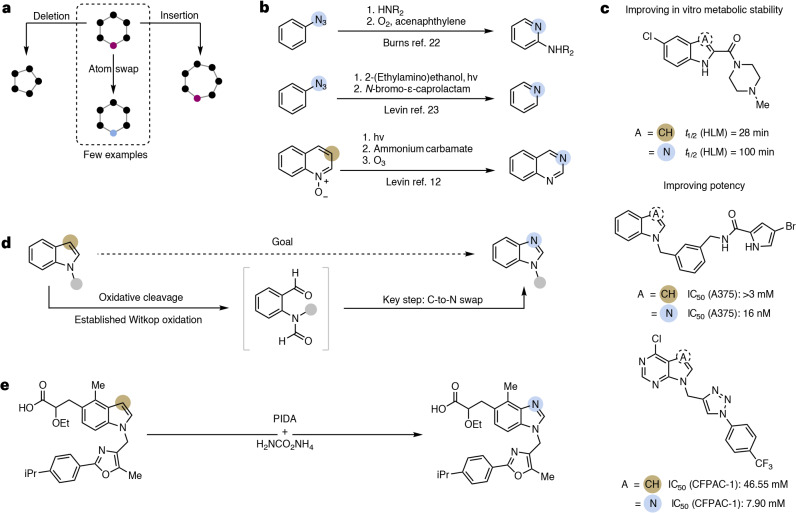
Background and concept. **a**, Context of the work: while atom deletion and atom insertion reactions have been described recently, atom swap reactions are scarce. **b**, Literature precedents for carbon-to-nitrogen atom swaps. Usually, multiple steps and specific functional groups are required^12,22,23^. hv represents a photochemical reaction (light irradiation) and *t*_1/2_ denotes half-life. **c**, Examples of the ‘necessary nitrogen’ effect by exchanging a carbon atom for a nitrogen atom, which leads to improvement in metabolic stability or potency^53–55^. IC_50_, half maximal inhibitory concentration. **d**, The reaction design for the direct transformation of indoles to benzimidazoles via the Witkop oxidation intermediate. **e**, Developed carbon-to-nitrogen atom swap methodology using ammonium carbamate and phenyliodine(III) diacetate (PIDA) allows the transformation of complex drug-like substrates.

Selectively editing organic molecules at the atomic level has been a long-standing vision of organic chemists^1^. Besides the conceptual appeal of this paradigm, it also bears disruptive potential for the rapid discovery and synthesis of key molecules in diverse areas, including pharmaceuticals, agrochemicals, fragrances and organic materials^2^. Inspired by venerable organic transformations, such as the Beckmann rearrangement, and equipped with advanced synthetic tools, chemists have recently developed creative approaches to directly edit the core skeleton of molecules^3^. For example, numerous methods to insert^4–8^, or delete single atoms^9–11^, such as carbon and nitrogen, from a molecular framework have recently been disclosed. As an alternative approach to traditional, orthogonal syntheses, these methods represent a straightforward strategy for scaffold diversification. Circumventing labour-intensive de novo synthesis by making use of already existing libraries offers tremendous potential to rapidly identify and optimize leads while assessing valuable structure–activity relationships (SARs) and, thus, accelerate drug discovery (Fig. 1a). Among skeletal editing strategies, the swap between two atoms is arguably the most challenging variant as it involves the selective sequential cleavage and formation of strong bonds around the same atom. At the same time, this type of transformation is the most appealing from a medicinal chemist’s point of view owing to minimal changes in the molecule’s topology, accounting for its utility in SARs^12,13^. Recently, various groups reported promising strategies for single-atom exchange reactions that expand the toolbox of late-stage editing methodologies. Besides isotope exchange reactions^14–17^, net N-to-C (refs. ^18–20^), O-to-N (refs. ^13,21^) and C-to-N (refs. ^12,22,23^) swaps have been disclosed (Fig. 1b). The latter are particularly desirable with regard to the notion of ‘necessary nitrogen atoms’^24^ and their potential to facilitate nitrogen atom scans of the respective parent compound. However, these are among the most challenging reactions to develop, because it is a daunting task to surgically perform and orchestrate a sequence of carbon atom excision, nitrogen insertion and ring closure, all while not altering any other peripheral functional groups. A strategy to circumvent this challenge is to preinstall an azide onto an arene, which—upon nitrene generation—can insert into benzene rings, ultimately forming pyridines^23^. A complementary strategy can convert quinoline *N*-oxides, a specific class of *N*-heterocycles, to quinazolines in a three-step protocol^12^. The creativity and complexity of these protocols not only underscore the challenges mentioned above, but also reveal numerous critical limitations in the state of the art: (1) the vast majority of (hetero)arenes cannot be used for a C-to-N swap; (2) substrate preactivation (versus using the ‘native’ heterocycle) is necessary, limiting their applicability, particularly with regard to late-stage derivatization of drug-like molecules.

Indoles and related π-excessive *N*-heterocycles (for example, aza-indoles) are prime examples of desirable substrates for the development of a C-to-N swap. They are present in a large number of commercial drugs (ranking sixth among the most frequently appearing *N*-heterocycles in FDA-approved drug molecules as of 2024)^25^, owing to their abundance in naturally occurring alkaloid compounds. Despite their wide-spread application in drug development programmes, indoles are considered challenging scaffolds in modern drug discovery campaigns as they tend to be oxidized easily at their metabolic hot spots (that is, 2- and 3-position) to highly reactive metabolites, leading to undesirable absorption–distribution–metabolism–excretion and/or toxicity profiles^26^. Changing from a more electron-rich heterocycle, such as an indole, to a less electron-rich one, such as a benzimidazole, often has a positive effect on pharmacokinetics (Fig. 1c, top) by removing metabolic hot spots and decreasing phase I (cytochrome P450 enzyme) oxidations, ultimately improving the success rate in lead optimization campaigns^27–29^. In addition, easy remodelling of indole drugs to benzimidazoles would allow a rapid library extension and, thus, open new avenues for lead identification and optimization due to a substantial change in the drug’s pharmacological properties and the introduction of a new target binding site with the potential of boosting potency (Fig. 1c, bottom). However, there is currently no method to directly convert indoles into benzimidazoles through a C-to-N swap, despite the enormous synthetic potential of such a process to perform late-stage diversification campaigns. Ideally, such a reaction would directly convert ‘native’ indole substrates, without any prefunctionalization, under simple reaction conditions to ensure rapid adoption by synthetic practitioners^30^.

In our design (Fig. 1d), we aimed to avoid the use of preactivation strategies to develop an operationally simple C-to-N swap in indoles. The most efficient approach would therefore be to leverage the innate reactivity of the native indole skeleton to initiate this process. We thus envisaged a strategy in which the oxidative cleavage (Witkop oxidation)^31,32^ of the electron-rich indole ring could be used for the initial ring-cleavage step to access a dicarbonyl intermediate. Next, we would need to remove a carbon unit and replace it with a nitrogen atom, before the final ring closure, to form the aromatic benzimidazole product. All steps would need to be orchestrated in a single reaction to maximize practicality and truly allow a direct indole-to-benzimidazole conversion without altering the peripheral functional groups.

Here, we describe the successful development of such a C-to-N swap reaction in which native indole and azaindole substrates are directly converted to the corresponding benzimidazoles and azabenzimidazoles using inexpensive, commercially available reagents. The high functional group tolerance of this reaction was demonstrated on a large number of drug-like molecules, unambiguously showcasing its potential for the immediate discovery of unprecedented medicinally relevant compounds (Fig. 1e).

## Results

**Fig. 2:**
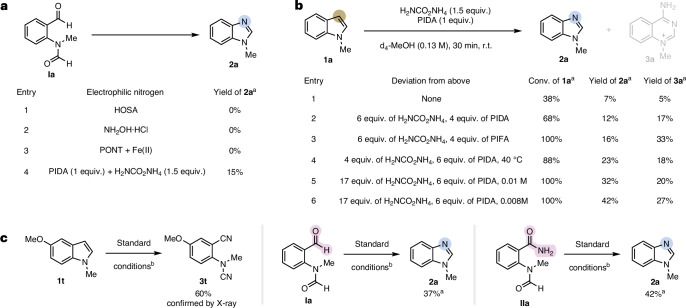
Reaction development. **a**, Experiments with *N*-(2-formylphenyl)-*N*-methylformamide **Ia** to access methyl benzimidazole **2a**. Reactions were performed on a 0.10-mmol scale. **b**, Optimization of the carbon-to-nitrogen atom swap with methyl indole **1a** as model substrate. Reactions were performed on a 0.10-mmol scale. **c**, Control experiments with different possible reaction intermediates confirming the oxidative cleavage of indoles under the reaction conditions and possible Hofmann rearrangement from substrates **Ia** and **IIa**. ^a^Conversion and yields were determined using ^1^H-NMR spectroscopy of the crude reaction mixture using 1,2-dimethoxyethane (1,2-DME) as an internal standard. ^b^Standard conditions refer to PIDA (6.0 equiv.) and ammonium carbamate (17.0 equiv.) in d_4_-MeOH (0.008 M) at room temperature (r.t.) performed on a 0.05-mmol scale. HOSA, hydroxylamine-*O*-sulfonic acid; conv., conversion.

To test the viability of our hypothesis and evaluate possible reaction conditions, we initially focused on possible rearrangement reactions starting from the Witkop oxidation product **Ia**, as this compound is readily accessible from the oxidative cleavage of the corresponding methyl indole **1a** (ref. ^33^). In these preliminary experiments, we explored a range of conditions that are known to facilitate Beckmann or Hofmann-type rearrangements, as well as conditions for the electrophilic amination of hydrocarbons (for example, Fe(II)/*O*-pivaloyl hydroxylammonium triflate (PONT)^34^ and iodonitrenes), aiming at detecting the formation of the corresponding benzimidazole **2a**. While most conditions failed to give the desired product, a combination of a hypervalent iodine reagent and ammonium carbamate gave the first positive results, allowing the formation of *N*-methyl benzimidazole **2a** in 15% ^1^H-NMR yield (Fig. 2a).

Given that hypervalent iodine reagents can also be used for the oxidative cleavage of indoles^35,36^, we directly attempted a one-pot reaction from indoles under the same reaction conditions. Excitingly, we obtained a low but promising ^1^H-NMR yield of 7% of the desired product **2a** allowing the direct conversion of methyl indole **1a** to the corresponding benzimidazole product (Fig. 2b). Further optimization of this reaction led to an improved ^1^H-NMR yield of 42% when more equivalents of ammonium carbamate and oxidant were used at lower reaction concentrations (Supplementary Table 8). Notably, it was possible to isolate 27% of 4-amino-1-methylquinazolin-1-ium **3a** as a side product, a species that degrades under the reaction conditions, thus accounting for the poor mass balance. Together with other side products (vide infra) it could easily be separated from the desired product by column chromatography (Supplementary Table 8).

Intrigued by the possibility to realize this cascade transformation, involving multiple bond cleaving and reforming steps under a single set of reaction conditions, we next conducted several control experiments (Fig. 2c). When 5-methoxy-1-methyl indole **1t** was subjected to the reaction conditions, a dinitrile species **3t** was isolated as a major side product, presumably originating from the overoxidation of the Witkop intermediate, confirming the occurrence of oxidative cleavage in the process. Next, we subjected a series of compounds to the reaction conditions to evaluate their chemical competence as possible intermediates. The Witkop substrate **Ia** gave the desired product in similar yield (37% compared with 42% with **1a** as starting material (s.m.)), supporting its possible intermediacy in the overall one-pot process. We envisage that, under our reaction conditions, this compound could next be converted to a primary amide through an oxidative amidation pathway in analogy to a literature-known process^37^. We thus probed whether amide **IIa** could be a competent intermediate, and again obtained the desired product **2a** in a similar yield. Mechanistically, this intermediate could then undergo a Hofmann-type rearrangement, which is also known to be mediated by hypervalent iodine reagents^38,39^, and a final cyclization step could explain the benzimidazole product formation.

With the optimized reaction conditions in hand, we set out to explore the functional group tolerance and synthetic utility of this transformation. The reaction tolerates different alkyl groups on the indole nitrogen, giving the corresponding benzimidazoles in good yields (Table 1). Little influence of steric hindrance of those groups was observed, and this generality is important in the context of late-stage skeletal editing of drug-like molecules (vide infra). Groups that are commonly used as protecting groups, that is, the benzyl group in molecule **1h**, were also well tolerated under the reaction conditions, allowing an atom swap-dealkylation sequence to access the free NH benzimidazole product **2h** in 47% isolated yield over two steps.

**Table 1:**
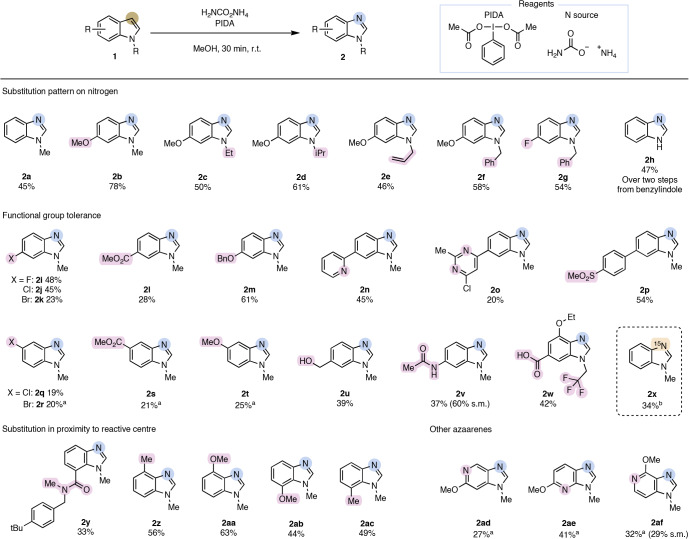
Substrate scope for the carbon-to-nitrogen atom swap

Yields are given for isolated products unless stated otherwise. The recovered s.m. is stated in brackets. Reaction conditions: indole (0.10–0.25 mmol) and ammonium carbamate (17.0 equiv.) were dissolved in methanol (0.008 M) in a pressure tube. After 15 min of stirring, PIDA (6.0 equiv.) was added, and the reaction mixture was stirred for another 15 min at r.t.

^a^To achieve further conversion, monitored by ^1^H-NMR after solvent evaporation, an additional portion of the reagents and solvent was added.

^b^Modified reaction conditions were used: ^15^N-ammonium chloride (17.0 equiv.), K_2_CO_3_ (17.0 equiv.) and PIDA (6.0 equiv.) in 0.008 M methanol.

Indoles with electron-donating functional groups, including methoxy and amide groups, allowed access to the corresponding atom swap products **2b** and **2v** in good yields. Benzimidazoles with electron-withdrawing groups, such as halogens (**2i** to **2k** and **2q** and **2r**), esters (**2l** and **2s**) or sulfones (**2p**), were obtained in moderate-to-good yields. Moreover, indoles bearing a free alcohol (**2u**) or pyridine substituents (**2n**) performed well and gave the desired benzimidazole. Despite being structurally simple, the access to several of these benzimidazoles is restricted given the limitations in common benzimidazole syntheses via condensation reactions^40^ or selectivity issues in the *N*-alkylation of benzimidazoles^41^. For both electron-donating and electron-withdrawing substituents, substitution in the 5-position (**2q** to **2t**) decreased the yield owing to the formation of the dinitrile derivative of the Witkop intermediate as a side product (vide supra). In those cases, it was necessary to add a second portion of reagents to achieve full starting material conversion. Indoles with substituents in proximity to the reactive centre, that is, substituents in 4- or 7-positions (**2y** to **2ac**), were also efficiently transformed into the corresponding benzimidazoles (Table 1, bottom left). To our delight, the C-to-N atom swap could also be successfully applied to various aza-indoles (**2ad** to **2af**; Table 1, bottom right). This further expands the synthetic utility of our method because protocols to access those imidazopyridines remain scarce regardless of their high relevance in medicinal chemistry^25^. Despite the broad generality of this method, nitro, nitrile and boronic ester functional groups were not tolerated (see ‘Unsuccessful substrates’ section in Supplementary Information). A slightly modified protocol allowed the synthesis of ^15^N-labelled methyl benzimidazole **2x**.

**Fig. 3:**
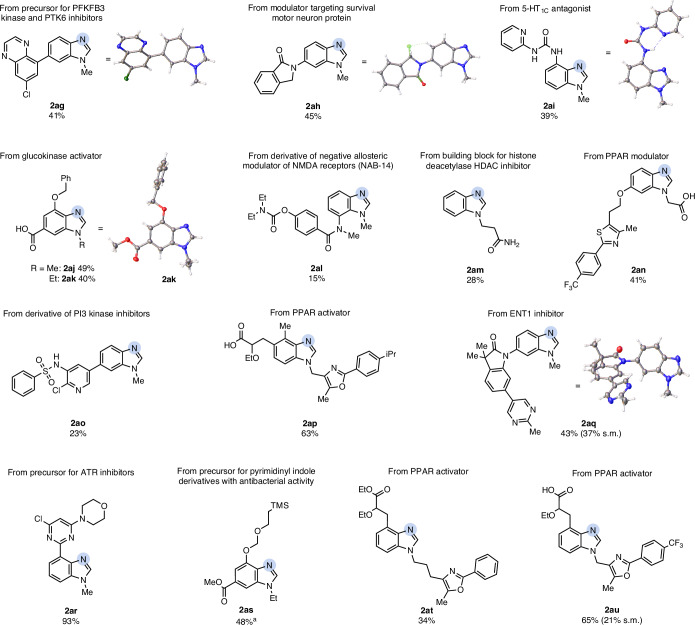
Carbon-to-nitrogen atom swap methodology applied to drug-like molecules. Reactions were performed on a 0.10–0.35-mmol scale, depending on the availability of the substrate. Yields are given for isolated products unless stated otherwise. Recovered starting material is stated in brackets. Reactions conditions: indole (0.10–0.35 mmol) and ammonium carbamate (17.0 equiv.) were dissolved in methanol (0.008 M) in a pressure tube. After 15 min of stirring, PIDA (6.0 equiv.) was added, and the reaction mixture was stirred for another 15 min at r.t. ^a^To achieve further conversion of the starting material, monitored by ^1^H-NMR after solvent evaporation, an additional portion of solvent and reagents was added.

As the above-described results clearly showed the potential of our methodology to access relevant benzimidazole building blocks while tolerating numerous functional groups, the main application area of this process lies in the late-stage diversification of drug-like compounds. We therefore tested our protocol on a wide range of complex, densely functionalized molecules (Fig. 3). The corresponding benzimidazole **2ag** of a precursor derivative for a 6-phosphofructo-2-kinase/fructose-2,6-bisphosphatase 3 (PFKFB3) kinase^42^ and protein tyrosine kinase 6 (PTK6) inhibitor^43^ was obtained in 41% isolated yield, demonstrating that other *N*-heterocycles remain intact under the reaction conditions. The skeleton of drug derivative **1ah**—a structural motif common in compounds known to target the survival motor neuron^44^—gave the corresponding benzimidazole product (**2ah**) in 45% isolated yield, leaving the lactam moiety untouched. Furthermore, a 5-HT_1C_ antagonist analogue^45^ was successfully transformed into the corresponding benzimidazole **2ai** in 39% isolated yield, demonstrating the reaction’s compatibility with urea groups. The benzimidazole analogues of a glucokinase activator (**2aj**)^46^ and a related derivative (**2ak**) could directly be accessed in 49% and 40% isolated yield, respectively. The derivative of NAB-14, a negative allosteric modulator of *N*-methyl-d-aspartate (NMDA) receptors^47^, afforded the corresponding atom swap product **2al** in 15% isolated yield. A histone deacetylase (HDAC) inhibitor motif^48^ yielded the desired product **2am** in 28%, showing the reaction’s tolerance to primary amides. Moreover, a phospatidylinositol-3 (PI3) kinase inhibitor^49^ **1ao** gave the corresponding benzimidazole derivative **2ao** in 23% isolated yield, further showcasing the functional group compatibility of the atom swap with medicinally relevant sulfonamides. To our delight, compound **1aq**, an equilibrative nucleoside transporter subtype 1 (ENT1) inhibitor^50^, gave the desired product **2aq** in 43% isolated yield. It was further demonstrated that valuable building blocks (compounds **2an**, **2ar** and **2as**)^51^ can be transformed into the corresponding benzimidazoles in up to 93% yield, underlining the broad functional group tolerance. Finally, a series of peroxisome proliferator-activated receptor (PPAR) modulators (compounds **2ap**, **2at** and **2au**) yielded the desired product in up to 65% isolated yield, showcasing the reactions compatibility with oxazoles and thiazoles and its applicability in SARs with advanced leads. The identities of **2ag**, **2ah**, **2ai**, **2ak** and **2aq** were unambiguously confirmed by single-crystal X-ray analysis. Overall, the C-to-N atom swap shows excellent compatibility with functional groups relevant in medicinal chemistry and applicability to complex, drug-like molecules.

## Discussion

In summary, we have developed a reaction that can directly convert structurally complex *N*-alkyl indoles into the corresponding benzimidazoles in a single step using commercially available reagents without the need for preinstalled functional groups. Given the simple reaction conditions and the applicability to complex drug-like structures, we believe that this reaction will have an immediate impact on discovery programmes in the pharmaceutical and agrochemical industry. This study also demonstrates that utilizing the innate reactivity of common heterocycles can pave the way for powerful strategies in atom-swapping reactions.

*Note added in proof*: After submission of this paper, Studer and co-workers reported a similar yet orthogonal C-to-N swap approach to convert indoles to benzimidazoles^52^.

## Methods

### C-to-N atom swap of indoles to benzimidazoles

The general procedure for the C-to-N atom swap of indoles to benzimidazoles was as follows. The alkylated indole (0.25 mmol, 1.0 equiv.) was added to a 150-ml pressure tube. Ammonium carbamate (332 mg, 17.0 equiv.) and methanol (30 ml) were added. The tube was sealed and stirred at room temperature at 600 rpm for 15 min. The stirring was stopped, and PIDA (483 mg, 6 equiv.) was added. The tube was sealed again, and the mixture was stirred for an additional 10 min. The solvent was removed under reduced pressure, and the crude product was purified by column chromatography on silica gel (0% to 100% ethyl acetate in hexanes, then 0% to 20% methanol in dichloromethane).

## Online content

Any methods, additional references, Nature Portfolio reporting summaries, source data, extended data, supplementary information, acknowledgements, peer review information; details of author contributions and competing interests; and statements of data and code availability are available at 10.1038/s41557-025-01904-x.

## Supplementary information

Supplementary Information Supplementary discussion, protocols, analytical data, schemes and Tables 1–8.

## Data Availability

Crystallographic data for compounds reported in this article are freely available at the Cambridge Crystallographic Data Centre under deposition CCDC 2379390 (**2ah**), 2388835 (**2ag**), 2388836 (**2ai**), 2388837 (**3t**), 2388838 (**2aq**), 2388839 (**2ak**) and 2388840 (**SI-8**). All other data are available in this article or its Supplementary Information.
