# Supplementary material for: Carbon-to-nitrogen atom swap enables direct access to benzimidazoles from drug-like indoles
Source: Nat Chem. 2025 Sep 2;17(11):1750–6. doi: 10.1038/s41557-025-01904-x (PMC12580326; doi:10.1038/s41557-025-01904-x)
Supplement: Supplementary file 1 — Supplementary discussion, protocols, analytical data, schemes and Tables 1–8. [file 41557_2025_1904_MOESM1_ESM.pdf]

nature chemistry

Supplementary information https://doi.org/10.1038/s41557-025-01904-x

Carbon-to-nitrogen atom swap enables direct access to benzimidazoles from drug-like indoles

In the format provided by the
authors and unedited

TABLE OF CONTENTS

Contents

1 General Information 2

2 Reaction Optimization 4

3 Reactions with Possible Intermediates and Mechanistic Experiments 12

4 Starting Material Synthesis 16

5 Substrate Scope 34

6 Crystallographic Data 56

7 References 63

8 NMR Spectra of Starting Materials 65

9 NMR Spectra of Products 107

1 General Information

Materials: Unless otherwise stated, reagents were used as supplied from commercial sources without any further purification. PIDA was purchased from Fluorochem, ammonium carbamate (99% purity) from Sigma-Aldrich, and methanol (≥ 99.9% HPLC gradient grade) from Sigma-Aldrich. Compounds **1w**, **1y**, **1aj**, **1ak**, **1am**, **1an**, **1ap**, **1aq**, **1as**, **1at** and **1au** were received from F. Hoffmann-La Roche and used without further purification.

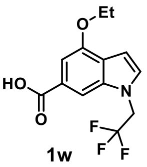

1w

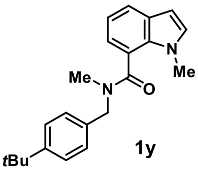

1y

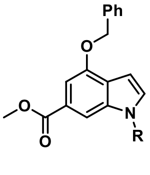

R = Me: 1aj
Et: 1ak

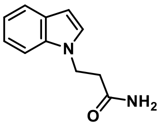

1am

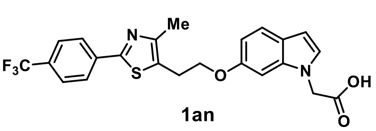

1an

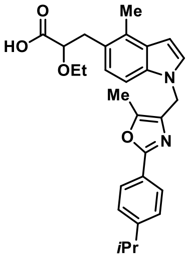

1ap

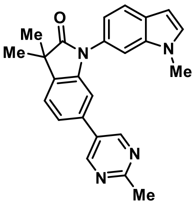

1aq

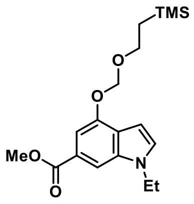

1as

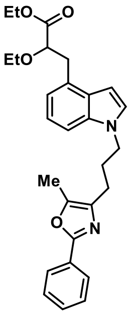

1at

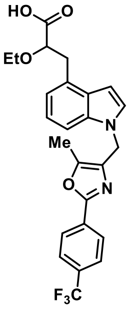

1au

NMR: ^{1}H-, ^{15}N-, ^{19}F- and ^{13}C-NMR spectra were recorded on a Bruker AVIII 400 MHz, a Bruker Neo 400 MHz, a Bruker AVIII HD 600 or a Bruker Neo 500 MHz spectrometer and are reported in parts per million (ppm). ^{1}H-NMR spectra are calibrated with respect to the corresponding solvent residual peak ($CHCl_3$: 7.26 ppm; CH_3OH: 3.31 ppm; DMSO: 2.50 ppm; acetone: 2.05 ppm; DCM: 5.32 ppm). ^{13}C-NMR spectra are calibrated with respect to the corresponding solvent residual peak ($CHCl_3$: 77.16 ppm; CH_3OH: 49.00 ppm; DMSO: 39.52 ppm; acetone: 29.84 ppm; DCM: 53.84 ppm). Multiplet signals are reported as follows: s = singlet, d = doublet, t = triplet, q = quartet, p = pentet, h = heptet, m = multiplet, br = broad or combinations thereof. ^{13}C signals are acquired with proton decoupling and are singlets unless otherwise stated. NMR yields were determined using 1,2-DME as an internal standard.

Analytical thin-layer chromatography (TLC) was performed using silica gel 60 F254 coated aluminum sheets (Merck). Visualization was achieved by ultraviolet fluorescence (λ = 254 nm) and/or staining with potassium permanganate ($KMnO_4$).

Flash column chromatography was performed using silica gel 60 (pore size = 60 Å, mesh: 40-63 µm from Sigma-Aldrich or SiliCycle). Automated flash column chromatography was performed on a Biotage Isolera One system with Sfär columns.

High resolution mass spectrometry (HRMS): HRMS data were obtained by the mass spectrometry service in the Laboratorium für Organische Chemie at ETH Zürich on VG-TRIBRIB for electron impact ionization (EI), a Varian IonSpec Spectrometer for electrospray ionization (ESI) or an IonSpec Ultima Fourier Transform Mass Spectrometer for matrix-assisted laser desorption/ionization (MALDI) and are reported as (m/z).

Reverse-phase high pressure liquid chromatography (RP-HPLC): Analytical RP-HPLC was performed on an Agilent infinity II 1260 using C18 column (5 μm, 250 ×4.6 mm). Preparative RP-HPLC was performed on an Agilent infinity II 1260 with C18 column (7 μm, 250 ×21 mm) using a gradient of MeCN in H_2O with 0.1% TFA.

X-Ray analysis: Single crystalline samples were prepared by dissolving the sample in DCM and slow evaporation of the solvent at rt. Single crystalline samples were measured on a Riguka Oxford Diffraction XtaLAB Synergy-S Dualflex kappa diffractometer equipped with a Dectris Pilatus 300 HPAD detector and using microfocus sealed tube Cu-Kα radiation with mirror optics (λ = 1.54178 Å). All measurements were carried out at 100 K (unless otherwise noted) using an Oxford Cryosystems Cryostream 800 sample cryostat. Data collected on the Rigaku instrument were integrated using CrysAlisPro and corrected for absorption effects using a combination of empirical (ABSPACK) and numerical corrections.[1] The structures were solved using SHELXT[2] or SHELXS[3] and refined by full-matrix least-squares analysis (SHELXL),[4] using the program package OLEX2.[5] Unless otherwise indicated below, all non-hydrogen atoms were refined anisotropically and hydrogen atoms were constrained to ideal geometries and refined with fixed isotropic displacement parameters (in terms of a riding model).

Melting points of crystallized samples were measured using a Büchi M-560 device.

2 Reaction Optimization

Equivalent screening with PIDA

In a 4-mL screw-cap vial equipped with a magnetic stirring bar, 1-methyl-1*H*-indole **1a** (13 mg, 13 µL, 0.10 mmol) and ammonium carbamate (x equiv) were dissolved in d_4-MeOH (0.75 mL). The mixture was cooled to 0 °C in an ice-water bath. Then, PIDA (y equiv) was added in one portion. The reaction was stirred for 30 minutes at 0 °C. 1,2-DME (10 µL) was added as an internal standard and the crude reaction mixture was submitted to ^{1}H NMR analysis.

Table 1. Equivalents screening with PIDA

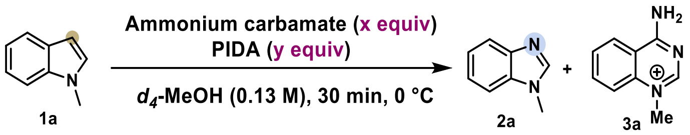

Entry	Equivalents of Ammonium carbamate	Equivalents of PIDA	NMR yield of 2a (%)	NMR conv. of 1a (%)	NMR yield of 3a (%)
1	1.5	1	7	38	5
2	3	2	11	67	13
3	6	4	12	68	17

Oxidant screening

Caution: Addition of the PIFA as the oxidant leads to a vigorously reacting mixture, therefore larger reaction vessels are needed.

In a 4-mL screw-cap vial equipped with a magnetic stirring bar, 1-methyl-1*H*-indole **1a** (13 mg, 13 µL, 0.10 mmol) and ammonium carbamate (47 mg, 0.60 mmol, 6.0 equiv) were dissolved in d_4-MeOH (0.75 mL). The mixture was cooled to 0 °C in an ice-water bath. Then, the indicated oxidant (0.4 mmol, 4 equiv) was added in one portion. The reaction was stirred for 30 minutes at 0 °C. 1,2-DME (10 µL) was added as an internal standard and the crude reaction mixture was submitted to ^{1}H NMR analysis.

Table 2. Oxidant screening

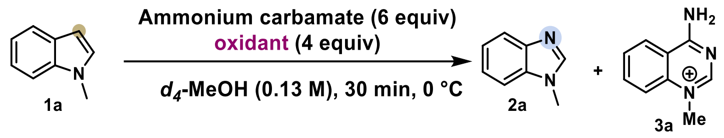

Entry	Oxidant	NMR yield of 2a (%)	NMR conv. of 1a (%)	NMR yield of 3a (%)
1	PIDA	12	68	17
2	PIFA	16	100	33
3	Bis(tert-butylcarbonyloxy)iodobenzene	10	100	19
4	Phenyliodosohydroxy methanesulfonate	4	100	22
5	HTIB	0	100	28
6	$NaIO_4$	0	6	0

Solvent screening

Caution: Addition of the PIFA as the oxidant leads to a vigorously reacting mixture, therefore larger reaction vessels are needed.

In a 4-mL screw-cap vial equipped with a magnetic stirring bar, 1-methyl-1*H*-indole **1a** (13 mg, 13 μL, 0.10 mmol) and ammonium carbamate (47 mg, 0.60 mmol, 6.0 equiv) were dissolved in the indicated solvent (0.75 mL). The mixture was cooled to 0 °C in an ice-water bath. Then, PIFA (170 mg, 0.4 mmol, 4 equiv) was added in one portion. The reaction was stirred for 30 minutes at 0 °C. 1,2-DME (10 μL) was added as an internal standard and the crude reaction mixture was submitted to ^{1}H NMR analysis.

Table 3. Solvent screening

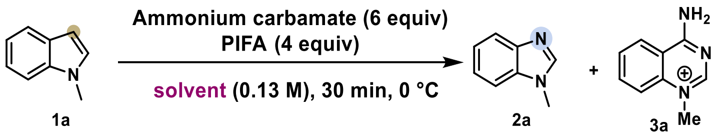

Entry	Solvent	NMR yield of 2a (%)	NMR conv. of 1a (%)	NMR yield of 3a (%)
1	d_4-methanol	16	100	33
2	d_3-MeCN	7	100	4
3	d_2-DCM	0	100	0
4	d_8-THF	15	100	20
5	*d*-chloroform	0	100	0
6	d_8-toluene	0	81	0
7	d_6-DMSO	16	90	0
8	d_6-acetone	0	100	5
9	d_4-acetic acid	0	100	0
10	HFIP	0	100	0

Nitrogen source screening

Caution: Addition of the PIFA as the oxidant leads to a vigorously reacting mixture, therefore larger reaction vessels are needed.

In a 4-mL screw-cap vial equipped with a magnetic stirring bar, 1-methyl-1*H*-indole **1a** (13 mg, 13 µL, 0.10 mmol) and the indicated nitrogen source (0.60 mmol, 6.0 equiv) were dissolved in d_4-MeOH (0.75 mL). The mixture was cooled to 0 °C in an ice-water bath. Then, PIFA (170 mg, 0.4 mmol, 4 equiv) was added in one portion. The reaction was stirred for 30 minutes at 0 °C. 1,2-DME (10 µL) was added as an internal standard and the crude reaction mixture was submitted to ^{1}H NMR analysis.

Table 4. Nitrogen source screening

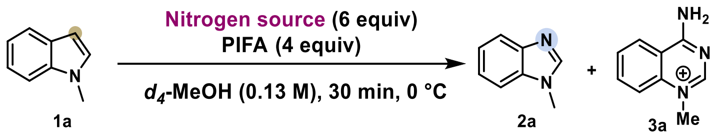

Entry	Nitrogen source	NMR yield of 2a (%)	NMR conv. of 1a (%)	NMR yield of 3a (%)
1	Ammonium carbamate	16	100	33
2	Ammonium chloride	0	100	0
3	Ammonium acetate	0	100	0
4	Ammonium carbonate	9	100	26
5	Ammonia (7 M in MeOH)	0	100	0

Equivalent screening with PIFA

Caution: Addition of the PIFA as the oxidant leads to a vigorously reacting mixture, therefore larger reaction vessels are needed.

In a 4-mL screw-cap vial equipped with a magnetic stirring bar, 1-methyl-1*H*-indole **1a** (13 mg, 13 µL, 0.10 mmol) and ammonium carbamate (x equiv) were dissolved in d_4-MeOH (0.75 mL). The mixture was cooled to 0 °C in an ice-water bath. Then, PIFA (y equiv) was added in one portion. The reaction was stirred for 30 minutes at 0 °C. 1,2-DME (10 µL) was added as an internal standard and the crude reaction mixture was submitted to ^{1}H NMR analysis.

Table 5. Equivalents screening with PIFA

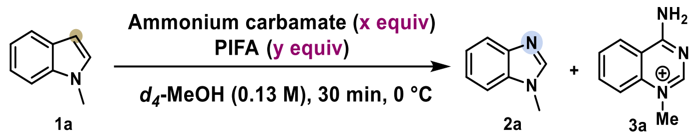

Entry	Equivalents of Ammonium carbamate	Equivalents of PIFA	NMR yield of 2a (%)	NMR conv. of 1a (%)	NMR yield of 3a (%)
1	6	4	16	100	33
2	1	2	0	100	0
3	2	2	0	91	4
4	3	2	9	73	12
5	4	2	9	73	15
6	1	3	0	100	0
7	2	3	0	100	0
8	3	3	0	81	6
9	4	3	13	100	23
10	1	4	0	100	0
11	2	4	0	92	9
12	3	4	0	96	4
13	4	4	0	80	13

Temperature and oxidant screening

Caution: Addition of the PIFA as the oxidant leads to a vigorously reacting mixture, therefore larger reaction vessels are needed.

In a 12-mL screw-cap vial equipped with a magnetic stirring bar, 1-methyl-1*H*-indole **1a** (13 mg, 13 μL, 0.10 mmol) and ammonium carbamate (47 mg, 0.60 mmol, 6.0 equiv) were dissolved in d_4-MeOH (0.75 mL). The mixture was stirred at the indicated temperature. Then, the indicated oxidant (0.4 mmol, 4 equiv) was added in one portion. The reaction was stirred for 30 minutes at the indicated temperature. 1,2-DME (10 μL) was added as an internal standard and the crude reaction mixture was submitted to ^{1}H NMR analysis.

Table 6. Temperature and oxidant screening

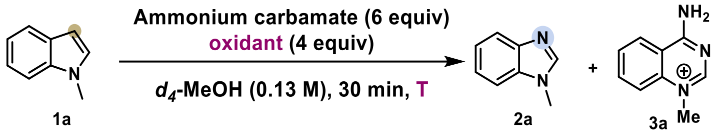

Entry	Temperature	Oxidant	NMR yield of 2a (%)	NMR conv. of 1a (%)	NMR yield of 3a (%)
1	0 °C	PIFA	16	100	33
2	0 °C	PIDA	12	68	17
3	25 °C	PIFA	17	100	27
4	25 °C	PIDA	19	93	18
5	40 °C	PIFA	22	100	0
6	40 °C	PIDA	23	88	18
7	65 °C	PIFA	22	100	0
8	65 °C	PIDA	21	86	14

Screening of equivalents and concentration

In a 20-mL screw-cap vial equipped with a magnetic stirring bar, 1-methyl-1*H*-indole **1a** (13 mg, 13 µL, 0.10 mmol) and ammonium carbamate (x equiv) were dissolved in d_4-MeOH (z M). The mixture was stirred at the indicated temperature. Then, PIDA (y equiv) was added in one portion. The reaction was stirred for 30 minutes at the indicated temperature. 1,2-DME (10 µL) was added as an internal standard and the crude reaction mixture was submitted to ^{1}H NMR analysis.

Table 7. Screening of equivalents and concentration

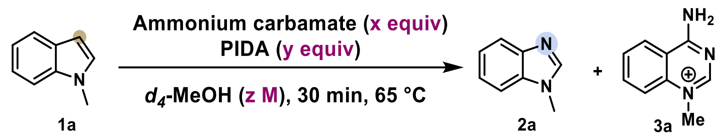

Entry	Equivalents of Ammonium carbamate	Equivalents of PIDA	Conc (M)	NMR yield of 2a (%)	NMR conv. of 1a (%)	NMR yield of 3a (%)
1	6	4	0.13	23	86	14
2	6	4	0.013	28	87	
3	10	3	0.013	20	76	
4	12	5	0.013	31	92	
5	17	6	0.013	32	100	20
6	17	6	0.008	35	100	19
7	10	3	0.008	12	65	7
8	24	12	0.008	32	100	22

Fine-tuning

In a 20-mL screw-cap vial equipped with a magnetic stirring bar, 1-methyl-1*H*-indole **1a** (13 mg, 13 µL, 0.10 mmol) and ammonium carbamate (133 mg, 1.70 mmol, 17.0 equiv) were dissolved in d_4-MeOH (12 mL). The mixture was stirred at 65 °C. Then, PIDA (193 mg, 0.60 mmol, 6.0 equiv) was added in one portion. The reaction was stirred for 30 minutes at this temperature. 1,2-DME (10 µL) was added as an internal standard and the crude reaction mixture was submitted to ^{1}H NMR analysis.

Table 8. Fine-tuning of the reaction conditions

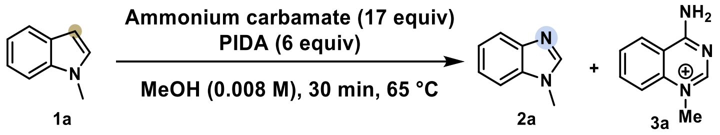

Entry	Deviation from above-described conditions	NMR yield of 2a (%)	NMR conv. of 1a (%)	NMR yield of 3a (%)
1	none	35	100	19
2	Concentration = 0.013 M	32	100	20
3	RT	28	100	28
4	0 °C	25	100	35
5	Addition of PIDA solution *via* syringe pump	5	45	0
6	Reaction in pressure tube	8	18	trace
7	Reaction in pressure tube at RT	42	100	27

3 Reactions with Possible Intermediates and Mechanistic Experiments

Reactions with Possible Intermediates

The following possible intermediates gave the desired product. Reactions were performed on 0.05 mmol scale. Yields were determined using ^{1}H NMR spectroscopy of the crude reaction mixture with 1,2-DME as an internal standard.

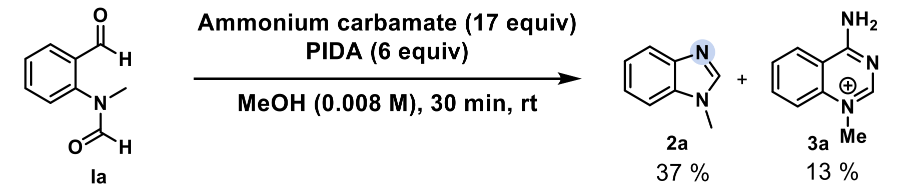

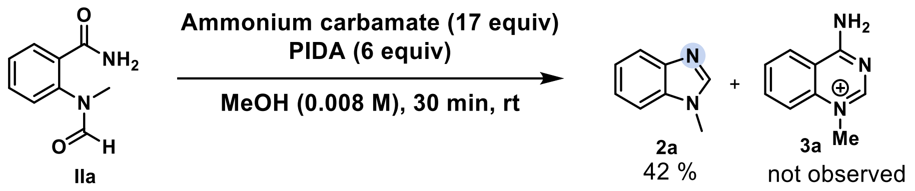

The following intermediates were tested to confirm necessity of the formamide and to exclude other pathways (Beckmann rearrangement, S$_N$Ar, …): **IIIa**, **IVa**, **Va**, **VIa**, **VIIa** and **IXa**. Either no conversion or decomposition and no desired product formation (**2a**) were observed.

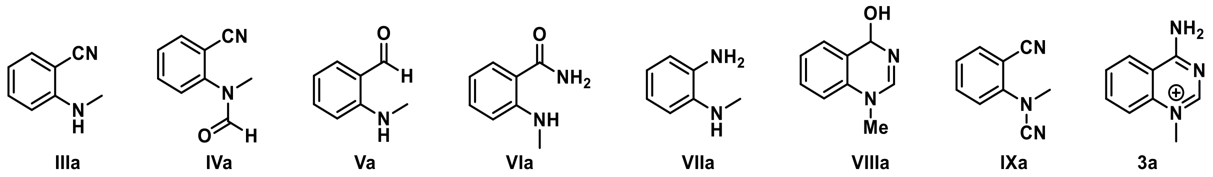

The compounds **IVa** and **IXa** were observed as side products of the reaction in varying amounts depending on the substitution of the starting material.

VIIIa was observed as intermediate and tested under the standard reaction conditions, giving 18 % ^{1}H NMR yield of both, **2a** and **3a**.

Subjection of side product **3a** to the standard reaction conditions leads to unproductive degradation of **3a**. Both, side product formation and their instability under the reaction conditions, account for the poor mass balance. Notably, remaining side products are easily removed from the desired products by flash column chromatography.

Time-dependent ^{1}H NMR Spectroscopy

Time-dependent ^{1}H NMR spectroscopy was performed at -40, -20, 0 and 25 °C for the reaction of **1a** under standard conditions. Even though the reaction proceeds slower at lower temperatures, none of the proposed highly reactive intermediates (isocyanate, aniline) were observed. Qualitative analysis of the spectra showed that intermediate **VIIIa** remains constant as long as starting material **1a** is not consumed. Once the starting material is consumed, it can be observed that **VIIIa** leads to **3a**, thus underlining its role as competent intermediate (see graph c and d).

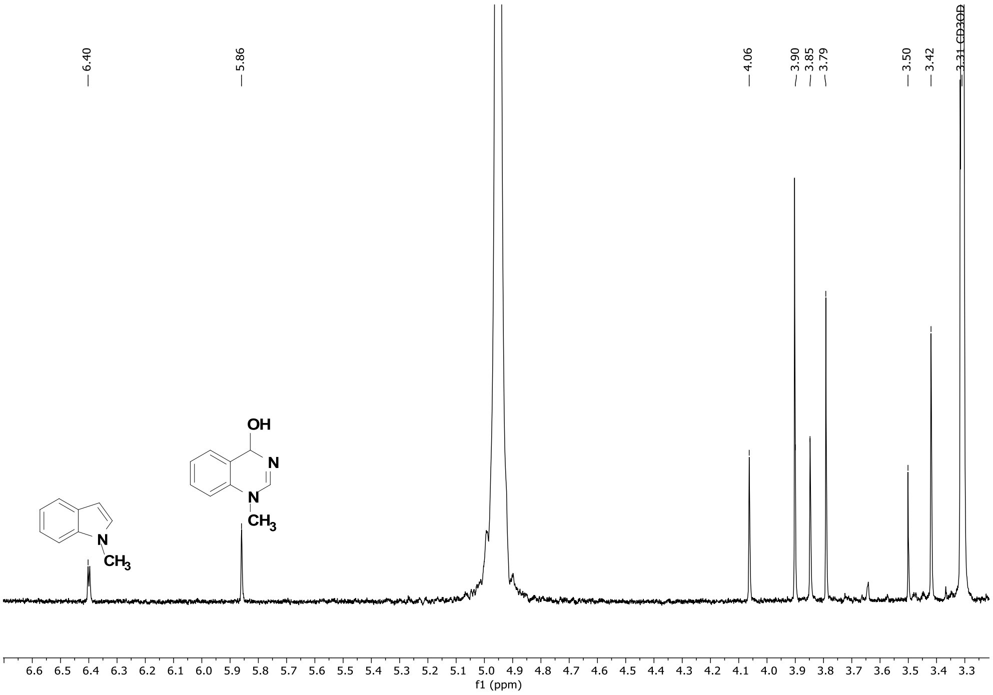

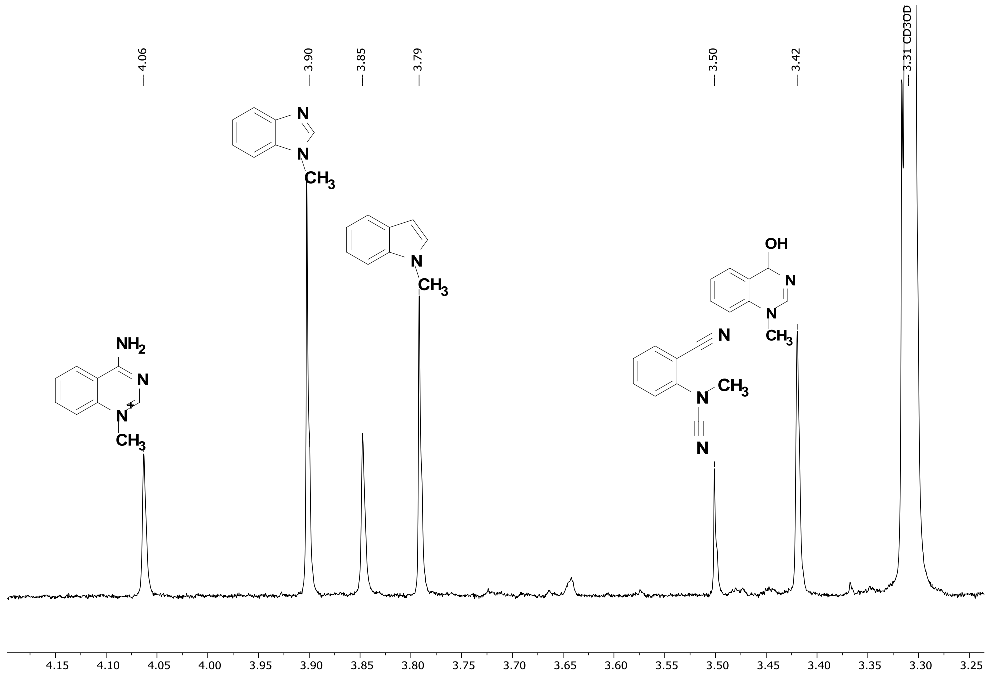

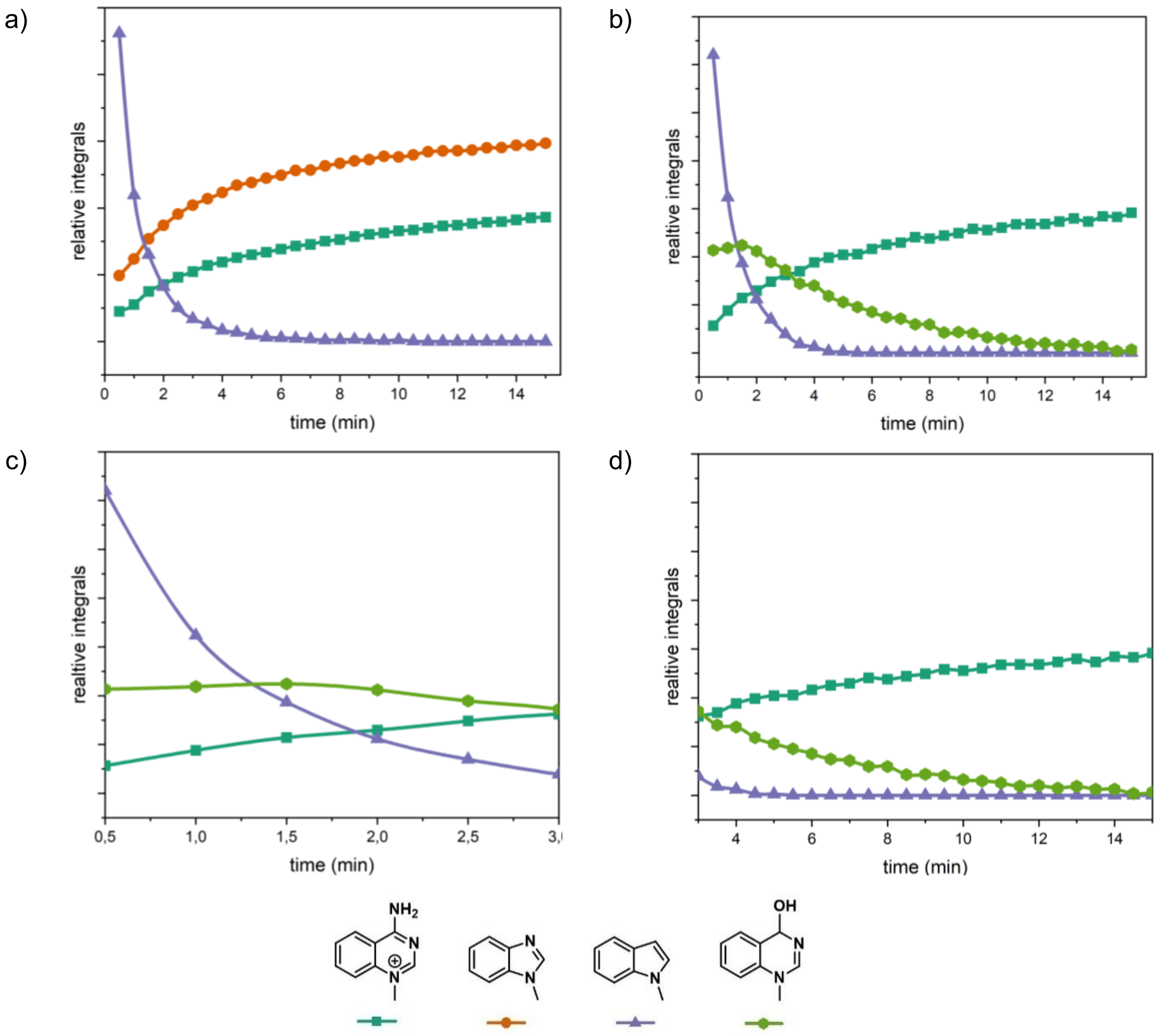
a)
b)
c)
d)
relative integrals
realtive integrals
time (min)
0 2 4 6 8 10 12 14
0,5 1,0 1,5 2,0 2,5 3,
4 6 8 10 12 14
NH2
OH
N

^{13}C-labelling Studies

^{13}C-labelled samples of **1a** (**^{13}C-1a**) and **Ia** (**^{13}C-Ia**) were prepared. When these starting materials were subjected to the standard reaction conditions **^{13}C-2a** and **^{13}C-3a** were observed in the crude NMR. **^{13}C-2a** was isolated. Both experiments show that C-2 is incorporated in the product and the side product, thus, supporting the Hofmann rearrangement hypothesis.

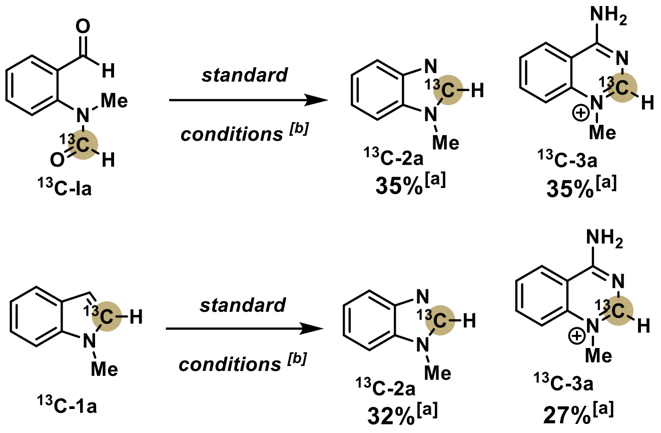

[a] refers to ^{1}H NMR yield with mesitylene as internal standard. [b] Standard conditions refer to PIDA (6.0 equiv) and ammonium carbamate (17.0 equiv) in MeOH (0.008 M) at room temperature.

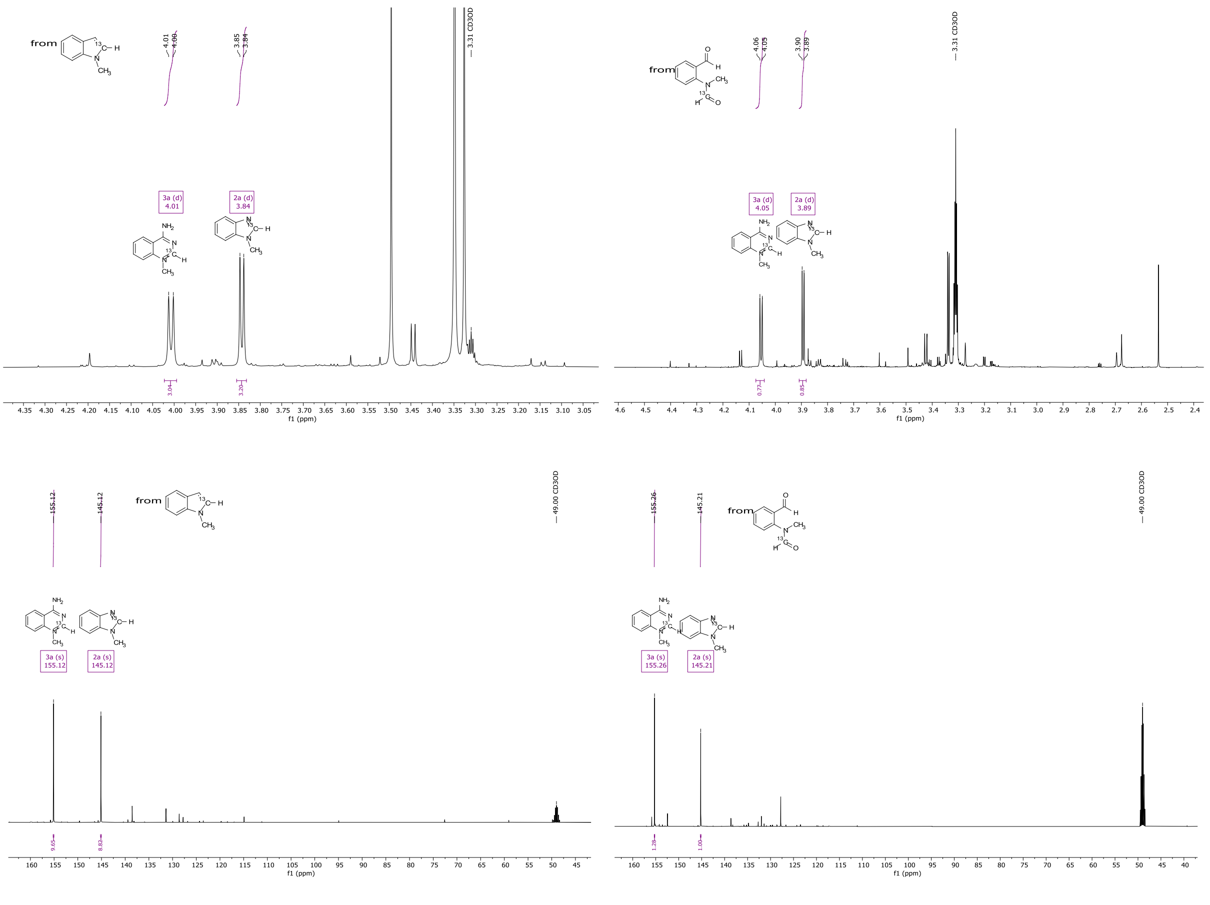

4 Starting Material Synthesis

General Procedure A

The indole derivative (1.0 equiv) was dissolved in DMF and cooled to 0 °C before NaH (1.5 equiv, 60% dispersion in mineral oil) was added portion wise. After stirring at 0 °C for 30 min and rt for an additional 30 min, alkyl halide (1.5 equiv) was added at 0 °C. The reaction was stirred at rt and the reaction was monitored by TLC. Upon full conversion of the starting material, the reaction was cooled to 0 °C and quenched with water. It was extracted with DCM (3 x). The combined organic layers were dried over anhydrous $MgSO_4$ and concentrated under reduced pressure. The crude product was purified with column chromatography on silica gel (0 to 100% EA in hexanes).

1-methyl-1*H*-indole-2-^{13}C (**^{13}C-1a**)

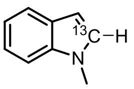

1-methyl-1*H*-indole-2-^{13}C (**^{13}C-1a**) was prepared according to General Procedure A using 1*H*-indole-2-^{13}C (118 mg) as starting material with methyl iodide (97 µL) as alkylating agent. The product was obtained as a light yellow liquid in 96% yield (12 mg).

^{1}H NMR (500 MHz, *d_4*-MeOH) δ 7.52 (ddt, *J* = 8.0, 1.2, 0.6 Hz, 1H), 7.32 (dq, *J* = 8.3, 0.9 Hz, 1H), 7.14 (dddd, *J* = 8.2, 7.0, 1.2, 0.4 Hz, 1H), 7.09 (dd, *J* = 181.6, 3.1 Hz, 1H), 7.01 (ddd, *J* = 8.0, 7.0, 1.0 Hz, 1H), 6.40 (ddd, *J* = 8.6, 3.1, 0.9 Hz, 1H), 3.75 (d, *J* = 3.3 Hz, 3H).

^{13}C NMR (126 MHz, *d_4*-MeOH) δ 134.2, 129.9 (intense), 127.6, 122.2 (d, *J* = 1.1 Hz), 121.5 (d, *J* = 5.5 Hz), 120.0, 110.1 (d, *J* = 2.7 Hz), 101.5 (d, *J* = 68.5 Hz), 32.7 (d, *J* = 2.3 Hz).

HRMS (ESI) m/z: [M+H$^+$] Calculated for $C_8{}^{13}CH_{10}N$ 133.0841 Found 133.0840.

6-Methoxy-1-methyl-1*H*-indole (**1b**)

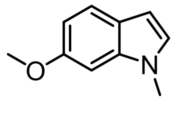

6-Methoxy-1-methyl-1*H*-indole (**1b**) was prepared according to General Procedure A using 6-methoxy-1*H*-indole (294 mg) as starting material with methyl iodide (188 µL) as alkylating agent. The product was obtained as a light yellow solid in 91% yield (292 mg).

^{1}H NMR (400 MHz, $CDCl_3$) δ 7.52 – 7.45 (m, 1H), 6.95 (d, *J* = 3.1 Hz, 1H), 6.82 – 6.77 (m, 2H), 6.42 (dd, *J* = 3.1, 0.8 Hz, 1H), 3.89 (s, 3H), 3.75 (s, 3H).

^{13}C NMR (101 MHz, $CDCl_3$) δ 156.4, 137.5, 127.9, 122.9, 121.6, 109.4, 100.9, 92.9, 55.9, 33.0.

HRMS (ESI) m/z: [M+H$^+$] Calculated for $C_{10}H_{12}NO$ 162.0913; Found 162.0914.

NMR spectra are in agreement with the reported data.[6]

1-Ethyl-6-methoxy-1*H*-indole (**1c**)

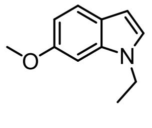

1-Ethyl-6-methoxy-1*H*-indole (**1c**) was prepared according to General Procedure A using 6-methoxy-1*H*-indole (294 mg) as starting material with ethyl iodide (241 µL) as alkylating agent. The product was obtained as yellow oil in 70% yield (244 mg).

^{1}H NMR (400 MHz, $CDCl_3$) δ 7.50 (dd, *J* = 8.5, 0.7 Hz, 1H), 7.02 (d, *J* = 3.2 Hz, 1H), 6.82 (dt, *J* = 1.5, 0.7 Hz, 1H), 6.79 (dd, *J* = 8.5, 2.3 Hz, 1H), 6.43 (dd, *J* = 3.2, 0.8 Hz, 1H), 4.12 (q, *J* = 7.3 Hz, 2H), 3.89 (s, 3H), 1.46 (t, *J* = 7.3 Hz, 3H).

^{13}C NMR (101 MHz, $CDCl_3$) δ 156.3, 136.5, 126.1, 123.1, 121.6, 109.2, 101.1, 93.1, 55.9, 41.0, 15.4.

HRMS (ESI) m/z: [M+H$^+$] Calculated for $C_{11}H_{14}NO$ 176.1070; Found 176.1069.

1-Isopropyl-6-methoxy-1*H*-indole (**1d**)

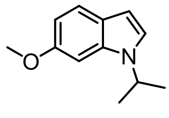

1-Isopropyl-6-methoxy-1*H*-indole (**1d**) was prepared according to General Procedure A using 6-methoxy-1*H*-indole (294 mg) as starting material with isopropyl iodide (300 µL) as alkylating agent. The product was obtained as yellow oil in 38% yield (144 mg).

^{1}H NMR (400 MHz, $CDCl_3$) δ 7.50 (dd, *J* = 8.6, 0.6 Hz, 1H), 7.12 (d, *J* = 3.3 Hz, 1H), 6.85 (d, *J* = 2.3 Hz, 1H), 6.78 (dd, *J* = 8.6, 2.2 Hz, 1H), 6.45 (dd, *J* = 3.3, 0.8 Hz, 1H), 4.60 (p, *J* = 6.7 Hz, 1H), 3.88 (s, 3H), 1.52 (d, *J* = 6.7 Hz, 6H).

^{13}C NMR (101 MHz, $CDCl_3$) δ 156.1, 136.3, 123.1, 122.6, 121.6, 109.2, 101.2, 93.5, 56.0, 47.0, 22.8.

HRMS (ESI) m/z: [M+H$^+$] Calculated for $C_{12}H_{16}NO$ 190.1226; Found 190.1223.

NMR spectra are in agreement with the reported data.[7]

1-Allyl-6-methoxy-1*H*-indole (**1e**)

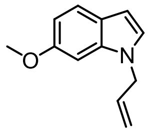

1-Allyl-6-methoxy-1*H*-indole (**1e**) was prepared according to General Procedure A using 6-methoxy-1*H*-indole (294 mg) as starting material with allyl bromide (258 µL) as alkylating agent. The product was obtained as yellow oil in 63% yield (235 mg).

^{1}H NMR (400 MHz, $CDCl_3$) δ 7.50 (d, *J* = 9.3 Hz, 1H), 6.99 (d, *J* = 3.2 Hz, 1H), 6.82 – 6.77 (m, 2H), 6.45 (dd, *J* = 3.2, 0.8 Hz, 1H), 5.99 (ddt, *J* = 17.1, 10.4, 5.3 Hz, 1H), 5.21 (dq, *J* = 10.2, 1.5 Hz, 1H), 5.09 (dtd, *J* = 17.1, 1.7, 1.2 Hz, 1H), 4.68 (dt, *J* = 5.3, 1.7 Hz, 2H), 3.86 (s, 3H).

^{13}C NMR (101 MHz, $CDCl_3$) δ 156.3, 137.0, 133.5, 126.9, 123.1, 121.6, 117.3, 109.4, 101.4, 93.5, 55.9, 49.0.

HRMS (ESI) m/z: [M+H$^+$] Calculated for $C_{12}H_{14}NO$ 188.1070; Found 188.1067.

1-Benzyl-6-methoxy-1*H*-indole (**1f**)

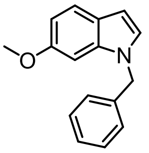

1-Benzyl-6-methoxy-1*H*-indole (**1f**) was prepared according to General Procedure A using 6-methoxy-1*H*-indole (294 mg) as starting material with benzyl bromide (357 µL) as alkylating agent. The product was obtained as a light orange solid in 18% yield (83 mg).

^{1}H NMR (400 MHz, $CDCl_3$) δ 7.52 (dd, *J* = 8.6, 0.6 Hz, 1H), 7.39 – 7.27 (m, 3H), 7.12 (dtd, *J* = 6.5, 1.6, 0.8 Hz, 2H), 7.02 (d, *J* = 3.2 Hz, 1H), 6.79 (dd, *J* = 8.6, 2.2 Hz, 1H), 6.74 (dd, *J* = 2.1, 0.9 Hz, 1H), 6.48 (dd, *J* = 3.2, 0.9 Hz, 1H), 5.27 (s, 2H), 3.80 (s, 3H).

^{13}C NMR (101 MHz, $CDCl_3$) δ 156.4, 137.6, 137.2, 128.9, 127.7, 127.4, 126.9, 123.1, 121.6, 109.5, 101.7, 93.6, 55.8, 50.2.

HRMS (ESI) m/z: [M+H$^+$] Calculated for $C_{16}H_{16}NO$ 238.1226; Found 238.1224.

NMR spectra are in agreement with the reported data.[8]

1-Benzyl-6-fluoro-1*H*-indole (**1g**)

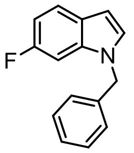

1-Benzyl-6-fluoro-1*H*-indole (**1g**) was prepared according to General Procedure A using 6-fluoro-1*H*-indole (135 mg) as starting material with benzyl bromide (178 µL) as alkylating agent. The product was obtained as white solid in quantitative yield (223 mg).

^{1}H NMR (400 MHz, $CDCl_3$) δ 7.55 (ddd, *J* = 8.6, 5.4, 0.5 Hz, 1H), 7.34 – 7.27 (m, 3H), 7.13 – 7.08 (m, 3H), 6.96 – 6.92 (m, 1H), 6.87 (ddd, *J* = 9.6, 8.6, 2.3 Hz, 1H), 6.53 (dd, *J* = 3.2, 0.9 Hz, 1H), 5.27 (d, *J* = 0.9 Hz, 2H).

^{19}F NMR (376 MHz, $CDCl_3$) δ -120.83.

^{13}C NMR (101 MHz, $CDCl_3$) δ 159.9 (d, *J* = 237.4 Hz), 137.2, 136.5, 129.0, 128.9 (d, *J* = 3.7 Hz), 127.9, 126.9, 125.3, 121.8 (d, *J* = 10.2 Hz), 108.5 (d, *J* = 24.6 Hz), 102.0, 96.3 (d, *J* = 26.3 Hz), 50.4.

HRMS (ESI) m/z: [M+H$^+$] Calculated for $C_{15}H_{13}FN$ 226.1027; Found 226.1028.

NMR spectra are in agreement with the reported data.[9]

6-Fluoro-1-methyl-1*H*-indole (**1i**)

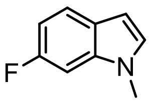

6-Fluoro-1-methyl-1*H*-indole (**1i**) was prepared according to General Procedure A using 6-fluoro-1*H*-indole (270 mg) as starting material with methyl iodide (188 µL) as alkylating agent. The product was obtained as yellow liquid in 93% yield (277 mg).

^{1}H NMR (400 MHz, $CDCl_3$) δ 7.53 (ddd, *J* = 8.6, 5.3, 0.5 Hz, 1H), 7.03 (d, *J* = 3.1 Hz, 1H), 7.00 (ddt, *J* = 9.9, 2.3, 0.7 Hz, 1H), 6.88 (ddd, *J* = 9.6, 8.6, 2.3 Hz, 1H), 6.47 (dd, *J* = 3.1, 0.9 Hz, 1H), 3.75 (s, 3H).

^{19}F NMR (471 MHz, $CDCl_3$) δ -121.23.

^{13}C NMR (126 MHz, $CDCl_3$) δ 160.0 (d, *J* = 237.3 Hz), 136.9 (d, *J* = 12.1 Hz), 129.3 (d, *J* = 3.7 Hz), 125.0, 121.7 (d, *J* = 10.1 Hz), 108.2 (d, *J* = 24.6 Hz), 101.3 (d, *J* = 0.8 Hz), 95.7 (d, *J* = 26.1 Hz), 33.1.

HRMS (ESI) m/z: [M+H$^+$] Calculated for C_9H_9FN 150.0714; Found 150.0715.

NMR spectra are in agreement with the reported data literature.[10]

6-Chloro-1-methyl-1*H*-indole (**1j**)

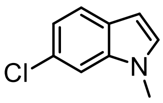

6-Chloro-1-methyl-1*H*-indole (**1j**) was prepared according to General Procedure A using 6-chloro-1*H*-indole (303 mg) as starting material with methyl iodide (188 µL) as alkylating agent. The product was obtained as yellow liquid in quantitative yield (330 mg).

^{1}H NMR (500 MHz, $CDCl_3$) δ 7.5 (dd, *J* = 8.4, 0.6 Hz, 1H), 7.3 (dt, *J* = 1.6, 0.7 Hz, 1H), 7.1 (dd, *J* = 8.4, 1.8 Hz, 1H), 7.0 (d, *J* = 3.1 Hz, 1H), 6.5 (dd, *J* = 3.1, 0.9 Hz, 1H), 3.8 (s, 3H).

^{13}C NMR (126 MHz, $CDCl_3$) δ 137.3, 129.6, 127.9, 127.1, 121.8, 120.1, 109.4, 101.3, 33.0.

HRMS (ESI) m/z: [M+H$^+$] Calculated for C_9H_9ClN 166.0418; Found 166.0420.

NMR spectra are in agreement with the reported data literature.[10]

6-Bromo-1-methyl-1*H*-indole (**1k**)

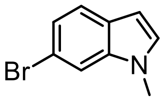

6-Bromo-1-methyl-1*H*-indole (**1k**) was prepared according to General Procedure A using 6-bromo-1*H*-indole (392 mg) as starting material with methyl iodide (188 µL) as alkylating agent. The product was obtained as light-brown solid in 96% yield (402.8 mg).

^{1}H NMR (400 MHz, $CDCl_3$) δ 7.52 – 7.45 (m, 2H), 7.21 (dd, *J* = 8.4, 1.7 Hz, 1H), 7.03 (d, *J* = 3.1 Hz, 1H), 6.46 (dd, *J* = 3.1, 0.9 Hz, 1H), 3.76 (s, 3H).

^{13}C NMR (101 MHz, $CDCl_3$) δ 137.7, 129.6, 127.4, 122.7, 122.2, 115.3, 112.4, 101.3, 33.0.

HRMS (ESI) m/z: [M+H$^+$] Calculated for C_9H_9BrN 209.9913; Found 209.9909.

NMR spectra are in agreement with the reported data.[6]

6-(Benzyloxy)-1-methyl-1*H*-indole (**1m**)

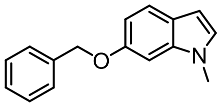

6-(Benzyloxy)-1-methyl-1*H*-indole (**1m**) was prepared according to General Procedure A using 6-(benzyloxy)-1*H*-indole (447 mg) as starting material with methyl iodide (188 µL) as alkylating agent. The product was obtained as light-yellow solid in 86% yield (408 mg).

^{1}H NMR (400 MHz, $CDCl_3$) δ 7.52 – 7.47 (m, 3H), 7.42 – 7.37 (m, 2H), 7.36 – 7.31 (m, 1H), 6.96 (d, *J* = 3.1 Hz, 1H), 6.90 – 6.85 (m, 2H), 6.42 (dd, *J* = 3.1, 0.8 Hz, 1H), 5.15 (s, 2H), 3.73 (s, 3H).

^{13}C NMR (101 MHz, $CDCl_3$) δ 155.6, 137.7, 137.5, 128.7, 128.0, 128.0, 127.7, 123.2, 121.6, 110.0, 101.0, 94.5, 70.9, 33.0.

HRMS (ESI) m/z: [M+H$^+$] Calculated for $C_{16}H_{16}NO$ 238.1226; Found 238.1227.

NMR spectra are in agreement with the reported data.[6]

1-Methyl-6-(pyridin-2-yl)-1*H*-indole (**1n**)

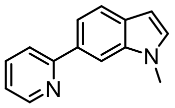

In a flame-dried 20 mL Schlenk tube under nitrogen atmosphere was put 1-methyl-6-(4,4,5,5-tetramethyl-1,3,2-dioxaborolan-2-yl)-1*H*-indole (257 mg, 1 equiv, 1.00 mmol), 2-bromopyridine (158 mg, 95.9 µL, 1 equiv, 1.00 mmol), K_2CO_3 (415 mg, 3 equiv, 3.00 mmol) and tetrakis(triphenylphosphine)palladium(0) (34.7 mg, 0.03 equiv, 30.0 µmol). Degassed THF (3.33 mL) and a freshly prepared, degassed NaOH solution (120 mg in 1.1 mL H_2O, 3 equiv, 3.00 mmol) were added and the mixture was stirred at 70 °C over night. The reaction was cooled to rt and brine was added. It was extracted with DCM three times. The organic layer was dried over anhydrous $MgSO_4$, filtered and the solvent was removed under reduced pressure. The crude product was purified by flash chromatography on silica gel (0 to 100% EA in hexanes) to afford 1-methyl-6-(pyridin-2-yl)-1*H*-indole (**1n**) as bright yellow oil in quantitative yield (209 mg).

^{1}H NMR (400 MHz, $CDCl_3$) δ 8.72 (ddd, *J* = 4.8, 1.9, 1.0 Hz, 1H), 8.11 (q, *J* = 1.1 Hz, 1H), 7.82 (dt, *J* = 8.0, 1.1 Hz, 1H), 7.78 – 7.71 (m, 3H), 7.19 (ddd, *J* = 7.4, 4.9, 1.2 Hz, 1H), 7.12 (d, *J* = 3.1 Hz, 1H), 6.52 (dd, *J* = 3.1, 0.9 Hz, 1H), 3.87 (s, 3H).

^{13}C NMR (101 MHz, $CDCl_3$) δ 158.7, 149.6, 137.4, 136.8, 133.2, 130.4, 129.4, 121.5, 121.0, 120.7, 118.5, 108.1, 101.0, 33.1.

HRMS (ESI) m/z: [M+H$^+$] Calculated for $C_{14}H_{13}N_2$ 209.1073; Found 209.1071.

6-(6-Chloro-2-methylpyrimidin-4-yl)-1-methyl-1*H*-indole (**1o**)

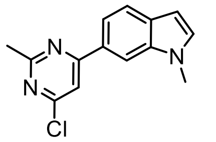

In an oven-dried 20 mL Schlenk tube under nitrogen atmosphere was added 1-methyl-6-(4,4,5,5-tetramethyl-1,3,2-dioxaborolan-2-yl)-1H-indole (309 mg, 1.2 Eq, 1.20 mmol), 4,6-dichloro-2-methylpyrimidine (163 mg, 1 Eq, 1.00 mmol), MeCN (2.57 mL), water (286 µL) and Na_2CO_3 (159 mg, 1.5 Eq, 1.50 mmol). The mixture was stirred for 5 min before tetrakis(triphenylphosphine)palladium(0) (116 mg, 0.1 Eq, 100 µmol) was added. The reaction mixture was heated at 110 °C for 4 hr. It was then cooled to rt, diluted with water and extracted with DCM three times. The organic layer was dried over anhydrous $MgSO_4$, filtered and the solvent was removed under reduced pressure. The crude material was purified by flash column chromatography on silica gel (0 to 100% EA in hexanes) to obtain 6-(6-chloro-2-methylpyrimidin-4-yl)-1-methyl-1*H*-indole (**1o**) as bright yellow solid in 61% yield (158 mg).

^{1}H NMR (400 MHz, $CDCl_3$) δ 8.20 (dt, *J* = 1.5, 0.8 Hz, 1H), 7.75 (dd, *J* = 8.4, 1.5 Hz, 1H), 7.70 (dd, *J* = 8.3, 0.7 Hz, 1H), 7.62 (t, *J* = 0.6 Hz, 1H), 7.19 (d, *J* = 3.1 Hz, 1H), 6.53 (dd, *J* = 3.1, 0.9 Hz, 1H), 3.91 (s, 3H), 2.79 (s, 3H).

^{13}C NMR (101 MHz, $CDCl_3$) δ 169.0, 167.0, 161.4, 137.1, 131.9, 131.4, 129.1, 121.4, 118.5, 113.7, 109.1, 101.5, 33.3, 26.3.

HRMS (ESI) m/z: [M+H$^+$] Calculated for $C_{14}H_{13}ClN_3$ 258.0793; Found 258.0792.

1-Methyl-6-(4-(methylsulfonyl)phenyl)-1*H*-indole (**1p**)

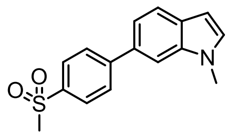

In a flame-dried 20 mL Schlenk tube under nitrogen atmosphere was put 1-methyl-6-(4,4,5,5-tetramethyl-1,3,2-dioxaborolan-2-yl)-1*H*-indole (257 mg, 1 equiv, 1.00 mmol), 4-bromophenyl methylsulfone (235 mg, 1 equiv, 1.00 mmol), K_2CO_3 (415 mg, 3 equiv, 3.00 mmol) and tetrakis(triphenylphosphine)palladium(0) (116 mg, 0.1 equiv, 100 µmol). A degassed mixture of ethanol and toluene (1:1, 8 mL) was added and the mixture was stirred at 80 °C over night. The reaction was cooled to rt and brine was added. It was extracted with DCM three times. The organic layer was dried over anhydrous $MgSO_4$, filtered and the solvent was removed under reduced pressure. The crude product was purified by flash chromatography on silica gel (0 to 100% EA in hexanes) to afford 1-methyl-6-(4-(methylsulfonyl)phenyl)-1*H*-indole (**1p**) as a light brown solid in 68% yield (194 mg).

^{1}H NMR (400 MHz, $CDCl_3$) δ 8.11 – 7.96 (m, 2H), 7.89 – 7.82 (m, 2H), 7.72 (dd, *J* = 8.3, 0.7 Hz, 1H), 7.55 (dt, *J* = 1.7, 0.8 Hz, 1H), 7.38 (dd, *J* = 8.3, 1.6 Hz, 1H), 7.14 (d, *J* = 3.1 Hz, 1H), 6.54 (dd, *J* = 3.1, 0.9 Hz, 1H), 3.87 (s, 3H), 3.11 (s, 3H).

^{13}C NMR (101 MHz, $CDCl_3$) δ 148.3, 138.4, 137.3, 132.9, 130.6, 129.0, 128.2, 128.0, 121.6, 119.2, 108.4, 101.2, 44.9, 33.1.

HRMS (ESI) m/z: [M+H$^+$] Calculated for $C_{16}H_{16}NO_2S$ 286.0896; Found 286.0894.

5-Chloro-1-methyl-1*H*-indole (**1q**)

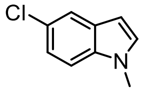

5-Chloro-1-methyl-1*H*-indole (**1q**) was prepared according to General Procedure A using 5-chloro-1*H*-indole (303 mg) as starting material with methyl iodide (188 μL) as alkylating agent. The product was obtained as a light yellow liquid in 62% yield (206 mg).

^{1}H NMR (400 MHz, $CDCl_3$) δ 7.59 (dd, *J* = 2.0, 0.6 Hz, 1H), 7.23 (dt, *J* = 8.7, 0.7 Hz, 1H), 7.17 (ddd, *J* = 8.7, 2.0, 0.4 Hz, 1H), 7.07 (d, *J* = 3.1 Hz, 1H), 6.43 (dd, *J* = 3.1, 0.8 Hz, 1H), 3.78 (s, 3H).

^{13}C NMR (101 MHz, $CDCl_3$) δ 135.3, 130.2, 129.6, 125.2, 121.9, 120.3, 110.3, 100.7, 33.2.

HRMS (ESI) m/z: [M+H$^+$] Calculated for C_9H_9ClN 166.0418; Found 166.0418.

NMR spectra are in agreement with the reported data.[6]

5-Bromo-1-methyl-1*H*-indole (**1r**)

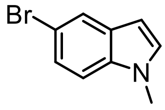

5-Bromo-1-methyl-1*H*-indole (**1r**) was prepared according to General Procedure A using 5-bromo-1*H*-indole (392 mg) as starting material with methyl iodide (188 μL) as alkylating agent. The product was obtained as yellow oil in quantitative yield (421 mg).

^{1}H NMR (500 MHz, $CDCl_3$) δ 7.75 (dd, *J* = 1.9, 0.6 Hz, 1H), 7.30 (ddd, *J* = 8.7, 1.9, 0.4 Hz, 1H), 7.19 (dt, *J* = 8.7, 0.7 Hz, 1H), 7.05 (d, *J* = 3.1 Hz, 1H), 6.42 (dd, *J* = 3.1, 0.9 Hz, 1H), 3.78 (s, 3H).

^{13}C NMR (126 MHz, $CDCl_3$) δ 135.5, 130.3, 130.1, 124.5, 123.4, 112.8, 110.8, 100.7, 33.1.

HRMS (ESI) m/z: [M+H$^+$] Calculated for C_9H_9BrN 209.9913; Found 209.9914.

NMR spectra are in agreement with the reported data.[11]

Methyl 1-methyl-1*H*-indole-5-carboxylate (**1s**)

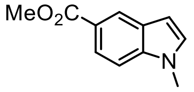

Methyl 1-methyl-1*H*-indole-5-carboxylate (**1s**) was prepared according to General Procedure A using methyl 1*H*-indole-5-carboxylate (350 mg) as starting material with methyl iodide (188 μL) as alkylating agent. The product was obtained as white solid in 97% yield (367 mg).

^{1}H NMR (400 MHz, $CDCl_3$) δ 8.40 (dd, *J* = 1.7, 0.7 Hz, 1H), 7.93 (dd, *J* = 8.7, 1.6 Hz, 1H), 7.33 (dt, *J* = 8.7, 0.8 Hz, 1H), 7.11 (d, *J* = 3.1 Hz, 1H), 6.59 (dd, *J* = 3.2, 0.9 Hz, 1H), 3.93 (s, 3H), 3.82 (s, 3H).

^{13}C NMR (101 MHz, $CDCl_3$) δ 168.4, 139.3, 130.3, 128.1, 124.1, 123.1, 121.5, 109.0, 102.8, 52.0, 33.2.

HRMS (ESI) m/z: [M+Na^+] Calculated for $C_{11}H_{11}NNaO_2$ 212.0682; Found 212.0681.

NMR spectra are in agreement with the reported data.[11]

5-Methoxy-1-methyl-1*H*-indole (**1t**)

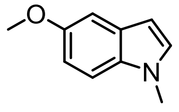

5-Methoxy-1-methyl-1*H*-indole (**1t**) was prepared according to General Procedure A using 5-methoxy-1*H*-indole (294 mg) as starting material with methyl iodide (188 µL) as alkylating agent. The product was obtained as a light yellow solid in 68% yield (220 mg).

^{1}H NMR (400 MHz, $CDCl_3$) δ 7.23 (dt, *J* = 8.8, 0.7 Hz, 1H), 7.12 (dd, *J* = 2.5, 0.6 Hz, 1H), 7.03 (d, *J* = 3.0 Hz, 1H), 6.91 (ddd, *J* = 8.8, 2.5, 0.5 Hz, 1H), 6.42 (dd, *J* = 3.0, 0.9 Hz, 1H), 3.87 (s, 3H), 3.77 (s, 3H).

^{13}C NMR (101 MHz, $CDCl_3$) δ 154.1, 132.3, 129.4, 128.9, 112.0, 110.0, 102.6, 100.5, 56.0, 33.1.

HRMS (ESI) m/z: [M+H^+] Calculated for $C_9H_{11}N_2O$ 163.0866; Found 163.0864.

NMR spectra are in agreement with the reported data.[6]

N-(1-methyl-1*H*-indol-6-yl)acetamide (**1v**)

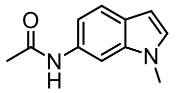

To a solution of 1-methyl-1*H*-indol-6-amine (146 mg, 1 equiv, 1.00 mmol) in DCM (6.67 mL) was added triethylamine (152 mg, 209 µL, 1.5 equiv, 1.50 mmol) and acetyl chloride (86.4 mg, 78.1 µL, 1.1 equiv, 1.10 mmol) at 0 °C. The reaction was stirred at 0 °C for 15 min and for an additional 30 min at rt. The mixture was then diluted with DCM and poured into cold water. The layers were separated, and the organic layer was washed subsequently with 1 M HCl, water and brine. The organic layer was dried over anhydrous $MgSO_4$, filtered and the solvent was removed under reduced pressure. The crude product was purified by flash column chromatography on silica gel (0 to 100% EA in hexanes) to give *N*-(1-methyl-1*H*-indol-6-yl)acetamide (**1v**) as a light rose colored solid in 95% yield (179 mg).

^{1}H NMR (400 MHz, DMSO-d_6) δ 9.92 – 9.78 (m, 1H), 7.88 (dt, *J* = 1.7, 0.7 Hz, 1H), 7.42 (dd, *J* = 8.5, 0.6 Hz, 1H), 7.22 (d, *J* = 3.1 Hz, 1H), 7.04 (dd, *J* = 8.5, 1.8 Hz, 1H), 6.33 (dd, *J* = 3.1, 0.9 Hz, 1H), 3.71 (s, 3H), 2.05 (s, 3H).

^{13}C NMR (101 MHz, DMSO-d_6) δ 168.3, 136.7, 134.2, 129.7, 124.6, 120.6, 112.8, 100.6, 100.6, 32.9, 24.5.

HRMS (ESI) m/z: [M+H^+] Calculated for $C_{11}H_{13}N_2O$ 189.1022; Found 189.1020.

1,4-Dimethyl-1*H*-indole (**1z**)

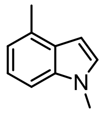

1,4-Dimethyl-1*H*-indole-5-carboxylate (**1z**) was prepared according to General Procedure A using 4-methyl 1*H*-indole (262 mg) as starting material with methyl iodide (188 µL) as alkylating agent. The product was obtained as yellow liquid in 56% yield (163 mg).

^{1}H NMR (400 MHz, $CDCl_3$) δ 7.21 – 7.11 (m, 2H), 7.05 (s, 1H), 6.93 – 6.89 (m, 1H), 6.51 (s, 1H), 3.81 – 3.76 (m, 3H), 2.64 – 2.51 (m, 3H).

^{13}C NMR (101 MHz, $CDCl_3$) δ 136.6, 130.5, 128.5, 128.3, 121.8, 119.6, 106.9, 99.5, 33.1, 18.9.

HRMS (ESI) m/z: [M+H$^+$] Calculated for $C_{10}H_{12}N$ 146.0964; Found 146.0964.

NMR spectra are in agreement with the reported data.[6]

4-Methoxy-1-methyl-1*H*-indole (**1aa**)

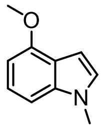

4-Methoxy-1-methyl-1*H*-indole (**1aa**) was prepared according to General Procedure A using 4-methoxy-1*H*-indole (294 mg) as starting material with methyl iodide (188 µL) as alkylating agent. The product was obtained as off-white solid in 86% yield (285 mg).

^{1}H NMR (400 MHz, $CDCl_3$) δ 7.18 (t, J = 8.0 Hz, 1H), 7.02 – 6.95 (m, 2H), 6.62 (dd, J = 3.1, 0.9 Hz, 1H), 6.56 (dd, J = 7.8, 0.7 Hz, 1H), 3.99 (s, 3H), 3.78 (s, 3H).

^{13}C NMR (101 MHz, $CDCl_3$) δ 153.5, 138.3, 127.4, 122.4, 119.1, 102.8, 99.3, 98.3, 55.4, 33.2.

HRMS (ESI) m/z: [M+H$^+$] Calculated for $C_{10}H_{12}NO$ 162.0913; Found 162.0913.

7-Methoxy-1-methyl-1*H*-indole (**1ab**)

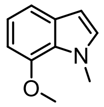

7-Methoxy-1-methyl-1*H*-indole (**1ab**) was prepared according to General Procedure A using 7-methoxy-1*H*-indole (294 mg) as starting material with methyl iodide (188 µL) as alkylating agent. The product was obtained as white solid in 85% yield (275 mg).

^{1}H NMR (400 MHz, $CDCl_3$) δ 7.24 – 7.18 (m, 1H), 7.01 – 6.95 (m, 1H), 6.93 (t, *J* = 2.2 Hz, 1H), 6.62 (dd, *J* = 8.0, 3.1 Hz, 1H), 6.42 (d, *J* = 4.7 Hz, 1H), 4.06 (s, 3H), 3.93 (s, 3H).

^{13}C NMR (101 MHz, $CDCl_3$) δ 148.0, 131.0, 129.9, 126.6, 119.9, 113.9, 102.4, 101.1, 55.5, 36.6.

HRMS (ESI) m/z: [M+H$^+$] Calculated for $C_{10}H_{12}NO$ 162.0913; Found 162.0913.

NMR spectra are in agreement with the reported data.[6]

1,7-Dimethyl-1*H*-indole (**1ac**)

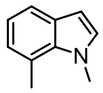

1,7-Dimethyl-1*H*-indole (**1ac**) was prepared according to General Procedure A 7-methyl-1*H*-indole (262 mg) as starting material with methyl iodide (188 µL) as alkylating agent. The product was obtained as off-white solid in 85% yield (247 mg).

^{1}H NMR (400 MHz, $CDCl_3$) δ 7.52 (ddd, J = 7.8, 1.3, 0.6 Hz, 1H), 7.06 – 7.00 (m, 1H), 6.99 – 6.94 (m, 2H), 6.49 (d, J = 3.1 Hz, 1H), 4.09 (s, 3H), 2.82 (t, J = 0.7 Hz, 3H).

^{13}C NMR (101 MHz, $CDCl_3$) δ 135.5, 130.5, 129.7, 124.2, 121.3, 119.7, 119.2, 101.0, 36.9, 19.8.

HRMS (ESI) m/z: [M+H$^+$] Calculated for $C_{10}H_{12}N$ 146.0964; Found 146.0962.

NMR spectra are in agreement with the reported data.[6]

6-Methoxy-1-methyl-1*H*-pyrrolo[3,2-*c*]pyridine (**1ad**)

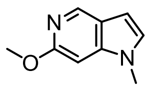

6-Methoxy-1-methyl-1*H*-pyrrolo[3,2-*c*]pyridine (**1ad**) was prepared according to General Procedure A using 6-methoxy-1*H*-pyrrolo[3,2-*c*]pyridine (296 mg) as starting material with methyl iodide (194 µL) as alkylating agent. The product was obtained as a light yellow oil in 95% yield (309 mg).

^{1}H NMR (400 MHz, $CDCl_3$) δ 8.44 (d, *J* = 1.0 Hz, 1H), 6.86 (d, *J* = 3.3 Hz, 1H), 6.51 (t, *J* = 1.0 Hz, 1H), 6.42 (dd, *J* = 3.3, 1.0 Hz, 1H), 3.95 (d, *J* = 0.4 Hz, 3H), 3.59 (d, *J* = 0.4 Hz, 3H).

^{13}C NMR (101 MHz, $CDCl_3$) δ 160.3, 144.0, 139.7, 129.8, 122.1, 100.4, 87.6, 54.0, 32.4.

HRMS (ESI) m/z: [M+H$^+$] Calculated for $C_9H_{11}N_2O$ 163.0866; Found 163.0864.

6-Methoxy-1-methyl-1*H*-pyrrolo[2,3-*b*]pyridine (**1ae**)

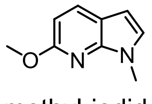

6-Methoxy-1-methyl-1*H*-pyrrolo[2,3-*b*]pyridine (**1ae**) was prepared according to General Procedure A using 6-methoxy-1*H*-pyrrolo[2,3-*b*]pyridine (445 mg) as starting material with methyl iodide (281 µL) as alkylating agent. The product was obtained as colorless liquid in 91% yield (442 mg).

^{1}H NMR (400 MHz, $CDCl_3$) δ 7.77 (d, *J* = 8.4 Hz, 1H), 6.96 (d, *J* = 3.4 Hz, 1H), 6.54 (d, *J* = 8.4 Hz, 1H), 6.36 (d, *J* = 3.4 Hz, 1H), 4.00 (s, 3H), 3.81 (s, 3H).

^{13}C NMR (101 MHz, $CDCl_3$) δ 160.9, 145.7, 131.7, 125.8, 114.2, 103.3, 99.7, 53.4, 31.1.

HRMS (ESI) m/z: [M+H$^+$] Calculated for $C_9H_{11}N_2O$ 163.0866; Found 163.0863.

4-Methoxy-1-methyl-1*H*-pyrrolo[3,2-*c*]pyridine (**1af**)

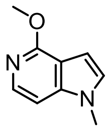

4-Methoxy-1-methyl-1*H*-pyrrolo[3,2-*c*]pyridine (**1af**) was prepared according to General Procedure A using 4-methoxy-1*H*-pyrrolo[3,2-*c*]pyridine (296 mg) as starting material with methyl iodide (194 µL) as alkylating agent. The product was obtained as off-white solid in 38% yield (123 mg).

^{1}H NMR (400 MHz, $CDCl_3$) δ 7.85 (d, *J* = 6.0 Hz, 1H), 6.92 (d, *J* = 3.1 Hz, 1H), 6.86 (dd, *J* = 6.0, 0.9 Hz, 1H), 6.57 (dd, *J* = 3.1, 0.9 Hz, 1H), 4.08 (s, 3H), 3.72 (s, 3H).

^{13}C NMR (101 MHz, $CDCl_3$) δ 158.3, 142.1, 137.7, 127.7, 112.6, 100.5, 99.9, 53.3, 33.1.

HRMS (ESI) m/z: [M+H$^+$] Calculated for $C_9H_{11}N_2O$ 163.0866; Found 163.0867.

7-Chloro-5-(1-methyl-1*H*-indol-6-yl)quinoxaline (**1ag**)

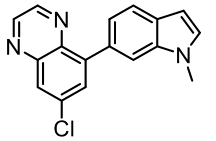

A flame-dried 20 mL Schlenk flask under nitrogen atmosphere was charged with 5-bromo-7-chloroquinoxaline (487 mg, 1 equiv, 2.00 mmol), 1-methyl-6-(4,4,5,5-tetramethyl-1,3,2-dioxaborolan-2-yl)-1*H*-indole (514 mg, 1 equiv, 2.00 mmol), DIPEA (517 mg, 697 µL, 2 equiv, 4.00 mmol), 1,4-dioxane (2.6 mL) and water (2.6 mL). The suspension was purged with nitrogen and 1,1'-bis(diphenylphosphino)ferrocene-palladium(II) dichloride (146 mg, 0.1 equiv, 200 µmol) was added. The reaction mixture was stirred at 85 °C for 3 h. The mixture was then cooled to rt, filtered through a pad of celite and the filtrate was diluted with DCM and extracted with water. The organic layer was dried over anhydrous $MgSO_4$, filtered and the solvent was removed under reduced pressure. The crude product was purified by flash column chromatography on silica gel (0 to 100% EA in hexanes) to obtain 7-chloro-5-(1-methyl-1*H*-indol-6-yl)quinoxaline (**1ag**) as bright yellow solid in 79% yield (464 mg).

^{1}H NMR (400 MHz, $CDCl_3$) δ 8.89 – 8.79 (m, 2H), 8.10 (d, *J* = 2.4 Hz, 1H), 7.85 (d, *J* = 2.4 Hz, 1H), 7.74 (dd, *J* = 8.2, 0.7 Hz, 1H), 7.61 (dt, *J* = 1.5, 0.8 Hz, 1H), 7.38 (dd, *J* = 8.2, 1.5 Hz, 1H), 7.13 (d, *J* = 3.1 Hz, 1H), 6.55 (dd, *J* = 3.1, 0.9 Hz, 1H), 3.85 (s, 3H).

^{13}C NMR (101 MHz, $CDCl_3$) δ 145.5, 144.7, 144.3, 144.0, 140.2, 136.8, 135.7, 131.6, 130.4, 130.1, 128.8, 127.1, 122.3, 120.7, 111.6, 101.2, 33.1.

HRMS (ESI) m/z: [M+H$^+$] Calculated for $C_{17}H_{13}ClN_3$ 294.0793; Found 294.0790.

NMR spectra are in agreement with the reported data.[12]

2-(1-Methyl-1*H*-indol-6-yl)isoindolin-1-one (**1ah**)

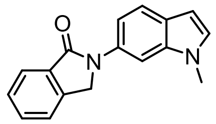

In a round bottom flask charged with a stir bar, ethyl 2-bromobenzoate (243 mg, 1.0 mmol, 1.0 equiv.) was dissolved in ethanol (12 mL), and 1-methyl-1*H*-indol-6-amine (146 mg, 1.2 mmol, 1.2 equiv.) as well as $(^iPr)_2NEt$ (0.21 mL, 1.2 mmol, 1.2 equiv.) were added to the mixture, which was stirred at 110 °C for 16 hours. The mixture was then concentrated under reduced pressure. Flash column chromatography (pentane:EA = 80:20 eluent) afforded the product **1ah** as a an off-white solid in 24% yield (64 mg).

^{1}H NMR (400 MHz, $CDCl_3$) δ 8.15 (dt, J = 1.8, 0.8 Hz, 1H), 7.99 – 7.91 (m, 1H), 7.68 – 7.48 (m, 4H), 7.30 (dd, J = 8.5, 2.0 Hz, 1H), 7.07 (d, J = 3.1 Hz, 1H), 6.47 (dd, J = 3.1, 0.9 Hz, 1H), 4.93 (d, J = 0.8 Hz, 2H), 3.81 (s, 3H).

^{13}C NMR (101 MHz, $CDCl_3$) δ 167.6, 140.5, 137.0, 134.0, 133.7, 131.9, 129.7, 128.4, 126.0, 124.1, 122.7, 121.2, 112.5, 102.2, 100.9, 52.0, 33.1.

HRMS (ESI) m/z: [M+H$^+$] Calculated for $C_{17}H_{15}N_2O$ 263.1179; Found 263.1173.

1-(1-Methyl-1*H*-indol-4-yl)-3-(pyridin-2-yl)urea (**1ai**)

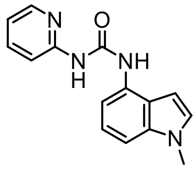

In 12 mL vials, pyridin-2-amine (122 mg, 1 equiv, 1.30 mmol) was dissolved in DCM (5.20 mL). Triethylamine (197 mg, 272 µL, 1.5 equiv, 1.95 mmol) and *N,N'*-carbonyldiimidazole (316 mg, 202 µL, 1.5 equiv, 1.95 mmol) was added. The mixture was stirred at rt for 2 h before 1-methyl-1*H*-indol-4-amine (190 mg, 1 equiv, 1.30 mmol) was added. The reaction was stirred for 16 h at rt. Brine was added to the mixture and it was extracted with DCM three times. The combined organic layers were dried over anhydrous $MgSO_4$, filtered and the solvent was removed under reduced pressure. The crude material was purified by flash column chromatography on silica gel (0 to 100% EA in hexanes) to obtain 1-(1-methyl-1*H*-indol-4-yl)-3-(pyridin-2-yl)urea (**1ai**) as orange solid in 60% yield (206 mg).

^{1}H NMR (600 MHz, $CDCl_3$) δ 8.37 (dq, *J* = 4.9, 1.7 Hz, 1H), 7.92 (d, *J* = 7.7 Hz, 1H), 7.70 – 7.66 (m, 1H), 7.26 (t, *J* = 8.0 Hz, 1H), 7.11 (dt, *J* = 8.2, 0.9 Hz, 1H), 7.06 (d, *J* = 3.1 Hz, 1H), 7.01 – 6.91 (m, 2H), 6.68 (d, *J* = 3.1 Hz, 1H), 3.82 (s, 3H).

^{13}C NMR (151 MHz, $CDCl_3$) δ 153.6, 153.2, 152.5, 138.6, 137.5, 131.2, 128.1, 122.6, 120.8, 117.3, 112.5, 110.0, 105.0, 97.9, 33.2.

HRMS (ESI) m/z: [M+H$^+$] Calculated for $C_{15}H_{15}N_4O$ 267.124; Found 267.1237.

Methyl 4-((diethylcarbamoyl)oxy)benzoate (**SI-1**)

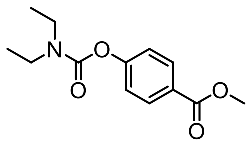

To a solution of methyl 4-hydroxybenzoate (1.0 g, 1 equiv, 6.6 mmol) in DMF (25 mL) was added K_2CO_3 (1.8 g, 2 equiv, 13 mmol). The mixture was stirred for 1 h at rt before *N,N*-diethylcarbamylchloride (0.98 g, 0.92 mL, 1.1 equiv, 7.3 mmol) was added. After stirring for 24 h at rt, the mixture was diluted with water, and it was extracted 3 x with DCM. The organic layer was dried over anhydrous $MgSO_4$, filtered and the solvent was removed under reduced pressure. The crude product was purified by flash column chromatography on silica gel (0 to 100% EA in hexanes) to afford methyl 4-((diethylcarbamoyl)oxy)benzoate (**SI-1**) as colorless oil in 98% yield (1.63 g).

^{1}H NMR (400 MHz, $CDCl_3$) δ 8.07 – 8.01 (m, 2H), 7.23 – 7.16 (m, 2H), 3.90 (s, 3H), 3.41 (dq, *J* = 20.1, 7.1 Hz, 4H), 1.23 (dt, *J* = 18.4, 7.1 Hz, 6H).

^{13}C NMR (101 MHz, $CDCl_3$) δ 166.7, 155.5, 153.6, 131.1, 127.0, 121.7, 52.2, 42.5, 42.1, 14.4, 13.5.

HRMS (ESI) m/z: [M+Na$^+$] Calculated for $C_{13}H_{17}NNaO_4$ 274.1050; Found 274.1051.

NMR spectra are in agreement with the reported data.[13]

4-((Diethylcarbamoyl)oxy)benzoic acid (**SI-2**)

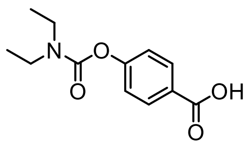

NaOH (456 mg, 5.70 mL, 2 molar, 3.8 equiv, 11.4 mmol) was added to a solution of methyl 4-((diethylcarbamoyl)oxy)benzoate (**SI-1**, 754 mg, 1 equiv, 3.00 mmol) in MeOH (6.00 mL). The reaction was stirred for 12 h at rt before the pH of the mixture was adjusted to 3 with 2 M HCl. The solvent was removed in vacuo and the aqueous mixture was stored at 4 °C for 12 hr. The formed precipitate was collected by vacuum filtration and washed with cyclohexane to afford 4-((diethylcarbamoyl)oxy)benzoic acid (**SI-2**) as white needles in 80% yield (566 mg).

^{1}H NMR (500 MHz, DMSO-d_6) δ 7.97 – 7.93 (m, 2H), 7.26 – 7.21 (m, 2H), 1.16 (dt, *J* = 38.3, 7.1 Hz, 6H). -N-CH_2-Me (δ 3.32) signal is not visible due to the water signal.

^{13}C NMR (126 MHz, DMSO-d_6) δ 166.8, 154.9, 152.8, 130.8, 127.6, 121.9, 41.9, 41.7, 14.2, 13.3.

HRMS (ESI) m/z: [M+Na$^+$] Calculated for $C_{12}H_{15}NNaO_4$ 260.0893; Found 260.0891.

NMR spectra are in agreement with the reported data.[13]

4-((1*H*-indol-7-yl)carbamoyl)phenyl diethylcarbamate (**SI-3**)

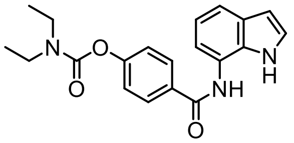

To a solution of 4-((diethylcarbamoyl)oxy)benzoic acid (**SI-2**, 308 mg, 1 equiv, 1.30 mmol) in DMF (8.13 mL) at 0 °C was added DMAP (175 mg, 1.1 equiv, 1.43 mmol) and 1-(3-dimethylaminopropyl)-3-ethylcarbodiimide hydrochloride (EDCI) (249 mg, 1 equiv, 1.30 mmol). It was stirred at 0 °C for 1 h before 1*H*-indol-7-amine (189 mg, 1.1 equiv, 1.43 mmol) was added. The mixture was slowly warmed to RT, and it was stirred overnight. 1 M HCl was added, and it was extracted with DCM three times. The organic layer was dried over anhydrous $MgSO_4$, filtered and the solvent was removed under reduced pressure. The crude product was purified by column chromatography on silica gel (0 to 100% EA in hexanes) to afford 4-((1H-indol-7-yl)carbamoyl)phenyl diethylcarbamate (**SI-3**) as a light pink solid in 79% yield (361 mg).

^{1}H NMR (500 MHz, $CDCl_3$) δ 9.93 (s, 1H), 7.92 – 7.88 (m, 2H), 7.53 (d, *J* = 7.8 Hz, 1H), 7.28 – 7.21 (m, 3H), 7.05 (t, *J* = 7.7 Hz, 1H), 6.91 (dd, *J* = 7.5, 0.9 Hz, 1H), 6.57 (dd, *J* = 3.1, 2.0 Hz, 1H), 3.44 (dq, *J* = 25.7, 7.1 Hz, 4H), 1.33 – 1.18 (m, 6H).

^{13}C NMR (126 MHz, $CDCl_3$) δ 165.2, 154.7, 153.8, 131.2, 131.0, 128.8, 128.35, 125.2, 122.7, 122.3, 119.5, 118.5, 113.6, 102.7, 42.6, 42.2, 14.4, 13.5.

HRMS (ESI) m/z: [M+H$^+$] Calculated for $C_{20}H_{22}N_3O_3$ 352.1656; Found 352.1648.

NMR spectra are in agreement with the reported data.[14]

4-(Methyl(1-methyl-1*H*-indol-7-yl)carbamoyl)phenyl diethylcarbamate (**1al**)

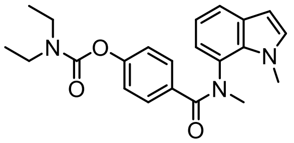

4-(Methyl(1-methyl-1*H*-indol-7-yl)carbamoyl)phenyl diethylcarbamate (**1al**) was prepared according to General Procedure A using 4-((1*H*-indol-7-yl)carbamoyl)phenyl diethylcarbamate (**SI-3**) (360 mg) as starting material with methyl iodide (96 µL) as alkylating agent. The product was obtained as yellow solid in 65% yield (254 mg).

^{1}H NMR (500 MHz, $CDCl_3$) δ 7.48 (dd, *J* = 7.9, 1.1 Hz, 1H), 7.34 – 7.30 (m, 2H), 6.98 (d, *J* = 3.2 Hz, 1H), 6.92 (t, *J* = 7.7 Hz, 1H), 6.86 – 6.81 (m, 3H), 6.47 (d, *J* = 3.1 Hz, 1H), 3.92 (s, 3H), 3.48 (s, 3H), 3.34 – 3.28 (m, 4H), 1.14 (dt, *J* = 10.1, 7.2 Hz, 6H).

^{13}C NMR (126 MHz, $CDCl_3$) δ 170.2, 153.6, 152.7, 131.8, 131.7, 131.6, 131.2, 129.9, 129.3, 122.7, 121.2, 120.9, 120.0, 102.1, 42.3, 42.0, 40.6, 35.1, 14.2, 13.4.

HRMS (ESI) m/z: [M+H$^+$] Calculated for $C_{22}H_{26}N_3O_3$ 380.1969; Found 380.1965.

2-Chloro-5-(1-methyl-1*H*-indol-6-yl)pyridin-3-amine (**SI-4**)

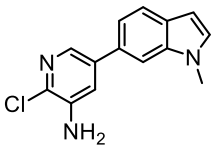

In a two-necked round bottom flask charged with a stir bar, 1-methyl-6-(4,4,5,5-tetramethyl-1,3,2-dioxaborolan-2-yl)-1*H*-indole (386 mg, 1.50 mmol, 1.33 equiv.) was dissolved in dioxane (12 mL) under N_2 atmosphere. 5-bromo-2-chloropyridin-3-amine (233 mg, 1.13 mmol, 1.0 equiv.), K_2CO_3 (394 mg, 2.85 mmol, 2.5 equiv.), $Pd(PPh_3)_4$ (31 mg, 0.027 mmol, 0.024 equiv.) and water (2.5 mL) were added to the mixture, which was stirred at 80 °C for 16 hours. The mixture was then concentrated under reduced pressure. The residue was then dissolved in ethyl acetate, washed with water, and the organic phase was dried over Na_2SO_4, filtered, and concentrated. Flash column chromatography (pentane:EA = 80:20 to 50:50 eluent) afforded the product **SI-4** as a pale brown solid in 52% yield (151 mg).

^{1}H NMR (400 MHz, $CDCl_3$) δ 8.11 (d, J = 2.2 Hz, 1H), 7.69 (dd, J = 8.2, 0.7 Hz, 1H), 7.45 (dt, J = 1.6, 0.8 Hz, 1H), 7.30 (d, J = 2.2 Hz, 1H), 7.29 – 7.25 (m, 1H), 7.11 (d, J = 3.1 Hz, 1H), 6.52 (dd, J = 3.1, 0.9 Hz, 1H), 3.84 (s, 3H).

^{13}C NMR (101 MHz, $CDCl_3$) δ 139.4, 138.5, 137.5, 137.2, 135.4, 130.7, 130.2, 128.6, 121.6, 121.3, 119.0, 107.9, 101.1, 33.1.

HRMS (ESI) m/z: [M+H$^+$] Calculated for $C_{14}H_{12}ClN_3$ 258.0793; Found 258.0792.

N-(2-chloro-5-(1-methyl-1*H*-indol-6-yl)pyridin-3-yl)benzenesulfonamide (**1ao**) and *N*-(2-chloro-5-(1-methyl-1*H*-indol-6-yl)pyridin-3-yl)-*N*-(phenylsulfonyl)benzenesulfonamide (**SI-5**)

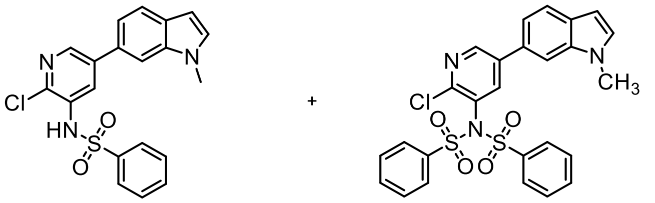

In a two-necked round bottom flask charged with a stir bar, 2-chloro-5-(1-methyl-1*H*-indol-6-yl)pyridin-3-amine (**SI-4**, 151 mg, 0.59 mmol, 1.0 equiv.) was dissolved in pyridine (5 mL) under N_2 atmosphere. Benzenesulfonyl chloride (0.15 mL, 1.18 mmol, 2.0 equiv.) was added to this mixture, which was then stirred at 50 °C for 16 hours. The reaction was quenched with saturated aq. $NaHCO_3$ solution, extracted with EA, then the combined organic phases were dried over Na_2SO_4, filtered, and concentrated. Flash column chromatography (DCM:MeOH = 100:0 to 95:5 eluent) afforded *N*-(2-chloro-5-(1-methyl-1*H*-indol-6-yl)pyridin-3-yl)benzenesulfonamide (**1ao**) as an off-white solid in 37% yield (86 mg) and *N*-(2-chloro-5-(1-methyl-1*H*-indol-6-yl)pyridin-3-yl)-*N*-(phenylsulfonyl)benzenesulfonamide (**SI-5**) as a white solid in 5% yield (169 mg).

*Spectral data for N-(2-chloro-5-(1-methyl-1H-indol-6-yl)pyridin-3-yl)benzenesulfonamide (**1ao**):*

^{1}H NMR (400 MHz, $CDCl_3$) δ 8.43 (d, J = 2.3 Hz, 1H), 8.28 (d, J = 2.3 Hz, 1H), 7.88 – 7.80 (m, 2H), 7.72 (dd, J = 8.2, 0.7 Hz, 1H), 7.63 – 7.54 (m, 1H), 7.52 – 7.43 (m, 3H), 7.33 – 7.25 (m, 1H), 7.15 (d, J = 3.1 Hz, 1H), 7.13 – 7.09 (m, 1H), 6.54 (dd, J = 3.1, 0.9 Hz, 1H), 3.87 (s, 3H).

^{13}C NMR (101 MHz, $CDCl_3$) δ 144.0, 140.3, 138.7, 138.5, 137.2, 133.8, 130.7, 130.5, 129.5, 129.2, 129.1, 128.8, 127.3, 121.8, 118.8, 108.0, 101.2, 33.1.

HRMS (ESI) m/z: [M+H$^+$] Calculated for $C_{20}H_{17}ClN_3O_2S$ 398.0725; Found 398.0724.

*Spectral data for N-(2-chloro-5-(1-methyl-1H-indol-6-yl)pyridin-3-yl)-N-(phenylsulfonyl)benzenesulfonamide (**SI-5**):*

^{1}H NMR (400 MHz, $CDCl_3$) δ 8.76 (d, J = 2.3 Hz, 1H), 8.09 – 8.01 (m, 4H), 7.77 – 7.67 (m, 4H), 7.64 – 7.55 (m, 4H), 7.37 (dt, J = 1.6, 0.8 Hz, 1H), 7.19 (dd, J = 8.2, 1.6 Hz, 1H), 7.15 (d, J = 3.1 Hz, 1H), 6.54 (dd, J = 3.1, 0.8 Hz, 1H), 3.85 (s, 3H).

^{13}C NMR (101 MHz, $CDCl_3$) δ 151.0, 149.0, 140.4, 139.2, 138.2, 137.2, 134.6, 130.8, 129.3, 129.3, 129.2, 129.2, 128.3, 121.9, 118.7, 108.0, 101.3, 33.2.

HRMS (ESI) m/z: [M+H$^+$] Calculated for $C_{26}H_{21}ClN_3O_4S_2$ 538.0657; Found 538.0650.

4-(2,6-Dichloropyrimidin-4-yl)morpholine (**SI-6**)

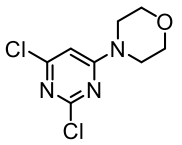

A 100 mL round-bottom flask, equipped with a stir bar, was charged with 2,4,6-trichloropyrimidine (1.83 g, 1 equiv, 10.0 mmol) and DCM (24 mL). At 0°C, triethylamine (1.01 g, 1.39 mL, 1 equiv, 10.0 mmol) and morpholine (871 mg, 871 µL, 1 Eq, 10.0 mmol) in DCM (16 mL), was slowly added. The reaction was stirred for 30 min at 0°C. The reaction mixture was diluted with H_2O (40 mL) and extracted with ethyl acetate (3 x 160 mL). The combined organic layers were dried over $MgSO_4$, filtered, and concentrated under reduced pressure. The product was recrystallized from DCM and pentane. The white crystals were filtered and washed with cold pentane (50 mL). The product was dried overnight. 4-(2,6-dichloropyrimidin-4-yl)morpholine (**SI-6**) was obtained as a white solid in 47% yield (1.11 g).

^{1}H NMR (400 MHz, $CDCl_3$) δ 6.40 (s, 1H), 3.80 – 3.73 (m, 4H), 3.64 (s, 4H).

^{13}C NMR (101 MHz, $CDCl_3$) δ 163.4, 160.9, 160.1, 99.9, 66.4, 44.7.

HRMS (ESI) m/z: [M+H$^+$] Calculated for $C_8H_{10}Cl_2N_3O$ 234.0195; Found 234.0192.

NMR spectra are in agreement with the reported data.[15]

4-(6-Chloro-2-(1-methyl-1*H*-indol-4-yl)pyrimidin-4-yl)morpholine (**1ar**)

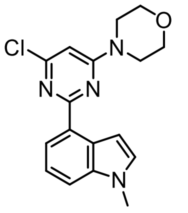

A 20 mL Schlenk tube, equipped with a stir bar, was charged under nitrogen with 4-(2,6-dichloropyrimidin-4-yl)morpholine (**SI-6**, 2.69 g, 1.8 equiv, 9.19 mmol), 4-(4,4,5,5-tetramethyl-1,3,2-dioxaborolan-2-yl)-1*H*-indole (1.22 g, 1.0 equiv, 5.00 mmol), 1,4-dioxane (6.75 mL), water (0.75 mL), tetrakis(triphenylphosphine)palladium(0) (1.16 g, 0.2 equiv, 1.00 mmol) and potassium carbonate (1.38 g, 2.0 equiv, 10.00 mmol). The reaction stirred under nitrogen at 110 °C for 2 h. The reaction was filtered through celite. Water (20 mL) was added and extracted with ethyl acetate (3 x 50 mL). The product was purified by flash column chromatography (0 - 100% EA in hexanes). NMR analysis showed that there was the product and an isomer. Separation of the isomers was only performed after the methylation (see below).

A 50 mL round-bottom flask, equipped with a stir bar, was charged with 4-(6-chloro-2-(1*H*-indol-4-yl)pyrimidin-4-yl)morpholine (570 mg, 1 equiv, 1.81 mmol) and THF (18 mL). NaH (109 mg, 60% Wt, 1.5 equiv, 2.72 mmol) was added at 0°C. After 15 min methyl iodide (386 mg, 176 µL, 1.5 equiv, 2.72 mmol) was added. The reaction stirred for 30 min at rt. The reaction was quenched with 18 mL saturated, aqueous $NaHCO_3$ solution and 18 mL H_2O. The reaction mixture was extracted with Et_2O (3 x 50 mL). The solvent was removed under reduced pressure. The product was purified by flash column chromatography (0 - 40% EA in hexanes). 4-(6-chloro-2-(1-methyl-1*H*-indol-4-yl)pyrimidin-4-yl)morpholine (**1ar**) was obtained as a white solid in 33% yield (195 mg, 65% purity). The purity of 65% was determined by quantitative ^{1}H NMR, using 8.1 mg of substrate and 10 µL of CH_2Br_2.

^{1}H NMR (400 MHz, $CDCl_3$) δ 8.19 (dd, *J* = 7.5, 1.0 Hz, 1H), 7.45 (dt, *J* = 8.1, 0.9 Hz, 1H), 7.37 (dd, *J* = 3.1, 0.9 Hz, 1H), 7.31 (t, *J* = 7.8 Hz, 1H), 7.17 (d, *J* = 3.1 Hz, 1H), 6.42 (s, 1H), 3.87 – 3.81 (m, 7H), 3.77 – 3.74 (m, 4H).

^{13}C NMR (101 MHz, $CDCl_3$) δ 165.8, 163.3, 160.5, 137.9, 130.2, 129.1, 127.3, 122.0, 121.3, 112.2, 102.8, 98.8, 66.6, 44.7, 33.2.

HRMS (ESI) m/z: [M+H$^+$] Calculated for $C_{17}H_{18}ClN_4O$ 329.1164; Found 329.1163.

N-(2-formylphenyl)-*N*-methylformamide (**Ia**)

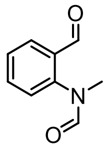

N-(2-formylphenyl)-*N*-methylformamide (**Ia**) was prepared as described in literature[16] from 1-methyl-1*H*-indole (3.00 mmol, 416 mg) and was obtained as orange oil in 69% yield (358 mg).

^{1}H NMR (400 MHz, $CDCl_3$) δ 10.11 (d, *J* = 0.6 Hz, 1H), 8.25 (d, *J* = 0.7 Hz, 1H), 7.98 (ddd, *J* = 7.7, 1.7, 0.5 Hz, 1H), 7.70 (ddd, *J* = 7.9, 7.4, 1.7 Hz, 1H), 7.56 – 7.51 (m, 1H), 7.30 (ddd, *J* = 7.9, 1.2, 0.5 Hz, 1H), 3.35 (d, *J* = 0.5 Hz, 3H).

^{13}C NMR (101 MHz, $CDCl_3$) δ 189.2, 162.9, 143.7, 135.5, 132.0, 131.1, 128.6, 128.0, 34.8.

HRMS (ESI) m/z: [M+Na$^+$] Calculated for $C_9H_9NNaO_2$ 186.0525; Found 186.0526.

NMR spectra are in agreement with the reported data.[16]

2-(*N*-methylformamido)benzamide (**IIa**)

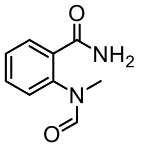

2-(*N*-methylformamido)benzamide (**IIa**) was prepared as described in literature[17] from 2-(methylamino)benzamide (1.00 mmol, 150 mg) and was obtained as white solid in 45% yield (80 mg).

^{1}H NMR (400 MHz, DMSO-d_6) δ 8.09 (d, *J* = 0.6 Hz, 1H), 7.85 (s, 1H), 7.56 – 7.47 (m, 3H), 7.43 – 7.34 (m, 2H), 3.11 (d, *J* = 0.6 Hz, 3H).

^{13}C NMR (101 MHz, DMSO-d_6) δ 169.1, 162.2, 139.1, 134.8, 130.6, 128.5, 127.4, 127.3, 32.9.

HRMS (ESI) m/z: [M+Na$^+$] Calculated for $C_9H_{10}N_2NaO_2$ 201.0634; Found 201.0635.

1-Methyl-1,4-dihydroquinazolin-4-ol (**VIIIa**)

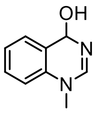

A 12 mL vial was charged with a stirring bar. *N*-(2-formylphenyl)-*N*-methylformamide **Ia** (49 mg, 1.0 equiv, 0.30 mmol) was dissolved in the methanol (3 mL). Ammonium carbamate (140 mg, 6 equiv, 1.8 mmol) was added and the reaction mixture was stirred for 90 min at 50 °C. After cooling to rt, the volatiles were removed under reduced pressure and the crude product was prufiied by column chromatography on silica gel. The product **VIIIa** was obtained as yellowish oil in 86% yield (50 mg).

^{1}H NMR (500 MHz, DMSO) δ 7.48 – 7.45 (m, 1H), 7.41 – 7.31 (m, 1H), 7.28 (ddt, *J* = 7.6, 1.6, 0.6 Hz, 1H), 7.14 (td, *J* = 7.5, 1.1 Hz, 1H), 6.99 (dd, *J* = 8.2, 1.1 Hz, 1H), 5.71 (s, 1H), 3.36 (br s, 1H), 3.29 (s, 3H).

^{13}C NMR (126 MHz, DMSO) δ 147.9, 136.8, 128.9, 128.1, 123.2, 118.5, 111.6, 83.2, 34.8.

HRMS (ESI) m/z: [M+H$^+$] Calculated for $C_9H_{11}N_2O$ 163.0866; Found 163.0868.

5 SUBSTRATE SCOPE

General Procedure B

The alkylated indole (0.25 mmol, 1.0 equiv) was added to a 150 mL pressure tube. Ammonium carbamate (332 mg, 17.0 equiv) and methanol (30 mL) were added. The tube was sealed and stirred at rt at 600 rpm for 15 min. The stirring was stopped and PIDA (483 mg, 6 equiv) was added. The tube was sealed again, and the mixture was stirred for an additional 10 min. The solvent was removed under reduced pressure, and the crude product was purified by column chromatography on silica gel (0 to 100% EA in hexanes, then 0 to 20% MeOH in DCM).

1-Methyl-1*H*-benzo[*d*]imidazole (**2a**)

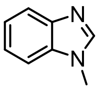

1-Methyl-1*H*-benzo[*d*]imidazole (**2a**) was prepared according to General Procedure B using 1-methyl-1*H*-indole (**1a**, 32.8 mg) as starting material. The product was obtained as a dark red solid in 45% yield (15 mg).

Scale up to 1 mmol: 1-methyl-1*H*-indole (**1a**, 131 mg) and ammonium carbamate (1.33 g) were added to a 250 mL flask, dissolved in 60 mL methanol and cooled to 0 °C. PIDA (1.93 g) was dissolved in 60 mL methanol and cooled to 0 °C. The PIDA solution was added to the ice-cooled solution of starting material and ammonium carbamate under vigorous stirring. The mixture was stirred for 30 min at 0 °C and then another 30 min at rt. The product was obtained as a dark red solid after flash column chromatography on silica gel in 40% yield (53 mg).

^{1}H NMR (500 MHz, DMSO-d_6) δ 8.17 (s, 1H), 7.65 – 7.63 (m, 1H), 7.56 (ddd, *J* = 8.0, 1.2, 0.7 Hz, 1H), 7.26 (ddd, *J* = 8.1, 7.1, 1.1 Hz, 1H), 7.22 – 7.18 (m, 1H), 3.83 (d, *J* = 0.4 Hz, 3H).

^{13}C NMR (126 MHz, DMSO-d_6) δ 144.6, 143.3, 134.6, 122.2, 121.4, 119.3, 110.2, 30.6.

HRMS (ESI) m/z: [M+H$^+$] Calculated for $C_8H_9N_2$ 133.0760; Found 133.0763.

NMR spectra are in agreement with the reported data.[18]

1-Methyl-1*H*-benzo[*d*]imidazole-2-^{13}C (**^{13}C-2a**)

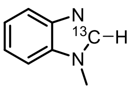

1-Methyl-1*H*-benzo[*d*]imidazole-2-^{13}C (**^{13}C-2a**) was prepared according to General Procedure B using 1-methyl-1*H*-indole-2-^{13}C (**^{13}C-1a**, 33 mg) as starting material. The product was obtained as a brown solid in 30% yield (10 mg).

^{1}H NMR (600 MHz, d_4-MeOH) δ 8.10 (d, *J* = 206.6 Hz, 1H), 7.66 (dt, *J* = 8.1, 1.0 Hz, 1H), 7.55 (dt, *J* = 8.1, 1.0 Hz, 1H), 7.34 (ddd, *J* = 8.2, 7.1, 1.1 Hz, 1H), 7.28 (ddd, *J* = 8.2, 7.1, 1.1 Hz, 1H), 3.90 (d, *J* = 3.5 Hz, 3H).

^{13}C NMR (151 MHz, d_4-MeOH) δ 145.2, 143.9 (d, *J* = 9.8 Hz), 135.8 (d, *J* = 8.0 Hz), 124.3, 123.5, 119.9 (d, *J* = 6.8 Hz), 111.1 (d, *J* = 3.4 Hz), 31.3 (d, *J* = 2.7 Hz).

HRMS (ESI) m/z: [M+H$^+$] Calculated for $C_7{}^{13}CH_9N_2$ 134.0794; Found 134.0794.

4-Amino-1-methylquinazolin-1-ium 2,2,2-trifluoroacetate (**3a**)

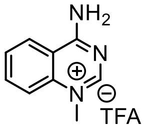

4-Amino-1-methylquinazolin-1-ium 2,2,2-trifluoroacetate (**3a**) was isolated as a side product from the reaction with 1-methyl-1*H*-indole **1a**. A sample was purified by preparative HPLC (1-70% ACN in H_2O with 0.1% TFA).

^{1}H NMR (500 MHz, d_4-MeOH) δ 8.73 (s, 1H), 8.42 (dd, *J* = 8.3, 1.3 Hz, 1H), 8.14 (ddd, *J* = 8.6, 7.2, 1.4 Hz, 1H), 8.01 – 7.95 (m, 1H), 7.82 (ddd, *J* = 8.2, 7.2, 1.0 Hz, 1H), 7.44 (br s, 2H), 4.06 (s, 3H).

^{19}F NMR (471 MHz, d_4-MeOH) δ -76.92.

^{13}C NMR (126 MHz, d_4-MeOH) δ 165.56, 155.23, 140.60, 138.25, 130.01, 126.64, 118.52, 113.99, 39.32.

HRMS (ESI) m/z: [M $^+$] Calculated for $C_9H_{10}N_3$ 160.0869; Found 160.0870.

6-Methoxy-1-methyl-1*H*-benzo[*d*]imidazole (**2b**)

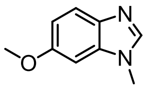

6-Methoxy-1-methyl-1*H*-benzo[*d*]imidazole (**2b**) was prepared according to General Procedure B using 6-methoxy-1-methyl-1*H*-indole (**1b**, 40.3 mg) as starting material. The product was obtained as a light orange oil in 78% yield (32 mg).

^{1}H NMR (400 MHz, d_4-MeOH) δ 7.97 (s, 1H), 7.51 (d, *J* = 8.8 Hz, 1H), 7.02 (d, *J* = 2.4 Hz, 1H), 6.90 (dd, *J* = 8.9, 2.4 Hz, 1H), 3.86 (s, 3H), 3.83 (s, 3H).

^{13}C NMR (101 MHz, d_4-MeOH) δ 158.6, 144.3, 138.1, 136.4, 120.4, 113.3, 94.1, 56.3, 31.3.

HRMS (ESI) m/z: [M+H$^+$] Calculated for $C_9H_{11}N_2O$ 163.0866; Found 163.0863.

NMR spectra are in agreement with the reported data.[19]

1-Ethyl-6-methoxy-1*H*-benzo[*d*]imidazole (**2c**)

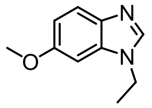

1-Ethyl-6-methoxy-1*H*-benzo[*d*]imidazole (**2c**) was prepared according to General Procedure B using 1-ethyl-6-methoxy-1*H*-indole (**1c**, 43.8 mg) as starting material. The product was obtained as brown oil in 50% yield (22.1 mg).

^{1}H NMR (400 MHz, *d_4*-MeOH) δ 8.04 (s, 1H), 7.52 (dd, *J* = 8.9, 0.5 Hz, 1H), 7.05 (d, *J* = 2.4 Hz, 1H), 6.90 (dd, *J* = 8.8, 2.4 Hz, 1H), 4.27 (q, *J* = 7.3 Hz, 2H), 3.86 (s, 3H), 1.49 (t, *J* = 7.3 Hz, 3H).

^{13}C NMR (101 MHz, *d_4*-MeOH) δ 158.5, 143.2, 138.4, 135.4, 120.5, 113.3, 94.3, 56.3, 40.8, 15.5.

HRMS (ESI) m/z: [M+H$^+$] Calculated for $C_{10}H_{13}N_2O$ 177.1022; Found 177.1019.

NMR spectra are in agreement with the reported data.[19]

1-Isopropyl-6-methoxy-1*H*-benzo[*d*]imidazole (**2d**)

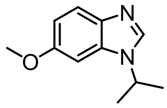

1-Isopropyl-6-methoxy-1*H*-benzo[*d*]imidazo (**2d**) was prepared according to General Procedure B using 1-isopropyl-6-methoxy-1*H*-indole (**1d**, 47.3 mg) as starting material. The product was obtained as brown oil in 61% yield (28.9 mg).

^{1}H NMR (500 MHz, *d_4*-MeOH) δ 8.15 (s, 1H), 7.55 – 7.50 (m, 1H), 7.07 (d, *J* = 2.3 Hz, 1H), 6.91 (dd, *J* = 8.8, 2.4 Hz, 1H), 4.72 (hept, *J* = 6.8 Hz, 1H), 3.87 (s, 3H), 1.60 (d, *J* = 6.7 Hz, 6H).

^{13}C NMR (126 MHz, *d_4*-MeOH) δ 158.4, 140.9, 138.3, 135.0, 120.5, 113.3, 94.9, 56.3, 22.6. -N-*C*-(Me)$_2$ (δ 49) signal is not visible due to overlap with solvent peak.

HRMS (ESI) m/z: [M+H$^+$] Calculated for $C_{11}H_{15}N_2O$ 191.1179; Found 191.1179.

1-Allyl-6-methoxy-1*H*-benzo[*d*]imidazole (**2e**)

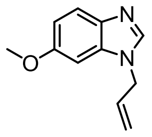

1-Allyl-6-methoxy-1*H*-benzo[*d*]imidazole (**2e**) was prepared according to General Procedure B using 1-allyl-6-methoxy-1*H*-indole (**1e**, 46.8 mg) as starting material. The product was obtained as brown oil in 46% yield (21.7 mg).

^{1}H NMR (400 MHz, *d_4*-MeOH) δ 8.02 (s, 1H), 7.53 (d, *J* = 8.8 Hz, 1H), 6.99 (d, *J* = 2.3 Hz, 1H), 6.90 (dd, *J* = 8.8, 2.4 Hz, 1H), 6.06 (ddt, *J* = 17.1, 10.6, 5.4 Hz, 1H), 5.26 (dq, *J* = 10.3, 1.4 Hz, 1H), 5.13 (dq, *J* = 17.0, 1.6 Hz, 1H), 4.86 (dt, *J* = 5.5, 1.7 Hz, 2H), 3.83 (s, 3H).

^{13}C NMR (101 MHz, *d_4*-MeOH) δ 158.6, 143.8, 138.3, 135.7, 134.0, 120.5, 118.3, 113.4, 94.7, 56.2, 48.1.

HRMS (ESI) m/z: [M+H$^+$] Calculated for $C_{11}H_{13}N_2O$ 189.1022; Found 189.1023.

1-Benzyl-6-methoxy-1*H*-benzo[*d*]imidazole (**2f**)

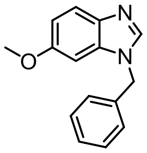

1-Benzyl-6-methoxy-1*H*-benzo[*d*]imidazole (**2f**) was prepared according to General Procedure B using 1-benzyl-6-methoxy-1*H*-indole (**1f**, 59.3 mg) as starting material. The product was obtained as brown solid in 58% yield (34.8 mg).

^{1}H NMR (400 MHz, *d_4*-MeOH) δ 8.11 (s, 1H), 7.64 – 7.48 (m, 1H), 7.42 – 7.18 (m, 5H), 6.89 (d, *J* = 7.8 Hz, 2H), 5.44 (s, 2H), 3.76 (s, 3H).

^{13}C NMR (101 MHz, *d_4*-MeOH) δ 158.6, 144.1, 138.5, 137.6, 135.7, 130.0, 129.1, 128.4, 120.62, 113.3, 94.9, 56.2, 49.5.

HRMS (ESI) m/z: [M+H$^+$] Calculated for $C_{15}H_{15}N_2O$ 239.1179; Found 239.1177.

NMR spectra are in agreement with the reported data.[19]

1-Benzyl-6-fluoro-1*H*-benzo[*d*]imidazole (**2g**)

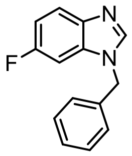

1-Benzyl-6-fluoro-1*H*-benzo[*d*]imidazole (**2g**) was prepared according to General Procedure B using 1-benzyl-6-fluoro-1*H*-indole (**1g**, 53.3 mg) as starting material. The product was obtained as brown solid in 54% yield (28.8 mg).

^{1}H NMR (500 MHz, *d_4*-MeOH) δ 8.27 (s, 1H), 7.64 (dd, *J* = 8.8, 4.7 Hz, 1H), 7.36 – 7.25 (m, 5H), 7.16 (dd, *J* = 8.8, 2.4 Hz, 1H), 7.03 (ddd, *J* = 9.6, 8.8, 2.4 Hz, 1H), 5.45 (s, 2H).

^{19}F NMR (471 MHz, *d_4*-MeOH) δ -120.20.

^{13}C NMR (126 MHz, *d_4*-MeOH) δ 161.2 (d, *J* = 239.3 Hz), 145.9, 140.7, 137.2, 135.2 (d, *J* = 13.2 Hz), 130.0, 129.3, 128.5, 121.2 (d, *J* = 10.2 Hz), 111.9 (d, *J* = 25.7 Hz), 98.4 (d, *J* = 28.1 Hz), 49.7.

HRMS (ESI) m/z: [M+H$^+$] Calculated for $C_{14}H_{12}FN_2$ 227,0979; Found 227.0980.

1-Benzyl-1*H*-benzo[*d*]imidazole (**SI-7**)

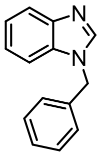

1-Benzyl-1*H*-benzo[*d*]imidazole (**SI-7**) was prepared according to General Procedure B using 1-benzyl-1*H*-indole (51.8 mg) as starting material. The product was obtained as a light brown solid in 47% yield (24.2 mg).

^{1}H NMR (500 MHz, *d_4*-MeOH) δ 8.25 (s, 1H), 7.70 – 7.65 (m, 1H), 7.43 – 7.38 (m, 1H), 7.35 – 7.22 (m, 7H), 5.47 (s, 2H).

^{13}C NMR (126 MHz, *d_4*-MeOH) δ 144.9, 144.2, 137.6, 135.0, 130.0, 129.1, 128.4, 124.3, 123.6, 120.2, 111.9, 49.6.

HRMS (ESI) m/z: [M+H$^+$] Calculated for $C_{14}H_{13}N_2$ 209.1073; Found 209.1077.

NMR spectra are in agreement with the reported data.[19]

1*H*-benzo[*d*]imidazole (**2h**)

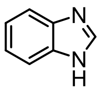

In an 8 mL vial under nitrogen atmosphere was put 1-benzyl-1*H*-benzo[*d*]imidazole (**SI-7**, 21 mg, 1 equiv, 0.10 mmol), Pd/C (2.1 mg, 10% Wt, 0.02 equiv, 2.0 µmol) and THF (0.50 mL). At rt triethylsilane (23 mg, 32 µL, 2 equiv, 0.20 mmol) was added. The mixture was stirred at rt overnight. The mixture was filtered through a pad of celite (eluent: DCM) and the filtrate was concentrated in vacuo. The crude product was purified by column chromatography on silica gel (0 to 100% MeOH in DCM) to afford 1*H*-benzo[*d*]imidazole (**2h**) as off-white solid in quantitative yield (12.1 mg).

^{1}H NMR (400 MHz, $CDCl_3$) δ 8.11 (s, 1H), 7.72 – 7.63 (m, 2H), 7.30 (dd, *J* = 6.1, 3.1 Hz, 2H).

NMR spectra are in agreement with the reported data[20] and an authentic sample.

6-Fluoro-1-methyl-1*H*-benzo[*d*]imidazole (**2i**)

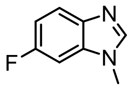

6-Fluoro-1-methyl-1*H*-benzo[*d*]imidazole (**2i**) was prepared according to General Procedure B using 6-fluoro-1-methyl-1*H*-indole (**1i**, 37.3 mg) as starting material. The product was obtained as a dark orange oil in 48% yield (18.1 mg).

^{1}H NMR (500 MHz, *d_4*-MeOH) δ 8.13 (s, 1H), 7.62 (dd, *J* = 8.8, 4.7 Hz, 1H), 7.31 (dd, *J* = 8.8, 2.5 Hz, 1H), 7.06 (ddd, *J* = 9.7, 8.8, 2.5 Hz, 1H), 3.86 (s, 3H).

^{19}F NMR (471 MHz, *d_4*-MeOH) δ -120.43.

^{13}C NMR (126 MHz, *d_4*-MeOH) δ 161.4 (d, *J* = 239.0 Hz), 146.1, 140.1, 136.0 (d, *J* = 13.7 Hz), 120.8 (d, *J* = 10.2 Hz), 111.8 (d, *J* = 25.7 Hz), 97.8 (d, *J* = 28.0 Hz), 31.5.

HRMS (ESI) m/z: [M+H$^+$] Calculated for $C_8H_8FN_2$ 151.0666; Found 151.0666.

NMR spectra are in agreement with the reported data.[21]

6-Chloro-1-methyl-1*H*-benzo[*d*]imidazole (**2j**)

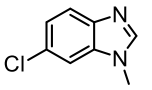

6-Chloro-1-methyl-1*H*-benzo[*d*]imidazole (**2j**) was prepared according to General Procedure B using 6-chloro-1-methyl-1*H*-indole (**1j**, 41.4 mg) as starting material. The product was obtained as white solid in 45% yield (18.8 mg).

^{1}H NMR (500 MHz, *d_4*-MeOH) δ 8.13 (s, 1H), 7.63 – 7.60 (m, 2H), 7.27 (dd, *J* = 8.7, 1.9 Hz, 1H), 3.88 (s, 3H).

^{13}C NMR (126 MHz, *d_4*-MeOH) δ 146.4, 142.6, 136.5, 130.1, 124.0, 121.0, 111.4, 31.5.

HRMS (ESI) m/z: [M+H$^+$] Calculated for $C_8H_8ClN_2$ 167.0371; Found 167.0370.

NMR spectra are in agreement with the reported data.[22]

6-Bromo-1-methyl-1*H*-benzo[*d*]imidazole (**2k**)

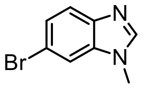

6-Bromo-1-methyl-1*H*-benzo[*d*]imidazole (**2k**) was prepared according to General Procedure B using 6-bromo-1-methyl-1*H*-indole (**1k**, 52.5 mg) as starting material. The product was obtained as yellow solid in 23% yield (12.1 mg).

^{1}H NMR (400 MHz, *d_4*-MeOH) δ 8.15 (s, 1H), 7.77 (dd, *J* = 1.9, 0.6 Hz, 1H), 7.57 (dd, *J* = 8.6, 0.6 Hz, 1H), 7.40 (dd, *J* = 8.6, 1.9 Hz, 1H), 3.88 (s, 3H).

^{13}C NMR (101 MHz, *d_4*-MeOH) δ 146.2, 142.7, 136.9, 126.8, 121.3, 117.4, 114.5, 31.5.

HRMS (ESI) m/z: [M+H$^+$] Calculated for $C_8H_8BrN_2$ 210.9865; Found 210.9867.

Methyl 1-methyl-1*H*-benzo[*d*]imidazole-6-carboxylate (**2l**)

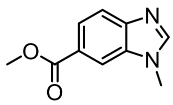

Methyl 1-methyl-1*H*-benzo[*d*]imidazole-6-carboxylate (**2l**) was prepared according to General Procedure B using methyl 1-methyl-1*H*-indole-6-carboxylate (47.3 mg) as starting material. The product was obtained as a light orange solid in 28% yield (13.2 mg).

^{1}H NMR (500 MHz, *d_4*-MeOH) δ 8.29 (s, 1H), 8.28 (dd, *J* = 1.6, 0.7 Hz, 1H), 7.98 (dd, *J* = 8.5, 1.6 Hz, 1H), 7.72 (dd, *J* = 8.5, 0.7 Hz, 1H), 3.96 (s, 3H), 3.95 (s, 3H).

^{13}C NMR (126 MHz, *d_4*-MeOH) δ 168.8, 148.2, 147.4, 135.6, 126.3, 124.7, 119.8, 113.5, 52.7, 31.6.

HRMS (ESI) m/z: [M+H$^+$] Calculated for $C_{10}H_{11}N_2O_2$ 191.0815; Found 191.0814.

NMR spectra are in agreement with the reported data.[23]

6-(Benzyloxy)-1-methyl-1*H*-benzo[*d*]imidazole (**2m**)

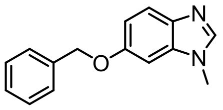

6-(Benzyloxy)-1-methyl-1*H*-benzo[*d*]imidazole (**2m**) was prepared according to General Procedure B using 6-(benzyloxy)-1-methyl-1*H*-indole (**1m**, 59.3 mg) as starting material. The product was obtained as brown solid in 61% yield (36.4 mg).

^{1}H NMR (500 MHz, *d4*-MeOH) δ 7.94 (s, 1H), 7.52 (d, *J* = 8.8 Hz, 1H), 7.45 (dd, *J* = 7.2, 1.8 Hz, 2H), 7.38 – 7.34 (m, 2H), 7.31 – 7.26 (m, 1H), 7.07 (d, *J* = 2.4 Hz, 1H), 6.97 (dd, *J* = 8.8, 2.4 Hz, 1H), 5.10 (s, 2H), 3.77 (s, 3H).

^{13}C NMR (126 MHz, *d4*-MeOH) δ 157.6, 144.4, 138.7, 138.3, 136.3, 129.5, 128.9, 128.7, 120.4, 113.9, 95.6, 71.7, 31.2.

HRMS (ESI) m/z: [M+H$^+$] Calculated for $C_{15}H_{15}N_2O$ 239.1179; Found 239.1179.

1-Methyl-6-(pyridin-2-yl)-1*H*-benzo[*d*]imidazole (**2n**)

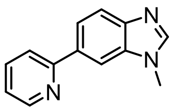

1-Methyl-6-(pyridin-2-yl)-1*H*-benzo[*d*]imidazole (**2n**) was prepared according to General Procedure B using 1-methyl-6-(pyridin-2-yl)-1*H*-indole (**1n**, 52.1 mg) as starting material. The product was obtained as white solid in 45% yield (23.3 mg).

^{1}H NMR (500 MHz, *d4*-MeOH) δ 8.63 (ddd, *J* = 4.9, 1.8, 1.0 Hz, 1H), 8.20 (d, *J* = 0.5 Hz, 1H), 8.17 (dd, *J* = 1.7, 0.7 Hz, 1H), 7.98 – 7.87 (m, 3H), 7.77 (dd, *J* = 8.5, 0.7 Hz, 1H), 7.37 (ddd, *J* = 7.3, 4.9, 1.3 Hz, 1H), 3.98 (s, 3H).

^{13}C NMR (126 MHz, *d4*-MeOH) δ 159.2, 150.3, 146.7, 144.8, 139.0, 136.3, 136.0, 123.5, 122.9, 122.8, 120.2, 110.1, 31.5.

HRMS (ESI) m/z: [M+H$^+$] Calculated for $C_{13}H_{12}N_3$ 210.1026; Found 210.1024.

6-(6-Chloro-2-methylpyrimidin-4-yl)-1-methyl-1*H*-benzo[*d*]imidazole (**2o**)

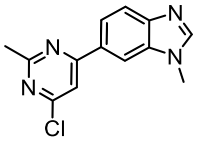

6-(6-Chloro-2-methylpyrimidin-4-yl)-1-methyl-1*H*-benzo[*d*]imidazole (**2o**) was prepared according to General Procedure B using 6-(6-chloro-2-methylpyrimidin-4-yl)-1-methyl-1*H*-indole (**1o**, 64.4 mg) as starting material. The product was obtained as brown solid in 20% yield (13.2 mg).

^{1}H NMR (500 MHz, *d4*-MeOH) δ 8.39 (dd, *J* = 1.7, 0.6 Hz, 1H), 8.23 (s, 1H), 8.07 (dd, *J* = 8.6, 1.7 Hz, 1H), 7.92 (d, *J* = 0.6 Hz, 1H), 7.74 (dd, *J* = 8.6, 0.7 Hz, 1H), 3.97 (s, 3H), 2.71 (s, 3H).

^{13}C NMR (126 MHz, *d$_4$*-MeOH) δ 170.1, 167.6, 162.7, 147.7, 146.5, 136.2, 131.9, 123.0, 120.4, 115.1, 111.1, 31.6, 25.8.

HRMS (ESI) m/z: [M+H$^+$] Calculated for $C_{13}H_{12}ClN_4$ 259.0745; Found 259.0742.

1-Methyl-6-(4-(methylsulfonyl)phenyl)-1*H*-benzo[*d*]imidazole (**2p**)

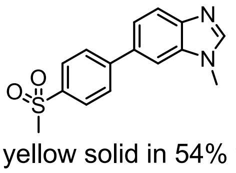

1-Methyl-6-(4-(methylsulfonyl)phenyl)-1*H*-benzo[*d*]imidazole (**2p**) was prepared according to General Procedure B using 1-methyl-6-(4-(methylsulfonyl)phenyl)-1*H*-indole (**1p**, 71.3 mg) as starting material. The product was obtained as a light yellow solid in 54% yield (38.7 mg).

^{1}H NMR 600 MHz, *d$_4$*-MeOH) δ 8.19 (s, 1H), 8.06 – 8.01 (m, 2H), 8.01 – 7.95 (m, 2H), 7.90 (dd, *J* = 1.8, 0.7 Hz, 1H), 7.77 (dd, *J* = 8.4, 0.7 Hz, 1H), 7.65 (dd, *J* = 8.4, 1.7 Hz, 1H), 3.97 (s, 3H), 3.17 (s, 3H).

^{13}C NMR (151 MHz, *d$_4$*-MeOH) δ 148.3, 146.6, 144.3, 140.4, 136.5, 135.9, 129.3, 129.0, 123.3, 120.6, 110.2, 44.5, 31.5.

HRMS (ESI) m/z: [M+H$^+$] Calculated for $C_{15}H_{15}N_2O_2S$ 287.0849; Found 287.0846.

5-Chloro-1-methyl-1*H*-benzo[*d*]imidazole (**2q**)

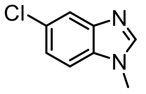

5-Chloro-1-methyl-1*H*-benzo[*d*]imidazole (**2q**) was prepared according to General Procedure B using 5-chloro-1-methyl-1*H*-indole (**1q**, 41.4 mg) as starting material. The product was obtained as a light yellow oil in 19% yield (7.9 mg).

^{1}H NMR (500 MHz, *d$_4$*-MeOH) δ 8.16 (s, 1H), 7.65 (dd, *J* = 1.9, 0.6 Hz, 1H), 7.54 (dd, *J* = 8.6, 0.6 Hz, 1H), 7.33 (dd, *J* = 8.7, 2.0 Hz, 1H), 3.90 (s, 3H).

^{13}C NMR (126 MHz, *d$_4$*-MeOH) δ 146.8, 144.8, 134.6, 129.3, 124.6, 119.6, 112.4, 31.5.

HRMS (ESI) m/z: [M+H$^+$] Calculated for $C_8H_8ClN_2$ 167.0371; Found 167.0371.

NMR spectra are in agreement with the reported data.[24]

5-Bromo-1-methyl-1*H*-benzo[*d*]imidazole (**2r**)

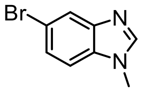

5-Bromo-1-methyl-1*H*-benzo[*d*]imidazole (**2r**) was prepared according to General Procedure B using 5-bromo-1-methyl-1*H*-indole (**1r**, 52.5 mg) as starting material. To achieve full conversion, monitored by ^{1}H-NMR after solvent evaporation, an additional

portion of the reagents and solvent was added (the General Procedure B was used again). The product was obtained as brown oil in 20% yield (8 mg).

^{1}H NMR (500 MHz, *d_4*-MeOH) δ 8.14 (s, 1H), 7.80 (d, *J* = 1.8 Hz, 1H), 7.53 – 7.40 (m, 2H), 3.89 (s, 3H).

^{13}C NMR (126 MHz, *d_4*-MeOH) δ 146.6, 145.3, 134.9, 127.2, 122.7, 116.4, 112.8, 31.5.

HRMS (ESI) m/z: [M+H$^+$] Calculated for $C_8H_8BrN_2$ 210.9865; Found 210.9865.

NMR spectra are in agreement with the reported data.[24]

Methyl 1-methyl-1*H*-benzo[*d*]imidazole-5-carboxylate (**2s**)

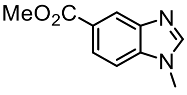

Methyl 1-methyl-1*H*-benzo[*d*]imidazole-5-carboxylate (**2s**) was prepared according to General Procedure B using methyl 1-methyl-1*H*-indole-5-carboxylate (**1s**, 47.3 mg) as starting material. To achieve full conversion, monitored by ^{1}H-NMR after solvent evaporation, an additional portion of the reagents and solvent was added (the General Procedure B was used again). The product was obtained as off-white solid in 21% yield (10 mg).

^{1}H NMR (400 MHz, *d_4*-MeOH) δ 8.34 (d, *J* = 1.5 Hz, 1H), 8.25 (s, 1H), 8.02 (dd, *J* = 8.6, 1.4 Hz, 1H), 7.62 (d, *J* = 8.6 Hz, 1H), 3.93 (s, 6H).

^{13}C NMR (126 MHz, *d_4*-MeOH) δ 169.0, 147.5, 143.6, 139.1, 125.8, 125.5, 122.3, 111.2, 52.6, 31.5.

HRMS (ESI) m/z: [M+H$^+$] Calculated for $C_{10}H_{11}N_2O_2$ 191.0815; Found 191.0817.

NMR spectra are in agreement with the reported data.[24]

5-Methoxy-1-methyl-1*H*-benzo[*d*]imidazole (**2t**)

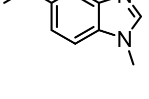

5-Methoxy-1-methyl-1*H*-benzo[*d*]imidazole (**2t**) was prepared according to General Procedure B using 5-methoxy-1-methyl-1*H*-indole (**1t**, 40.3 mg) as starting material. To achieve full conversion, monitored by ^{1}H-NMR after solvent evaporation, an additional portion of the reagents and solvent was added (the General Procedure B was used again). The product was obtained as orange oil in 25% yield (10.3 mg). *N*-(2-cyano-4-methoxyphenyl)-*N*-methylcyanamide (**3t**) was isolated in 60% yield (14.0 mg); the identity of the side product was unambiguously confirmed by XRD.

*Analytical data for 5-Methoxy-1-methyl-1H-benzo[d]imidazole (**2t**):*

^{1}H NMR (500 MHz, *d_4*-MeOH) δ 8.05 (s, 1H), 7.42 (d, *J* = 8.9 Hz, 1H), 7.15 (d, *J* = 2.4 Hz, 1H), 6.98 (dd, *J* = 8.8, 2.3 Hz, 1H), 3.86 (s, 3H), 3.84 (s, 3H).

^{13}C NMR (126 MHz, *d$_4$*-MeOH) δ 158.0, 145.2, 144.6, 130.4, 114.4, 111.5, 102.0, 56.2, 31.4.

HRMS (ESI) m/z: [M+H$^+$] Calculated for $C_9H_{11}N_2O$ 163.0866; Found 163.0864.

NMR spectra are in agreement with the reported data.[24]

*Analytical data for N-(2-cyano-4-methoxyphenyl)-N-methylcyanamide (**3t**):*

^{1}H NMR (500 MHz, *d$_4$*-MeOH) δ 7.51 (dd, *J* = 9.0, 0.4 Hz, 1H), 7.35 (dd, *J* = 2.9, 0.4 Hz, 1H), 7.31 (dd, *J* = 9.0, 3.0 Hz, 1H), 3.86 (s, 3H), 3.40 (s, 3H).

^{13}C NMR (126 MHz, *d$_4$*-MeOH) δ 159.9, 138.1, 126.5, 122.0, 119.6, 116.4, 116.0, 109.8, 56.6, 42.0.

HRMS (ESI) m/z: [M+H$^+$] Calculated for $C_{10}H_{10}N_3O$ 188.0818; Found 188.0819.

(1-Methyl-1*H*-benzo[*d*]imidazol-6-yl)methanol (**2u**)

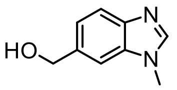

(1-Methyl-1*H*-benzo[*d*]imidazol-6-yl)methanol (**2u**) was prepared according to General Procedure B using (1-methyl-1*H*-indol-6-yl)methanol (40.3 mg) as starting material. The product was obtained as orange-brown solid in 39% yield (15.8 mg).

^{1}H NMR (400 MHz, *d$_4$*-MeOH) δ 8.07 (s, 1H), 7.62 (dd, *J* = 8.4, 0.7 Hz, 1H), 7.53 (dq, *J* = 1.5, 0.7 Hz, 1H), 7.28 (ddt, *J* = 8.3, 1.5, 0.5 Hz, 1H), 4.75 (s, 2H), 3.87 (s, 3H).

^{13}C NMR (101 MHz, *d$_4$*-MeOH) δ 145.4, 143.3, 138.3, 135.8, 123.0, 119.7, 109.5, 65.5, 31.3.

HRMS (ESI) m/z: [M+H$^+$] Calculated for $C_9H_{11}N_2O$ 163.0866; Found 163.0867.

N-(1-methyl-1*H*-benzo[*d*]imidazol-6-yl)acetamide (**2v**)

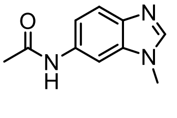

N-(1-methyl-1*H*-benzo[*d*]imidazol-6-yl)acetamide (**2v**) was prepared according to General Procedure B using *N*-(1-methyl-1*H*-indol-6-yl)acetamide (**1v**, 47.1 mg) as starting material. The product was obtained as brown solid in 37% yield (17.3 mg). The starting material was recovered in 60% yield (28.3 mg).

^{1}H NMR (600 MHz, *d$_4$*-MeOH) δ 8.07 (s, 1H), 8.06 (d, *J* = 2.0 Hz, 1H), 7.58 (dd, *J* = 8.7, 0.7 Hz, 1H), 7.20 (dd, *J* = 8.7, 2.0 Hz, 1H), 3.87 (s, 3H), 2.17 (s, 3H).

^{13}C NMR (151 MHz, *d$_4$*-MeOH) δ 171.6, 145.5, 140.6, 135.9, 135.8, 119.9, 117.0, 102.9, 31.3, 23.8.

HRMS (ESI) m/z: [M+H$^+$] Calculated for $C_{10}H_{12}N_3O$ 190.0975; Found 190.0977.

4-Ethoxy-1-(2,2,2-trifluoroethyl)-1*H*-benzo[*d*]imidazole-6-carboxylic acid (**2w**)

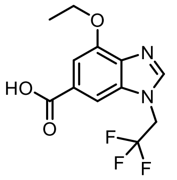

4-Ethoxy-1-(2,2,2-trifluoroethyl)-1*H*-benzo[*d*]imidazole-6-carboxylic acid (**2w**) was prepared according to General Procedure B using 4-ethoxy-1-(2,2,2-trifluoroethyl)-1*H*-indole-6-carboxylic acid (**1w**, 68.0 mg) as starting material. To achieve full conversion, monitored by ^{1}H-NMR after solvent evaporation, an additional portion of the reagents and solvent was added (the General Procedure B was used again). The product was obtained as yellow solid in 42% yield (28.8 mg). To improve solubility in d_4-MeOH, a drop of acetic acid was added.

^{1}H NMR (500 MHz, d_4-MeOH) δ 8.24 (s, 1H), 7.91 (s, 1H), 7.49 (s, 1H), 5.17 (q, *J* = 8.8 Hz, 2H), 4.29 (p, *J* = 7.8, 7.1 Hz, 2H), 2.00 (d, *J* = 15.9 Hz, 3H).

^{19}F NMR (470 MHz, d_4-MeOH) δ -72.99 (t, *J* = 8.9 Hz).

^{13}C NMR (126 MHz, d_4-MeOH) δ 163.5, 151.4, 146.1, 144.0, 136.6, 136.2, 125.2 (q, *J* = 278.9 Hz), 106.8, 106.3, 65.4, 46.6 (q, *J* = 35.6 Hz), 15.1.

HRMS (ESI) m/z: [M+H$^+$] Calculated for $C_{12}H_{12}F_3N_2O_3$ 289.0795; Found 289.0798.

1-methyl-1*H*-benzo[*d*]imidazole-3-^{15}N (**2x**)

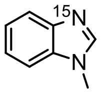

1-methyl-1*H*-benzo[*d*]imidazole-3-^{15}N (**2x**) was prepared using 1-methyl-1*H*-indole (**1a**, 32.8 mg) as starting material. The indole was added to a 150 mL pressure tube. ^{15}N-Ammonium chloride (232 mg, 17.0 equiv), K_2CO_3 (587 mg, 17.0 equiv) and methanol (30 mL) were added. The tube was sealed and stirred at rt at 600 rpm for 15 min. The stirring was stopped and PIDA (483 mg, 6 equiv) was added. The tube was sealed again, and the mixture was stirred for another 10 min. The solvent was evaporated, and the crude product was purified by column chromatography on silica gel. The product was obtained as orange-brown solid in 34% yield (11.1 mg). The ^{15}N incorporation is >95%.

^{1}H NMR (500 MHz, d_4-MeOH) δ 8.09 (d, *J* = 11.1 Hz, 1H), 7.66 (dq, *J* = 7.9, 0.8 Hz, 1H), 7.53 (dt, *J* = 8.2, 1.0 Hz, 1H), 7.33 (ddd, *J* = 8.1, 7.1, 1.2 Hz, 1H), 7.27 (ddt, *J* = 7.9, 7.1, 0.9 Hz, 1H), 3.88 (s, 3H).

^{13}C NMR (126 MHz, d_4-MeOH) δ 145.2 (d, *J* = 2.0 Hz), 143.9, 135.8, 124.2, 123.4 (d, *J* = 2.3 Hz), 119.93 (d, *J* = 5.5 Hz), 111.1, 31.3.

^{15}N NMR (51 MHz, d_4-MeOH) δ 230.00 (d, *J* = 11.0 Hz).

HRMS (ESI) m/z: [M+H$^+$] Calculated for $C_8H_9N^{15}N$ 134.0731; Found 134.0733.

N-(4-(tert-butyl)benzyl)-*N*,1-dimethyl-1*H*-benzo[*d*]imidazole-7-carboxamide (**2y**)

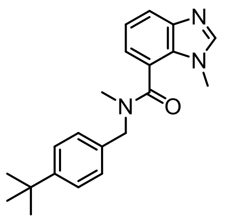

N-(4-(tert-butyl)benzyl)-*N*,1-dimethyl-1*H*-benzo[*d*]imidazole-7-carboxamide (**2y**) was prepared according to General Procedure B using *N*-(4-(tert-butyl)benzyl)-*N*,1-dimethyl-1*H*-indole-7-carboxamide (**1y**, 67.0 mg) as starting material. The product was obtained as a light-yellow solid in 33% yield (22.3 mg). Rotamers were obtained in a ratio of approximately 1.0:0.75 and were assigned if possible.

^{1}H NMR (400 MHz, *d_4*-MeOH) δ 8.14 (s, 1H, major), 8.12 (s, 0.75H, minor), 7.78 – 7.74 (m, 1.93H, not distinguishable), 7.47 – 7.43 (m, 2H, major), 7.41 – 7.39 (m, 2H, major), 7.37 – 7.32 (m, 3.13H, not distinguishable), 7.31 – 7.28 (m, 1.67H, minor), 7.25 (dd, *J* = 7.4, 1.0 Hz, 1H, not distinguishable), 6.93 – 6.90 (m, 1.66H, minor), 4.81 (s, 2H, major), 4.44 (s, 1.56H, minor), 3.72 (s, 3H, major), 3.66 (s, 2.36H, minor), 3.19 (s, 2.39H, minor), 2.87 (s, 3H, major), 1.34 (s, 9H, major), 1.28 (s, 6.77H, minor).

^{13}C NMR(126 MHz, *d_4*-MeOH) δ 171.3 (minor), 170.8 (major), 152.2 (minor), 152.1 (major), 147.1 (major+minor), 145.3 (major+minor), 134.8 (major), 134.4 (minor), 131.6 (minor), 131.4 (major), 129.5 (major), 128.4 (minor), 126.7 (major), 126.7 (minor), [123.3, 123.3] (not distinguishable), [122.4, 122.3] (not distinguishable), [121.9, 121.8] (not distinguishable), [121.6, 121.5] (not distinguishable), 56.1 (minor), 51.4 (major), 37.5 (major), 35.4 (major), 35.4 (minor), 34.1 (minor), 33.2 (major), 33.0 (minor), 31.8 (major), 31.7 (minor).

HRMS (ESI) m/z: [M+H$^+$] Calculated for $C_{21}H_{26}N_3O$ 336.207; Found 336.207.

1,4-Dimethyl-1*H*-benzo[*d*]imidazole (**2z**)

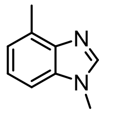

1,4-Dimethyl-1*H*-benzo[*d*]imidazole (**2z**) was prepared according to General Procedure B using 1,4-dimethyl-1*H*-indole (**1z**, 36.3 mg) as starting material. The product was obtained as brown oil in 56% yield (20.5 mg).

^{1}H NMR (500 MHz, *d_4*-MeOH) δ 8.06 (s, 1H), 7.32 (ddq, *J* = 8.3, 1.2, 0.6 Hz, 1H), 7.20 (dd, *J* = 8.1, 7.2 Hz, 1H), 7.06 (dp, *J* = 7.2, 0.9 Hz, 1H), 3.85 (s, 3H), 2.58 (s, 3H).

^{13}C NMR (126 MHz, *d_4*-MeOH) δ 144.47, 143.1, 135.5, 130.1, 124.2, 123.8, 108.5, 31.4, 16.7.

HRMS (ESI) m/z: [M+H$^+$] Calculated for $C_9H_{11}N_2$ 147.0917; Found 147.0917.

NMR spectra are in agreement with the reported data.[25]

4-Methoxy-1-methyl-1*H*-benzo[*d*]imidazole (**2aa**)

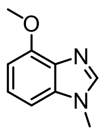

4-Methoxy-1-methyl-1*H*-benzo[*d*]imidazole (**2aa**) was prepared according to General Procedure B using 4-methoxy-1-methyl-1*H*-indole (**1aa**, 40.2 mg) as starting material. The product was obtained as brown in 63% yield (26 mg).

^{1}H NMR (400 MHz, *d_4*-MeOH) δ 7.99 (s, 1H), 7.30 – 7.16 (m, 1H), 7.11 (dd, J = 8.2, 0.8 Hz, 1H), 6.78 (dd, J = 7.9, 0.8 Hz, 1H), 3.97 (s, 3H), 3.86 (s, 3H).

^{13}C NMR (101 MHz, *d_4*-MeOH) δ 152.7, 143.8, 137.3, 134.0, 125.2, 104.0, 103.7, 56.0, 31.4.

HRMS (ESI) m/z: [M+H$^+$] Calculated for $C_9H_{11}N_2O$ 163.0866; Found 163.0866.

7-Methoxy-1-methyl-1*H*-benzo[*d*]imidazole (**2ab**)

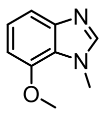

7-Methoxy-1-methyl-1*H*-benzo[*d*]imidazole (**2ab**) was prepared according to General Procedure B using 7-methoxy-1-methyl-1*H*-indole (**1ab**, 40.3 mg) as starting material. The product was obtained as brown solid in 44% yield (18 mg).

^{1}H NMR (500 MHz, *d_4*-MeOH) δ 7.94 (s, 1H), 7.21 (dd, *J* = 8.2, 0.9 Hz, 1H), 7.14 (t, *J* = 8.0 Hz, 1H), 6.78 (dd, *J* = 7.8, 0.8 Hz, 1H), 4.05 (s, 3H), 3.94 (s, 3H).

^{13}C NMR (126 MHz, *d_4*-MeOH) δ 149.3, 145.9, 145.2, 125.3, 124.2, 112.7, 105.0, 56.2, 34.4.

HRMS (ESI) m/z: [M+H$^+$] Calculated for $C_9H_{11}N_2O$ 163.0866; Found 163.0864.

NMR spectra are in agreement with the reported data.[26]

1,7-Dimethyl-1*H*-benzo[*d*]imidazole (**2ac**)

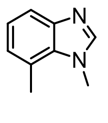

1,7-Dimethyl-1*H*-benzo[*d*]imidazole (**2ac**) was prepared according to General Procedure B using 1,7-dimethyl-1*H*-indole (**1ac**, 36.2 mg) as starting material. The product was obtained as brown solid in 49% yield (18 mg).

^{1}H NMR (400 MHz, *d_4*-MeOH) δ 7.96 (s, 1H), 7.48 – 7.41 (m, 1H), 7.10 (dd, J = 8.1, 7.3 Hz, 1H), 6.99 (dt, J = 7.2, 1.0 Hz, 1H), 4.07 (s, 3H), 2.73 (t, J = 0.7 Hz, 3H).

^{13}C NMR (101 MHz, *d_4*-MeOH) δ 146.0, 144.6, 134.2, 126.2, 123.6, 123.5, 118.0, 34.5, 18.4.

HRMS (ESI) m/z: [M+H$^+$] Calculated for $C_9H_{11}N_2$ 147.0917; Found 147.0918.

NMR spectra are in agreement with the reported data.[25]

6-Methoxy-1-methyl-1*H*-imidazo[4,5-*c*]pyridine (**2ad**)

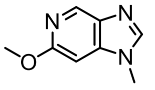

6-Methoxy-1-methyl-1*H*-imidazo[4,5-*c*]pyridine (**2ad**) was prepared according to General Procedure B using 6-methoxy-1-methyl-1*H*-pyrrolo[3,2-*c*]pyridine (**1ad**, 40.5 mg) as starting material. To achieve full conversion, monitored by ^{1}H-NMR after solvent evaporation, an additional portion of the reagents and solvent was added (the General Procedure B was used again). The product was obtained as brown oil in 27% yield (11.1 mg).

^{1}H NMR (500 MHz, *d4*-MeOH) δ 8.48 (d, *J* = 1.0 Hz, 1H), 8.11 (s, 1H), 6.91 (d, *J* = 1.0 Hz, 1H), 3.96 (s, 3H), 3.83 (s, 3H).

^{13}C NMR (126 MHz, *d4*-MeOH) δ 162.2, 147.8, 144.4, 138.7, 137.8, 89.6, 55.0, 31.2.

HRMS (ESI) m/z: [M+H$^+$] Calculated for $C_8H_{10}N_3O$ 164.0818; Found 164.0817.

5-Methoxy-3-methyl-3*H*-imidazo[4,5-*b*]pyridine (**2ae**)

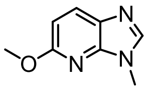

5-Methoxy-3-methyl-3*H*-imidazo[4,5-*b*]pyridine (**2ae**) was prepared according to General Procedure B using 6-methoxy-1-methyl-1*H*-pyrrolo[2,3-*b*]pyridine (**1ae**, 40.5 mg) as starting material. To achieve full conversion, monitored by ^{1}H-NMR after solvent evaporation, an additional portion of the reagents and solvent was added (the General Procedure B was used again). The product was obtained as brown solid in 41% yield (16.6 mg).

^{1}H NMR (500 MHz, *d4*-MeOH) δ 8.07 (s, 1H), 7.87 (d, *J* = 8.7 Hz, 1H), 6.70 (d, *J* = 8.7 Hz, 1H), 3.98 (s, 3H), 3.85 (d, *J* = 0.4 Hz, 3H).

^{13}C NMR (126 MHz, *d4*-MeOH) δ 163.3, 145.8, 143.6, 130.9, 130.1, 107.4, 54.1, 29.8.

HRMS (ESI) m/z: [M+H$^+$] Calculated for $C_8H_{10}N_3O$ 164.0818; Found 164.082.

4-Methoxy-1-methyl-1*H*-imidazo[4,5-*c*]pyridine (**2af**)

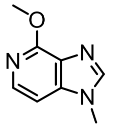

4-Methoxy-1-methyl-1*H*-imidazo[4,5-*c*]pyridine (**2af**) was prepared according to General Procedure B using 4-methoxy-1-methyl-1*H*-pyrrolo[3,2-*c*]pyridine (**1af**, 40.5 mg) as starting material. To achieve full conversion, monitored by ^{1}H-NMR after solvent evaporation, an additional portion of the reagents and solvent was added (the General Procedure B was used again). However, the starting material was isolated in 29% yield (11.6 mg) alongside the product which was obtained as brown solid in 32% yield (12.9 mg).

^{1}H NMR (500 MHz, *d4*-MeOH) δ 8.10 (s, 1H), 7.91 (d, *J* = 5.9 Hz, 1H), 7.20 (d, *J* = 5.9 Hz, 1H), 4.09 (s, 3H), 3.89 (d, *J* = 0.4 Hz, 3H).

^{13}C NMR (126 MHz, *d4*-MeOH) δ 157.4, 145.1, 142.5, 140.1, 128.8, 102.1, 54.0, 31.7.

HRMS (ESI) m/z: [M+H$^+$] Calculated for $C_8H_{10}N_3O$ 164.0818; Found 164.082.

7-Chloro-5-(1-methyl-1*H*-benzo[*d*]imidazol-6-yl)quinoxaline (**2ag**)

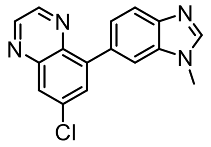

7-Chloro-5-(1-methyl-1*H*-benzo[*d*]imidazol-6-yl)quinoxaline (**2ag**) was prepared according to General Procedure B using 7-chloro-5-(1-methyl-1*H*-indol-6-yl)quinoxaline (**1ag**, 73.4 mg) as starting material. The product was obtained as light yellow solid in 41% yield (29.9 mg). To obtain a crystal structure, the sample was purified by preparative HPLC (1-50% ACN in H_2O with 0.1% TFA).

^{1}H NMR (400 MHz, *d$_4$*-MeOH) δ 8.86 (d, *J* = 1.8 Hz, 1H), 8.83 (d, *J* = 1.7 Hz, 1H), 8.16 (s, 1H), 8.03 (d, *J* = 2.4 Hz, 1H), 7.84 (d, *J* = 2.4 Hz, 1H), 7.76 (dd, *J* = 1.7, 0.7 Hz, 1H), 7.72 (dd, *J* = 8.4, 0.7 Hz, 1H), 7.52 (dd, *J* = 8.5, 1.6 Hz, 1H), 3.89 (s, 3H).

^{13}C NMR(101 MHz, *d$_4$*-MeOH) δ 147.1, 146.3, 146.3, 144.7, 144.4, 143.8, 140.7, 136.6, 135.7, 133.3, 132.5, 128.0, 126.6, 119.4, 113.6, 31.5.

HRMS (ESI) m/z: [M+H$^+$] Calculated for $C_{16}H_{12}ClN_4$ 295.0745; Found 295.0741.

2-(1-Methyl-1H-benzo[d]imidazol-6-yl)isoindolin-1-one (**2ah**)

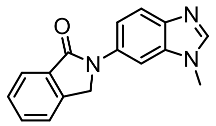

2-(1-Methyl-1*H*-benzo[*d*]imidazol-6-yl)isoindolin-1-one (**2ah**) was prepared according to General Procedure B using 2-(1-Methyl-1*H*-indol-6-yl)isoindolin-1-one (**1ah**, 66 mg) as starting material. The product was obtained as white solid in 45% yield (30 mg). To obtain a crystal structure, the sample was purified by preparative HPLC (1-50% ACN in H_2O with 0.1% TFA).

^{1}H NMR (500 MHz, *d$_4$*-MeOH) δ 8.12 – 8.07 (m, 2H), 7.82 (dt, J = 7.7, 1.0 Hz, 1H), 7.72 – 7.58 (m, 4H), 7.53 (dddt, J = 7.6, 7.0, 1.4, 0.7 Hz, 1H), 5.00 (s, 2H), 3.89 (s, 3H).

^{13}C NMR (126 MHz, *d$_4$*-MeOH) δ 169.6, 146.1, 142.6, 141.3, 136.3, 135.9, 133.8, 133.5, 129.4, 124.5, 124.1, 120.2, 117.3, 104.0, 53.3, 31.4.

HRMS (ESI) m/z: [M+H$^+$] Calculated for $C_{16}H_{14}N_3O$ 264.1131; Found 264.113.

1-(1-Methyl-1*H*-benzo[*d*]imidazol-4-yl)-3-(pyridin-2-yl)urea (**2ai**)

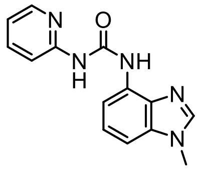

1-(1-Methyl-1*H*-benzo[*d*]imidazol-4-yl)-3-(pyridin-2-yl)urea (**2ai**) was prepared according to General Procedure B using 1-(1-methyl-1*H*-indol-4-yl)-3-(pyridin-2-yl)urea (**1ai**, 66.6 mg) as starting material. The product was obtained as off-white

solid in 39% yield (26 mg). To obtain a crystal structure, the sample was purified by preparative HPLC (1-50% ACN in H_2O with 0.1% TFA).

^{1}H NMR (600 MHz, *d$_4$*-MeOH) δ 8.46 – 8.43 (m, 1H), 8.08 (s, 1H), 8.00 (dd, *J* = 7.6, 1.1 Hz, 1H), 7.73 (ddd, *J* = 8.7, 7.3, 1.9 Hz, 1H), 7.29 (t, *J* = 7.9 Hz, 1H), 7.25 (dd, *J* = 8.2, 1.0 Hz, 1H), 7.14 (d, *J* = 8.3 Hz, 1H), 7.04 – 7.01 (m, 1H), 3.91 (s, 3H).

^{13}C NMR (151 MHz, *d$_4$*-MeOH) δ 155.2, 154.5, 147.9, 144.5, 139.8, 136.5, 135.7, 131.2, 124.7, 118.6, 113.4, 113.1, 105.9, 31.4.

HRMS (ESI) m/z: [M+H$^+$] Calculated for $C_{14}H_{14}N_5O$ 268.1193; Found 268.1189.

Methyl 4-(benzyloxy)-1-methyl-1*H*-benzo[*d*]imidazole-6-carboxylate (**2aj**)

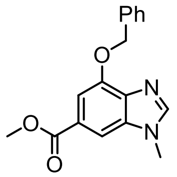

Methyl 4-(benzyloxy)-1-methyl-1*H*-benzo[*d*]imidazole-6-carboxylate (**2aj**) was prepared according to General Procedure B using methyl 4-(benzyloxy)-1-methyl-1*H*-indole-6-carboxylate (**1aj**, 69.4 mg) as starting material. The product was obtained as brown solid in 49% yield (33.9 mg).

^{1}H NMR (400 MHz, *d$_4$*-MeOH) δ 8.13 (s, 1H), 7.80 (d, *J* = 1.3 Hz, 1H), 7.57 – 7.48 (m, 2H), 7.44 (d, *J* = 1.3 Hz, 1H), 7.40 – 7.26 (m, 3H), 4.87 (s, 2H), 3.91 (s, 3H), 3.87 (s, 3H).

^{13}C NMR (101 MHz, *d$_4$*-MeOH) δ 168.7, 151.1, 146.6, 138.2, 137.8, 136.9, 129.5, 129.0, 128.8, 126.9, 106.8, 106.4, 71.5, 52.7, 31.6.

HRMS (ESI) m/z: [M+H$^+$] Calculated for $C_{17}H_{17}N_2O_3$ 297.1234; Found 297.1228.

Methyl 4-(benzyloxy)-1-ethyl-1*H*-benzo[*d*]imidazole-6-carboxylate (**2ak**)

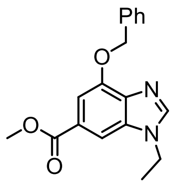

Methyl 4-(benzyloxy)-1-ethyl-1*H*-benzo[*d*]imidazole-6-carboxylate (**2ak**) was prepared according to General Procedure B using methyl 4-(benzyloxy)-1-ethyl-1*H*-indole-6-carboxylate (**1ak**, 63.0 mg) as starting material. The product was obtained as brown solid in 40% yield (25.5mg). To obtain a crystal structure, the sample was purified by preparative HPLC (1-50% ACN in H_2O with 0.1% TFA).

^{1}H NMR (600 MHz, *d$_4$*-MeOH) δ 8.23 (s, 1H), 7.87 (s, 1H), 7.54 (ddt, *J* = 7.6, 1.3, 0.7 Hz, 2H), 7.47 (s, 1H), 7.40 – 7.34 (m, 2H), 7.34 – 7.28 (m, 1H), 5.33 (s, 2H), 4.33 (q, *J* = 7.3 Hz, 2H), 3.92 (s, 3H), 1.51 (t, *J* = 7.3 Hz, 3H).

^{13}C NMR (151 MHz, *d$_4$*-MeOH) δ 168.7, 151.3, 145.6, 138.2, 138.1, 135.9, 129.5, 129.0, 128.8, 126.9, 107.0, 106.4, 71.6, 52.8, 41.3, 15.6.

HRMS (ESI) m/z: [M+H$^+$] Calculated for $C_{18}H_{19}N_2O_3$ 311.139; Found 311.1387.

4-(Methyl(1-methyl-1*H*-benzo[*d*]imidazol-7-yl)carbamoyl)phenyl diethylcarbamate (**2al**)

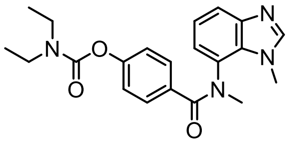

4-(Methyl(1-methyl-1*H*-benzo[*d*]imidazol-7-yl)carbamoyl)phenyl diethylcarbamate (**2al**) was prepared according to General Procedure B using 4-(methyl(1-methyl-1*H*-indol-7-yl)carbamoyl)phenyl diethylcarbamate (**1al**, 94.9 mg) as starting material. The product was obtained as a light yellow solid in 15% yield (14.6 mg).

^{1}H NMR (500 MHz, *d$_4$*-MeOH) δ 8.09 (s, 1H), 7.59 (dd, *J* = 7.9, 1.2 Hz, 1H), 7.35 – 7.30 (m, 2H), 7.23 – 7.14 (m, 2H), 6.90 – 6.85 (m, 2H), 4.01 (s, 3H), 3.53 (s, 3H), 3.38 (p, *J* = 6.3 Hz, 2H), 1.15 (dt, *J* = 25.2, 7.1 Hz, 6H). -N-C*H$_2$*-Me (δ 3.30) signal is not visible due to overlap with solvent peak.

^{13}C NMR (126 MHz, *d$_4$*-MeOH) δ 172.4, 155.3, 154.1, 147.2, 146.4, 133.4, 131.2, 130.8, 129.9, 125.4, 124.0, 122.2, 120.6, 43.3 (d, *J* = 32.0 Hz), 40.5, 33.4, 13.9 (d, *J* = 111.8 Hz).

HRMS (ESI) m/z: [M+H$^+$] Calculated for $C_{21}H_{25}N_4O_3$ 381.1921; Found 381.1912.

3-(1*H*-benzo[*d*]imidazol-1-yl)propanamide (**2am**)

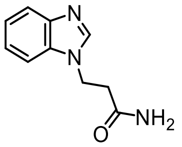

3-(1*H*-benzo[*d*]imidazol-1-yl)propanamide (**2am**) was prepared according to General Procedure B using 3-(1*H*-indol-1-yl)propanamide (**1am**, 55.0 mg) as starting material. The product was obtained as a light orange oil in 28% yield (15.5 mg).

^{1}H NMR (500 MHz, *d$_4$*-MeOH) δ 8.13 (s, 1H), 7.63 (ddt, *J* = 23.4, 8.1, 1.0 Hz, 2H), 7.30 (dddd, *J* = 27.3, 8.3, 7.2, 1.2 Hz, 2H), 4.58 (t, *J* = 6.6 Hz, 2H), 2.79 (t, *J* = 6.6 Hz, 2H).

^{13}C NMR (126 MHz, *d$_4$*-MeOH) δ 175.2, 144.9, 144.0, 134.7, 124.3, 123.6, 120.1, 111.4, 42.0, 36.2.

HRMS (ESI) m/z: [M+H$^+$] Calculated for $C_{10}H_{12}N_3O$ 190.0975; Found 190.0975.

NMR spectra are in agreement with the reported data.[27]

2-(6-(2-(4-Methyl-2-(4-(trifluoromethyl)phenyl)thiazol-5-yl)ethoxy)-1*H*-benzo[*d*]imidazol-1-yl)acetic acid (**2an**)

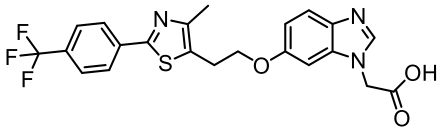

2-(6-(2-(4-Methyl-2-(4-(trifluoromethyl)phenyl)thiazol-5-yl)ethoxy)-1*H*-benzo[*d*]imidazol-1-yl)acetic acid (**2an**) was prepared according to General Procedure B using 2-(6-(2-(4-methyl-2-(4-(trifluoromethyl)phenyl)thiazol-5-yl)ethoxy)-1*H*-indol-1-yl)acetic acid (**1an**, 65.0 mg) as

starting material. The product was obtained as orange-brown solid in 41% yield (27.0 mg). To improve solubility in d_4-MeOH, a drop of acetic acid was added.

^{1}H NMR (400 MHz, d_4-MeOH) δ 8.00 (d, J = 8.2 Hz, 3H), 7.70 (d, J = 8.2 Hz, 2H), 7.50 (d, J = 8.8 Hz, 1H), 6.98 (d, J = 2.4 Hz, 1H), 6.89 (dd, J = 8.8, 2.2 Hz, 1H), 4.73 (s, 2H), 4.20 (t, J = 6.0 Hz, 2H), 3.27 (t, J = 5.9 Hz, 2H), 2.44 (s, 3H).

^{13}C NMR (101 MHz, d_4-MeOH) δ 174.9, 164.4, 157.1, 151.8, 145.0, 138.3, 138.2, 136.3, 132.2 (d, J = 32.5 Hz), 131.9, 127.6, 127.0 (d, J = 3.9 Hz), 125.5 (d, J = 271.4 Hz), 120.4, 113.4, 95.7, 69.4, 27.7, 15.1. One signal is obstructed by the solvent signal.

^{19}F NMR (470 MHz, d_4-MeOH) δ -64.25.

HRMS (ESI) m/z: [M+H$^+$] Calculated for $C_{22}H_{19}F_3N_3O_3S$ 462.1094; Found 462.1092.

N-(2-Chloro-5-(1-methyl-1*H*-benzo[*d*]imidazol-6-yl)pyridin-3-yl)benzenesulfonamide (**2ao**)

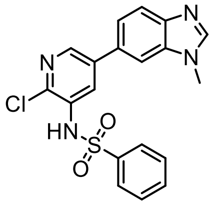

N-(2-Chloro-5-(1-methyl-1*H*-benzo[*d*]imidazol-6-yl)pyridin-3-yl)benzenesulfonamide (**2ao**) was prepared according to General Procedure B using *N*-(2-chloro-5-(1-methyl-1*H*-indol-6-yl)pyridin-3-yl)benzenesulfonamide (**1ao**, 80 mg) as starting material. The product was obtained as brown solid in 23% yield (18 mg).

^{1}H NMR (500 MHz, d_4-MeOH) δ 8.51 (d, J = 2.4 Hz, 1H), 8.36 (s, 1H), 8.22 (d, J = 2.3 Hz, 1H), 7.87 (dd, J = 1.7, 0.7 Hz, 1H), 7.83 – 7.78 (m, 3H), 7.69 – 7.62 (m, 1H), 7.60 – 7.49 (m, 3H), 4.01 (d, J = 0.5 Hz, 3H).

^{13}C NMR (126 MHz, d_4-MeOH) δ 146.4, 145.6, 144.9, 143.0, 141.4, 138.4, 136.3, 134.5, 134.5, 132.7, 132.7, 130.4, 128.3, 123.4, 120.4, 110.4, 31.8.

HRMS (ESI) m/z: [M+H$^+$] Calculated for $C_{19}H_{16}ClN_4O_2S$ 399.0677; Found 399.0677.

2-Ethoxy-3-(1-((2-(4-isopropylphenyl)-5-methyloxazol-4-yl)methyl)-4-methyl-1*H*-benzo[*d*]imidazol-5-yl)propanoic acid (**2ap**)

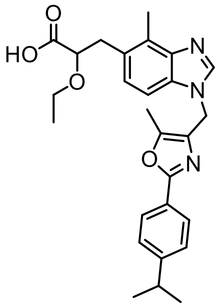

2-Ethoxy-3-(1-((2-(4-isopropylphenyl)-5-methyloxazol-4-yl)methyl)-4-methyl-1*H*-benzo[*d*]imidazol-5-yl)propanoic acid (**2ap**) was prepared according to General Procedure B using 2-ethoxy-3-(1-((2-(4-isopropylphenyl)-5-methyloxazol-4-yl)methyl)-4-methyl-1*H*-indol-5-yl)propanoic acid (**1ap**, 64.0 mg) as starting material. The product was obtained as orange solid in 63% yield (40.3 mg).

^{1}H NMR (500 MHz, Methylene Chloride-*d*$_2$) δ 8.08 (s, 1H), 7.89 – 7.83 (m, 2H), 7.32 – 7.25 (m, 3H), 7.20 (d, *J* = 8.3 Hz, 1H), 5.18 (s, 2H), 4.07 (dd, *J* = 7.4, 5.3 Hz, 1H), 3.60 – 3.51 (m, 1H), 3.35 (dq, *J* = 9.3, 7.0 Hz, 1H), 3.28 (dd, *J* = 14.2, 5.3 Hz, 1H), 3.11 (dd, *J* = 14.2, 7.4 Hz, 1H), 2.93 (p, *J* = 6.9 Hz, 1H), 2.56 (d, *J* = 2.7 Hz, 3H), 2.35 (s, 3H), 1.25 (d, *J* = 6.9 Hz, 6H), 1.11 (t, *J* = 7.0 Hz, 3H).

^{13}C NMR (126 MHz, Methylene Chloride-*d*$_2$) δ 174.1, 160.9, 152.1, 146.4, 143.3, 143.1, 132.7, 131.2, 129.7, 129.1, 127.4, 126.6, 126.5, 125.4, 107.7, 80.7, 67.2, 41.4, 36.6, 34.7, 24.1, 15.5, 13.5, 10.7.

HRMS (ESI) m/z: [M+H$^+$] Calculated for $C_{27}H_{32}N_3O_4$ 462.2387; Found 462.2376.

3,3-Dimethyl-1-(1-methyl-1*H*-benzo[*d*]imidazol-6-yl)-6-(2-methylpyrimidin-5-yl)indolin-2-one (**2aq**)

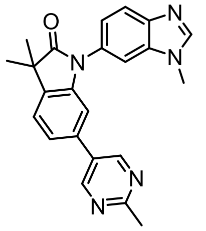

3,3-Dimethyl-1-(1-methyl-1*H*-benzo[*d*]imidazol-6-yl)-6-(2-methylpyrimidin-5-yl)indolin-2-one (**2aq**) was prepared according to General Procedure B using 3,3-dimethyl-1-(1-methyl-1*H*-indol-6-yl)-6-(2-methylpyrimidin-5-yl)indolin-2-one (**1aq**, 65.1 mg) as starting material. The product was obtained as orange oil in 43% yield (28 mg). The starting material was recovered in 37% yield (24.3 mg).

^{1}H NMR (500 MHz, *d*$_4$-MeOH) δ 8.80 (s, 2H), 8.23 (s, 1H), 7.85 – 7.81 (m, 1H), 7.72 (dd, *J* = 2.0, 0.5 Hz, 1H), 7.54 (d, *J* = 7.7 Hz, 1H), 7.42 (dd, *J* = 7.7, 1.6 Hz, 1H), 7.33 (dd, *J* = 8.5, 1.9 Hz, 1H), 7.03 (d, *J* = 1.5 Hz, 1H), 3.91 (s, 3H), 2.65 (s, 3H), 1.53 (s, 6H).

^{13}C NMR (126 MHz, *d*$_4$-MeOH) δ 183.3, 167.6, 156.2, 147.1, 145.4, 143.8, 137.5, 136.4, 135.4, 132.7, 130.8, 124.8, 123.3, 122.5, 121.2, 110.9, 108.9, 45.6, 31.6, 25.1 (d, *J* = 4.4 Hz), 24.8.

HRMS (ESI) m/z: [M+H$^+$] Calculated for $C_{23}H_{22}N_5O$ 384.1819; Found 384.1818.

4-(6-Chloro-2-(1-methyl-1*H*-benzo[*d*]imidazol-4-yl)pyrimidin-4-yl)morpholine (**2ar**)

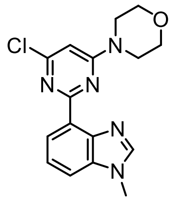

4-(6-Chloro-2-(1-methyl-1*H*-benzo[*d*]imidazol-4-yl)pyrimidin-4-yl)morpholine (**2ar**) was prepared according to General Procedure B using 4-(6-chloro-2-(1-methyl-1*H*-indol-4-yl)pyrimidin-4-yl)morpholine (**1ar**, 82.2 mg) as starting material. The product was obtained as a yellow solid in 93% yield (76.6 mg).

^{1}H NMR (600 MHz, *d$_6$*-acetone) δ 8.11 (s, 1H), 8.02 (dd, *J* = 7.5, 1.1 Hz, 1H), 7.66 (dd, *J* = 8.1, 1.2 Hz, 1H), 7.39 – 7.35 (m, 1H), 6.70 (s, 1H), 3.96 (s, 3H), 3.85 (s, 4H), 3.77 – 3.75 (m, 4H).

^{13}C NMR (151 MHz, *d$_6$*-acetone) δ 165.27, 164.12, 160.97, 145.77, 143.45, 137.06, 130.50, 124.05, 122.56, 112.79, 99.54, 67.07, 45.43, 31.22.

HRMS (ESI) m/z: [M+H$^+$] Calculated for $C_{16}H_{17}ClN_5O$ 330.1116; Found 330.1115.

Methyl 1-ethyl-4-((2-(trimethylsilyl)ethoxy)methoxy)-1*H*-benzo[*d*]imidazole-6-carboxylate (**2as**)

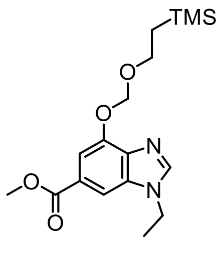

Methyl 1-ethyl-4-((2-(trimethylsilyl)ethoxy)methoxy)-1*H*-benzo[*d*]imidazole-6-carboxylate (**2as**) was prepared according to General Procedure B using methyl 1-ethyl-4-((2-(trimethylsilyl)ethoxy)methoxy)-1*H*-indole-6-carboxylate (**1as**, 66.9 mg) as starting material. To achieve full conversion, monitored by ^{1}H-NMR after solvent evaporation, an additional portion of the reagents and solvent was added (the General Procedure B was used again). The product was obtained as orange oil in 48% yield (32.4 mg).

^{1}H NMR (400 MHz, *d$_4$*-MeOH) δ 8.25 (s, 1H), 7.92 (d, *J* = 1.3 Hz, 1H), 7.63 (d, *J* = 1.3 Hz, 1H), 5.47 (s, 2H), 4.35 (q, *J* = 7.3 Hz, 2H), 3.93 (s, 3H), 3.88 – 3.83 (m, 2H), 1.52 (t, *J* = 7.3 Hz, 3H), 0.97 – 0.90 (m, 2H), -0.04 (s, 9H).

^{13}C NMR (101 MHz, *d$_4$*-MeOH) δ 168.7, 149.7, 145.8, 138.5, 136.0, 126.9, 108.9, 107.5, 94.4, 67.6, 52.7, 41.3 (d, *J* = 2.1 Hz), 18.8, 15.6 (d, *J* = 1.1 Hz), -1.4.

HRMS (ESI) m/z: [M+H$^+$] Calculated for $C_{17}H_{27}N_2O_4Si$ 351.1735; Found 351.1727.

Ethyl 2-ethoxy-3-(1-(3-(5-methyl-2-phenyloxazol-4-yl)propyl)-1*H*-benzo[*d*]imidazol-4-yl)propanoate (**2at**)

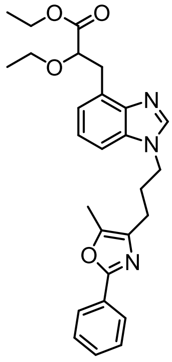

Ethyl 2-ethoxy-3-(1-(3-(5-methyl-2-phenyloxazol-4-yl)propyl)-1*H*-benzo[*d*]imidazol-4-yl)propanoate (**2at**) was prepared according to General Procedure B using ethyl 2-ethoxy-3-(1-(3-(5-methyl-2-phenyloxazol-4-yl)propyl)-1*H*-indol-4-yl)propanoate (**1at**, 70.0 mg) as starting material. The product was obtained as brown solid in 34% yield (23.9 mg).

^{1}H NMR (500 MHz, *d₄*-MeOH) δ 8.19 (s, 1H), 7.93 – 7.89 (m, 2H), 7.48 – 7.42 (m, 4H), 7.24 – 7.20 (m, 1H), 7.09 (dd, *J* = 7.3, 1.0 Hz, 1H), 4.38 – 4.32 (m, 3H), 4.05 (qd, *J* = 7.1, 2.1 Hz, 2H), 3.55 (dq, *J* = 9.2, 7.0 Hz, 1H), 3.41 – 3.29 (m, 3H), 2.54 (t, *J* = 7.2 Hz, 2H), 2.31 – 2.27 (m, 2H), 2.24 (s, 3H), 1.09 (td, *J* = 7.1, 5.8 Hz, 6H).

^{13}C NMR (126 MHz, d_4-MeOH) δ 174.3, 161.1, 145.7, 144.4, 143.1, 135.6, 134.9, 131.4, 130.0, 129.2, 128.5, 126.9, 124.4, 124.1, 110.2, 80.1, 67.0, 61.8, 45.4, 35.6, 29.5, 23.3, 15.4, 14.4, 9.9.

HRMS (ESI) m/z: [M+H$^+$] Calculated for $C_{27}H_{32}N_3O_4$ 462.2387; Found 462.2381.

2-Ethoxy-3-(1-((5-methyl-2-(4-(trifluoromethyl)phenyl)oxazol-4-yl)methyl)-1*H*-benzo[*d*]imidazol-4-yl)propanoic acid (**2au**)

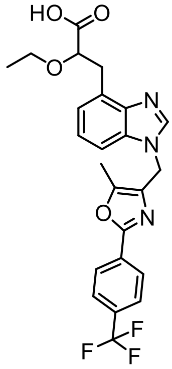

2-Ethoxy-3-(1-((5-methyl-2-(4-(trifluoromethyl)phenyl)oxazol-4-yl)methyl)-1*H*-benzo[*d*]imidazol-4-yl)propanoic acid (**2au**) was prepared according to General Procedure B using 2-ethoxy-3-(1-((5-methyl-2-(4-(trifluoromethyl)phenyl)oxazol-4-yl)methyl)-1*H*-indol-4-yl)propanoic acid (**1au**, 69.0 mg) as starting material. The product was obtained as off-white solid in 65% yield (45.2 mg). The starting material was recovered in 21% yield (14.6 mg).

^{1}H NMR (600 MHz, d_4-MeOH) δ 8.32 (s, 1H), 8.05 (ddd, *J* = 8.0, 1.6, 0.8 Hz, 2H), 7.73 – 7.70 (m, 2H), 7.47 (dd, *J* = 7.9, 1.3 Hz, 1H), 7.26 – 7.20 (m, 2H), 5.38 (s, 2H), 4.12 (dd, *J* = 8.8, 4.7 Hz, 1H), 3.57 – 3.42 (m, 2H), 3.30 – 3.21 (m, 2H), 2.46 (s, 3H), 0.95 (t, *J* = 6.9 Hz, 3H).

^{19}F NMR (470 MHz, d_4-MeOH) δ -64.45.

^{13}C NMR (151 MHz, d_4-MeOH) δ 180.7, 160.1, 149.0, 144.4, 143.1, 134.7, 133.0, 132.9 (q, *J* = 32.5 Hz), 131.6, 131.0, 127.5, 126.9 (q, *J* = 3.6 Hz), 125.3 (q, *J* = 271.4 Hz), 124.6, 124.4, 109.8, 82.7, 66.8, 41.3, 35.8, 15.2, 10.1.

HRMS (ESI) m/z: [M+H$^+$] Calculated for $C_{24}H_{23}F_3N_3O_4$ 474.1635; Found 474.1629.

Unsuccessful Substrates

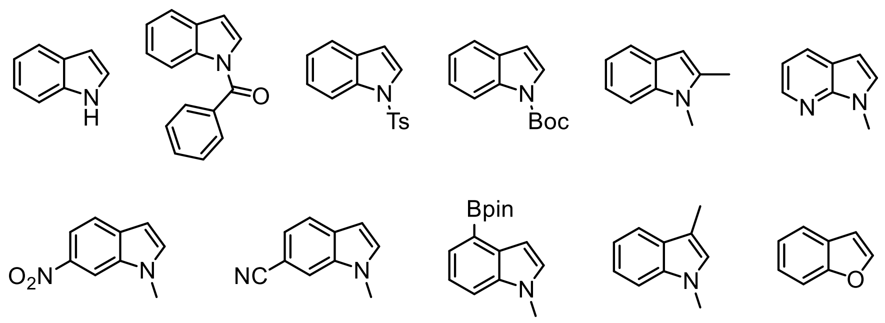

6 CRYSTALLOGRAPHIC DATA

7-Chloro-5-(1-methyl-1*H*-benzo[*d*]imidazol-6-yl)quinoxaline (**2ag**)

CCDC 2388835, melting point: 208.2 – 210.0 °C

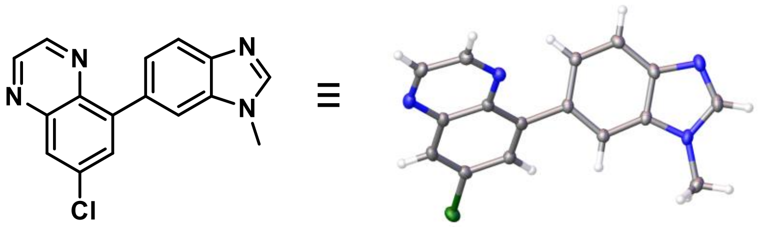

Identification code	bm010424_2_1
Empirical formula	$C_{16}H_{11}ClN_4$
Formula weight	294.74
Temperature [K]	100.0(1)
Crystal system	triclinic
Space group (number)	$P\bar{1}$ (2)
a [Å]	7.34320(10)
b [Å]	9.00530(10)
c [Å]	11.17280(10)
α [°]	95.5820(10)
β [°]	101.1100(10)
γ [°]	112.0100(10)
Volume [Å^3]	660.379(14)
Z	2
ρ_{calc} [gcm^{-3}]	1.482
μ [mm^{-1}]	2.537

F(000)	304
Crystal size [mm^3]	0.196×0.104×0.064
Radiation	Cu K_α (λ=1.54184 Å)
2θ range [°]	8.21 to 147.16 (0.80 Å)
Index ranges	−9 ≤h ≤ 8<br>−11 ≤ k ≤ 11<br>−13 ≤ l ≤ 13
Reflections collected	24141
Independent reflections	2545<br>R_{int} = 0.0213<br>R_{sigma} = 0.0094
Data / Restraints / Parameters	2545/0/191
Absorption correction T_{min}/T_{max} (method)	0.685/1.000 (gaussian)
Goodness-of-fit on F^2	1.073
Final *R* indexes [I≥2σ(I)]	R_1 = 0.0279<br>wR_2 = 0.0754
Final *R* indexes [all data]	R_1 = 0.0286<br>wR_2 = 0.0759
Largest peak/hole [eÅ^{-3}]	0.25/−0.25

2-(1-Methyl-1H-benzo[d]imidazol-6-yl)isoindolin-1-one (**2ah**)
CCDC 2379390, melting point: 226.7 – 228.8 °C

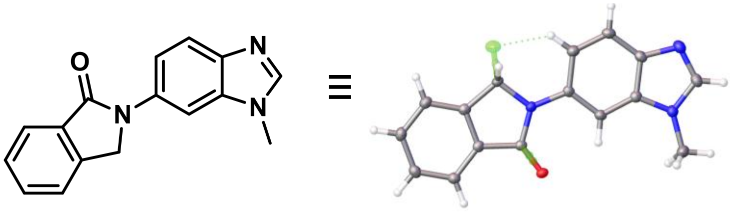

Identification code	bm280424_1_1
Empirical formula	$C_{16}H_{13}N_3O$
Formula weight	263.29
Temperature [K]	100.0(1)
Crystal system	monoclinic
Space group (number)	$P2_1/c$ (14)
a [Å]	13.71640(10)
b [Å]	7.38380(10)
c [Å]	12.94220(10)
α [°]	90
β [°]	110.8330(10)
γ [°]	90
Volume [Å³]	1225.08(2)
Z	4
ρ_{calc} [gcm⁻³]	1.428
μ [mm⁻¹]	0.741

F(000)	552
Crystal size [mm³]	0.195×0.149×0.122
Radiation	Cu K_α (λ=1.54184 Å)
2θ range [°]	6.90 to 148.97 (0.80 Å)
Index ranges	−16 ≤ h ≤ 16<br>−9 ≤ k ≤ 9<br>−16 ≤ l ≤ 16
Reflections collected	37540
Independent reflections	2439<br>R_{int} = 0.0160<br>R_{sigma} = 0.0050
Data / Restraints / Parameters	2439/50/192
Absorption correction T_{min}/T_{max} (method)	0.922/1.000 (gaussian)
Goodness-of-fit on F^2	1.081
Final *R* indexes [I≥2σ(I)]	R_1 = 0.0344<br>wR_2 = 0.0878
Final *R* indexes [all data]	R_1 = 0.0345<br>wR_2 = 0.0879
Largest peak/hole [eÅ⁻³]	0.23/−0.22

1-(1-Methyl-1*H*-benzo[*d*]imidazol-4-yl)-3-(pyridin-2-yl)urea (**2ai**)

CCDC 2388836, melting point: 225.7 – 227.3 °C

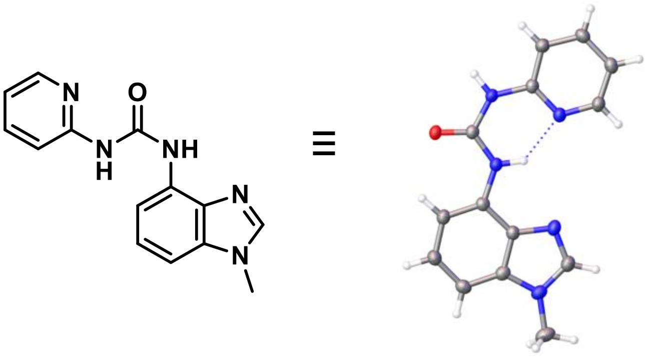

Identification code	bm030424_1_1
Empirical formula	$C_{14}H_{13}N_5O$
Formula weight	267.29
Temperature [K]	100.0(1)
Crystal system	monoclinic
Space group (number)	$P2_1/n$ (14)
a [Å]	12.5857(4)
b [Å]	5.25310(10)
c [Å]	19.0057(6)
α [°]	90
β [°]	100.359(3)
γ [°]	90
Volume [$Å^3$]	1236.06(6)
Z	4
ρ_{calc} [gcm^{-3}]	1.436
μ [mm^{-1}]	0.788

F(000)	560
Crystal size [mm^3]	0.138×0.038×0.015
Radiation	Cu K_α (λ=1.54184 Å)
2θ range [°]	7.82 to 147.67 (0.80 Å)
Index ranges	−14 ≤ h ≤ 15<br>−6 ≤ k ≤ 6<br>−23 ≤ l ≤ 22
Reflections collected	16462
Independent reflections	2421<br>R_{int} = 0.0317<br>R_{sigma} = 0.0228
Data / Restraints / Parameters	2421/2/188
Absorption correction T_{min}/T_{max} (method)	0.912/1.000 (gaussian)
Goodness-of-fit on F^2	1.077
Final *R* indexes [I≥2σ(I)]	R_1 = 0.0401<br>wR_2 = 0.1021
Final *R* indexes [all data]	R_1 = 0.0472<br>wR_2 = 0.1062
Largest peak/hole [$eÅ^{-3}$]	0.20/−0.27

Methyl 4-(benzyloxy)-1-ethyl-1H-benzo[d]imidazole-6-carboxylate (**2ak**)

CCDC 2388839, melting point: 137.2 – 139.9 °C

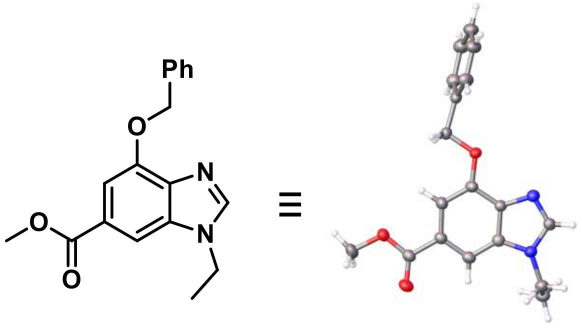

Identification code	bm280924_1_1
Empirical formula	$C_{18}H_{18}N_2O_3$
Formula weight	310.34
Temperature [K]	100.0(1)
Crystal system	monoclinic
Space group (number)	$P2_1/c$ (14)
a [Å]	11.6236(4)
b [Å]	5.33982(16)
c [Å]	25.6654(8)
α [°]	90
β [°]	95.176(3)
γ [°]	90
Volume [Å^3]	1586.50(9)
Z	4
ρ_{calc} [gcm^{-3}]	1.299
μ [mm^{-1}]	0.728

F(000)	656
Crystal size [mm^3]	0.008×0.018×0.415
Radiation	Cu K_α (λ=1.54184 Å)
2θ range [°]	6.92 to 149.82 (0.80 Å)
Index ranges	−14 ≤ h ≤ 12<br>−4 ≤ k ≤ 6<br>−31 ≤ l ≤ 31
Reflections collected	16015
Independent reflections	3129<br>R_{int} = 0.0529<br>R_{sigma} = 0.0408
Data / Restraints / Parameters	3129 / 0 / 210
Absorption correction T_{min}/T_{max} (method)	0.6690 / 1.0000 (gaussian)
Goodness-of-fit on F^2	1.076
Final *R* indexes [I≥2σ(I)]	R_1 = 0.0599<br>wR_2 = 0.1552
Final *R* indexes [all data]	R_1 = 0.0787<br>wR_2 = 0.1689
Largest peak/hole [eÅ^{-3}]	0.23/−0.38

3,3-Dimethyl-1-(1-methyl-1*H*-benzo[*d*]imidazol-6-yl)-6-(2-methylpyrimidin-5-yl)indolin-2-one (**2aq**)

CCDC 2388838, melting point: 202.9 – 204.7 °C

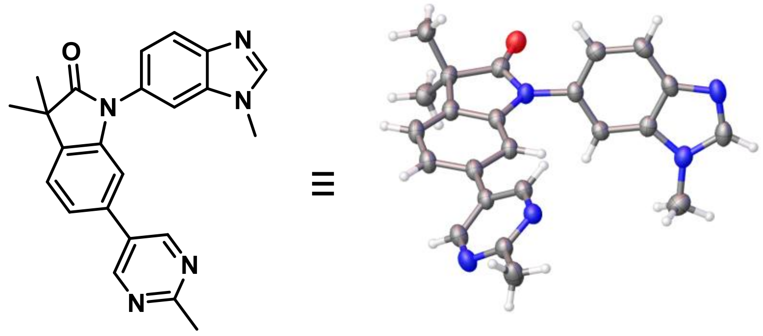

Identification code	bm230924_2_1
Empirical formula	$C_{23}H_{21}N_5O$
Formula weight	383.45
Temperature [K]	100.0(1)
Crystal system	triclinic
Space group (number)	$P\bar{1}$ (2)
a [Å]	7.1591(2)
b [Å]	9.0936(3)
c [Å]	16.6798(5)
α [°]	99.532(3)
β [°]	93.941(2)
γ [°]	110.972(3)
Volume [Å^3]	990.25(6)
Z	2
ρ_{calc} [gcm^{-3}]	1.286
μ [mm^{-1}]	0.656

F(000)	404
Crystal size [mm^3]	0.028×0.089×0.097
Radiation	Cu K_α (λ=1.54184 Å)
2θ range [°]	5.43 to 149.44 (0.80 Å)
Index ranges	−8 ≤ h ≤ 8<br>−11 ≤ k ≤ 9<br>−20 ≤ l ≤ 20
Reflections collected	21066
Independent reflections	3869<br>R_{int} = 0.0296<br>R_{sigma} = 0.0278
Data / Restraints / Parameters	99.2 %
Absorption correction T_{min}/T_{max} (method)	3869 / 0 / 266
Goodness-of-fit on F^2	0.8860 / 1.0000 (gaussian)
Final *R* indexes [I≥2σ(I)]	1.064
Final *R* indexes [all data]	R_1 = 0.0460<br>wR_2 = 0.1236
Largest peak/hole [eÅ^{-3}]	R_1 = 0.0588<br>wR_2 = 0.1313

4-Amino-1-benzylquinazolin-1-ium TFA salt (**SI-8**)

CCDC 2388840, melting point: 110.3 – 113.4 °C

4-Amino-1-benzylquinazolin-1-ium TFA salt (**SI-8**) was isolated as a side product from the reaction with 1-benzyl-1*H*-indole. A sample was purified by preparative HPLC (1-70% ACN in H_2O with 0.1% TFA) to obtain a crystal structure.

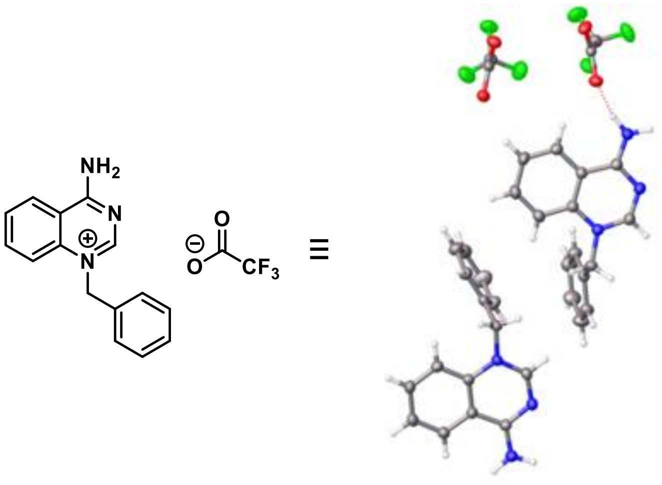

Identification code	bm270223_1_1
Empirical formula	$C_{17}H_{14}F_3N_3O_2$
Formula weight	349.31
Temperature [K]	100.0(1)
Crystal system	triclinic
Space group (number)	$P\bar{1}$ (2)
a [Å]	8.5898(3)
b [Å]	12.1548(2)
c [Å]	19.3463(6)
α [°]	94.368(2)
β [°]	93.686(2)
γ [°]	109.796(2)
Volume [Å^3]	1886.25(10)
Z	4
ρ_{calc} [gcm^{-3}]	1.230
μ [mm^{-1}]	0.882

F(000)	720
Crystal size [mm^3]	0.253×0.062×0.06
Radiation	Cu K_α (λ=1.54184 Å)
2θ range [°]	4.60 to 157.53 (0.79 Å)
Index ranges	−10 ≤ h ≤ 10 −15 ≤ k ≤ 15 −24 ≤ l ≤ 24
Reflections collected	9147
Independent reflections	9147 R_{int} = 0.0330 R_{sigma} = 0.0225
Data / Restraints / Parameters	9147/4/464
Absorption correction T_{min}/T_{max} (method)	0.668/1.000 (gaussian)
Goodness-of-fit on F^2	1.042
Final *R* indexes [I≥2σ(I)]	R_1 = 0.0496 wR_2 = 0.1347
Final *R* indexes [all data]	R_1 = 0.0560 wR_2 = 0.1399
Largest peak/hole [eÅ^{-3}]	0.54/−0.26

N-(2-cyano-4-methoxyphenyl)-*N*-methylcyanamide (**3t**)

CCDC 2388837, melting point: 100.7 – 102.9 °C

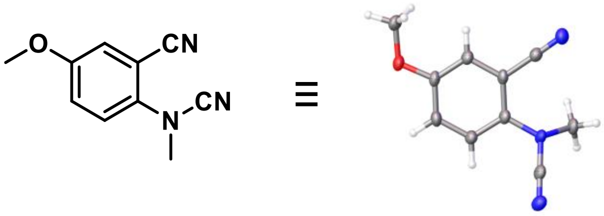

Identification code	bm540224_1_2
Empirical formula	$C_{10}H_9N_3O$
Formula weight	187.20
Temperature [K]	100.0(1)
Crystal system	monoclinic
Space group (number)	$P2/n$ (13)
a [Å]	8.7010(2)
b [Å]	3.83554(12)
c [Å]	27.7337(8)
α [°]	90
β [°]	98.826(3)
γ [°]	90
Volume [Å^3]	914.60(5)
Z	4
ρ_{calc} [gcm^{-3}]	1.360
μ [mm^{-1}]	0.756

F(000)	392
Crystal size [mm^3]	0.216×0.06×0.031
Radiation	Cu K_α (λ=1.54184 Å)
2θ range [°]	6.45 to 149.86 (0.80 Å)
Index ranges	−10 ≤ h ≤ 10<br>−4 ≤ k ≤ 4<br>−34 ≤ l ≤ 33
Reflections collected	17697
Independent reflections	1796<br>R_{int} = 0.0971<br>R_{sigma} = 0.0330
Data / Restraints / Parameters	1796/0/129
Absorption correction T_{min}/T_{max} (method)	0.885/1.000 (gaussian)
Goodness-of-fit on F^2	1.147
Final *R* indexes [I≥2 σ (I)]	R_1 = 0.0886<br>wR_2 = 0.2425
Final *R* indexes [all data]	R_1 = 0.0959<br>wR_2 = 0.2467
Largest peak/hole [eÅ^{-3}]	0.46/−0.45

7 REFERENCES

1. Rigaku. CrysAlisPro. (2016). Available at: https://rigaku.com/products/crystallography/x-ray-diffraction/crysalispro.

2. Sheldrick, G. M. SHELXT – Integrated space-group and crystal-structure determination. *Acta Crystallogr. Sect. A Found. Adv.* **71**, 3–8 (2015).

3. Sheldrick, G. M. A short history of SHELX. Acta Crystallogr. *Sect. A Found. Crystallogr.* **64**, 112–122 (2008).

4. Sheldrick, G. M. Crystal structure refinement with SHELXL. *Acta Crystallogr. Sect. C Struct. Chem.* **71**, 3–8 (2015).

5. Dolomanov, O. V., Bourhis, L. J., Gildea, R. J., Howard, J. A. K. & Puschmann, H. OLEX2 : a complete structure solution, refinement and analysis program. *J. Appl. Crystallogr.* **42**, 339–341 (2009).

6. Robert, E. G. L., Pirenne, V., Wodrich, M. D. & Waser, J. Donor-Acceptor Aminocyclobutane Monoesters: Synthesis and Silylium-Catalyzed (4+2) Annulation with Indoles. *Angew. Chemie Int. Ed.* **62**, e202302420 (2023).

7. Colley, H. E. et al. An Orally Bioavailable, Indole-3-glyoxylamide Based Series of Tubulin Polymerization Inhibitors Showing Tumor Growth Inhibition in a Mouse Xenograft Model of Head and Neck Cancer. *J. Med. Chem.* **58**, 9309–9333 (2015).

8. Sar, S., Tripathi, A., Dubey, K. D. & Sen, S. Iodine-Catalyzed Aerobic Diazenylation–Amination of Indole Derivatives. *J. Org. Chem.* **85**, 3748–3756 (2020).

9. Yadav, V., Jagtap, S. G., Balaraman, E. & Mhaske, S. B. Nickel-Catalyzed Direct Synthesis of N-Substituted Indoles from Amino Alcohols and Alcohols. *Org. Lett.* **24**, 9054–9059 (2022).

10. Su, Y. et al. Visible Light-Mediated C–H Difluoromethylation of Electron-Rich Heteroarenes. *Org. Lett.* **16**, 2958–2961 (2014).

11. Kim, J., Kim, H. & Chang, S. Copper-Mediated Selective Cyanation of Indoles and 2-Phenylpyridines with Ammonium Iodide and DMF. *Org. Lett.* **14**, 3924–3927 (2012).

12. Boutard, N. et al. Discovery and Structure–Activity Relationships of N -Aryl 6-Aminoquinoxalines as Potent PFKFB3 Kinase Inhibitors. *ChemMedChem* **14**, 169–181 (2019).

13. Summer, S. L. et al. Di-aryl Sulfonamide Motif Adds π-Stacking Bulk in Negative Allosteric Modulators of the NMDA Receptor. *ACS Med. Chem. Lett.* **10**, 248–254 (2019).

14. Swanger, S. A. et al. A Novel Negative Allosteric Modulator Selective for GluN2C/2D-Containing NMDA Receptors Inhibits Synaptic Transmission in Hippocampal Interneurons. *ACS Chem. Neurosci.* **9**, 306–319 (2018).

15. Shet, H. et al. Cu(II)/PTABS-Promoted, Regioselective S N Ar Amination of Polychlorinated Pyrimidines with Mechanistic Understanding. *J. Org. Chem.* **88**, 11036–11044 (2023).

16. Ji, X. et al. Aerobic C–C Bond Cleavage of Indoles by Visible-Light Photoredox Catalysis with $Ru(bpy)_3^{2+}$. *European J. Org. Chem.* 6652–6659 (2017).

17. Clark, P. G. K., Lein, M. & Keyzers, R. A. Studies of the H–D exchange mechanism of malonganenone B. *Org. Biomol. Chem.* **10**, 1725 (2012).

18. Shyshkanov, S., Nguyen, T. N., Ebrahim, F. M., Stylianou, K. C. & Dyson, P. J. In Situ Formation of Frustrated Lewis Pairs in a Water-Tolerant Metal-Organic Framework for the Transformation of CO 2. *Angew. Chemie Int. Ed.* **58**, 5371–5375 (2019).

19. Li, H. et al. Methanol as the C 1 source: redox coupling of nitrobenzenes and alcohols for the synthesis of benzimidazoles. *Green Chem.* **24**, 748–753 (2022).

20. Gallage, P. C., McKee, M. G. & Pitre, S. P. 1,4-Dihydropyridine Anions as Potent Single-Electron Photoreductants. *Org. Lett.* **26**, 1975–1979 (2024).

21. Kanitz, N. E. & Lindel, T. Photoreactivity of monofluorinated 2-azidobenzimidazoles towards carboxylic acids. *Zeitschrift für Naturforsch. B* **71**, 1287–1300 (2016).

22. Nakao, Y., Kanyiva, K. S., Oda, S. & Hiyama, T. Hydroheteroarylation of Alkynes under Mild Nickel Catalysis. *J. Am. Chem. Soc.* **128**, 8146–8147 (2006).

23. Soria-Arteche, O. et al. Synthesis and antiprotozoal activity of nitazoxanide–N-methylbenzimidazole hybrids. *Bioorg. Med. Chem. Lett.* **23**, 6838–6841 (2013).

24. Dong, Y. & Breit, B. Cu-Catalyzed C–H Allylation of Benzimidazoles with Allenes. *Org. Lett.* **23**, 6765–6769 (2021).

25. Fu, X. et al. Carbon Chain Rupture: Base-Induced Radical C–C Bond Cleavage of Alkylbenzimidazoles. *Synthesis (Stuttg).* **54**, 4481–4494 (2022).

26. Zhou, J. et al. Synthesis and antiproliferative evaluation of novel benzoimidazole-contained oxazole-bridged analogs of combretastatin A-4. Eur. *J. Med. Chem.* **68**, 222–232 (2013).

27. Zbancioc, G., Mangalagiu, I. I. & Moldoveanu, C. Ultrasound assisted synthesis of imidazolium salts: An efficient way to ionic liquids. *Ultrason. Sonochem.* **23**, 376–384 (2015).

8 NMR Spectra of Starting Materials

1-methyl-1*H*-indole-2-^{13}C (**^{13}C-1a**)

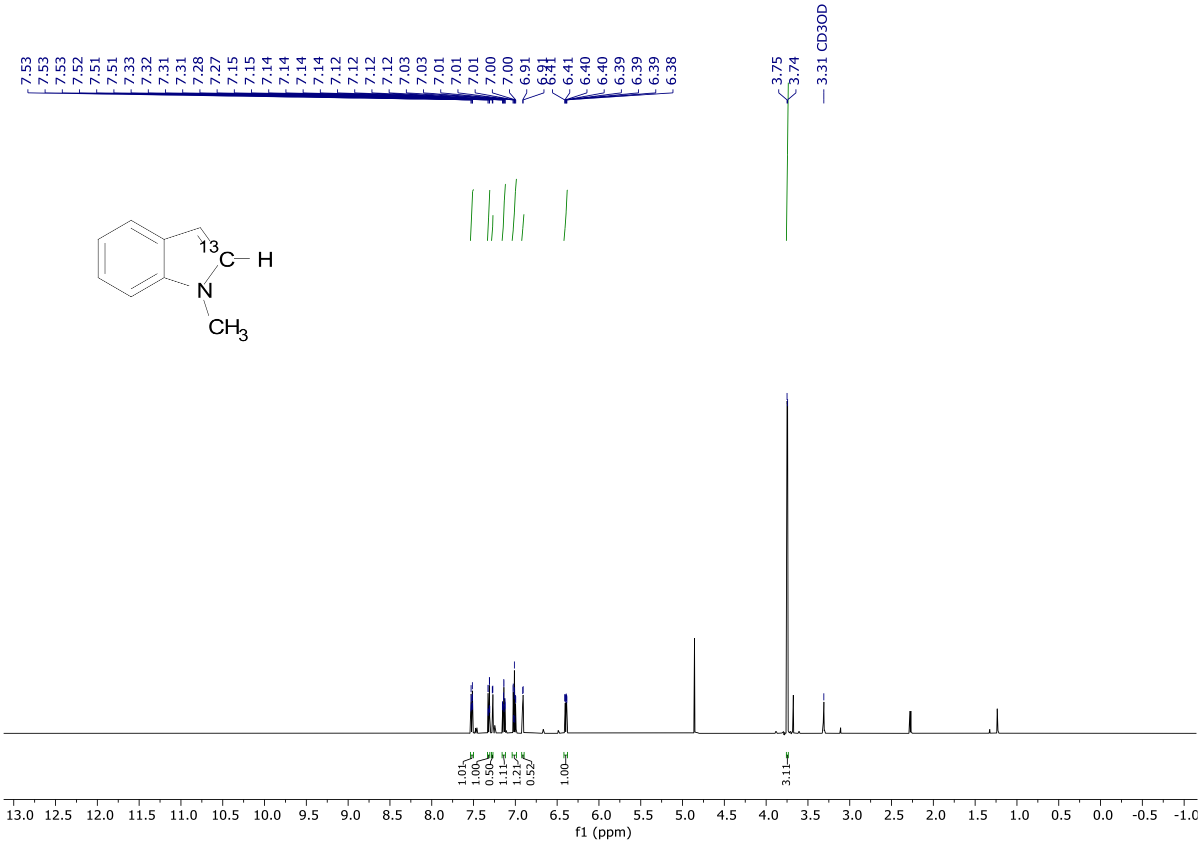

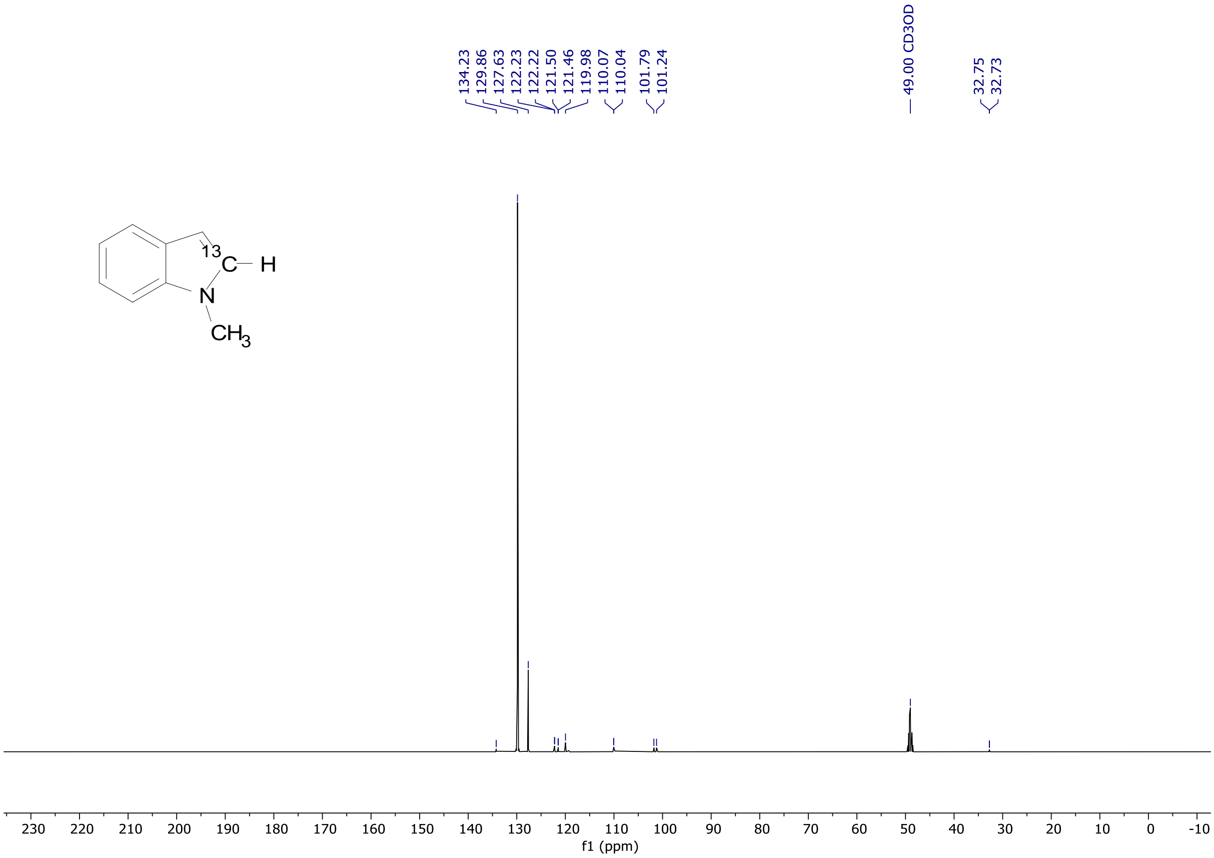

6-Methoxy-1-methyl-1*H*-indole (**1b**)

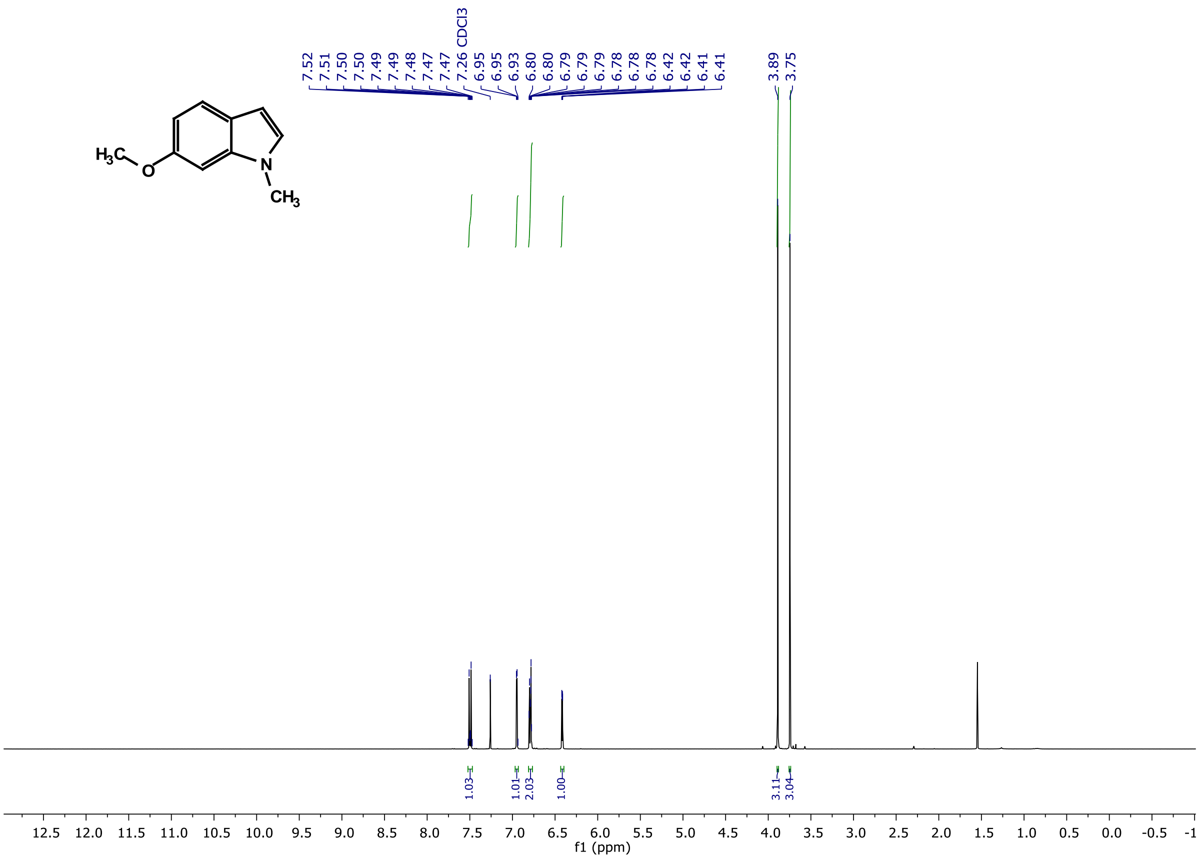

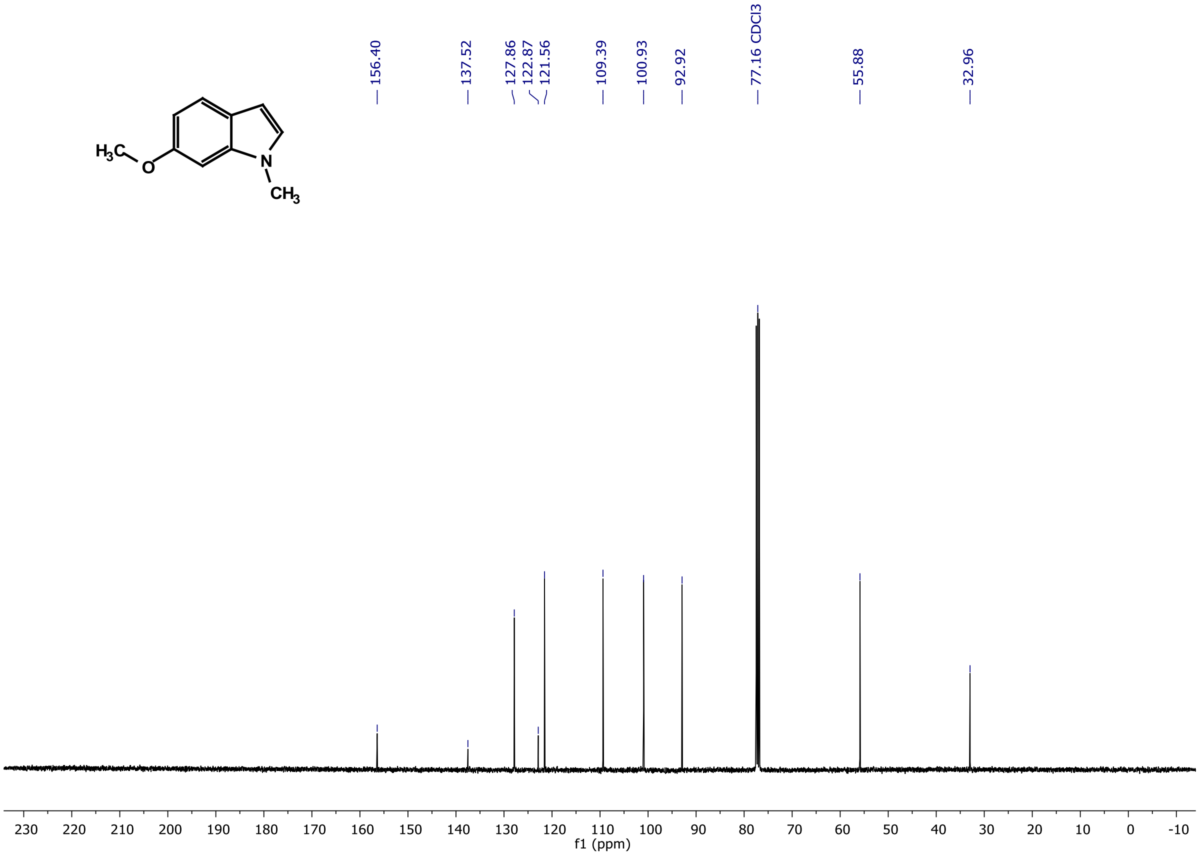

1-Ethyl-6-methoxy-1H-indole (**1c**)

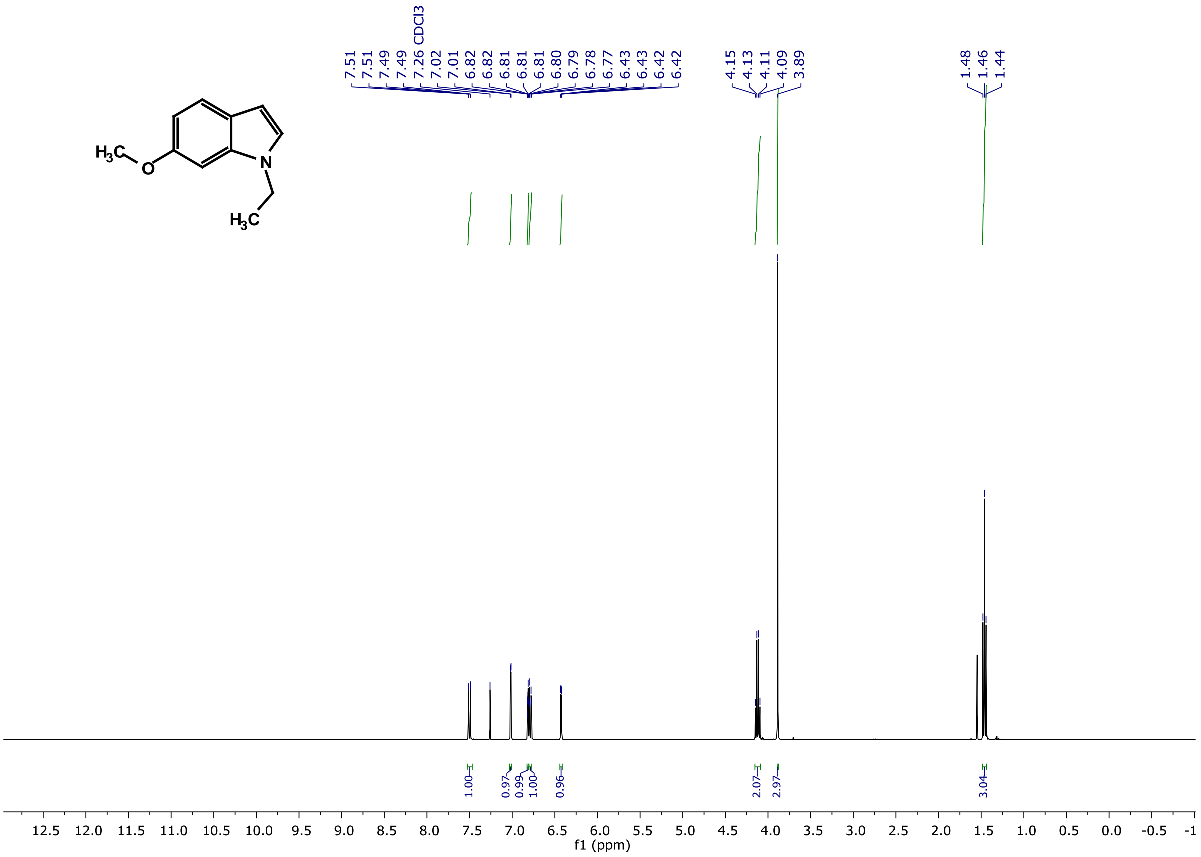

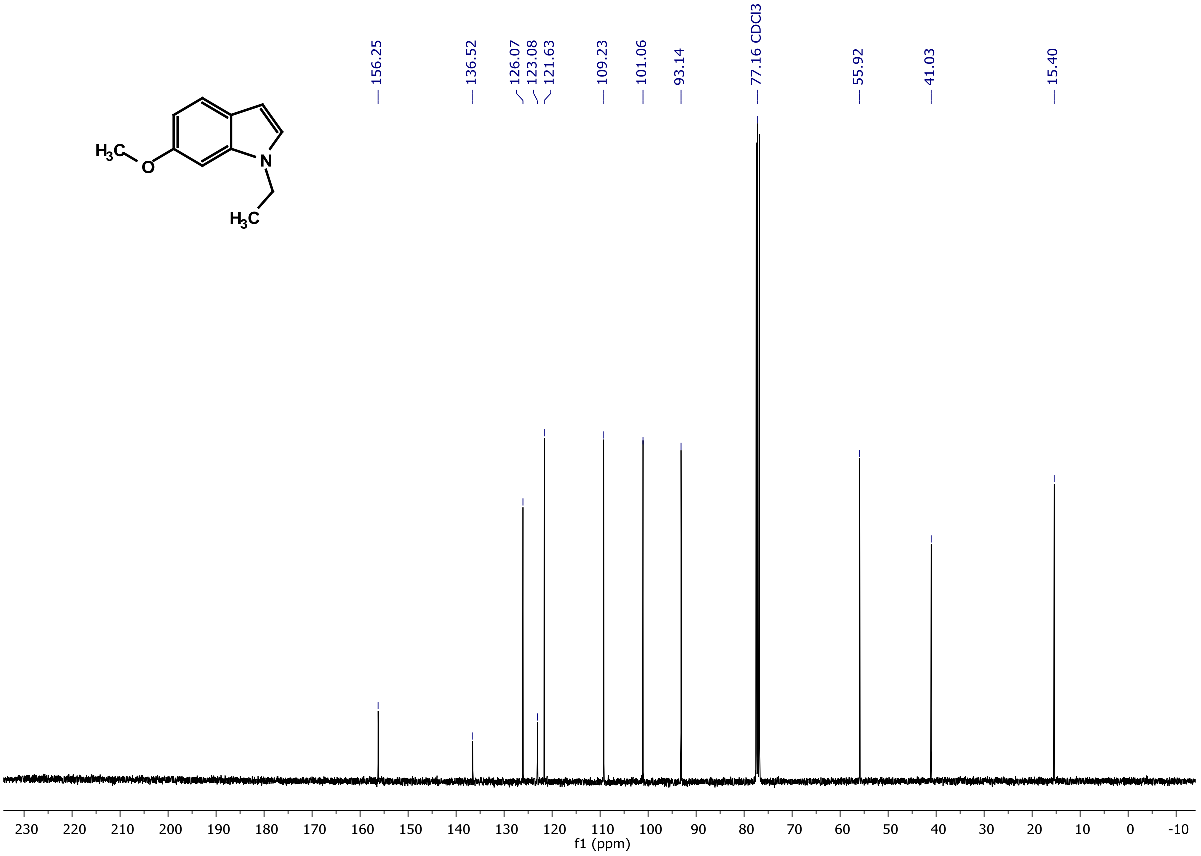

1-Isopropyl-6-methoxy-1H-indole (**1d**)

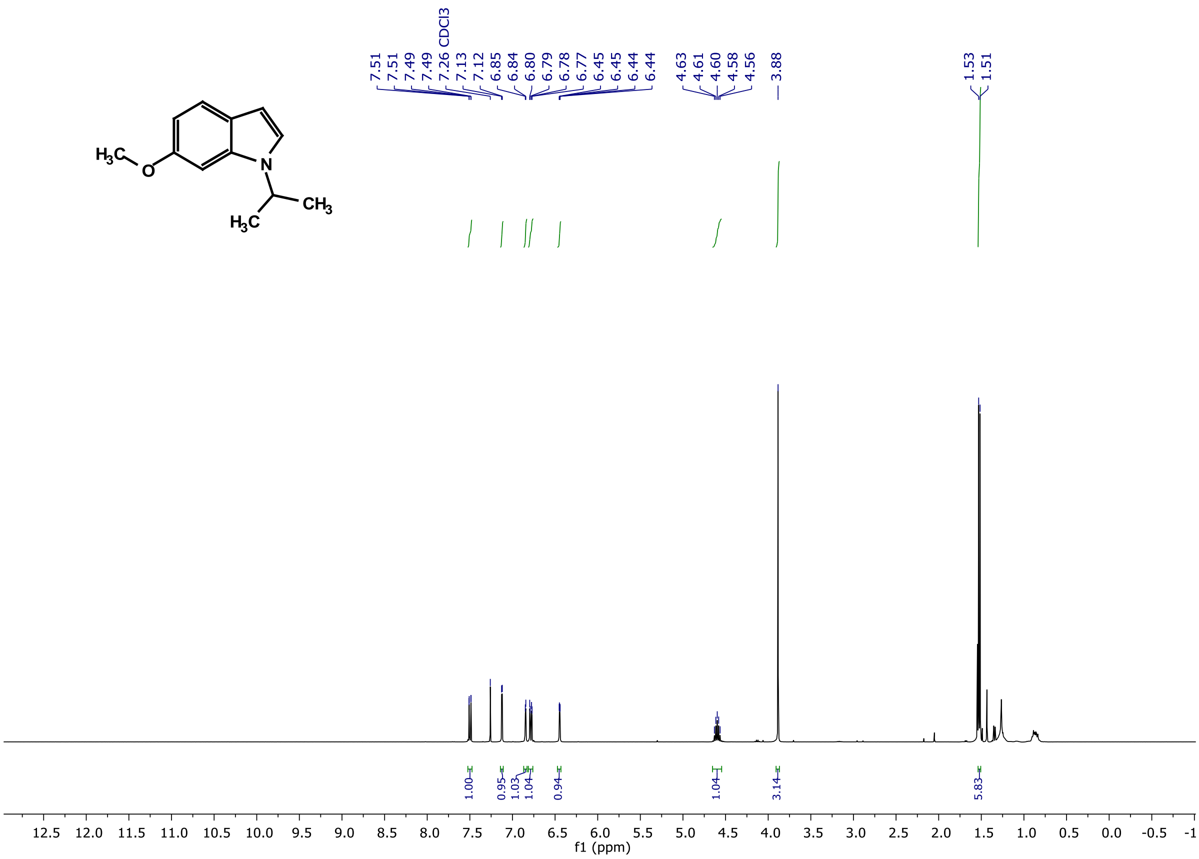

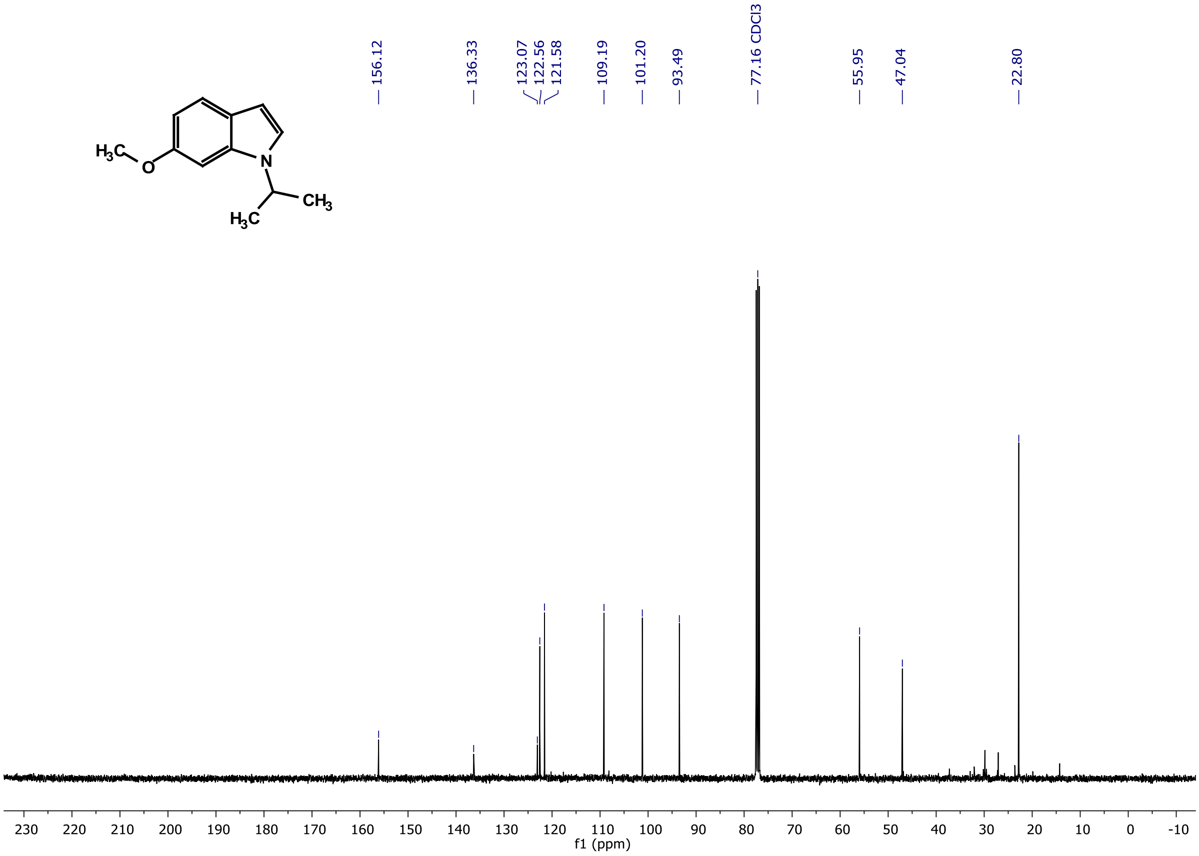

1-Allyl-6-methoxy-1H-indole (**1e**)

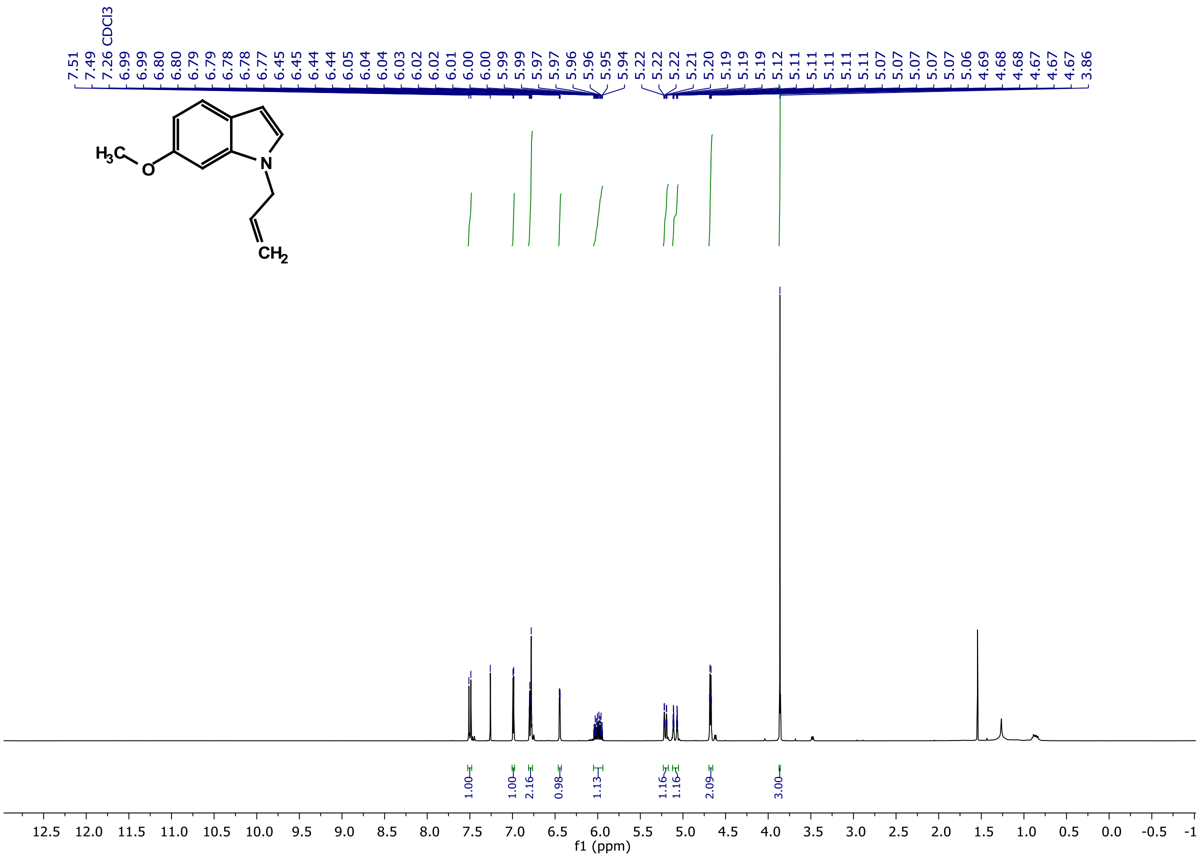

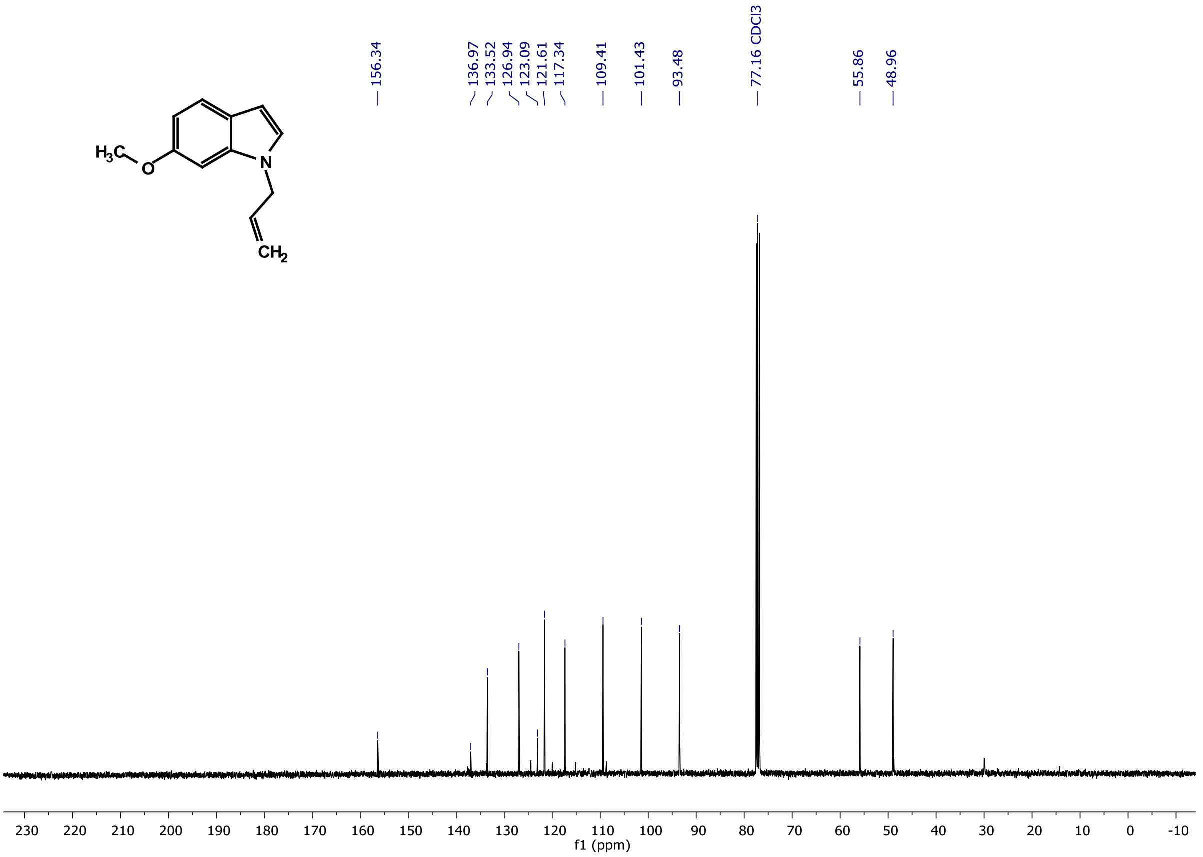

1-Benzyl-6-methoxy-1H-indole (**1f**)

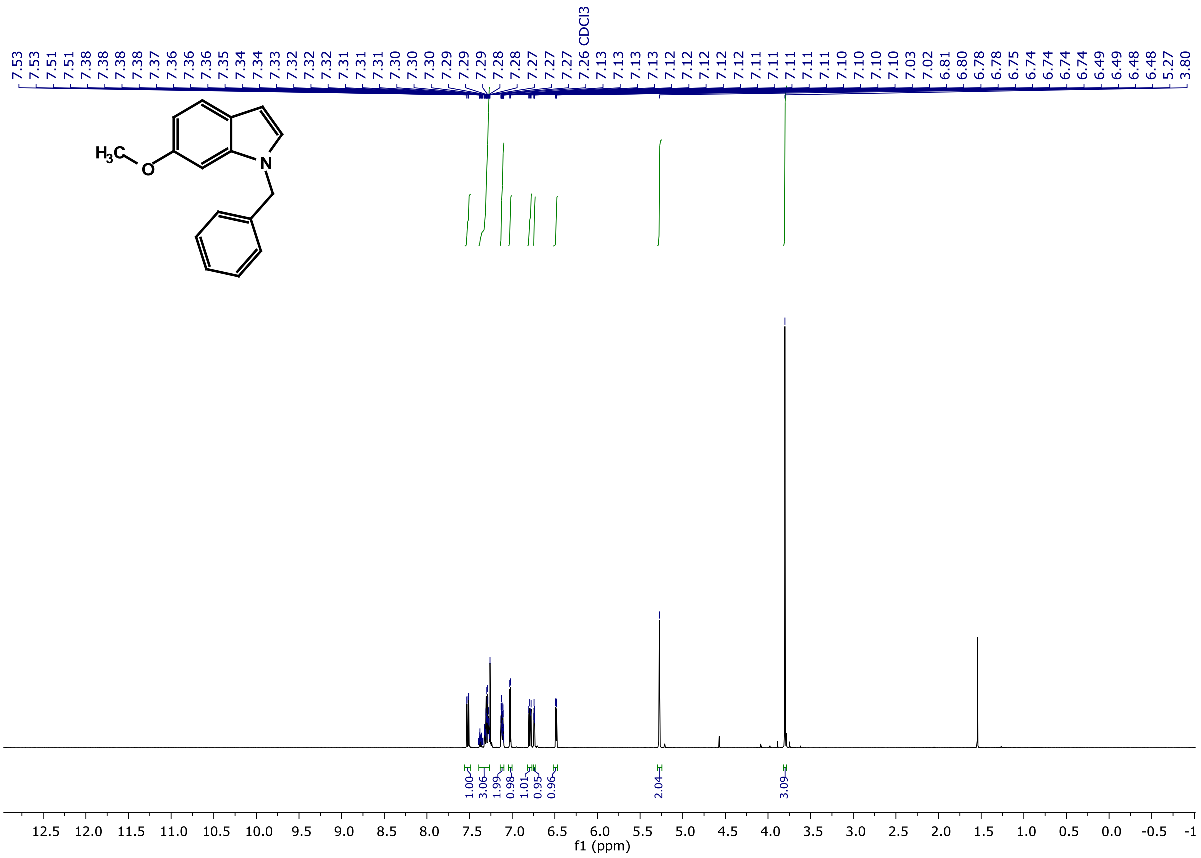

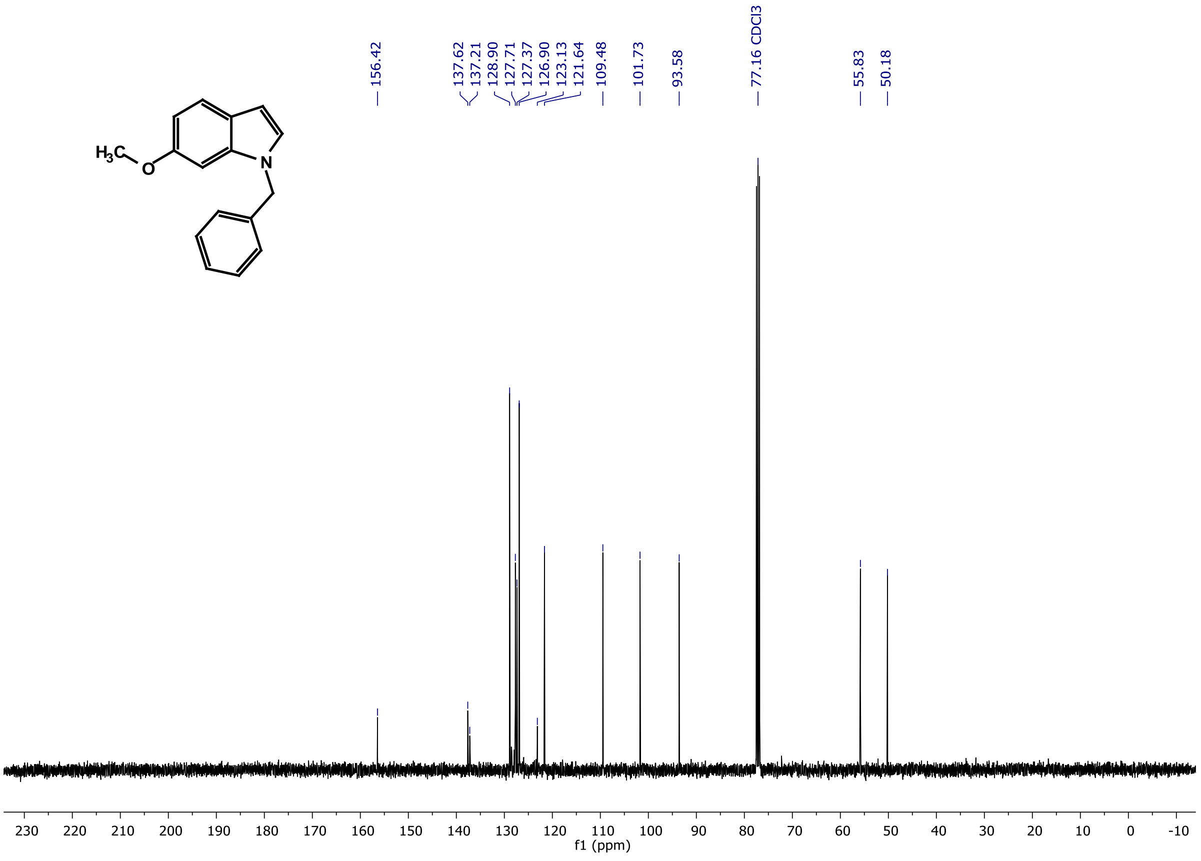

1-Benzyl-6-fluoro-1H-indole (**1g**)

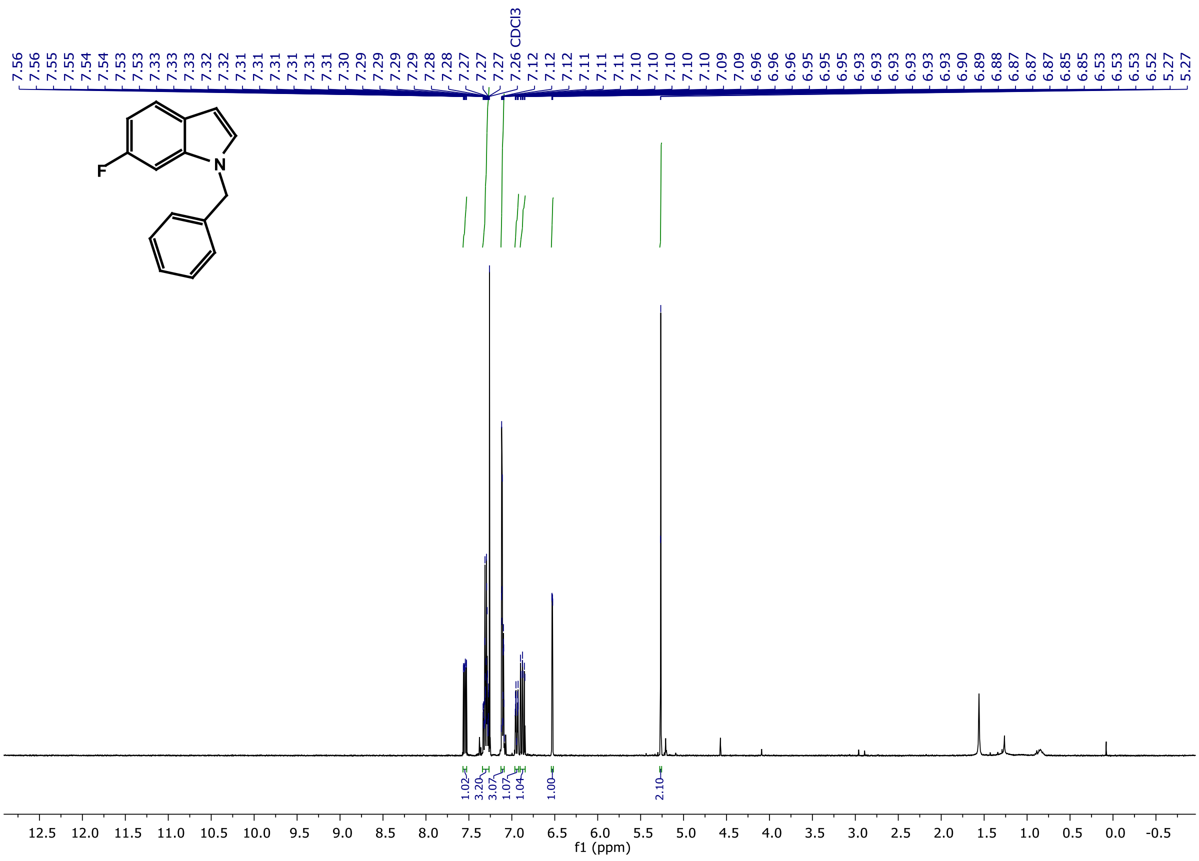

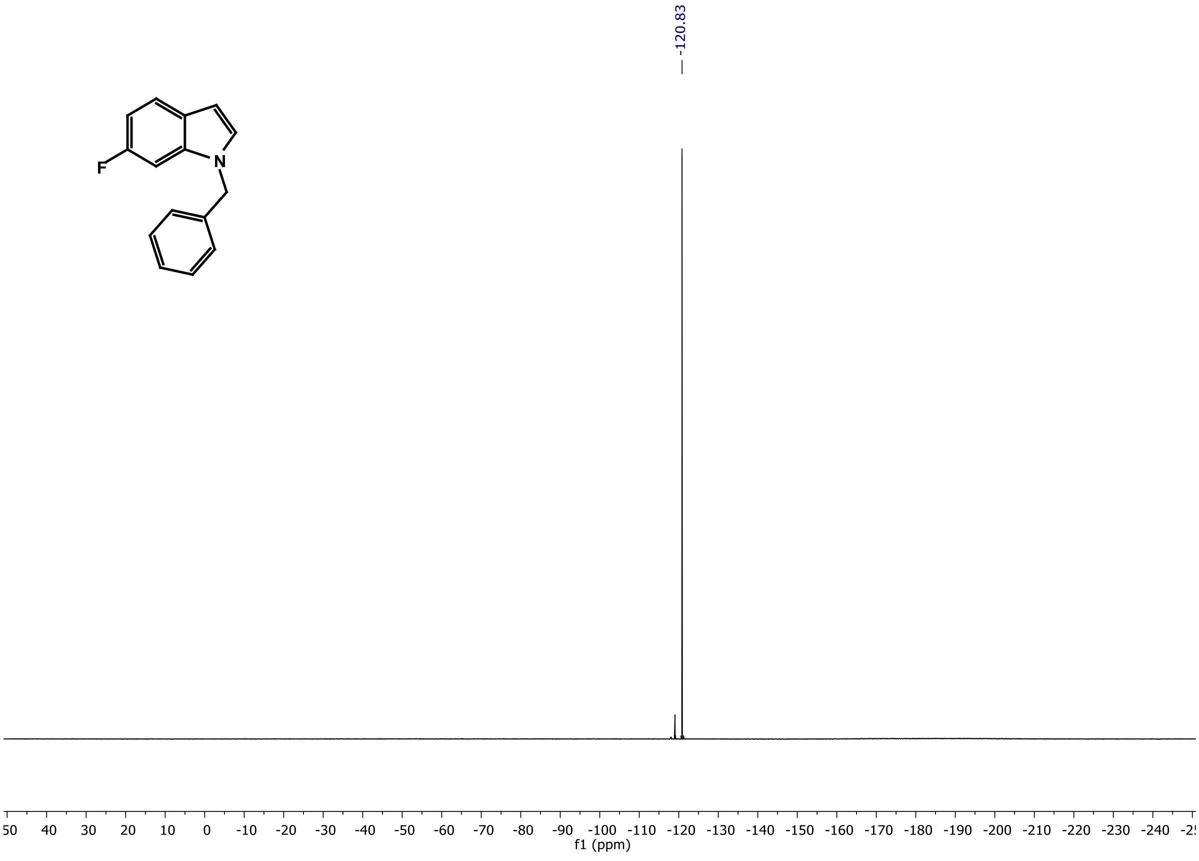

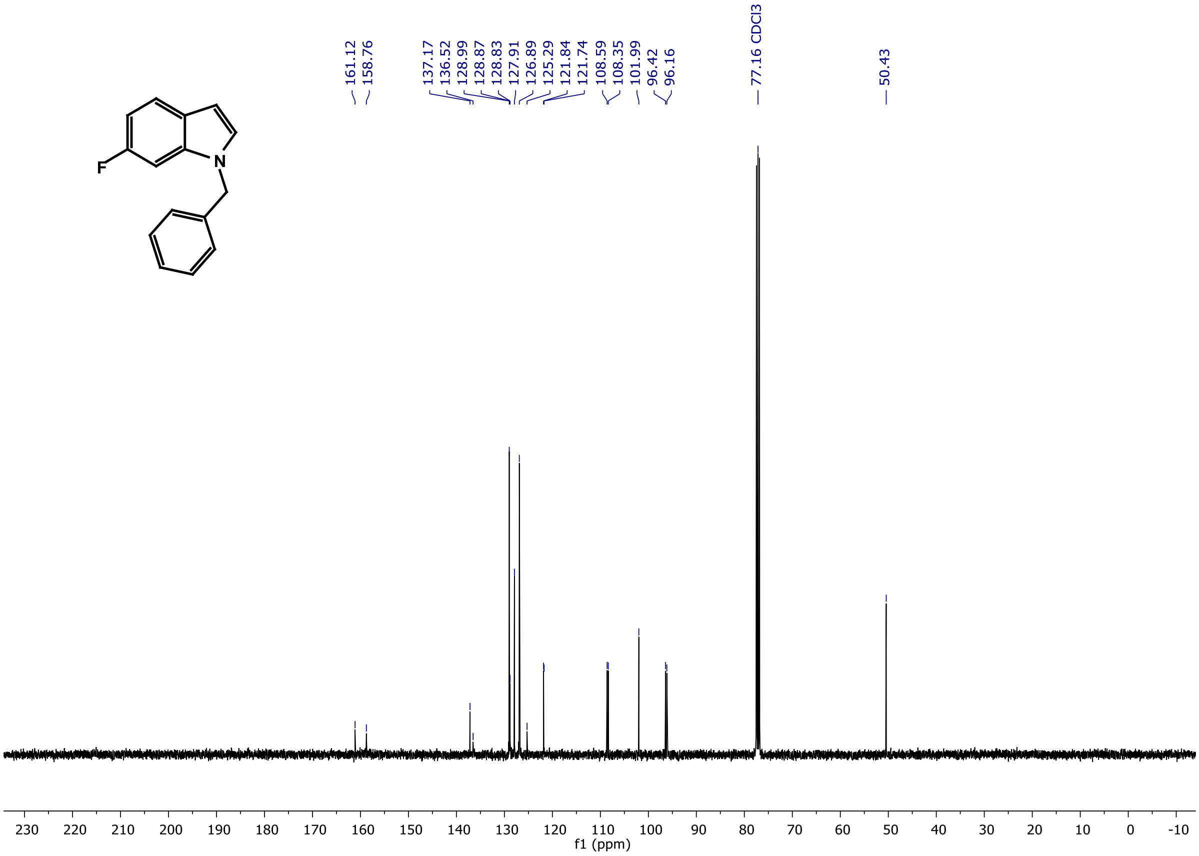

6-Fluoro-1-methyl-1H-indole (**1i**)

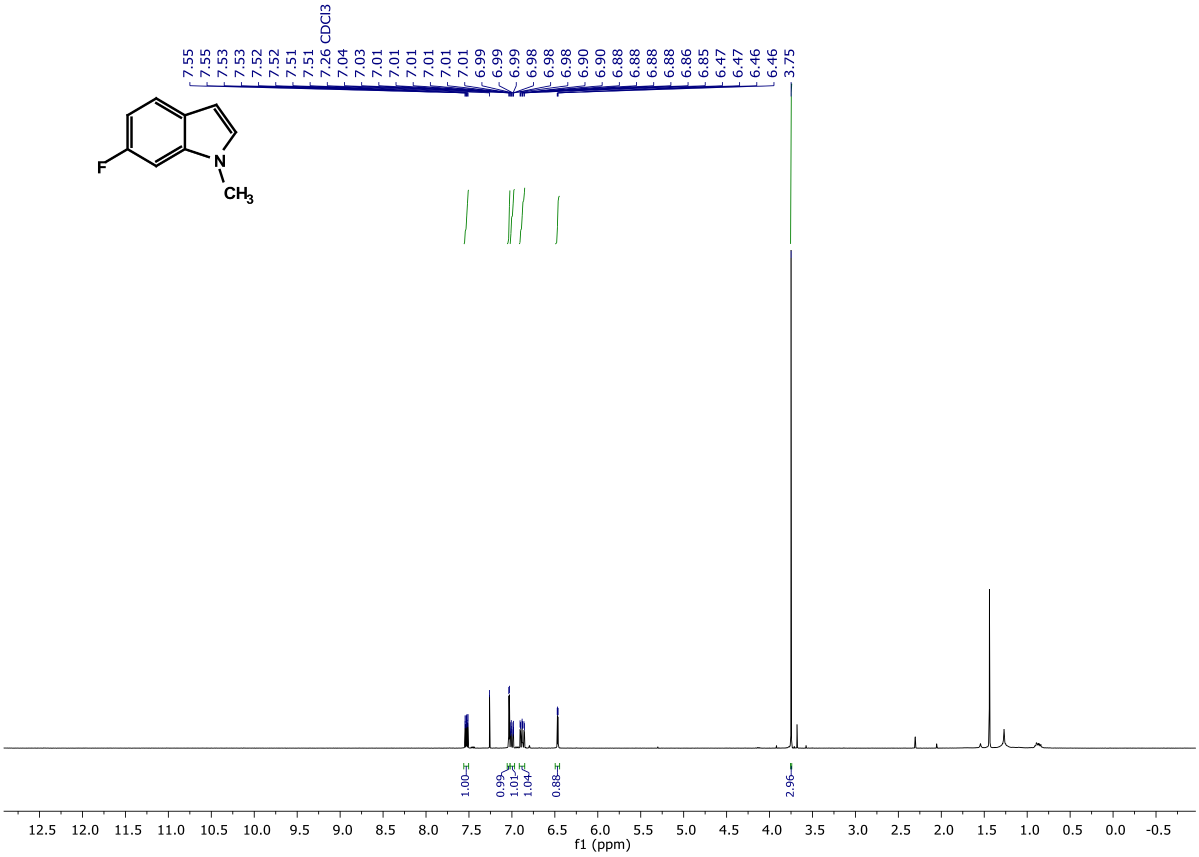

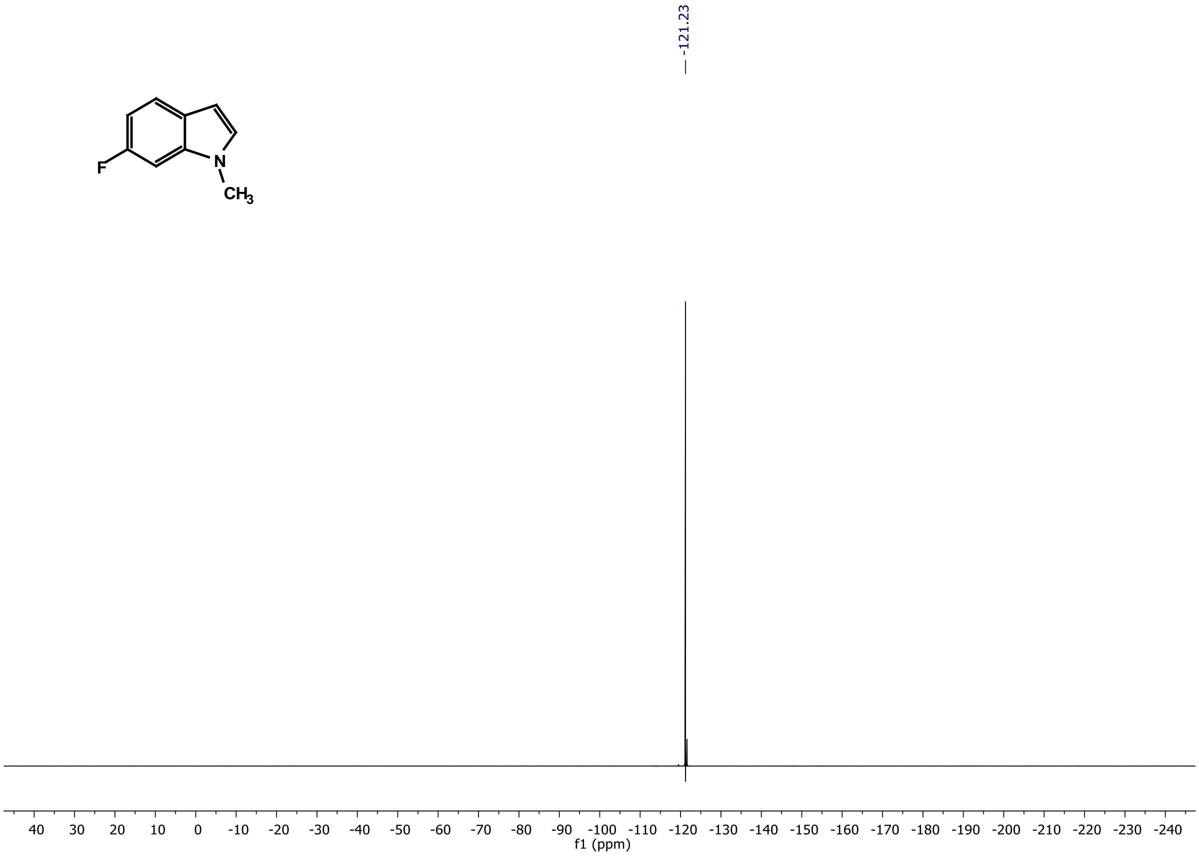
-121.23
F
N
CH3
40 30 20 10 0 -10 -20 -30 -40 -50 -60 -70 -80 -90 -100 -110 -120 -130 -140 -150 -160 -170 -180 -190 -200 -210 -220 -230 -240
f1 (ppm)

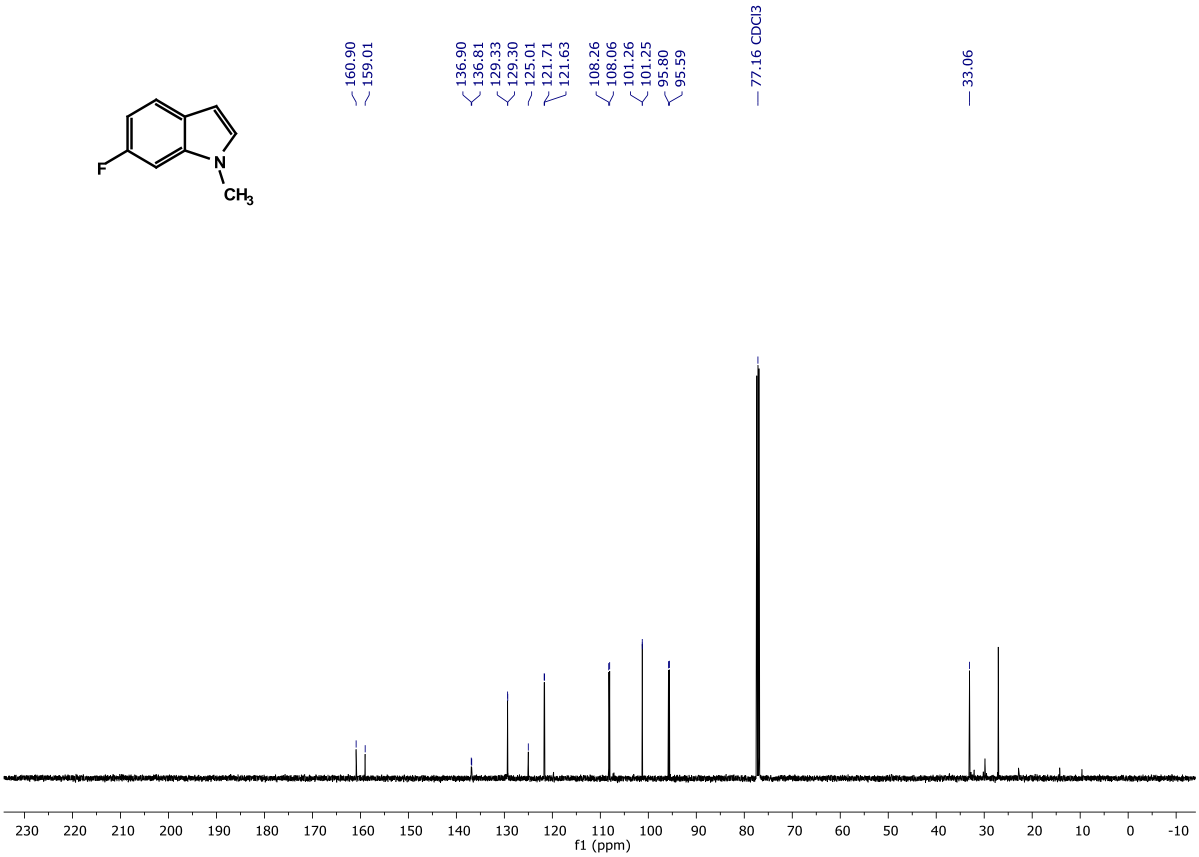
160.90
159.01
136.90
136.81
129.33
129.30
125.01
121.71
121.63
108.26
108.06
101.26
101.25
95.80
95.59
77.16 CDCl3
33.06
F
N
CH3
230 220 210 200 190 180 170 160 150 140 130 120 110 100 90 80 70 60 50 40 30 20 10 0 -10
f1 (ppm)

6-Chloro-1-methyl-1*H*-indole (**1j**)

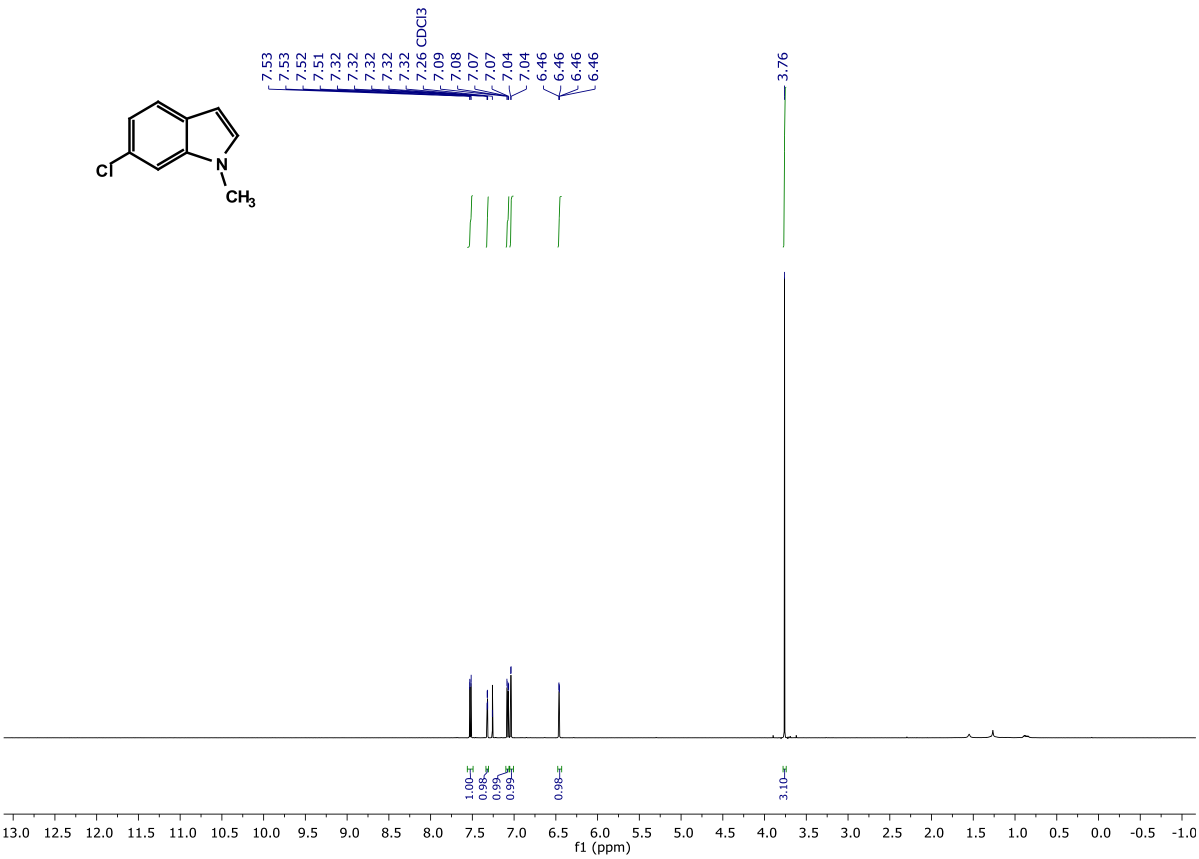

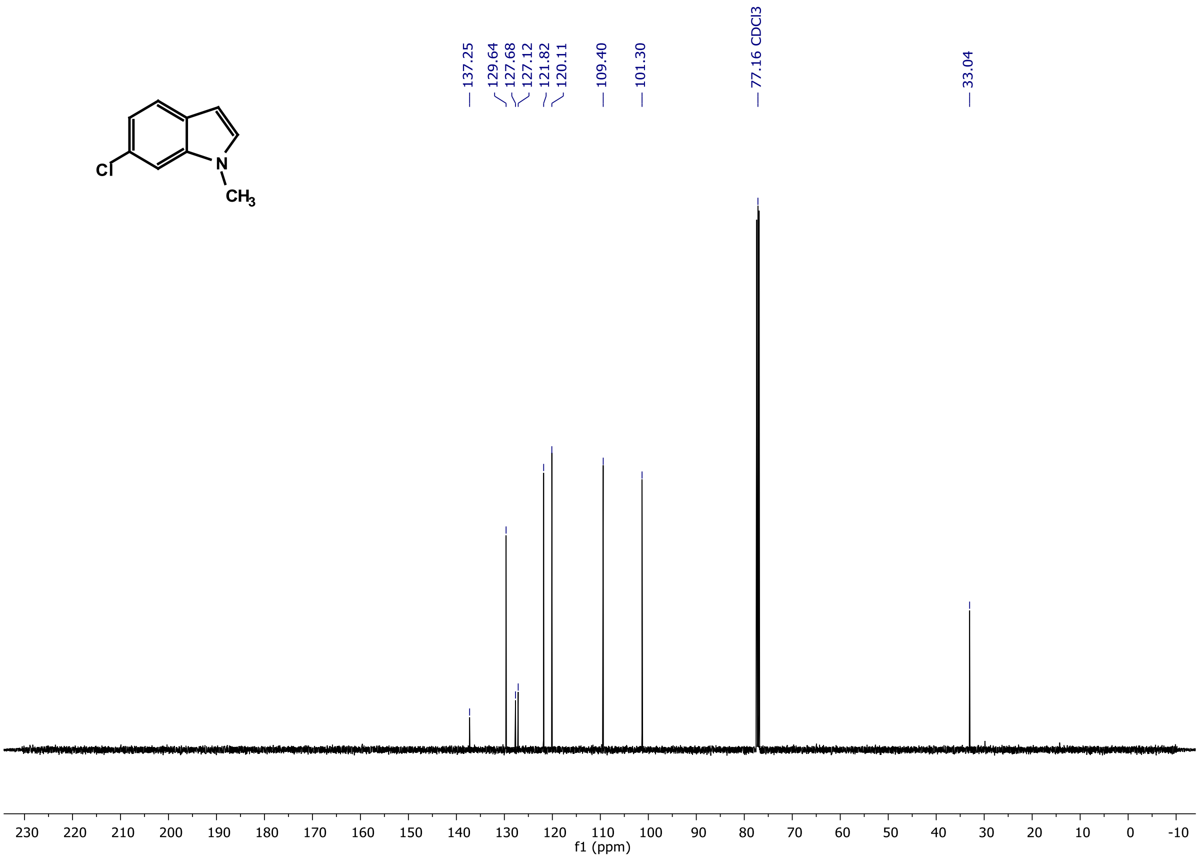

6-Bromo-1-methyl-1*H*-indole (**1k**)

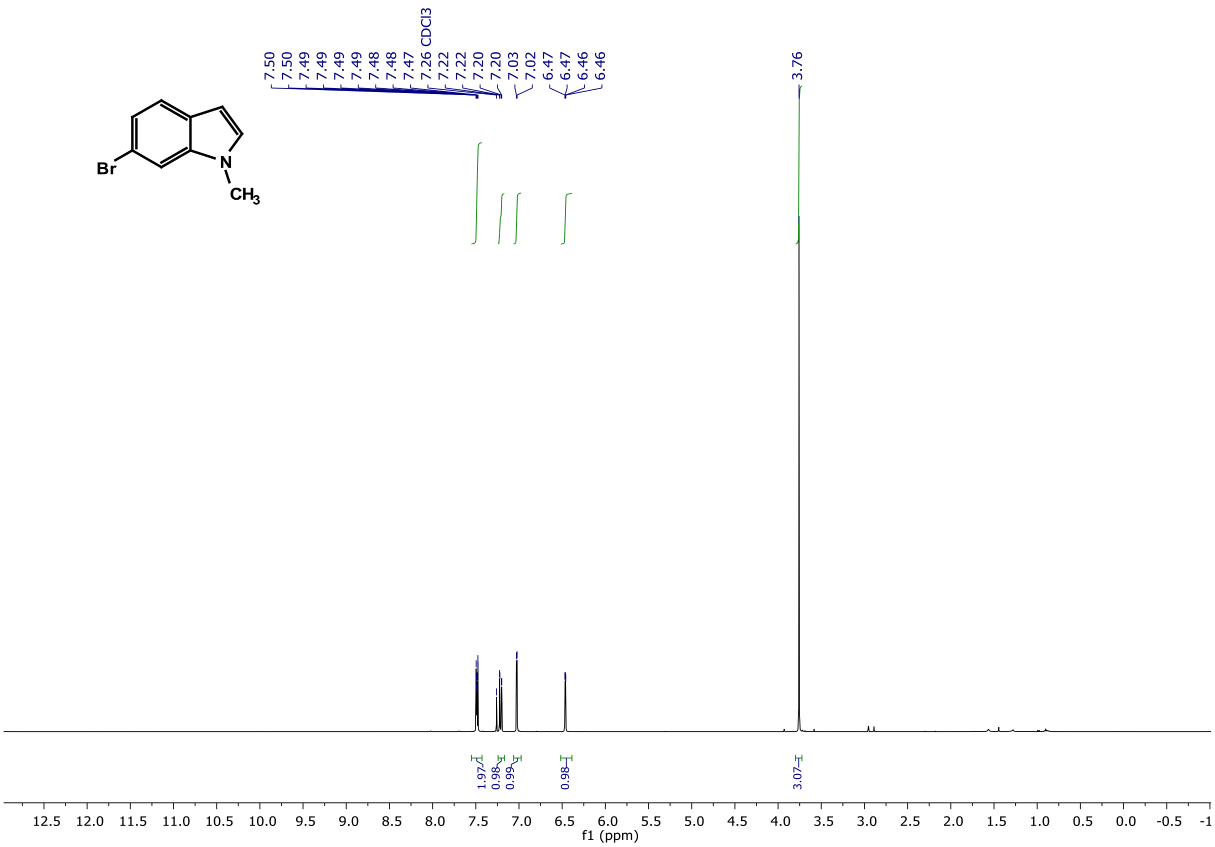

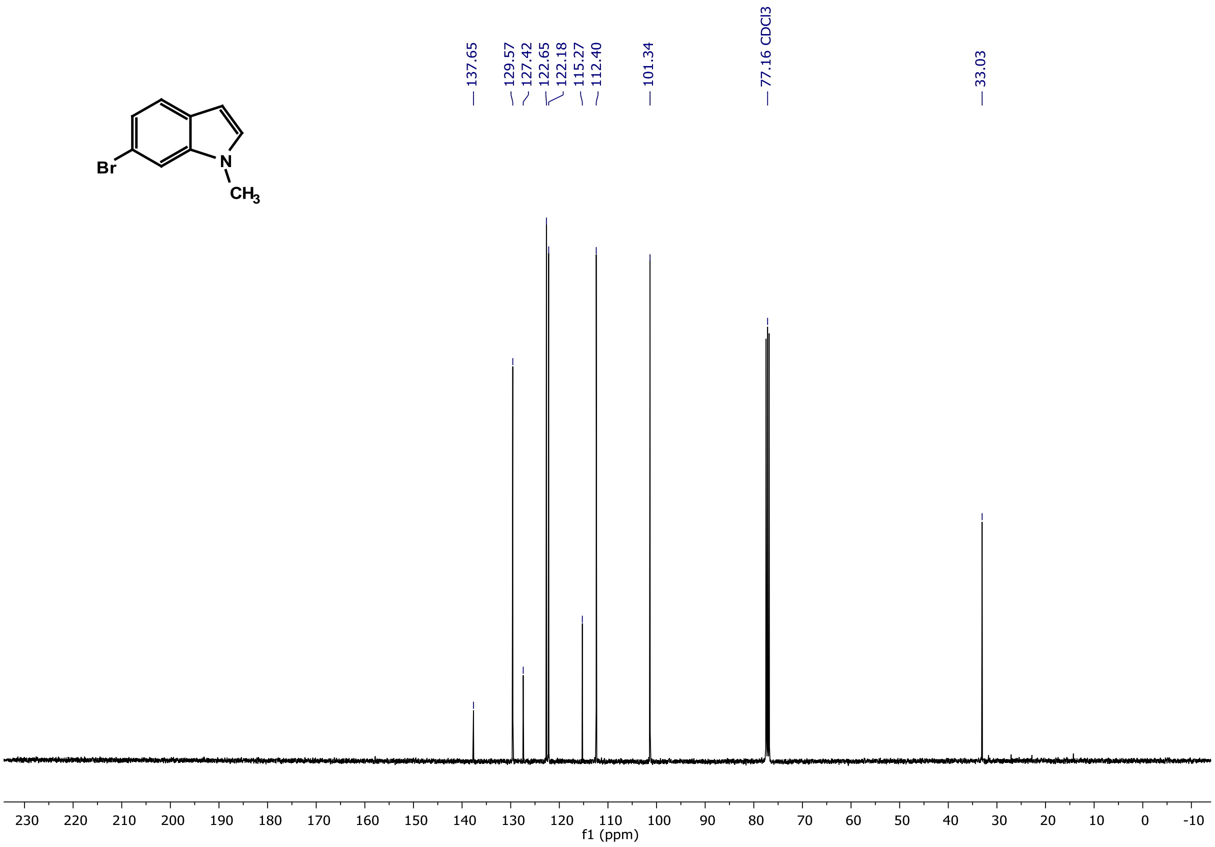

6-(Benzyloxy)-1-methyl-1H-indole (**1m**)

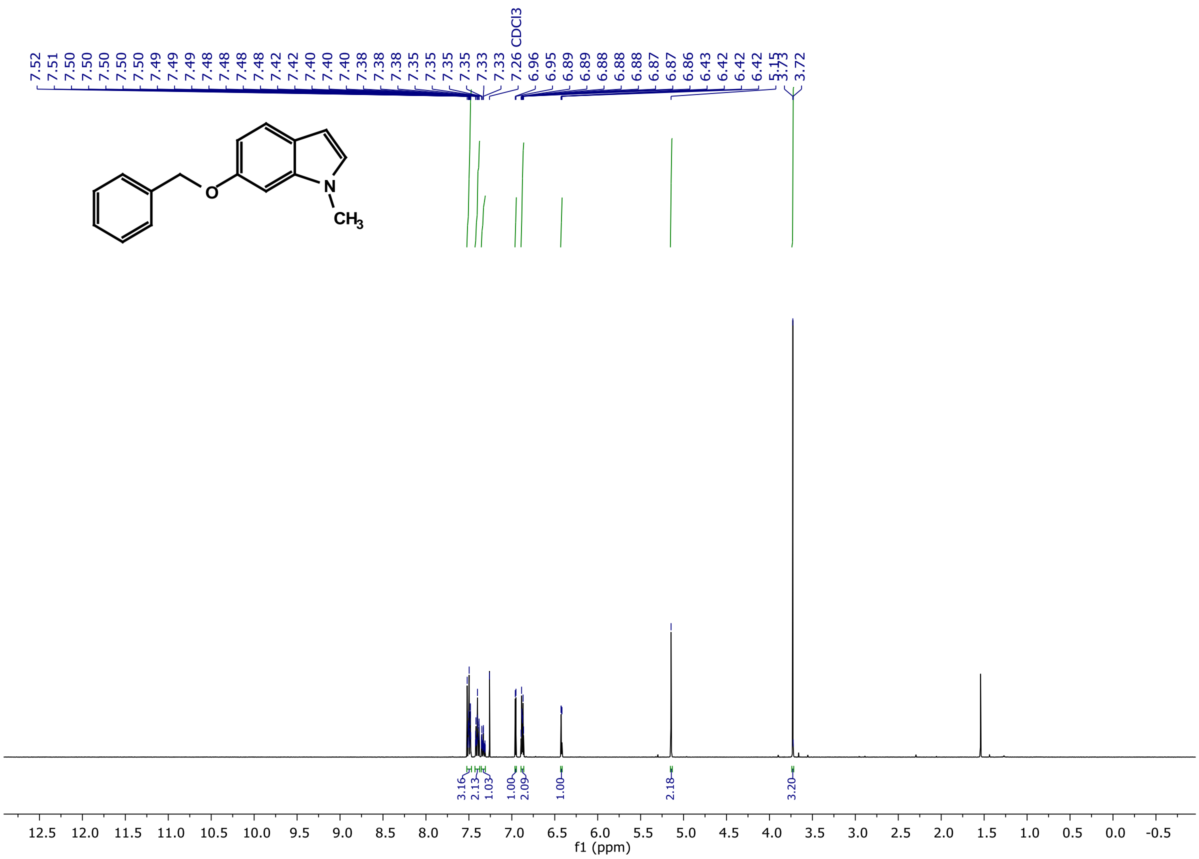

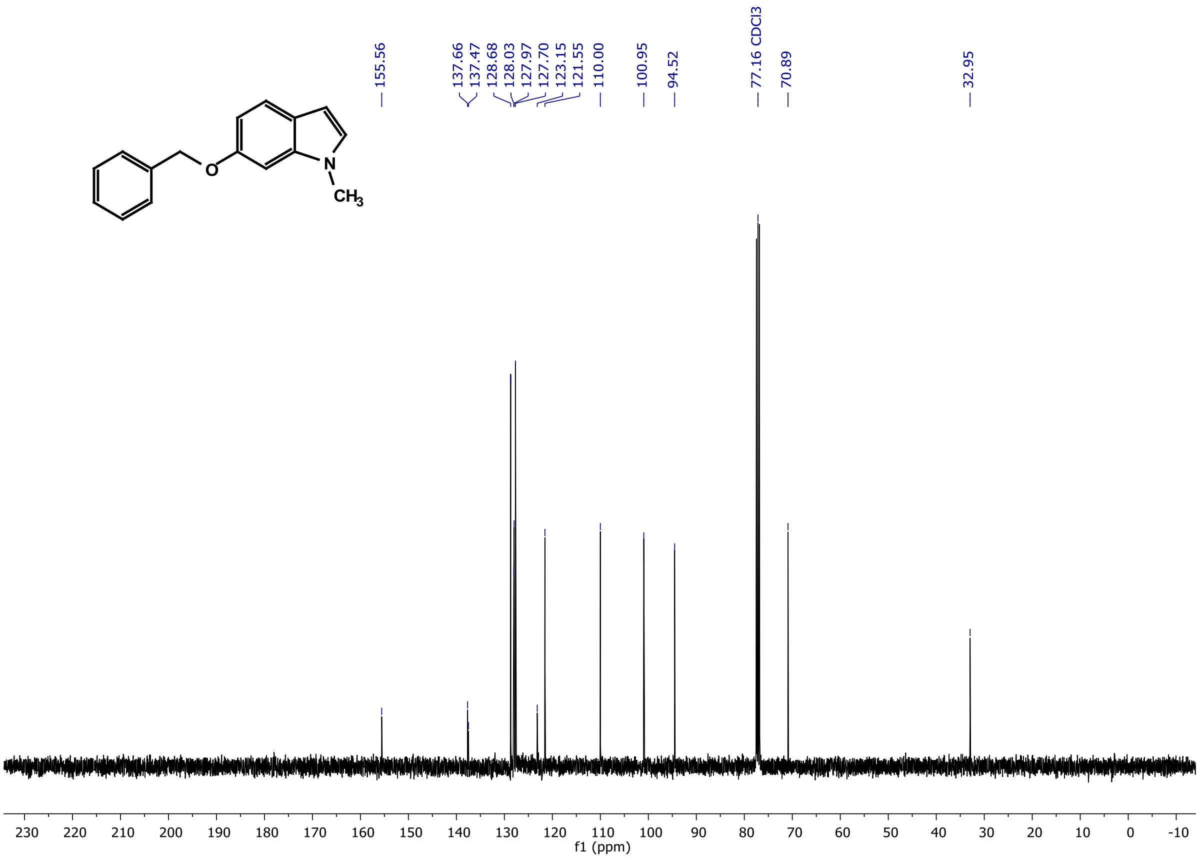

1-Methyl-6-(pyridin-2-yl)-1*H*-indole (**1n**)

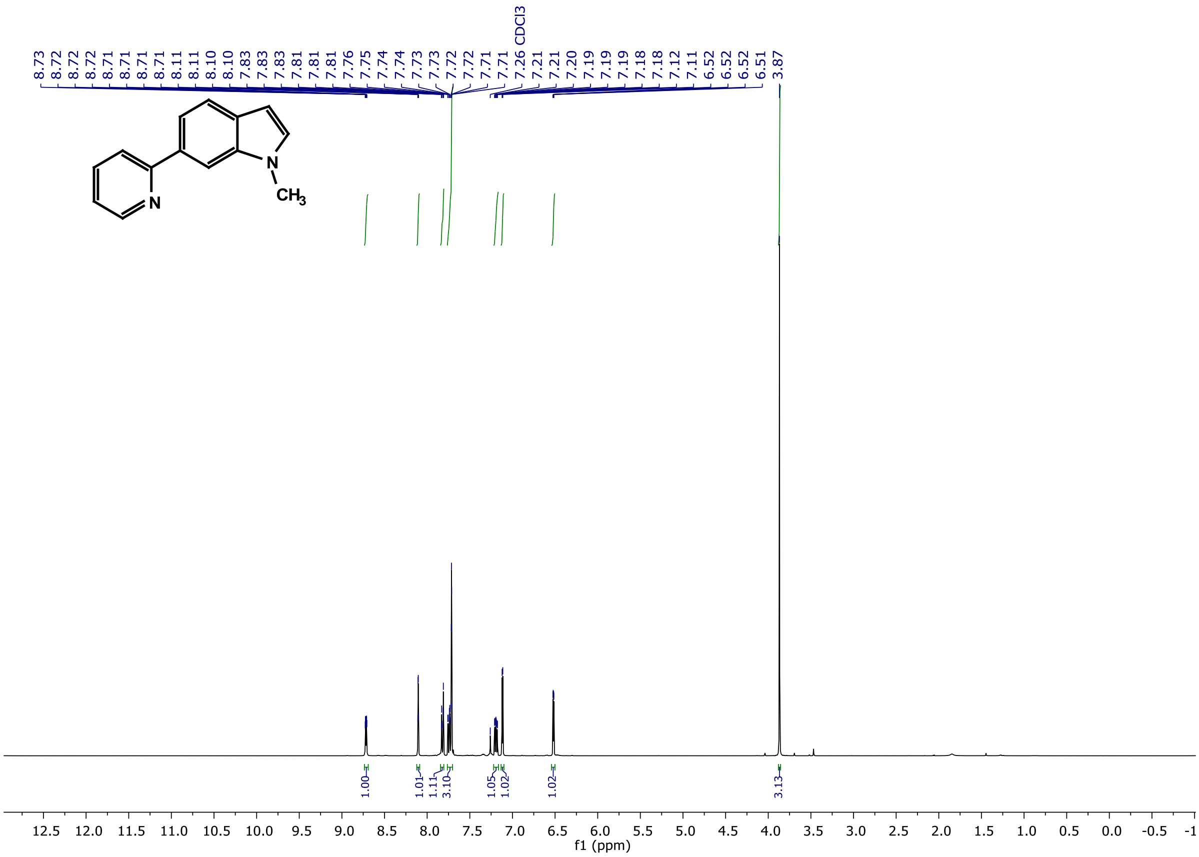

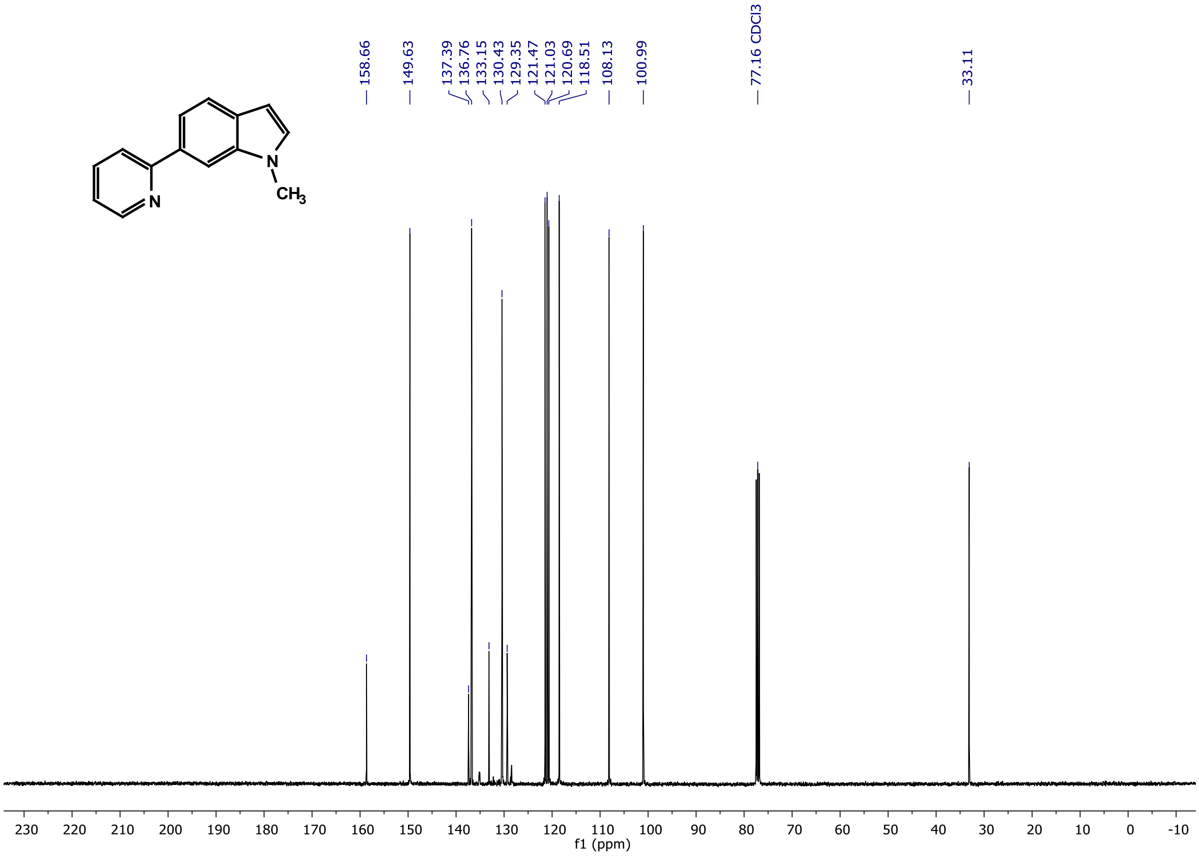

6-(6-chloro-2-methylpyrimidin-4-yl)-1-methyl-1H-indole (**1o**)

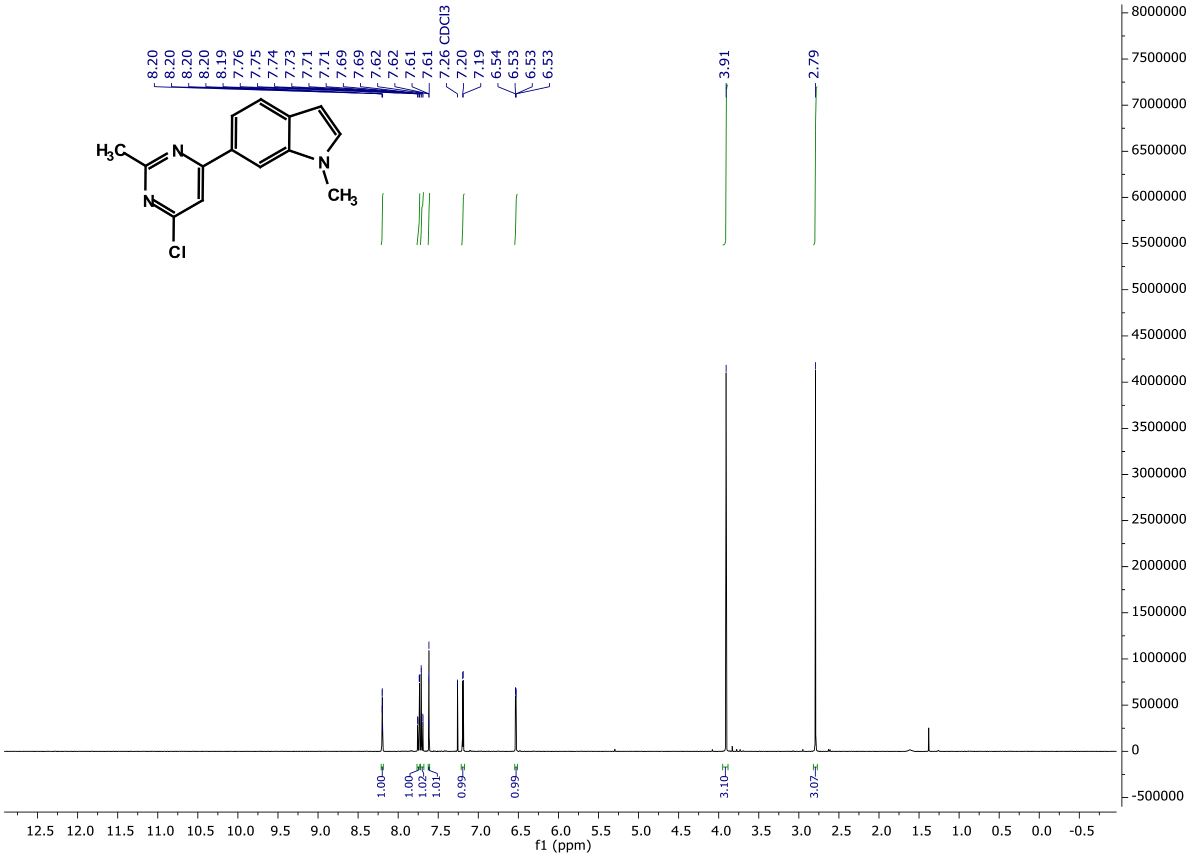

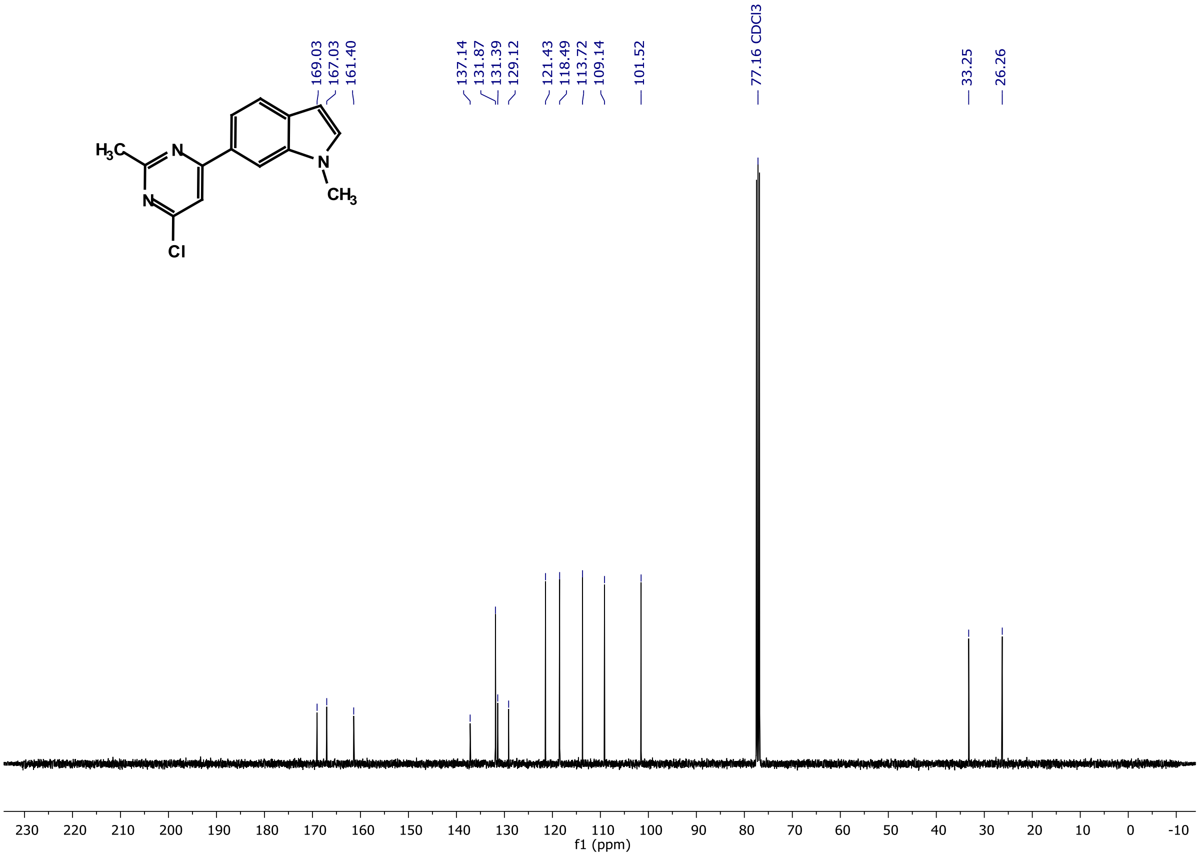

1-Methyl-6-(4-(methylsulfonyl)phenyl)-1*H*-indole (**1p**)

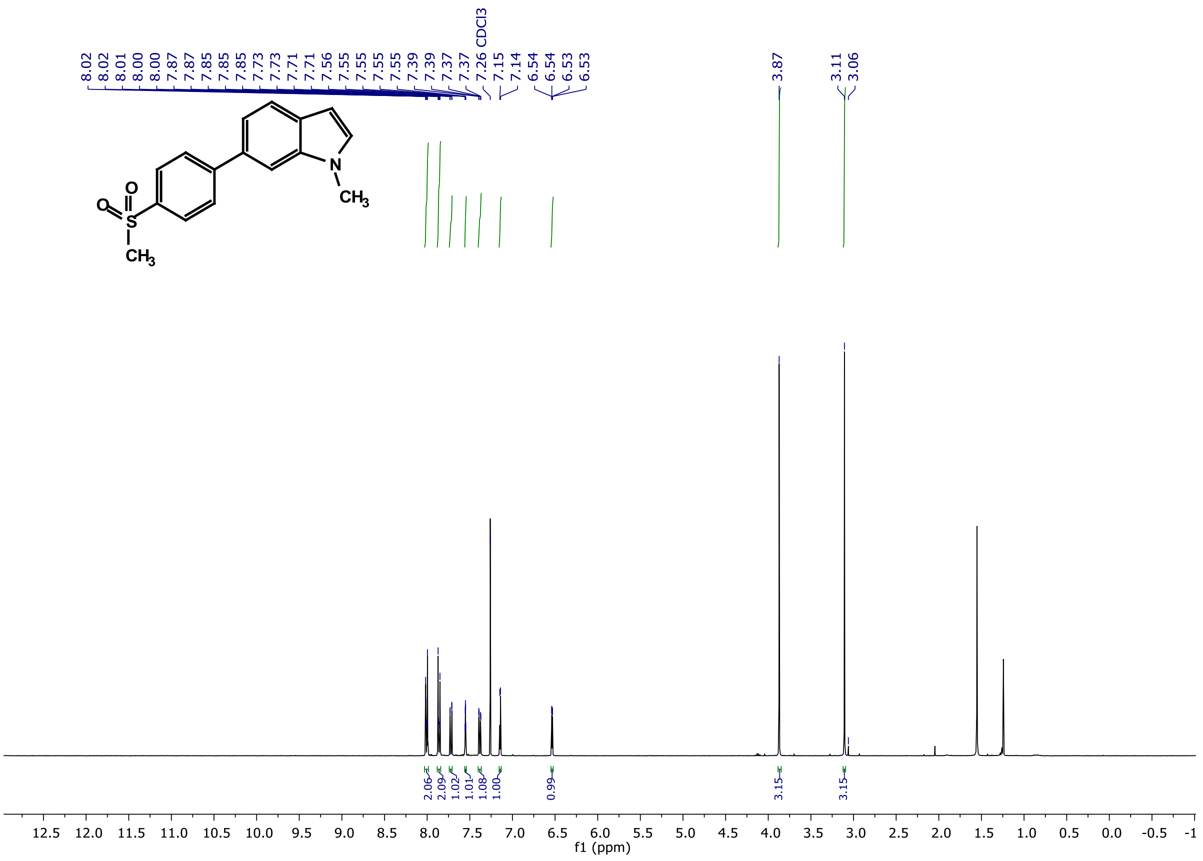

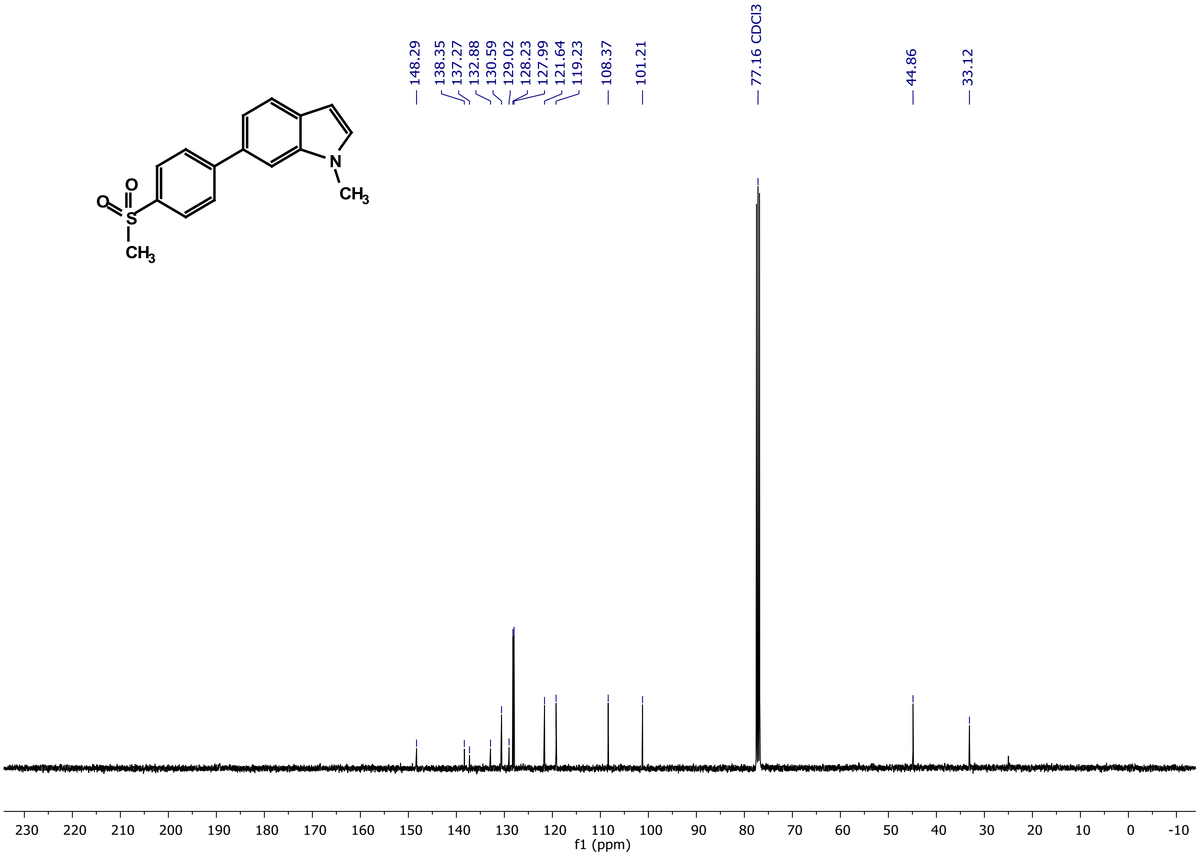

5-Chloro-1-methyl-1*H*-indole (**1q**)

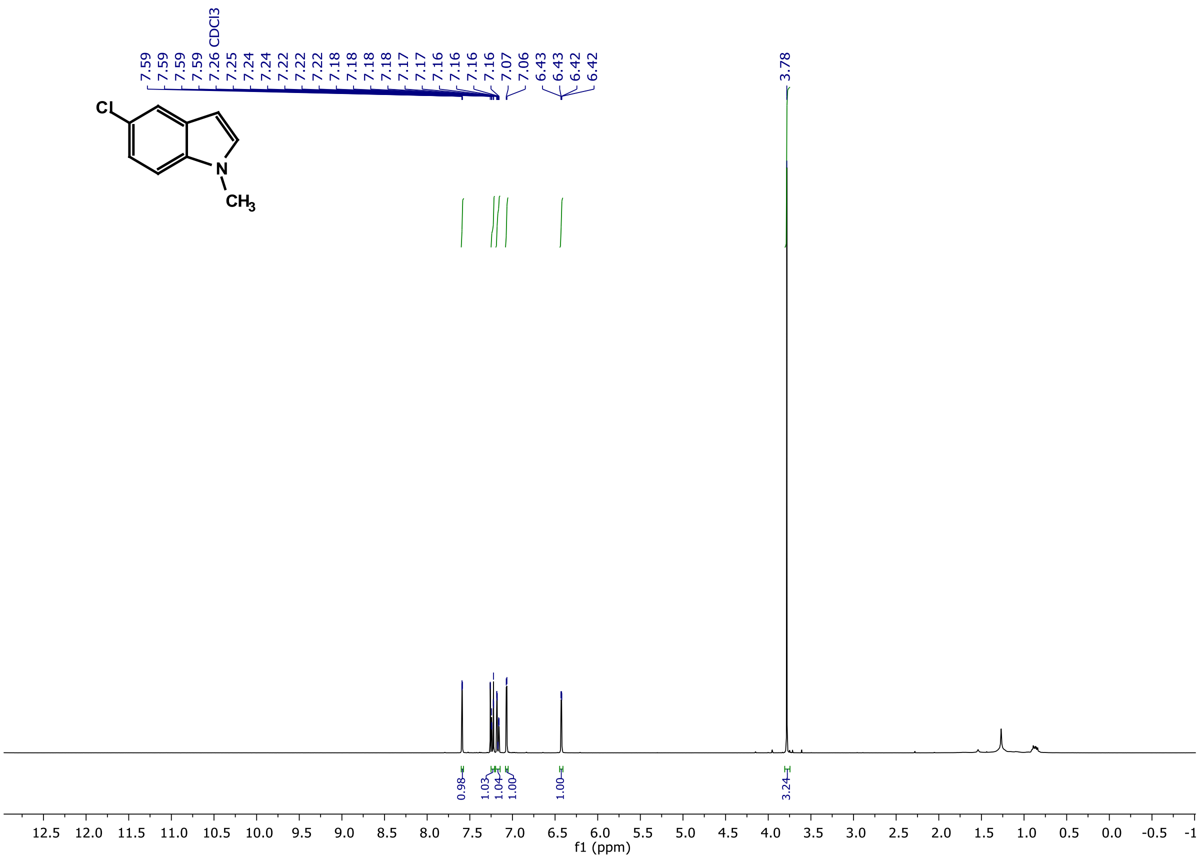

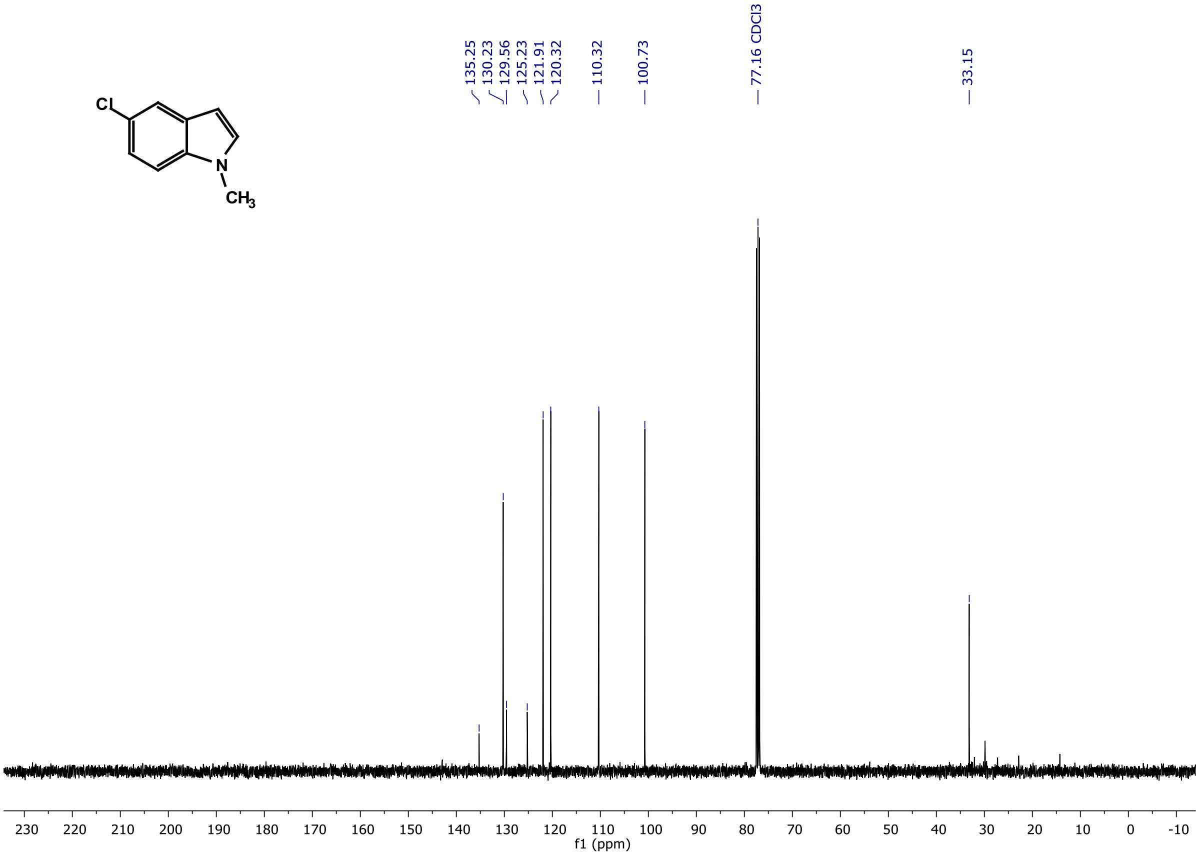

5-Bromo-1-methyl-1*H*-indole (**1r**)

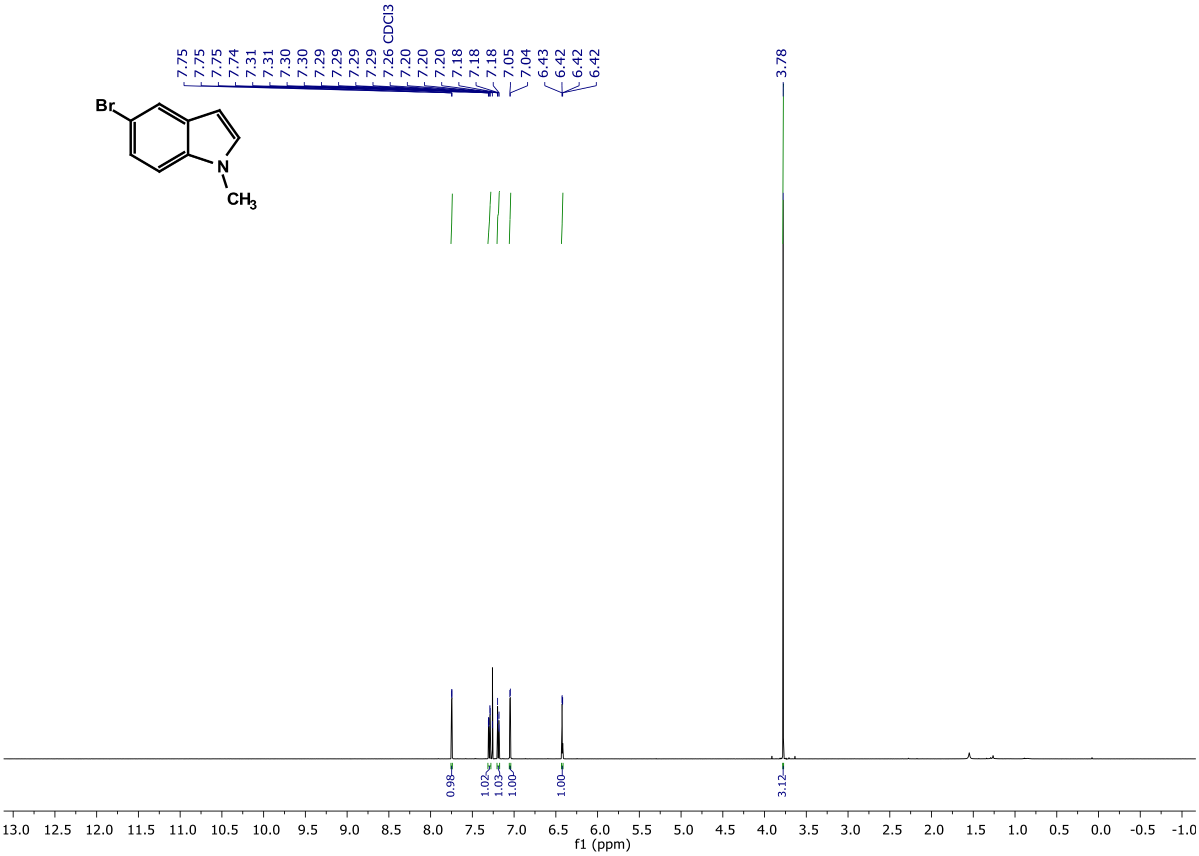

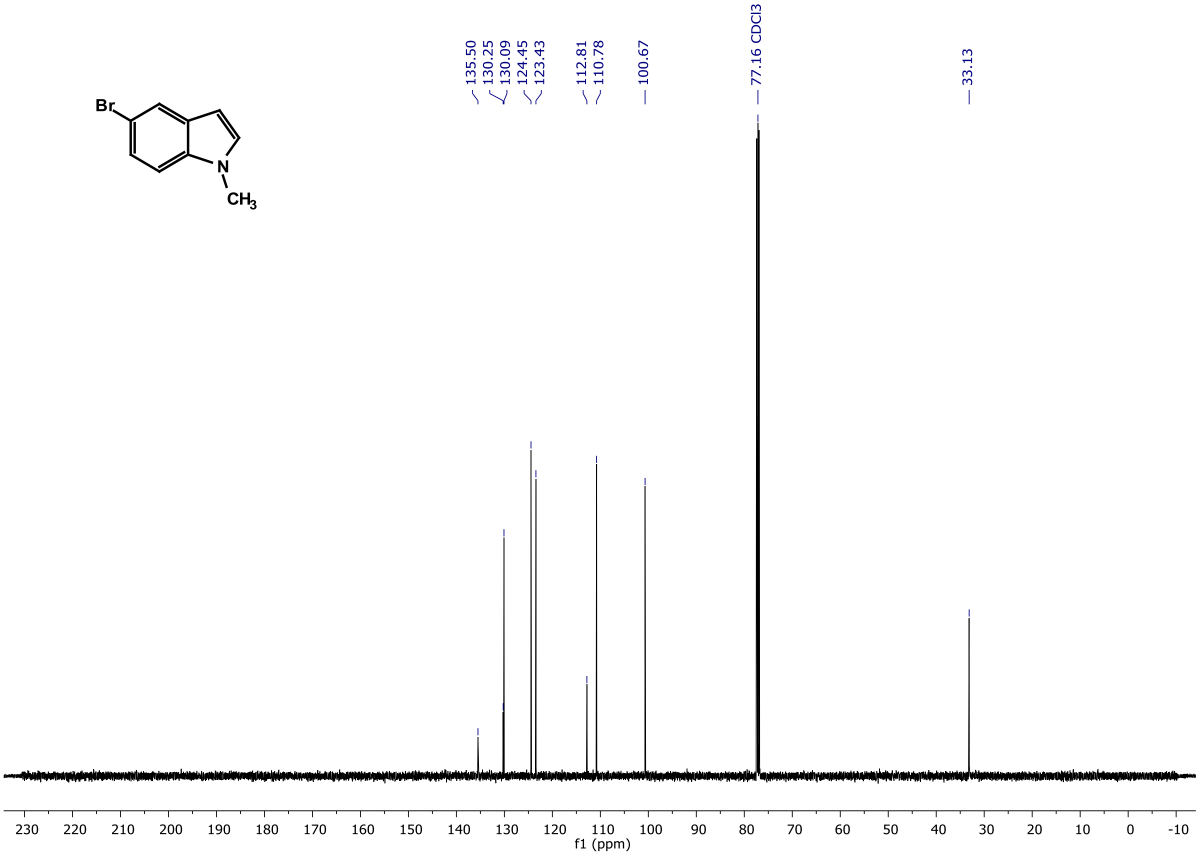

Methyl 1-methyl-1*H*-indole-5-carboxylate (**1s**)

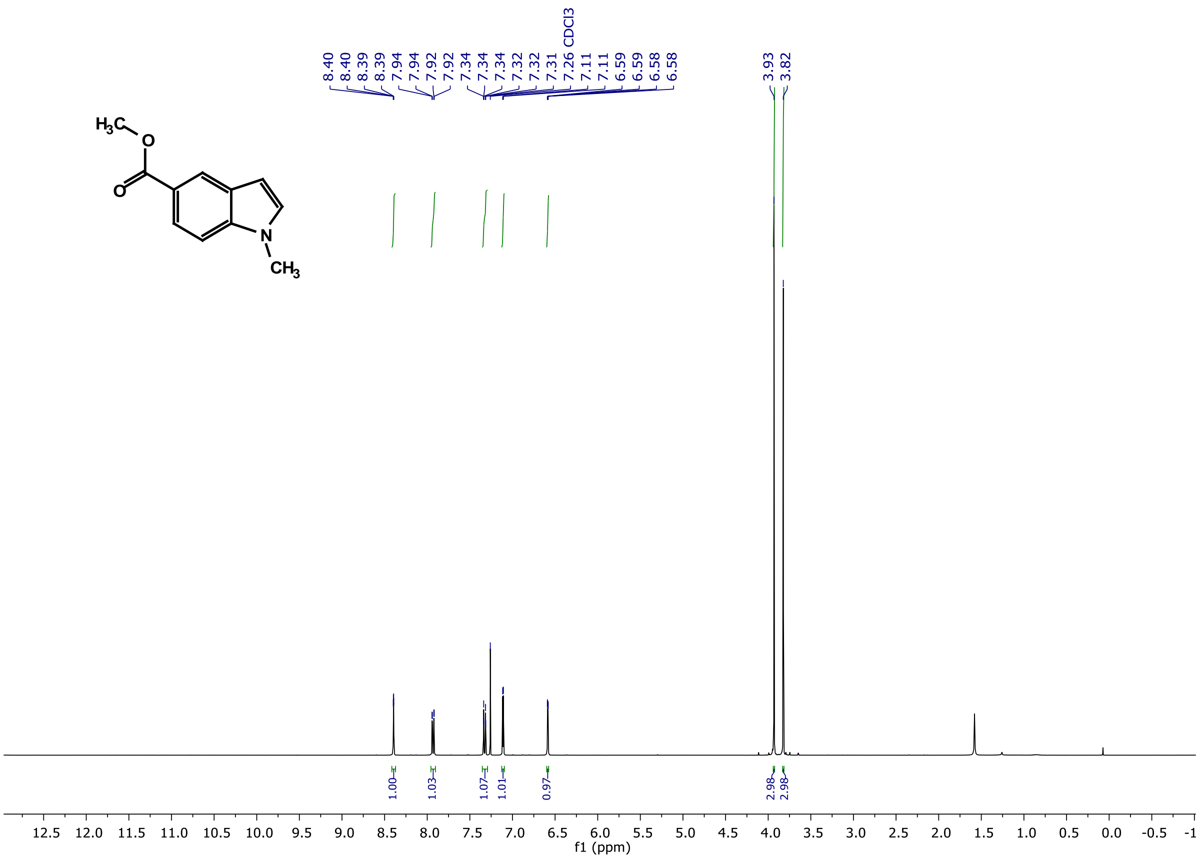

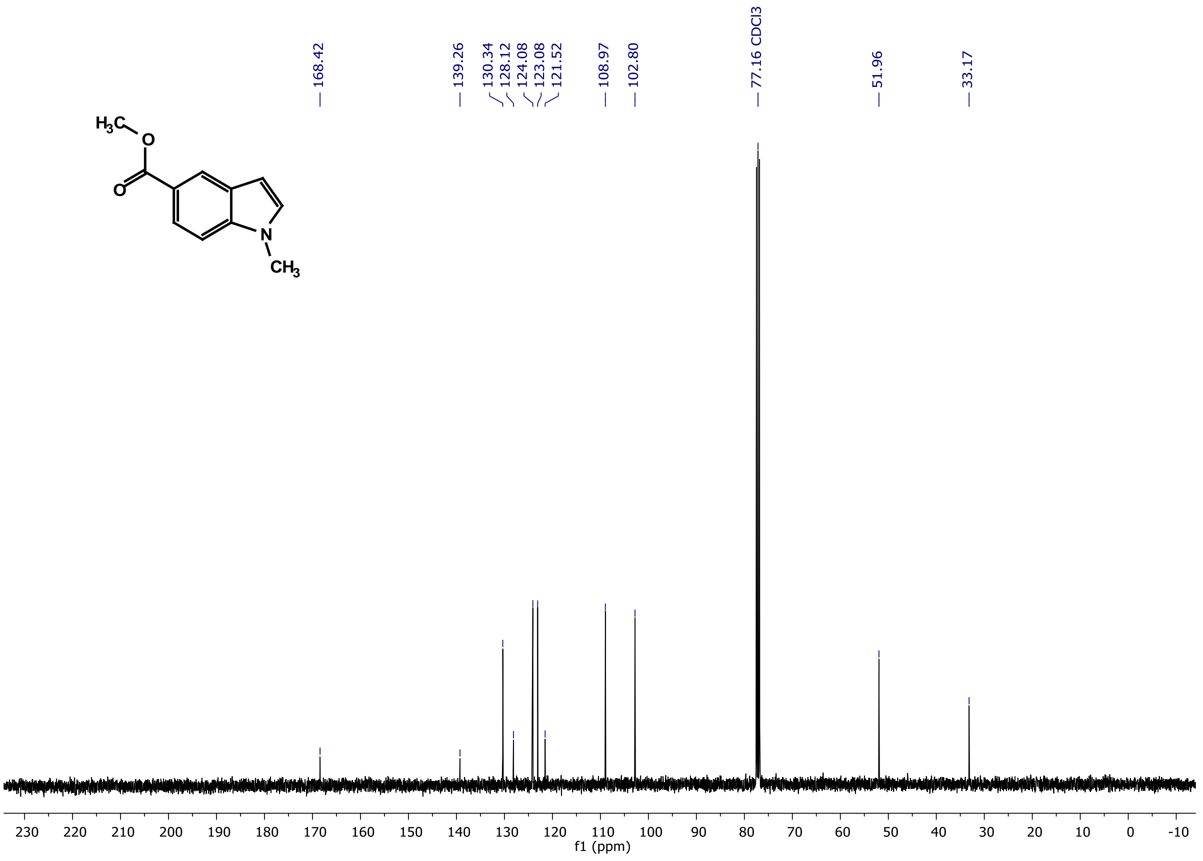

5-Methoxy-1-methyl-1*H*-indole (**1t**)

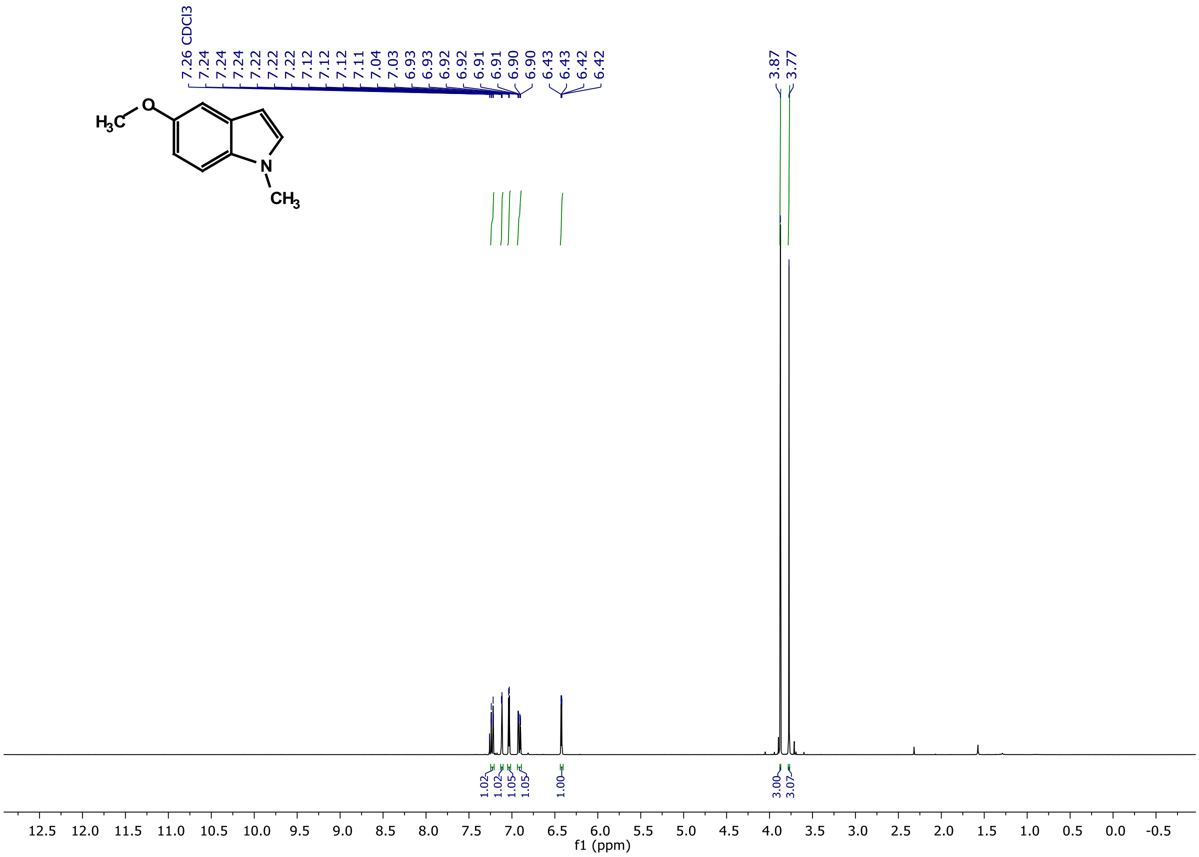

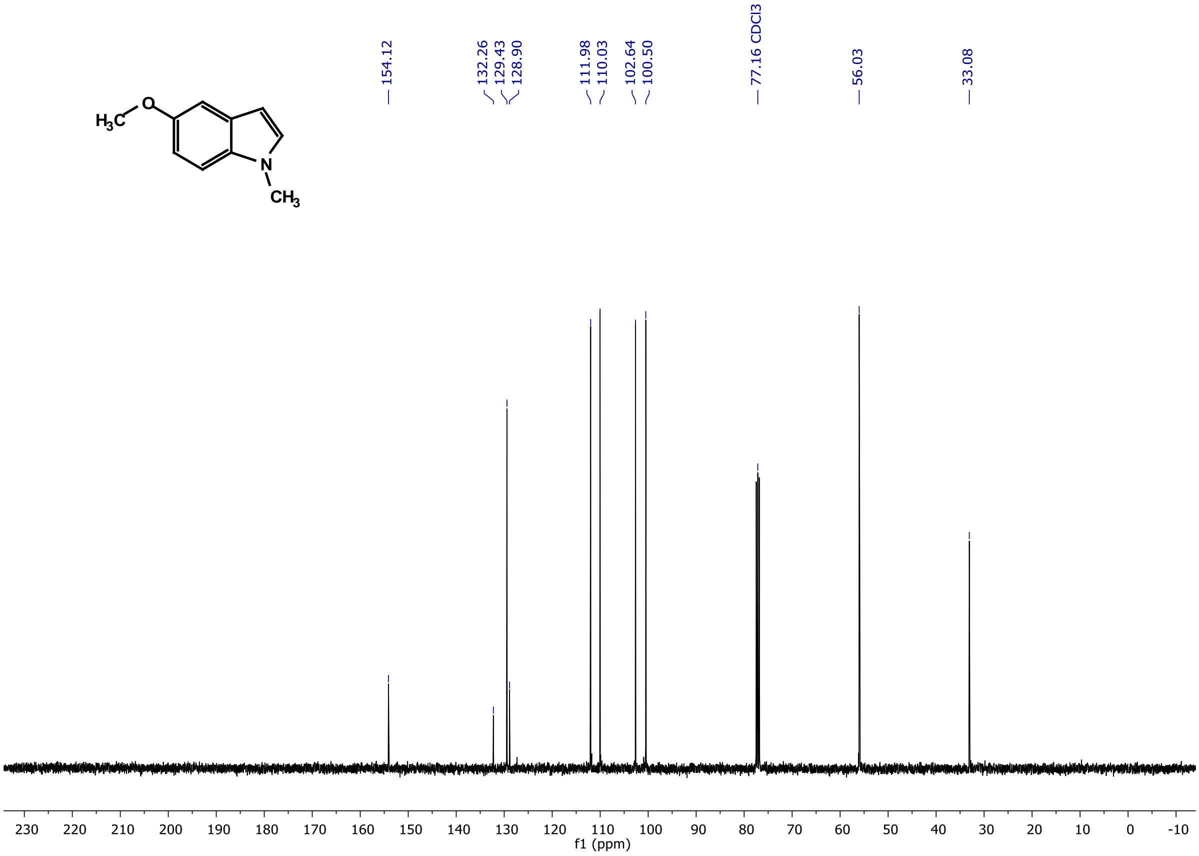

N-(1-methyl-1*H*-indol-6-yl)acetamide (**1v**)

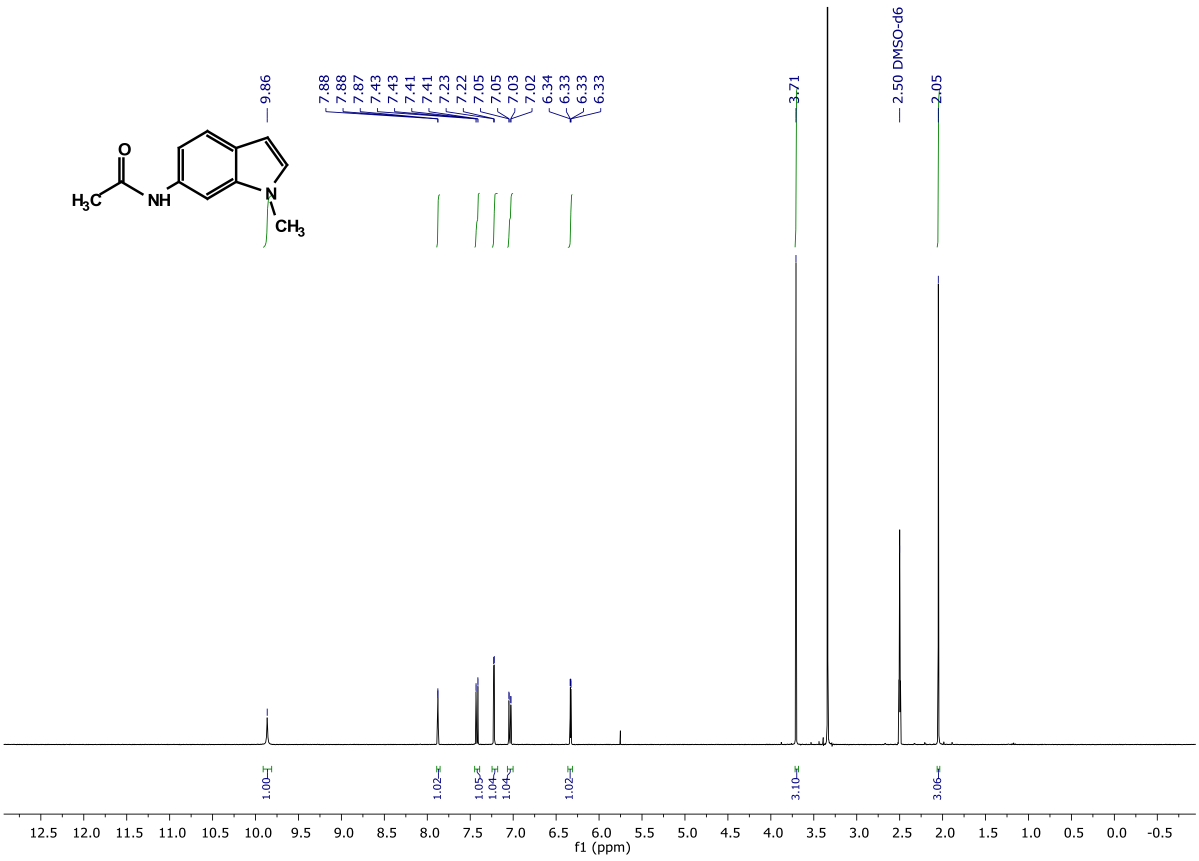

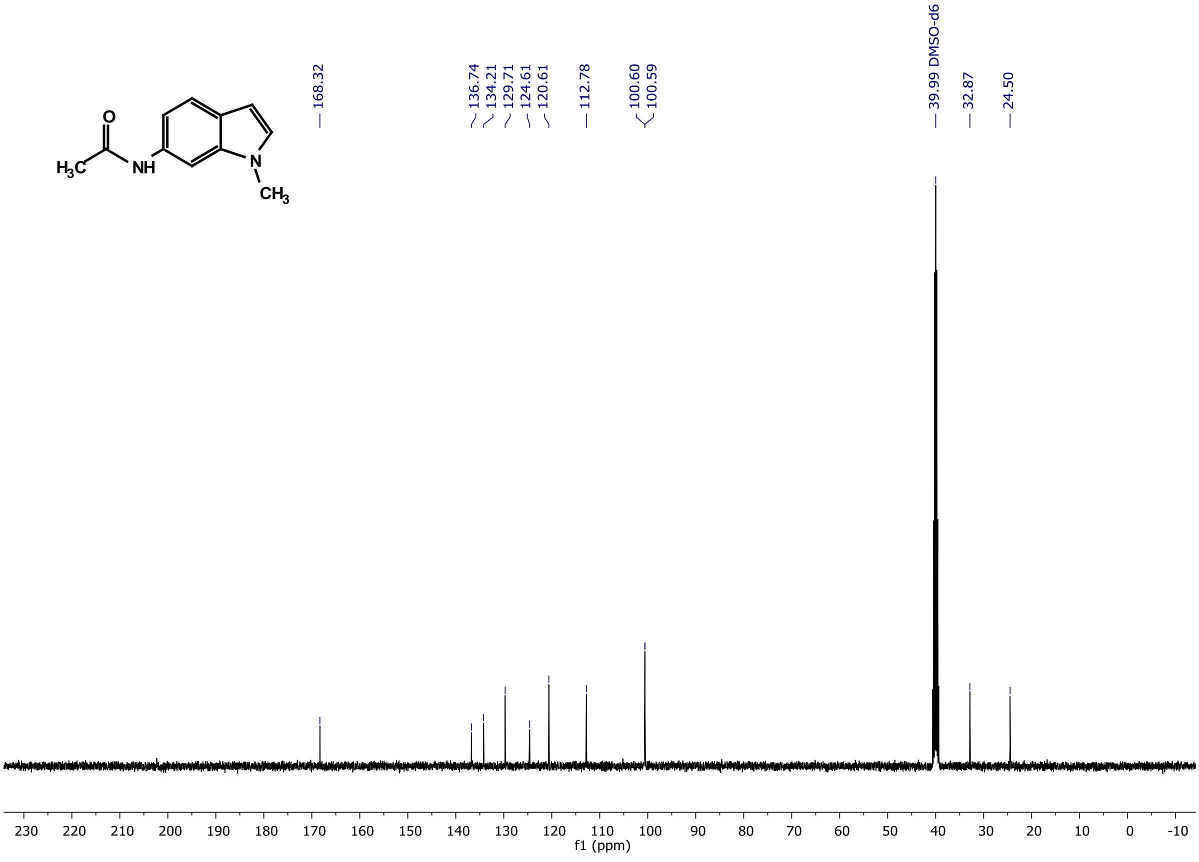

1,4-Dimethyl-1*H*-indole (**1z**)

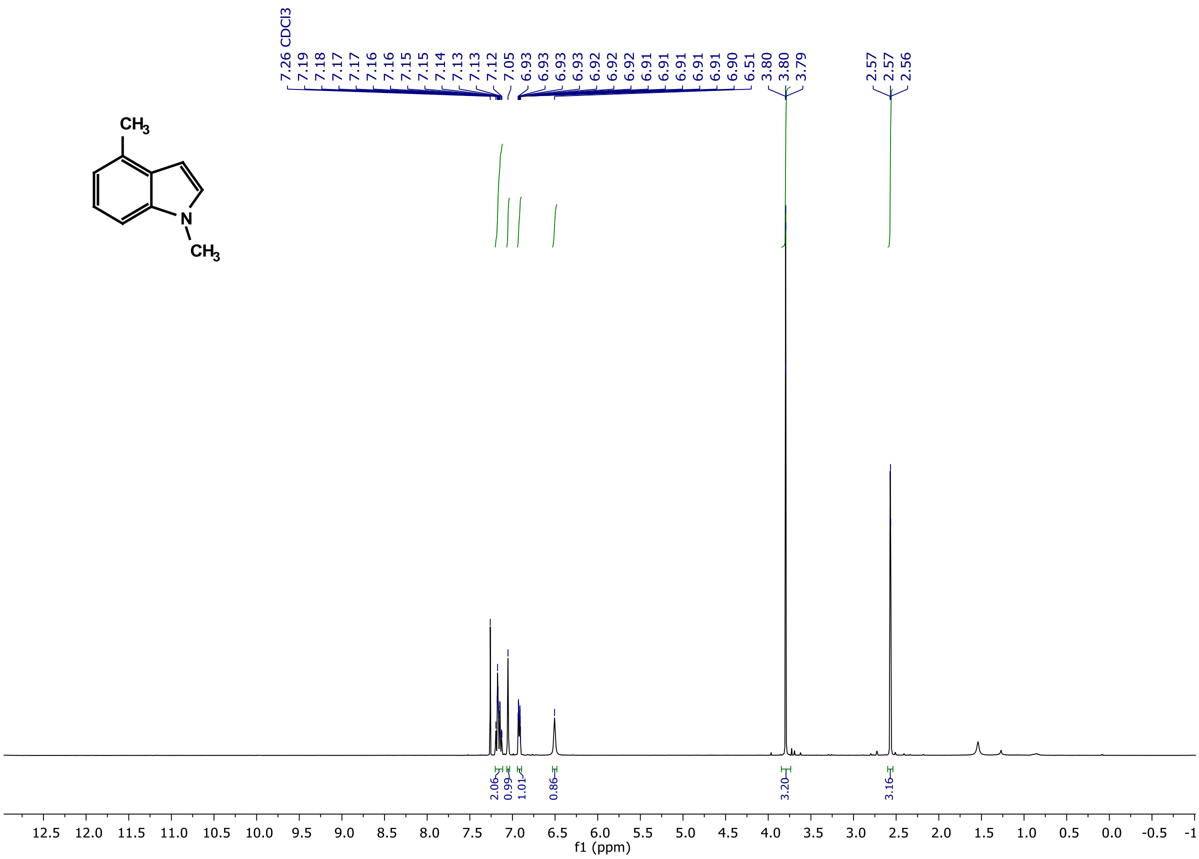

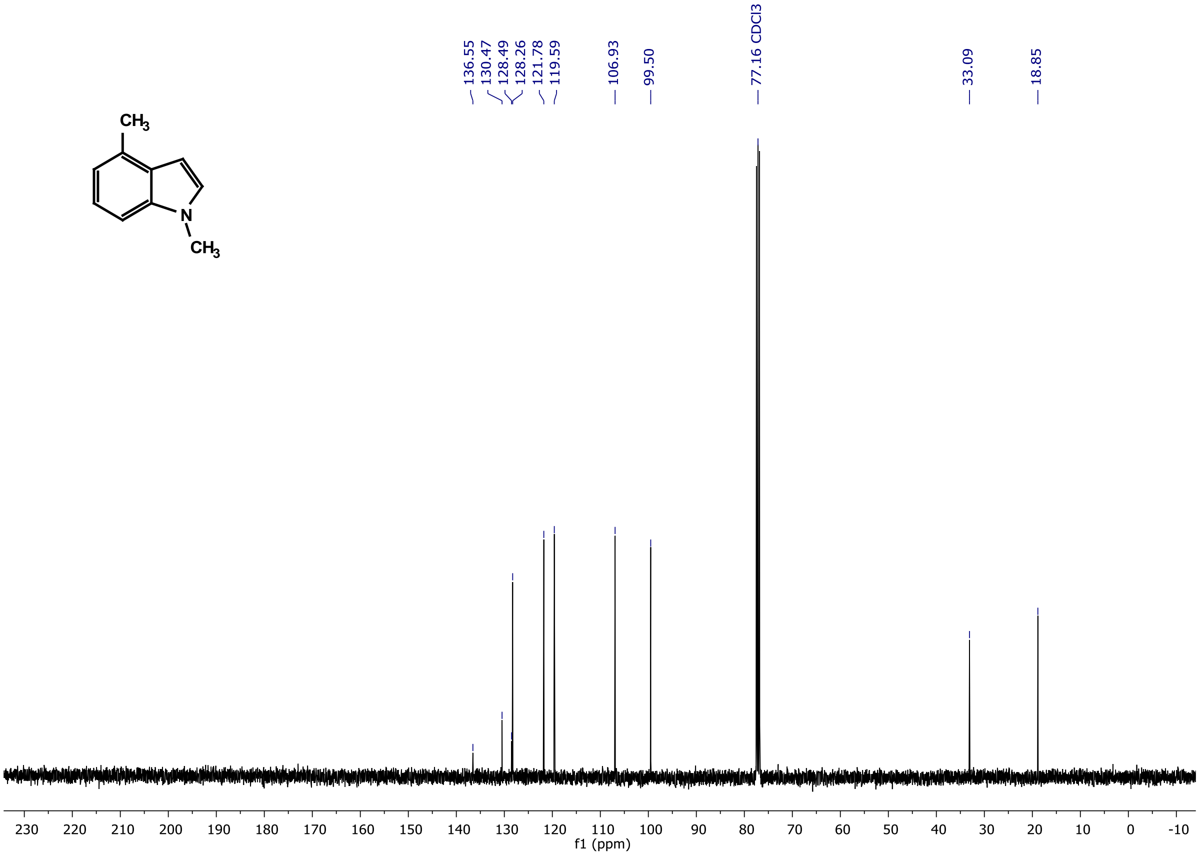

4-Methoxy-1-methyl-1*H*-indole (**1aa**)

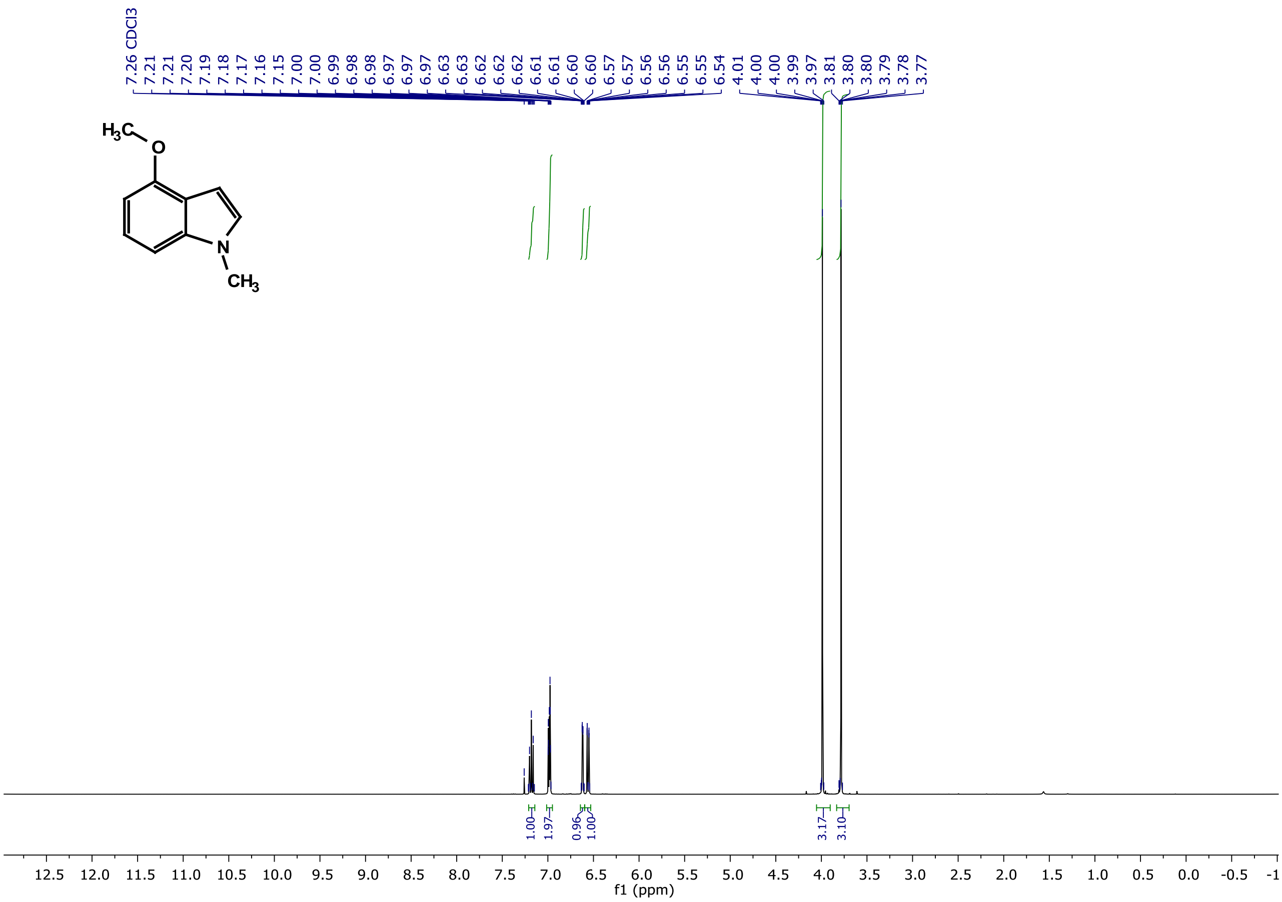

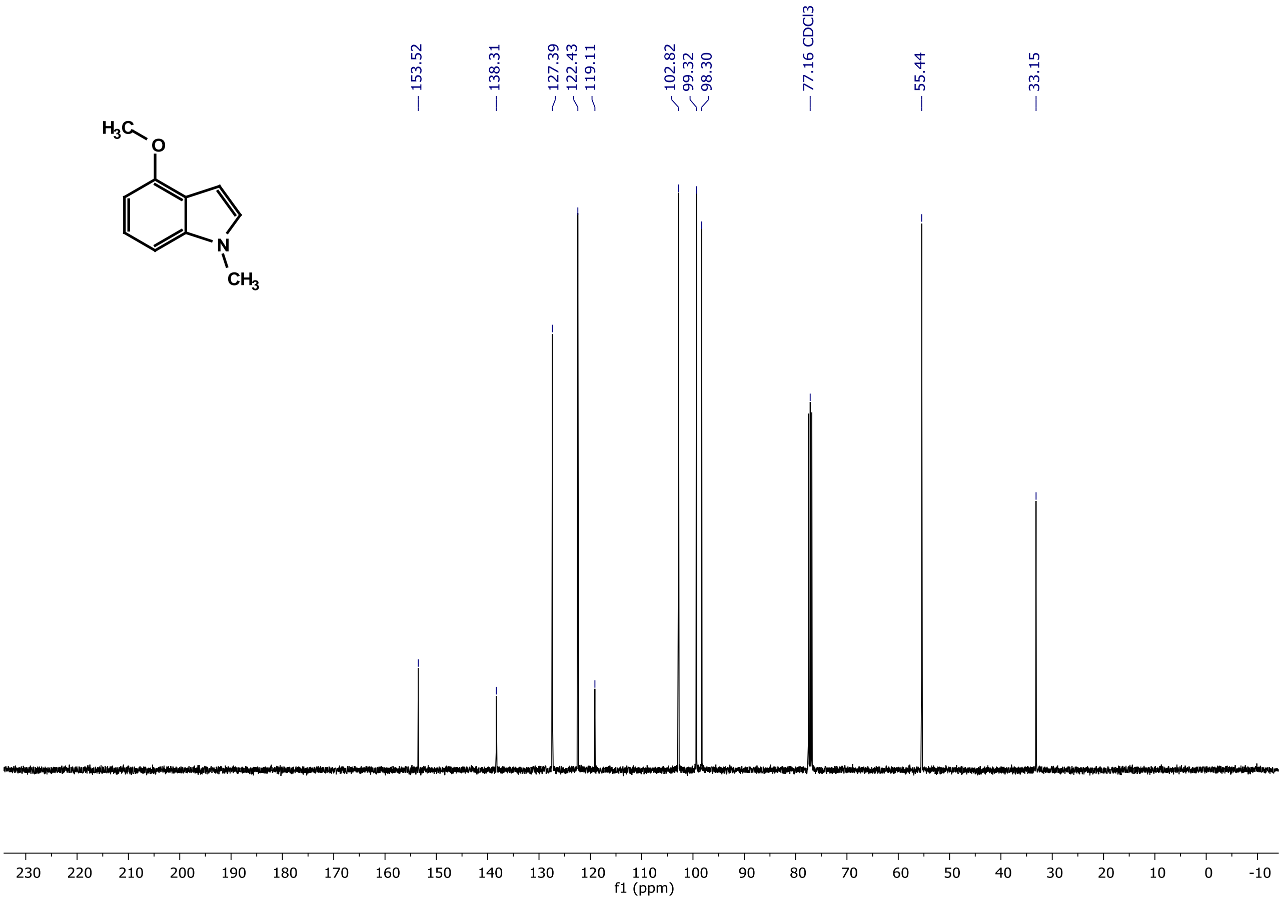

7-Methoxy-1-methyl-1*H*-indole (**1ab**)

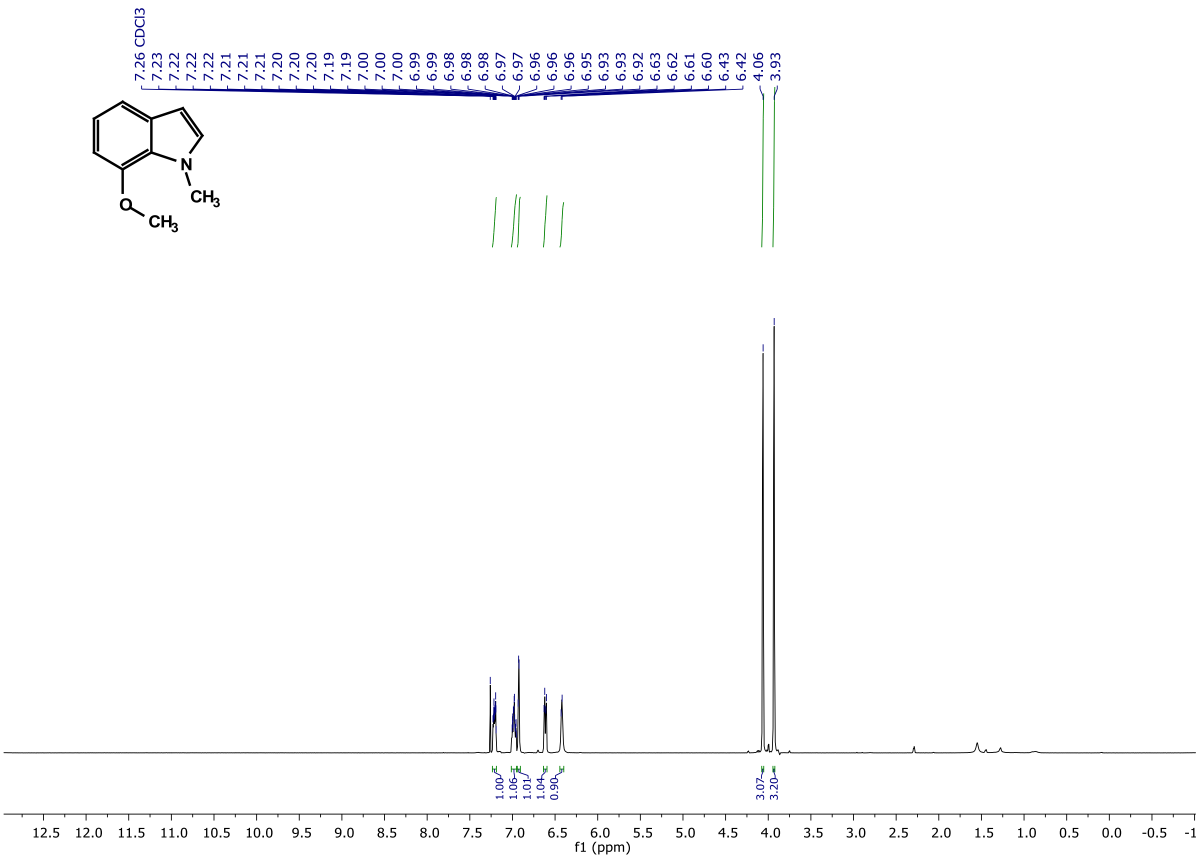

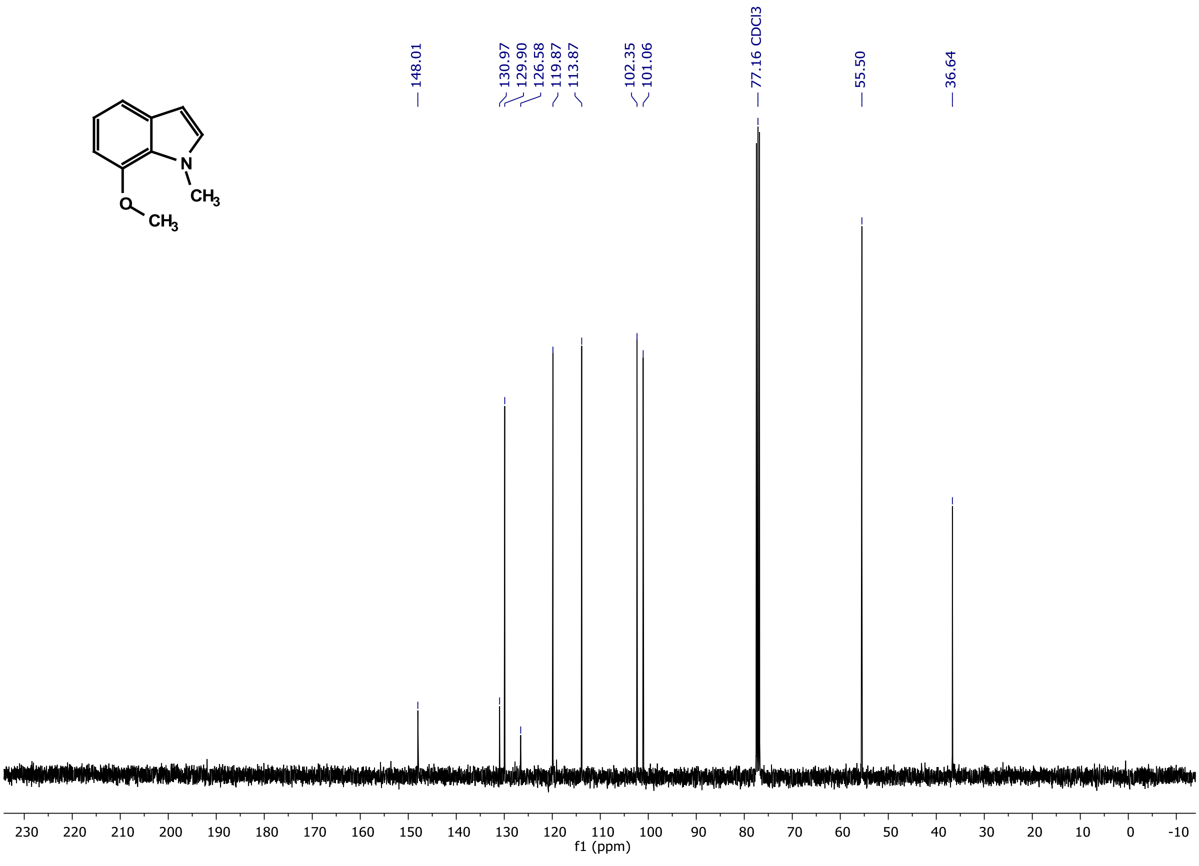

1,7-Dimethyl-1*H*-indole (**1ac**)

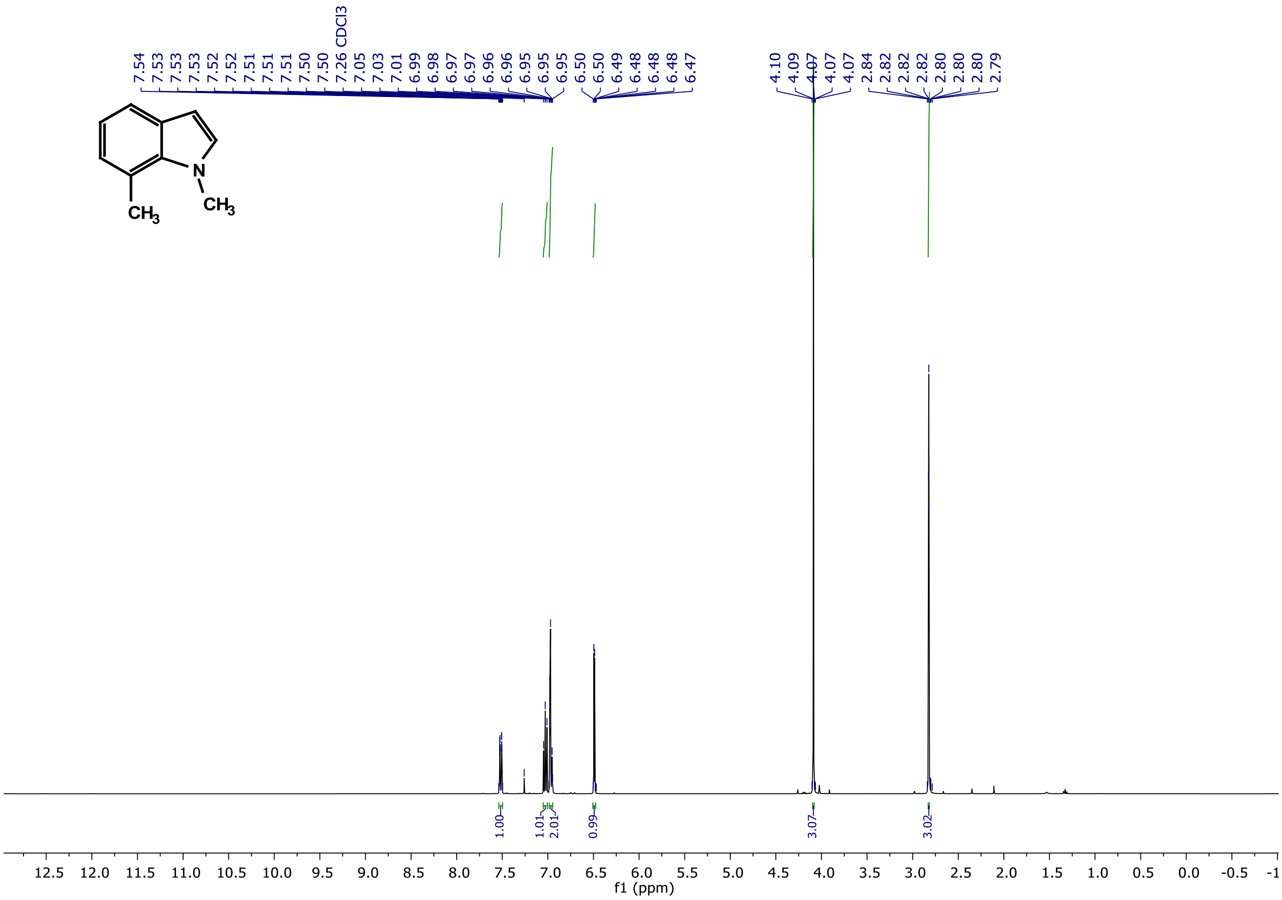

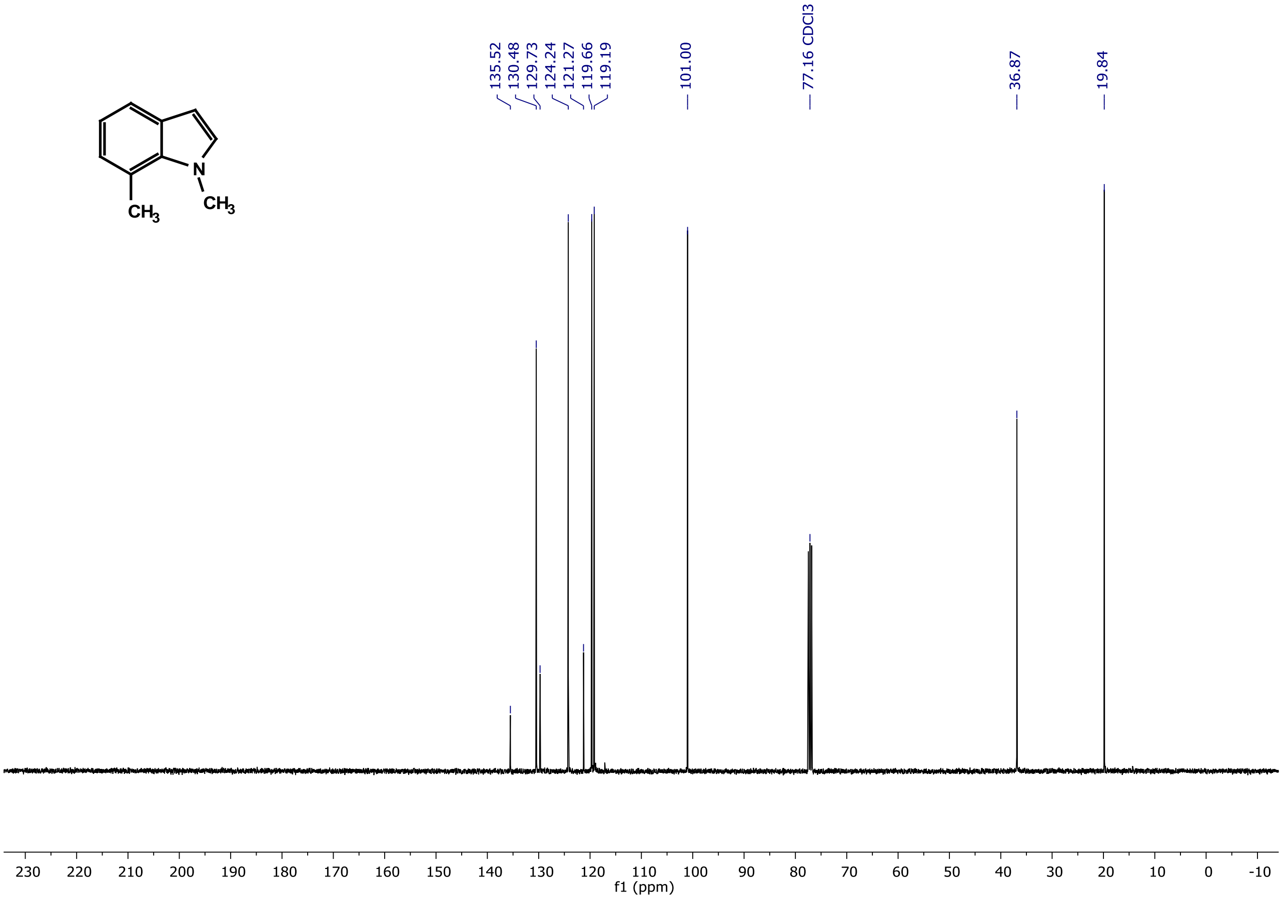

6-Methoxy-1-methyl-1*H*-pyrrolo[3,2-*c*]pyridine (**1ad**)

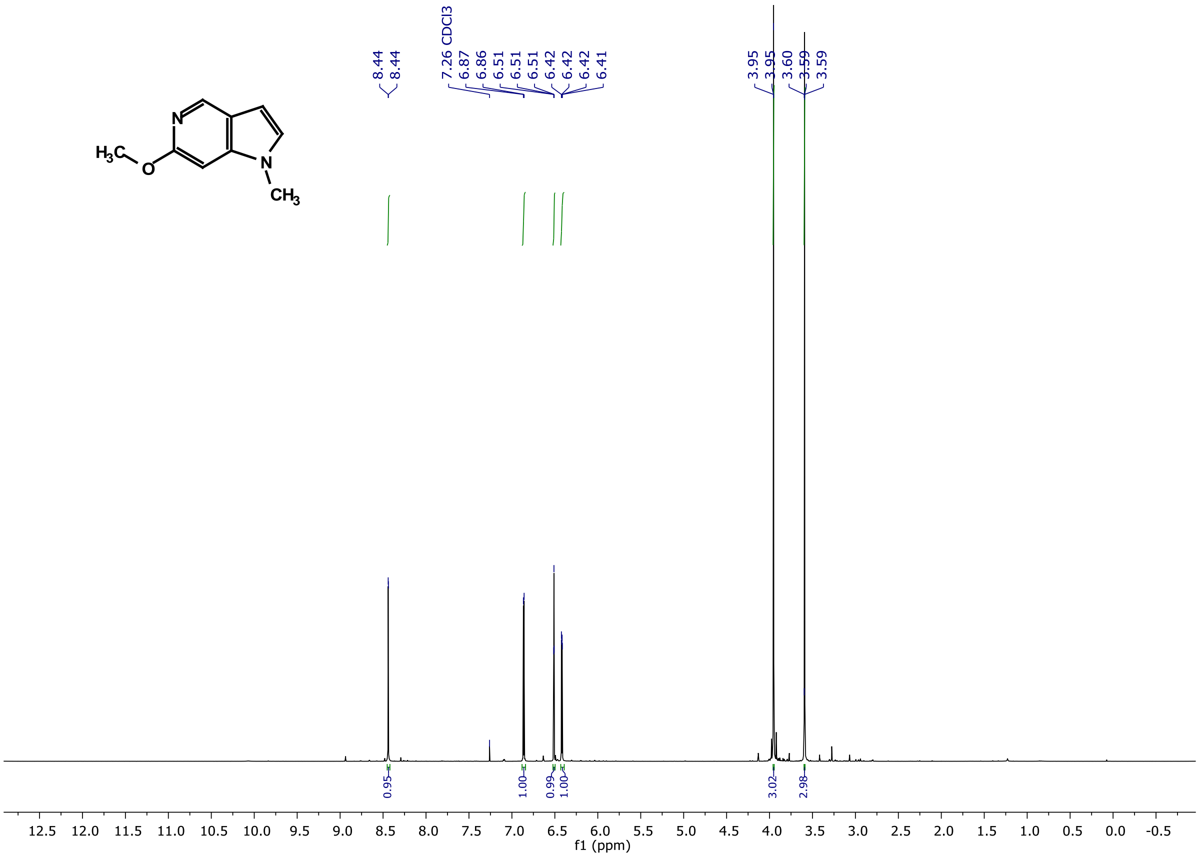

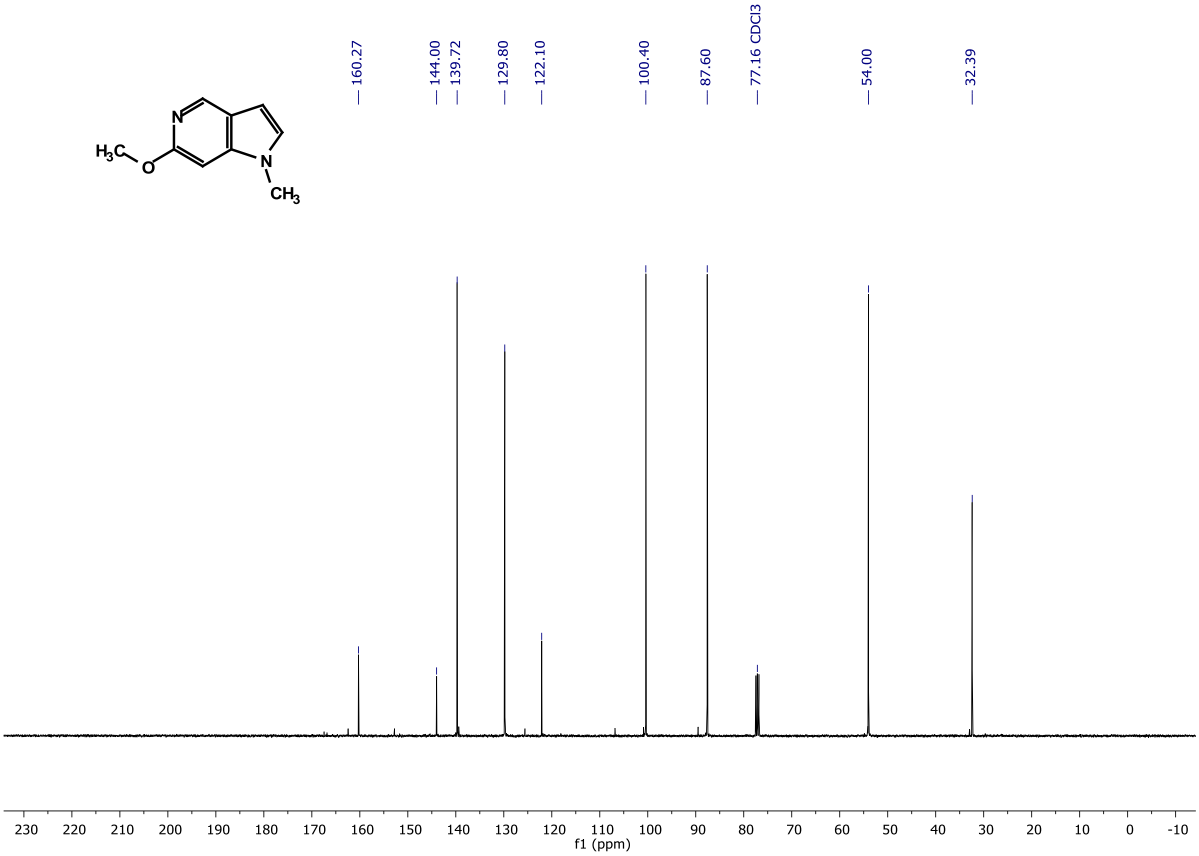

6-Methoxy-1-methyl-1*H*-pyrrolo[2,3-*b*]pyridine (**1ae**)

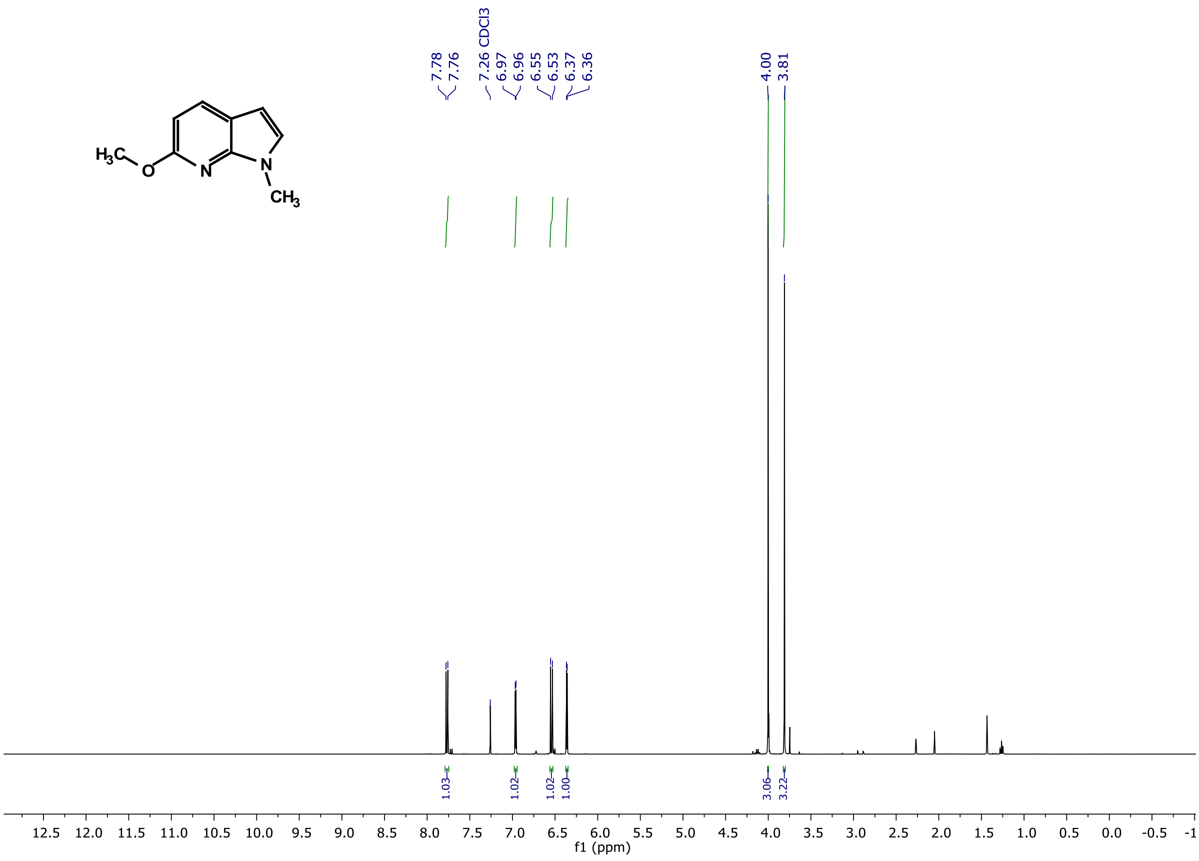

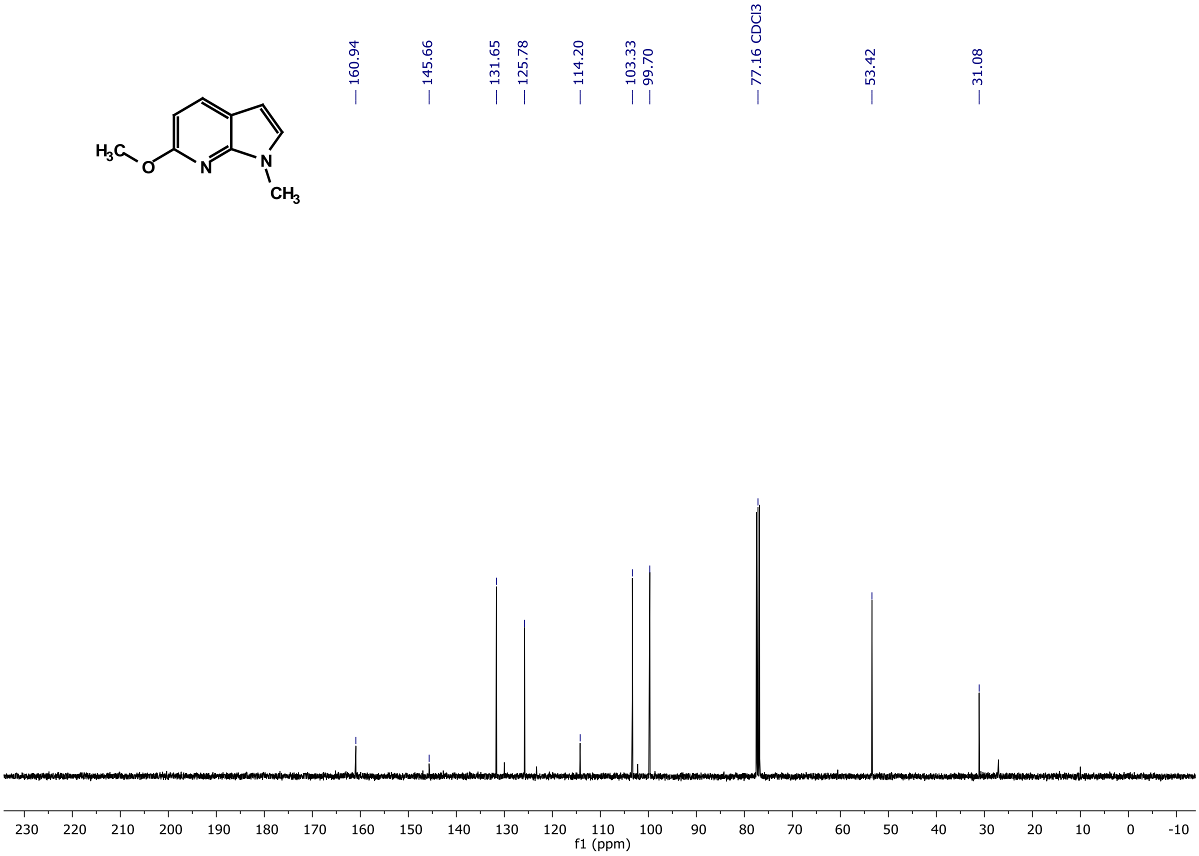

4-Methoxy-1-methyl-1*H*-pyrrolo[3,2-*c*]pyridine (**1af**)

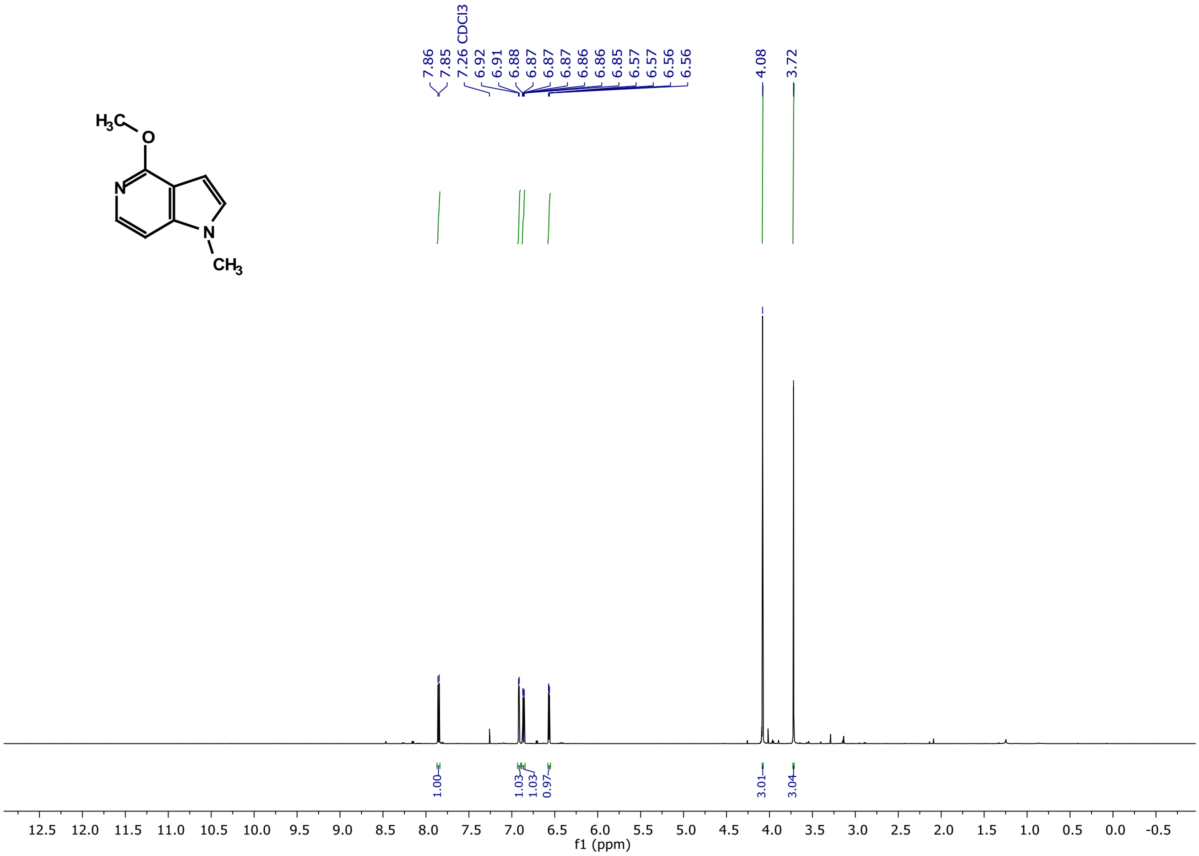

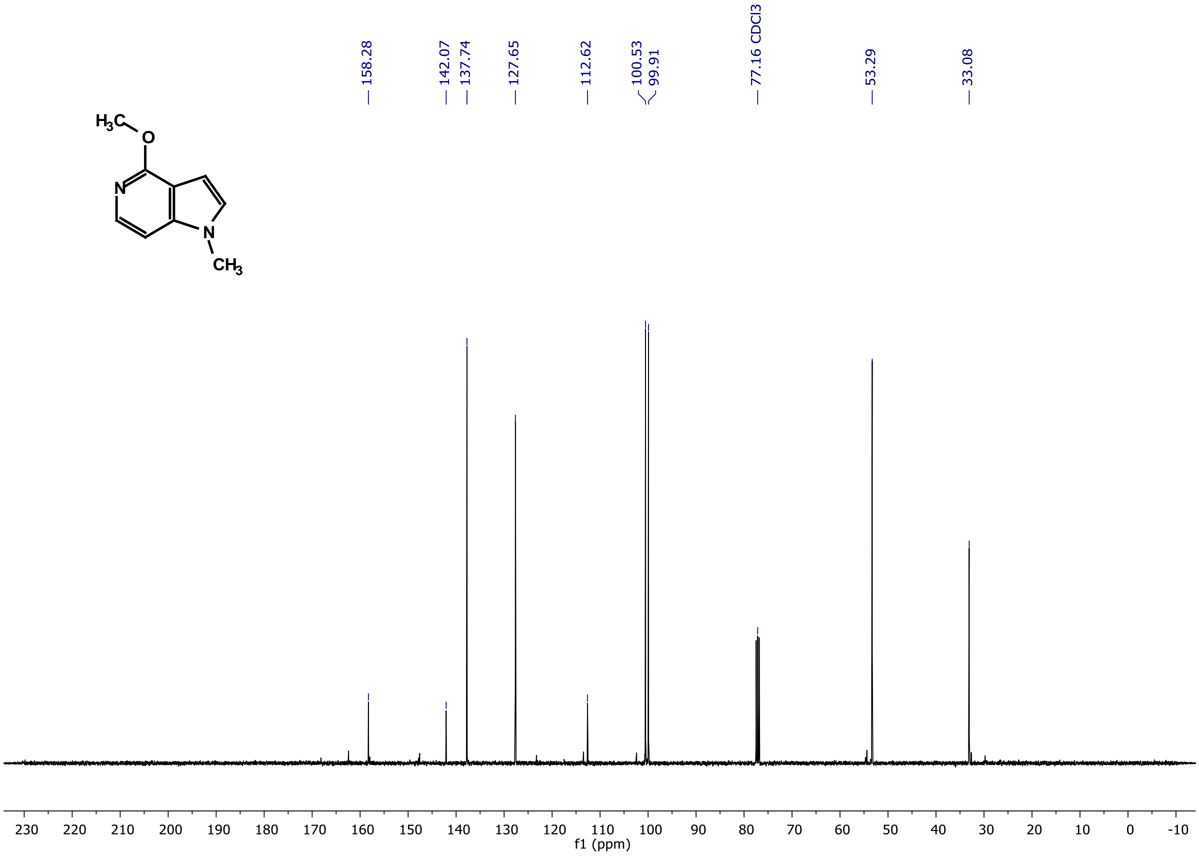

7-Chloro-5-(1-methyl-1*H*-indol-6-yl)quinoxaline (**1ag**)

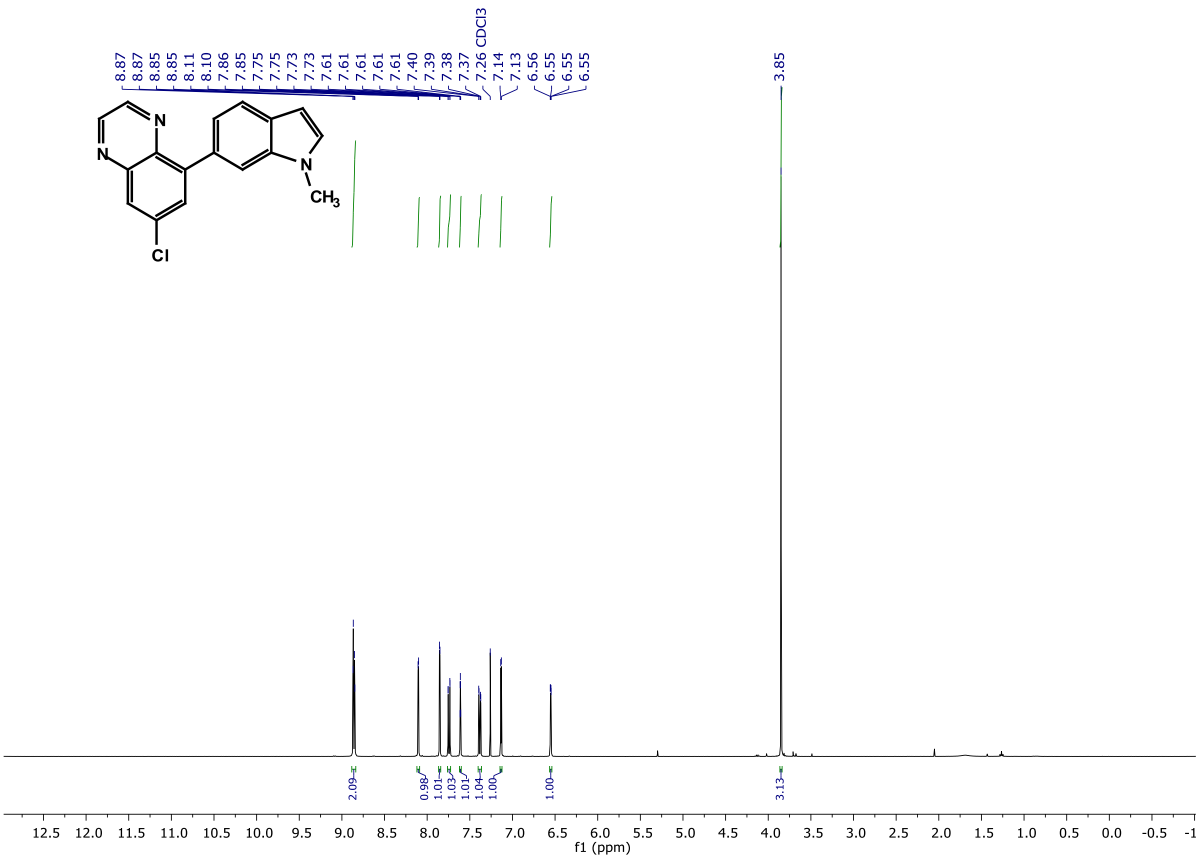

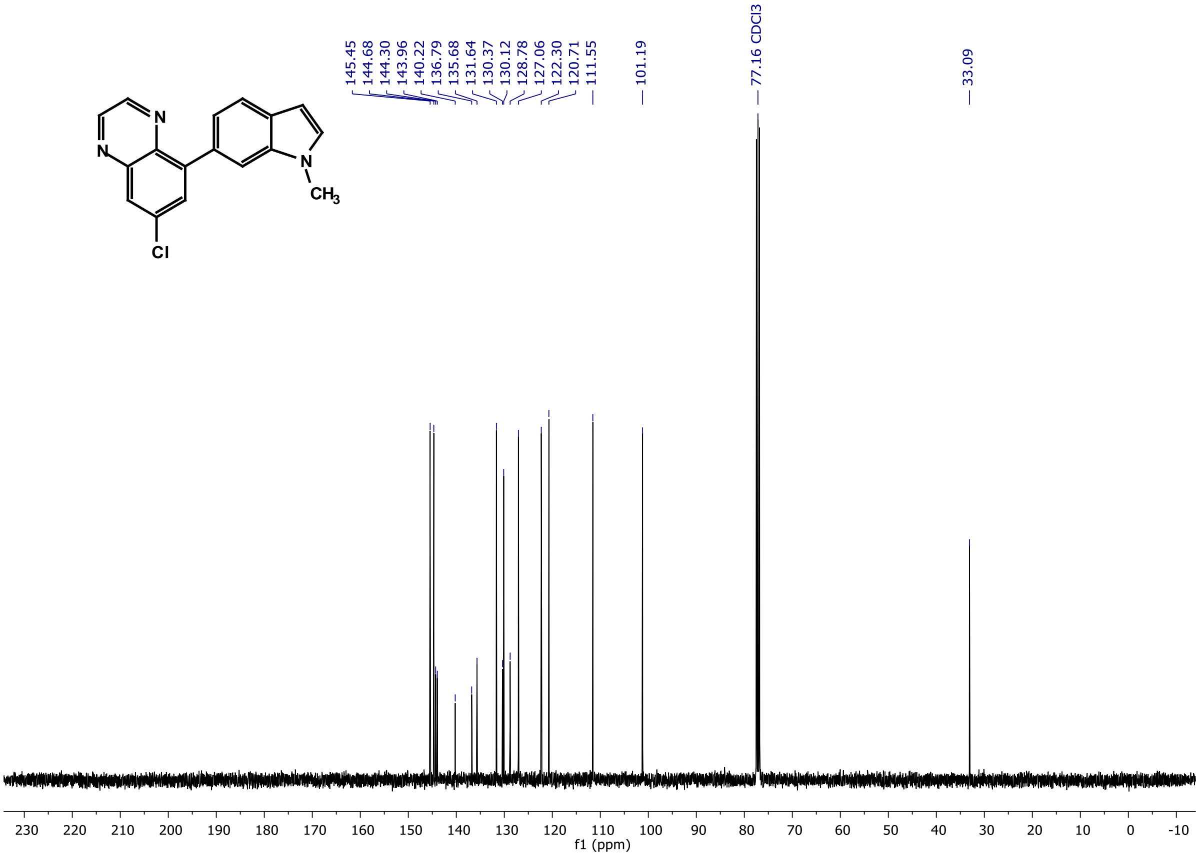

2-(1-Methyl-1*H*-indol-6-yl)isoindolin-1-one (**1ah**)

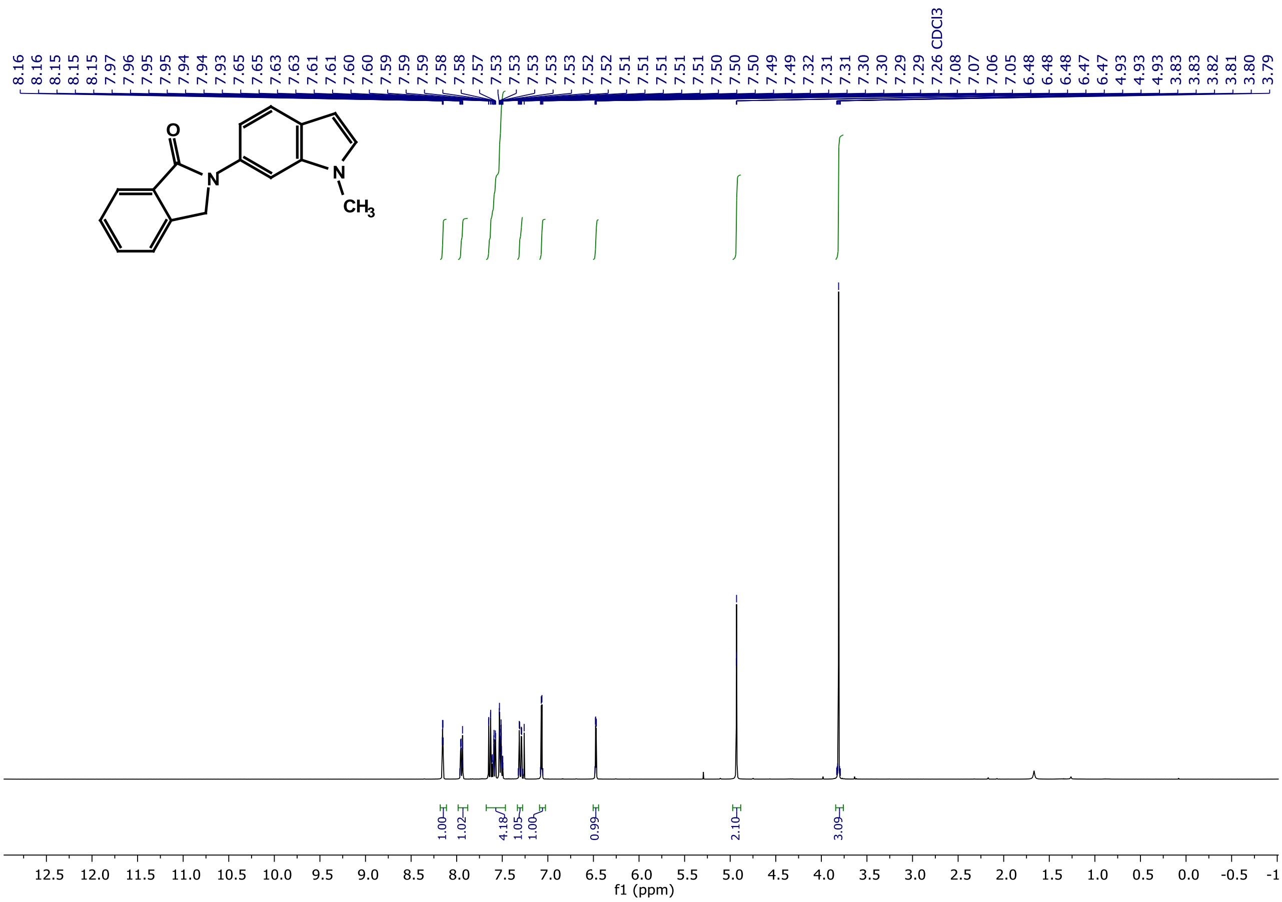

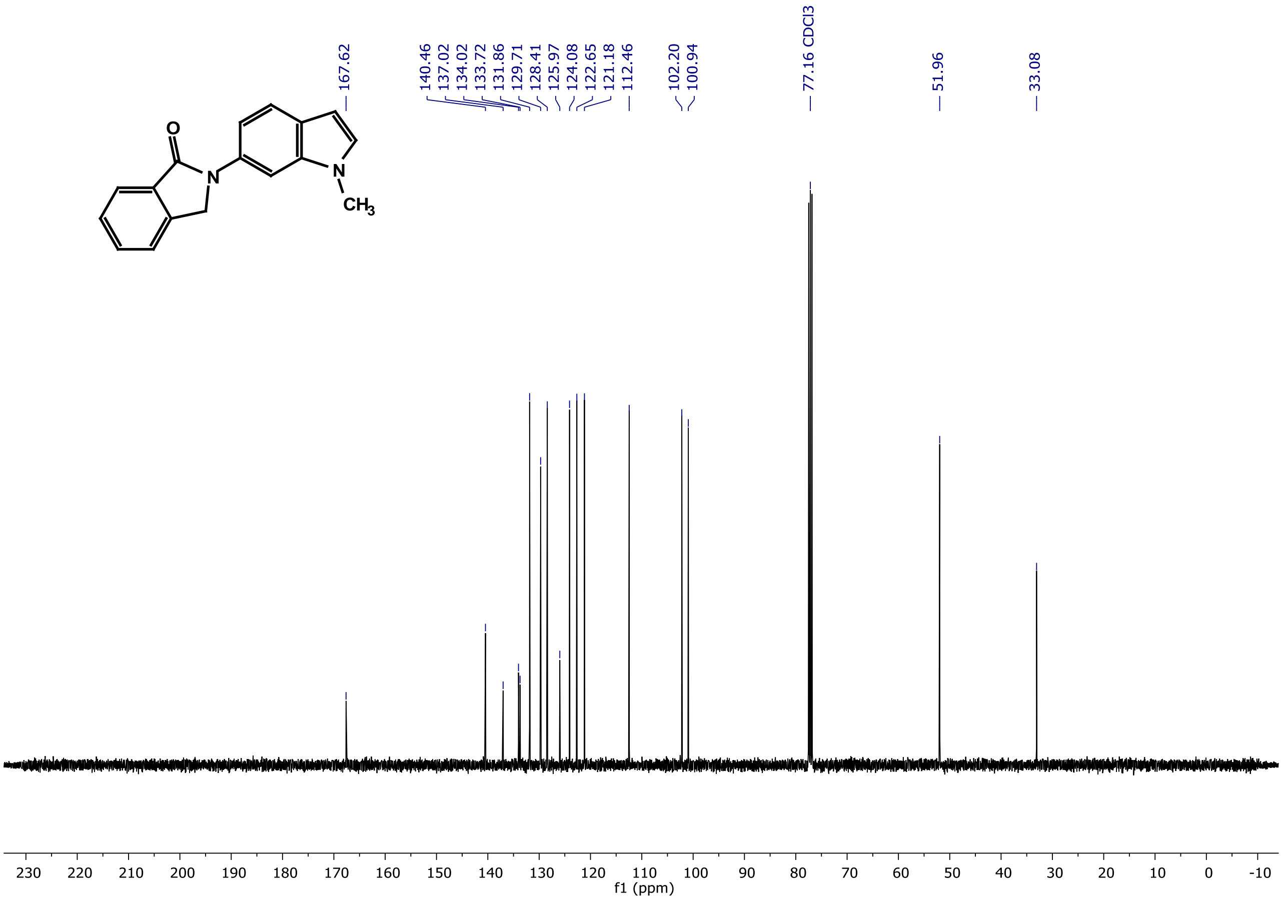

1-(1-Methyl-1*H*-indol-4-yl)-3-(pyridin-2-yl)urea (**1ai**)

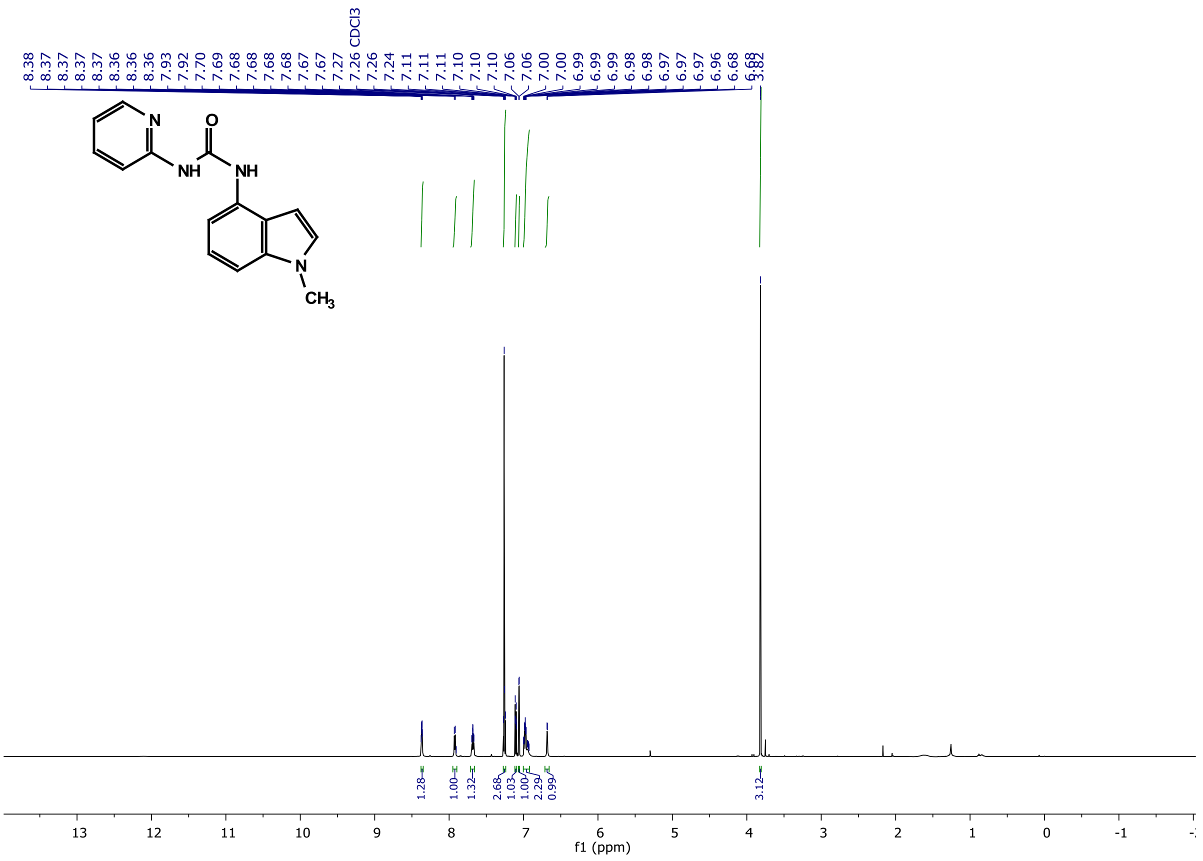

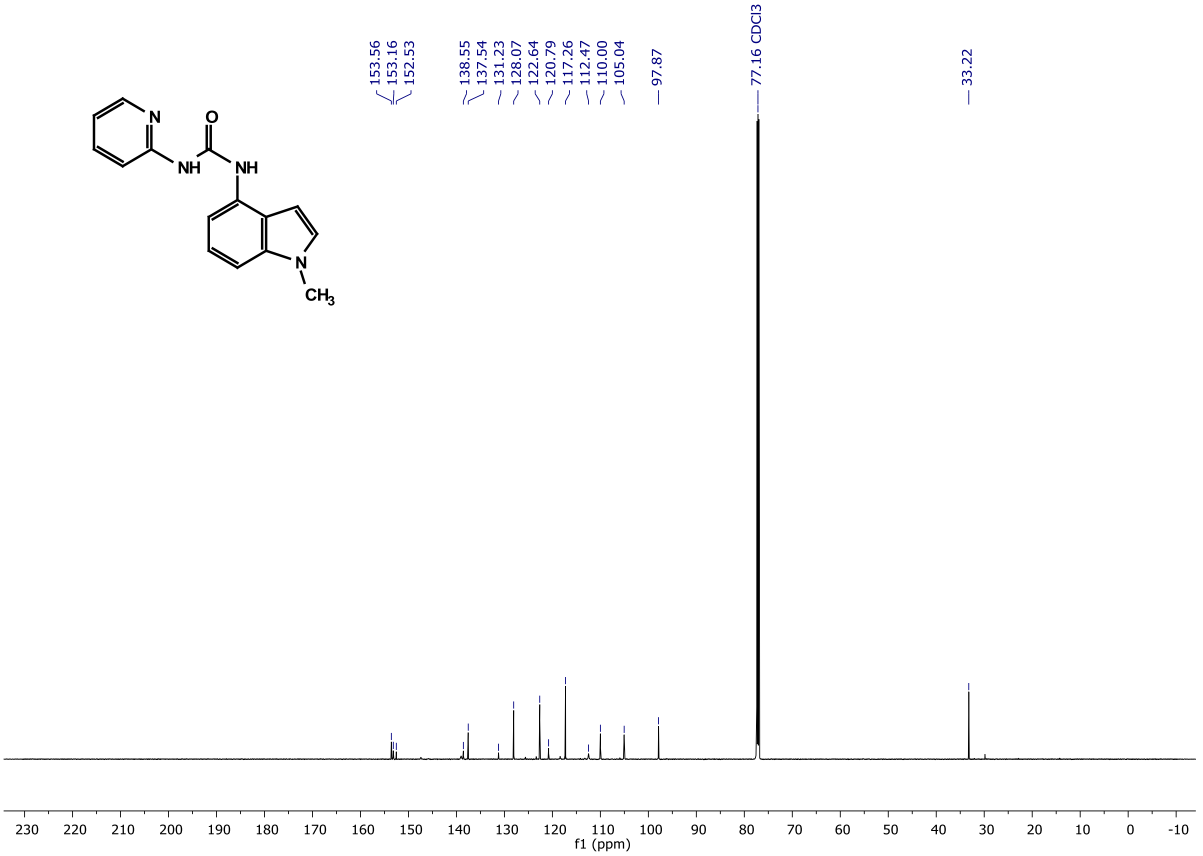

Methyl 4-((diethylcarbamoyl)oxy)benzoate (**SI-1**)

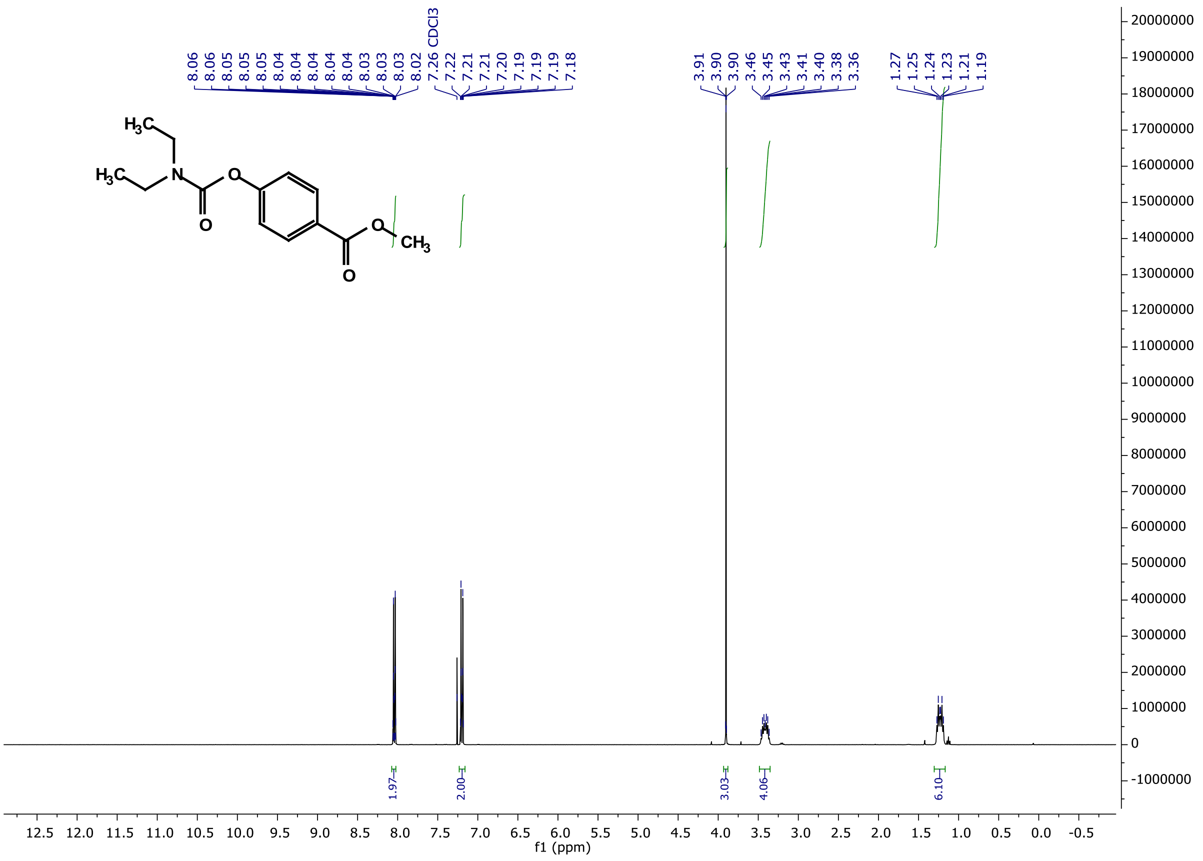

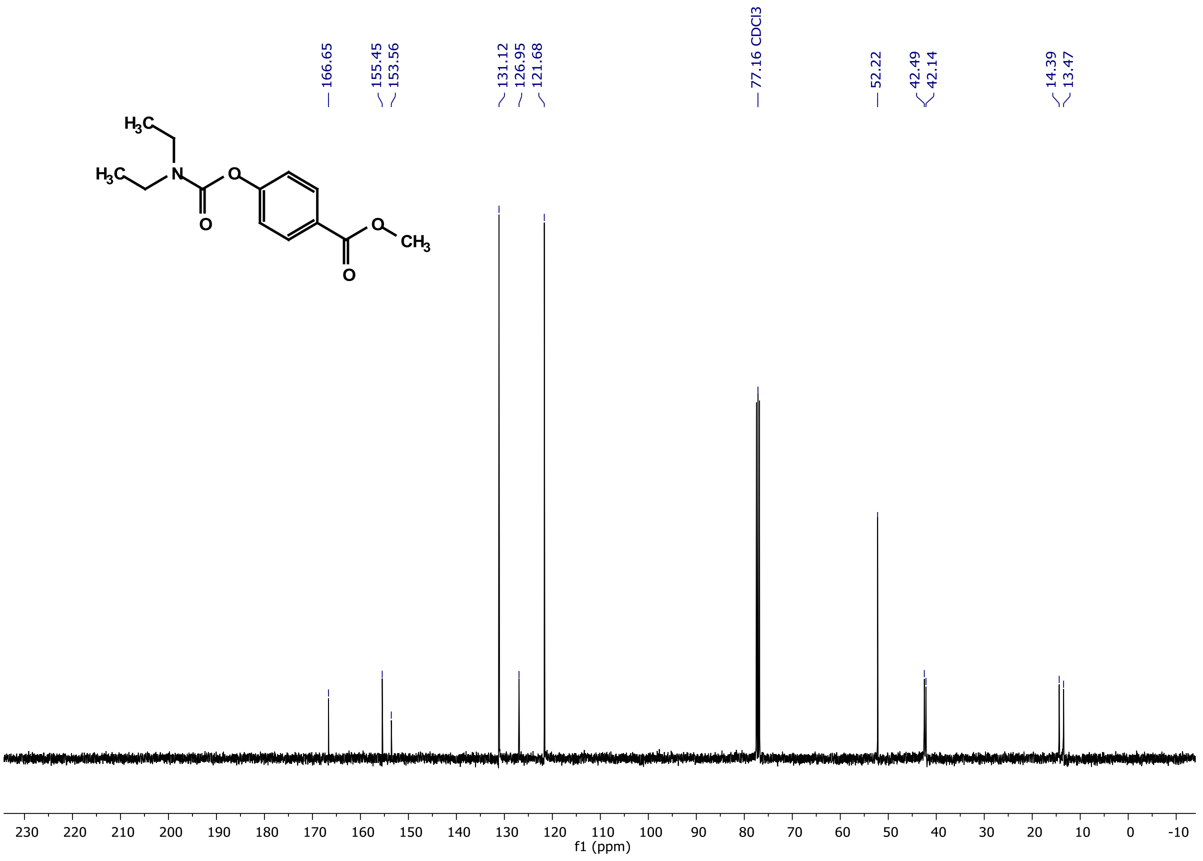

4-((Diethylcarbamoyl)oxy)benzoic acid (**SI-2**)

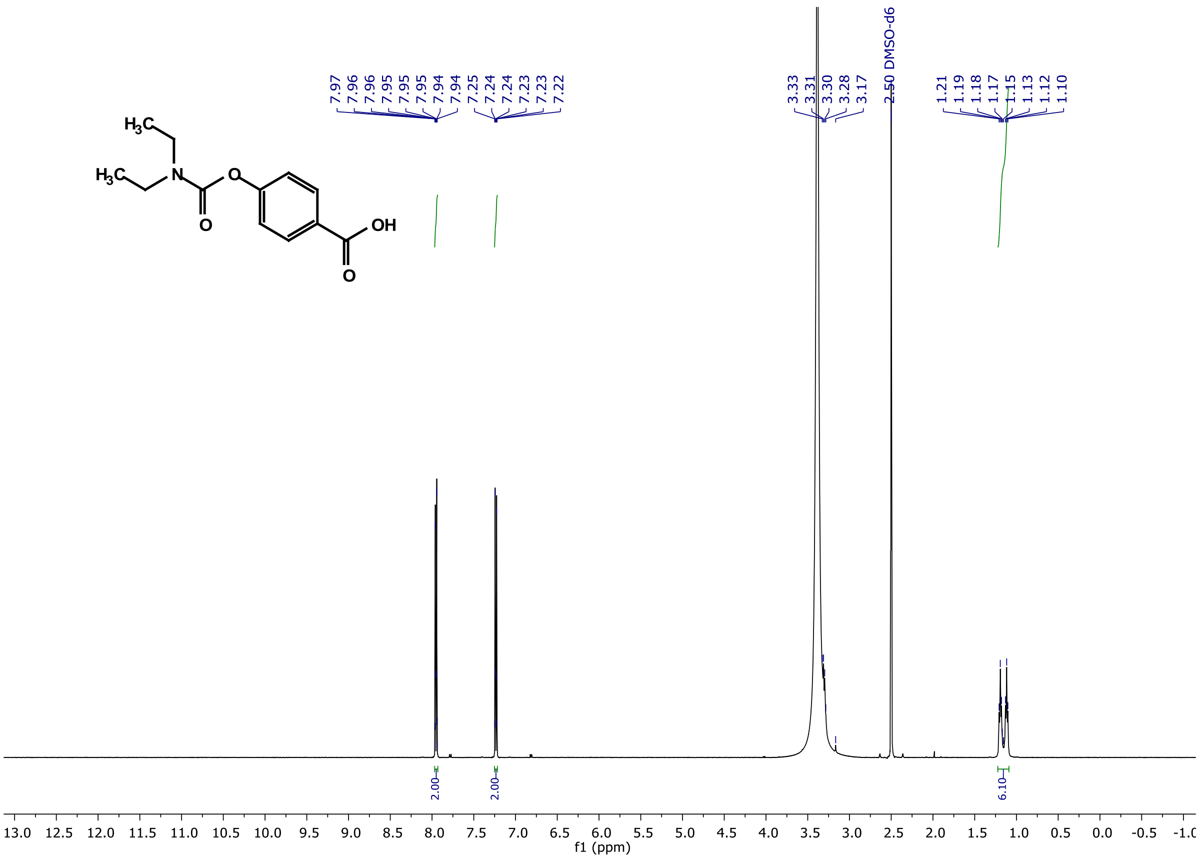

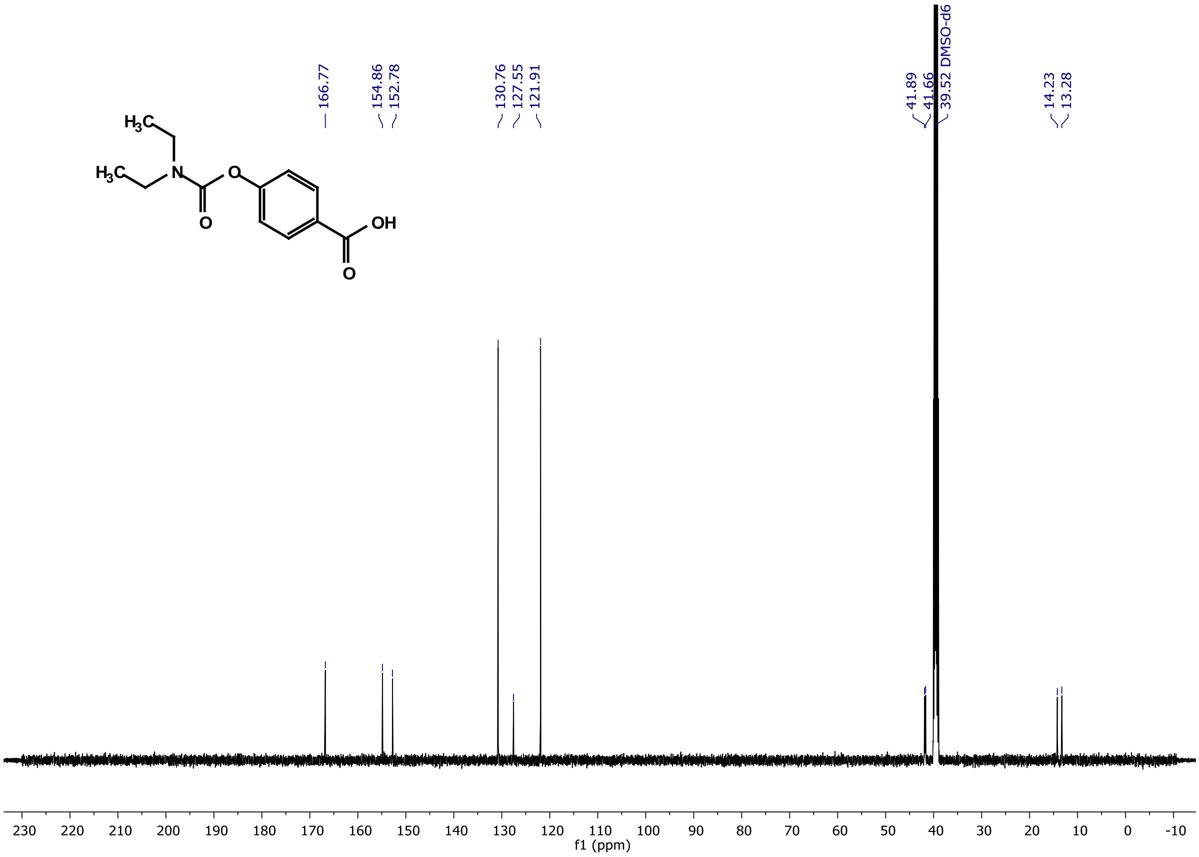

4-((1*H*-indol-7-yl)carbamoyl)phenyl diethylcarbamate (**SI-3**)

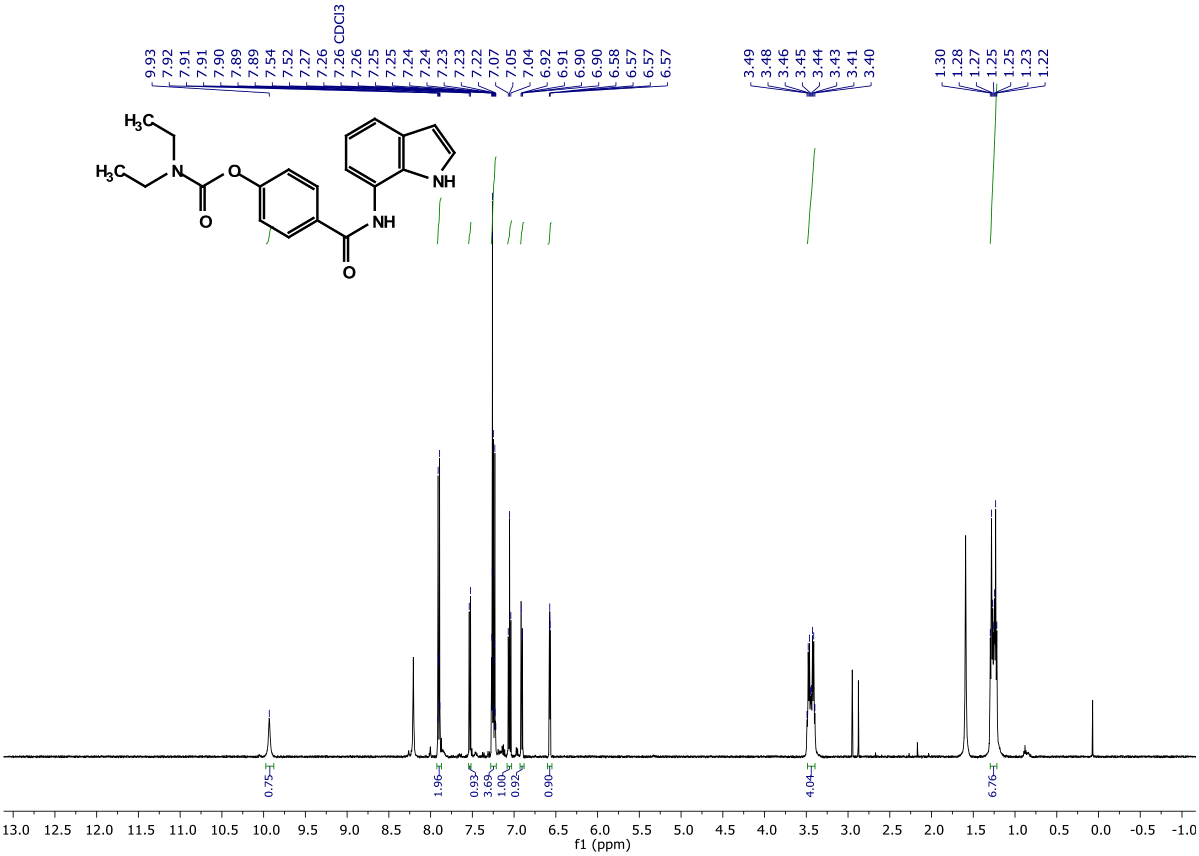

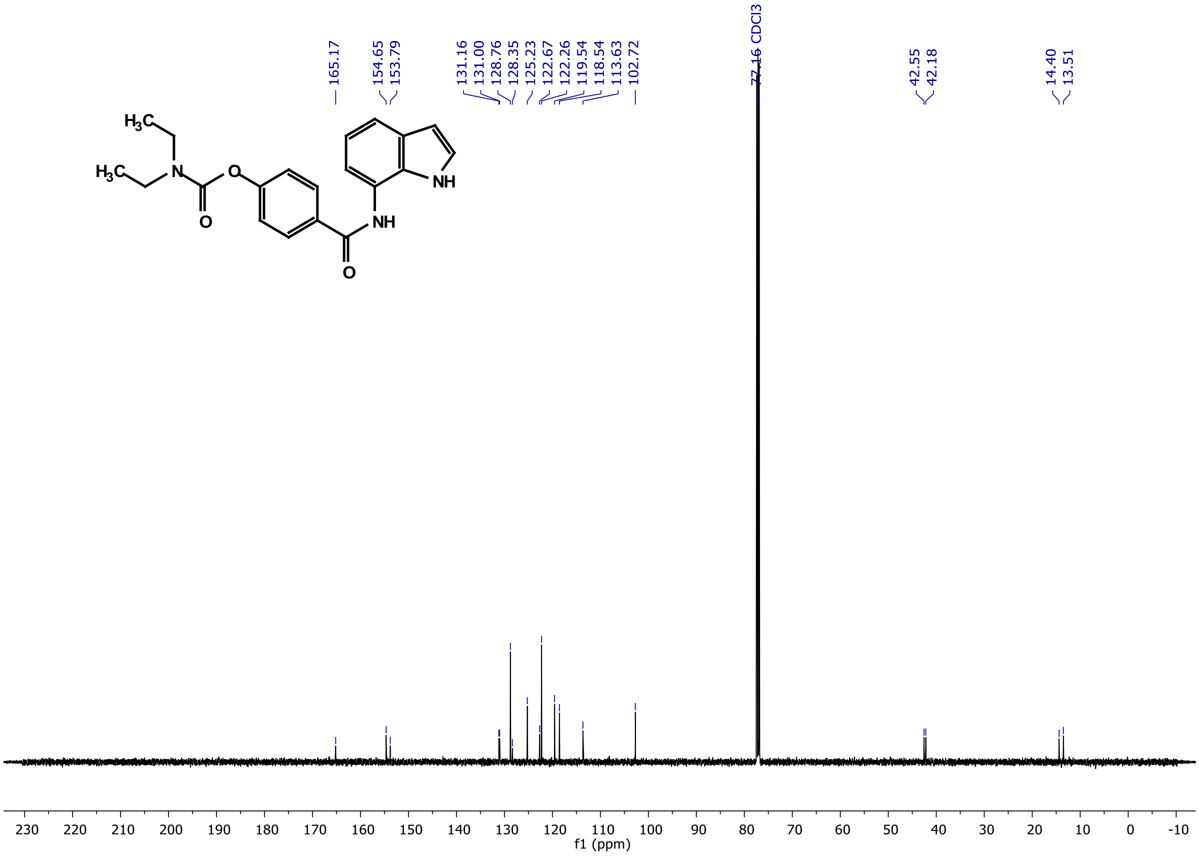

4-(Methyl(1-methyl-1*H*-indol-7-yl)carbamoyl)phenyl diethylcarbamate (**1al**)

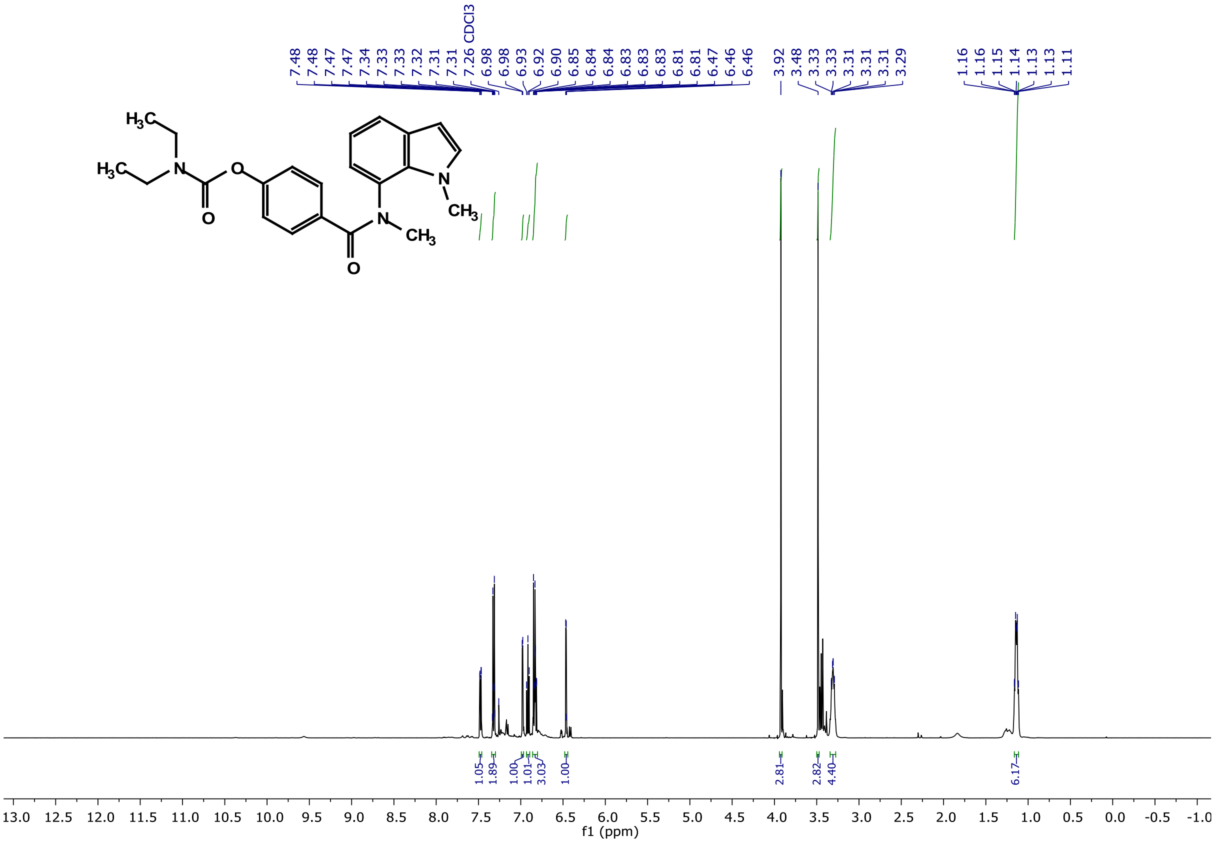

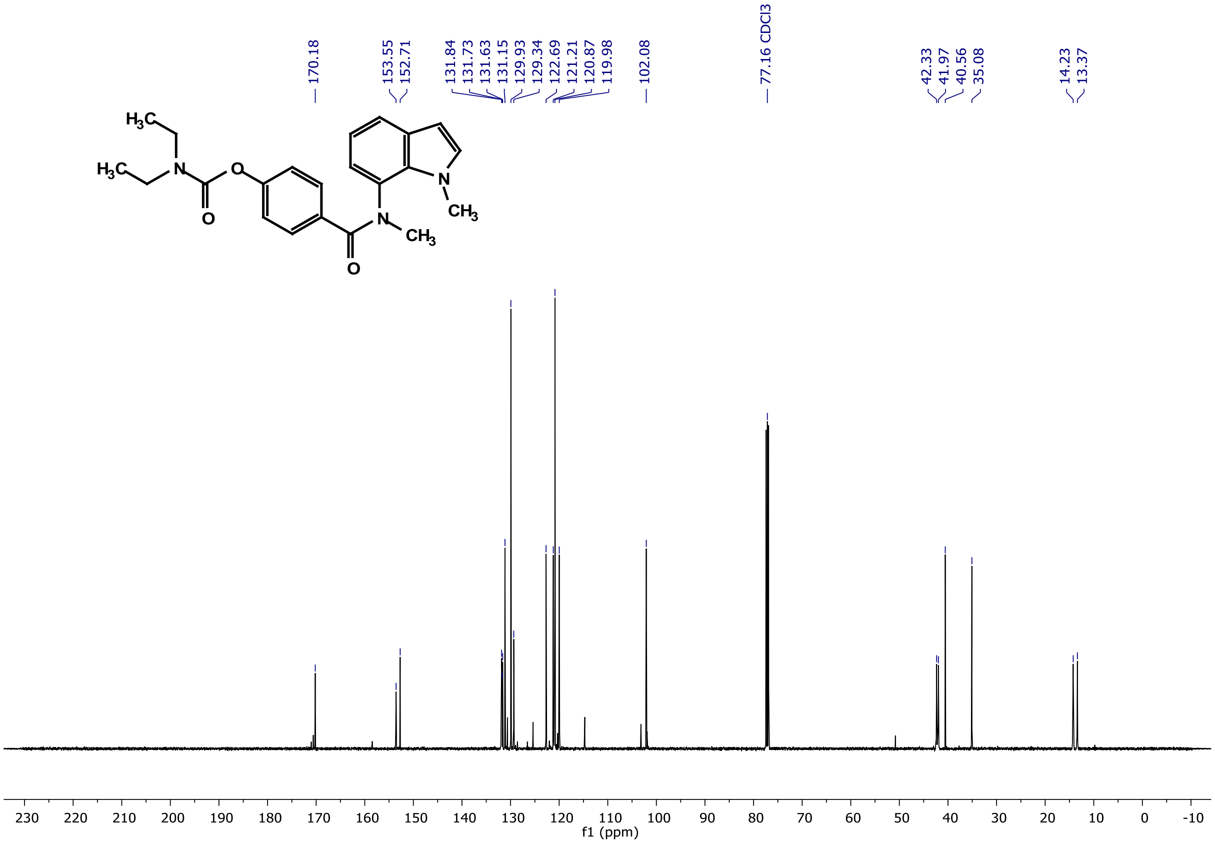

2-Chloro-5-(1-methyl-1*H*-indol-6-yl)pyridin-3-amine (**SI-4**)

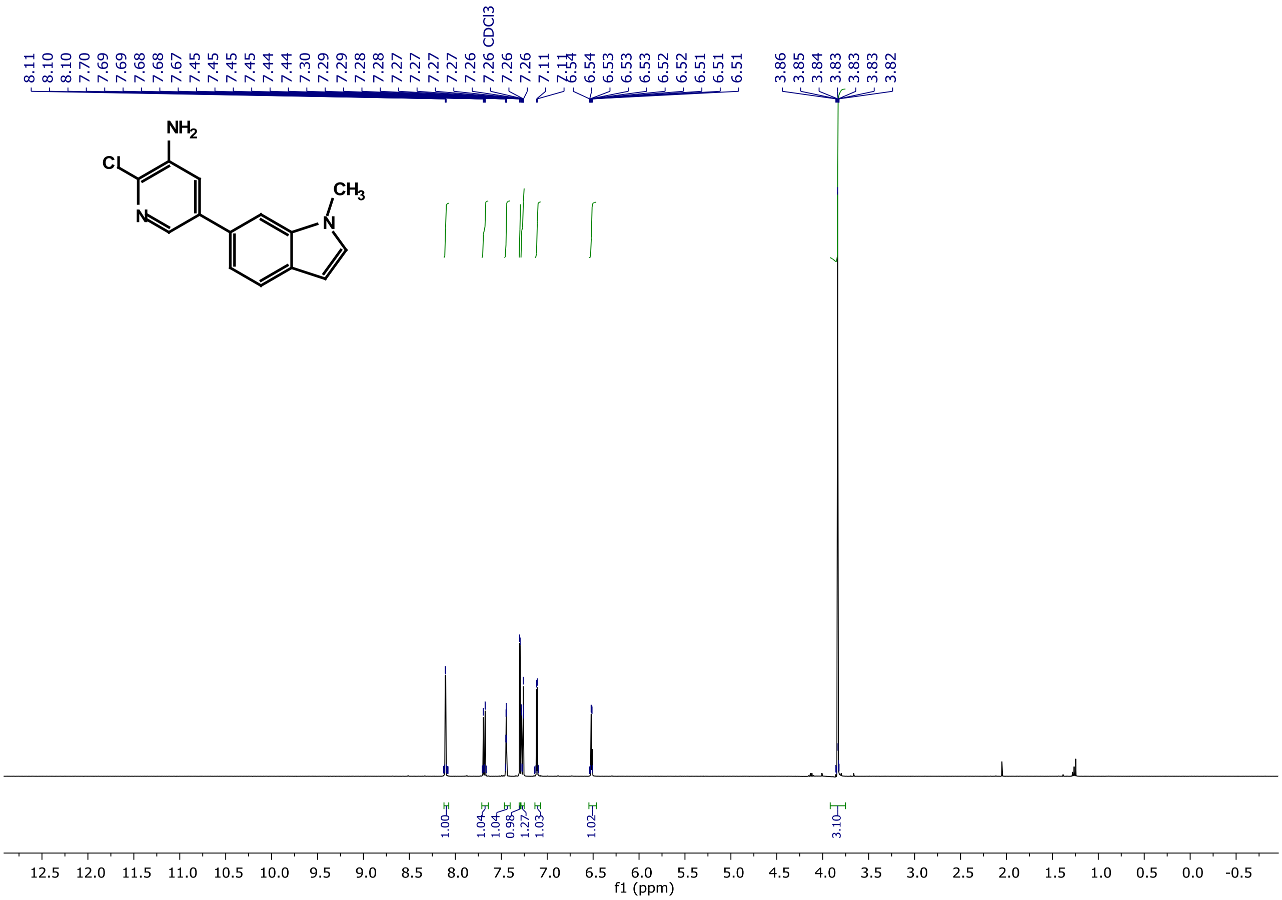

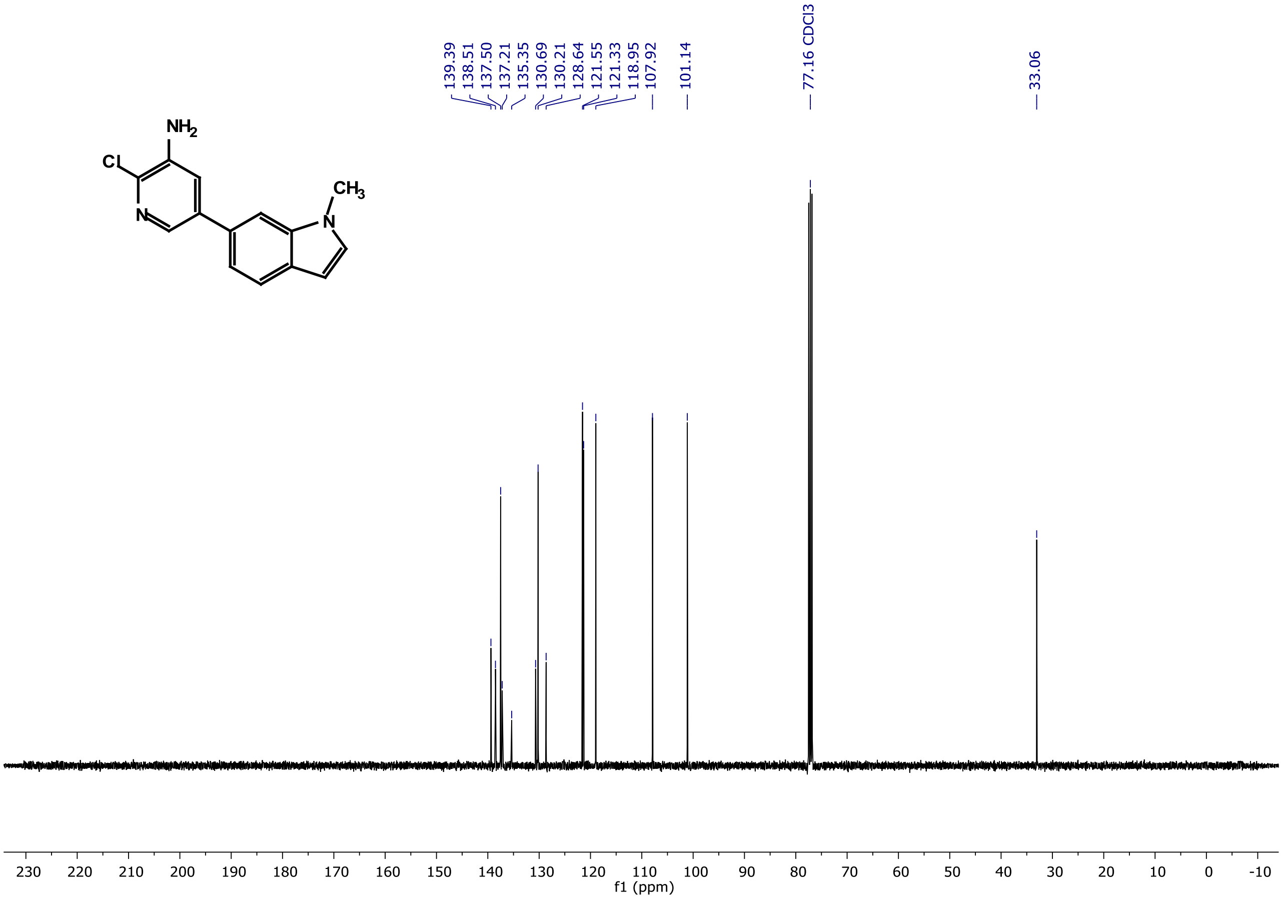

N-(2-chloro-5-(1-methyl-1*H*-indol-6-yl)pyridin-3-yl)-*N*-(phenylsulfonyl)benzenesulfonamide (**SI-5**)

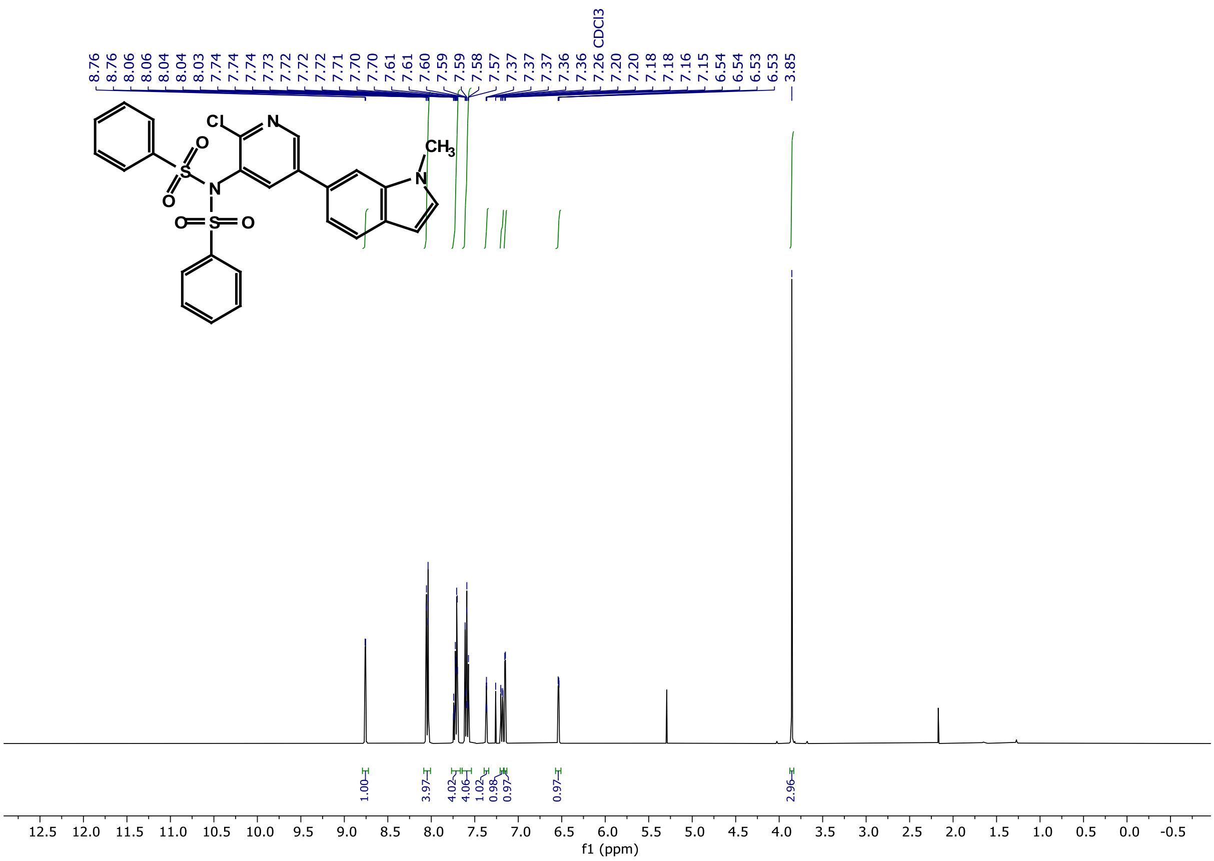

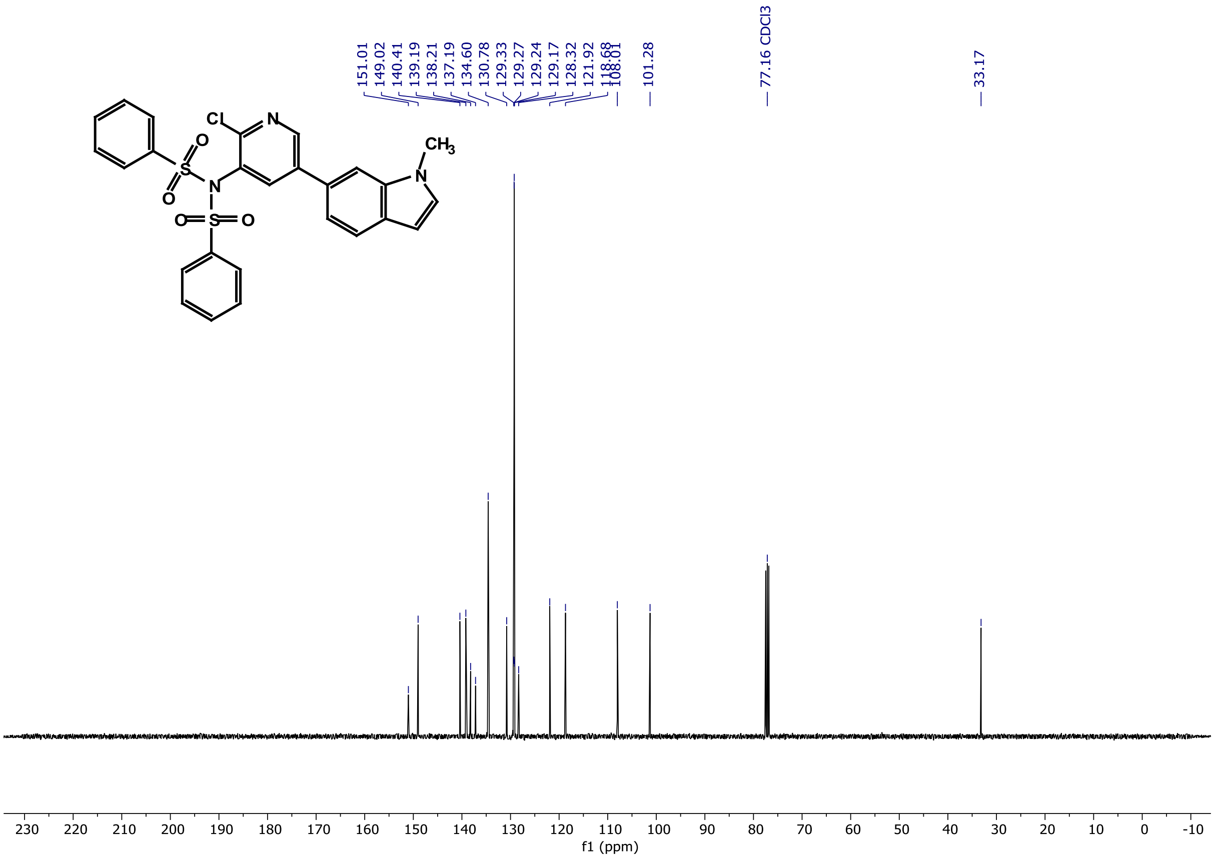

N-(2-chloro-5-(1-methyl-1*H*-indol-6-yl)pyridin-3-yl)benzenesulfonamide (**1ao**)

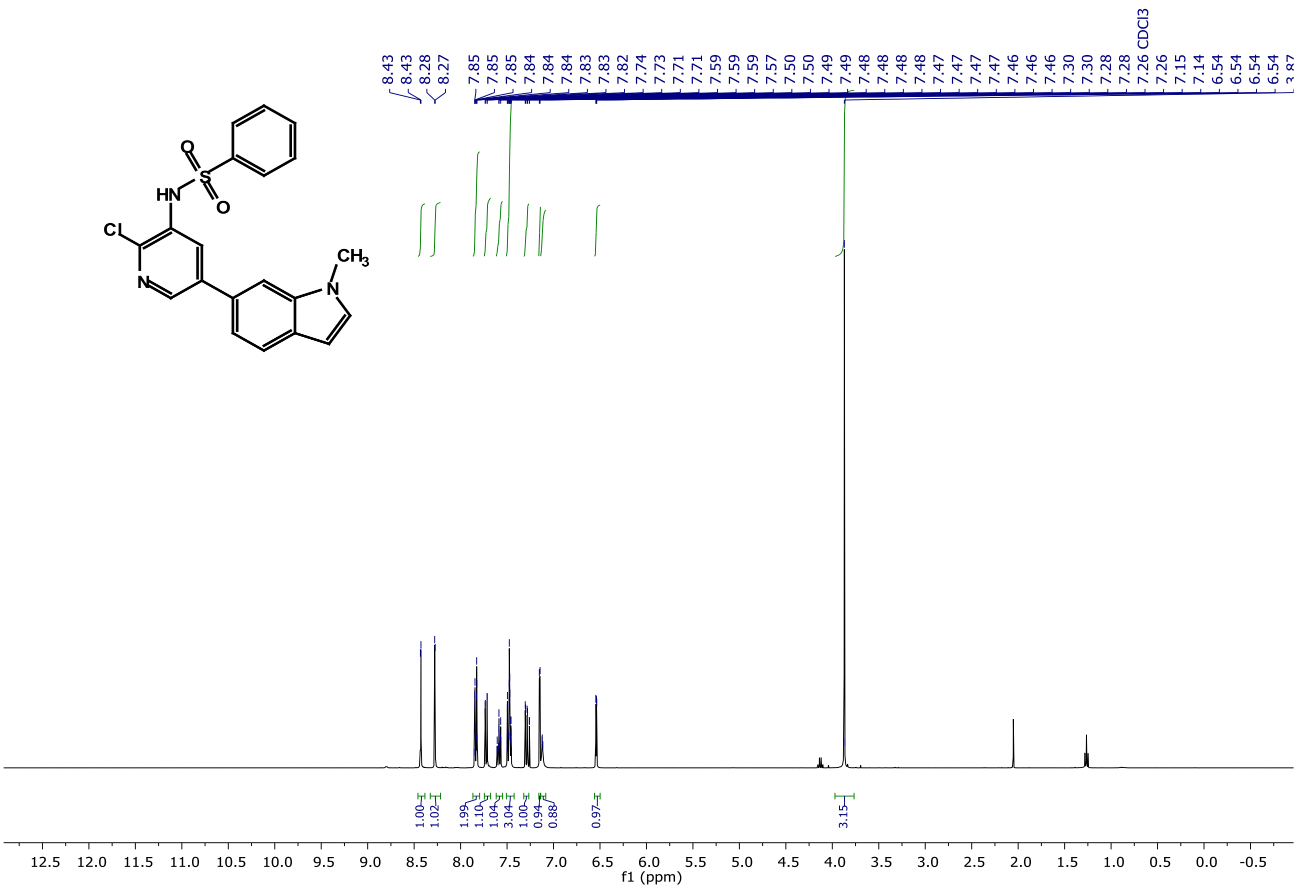

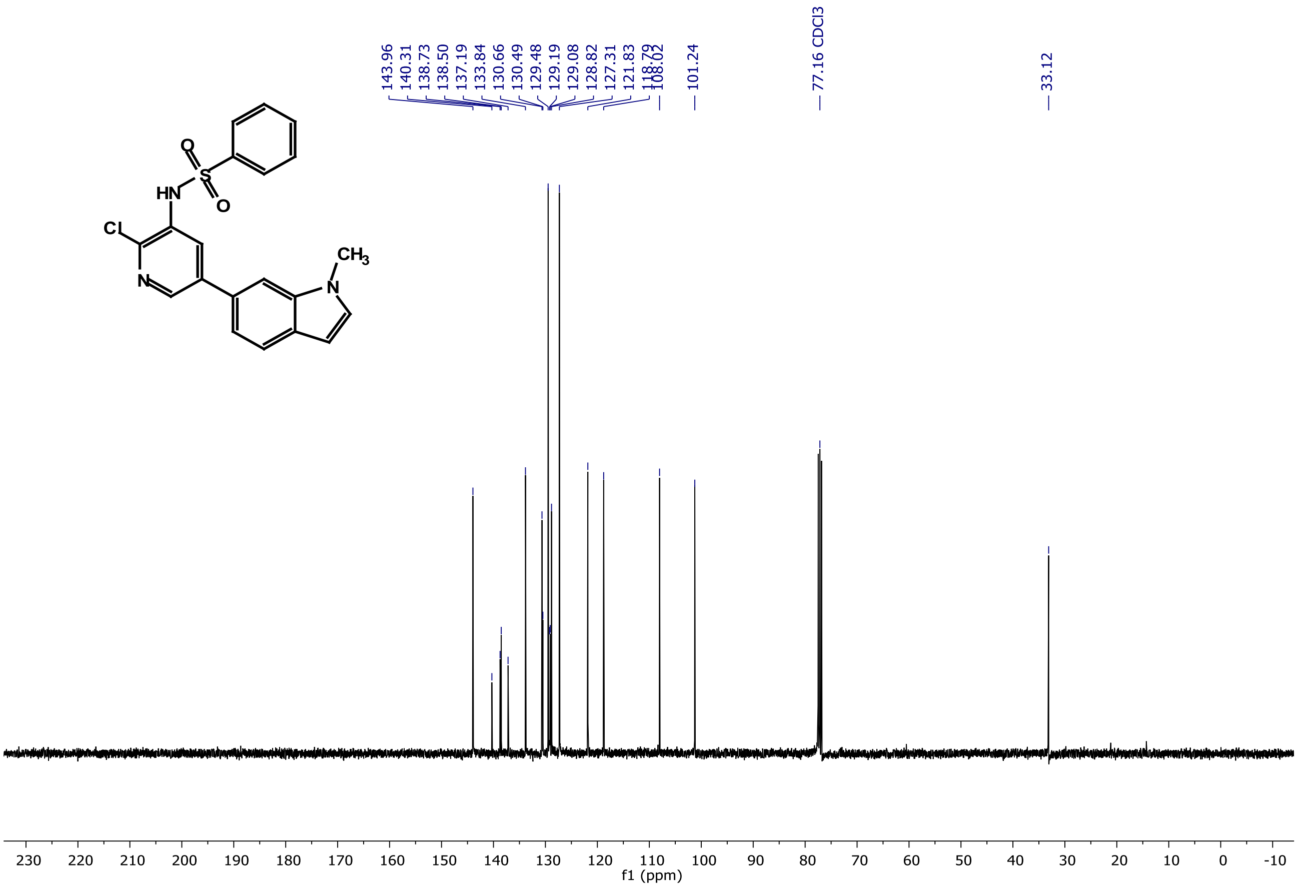

4-(2,6-dichloropyrimidin-4-yl)morpholine (**SI-6**)

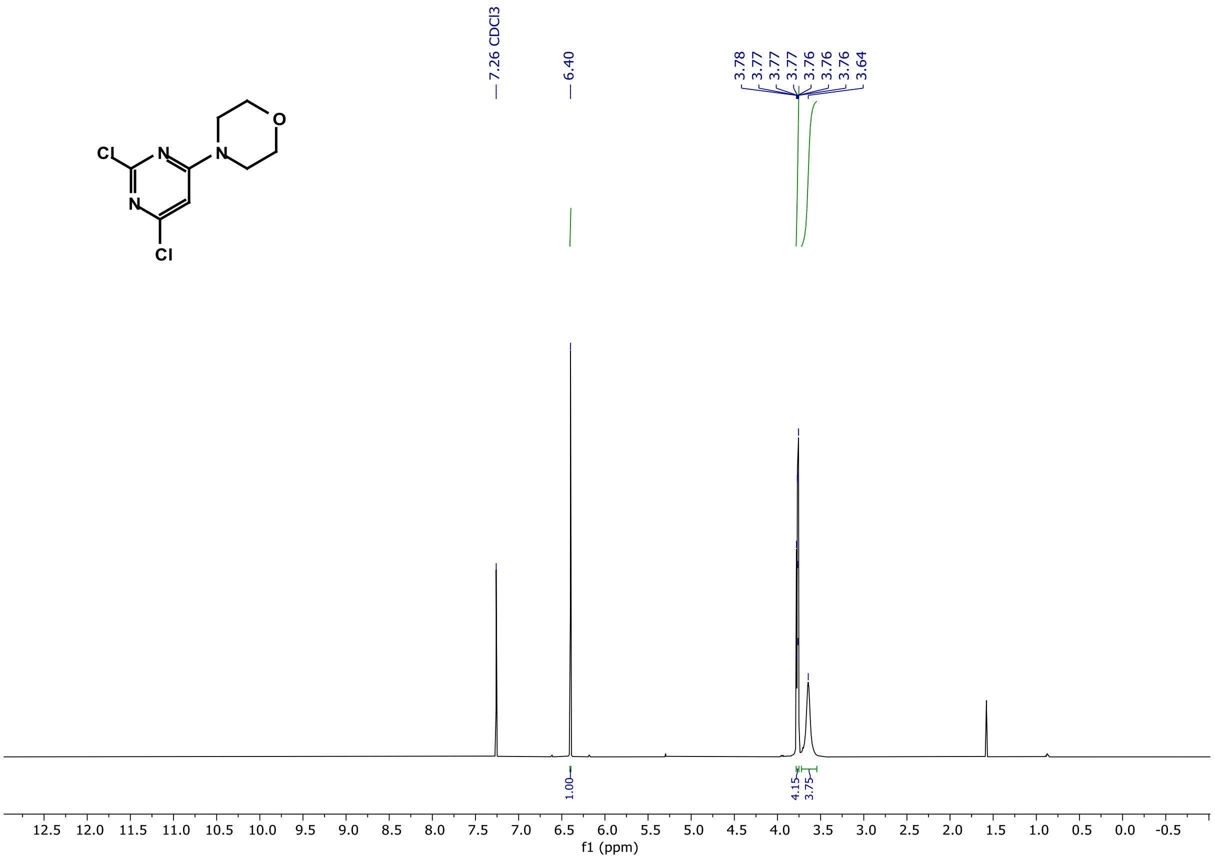

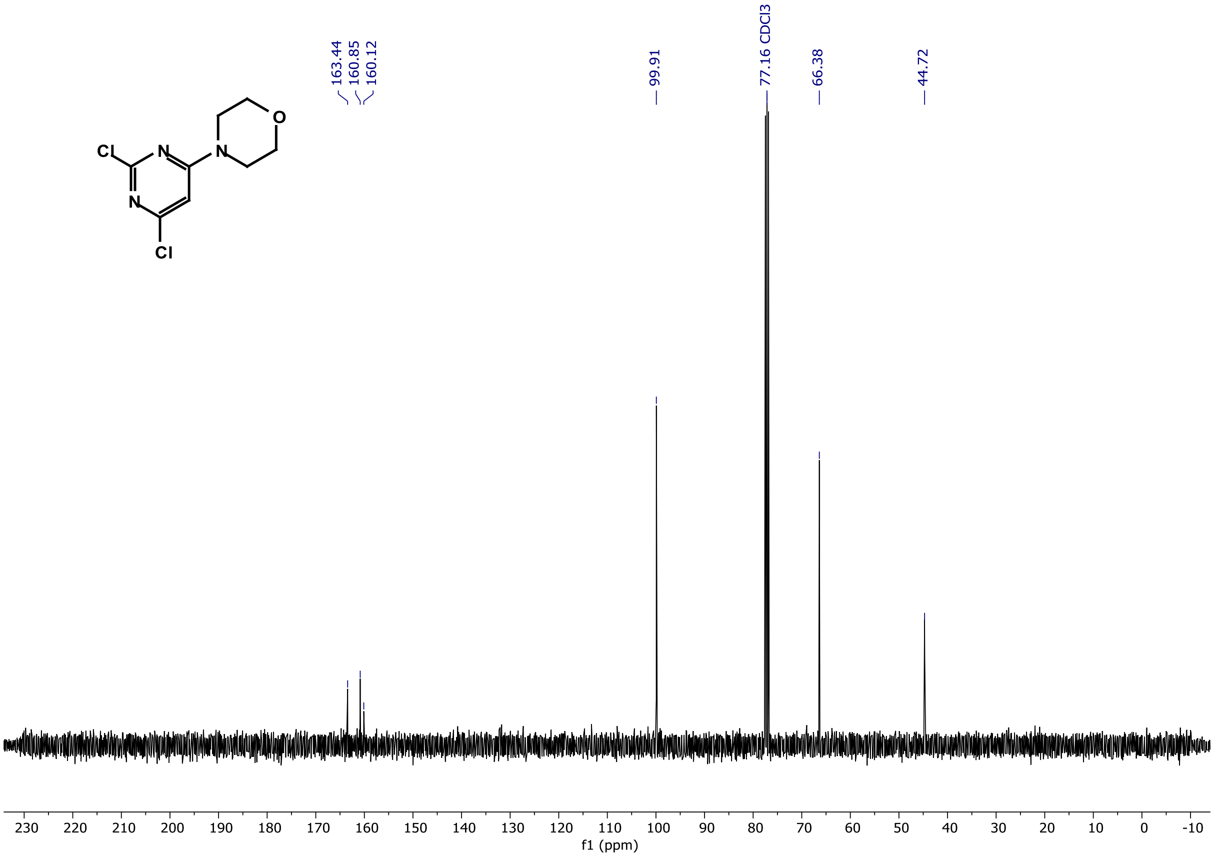

4-(6-chloro-2-(1-methyl-1*H*-indol-4-yl)pyrimidin-4-yl)morpholine (**1ar**)

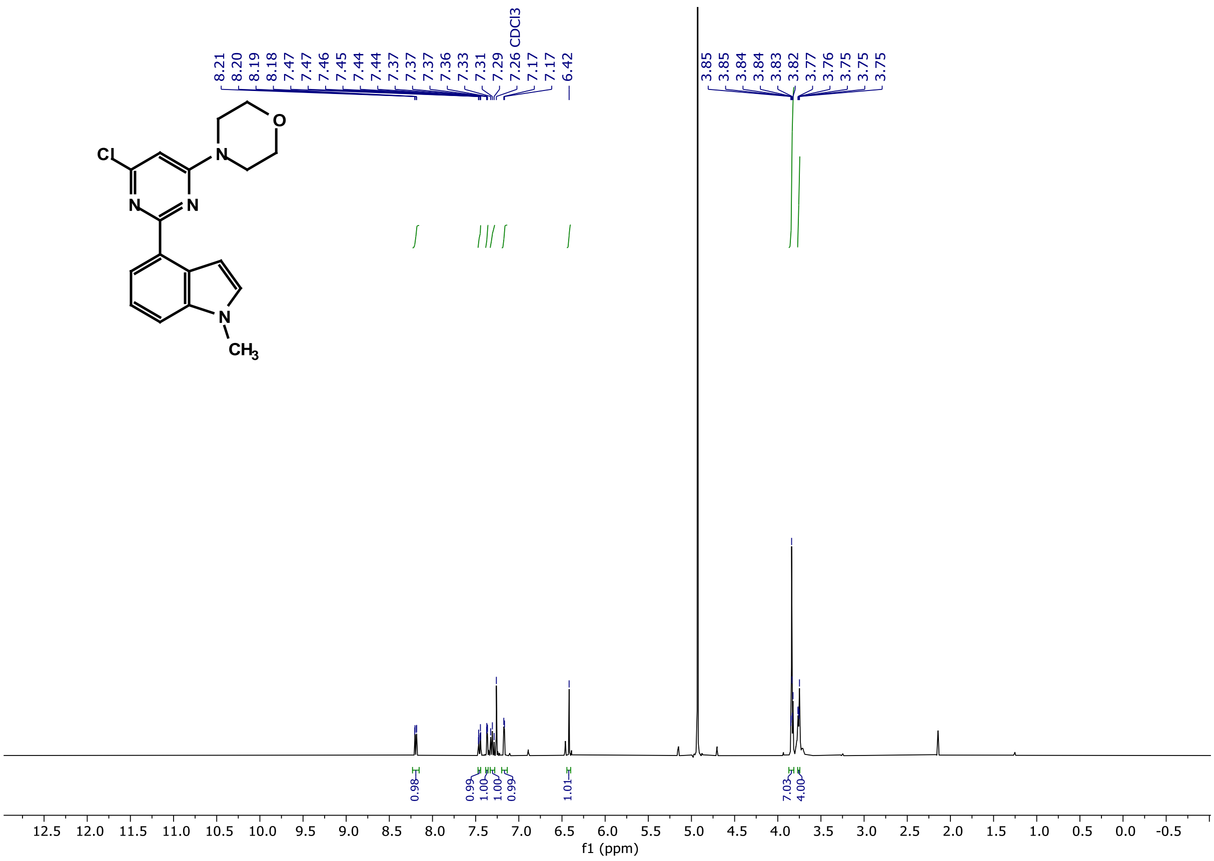

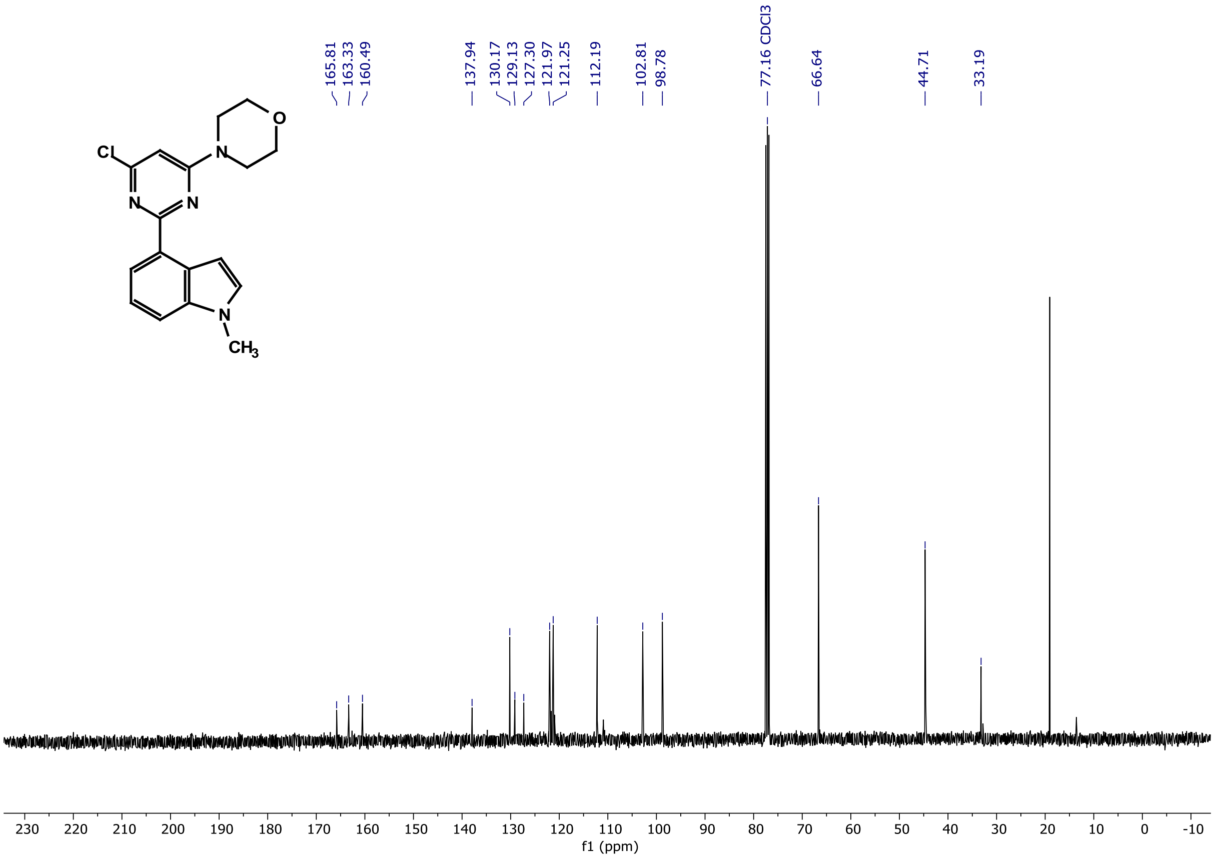

N-(2-formylphenyl)-*N*-methylformamide (**Ia**)

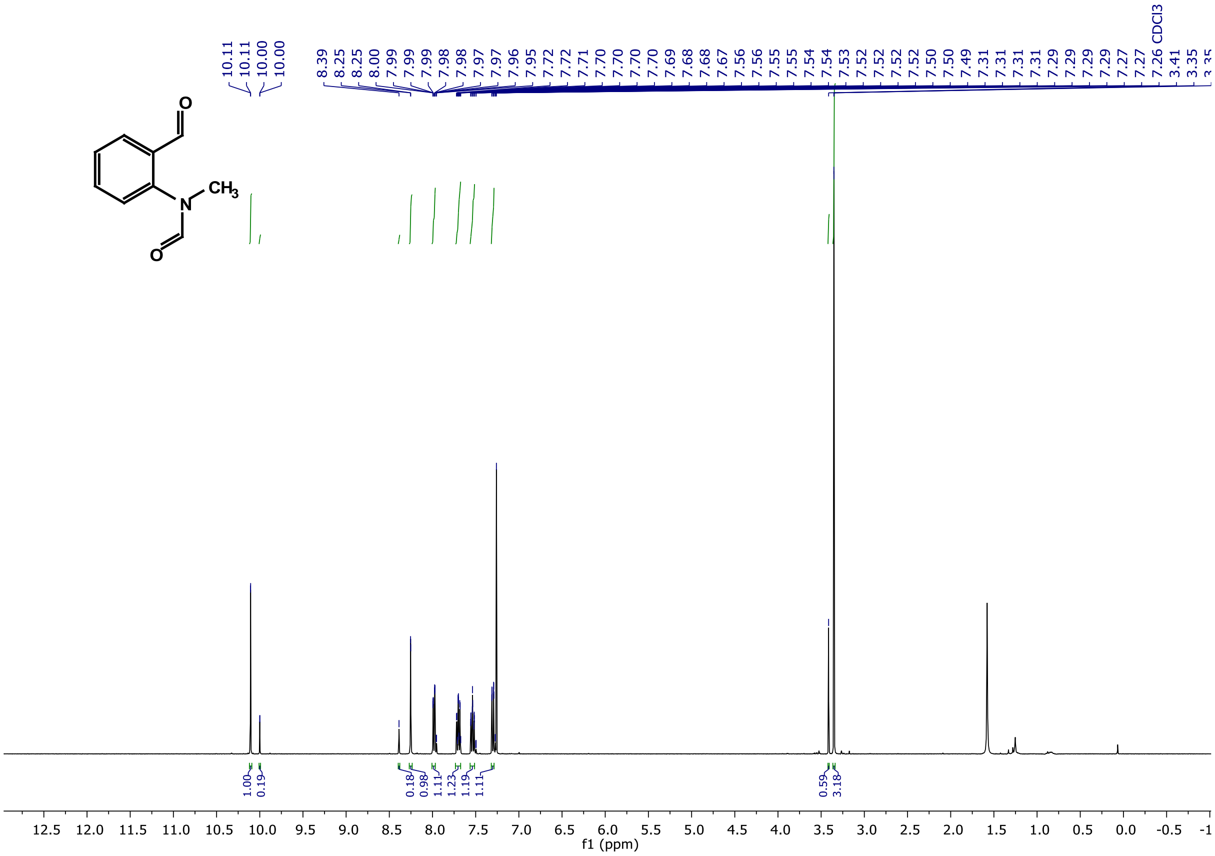

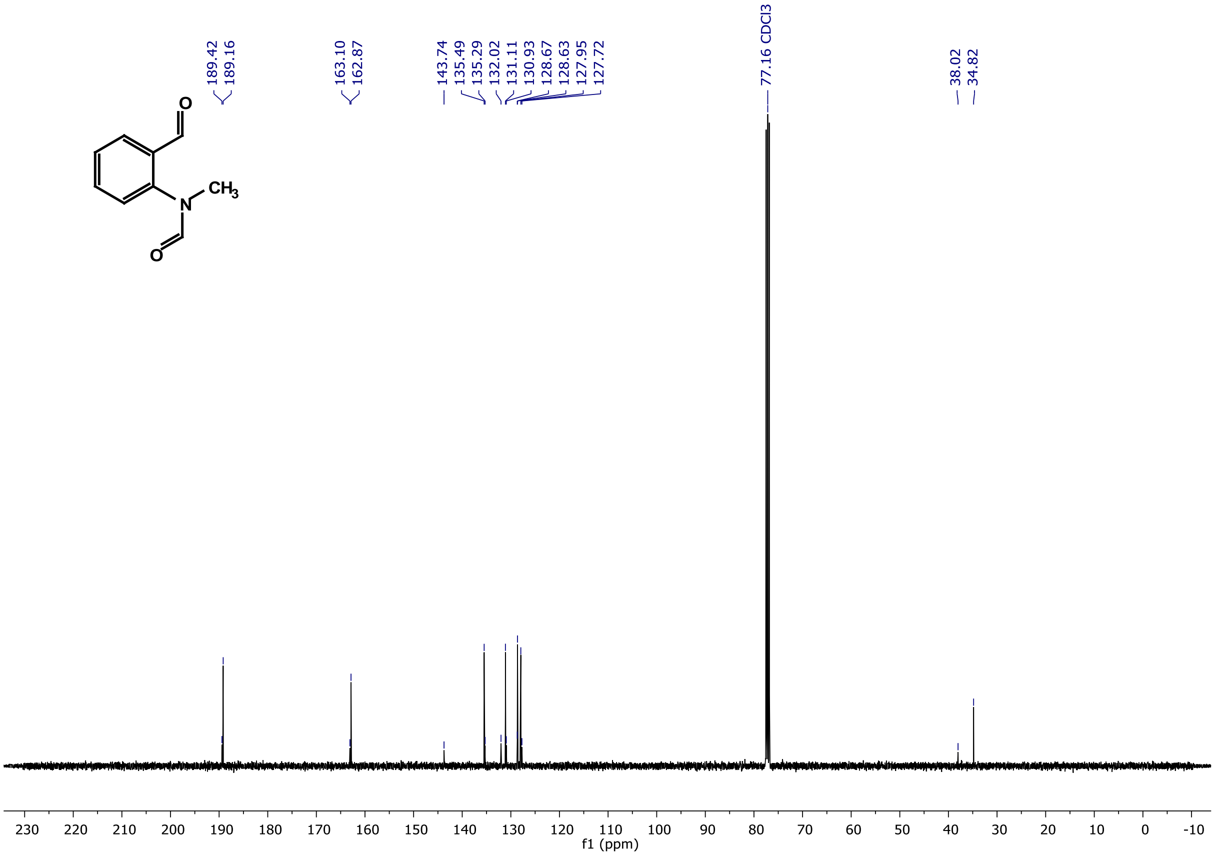

2-(*N*-methylformamido)benzamide (**IIa**)

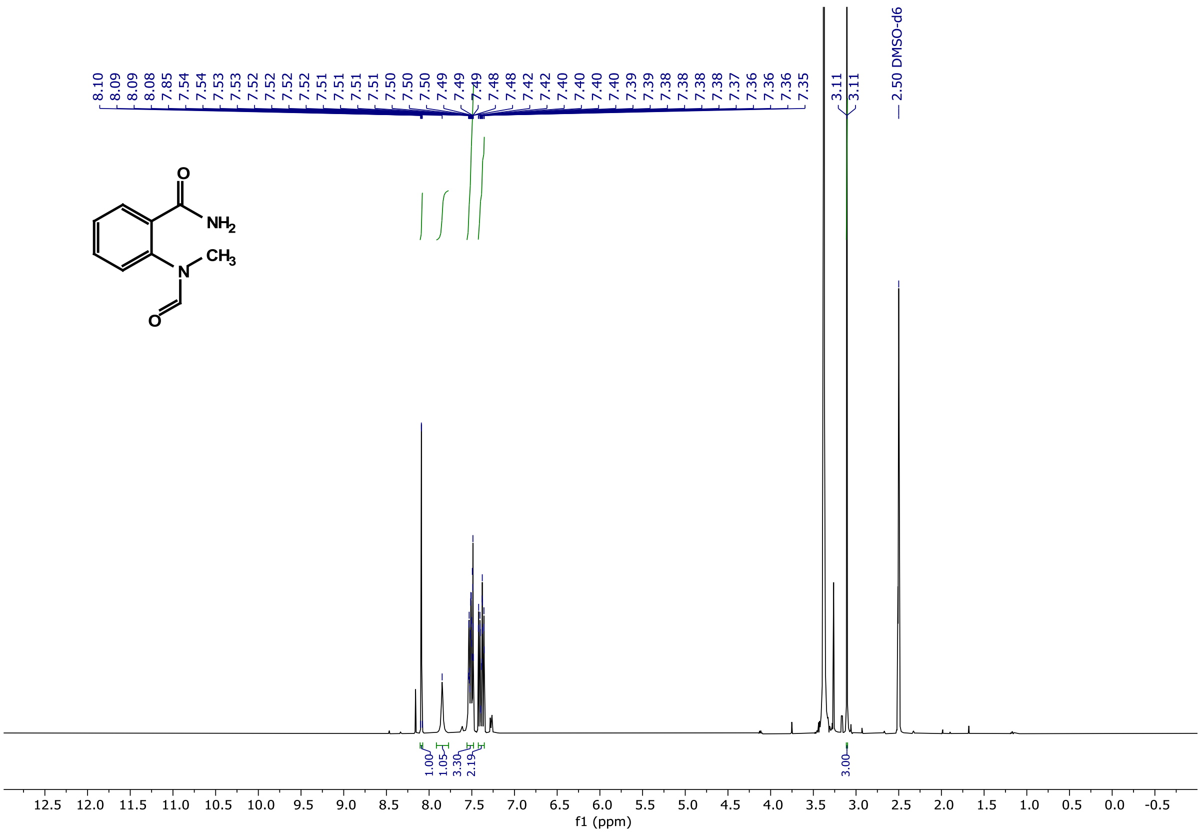

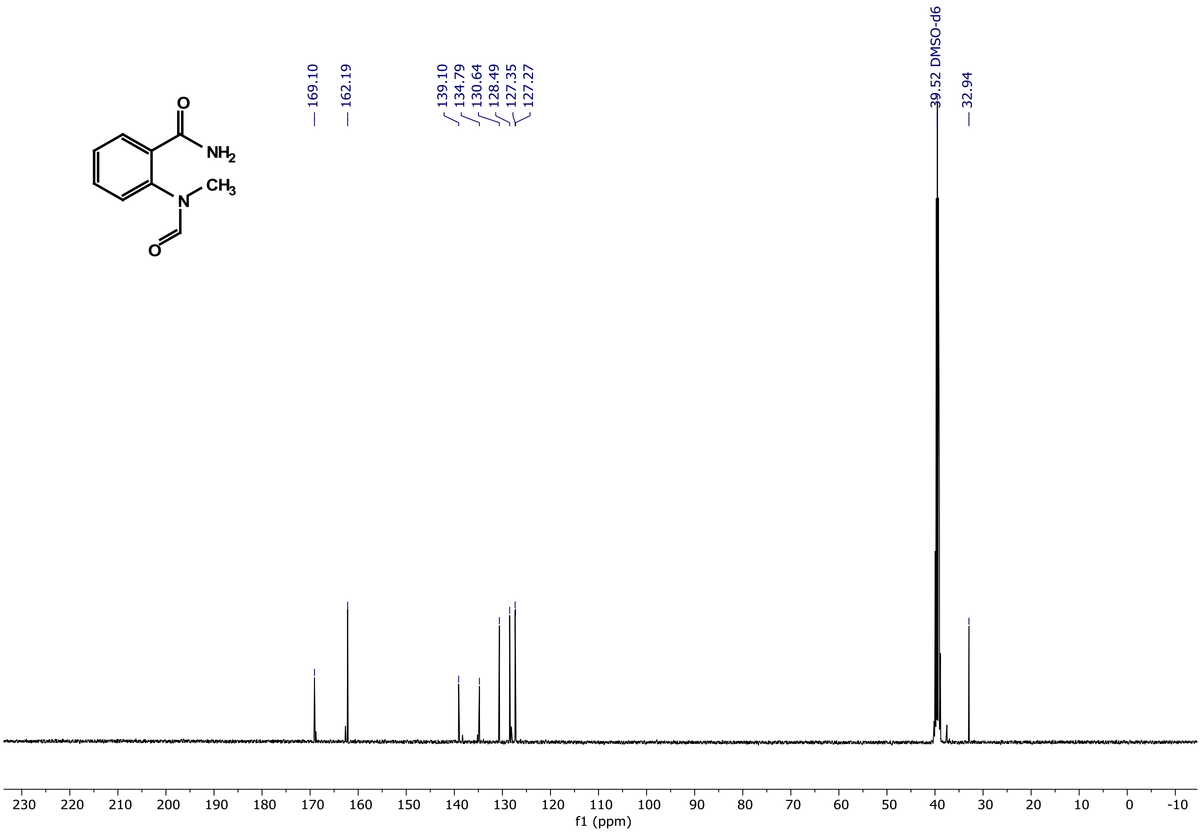

1-Methyl-1,4-dihydroquinazolin-4-ol (**VIIIa**)

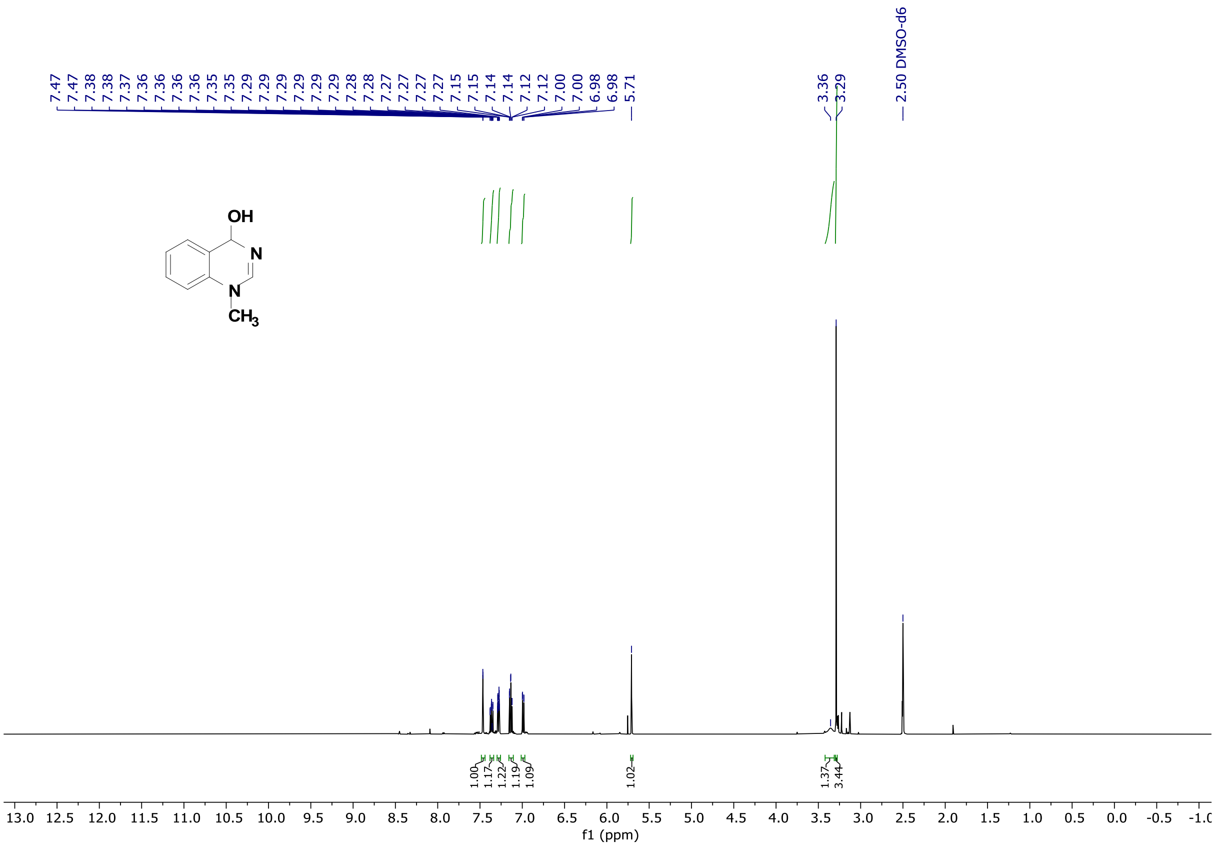

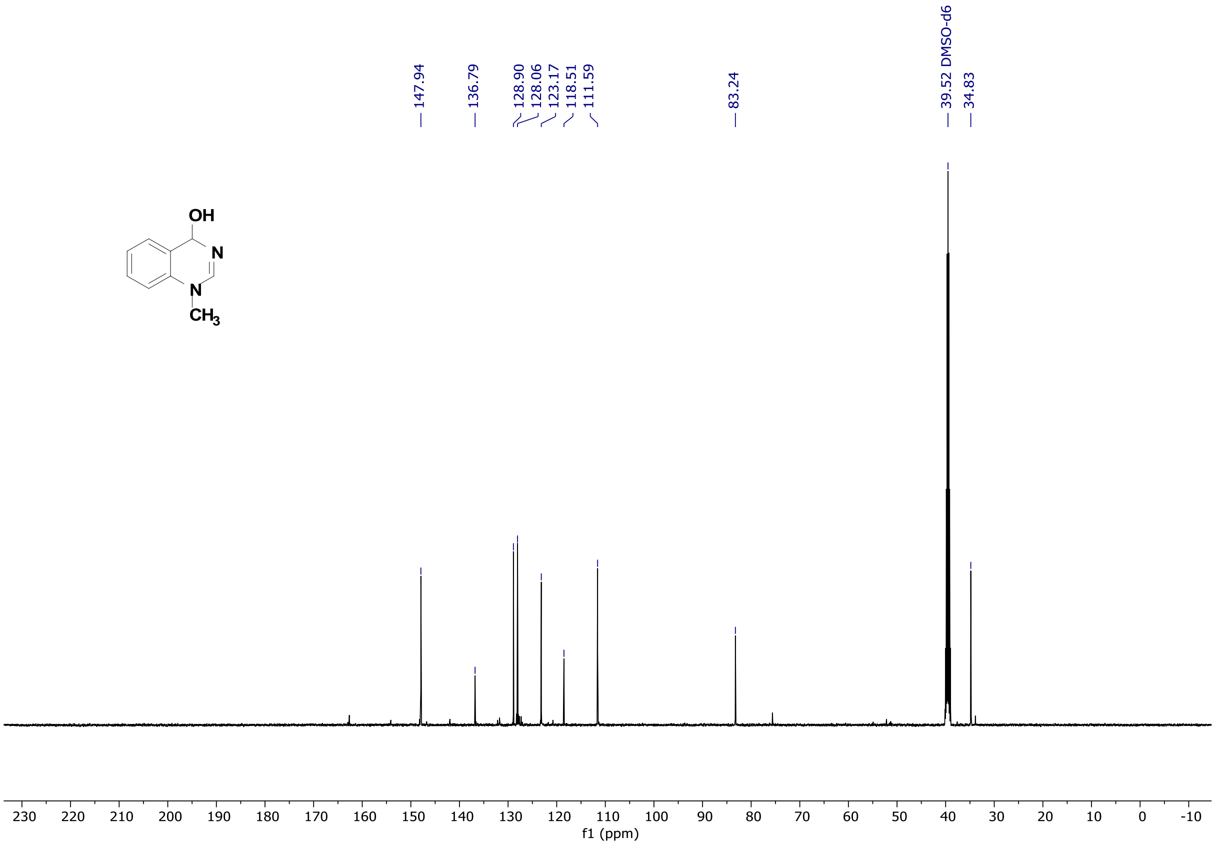

9 NMR SPECTRA OF PRODUCTS

1-Methyl-1*H*-benzo[*d*]imidazole (**2a**)

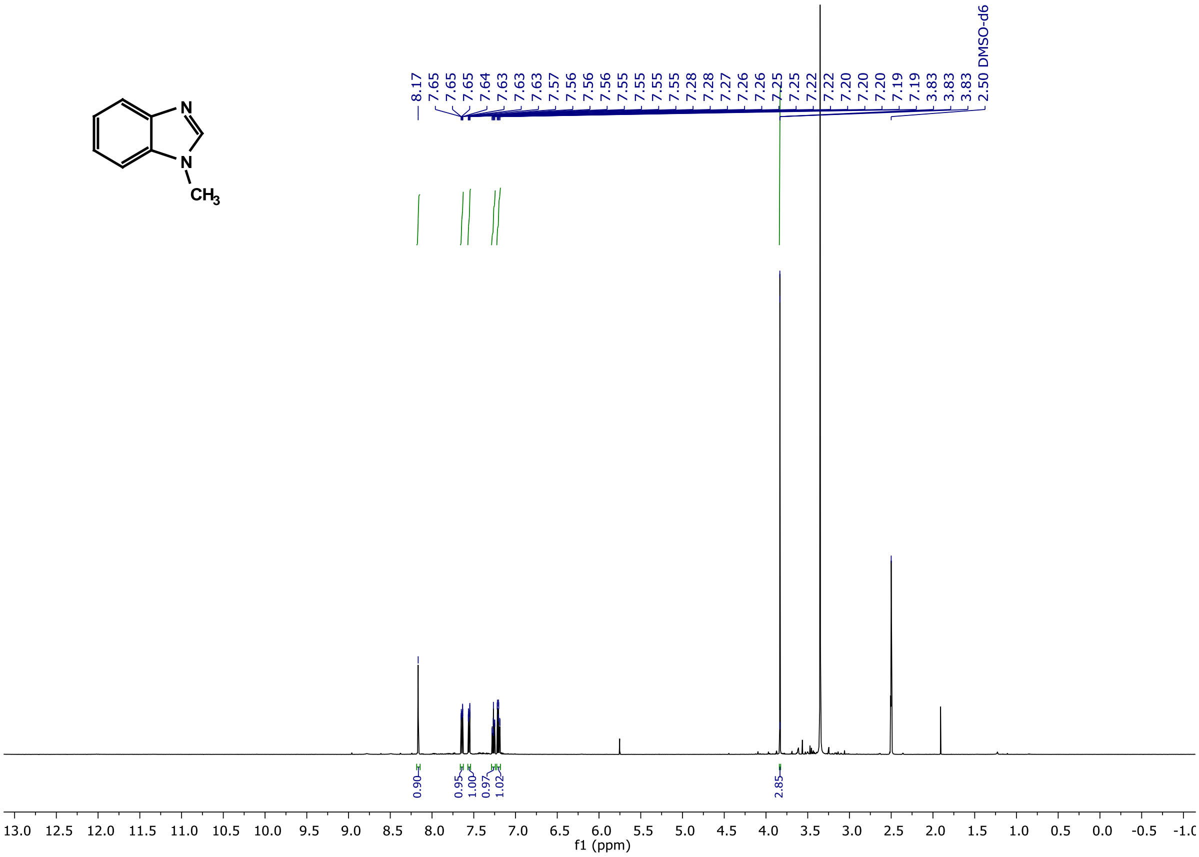

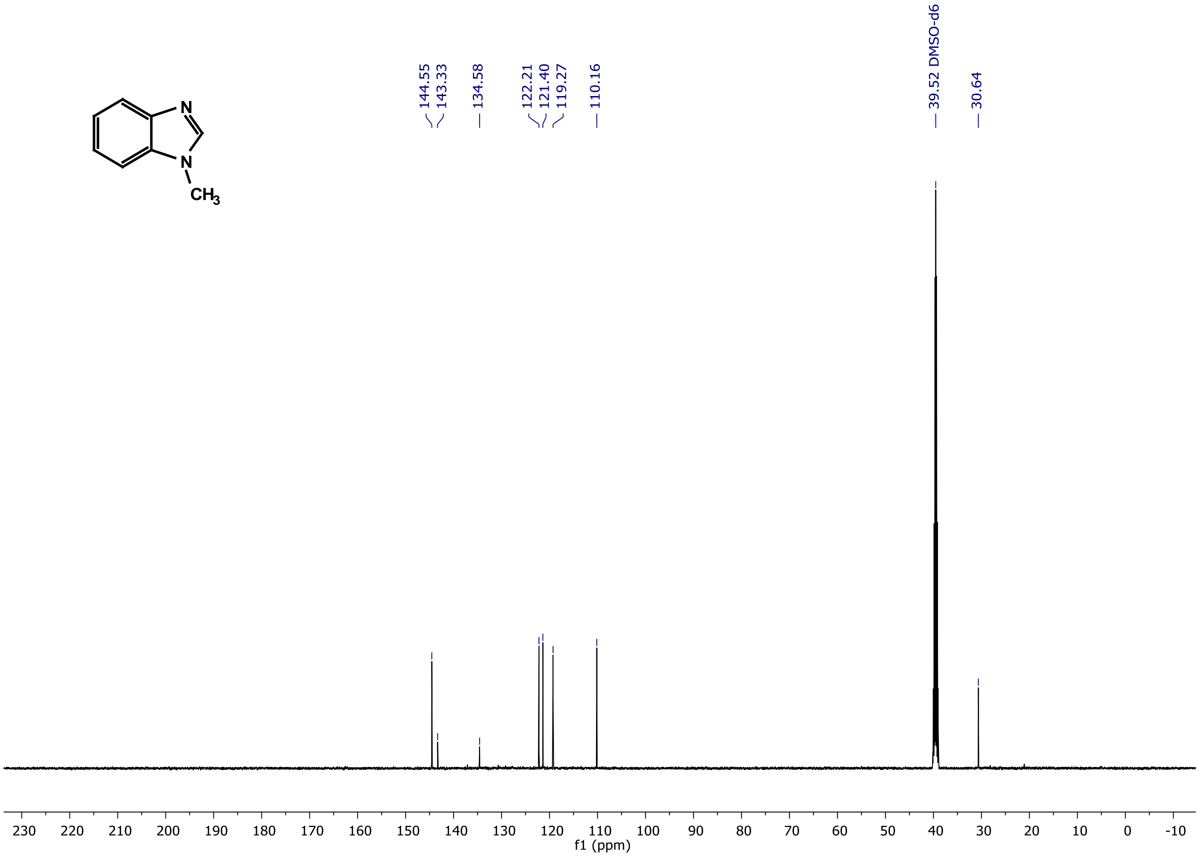

1-Methyl-1*H*-benzo[*d*]imidazole-2-^{13}C (**^{13}C-2a**)

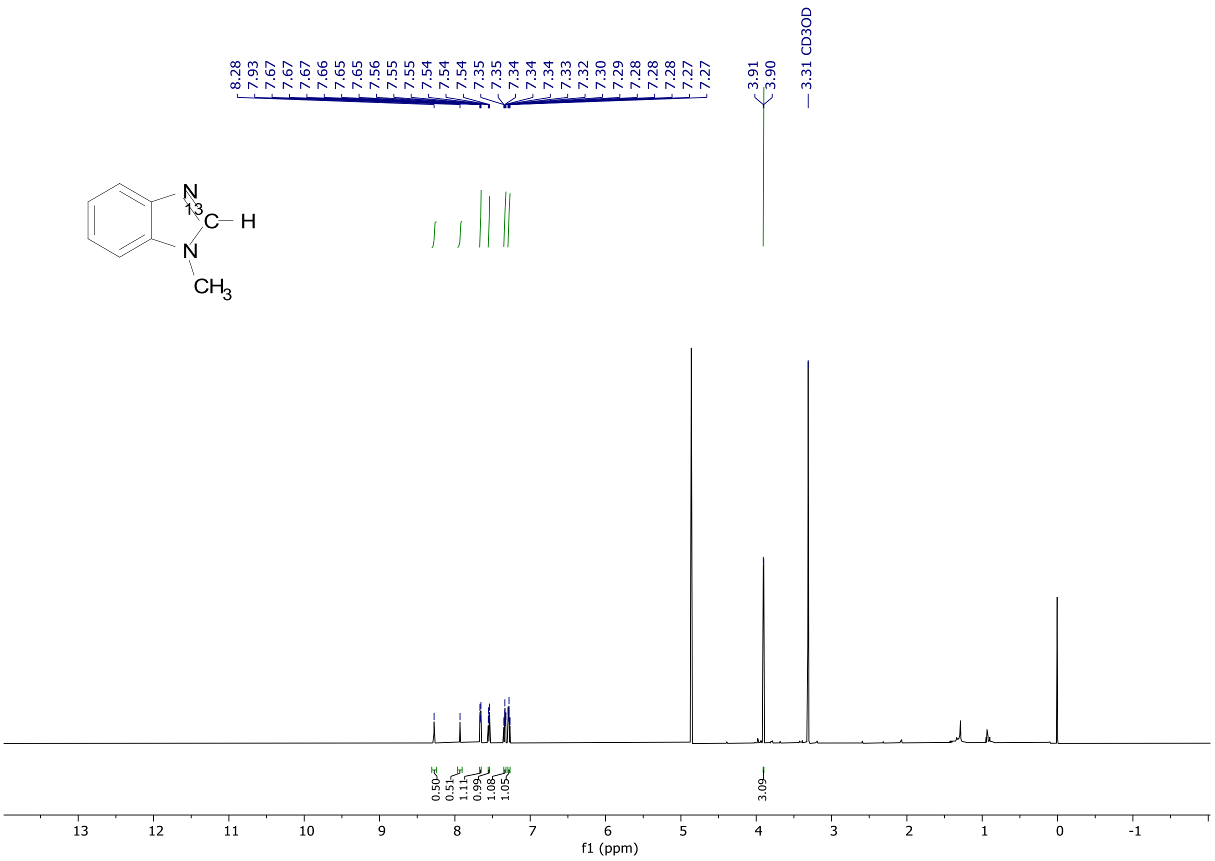

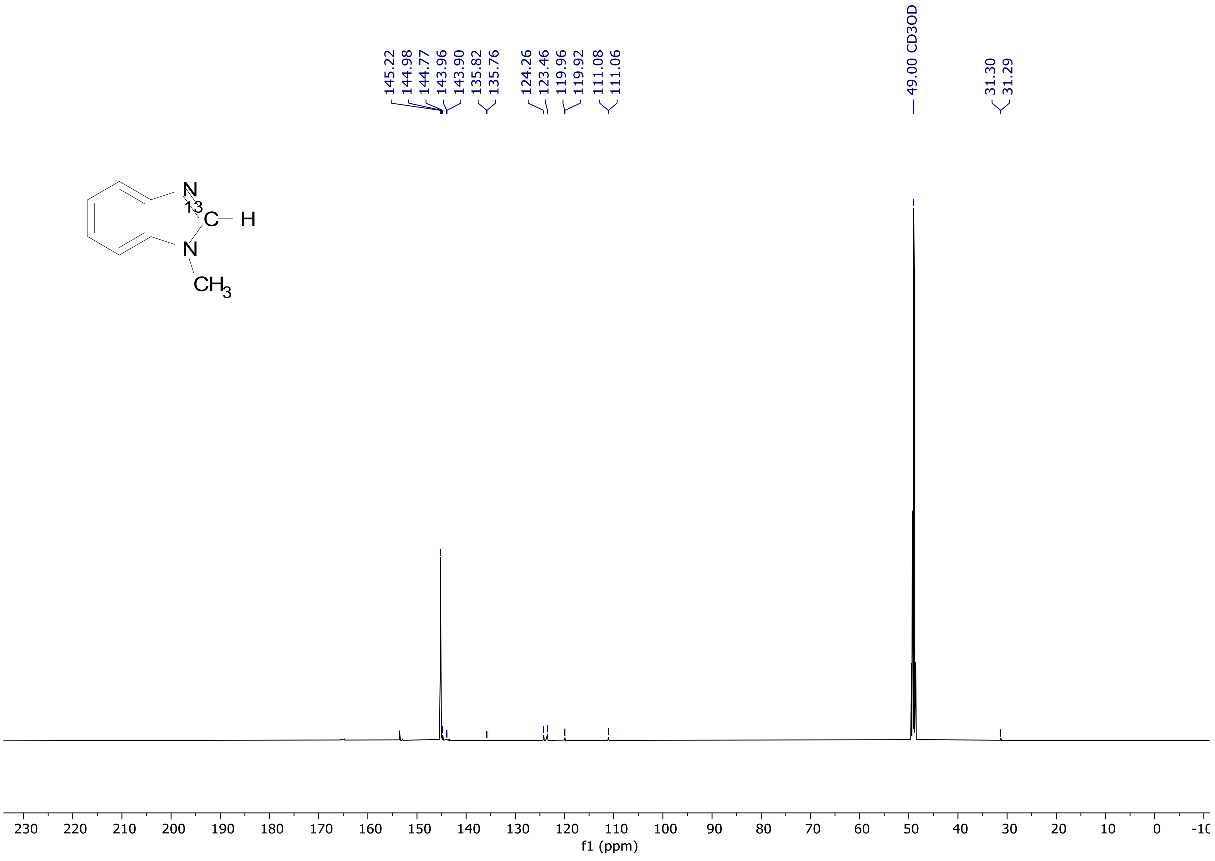

4-Amino-1-methylquinazolin-1-ium 2,2,2-trifluoroacetate (**3a**)

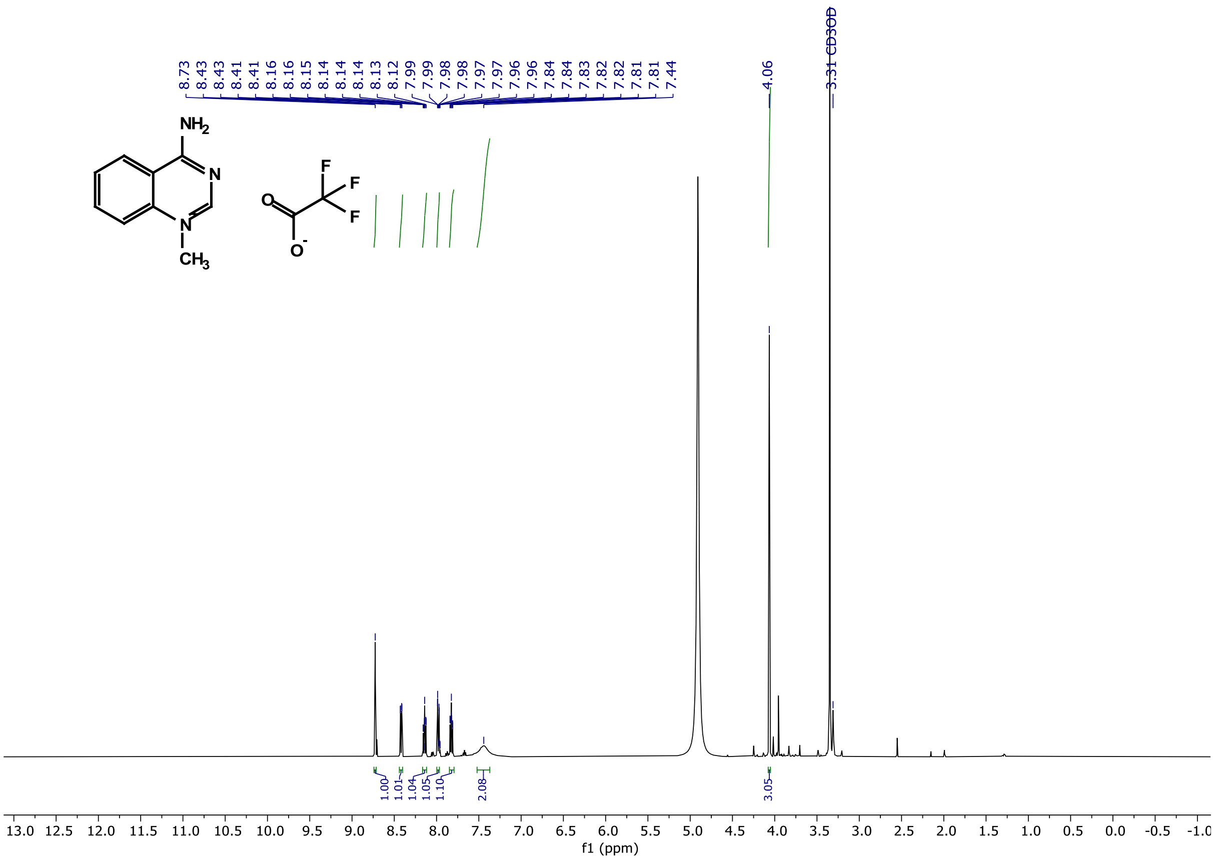

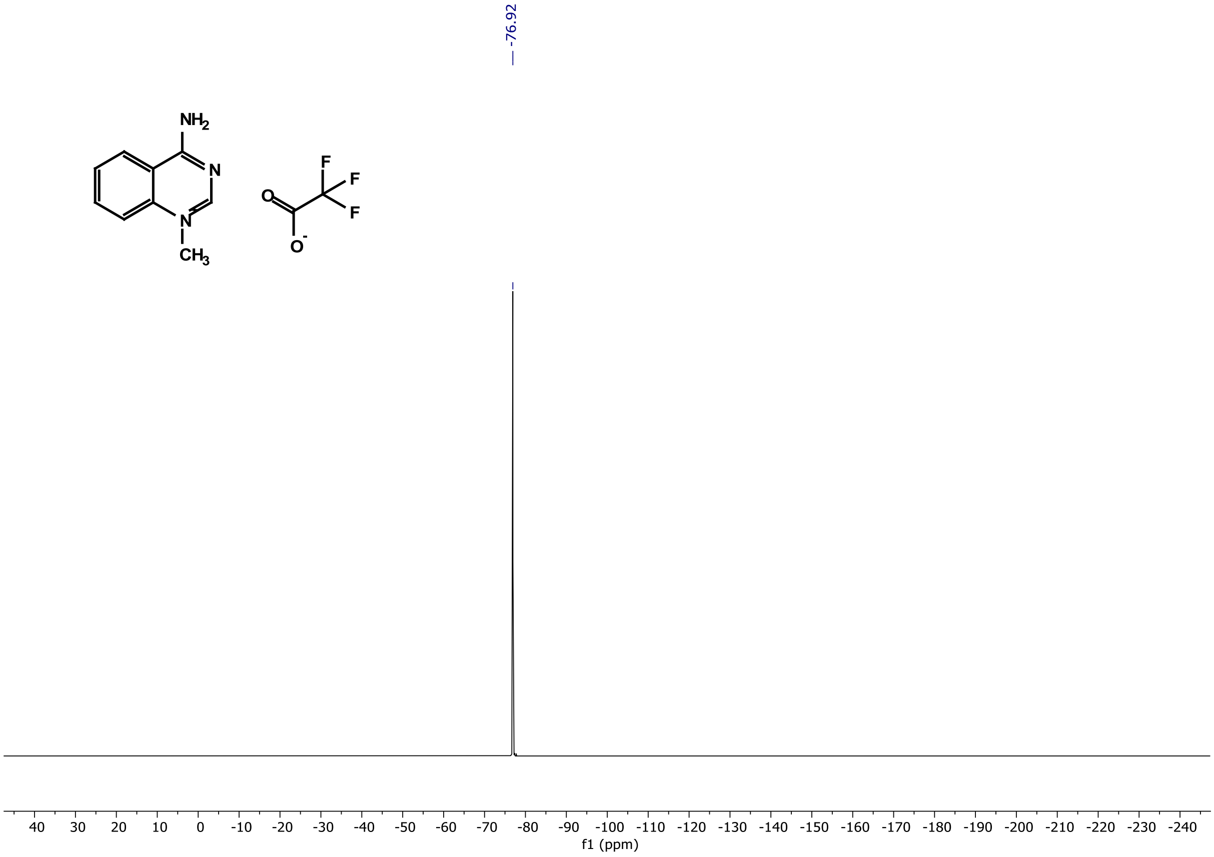

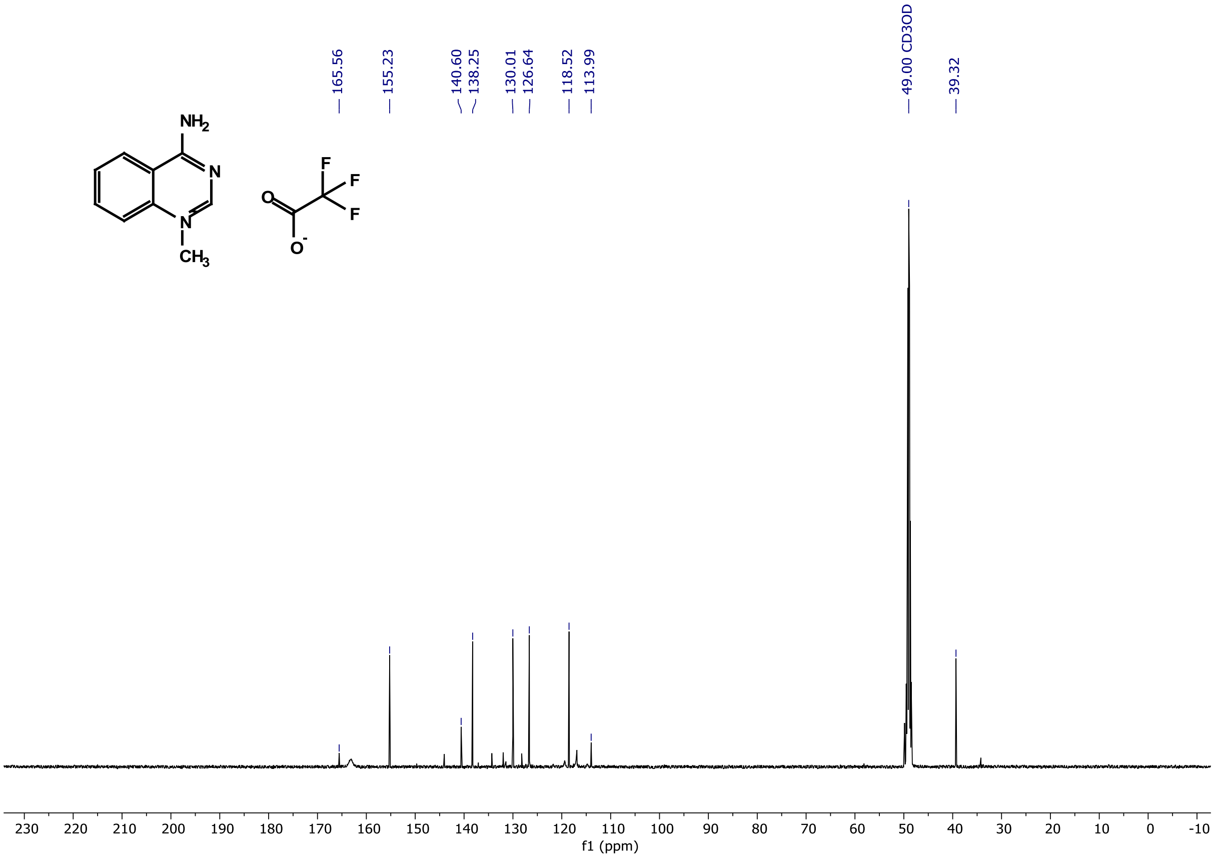

6-Methoxy-1-methyl-1*H*-benzo[*d*]imidazole (**2b**)

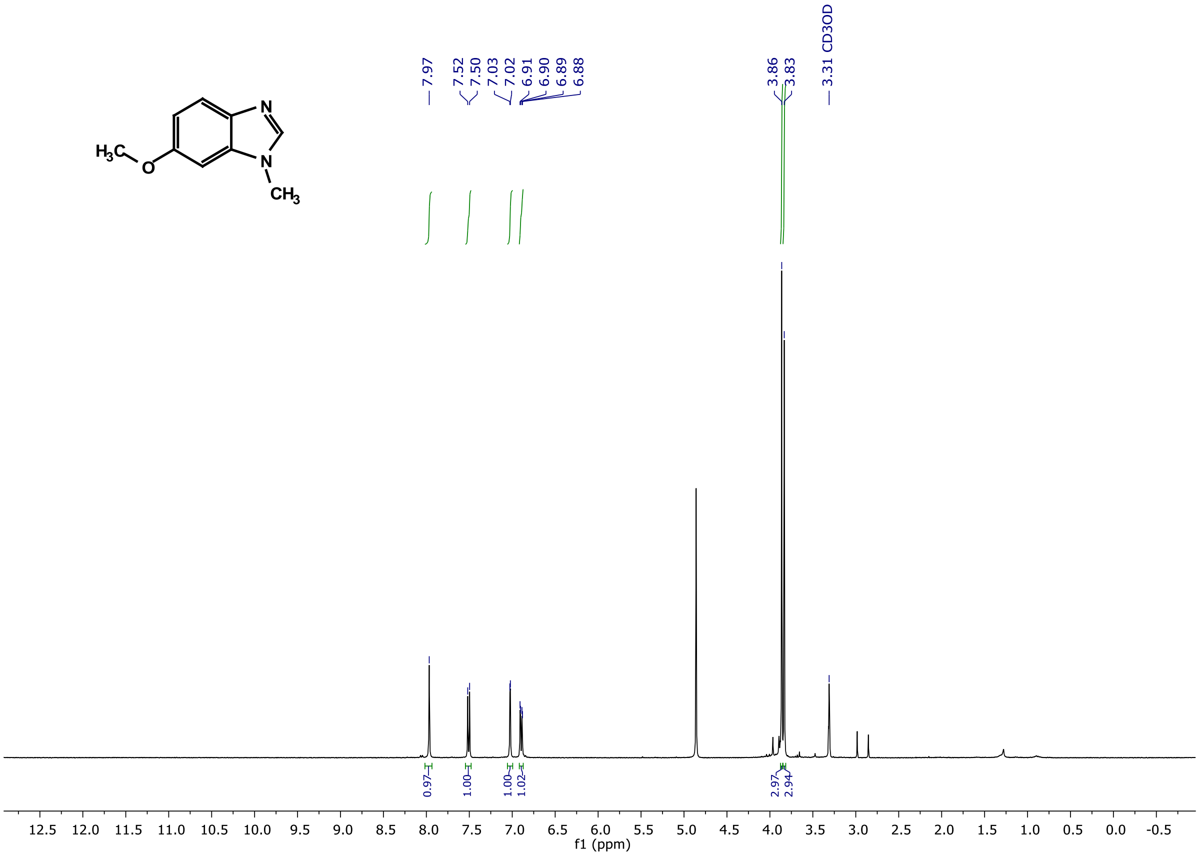

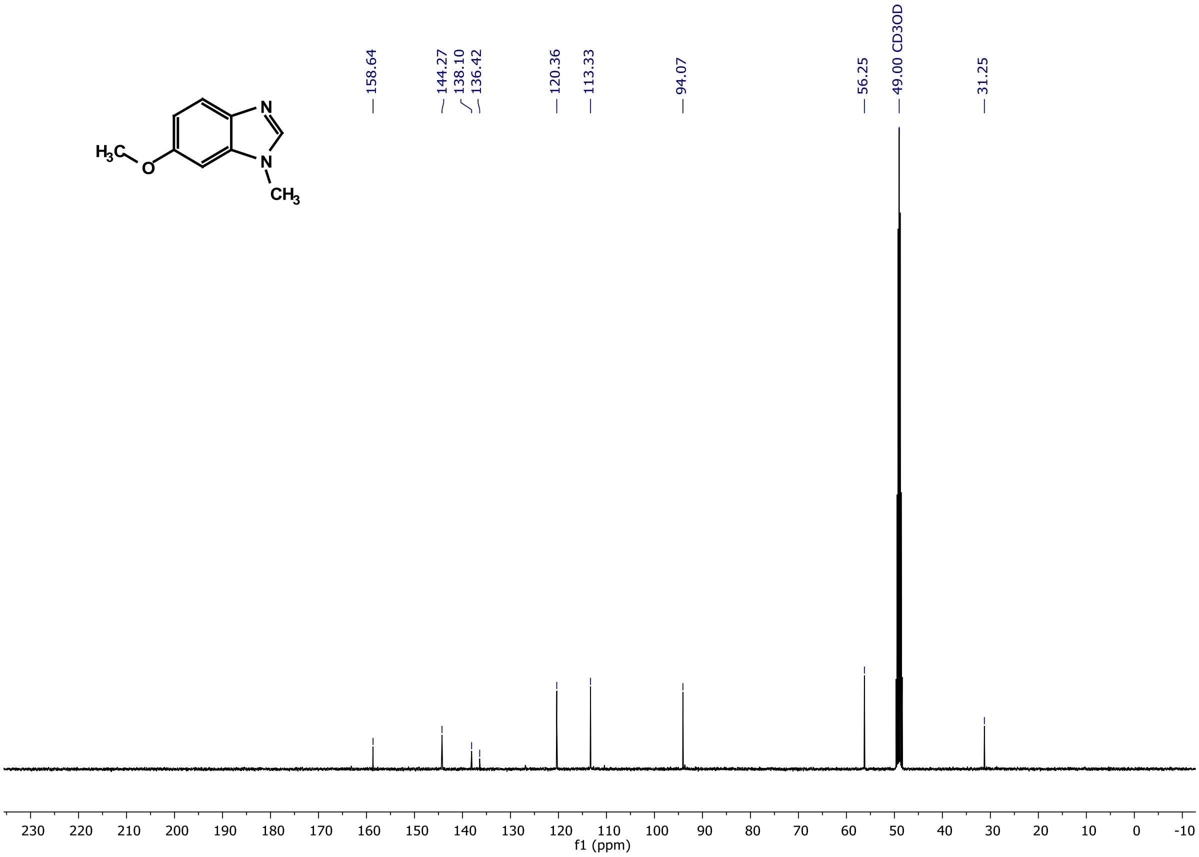

1-Ethyl-6-methoxy-1*H*-benzo[*d*]imidazole (**2c**)

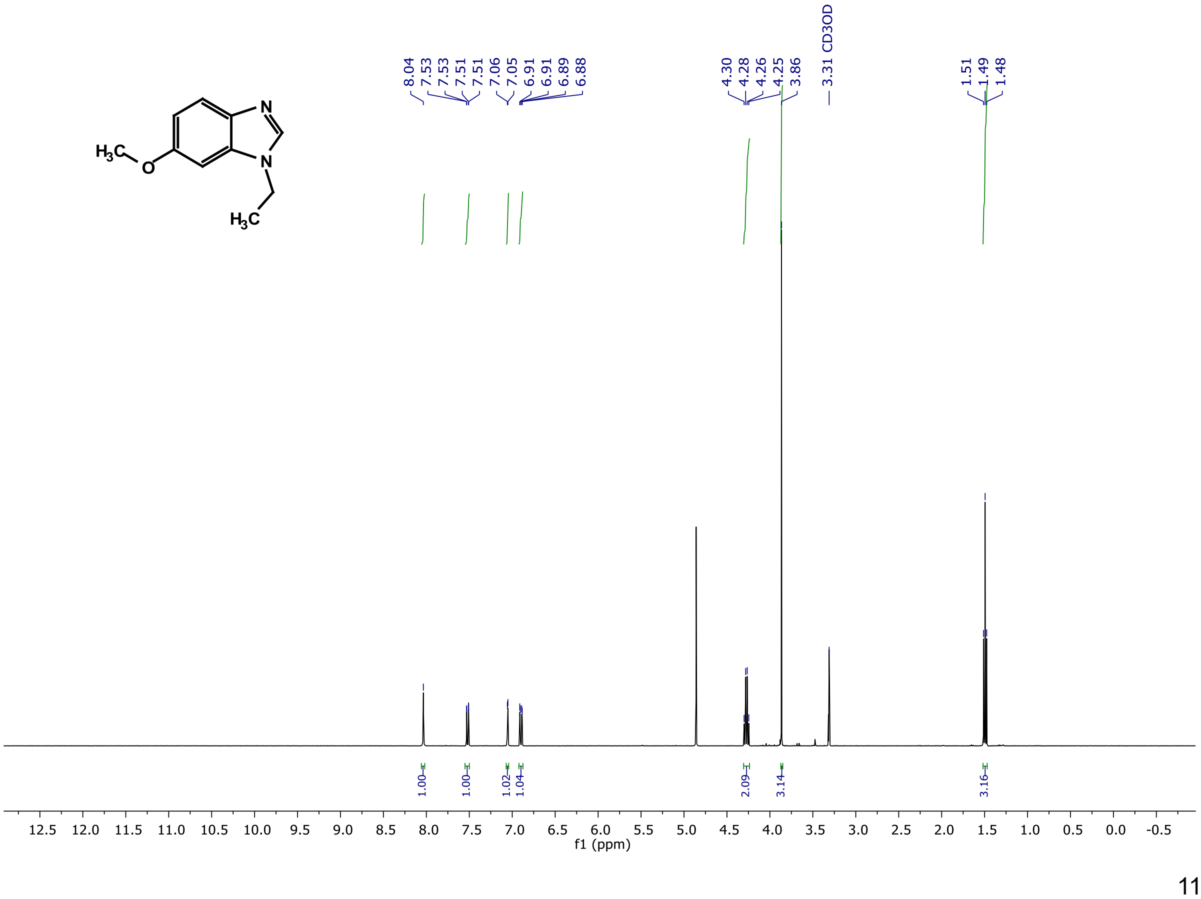

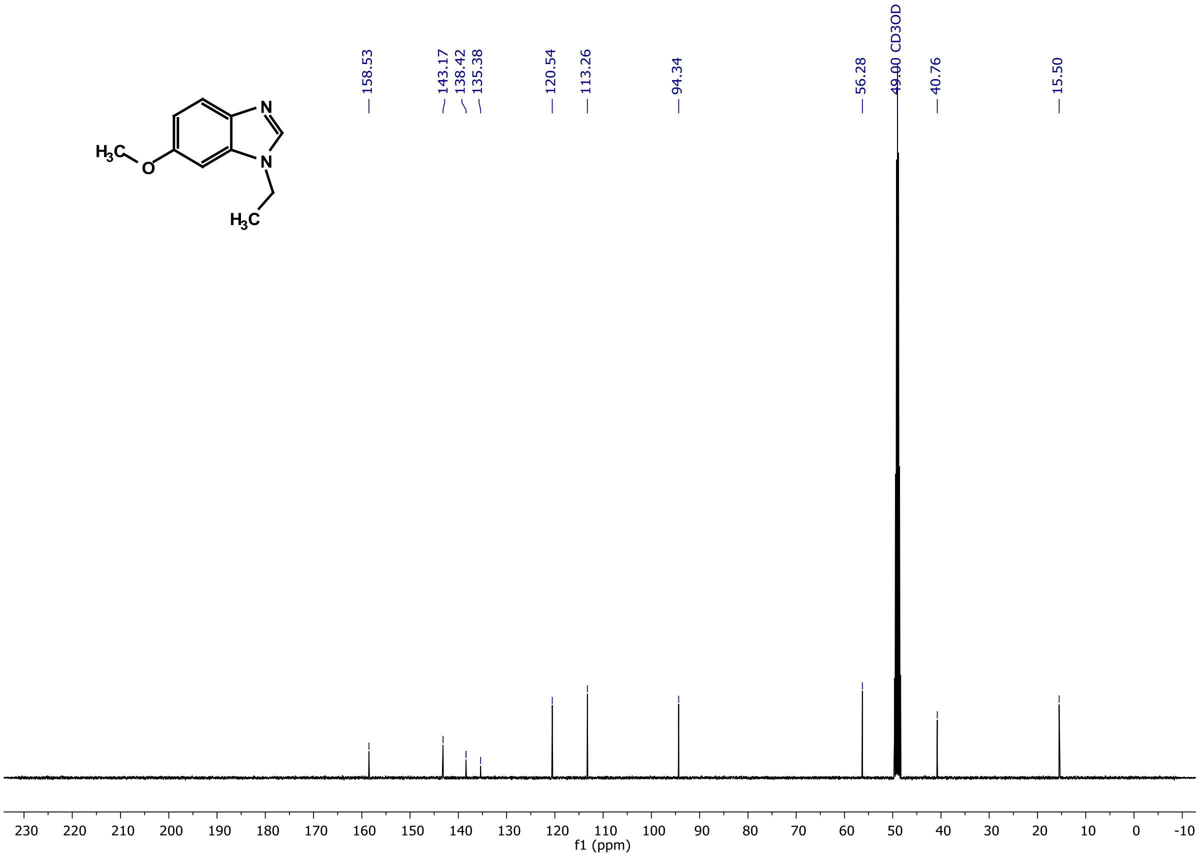

1-Isopropyl-6-methoxy-1*H*-benzo[*d*]imidazole (**2d**)

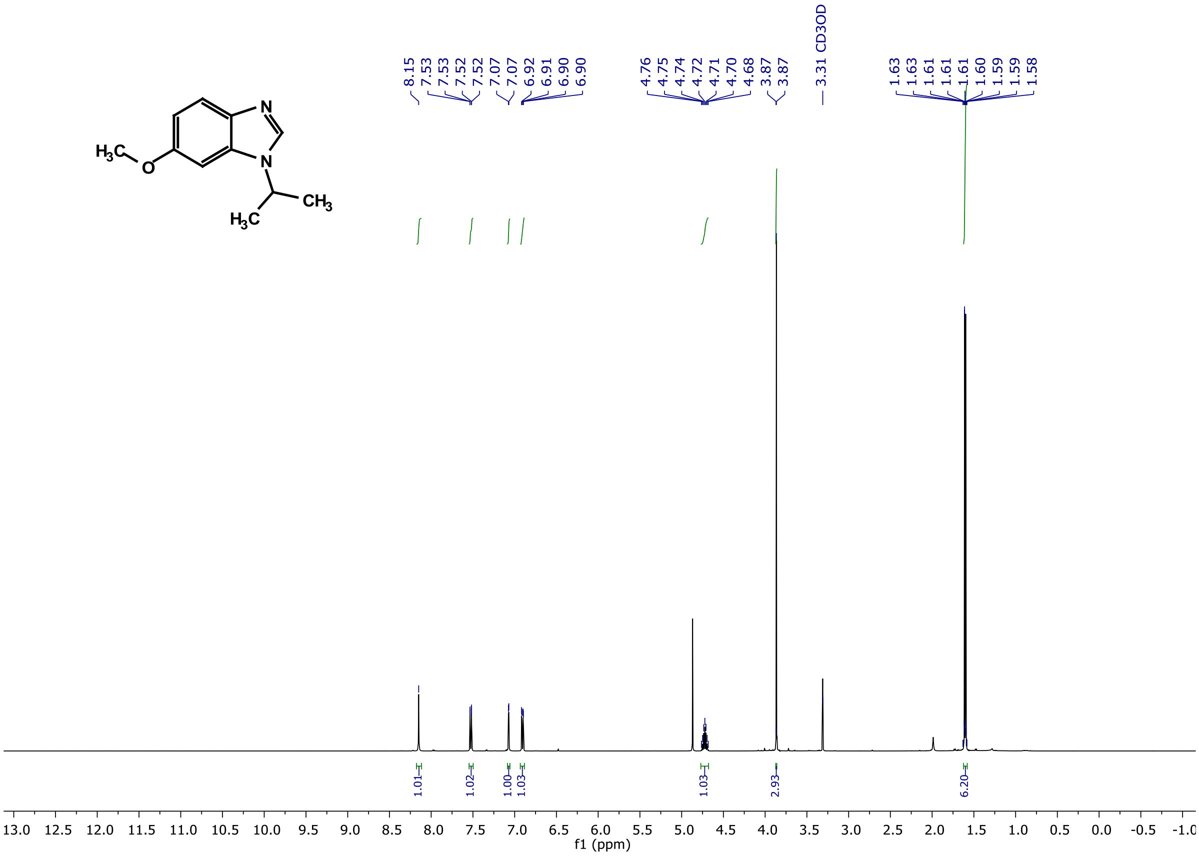

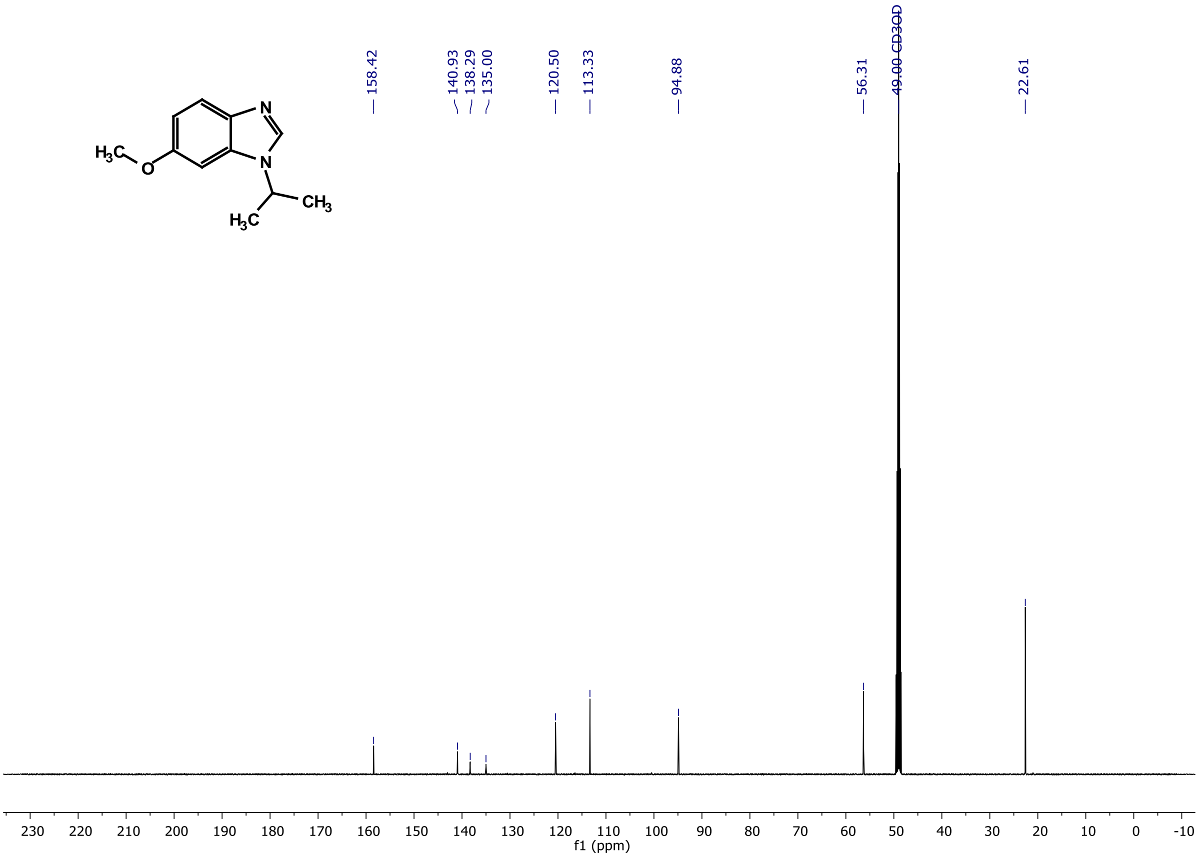

1-Allyl-6-methoxy-1*H*-benzo[*d*]imidazole (**2e**)

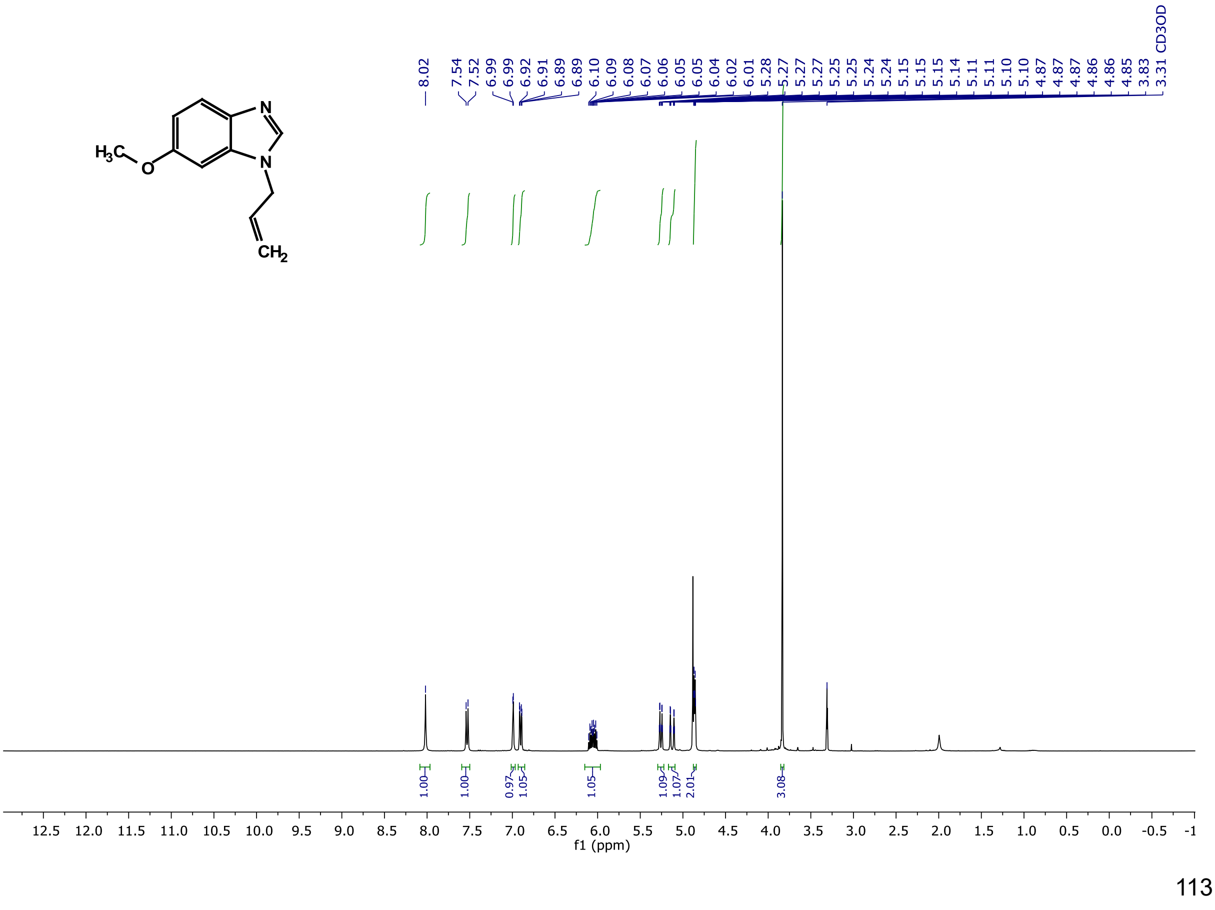

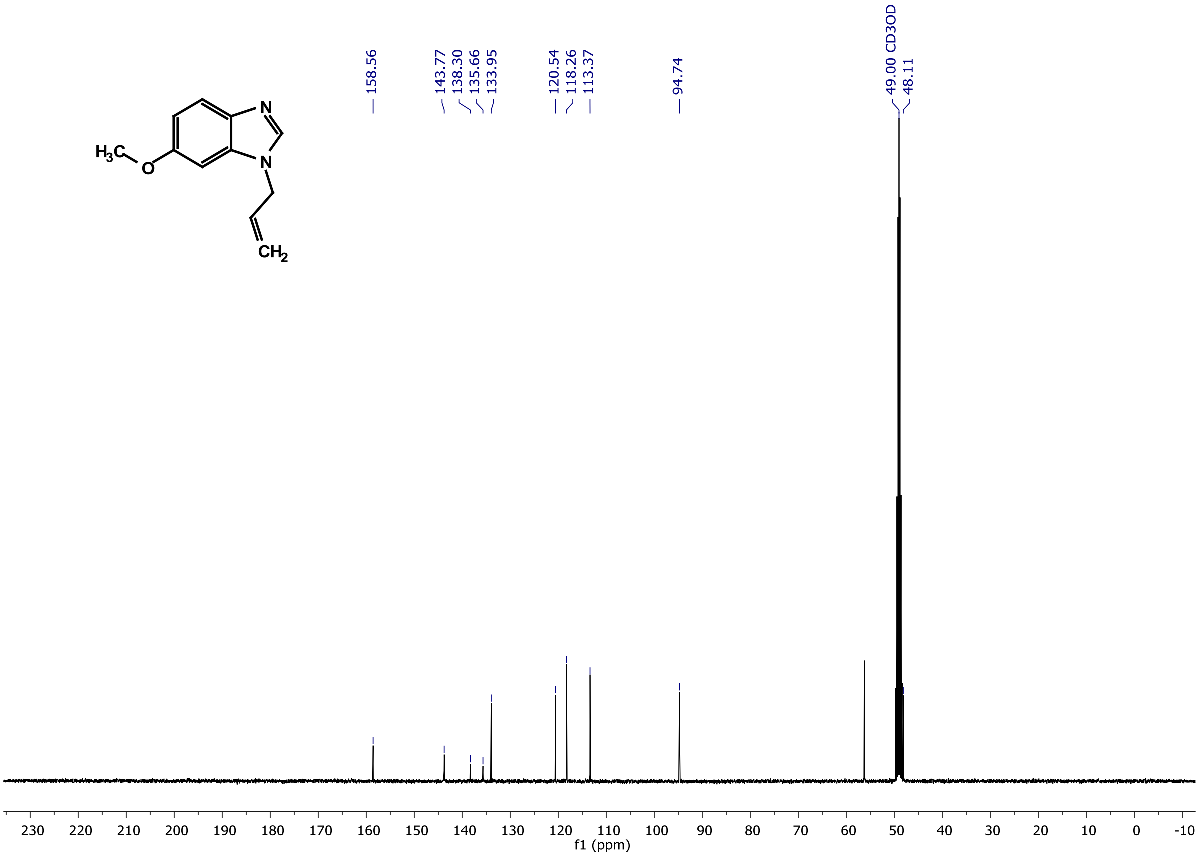

1-Benzyl-6-Methoxy-1*H*-benzo[*d*]imidazole (**2f**)

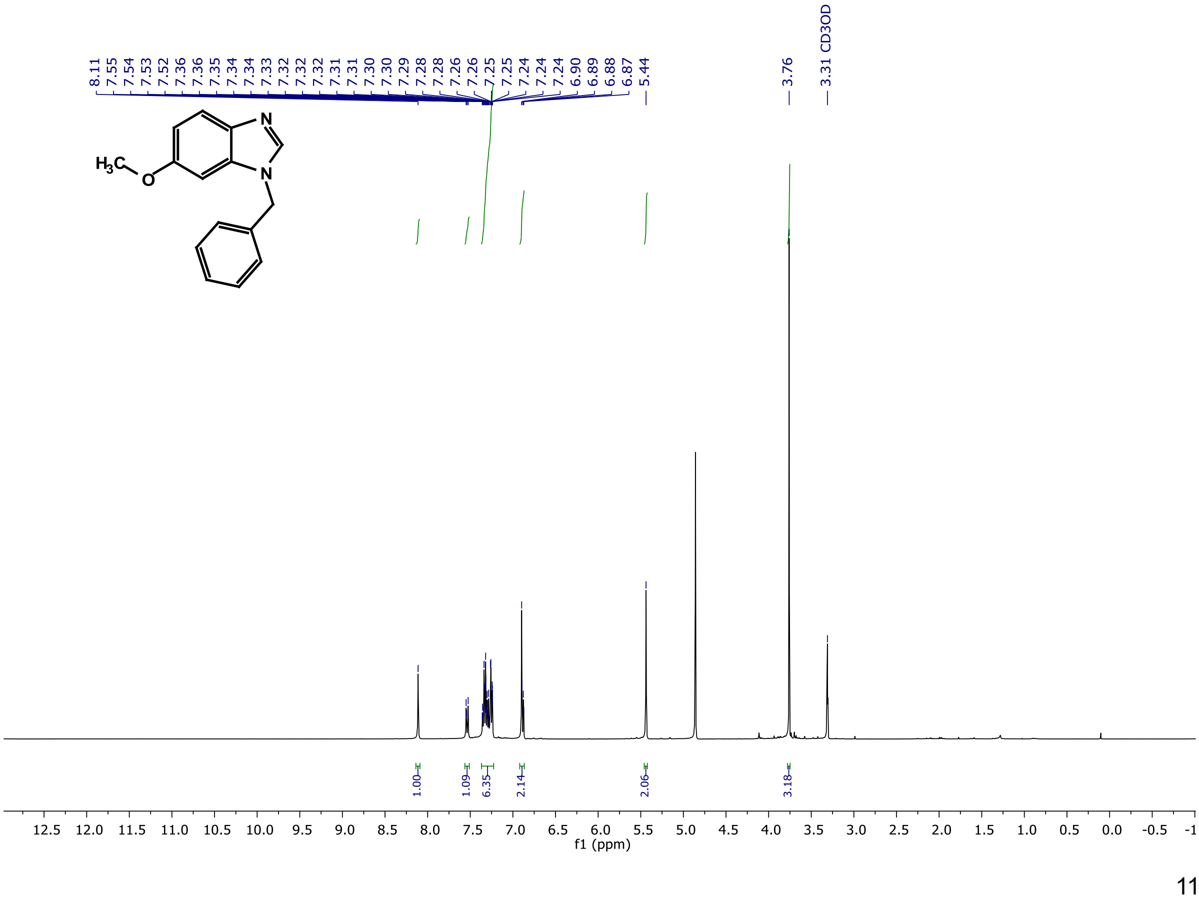

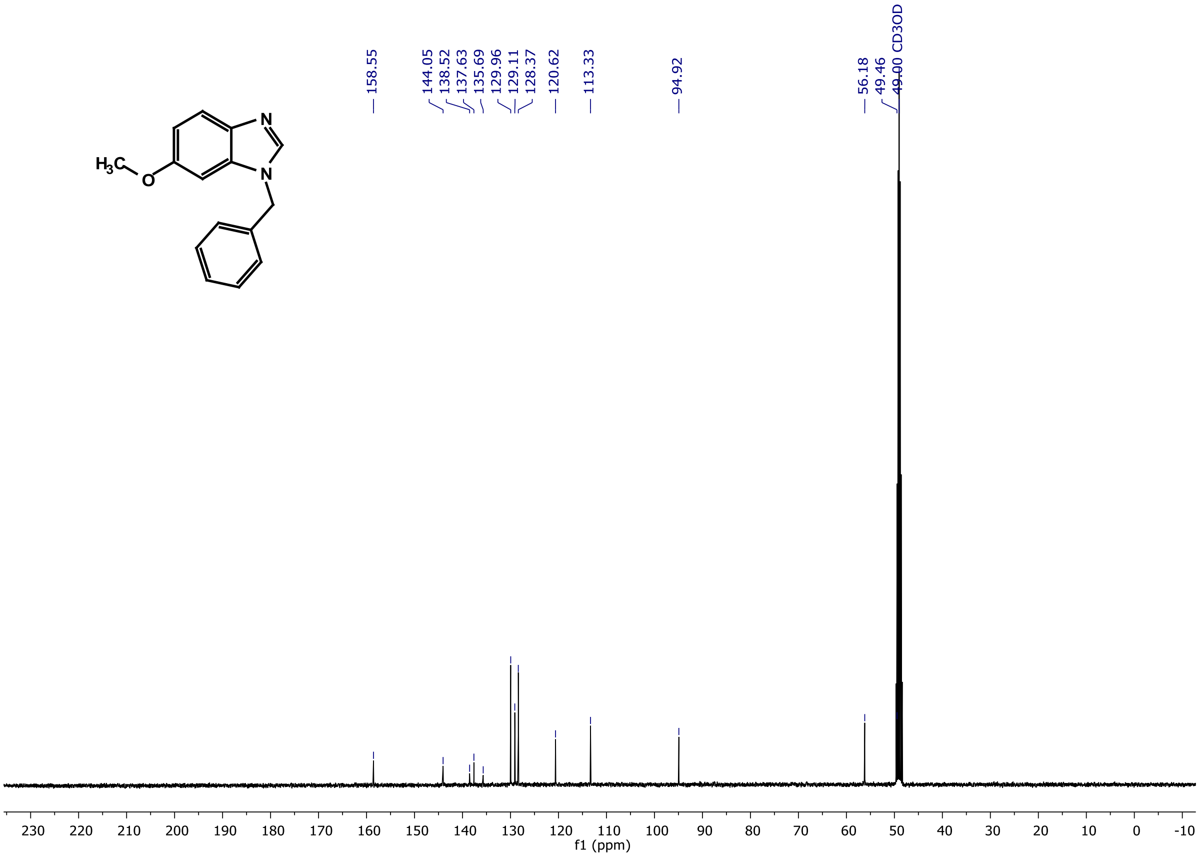

1-Benzyl-6-fluoro-1*H*-benzo[*d*]imidazole (**2g**)

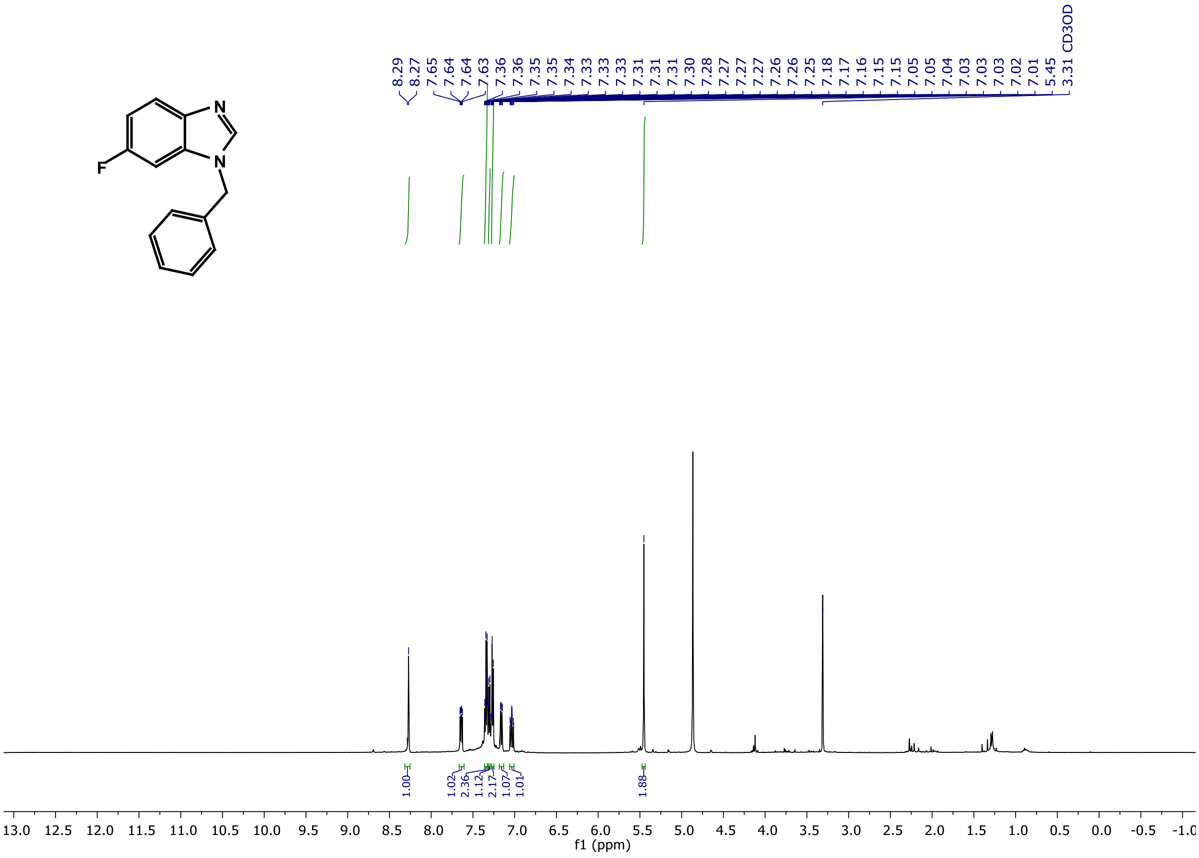

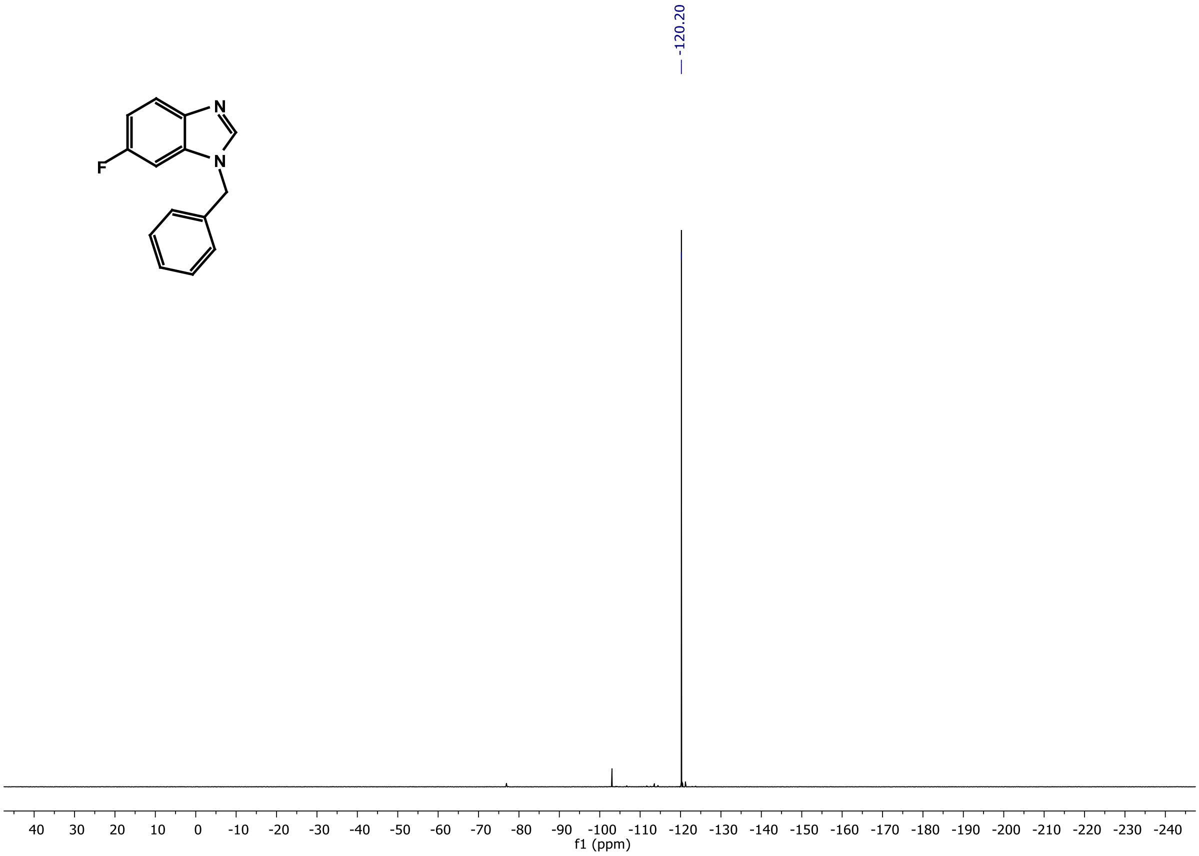
-120.20
N
N
F
40 30 20 10 0 -10 -20 -30 -40 -50 -60 -70 -80 -90 -100 -110 -120 -130 -140 -150 -160 -170 -180 -190 -200 -210 -220 -230 -240
f1 (ppm)

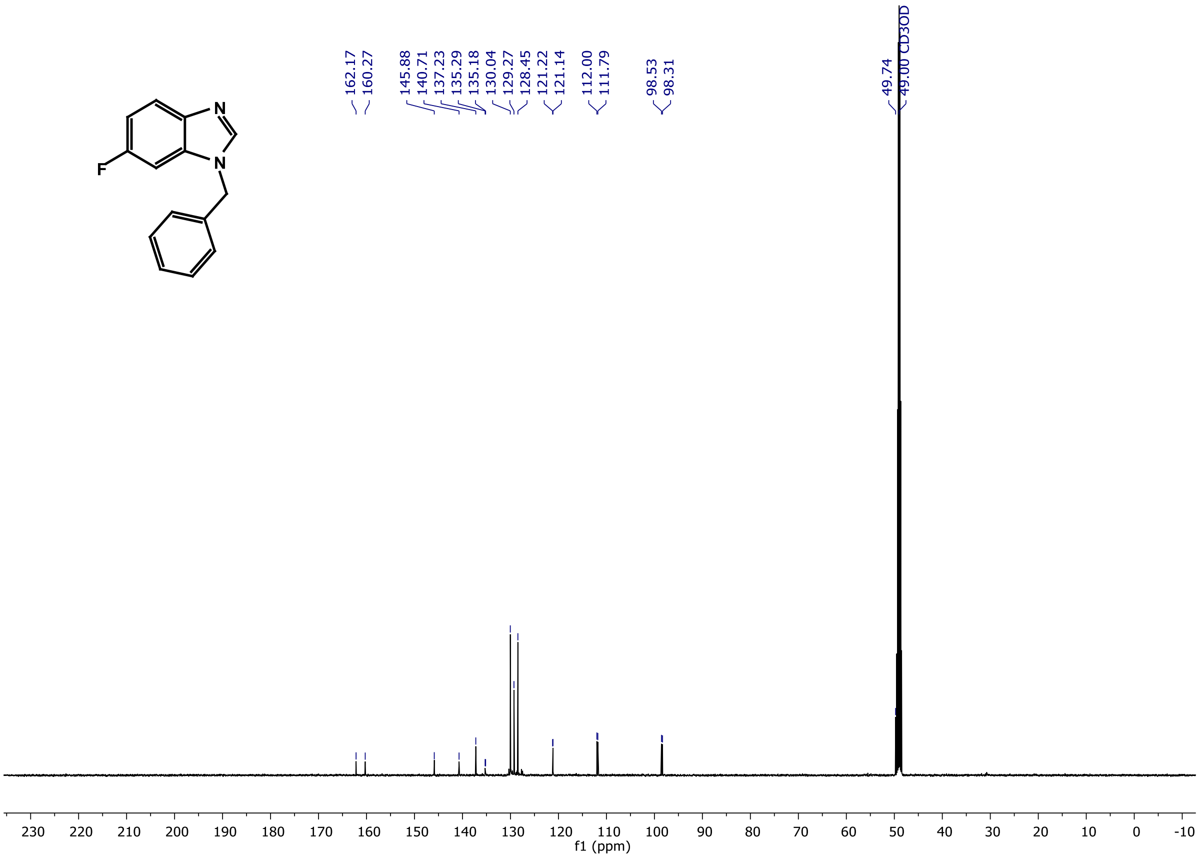
162.17
160.27
145.88
140.71
137.23
135.29
135.18
130.04
129.27
128.45
121.22
121.14
112.00
111.79
98.53
98.31
49.74
49.00 CD3OD
N
N
F
230 220 210 200 190 180 170 160 150 140 130 120 110 100 90 80 70 60 50 40 30 20 10 0 -10
f1 (ppm)

1-Benzyl-1*H*-benzo[*d*]imidazole (**SI-7**)

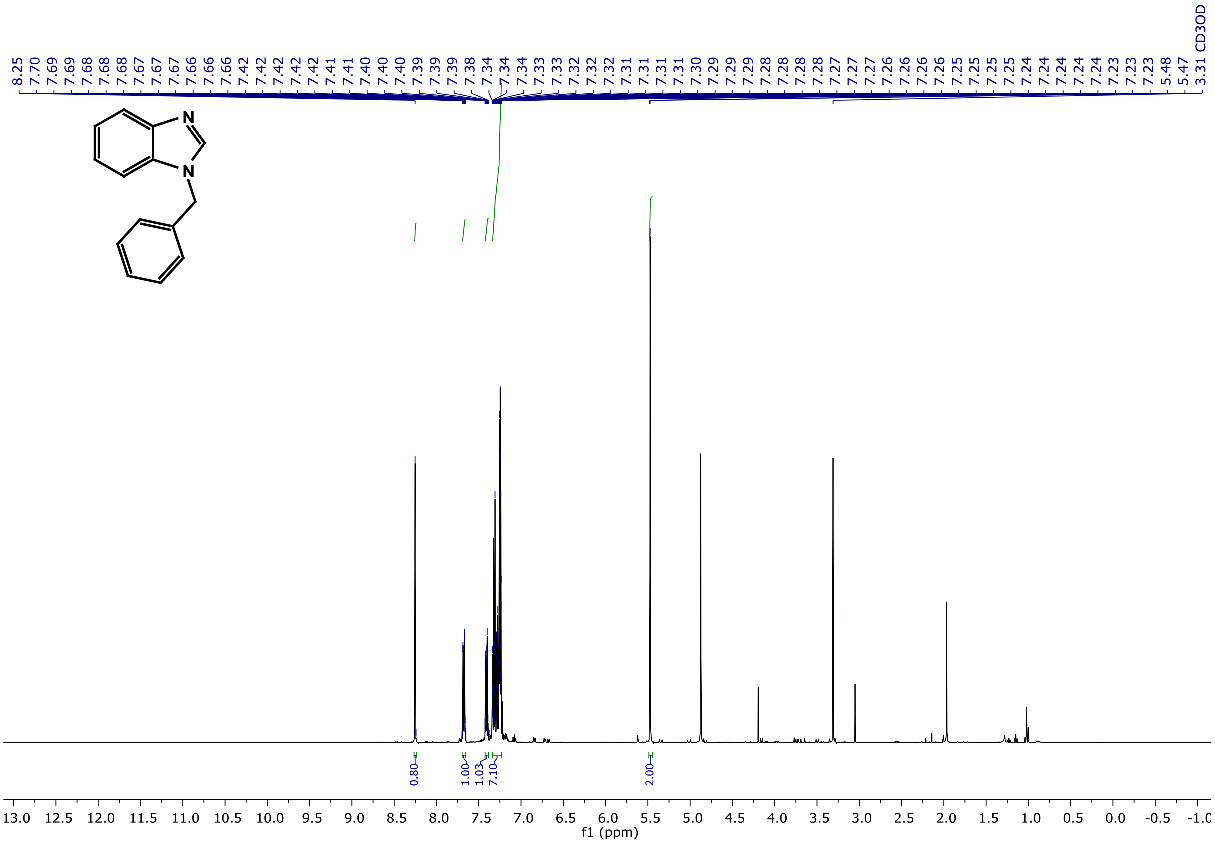

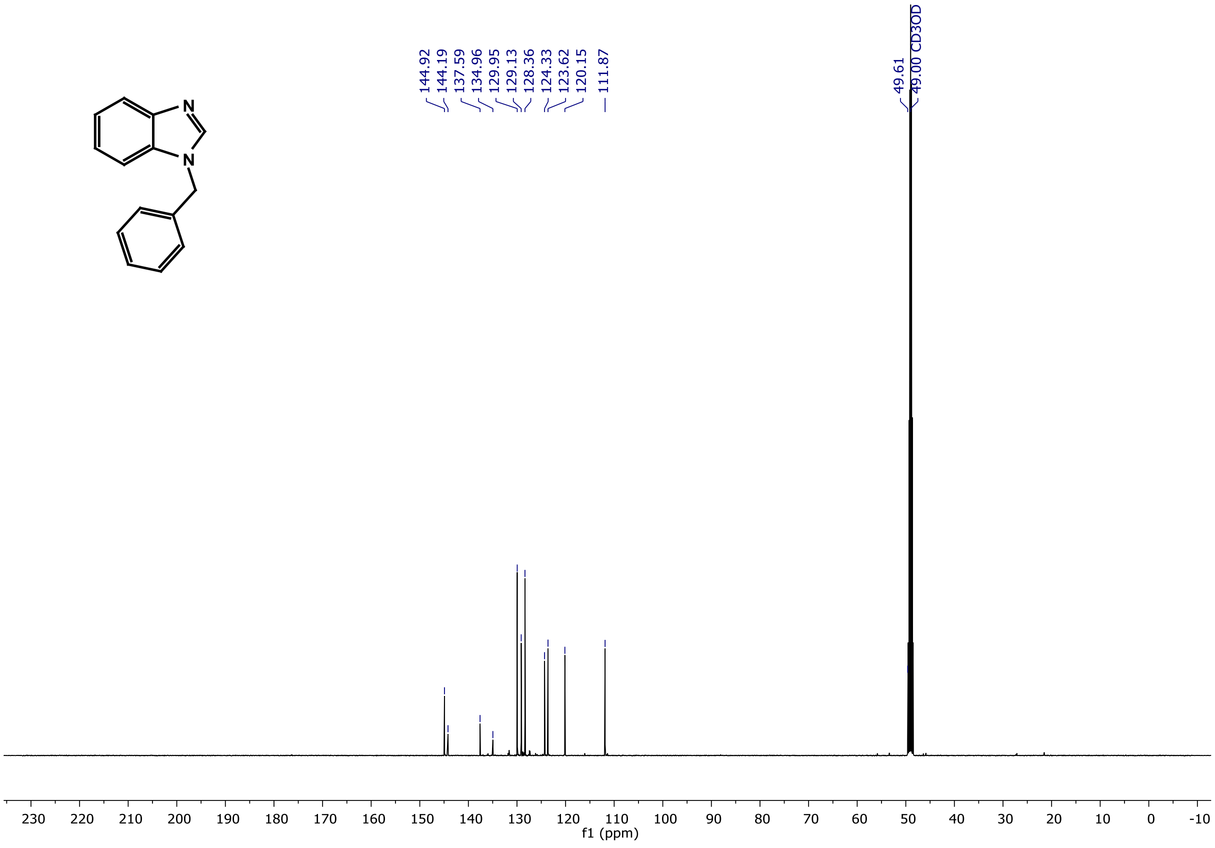

1*H*-benzo[*d*]imidazole (**2h**)

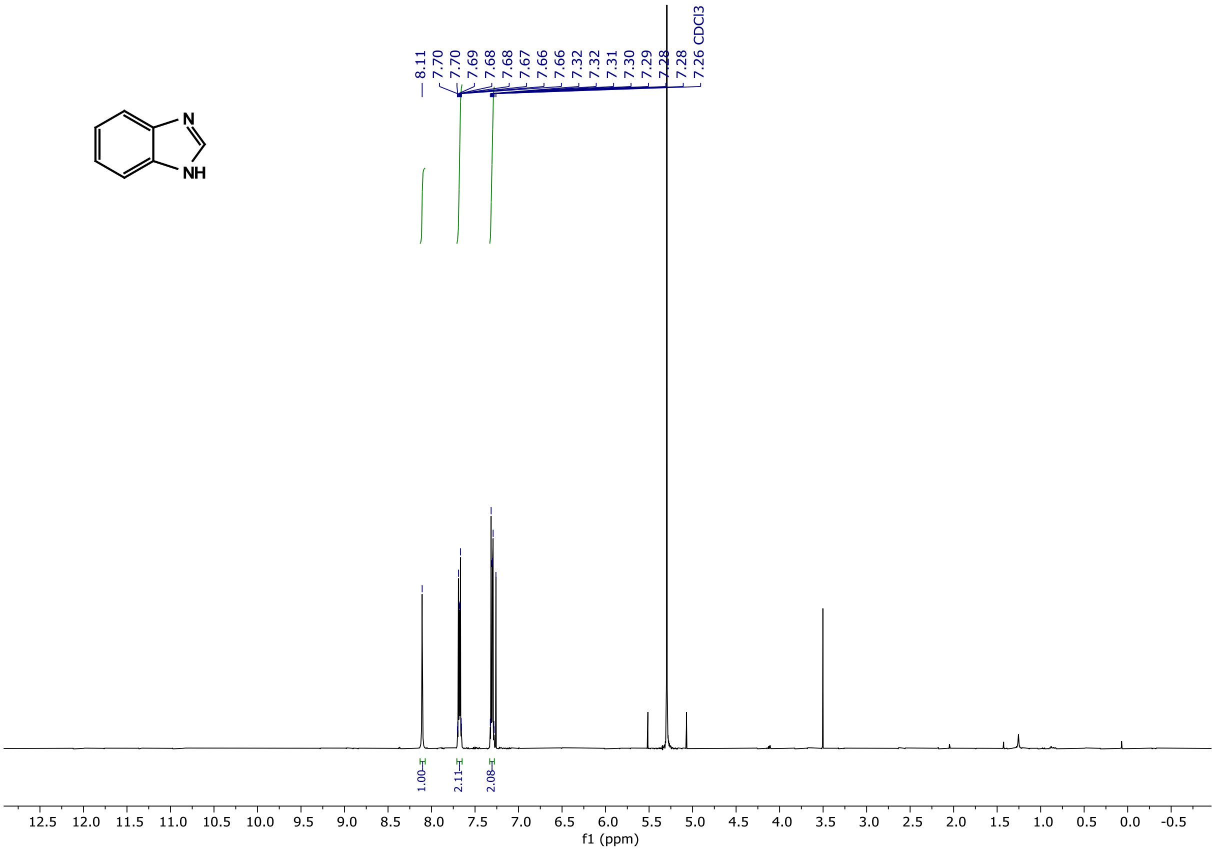

6-Fluoro-1-methyl-1*H*-benzo[*d*]imidazole (**2i**)

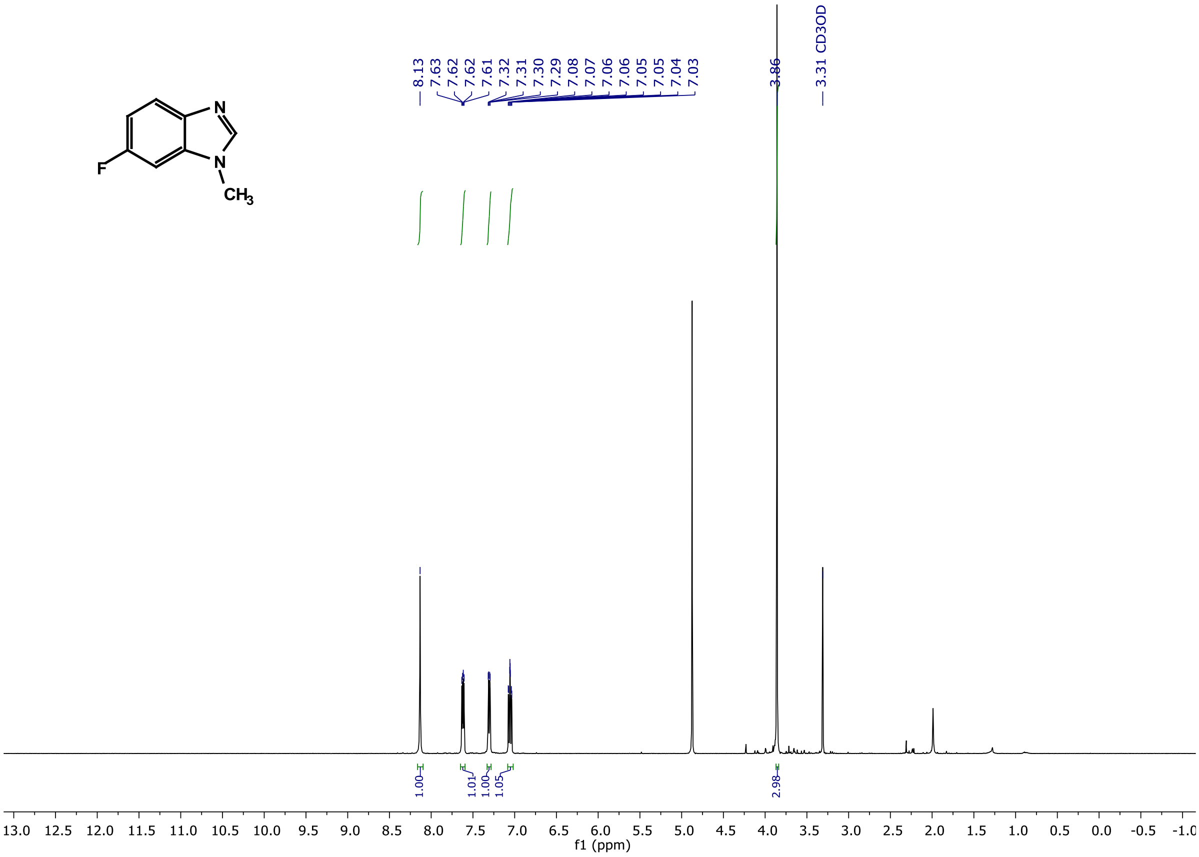

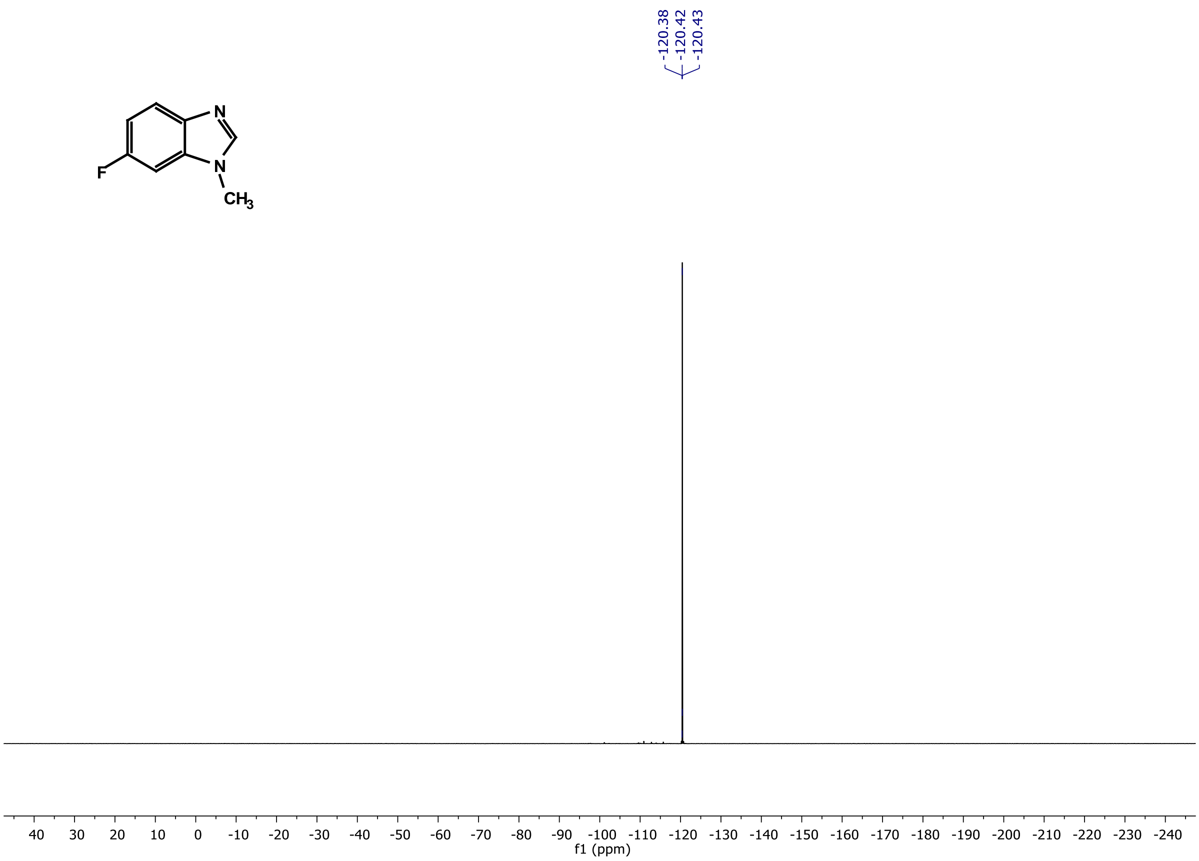

-120.38
-120.42
-120.43
F
N
N
CH3
40 30 20 10 0 -10 -20 -30 -40 -50 -60 -70 -80 -90 -100 -110 -120 -130 -140 -150 -160 -170 -180 -190 -200 -210 -220 -230 -240
f1 (ppm)

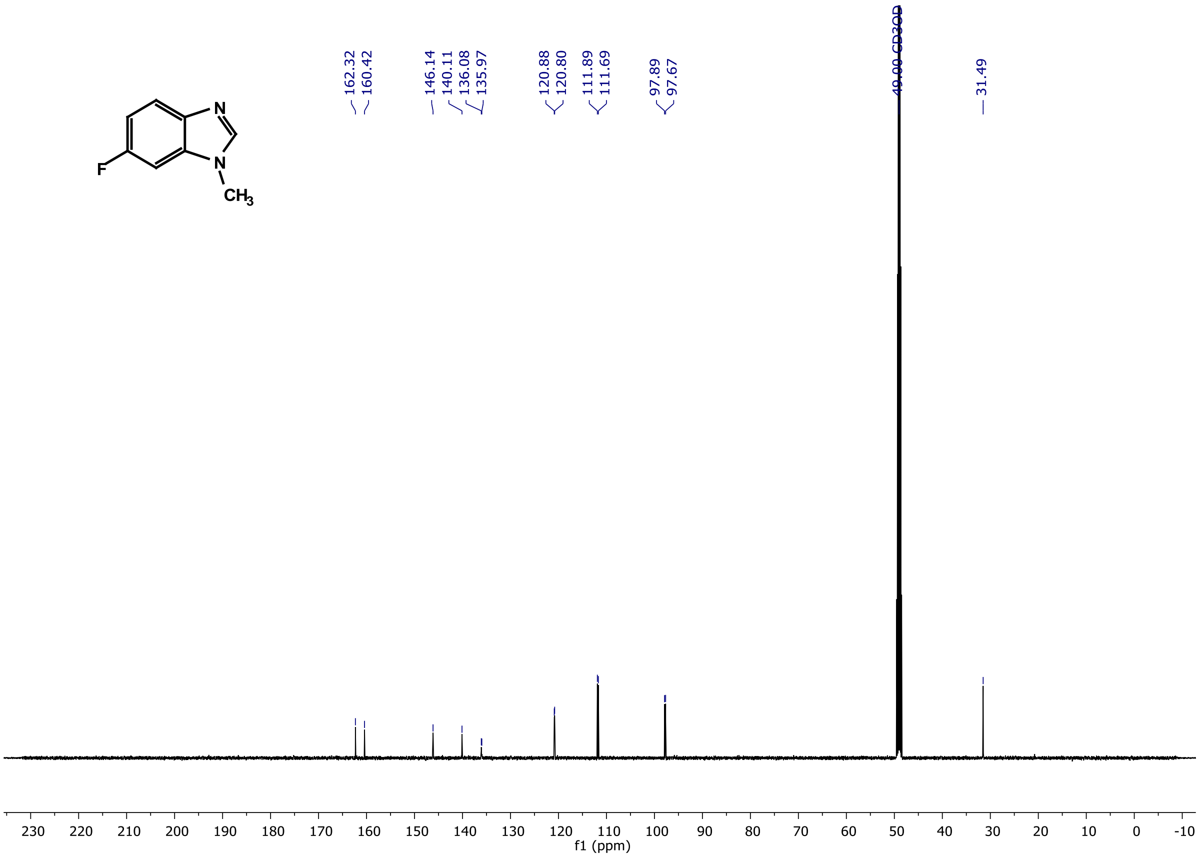

162.32
160.42
146.14
140.11
136.08
135.97
120.88
120.80
111.89
111.69
97.89
97.67
49.00 CD3OD
31.49
F
N
N
CH3
230 220 210 200 190 180 170 160 150 140 130 120 110 100 90 80 70 60 50 40 30 20 10 0 -10
f1 (ppm)

6-Chloro-1-methyl-1*H*-benzo[*d*]imidazole (**2j**)

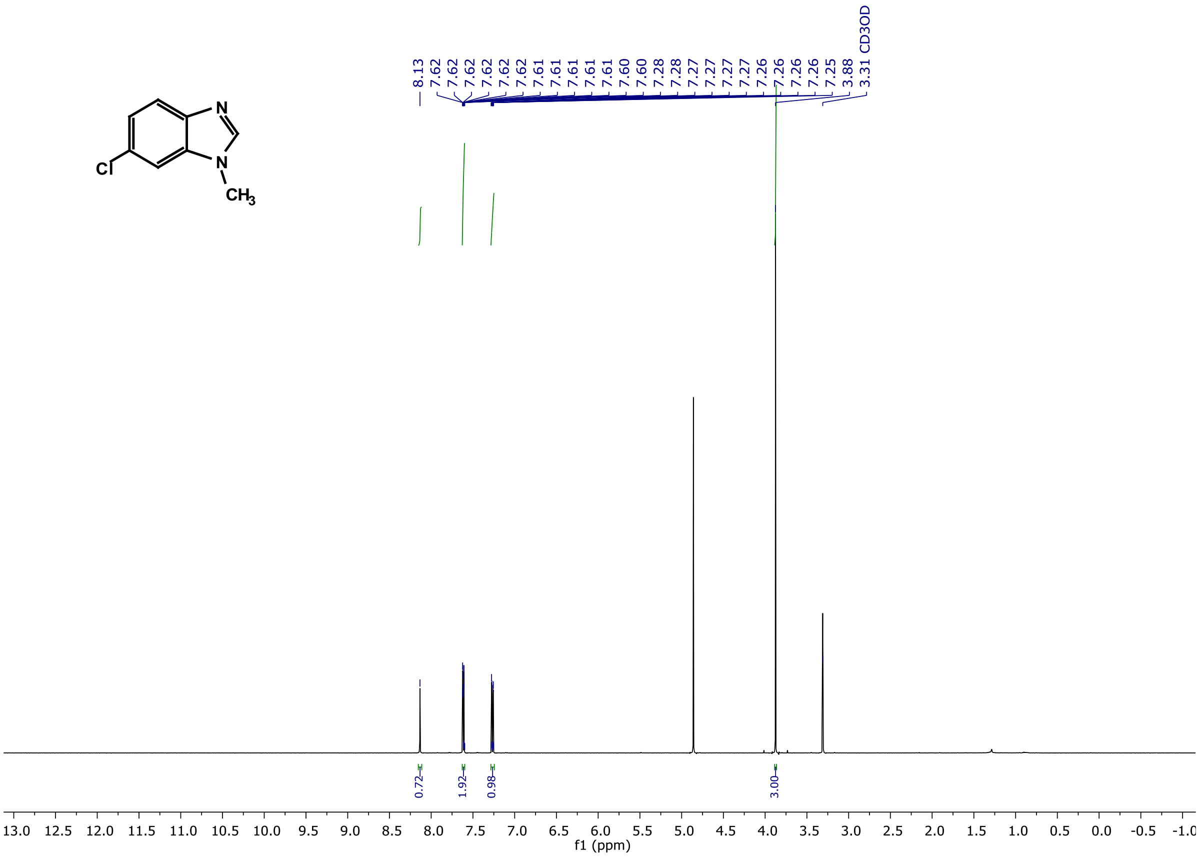

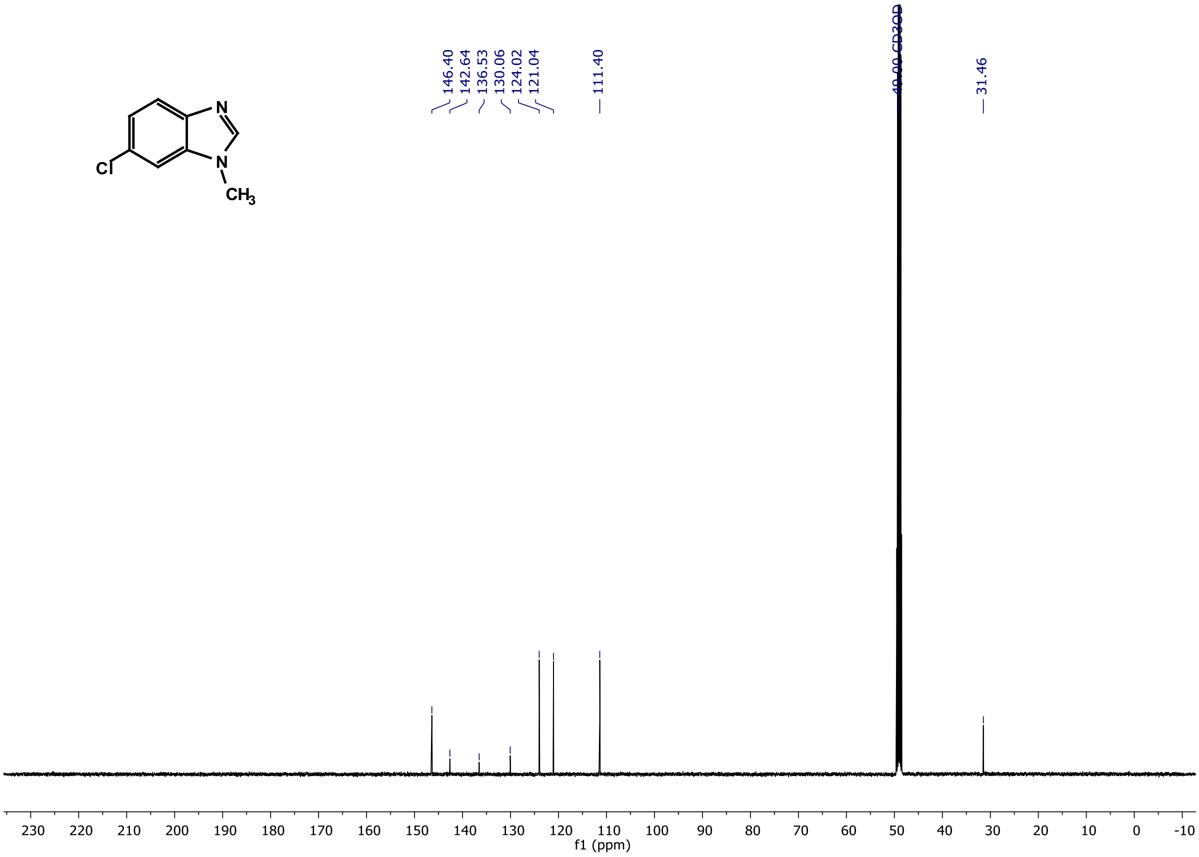

6-Bromo-1-methyl-1*H*-benzo[*d*]imidazole (**2k**)

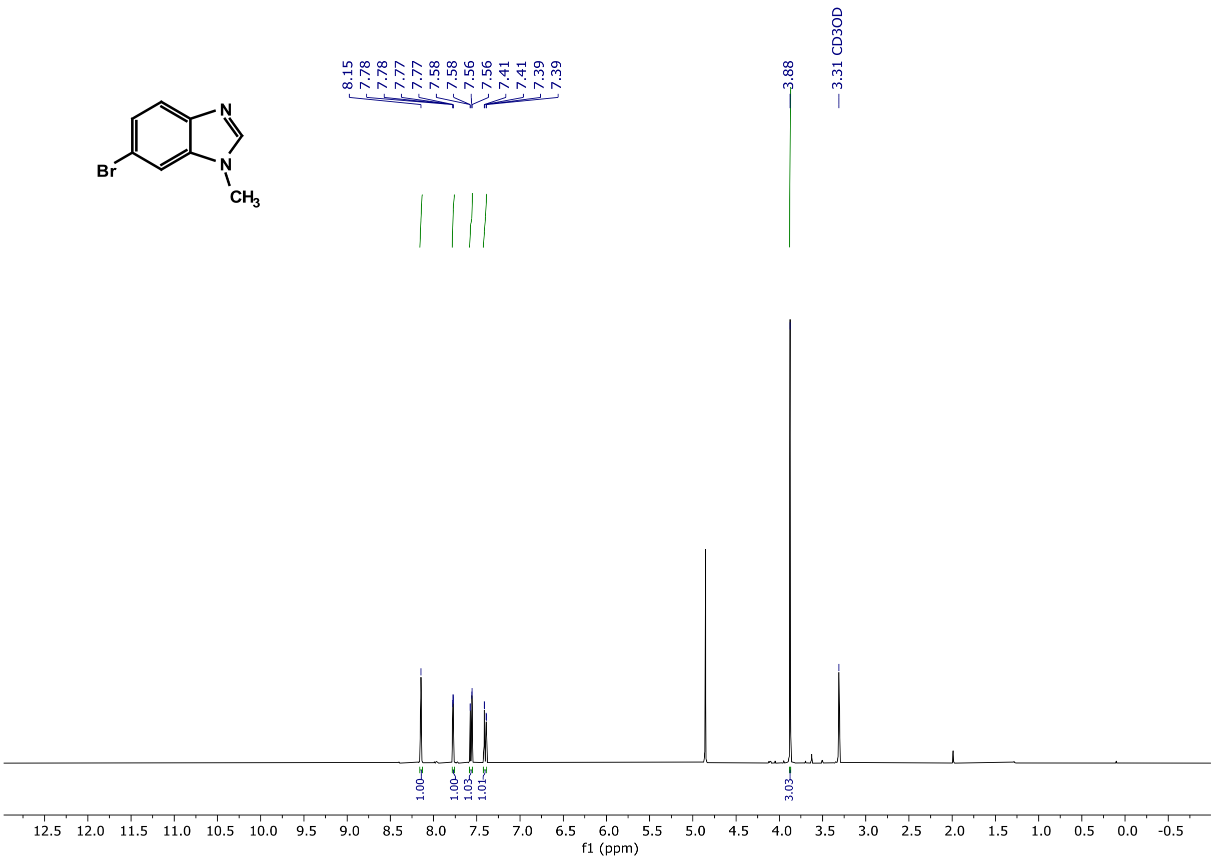

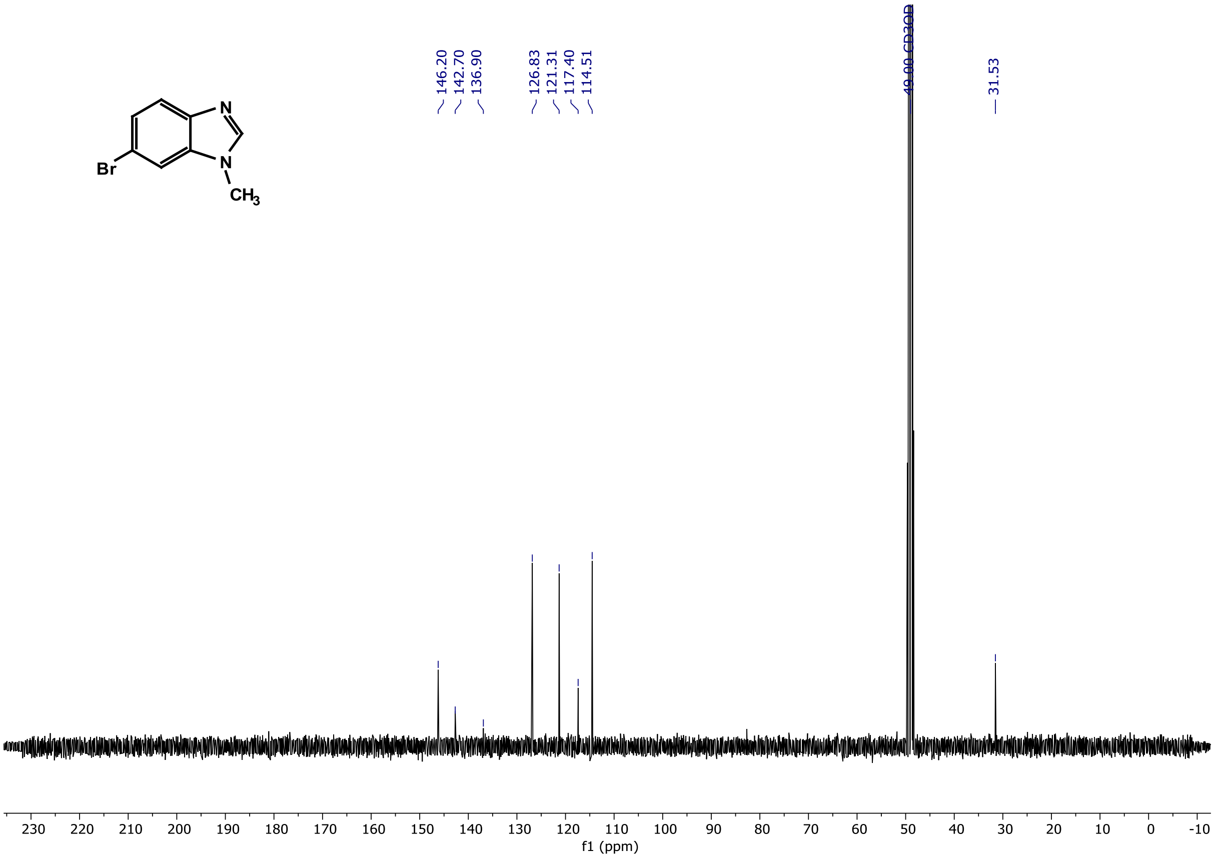

Methyl 1-methyl-1*H*-benzo[*d*]imidazole-6-carboxylate (**2l**)

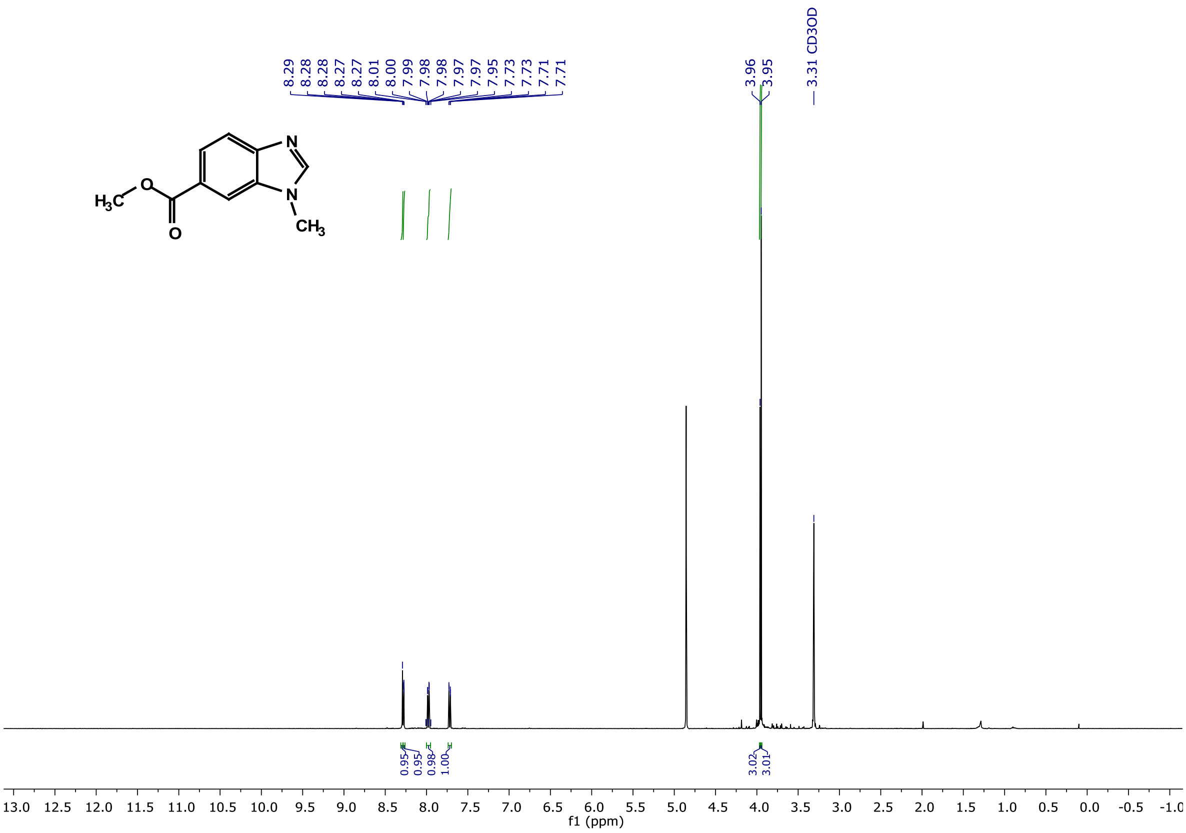

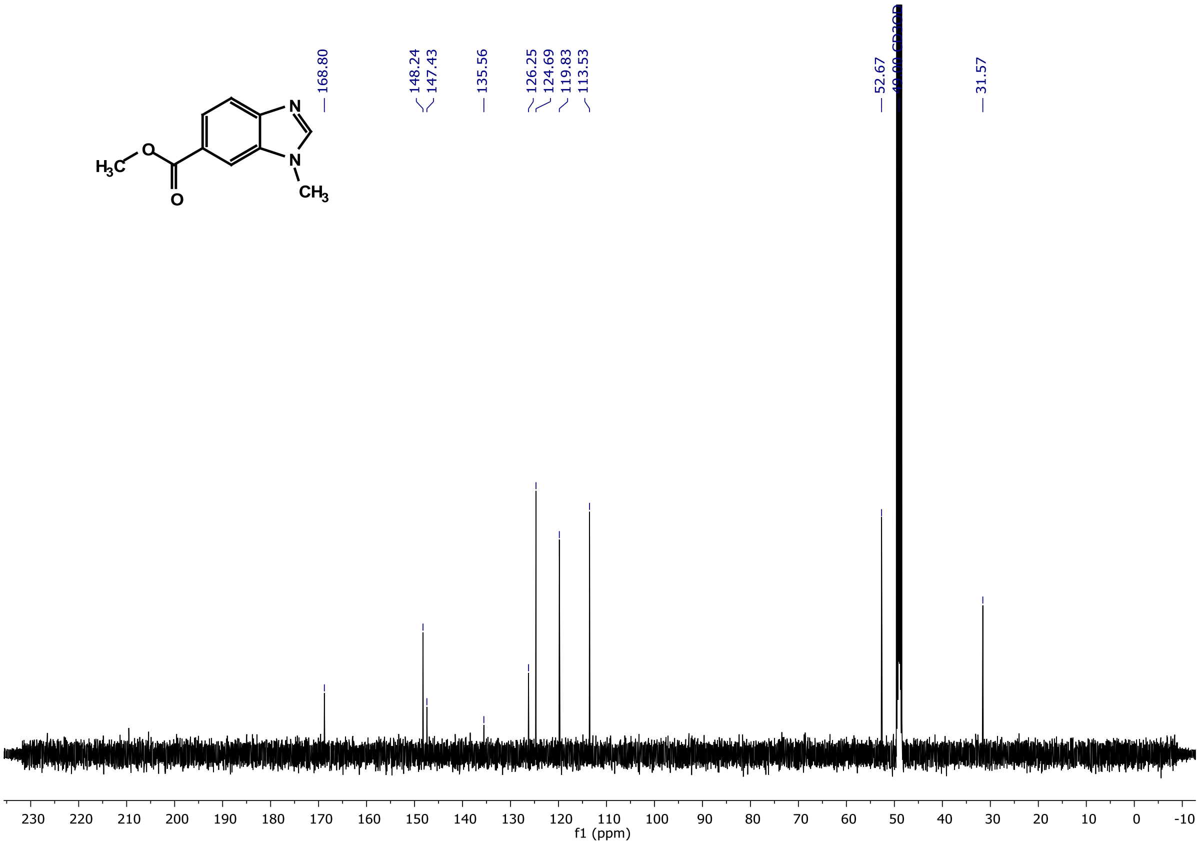

6-(Benzyloxy)-1-methyl-1*H*-benzo[*d*]imidazole (**2m**)

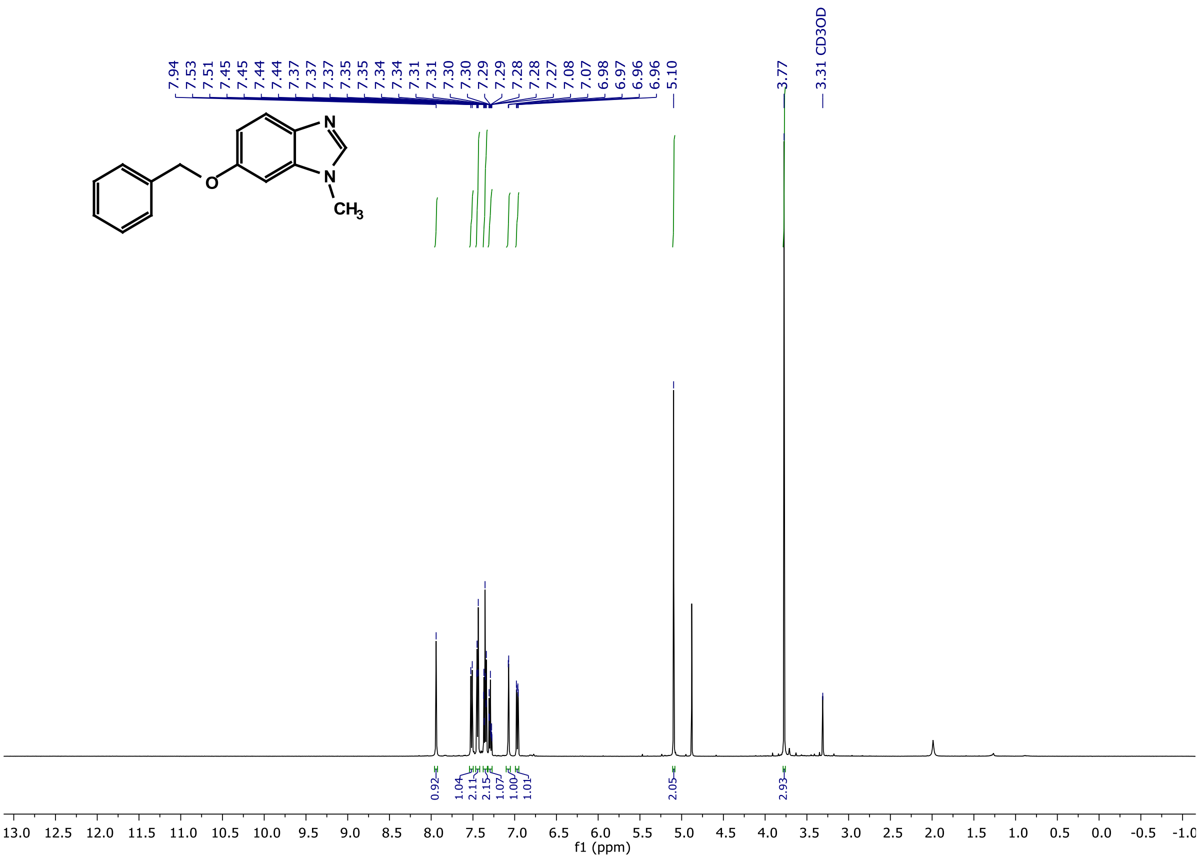

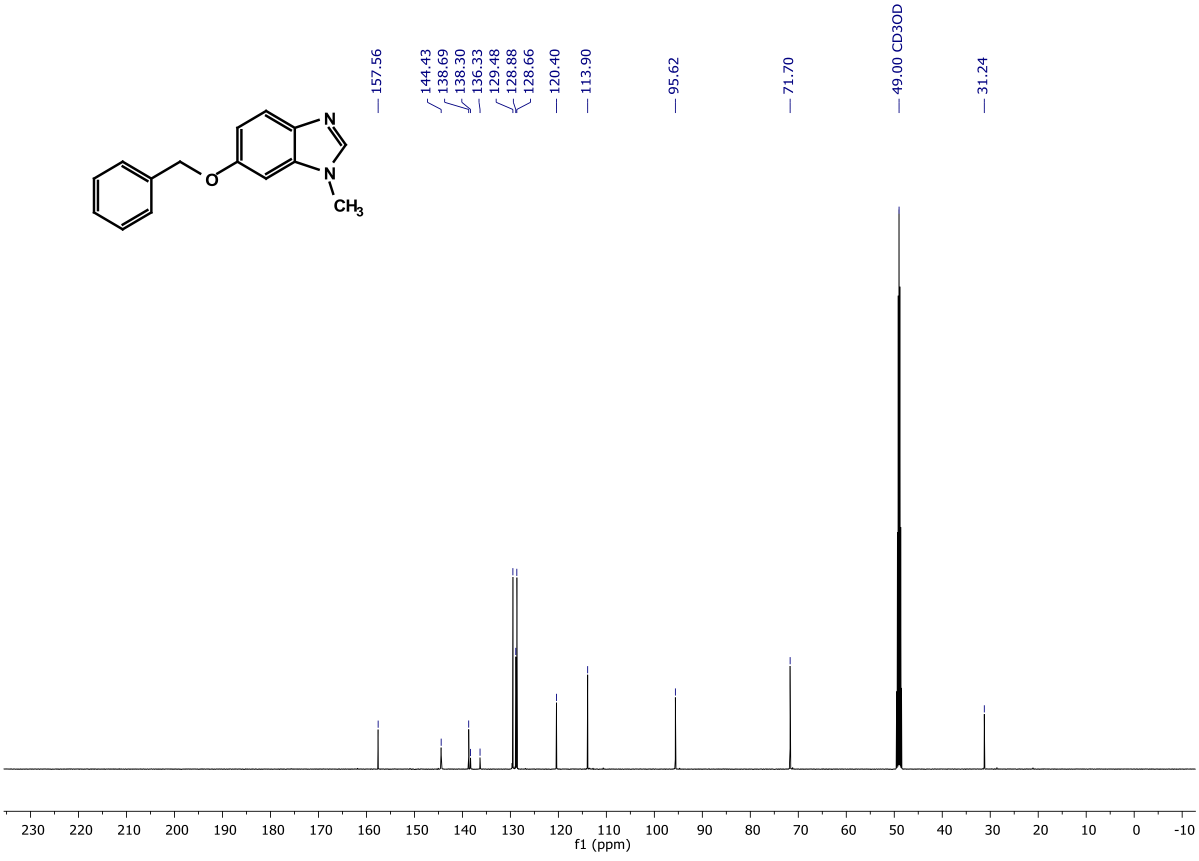

1-Methyl-6-(pyridin-2-yl)-1*H*-benzo[*d*]imidazole (**2n**)

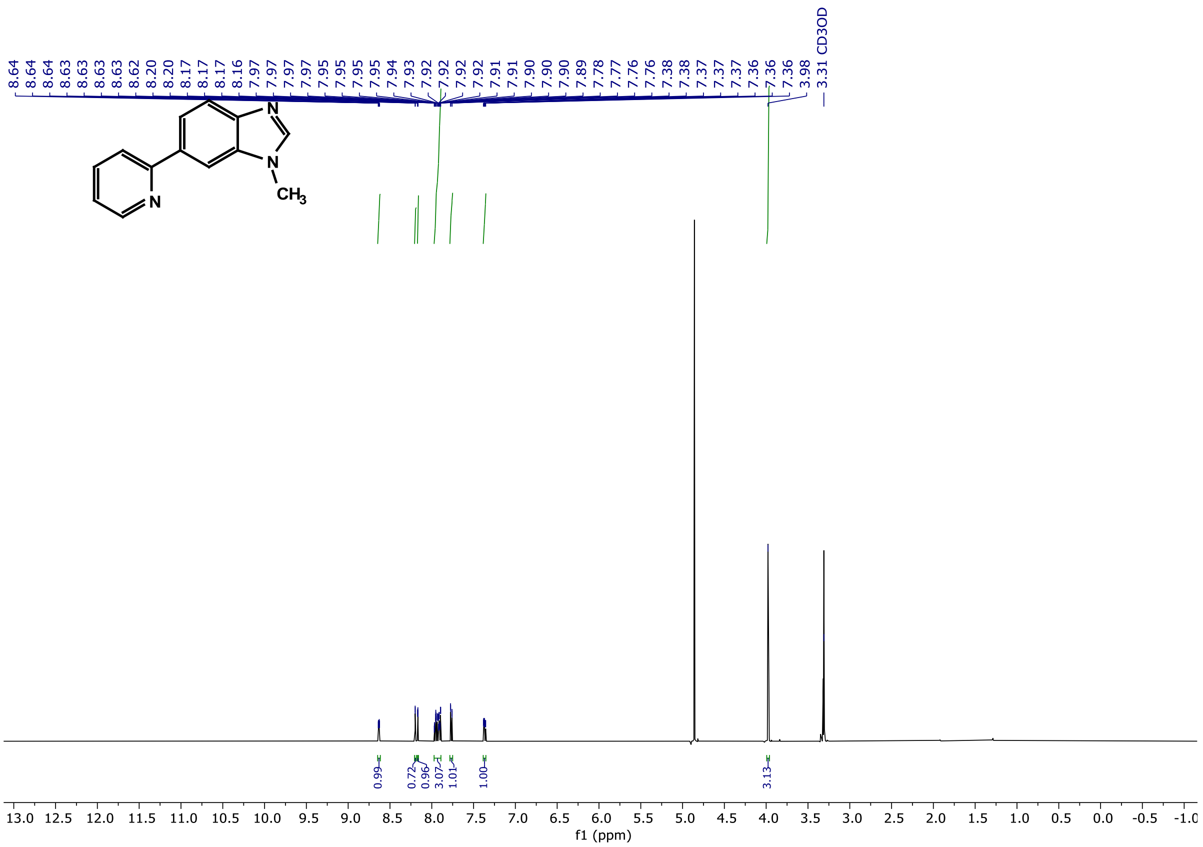

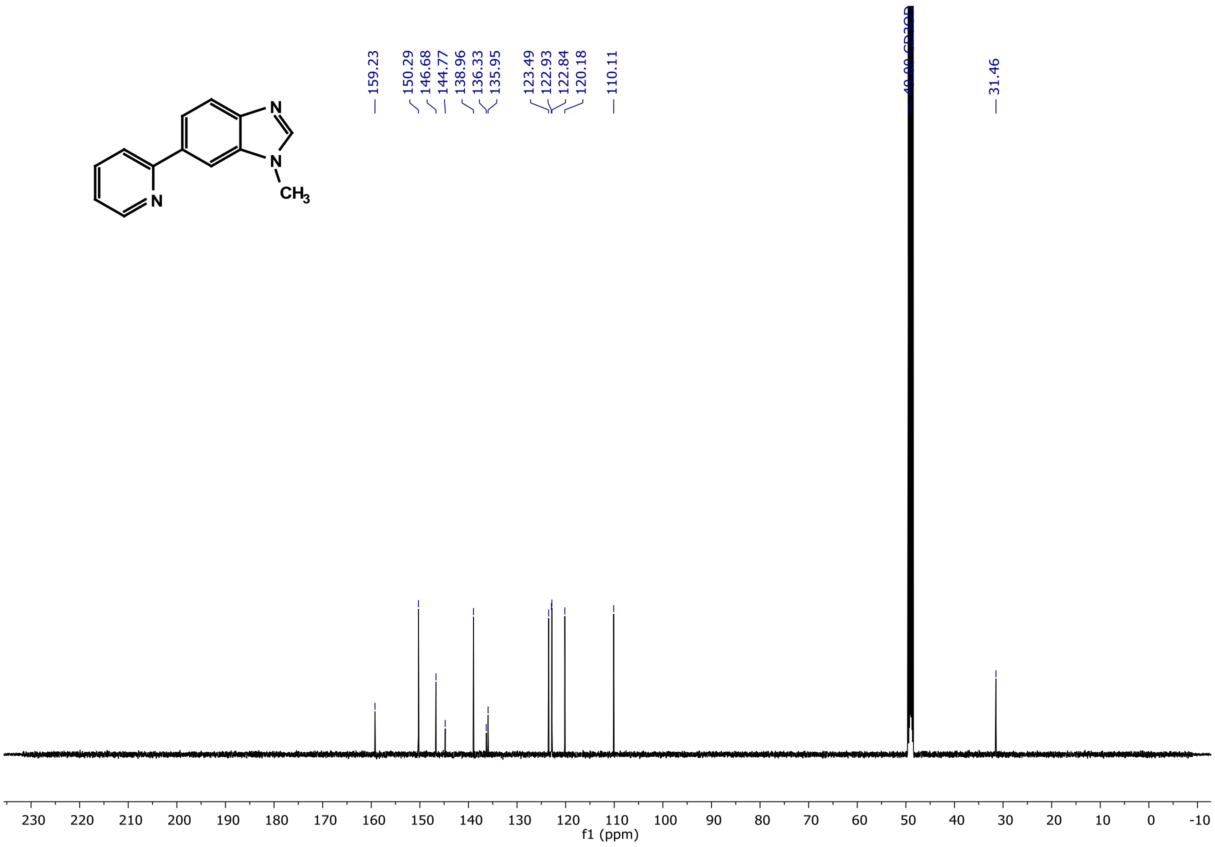

6-(6-Chloro-2-methylpyrimidin-4-yl)-1-methyl-1*H*-benzo[*d*]imidazole (**2o**)

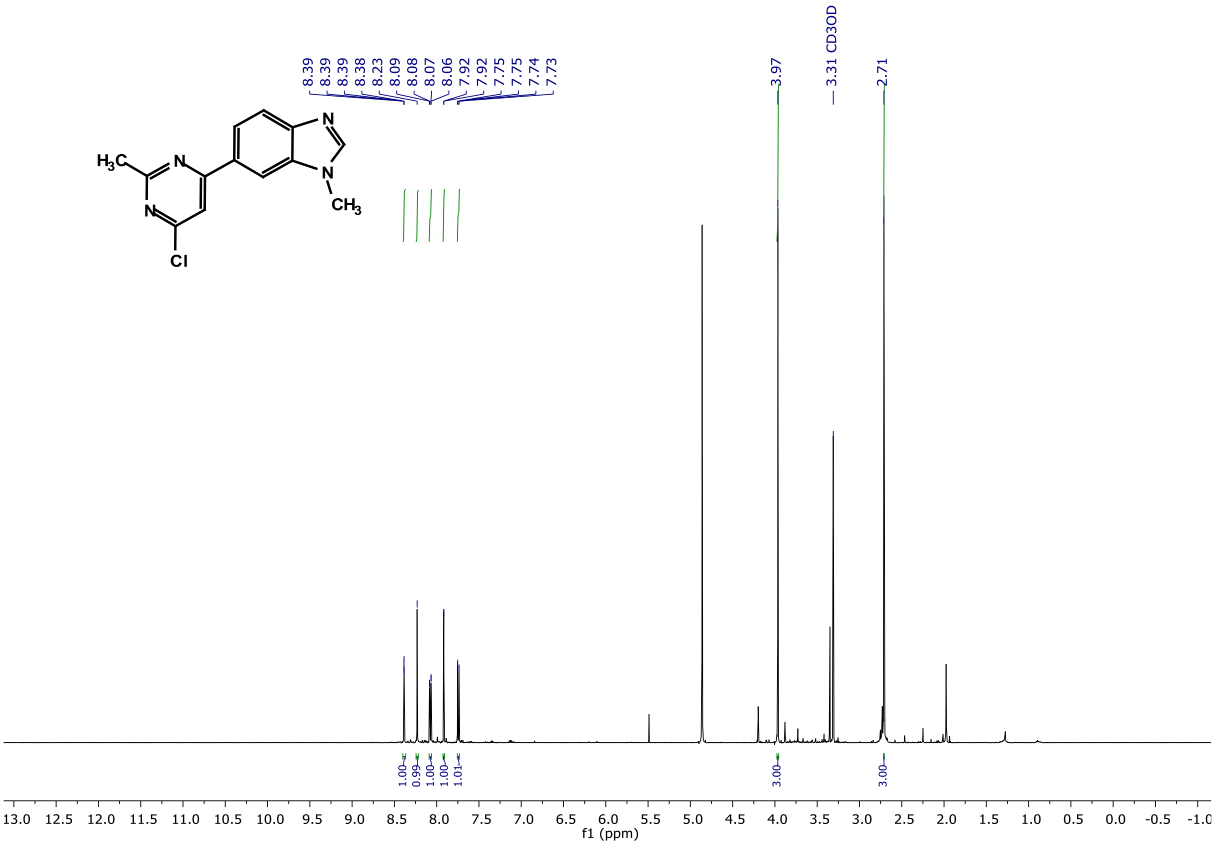

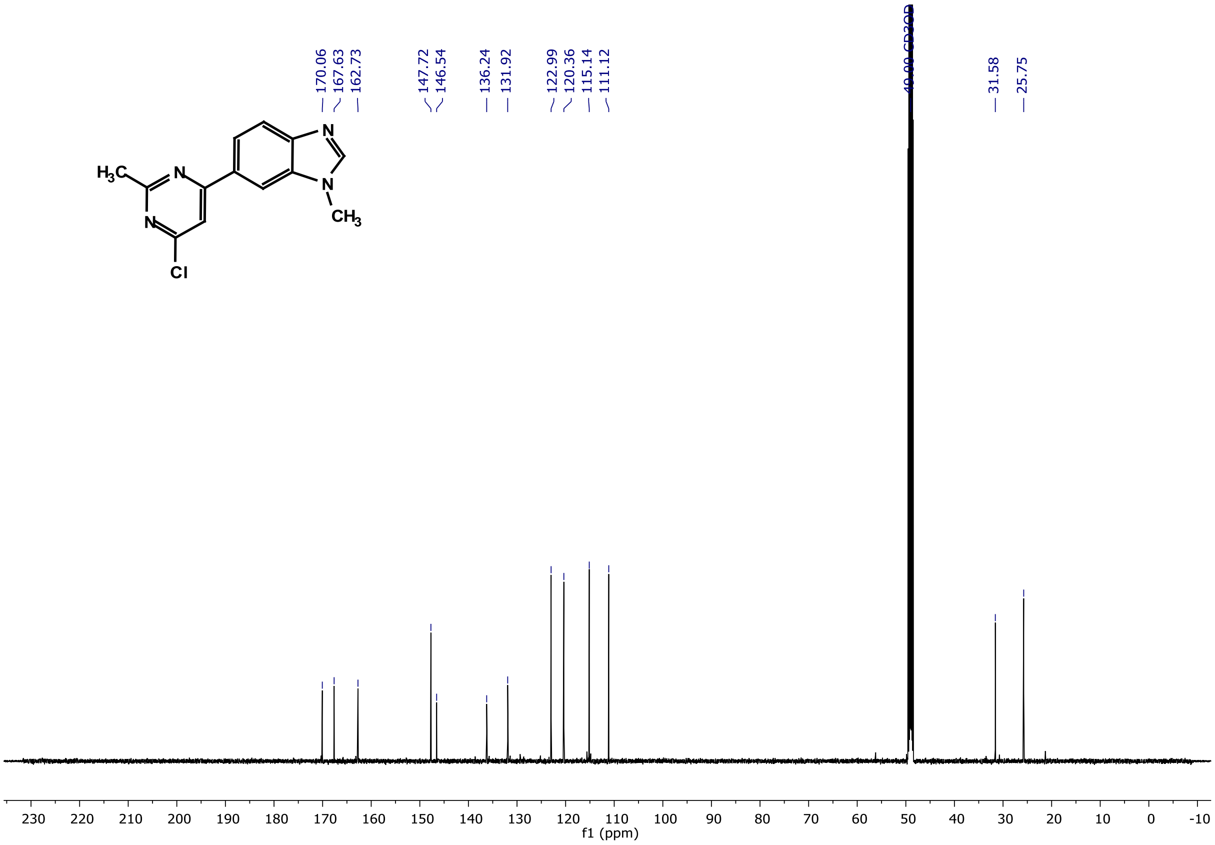

1-Methyl-6-(4-(methylsulfonyl)phenyl)-1*H*-benzo[*d*]imidazole (**2p**)

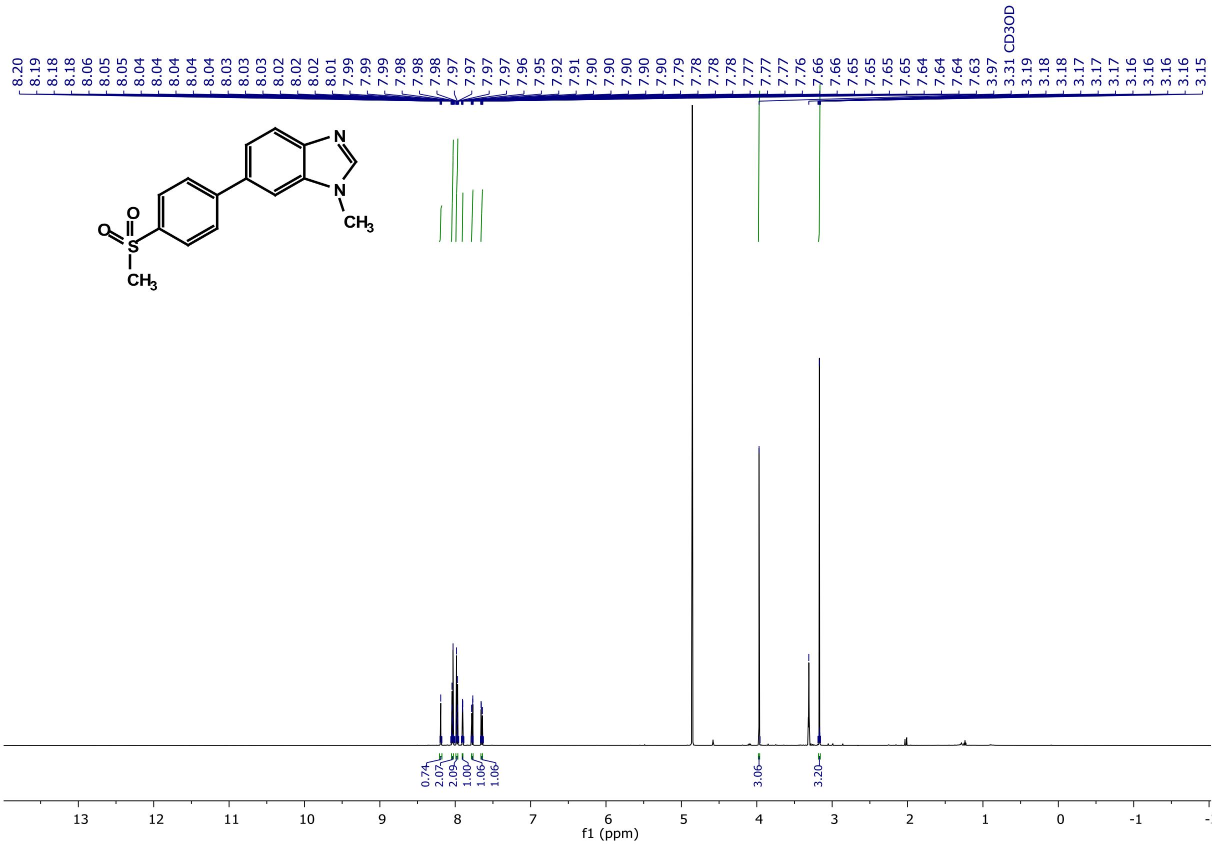

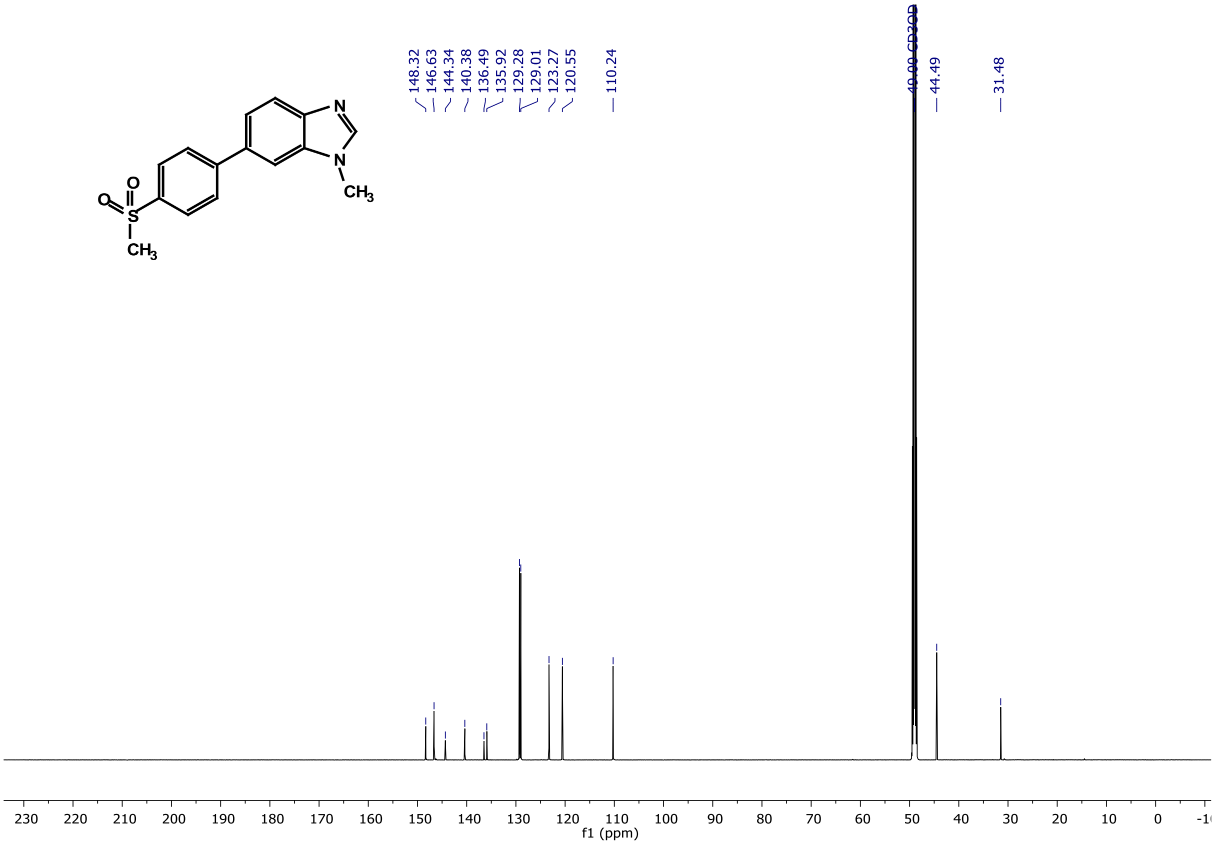

5-Chloro-1-methyl-1*H*-benzo[*d*]imidazole (**2q**)

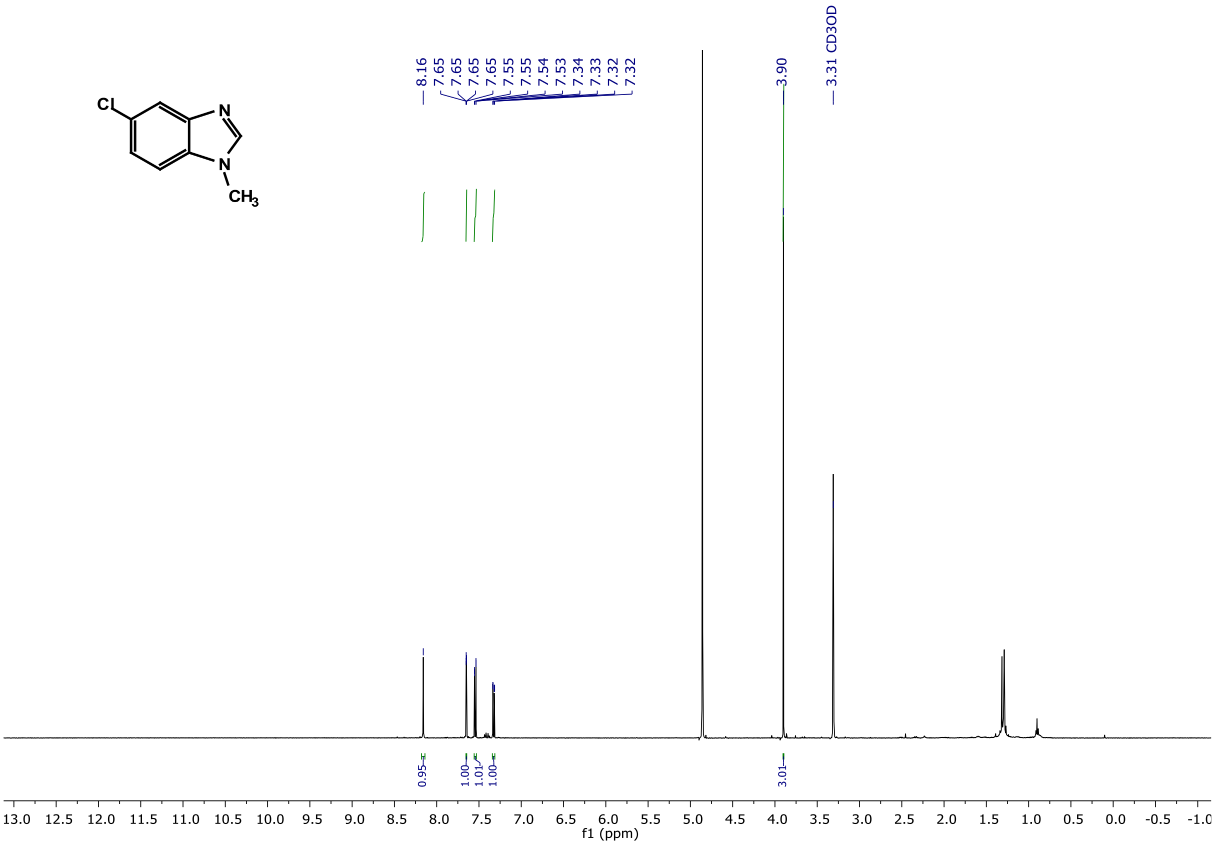

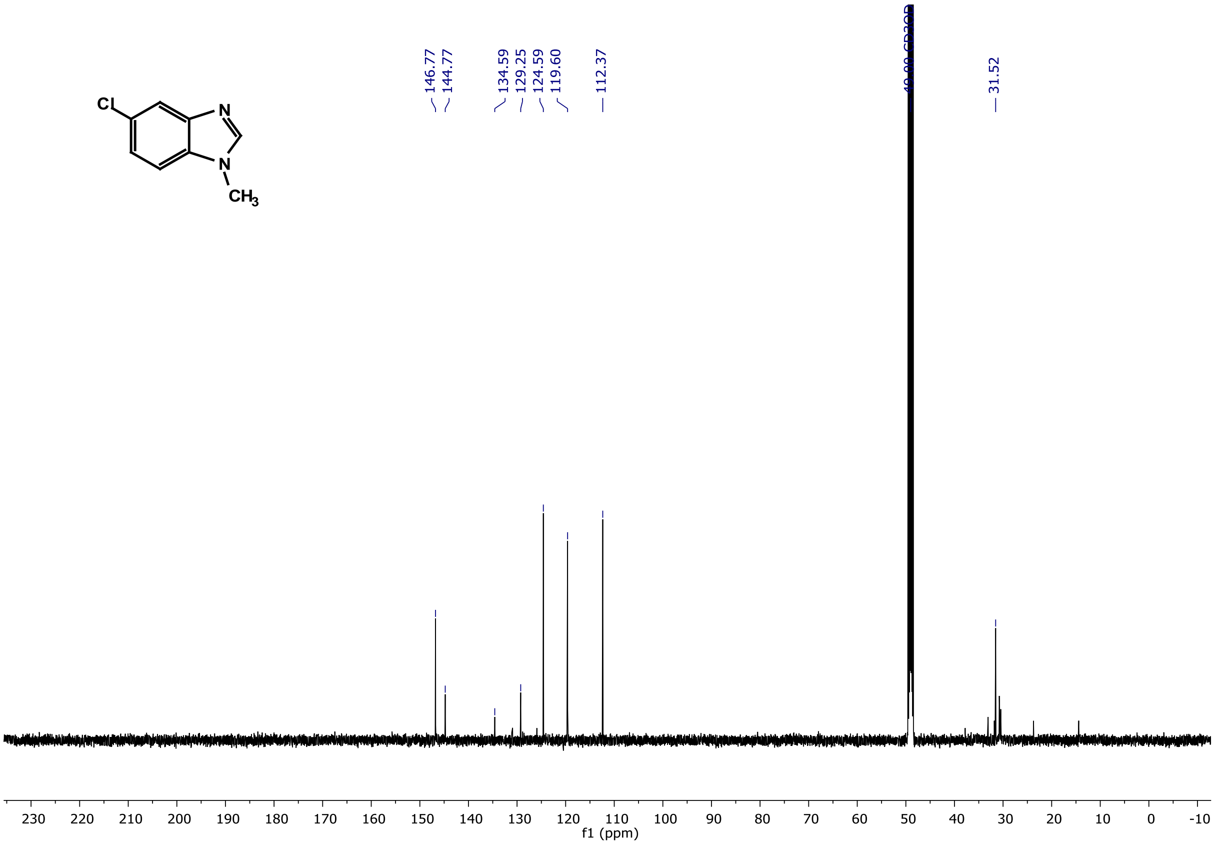

5-Bromo-1-methyl-1*H*-benzo[*d*]imidazole (**2r**)

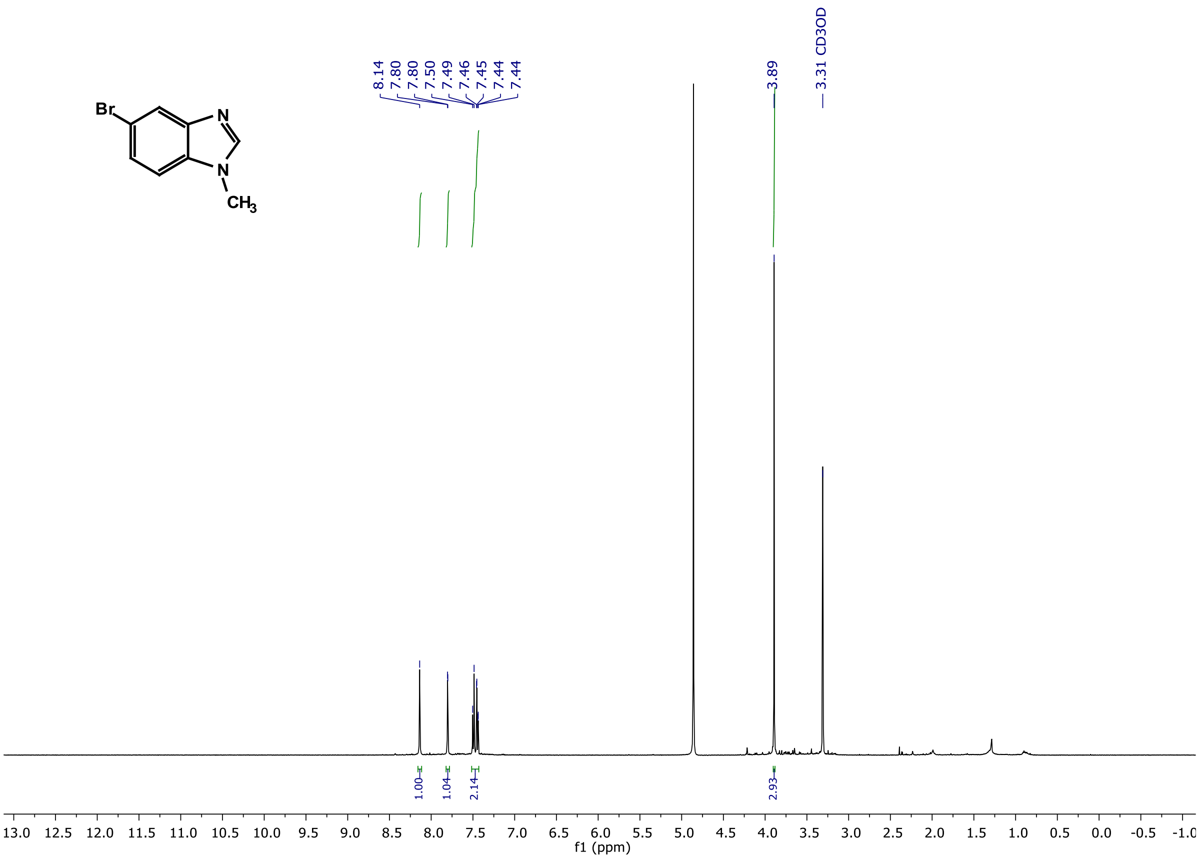

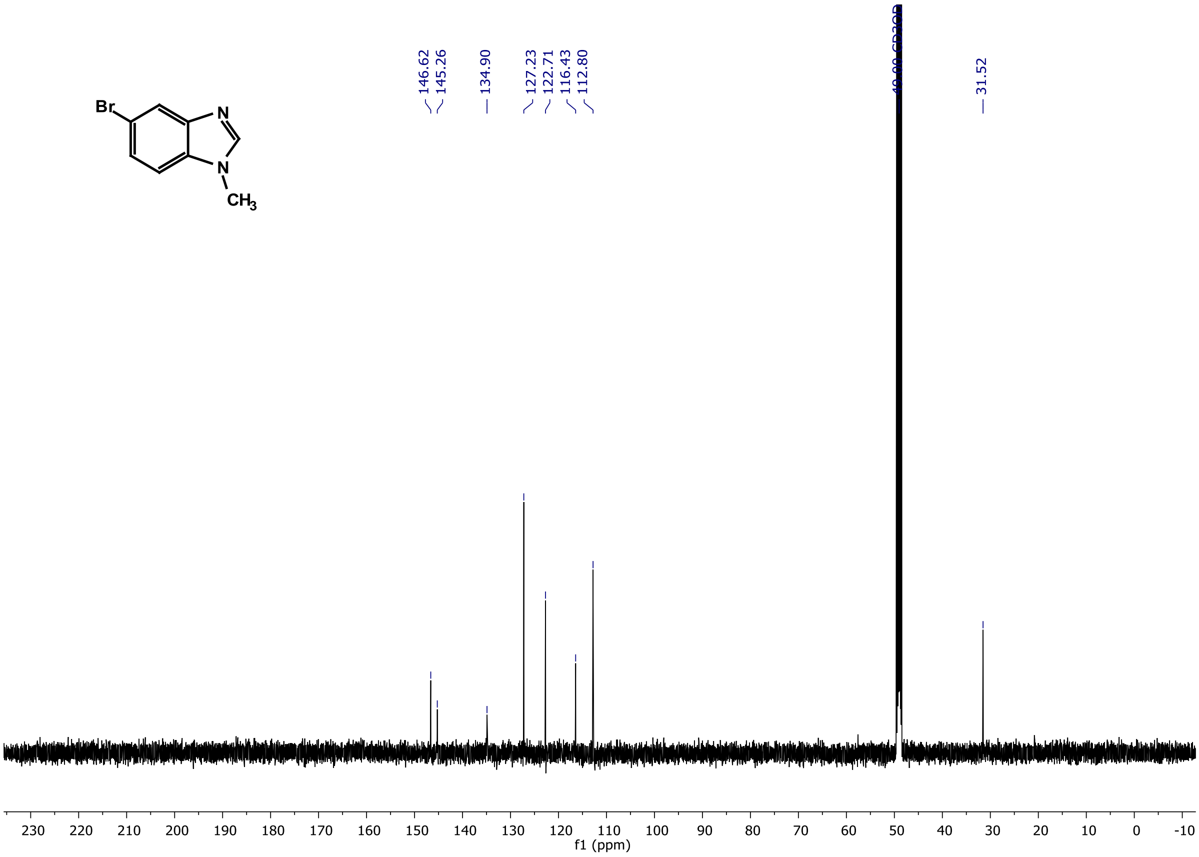

Methyl 1-methyl-1*H*-benzo[*d*]imidazole-5-carboxylate (**2s**)

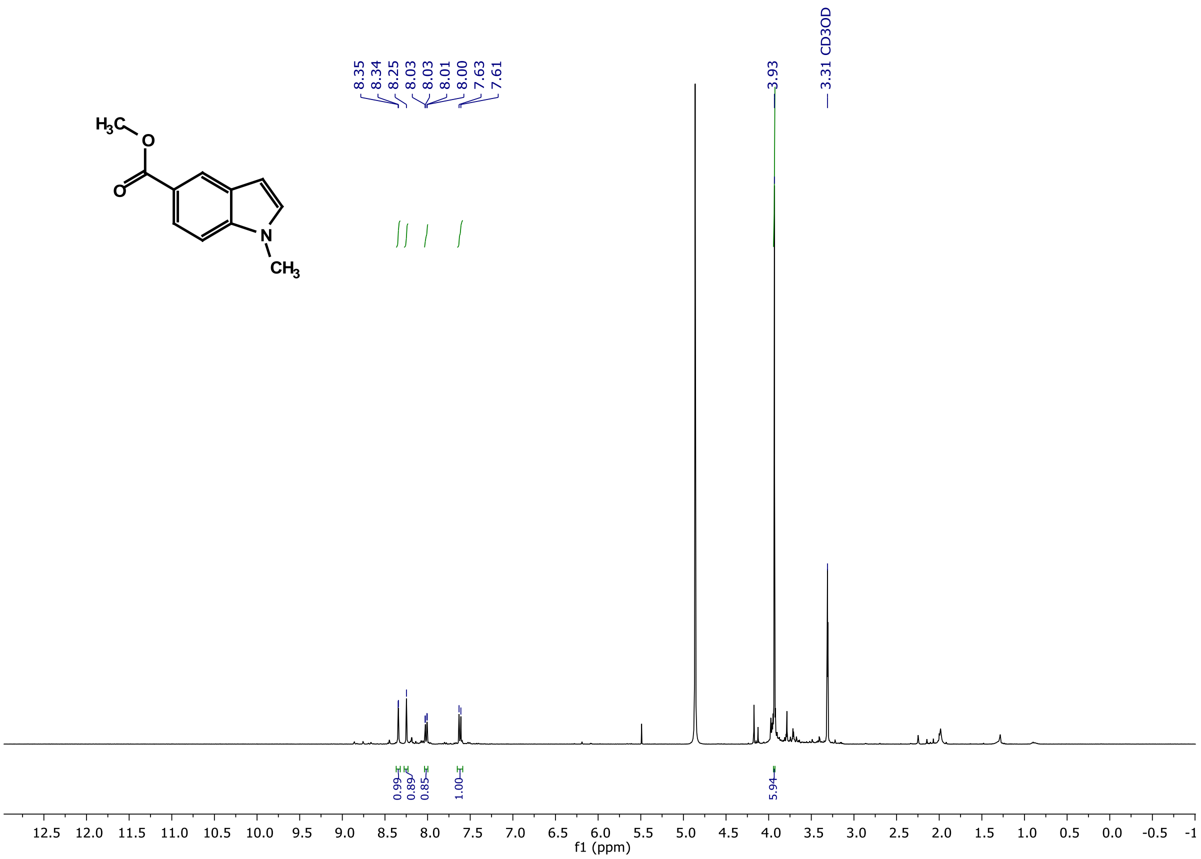

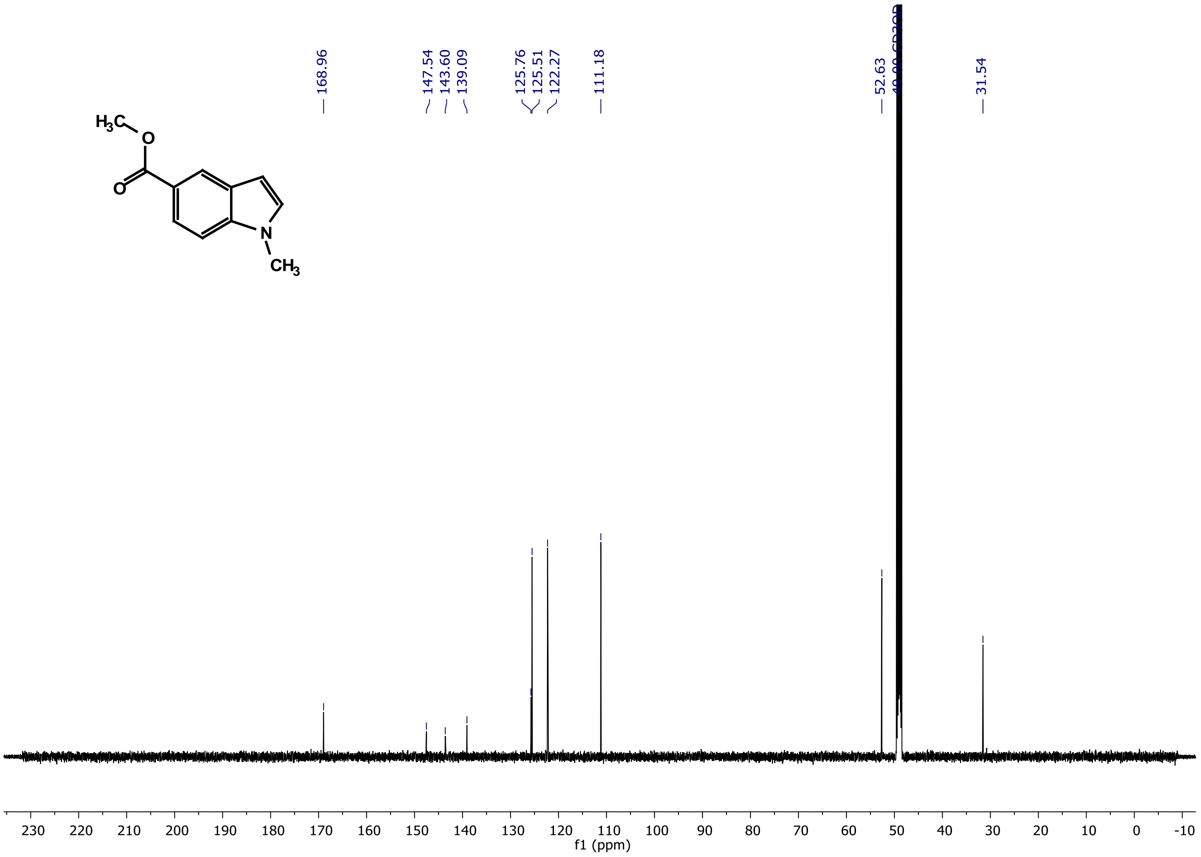

5-Methoxy-1-methyl-1*H*-benzo[*d*]imidazole (**2t**)

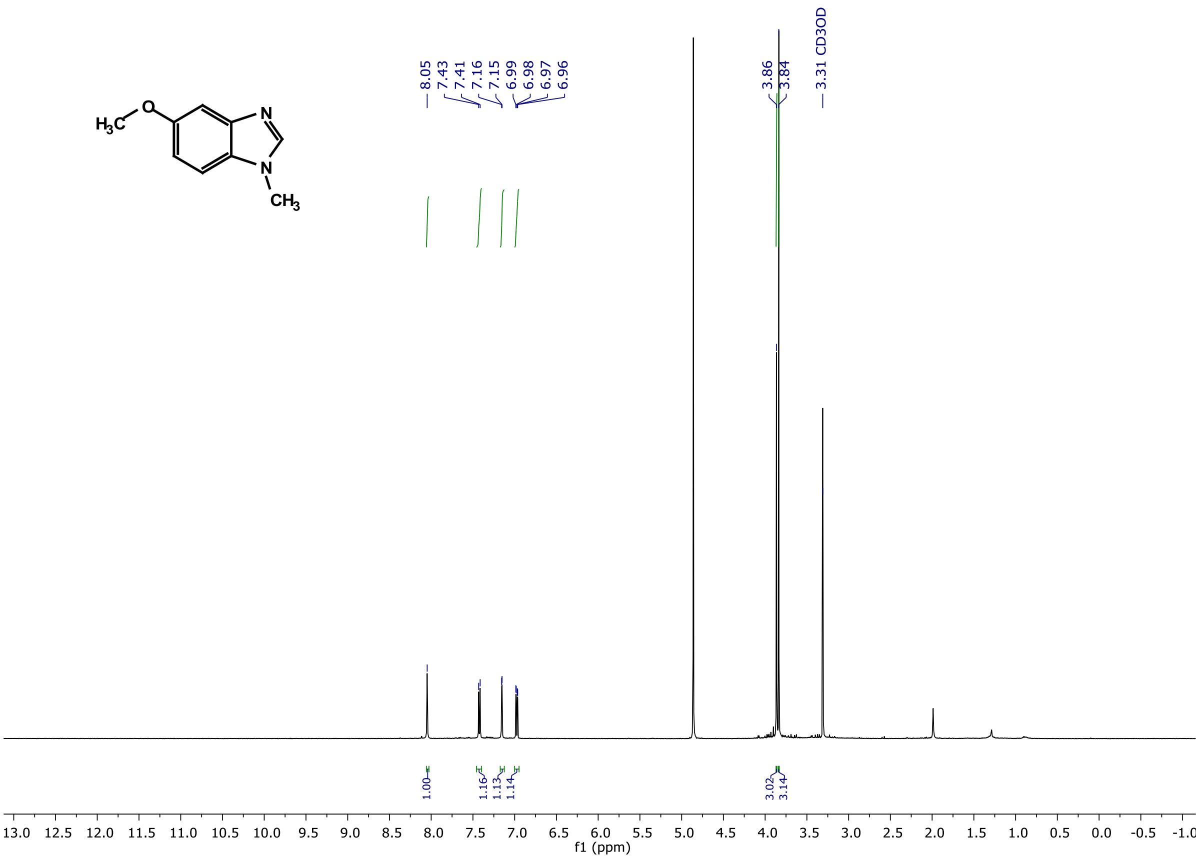

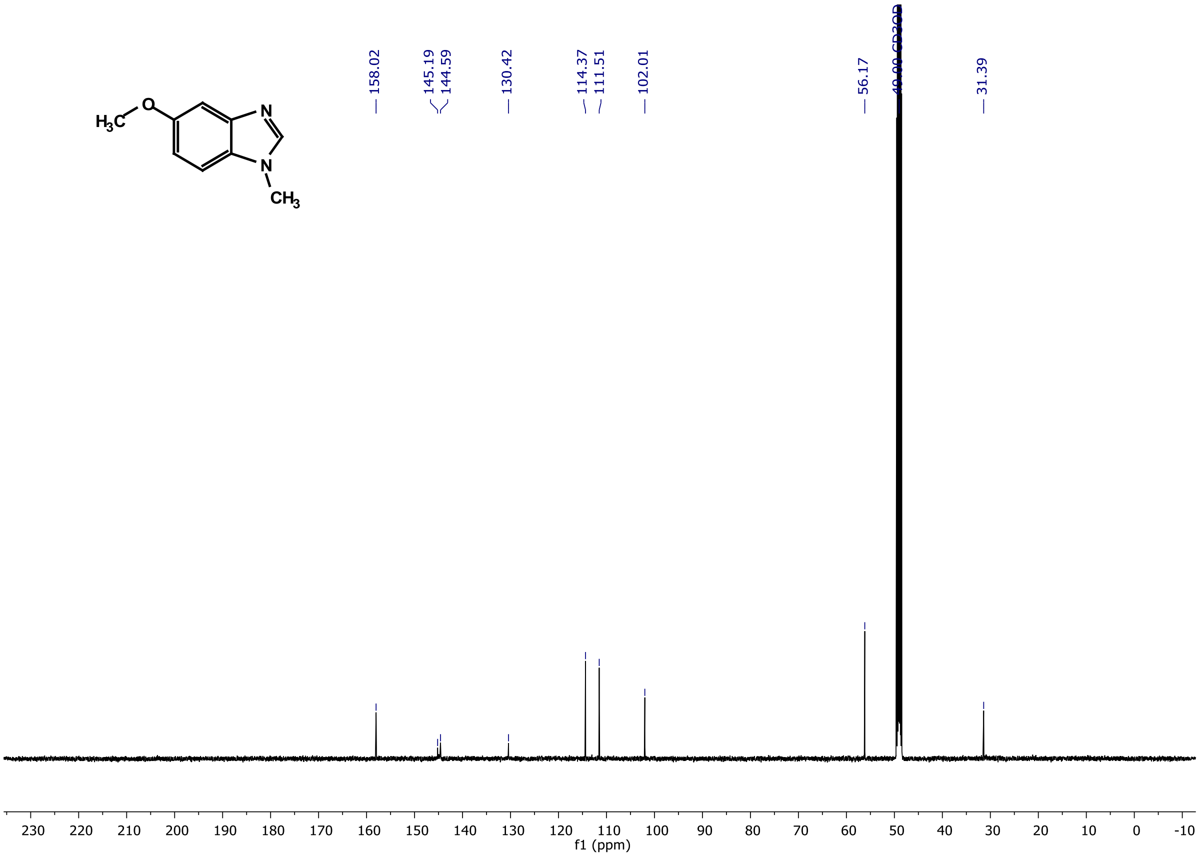

N-(2-cyano-4-methoxyphenyl)-*N*-methylcyanamide (**3t**)

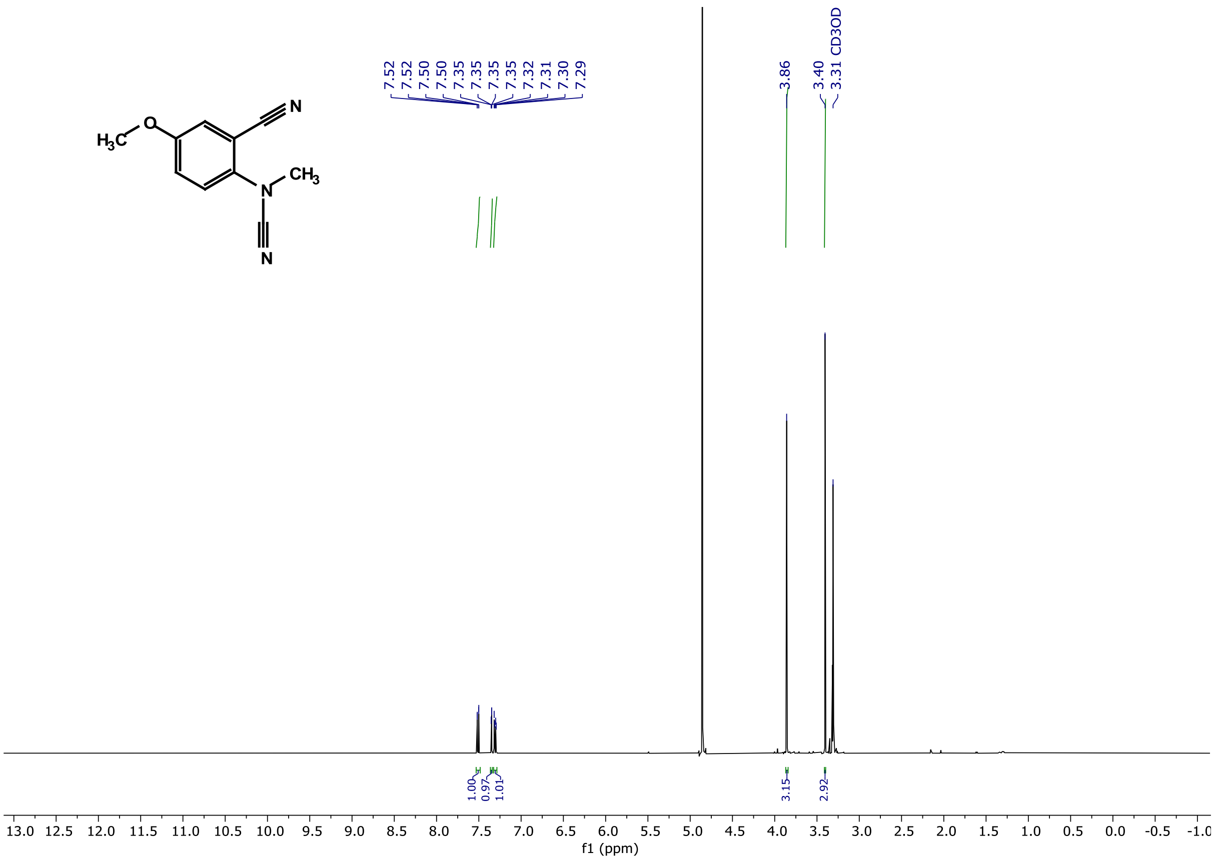

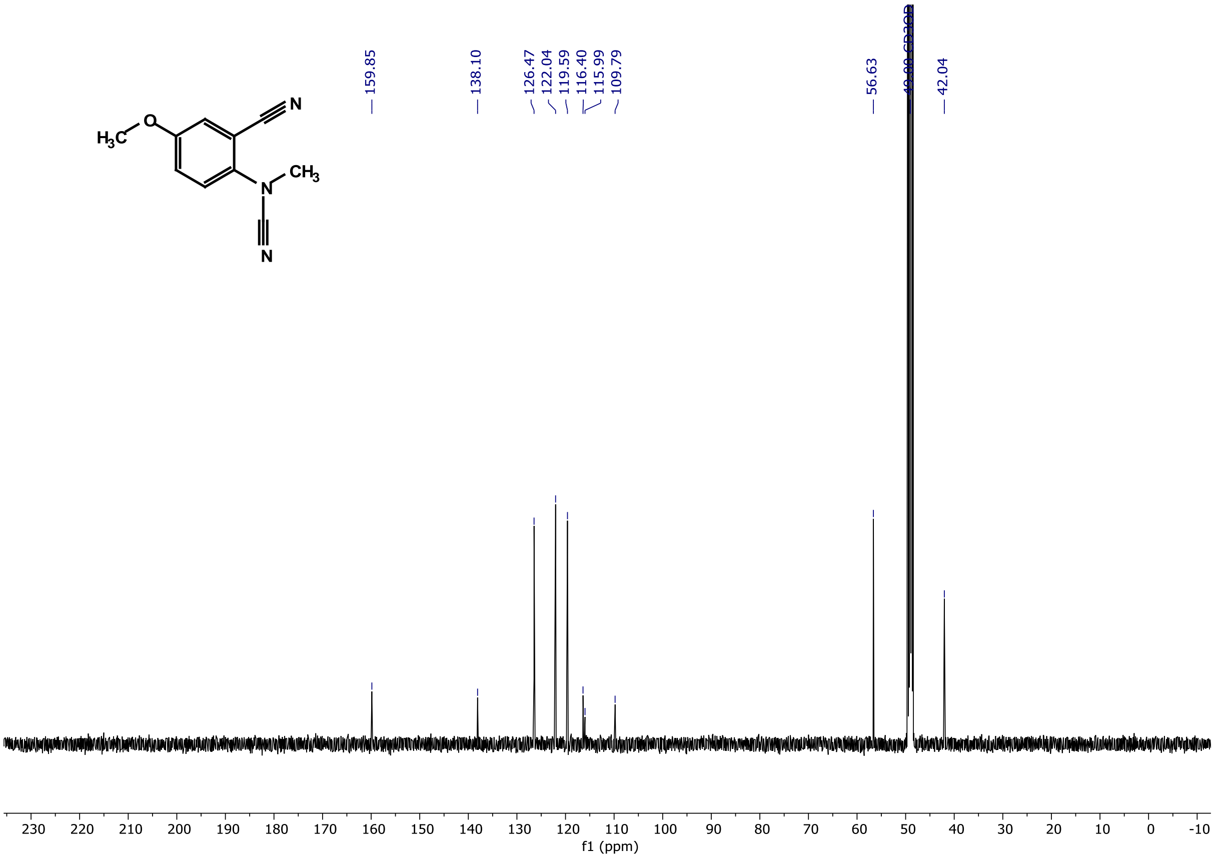

(1-Methyl-1H-benzo[d]imidazol-6-yl)methanol (**2u**)

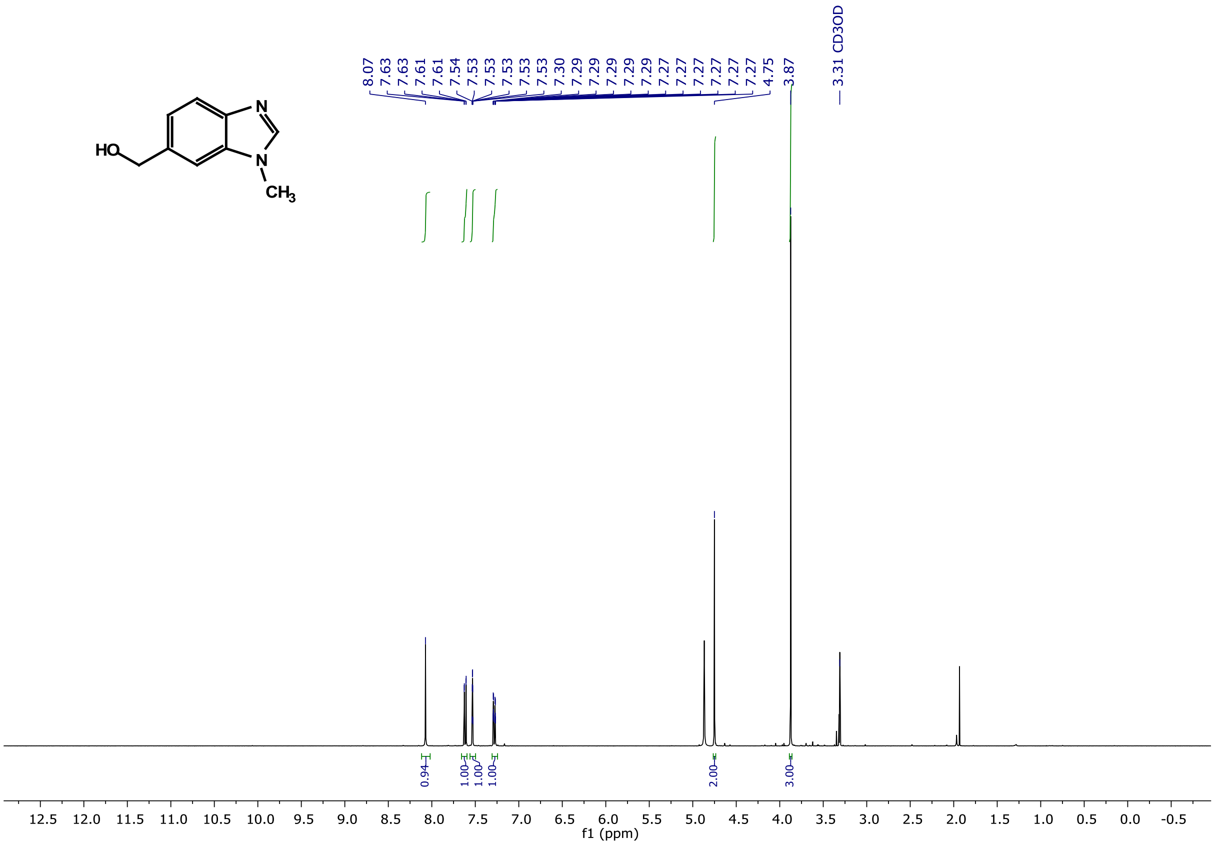

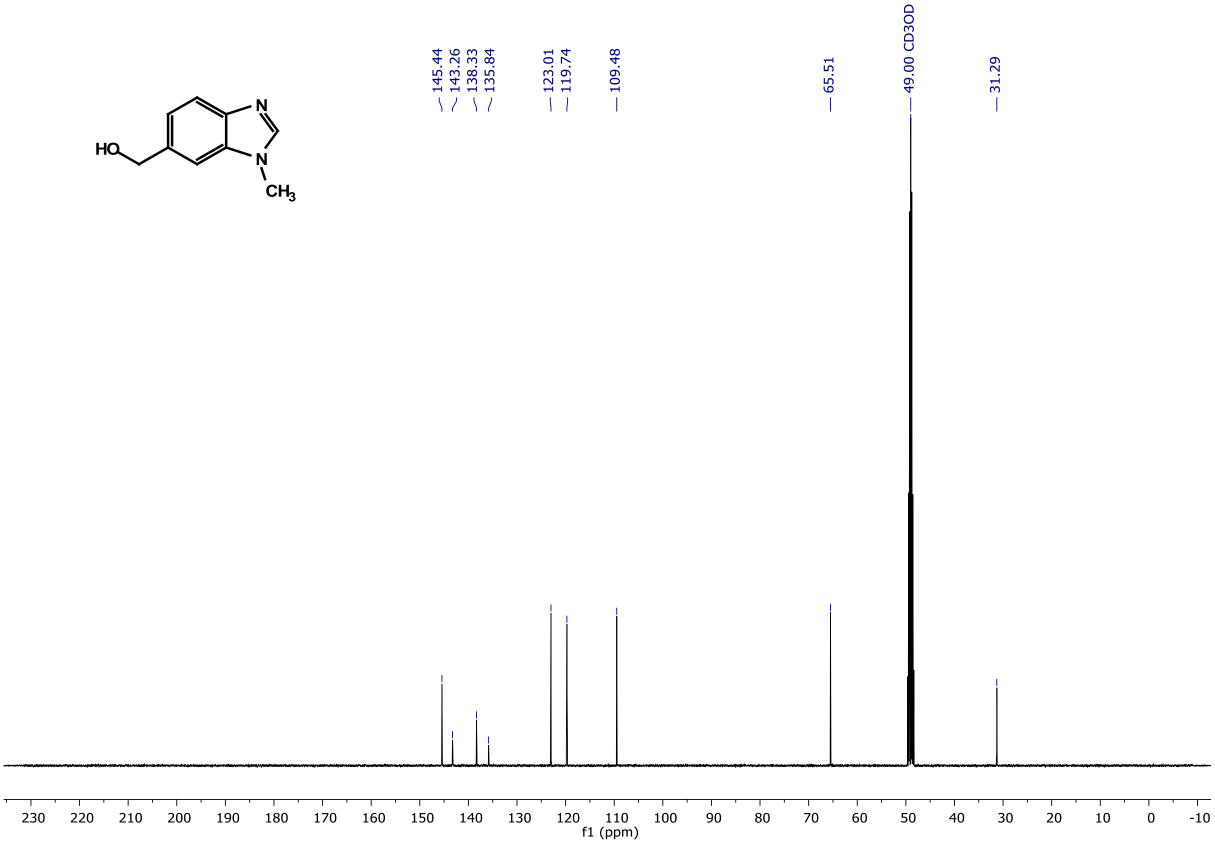

N-(1-methyl-1*H*-benzo[*d*]imidazol-6-yl)acetamide (**2v**)

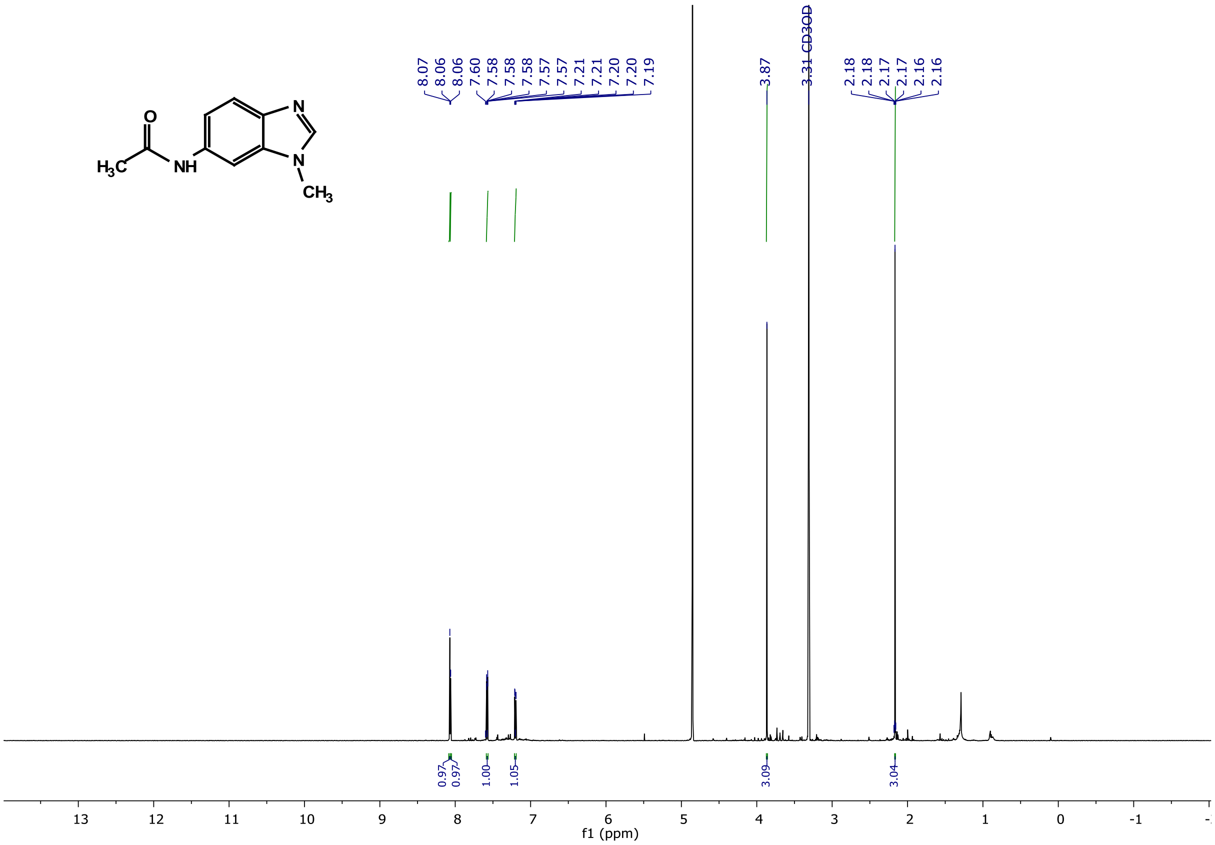

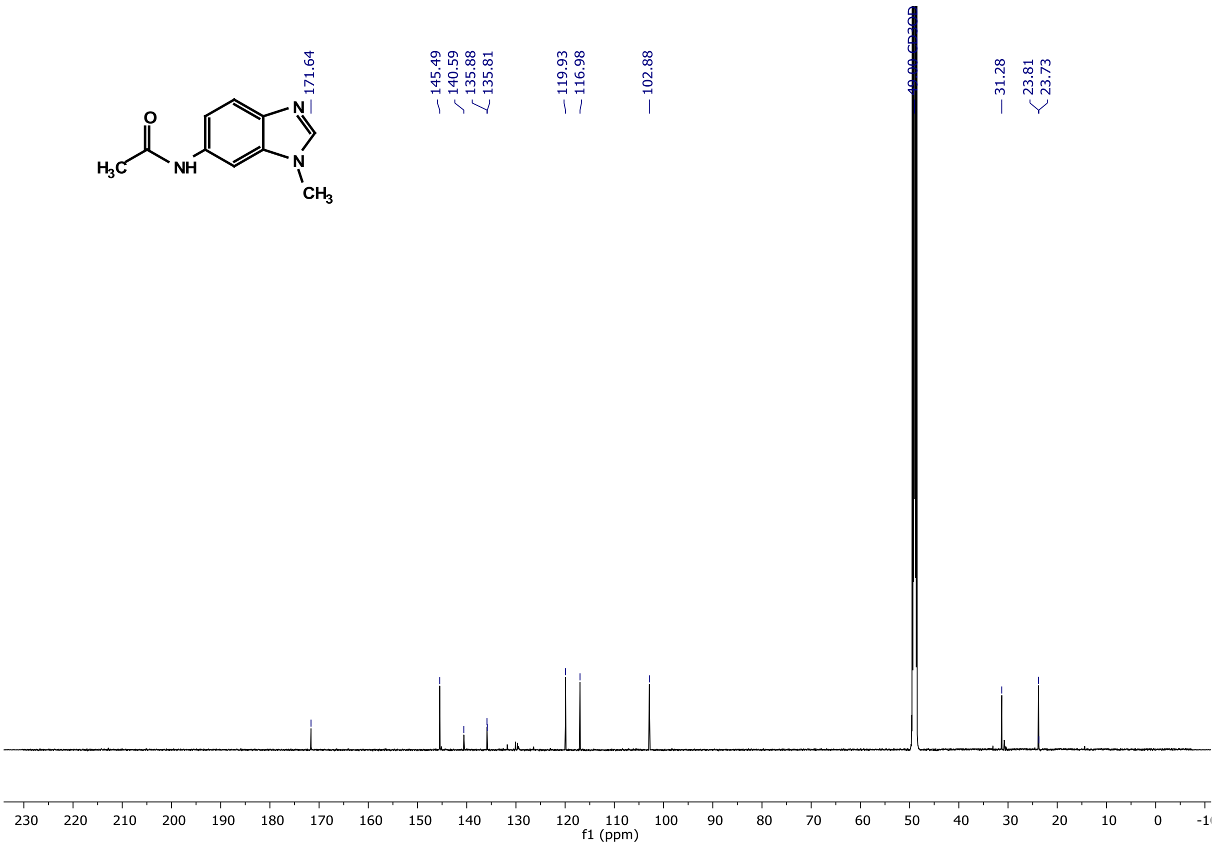

4-Ethoxy-1-(2,2,2-trifluoroethyl)-1*H*-benzo[*d*]imidazole-6-carboxylic acid (**2w**)

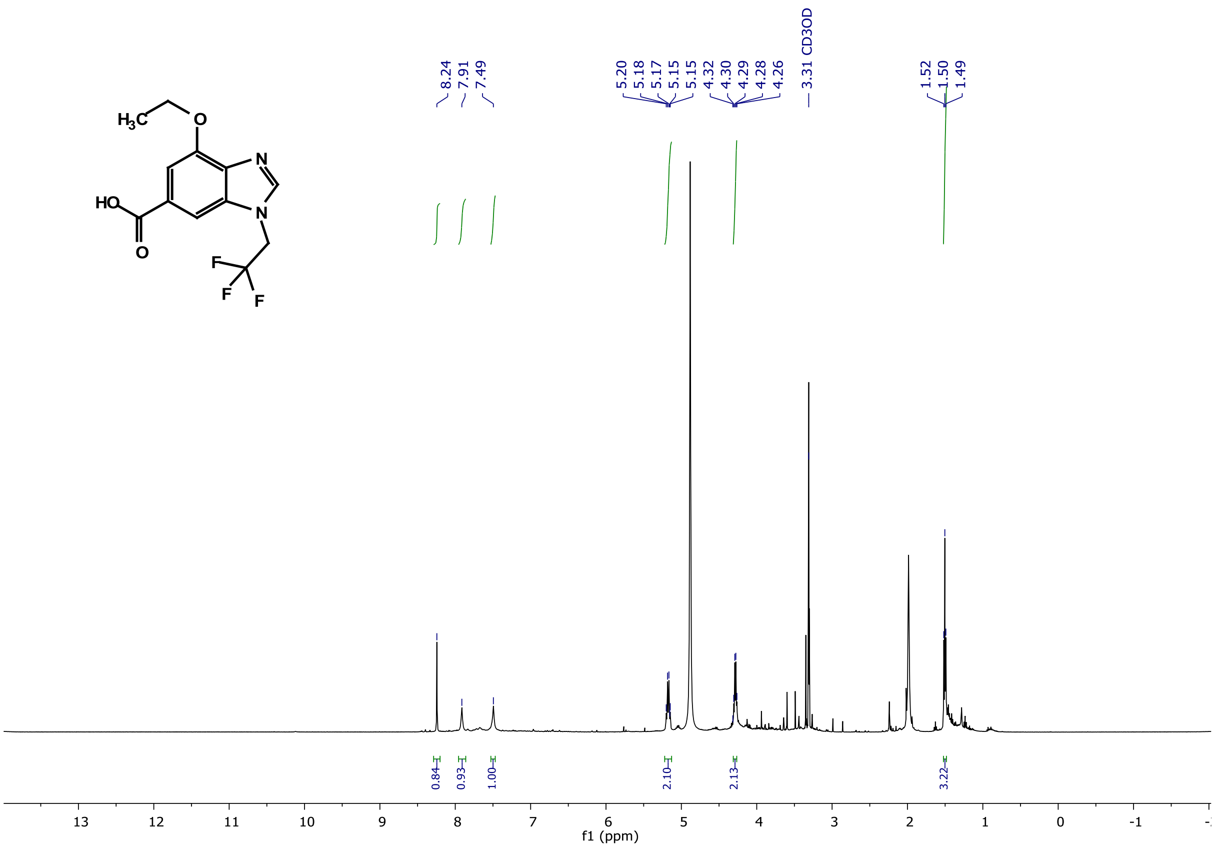

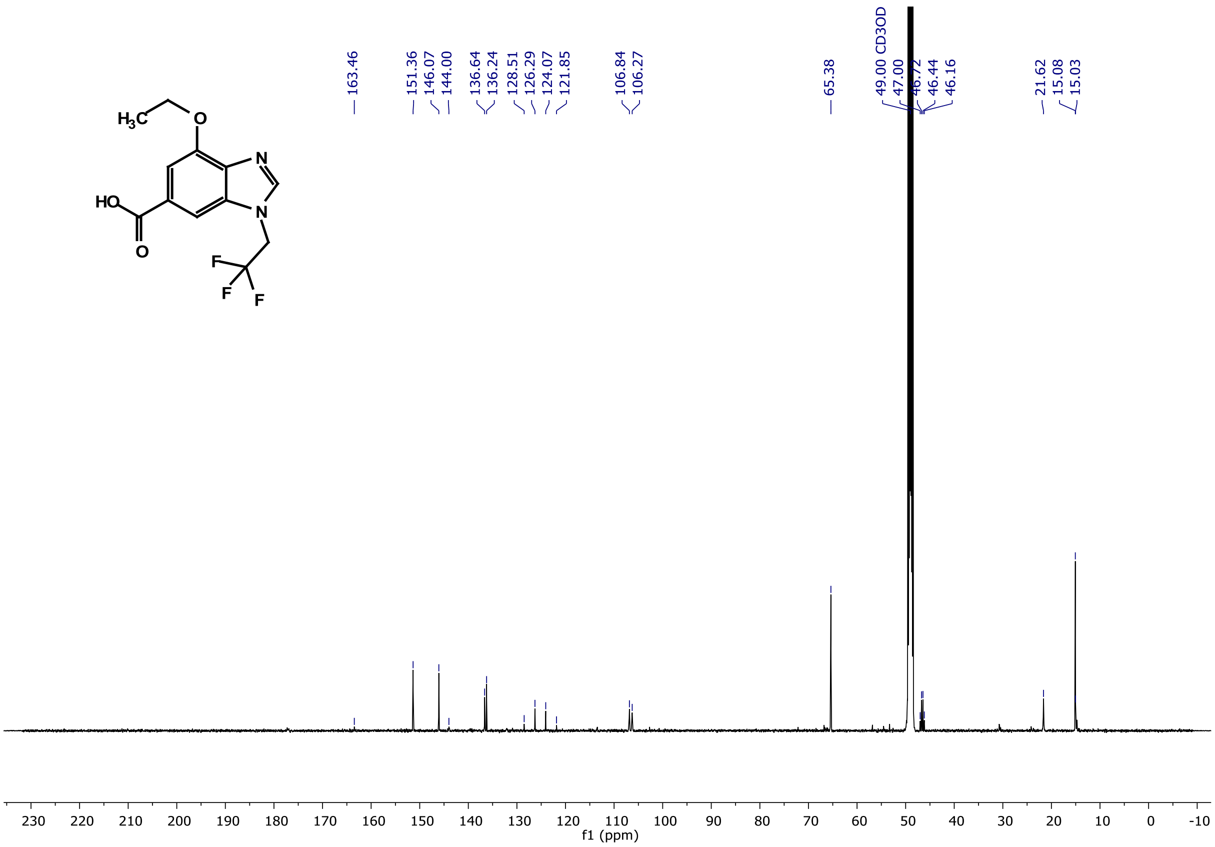

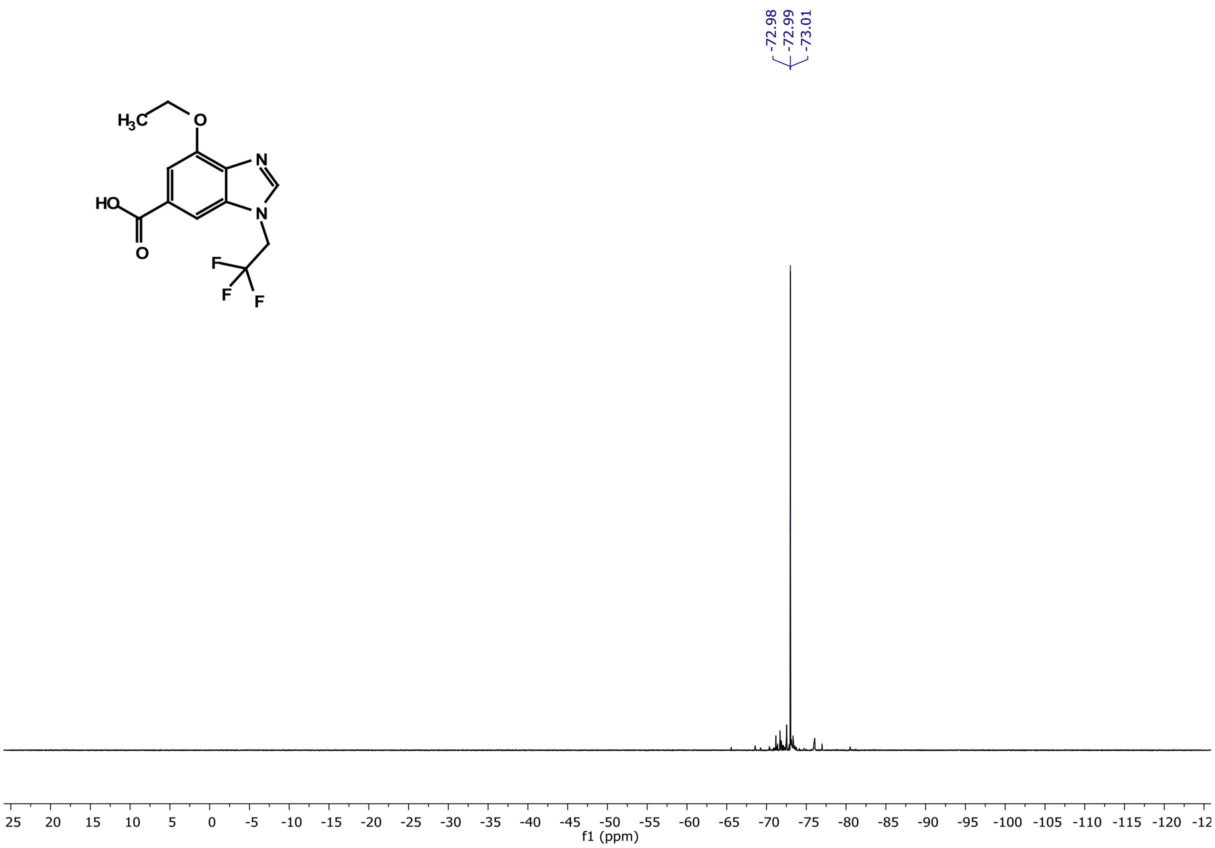

1-Methyl-1*H*-benzo[*d*]imidazole-3-^{15}N (**2x**)

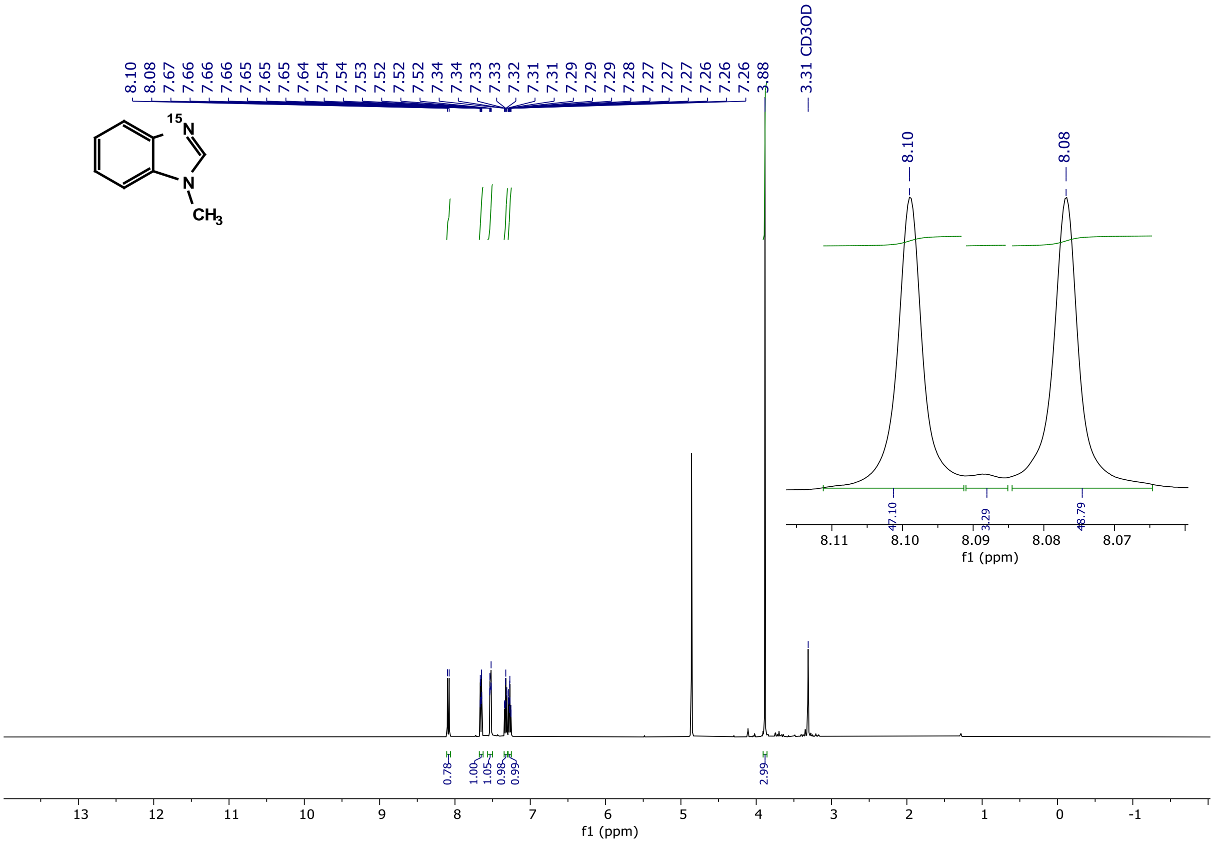

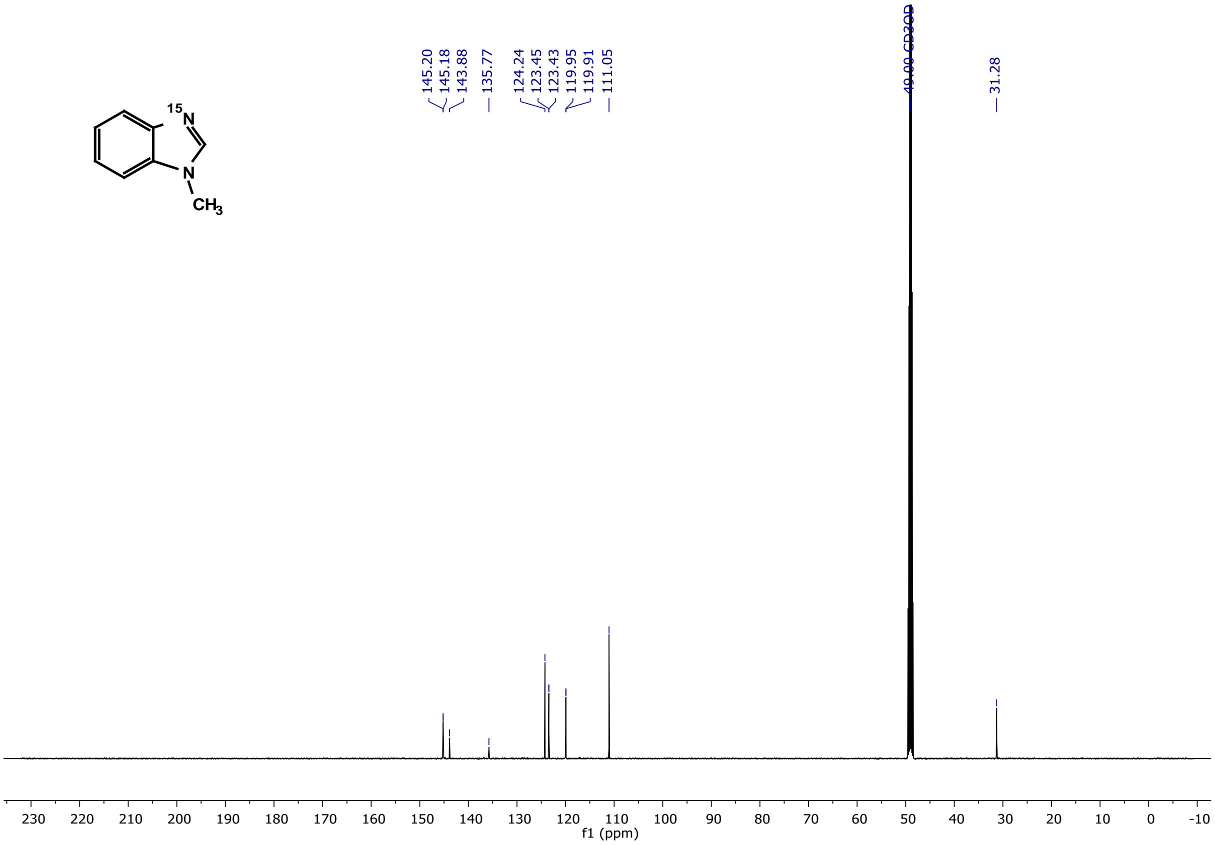

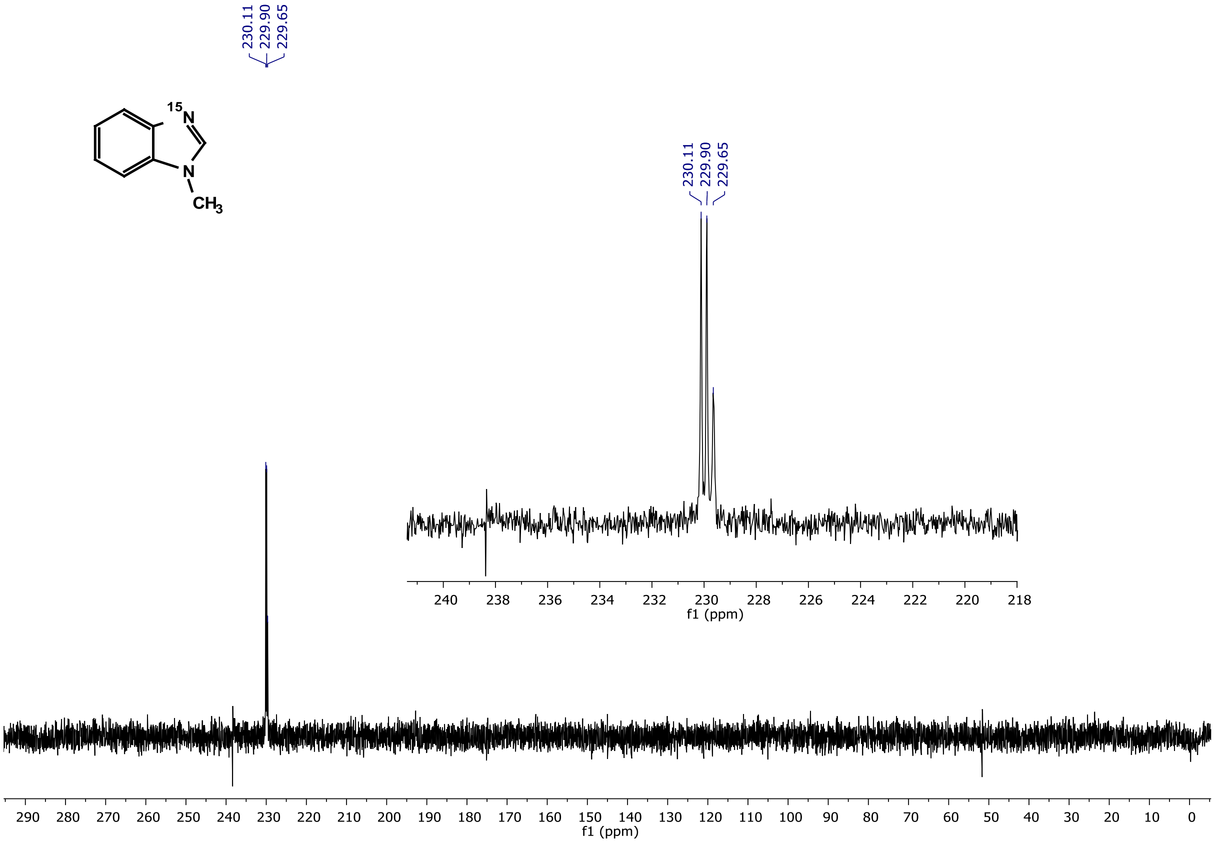

Partial deuteration in C-2 position leads to the third signal at 229.65 ppm.

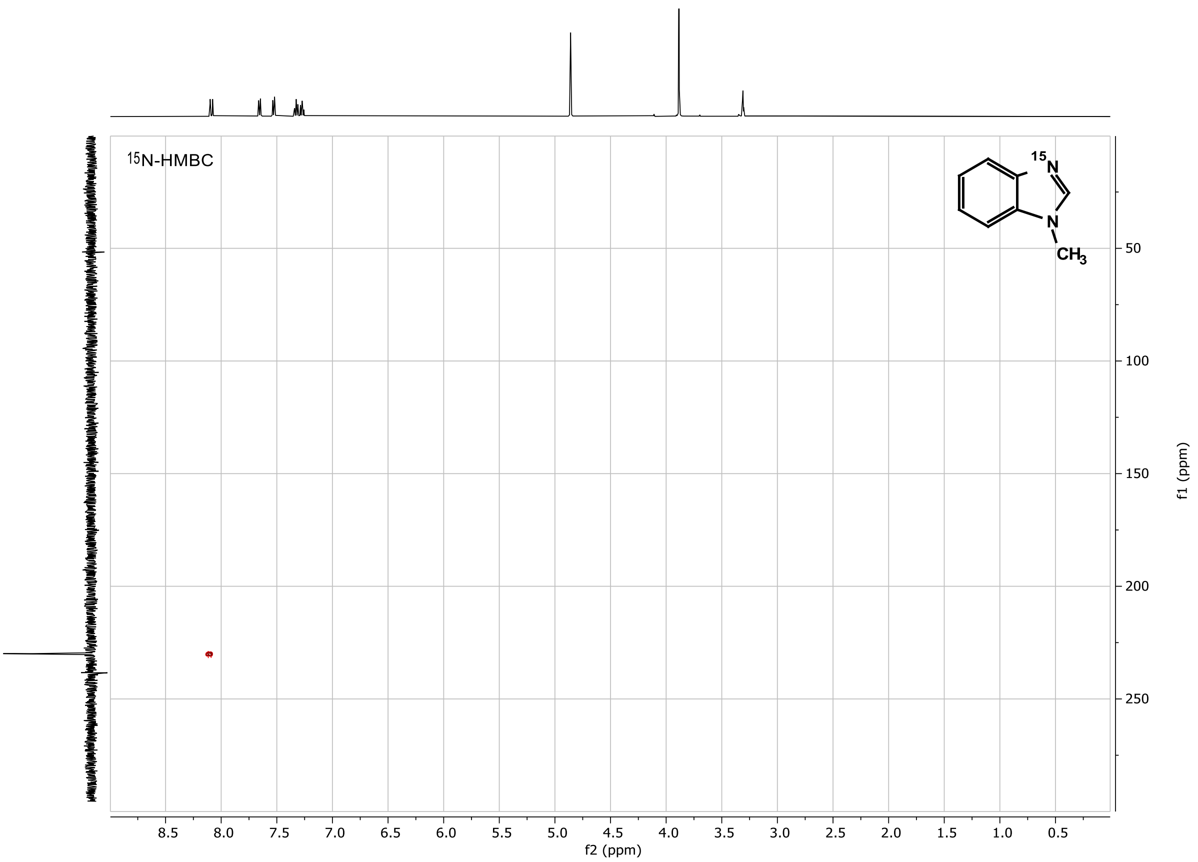

N-(4-(tert-butyl)benzyl)-*N*,1-dimethyl-1*H*-benzo[*d*]imidazole-7-carboxamide (**2y**)

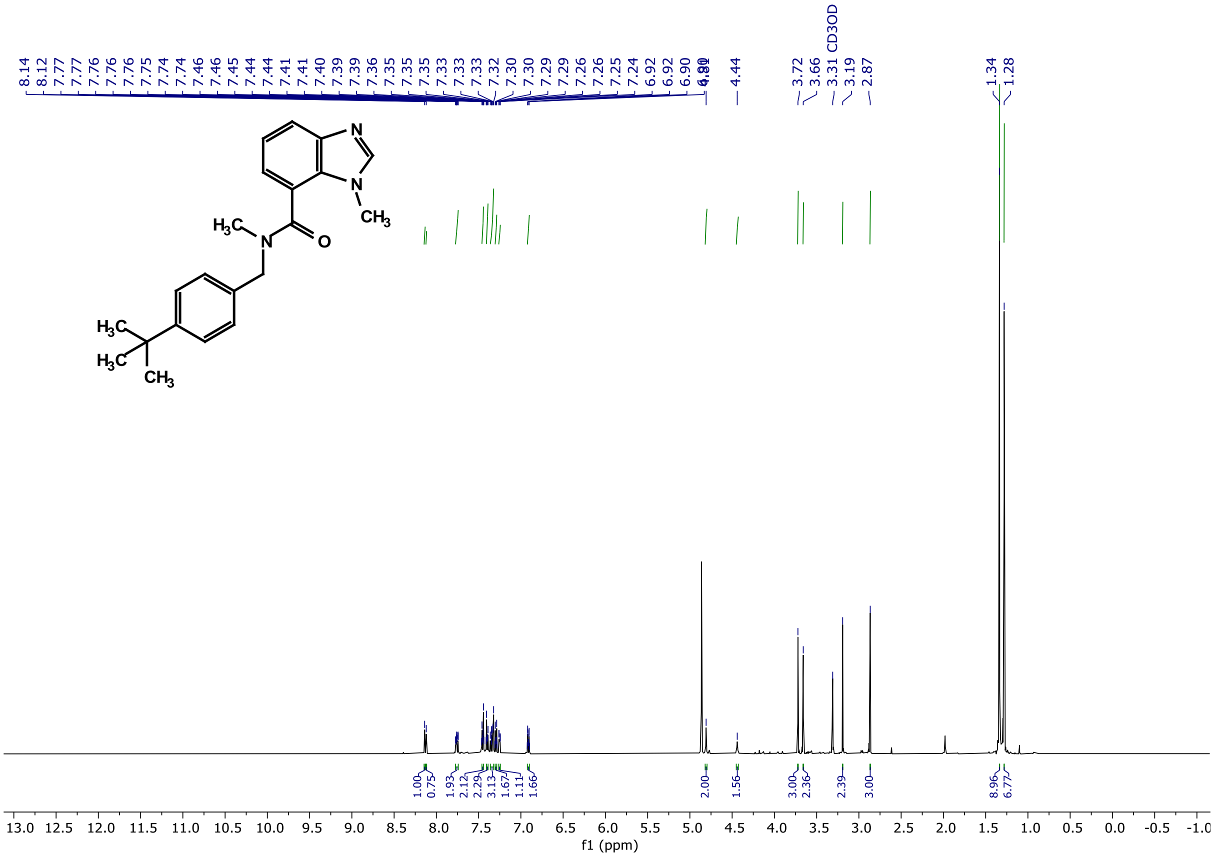

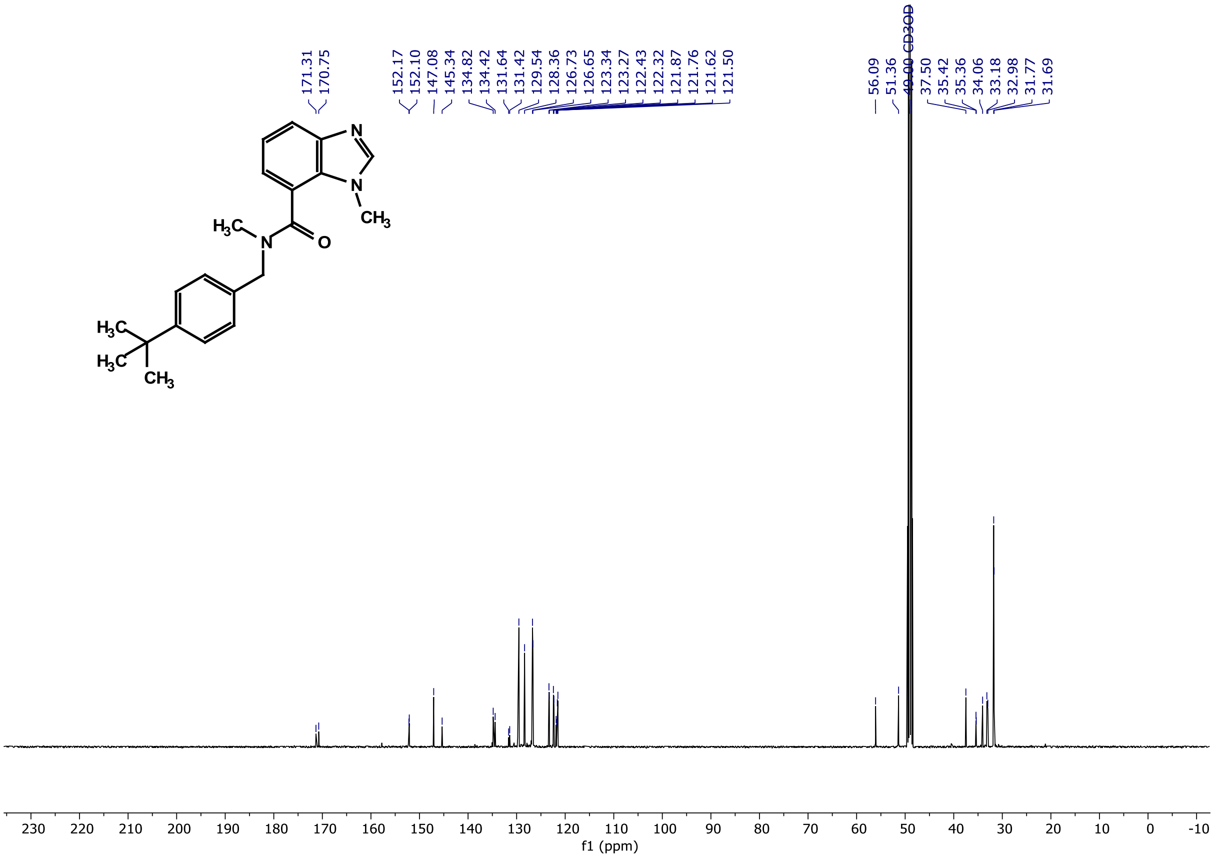
171.31
170.75
152.17
152.10
147.08
145.34
134.82
134.42
131.64
131.42
129.54
128.36
126.73
126.65
123.34
123.27
122.43
122.32
121.87
121.76
121.62
121.50
56.09
51.36
49.00 CD3OD
37.50
35.42
35.36
34.06
33.18
32.98
31.77
31.69
f1 (ppm)

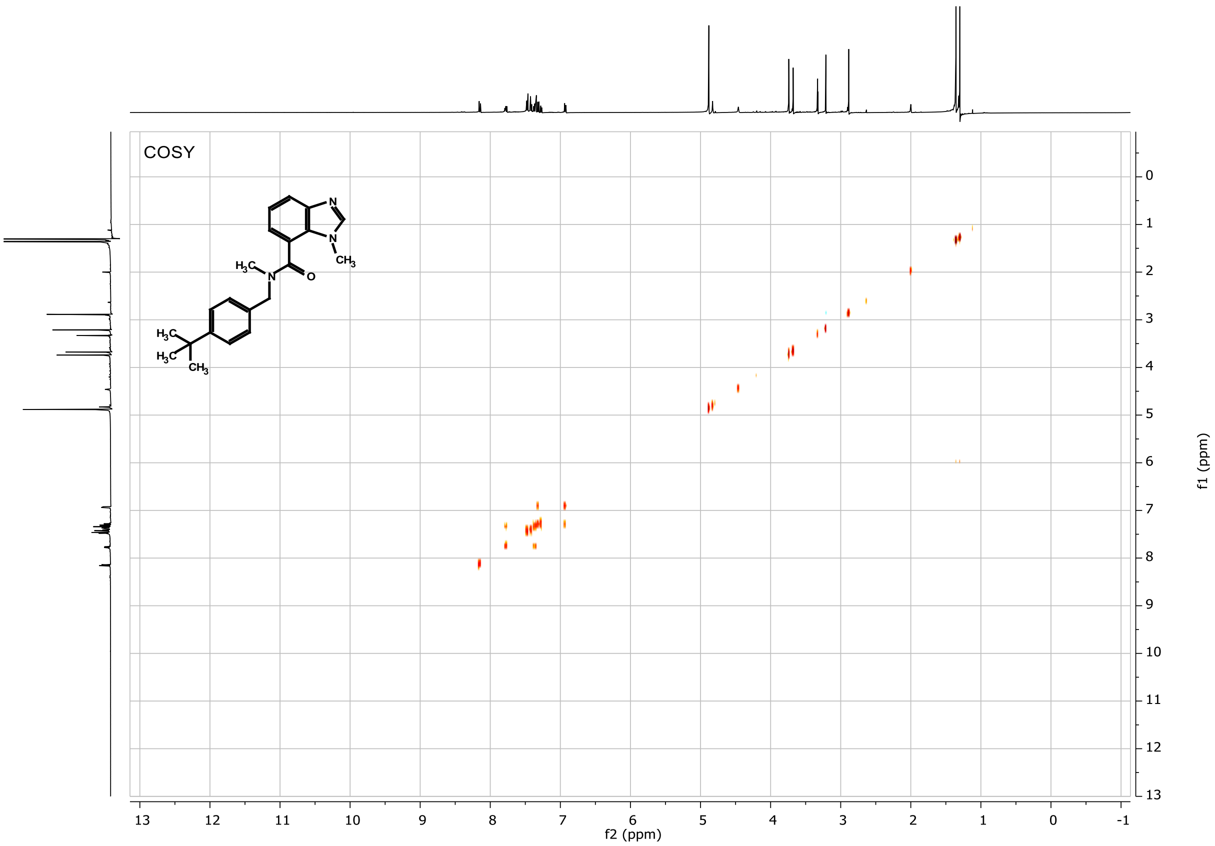
COSY
f2 (ppm)
f1 (ppm)

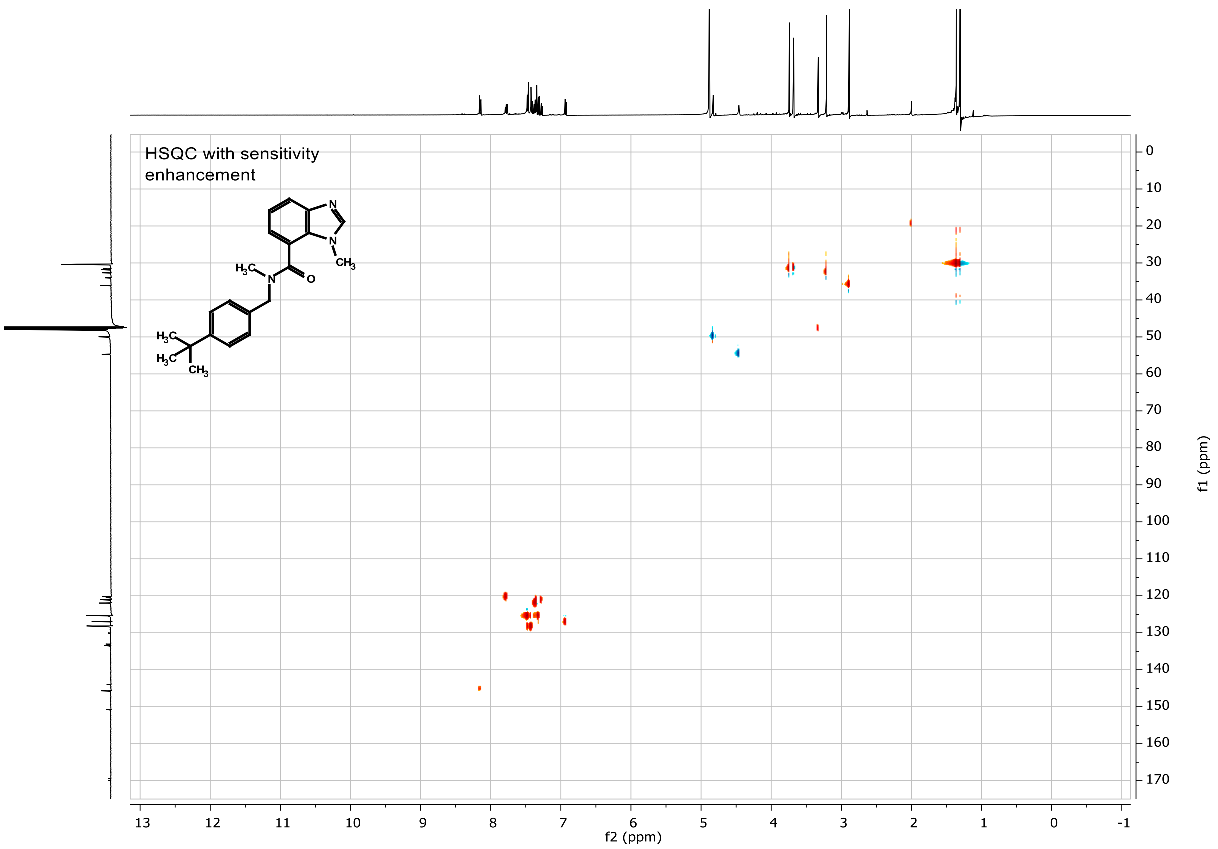
HSQC with sensitivity enhancement
f2 (ppm)
f1 (ppm)

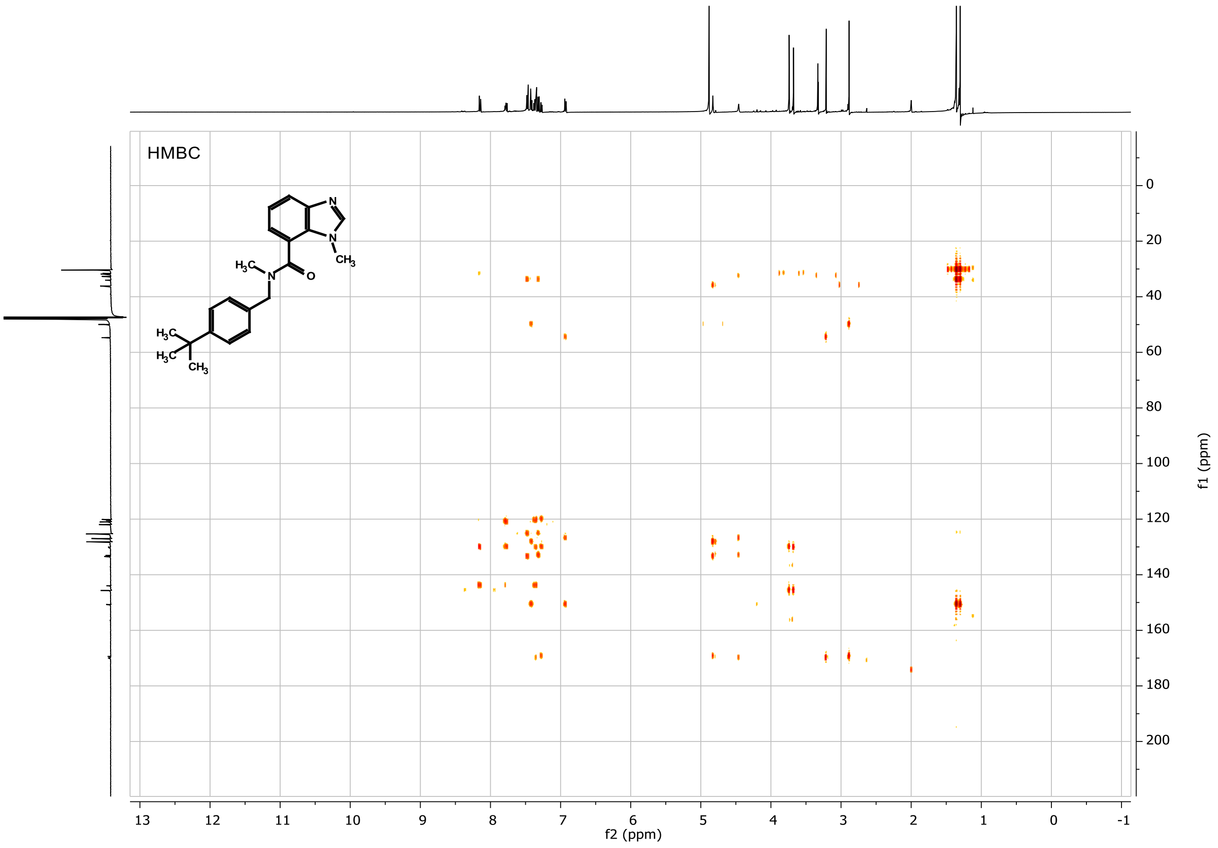
HMBC
f2 (ppm)
f1 (ppm)

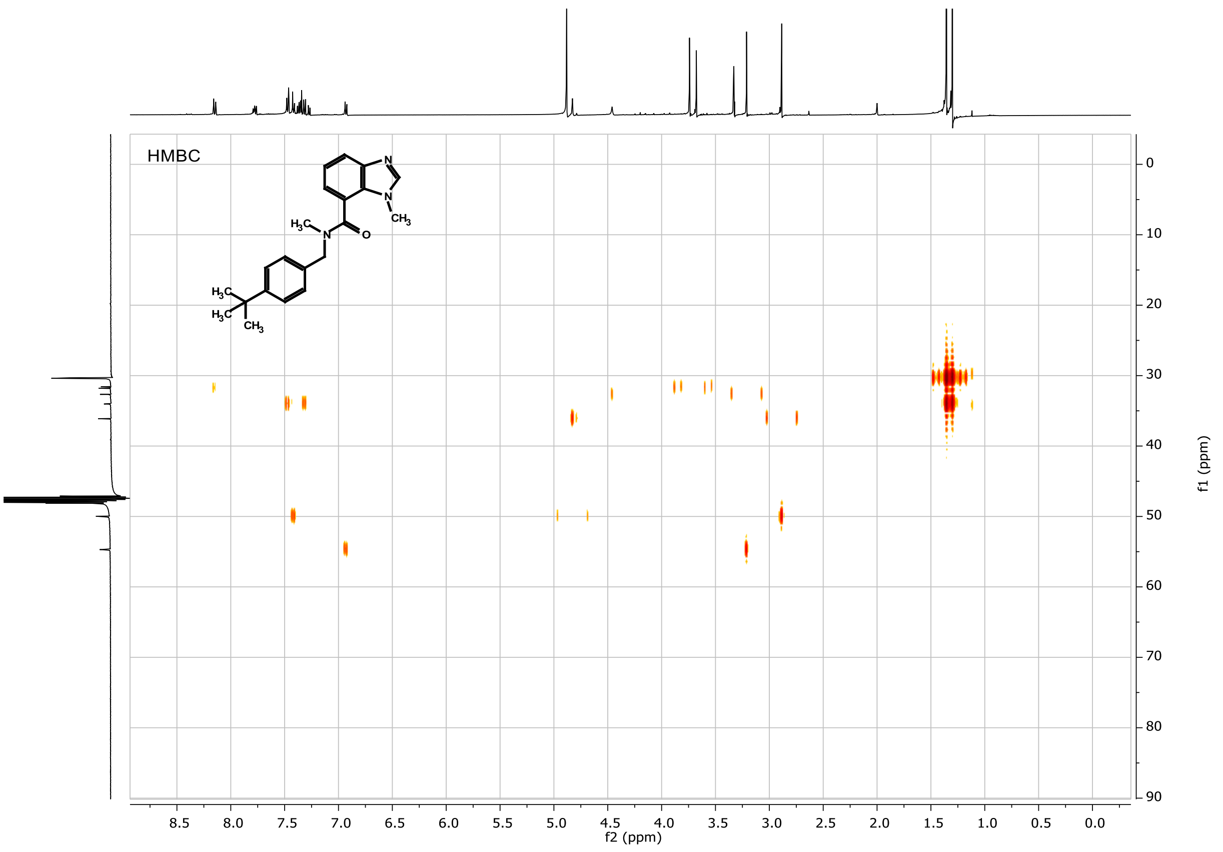
HMBC
f2 (ppm)
f1 (ppm)

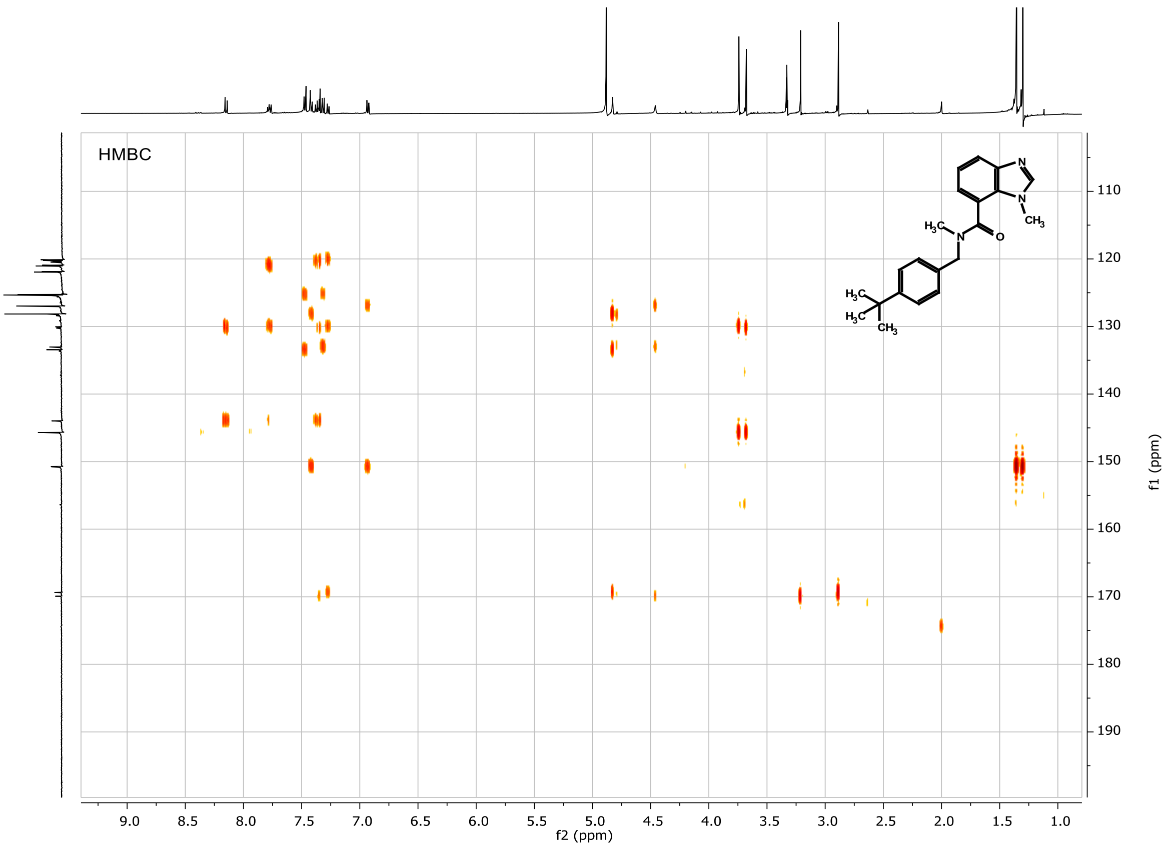
HMBC
f2 (ppm)
f1 (ppm)

1,4-Dimethyl-1*H*-benzo[*d*]imidazole (**2z**)

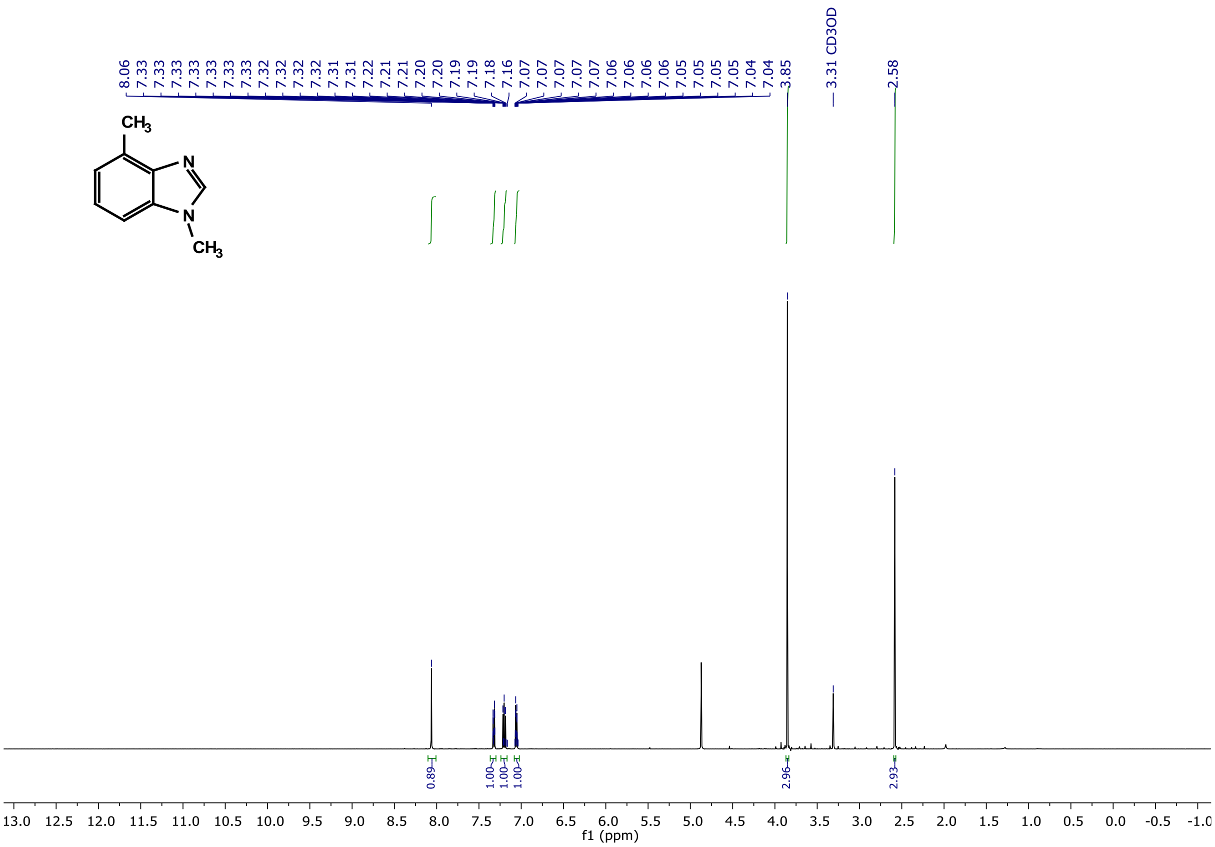

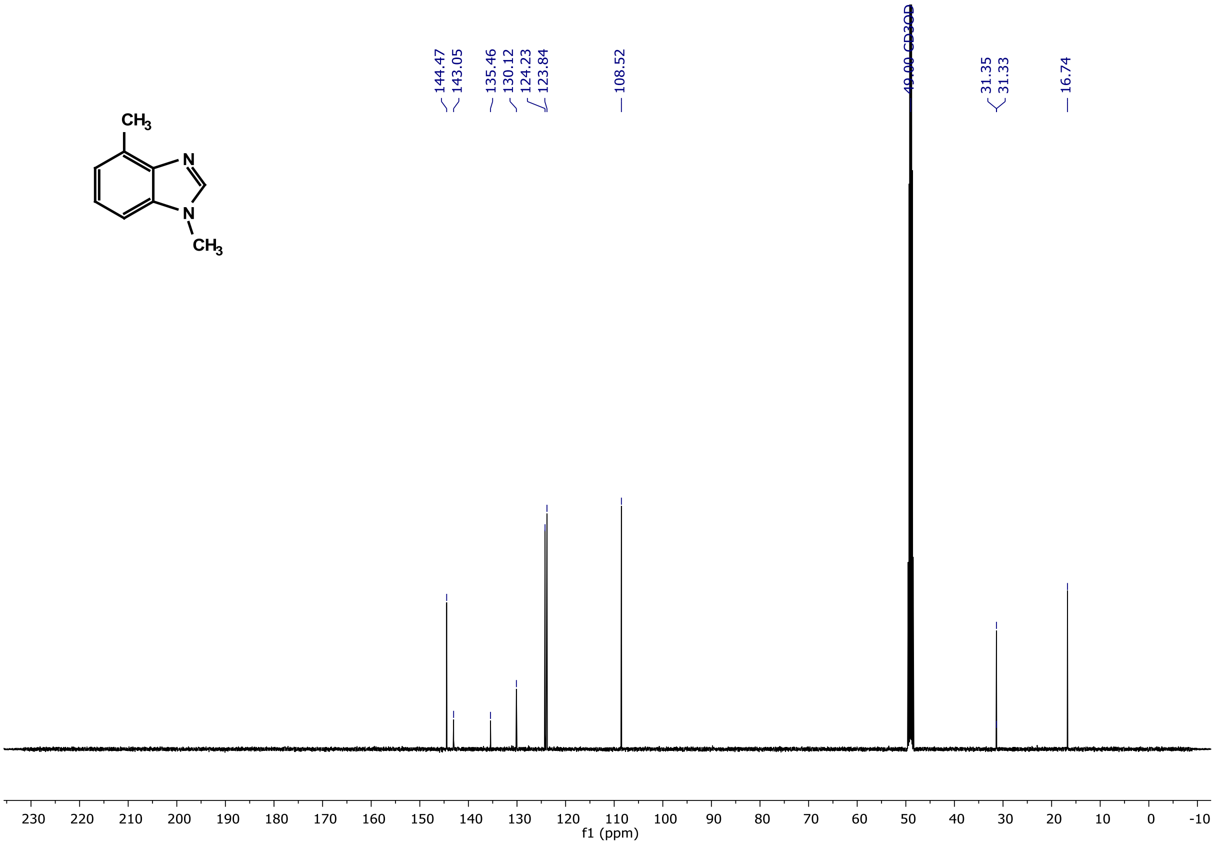

4-Methoxy-1-methyl-1*H*-benzo[*d*]imidazole (**2aa**)

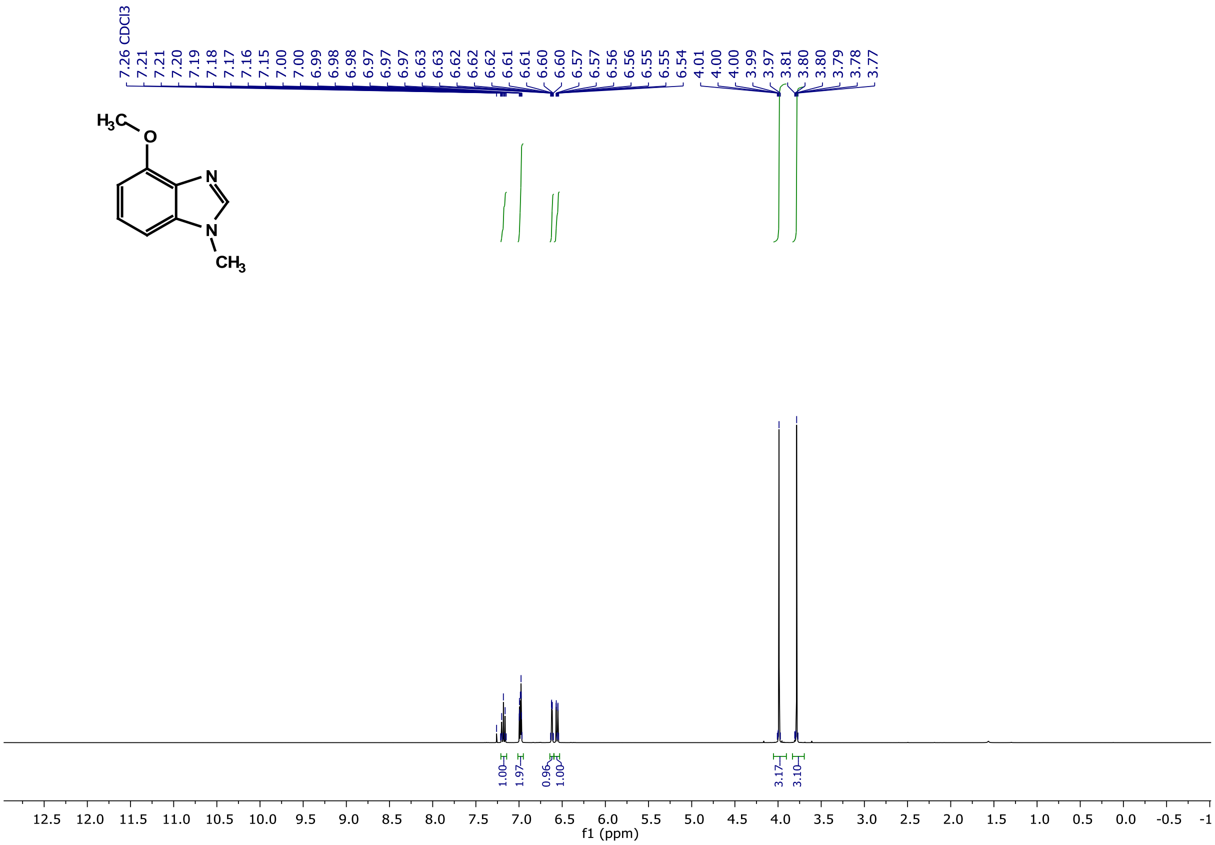

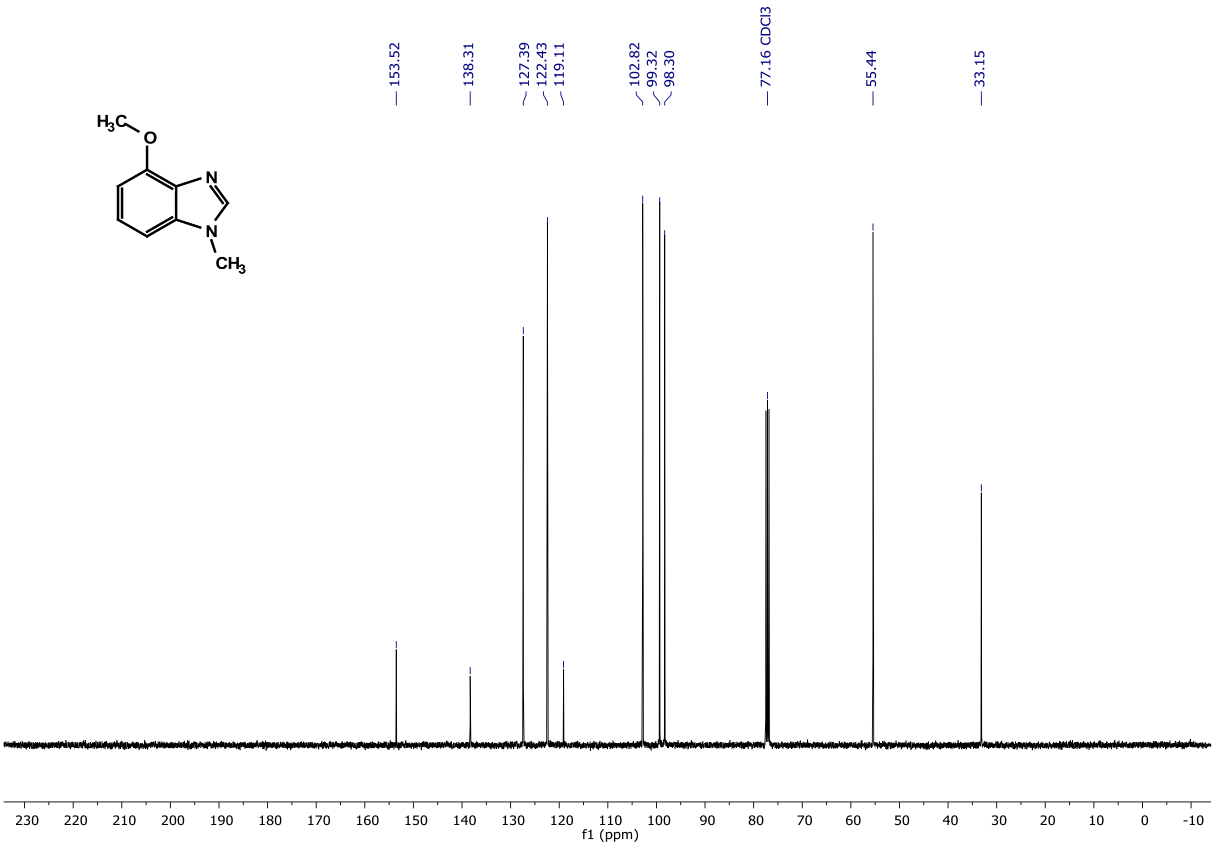

7-Methoxy-1-methyl-1*H*-benzo[*d*]imidazole (**2ab**)

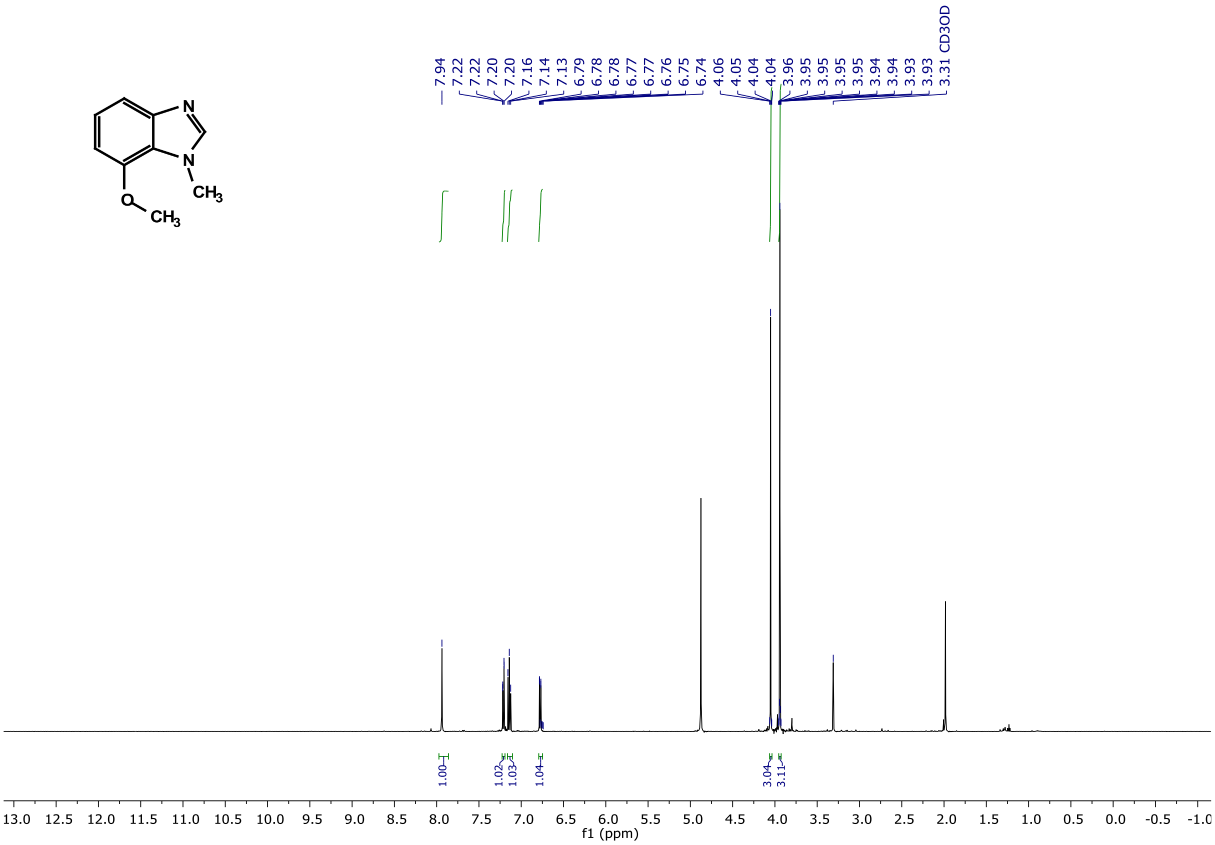

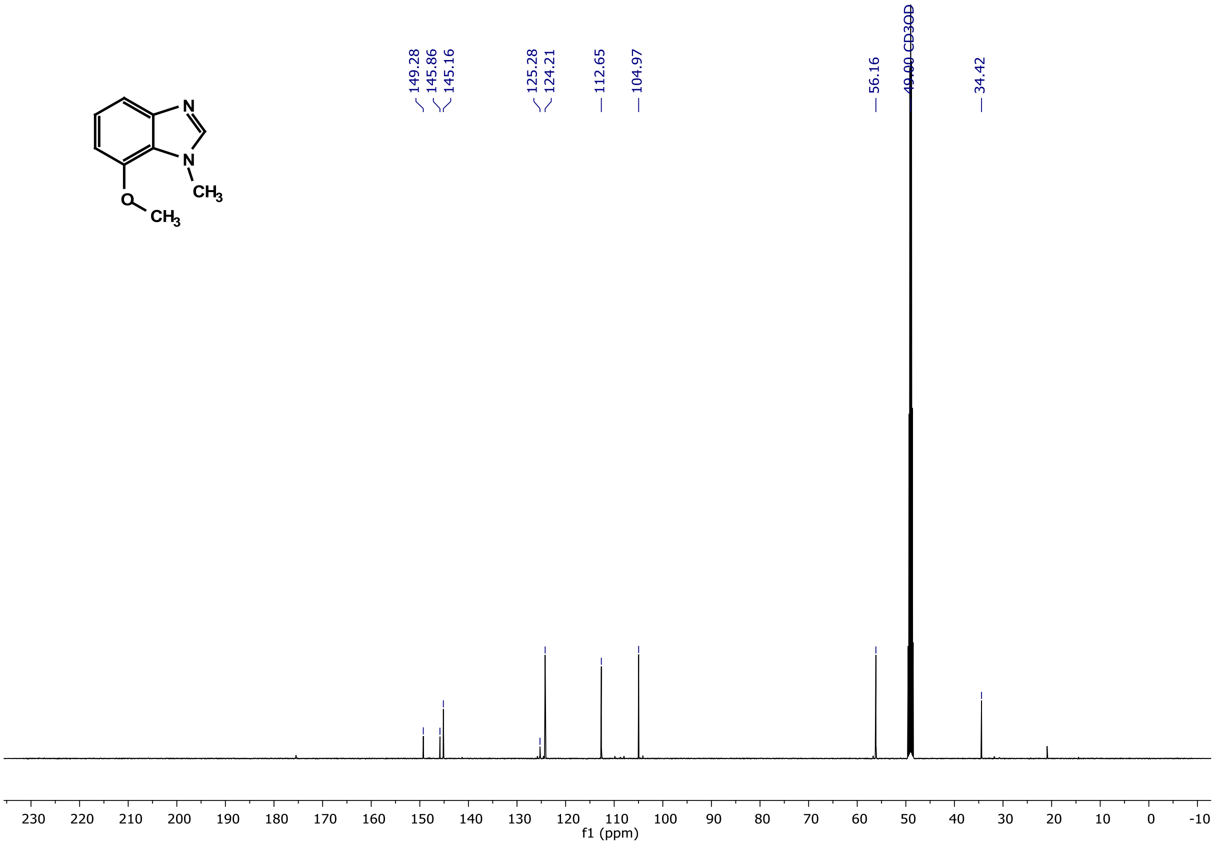

1,7-Dimethyl-1*H*-benzo[*d*]imidazole (**2ac**)

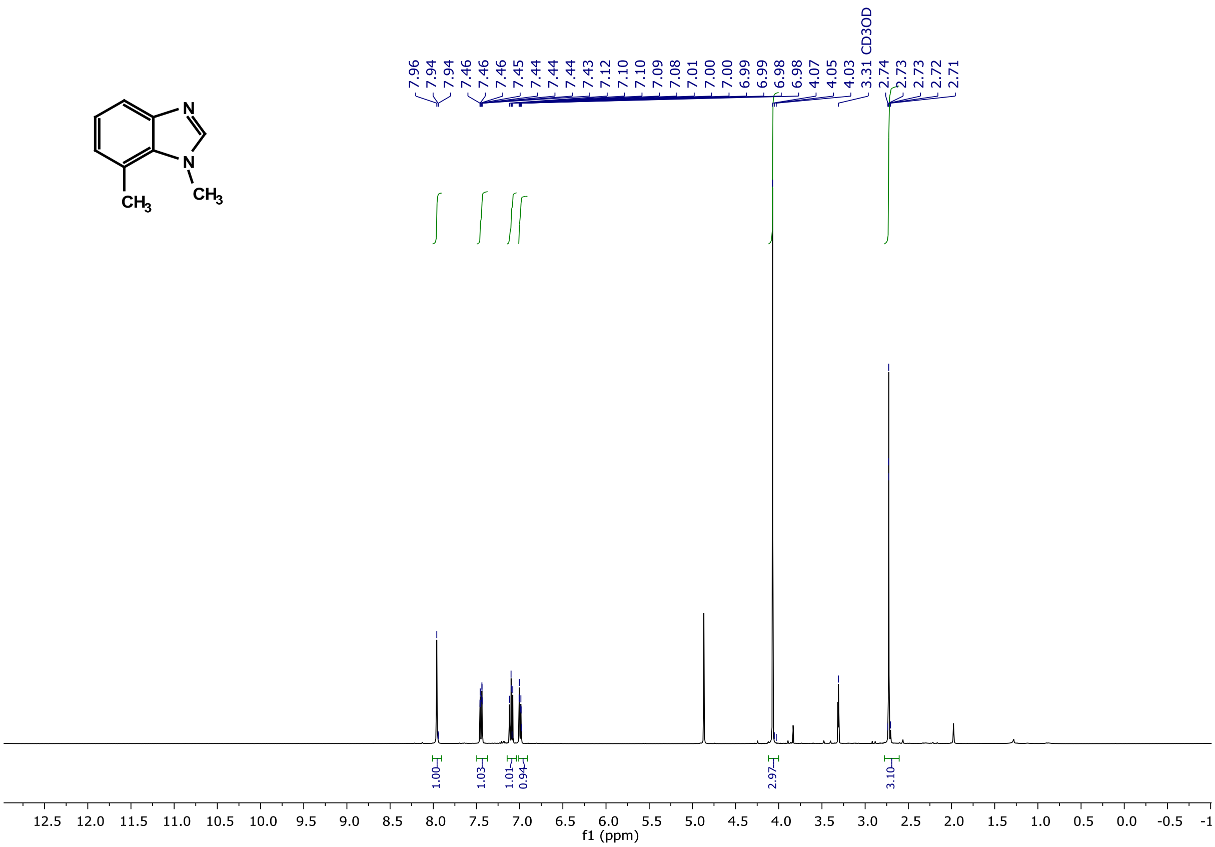

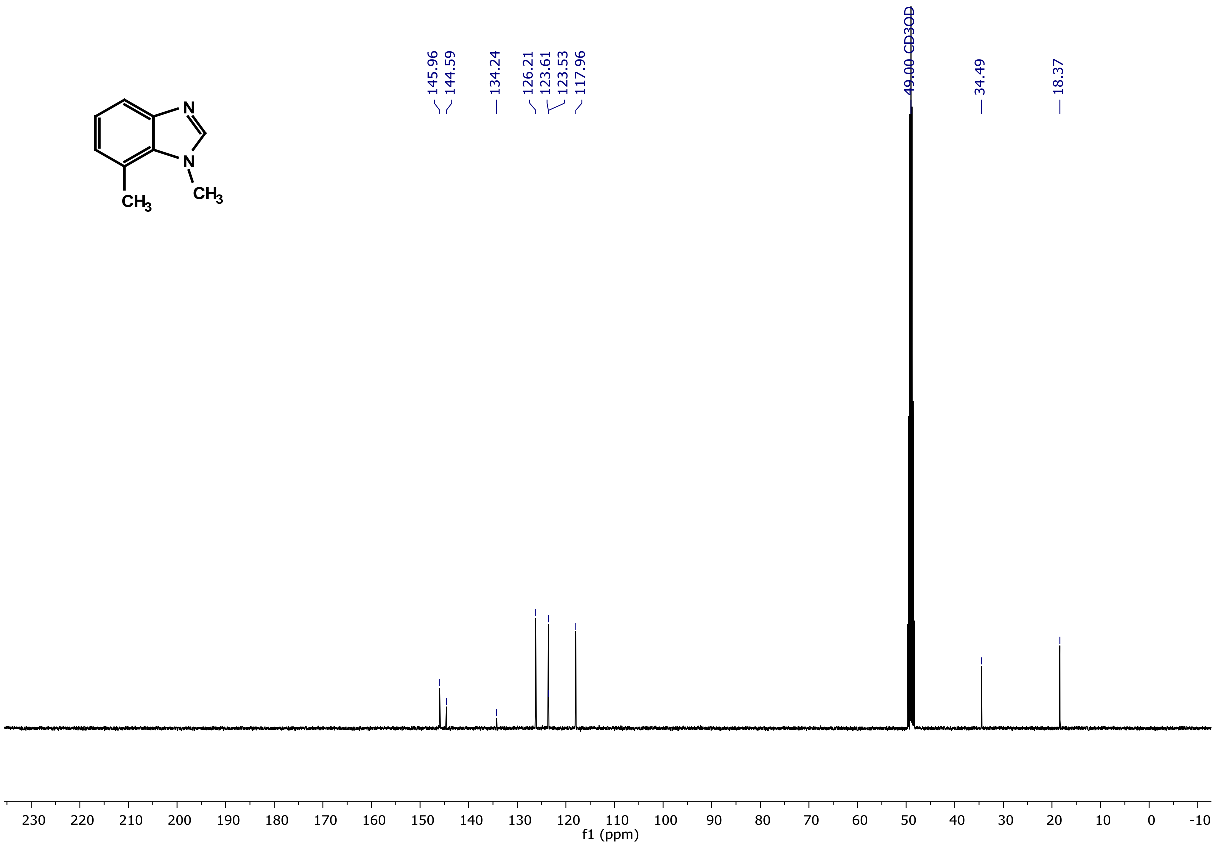

6-Methoxy-1-methyl-1*H*-imidazo[*4,5-c*]pyridine (**2ad**)

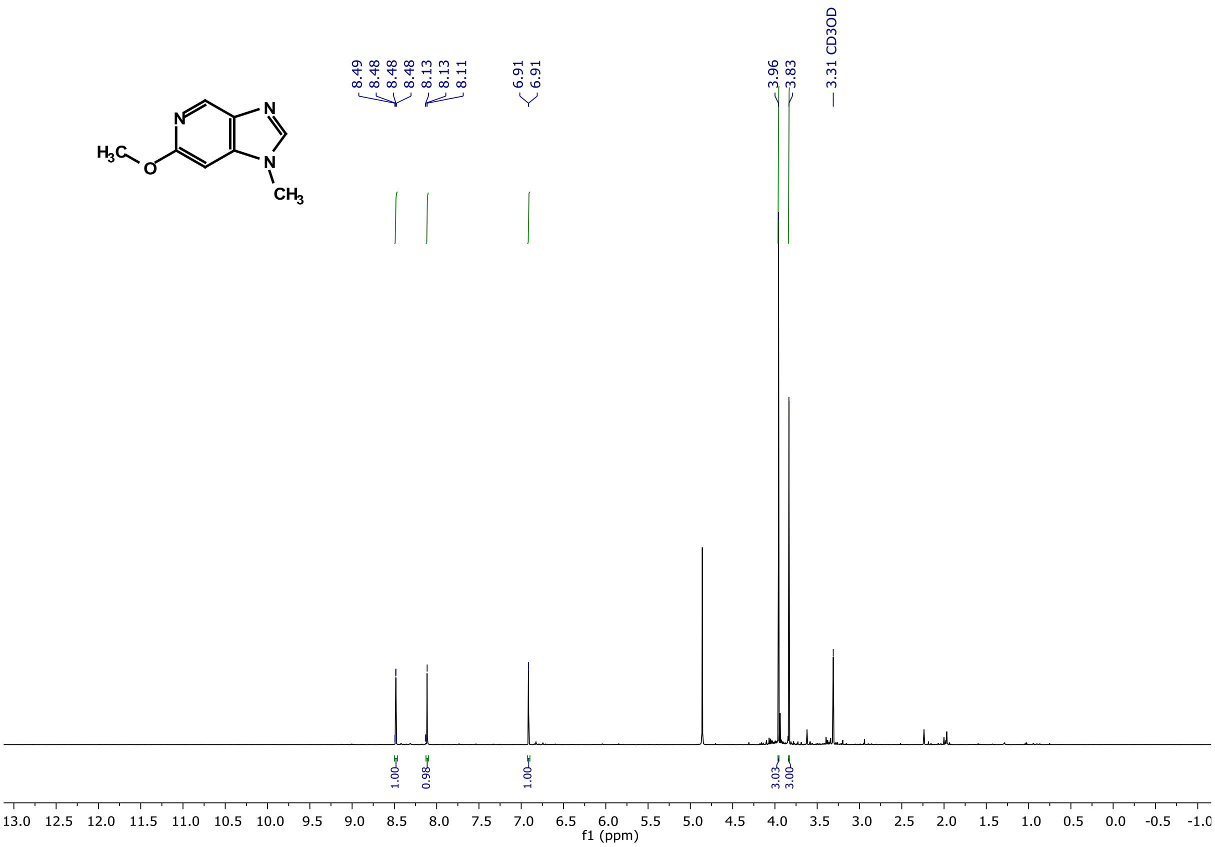

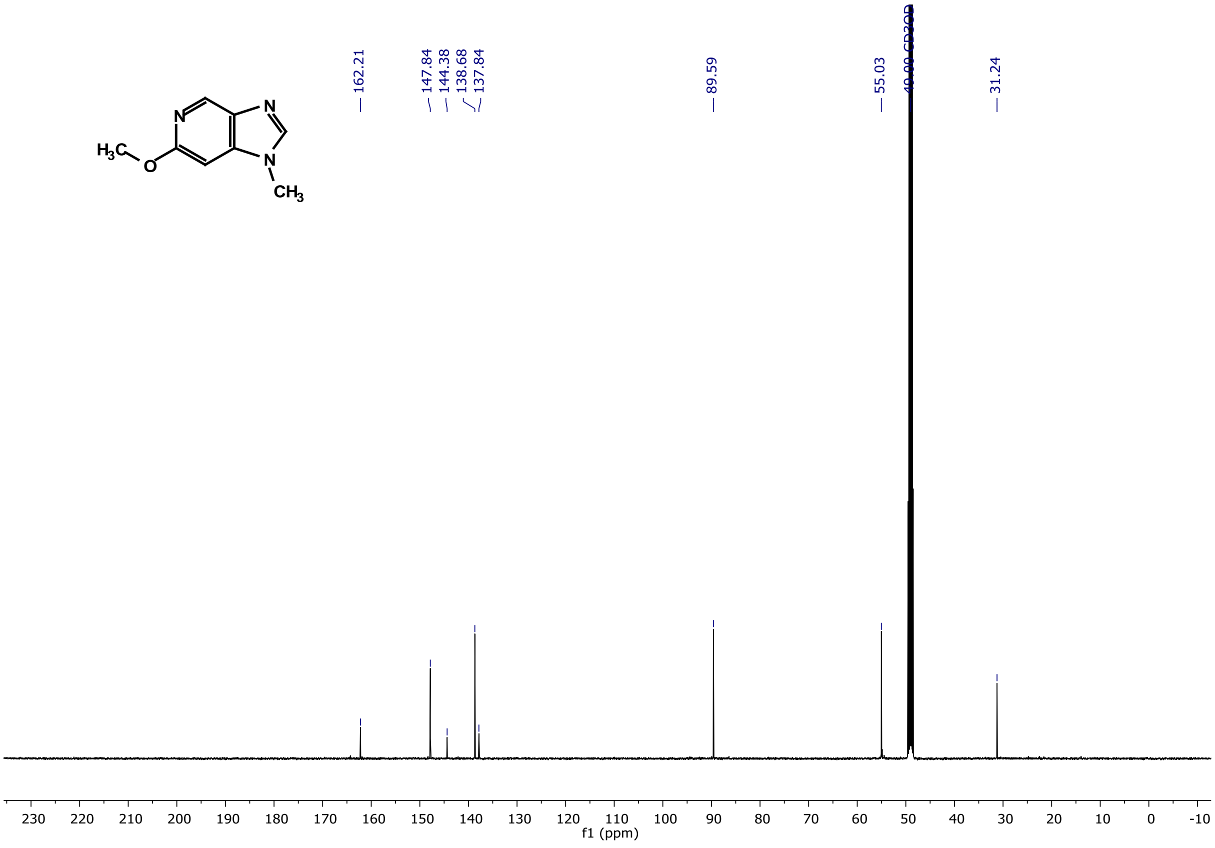

5-Methoxy-3-methyl-3*H*-imidazo[*4,5-b*]pyridine (**2ae**)

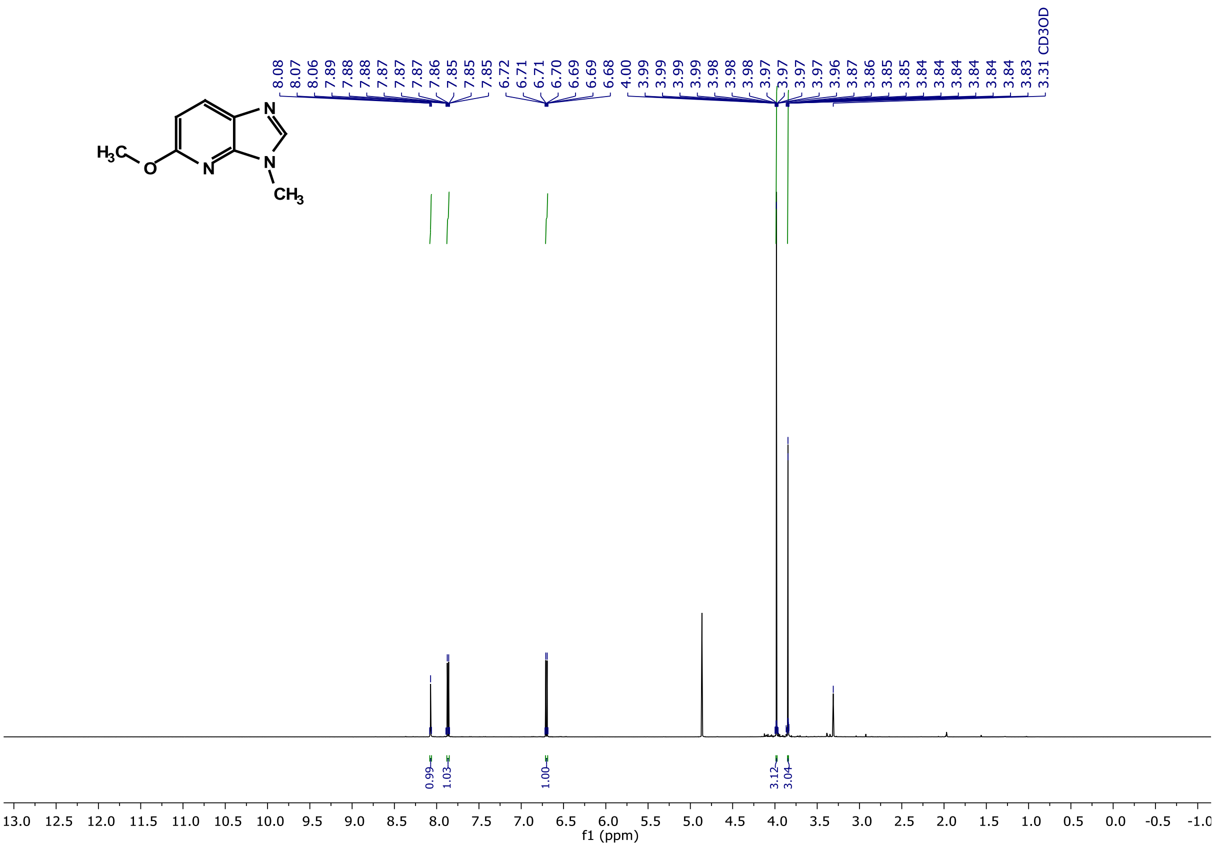

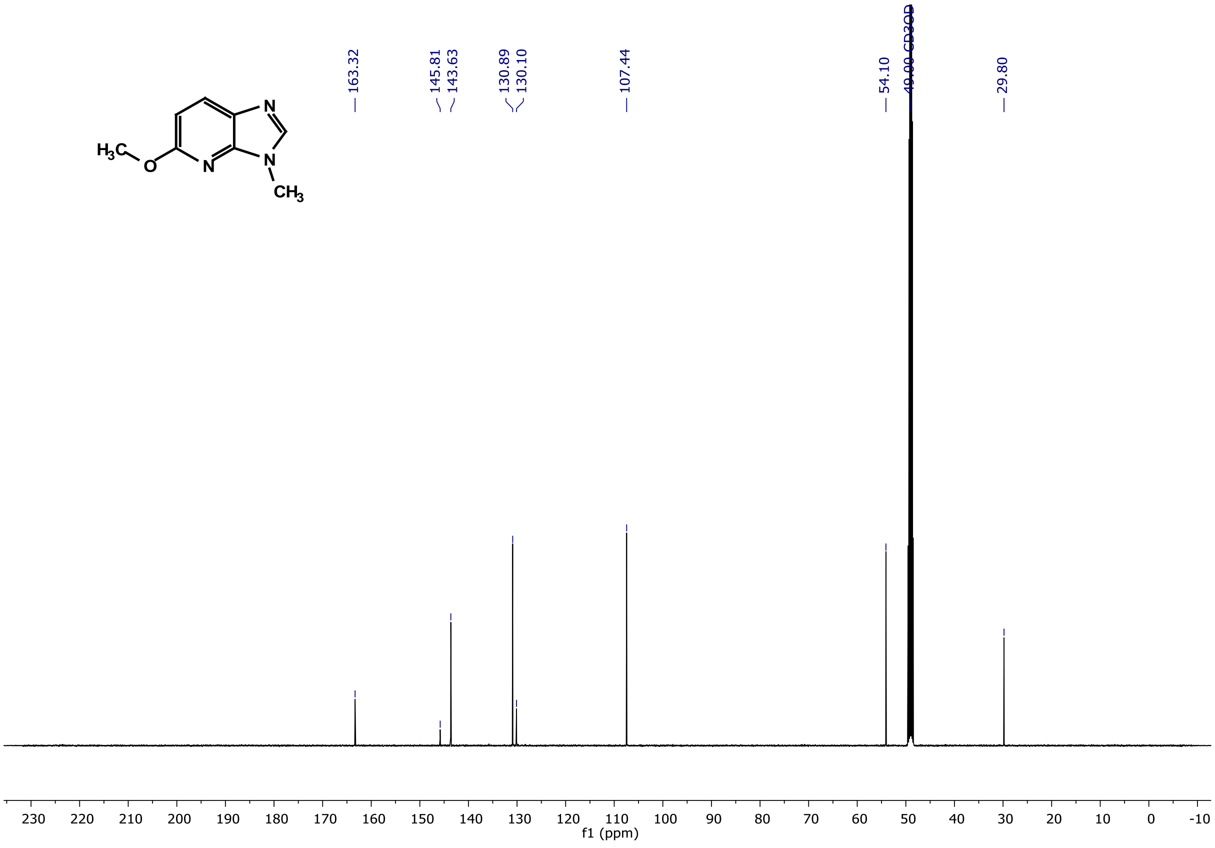

4-Methoxy-1-methyl-1*H*-imidazo[*4,5-c*]pyridine (**2af**)

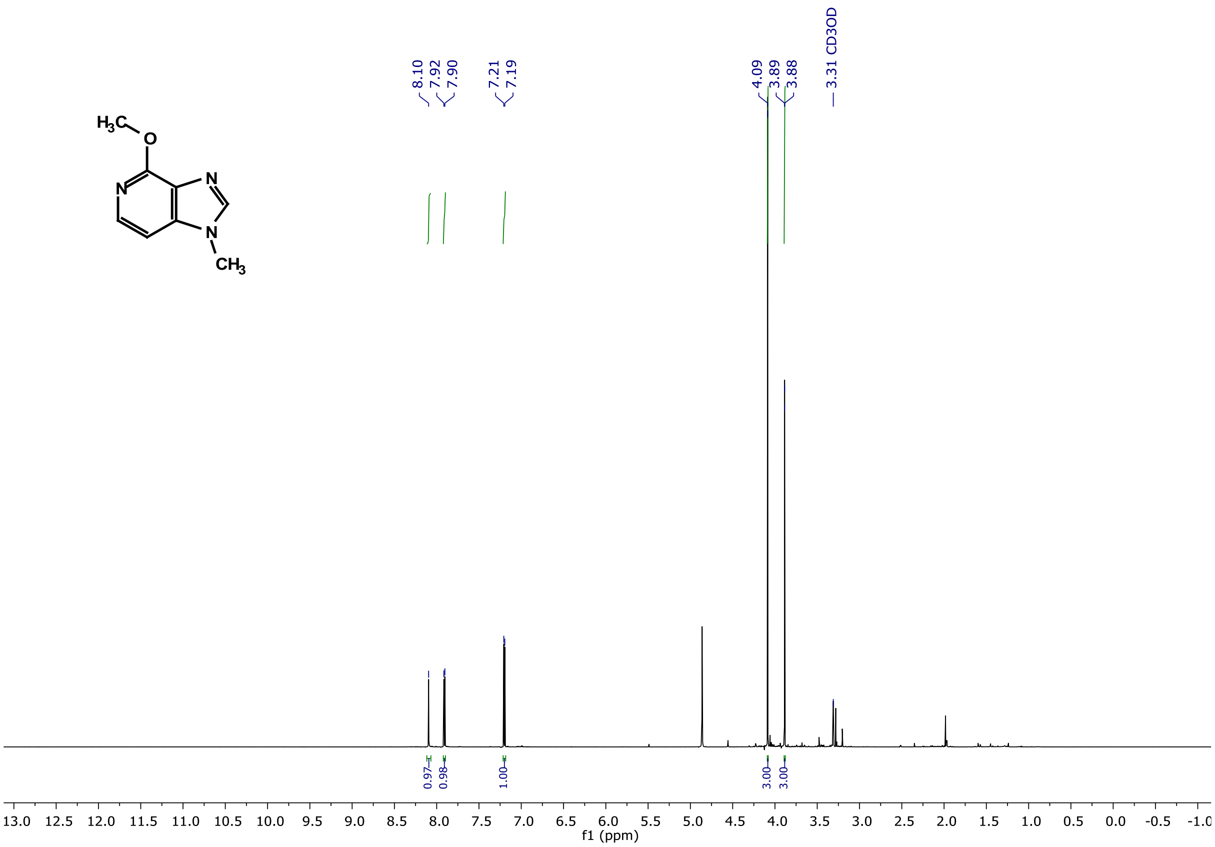

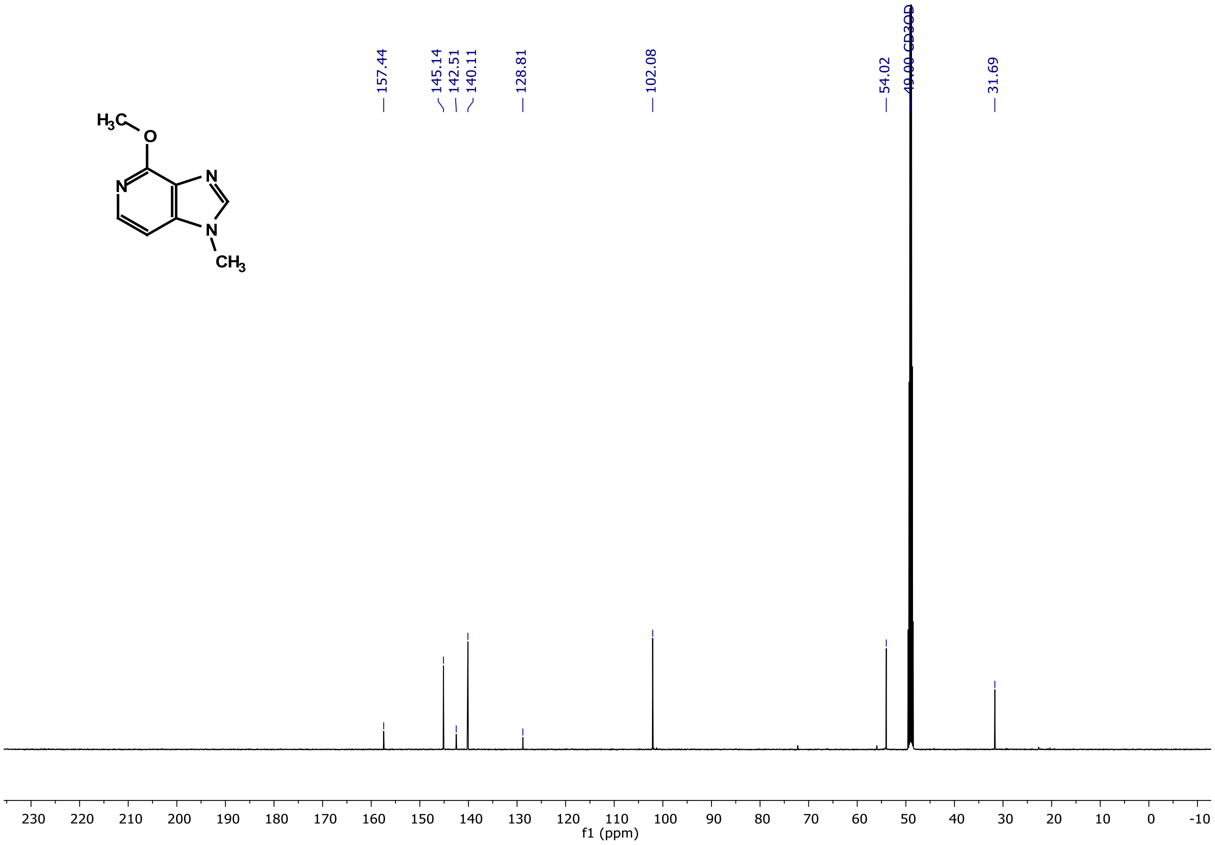

7-Chloro-5-(1-methyl-1*H*-benzo[*d*]imidazol-6-yl)quinoxaline (**2ag**)

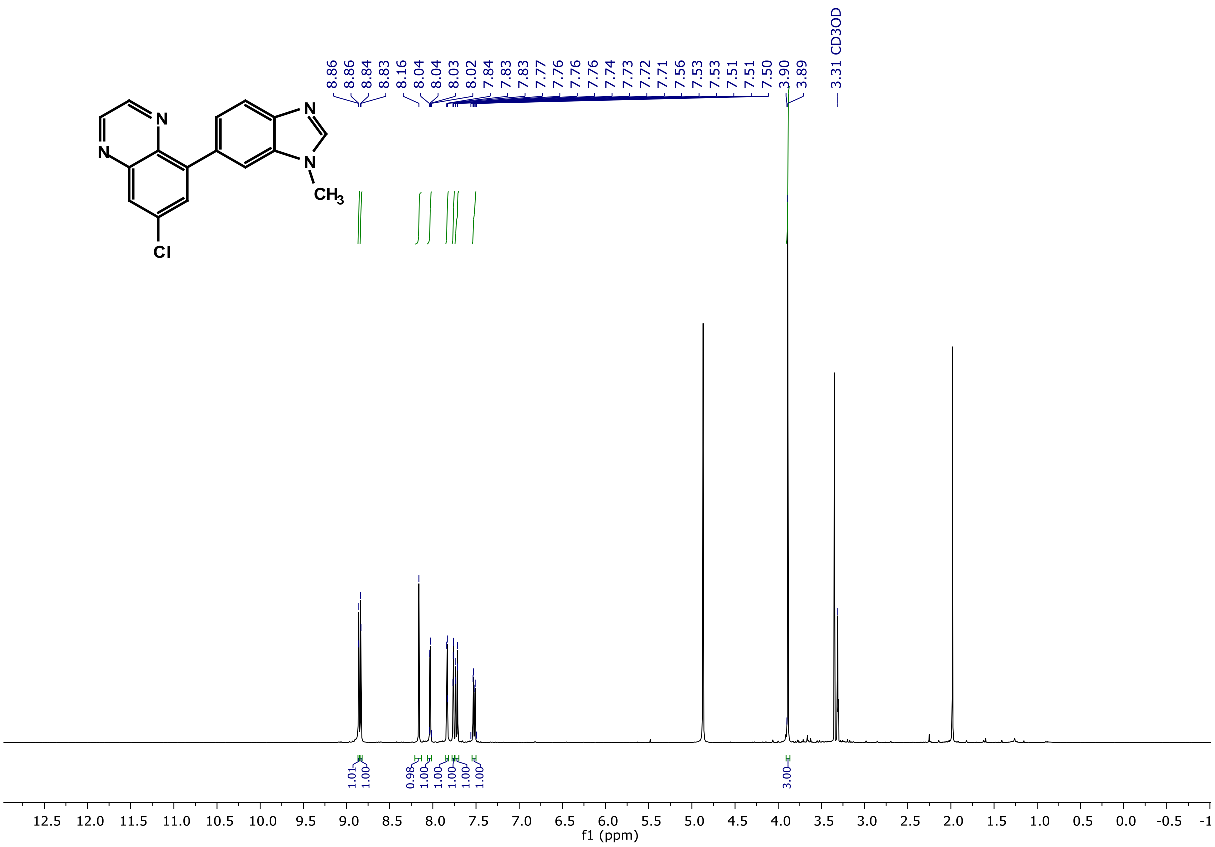

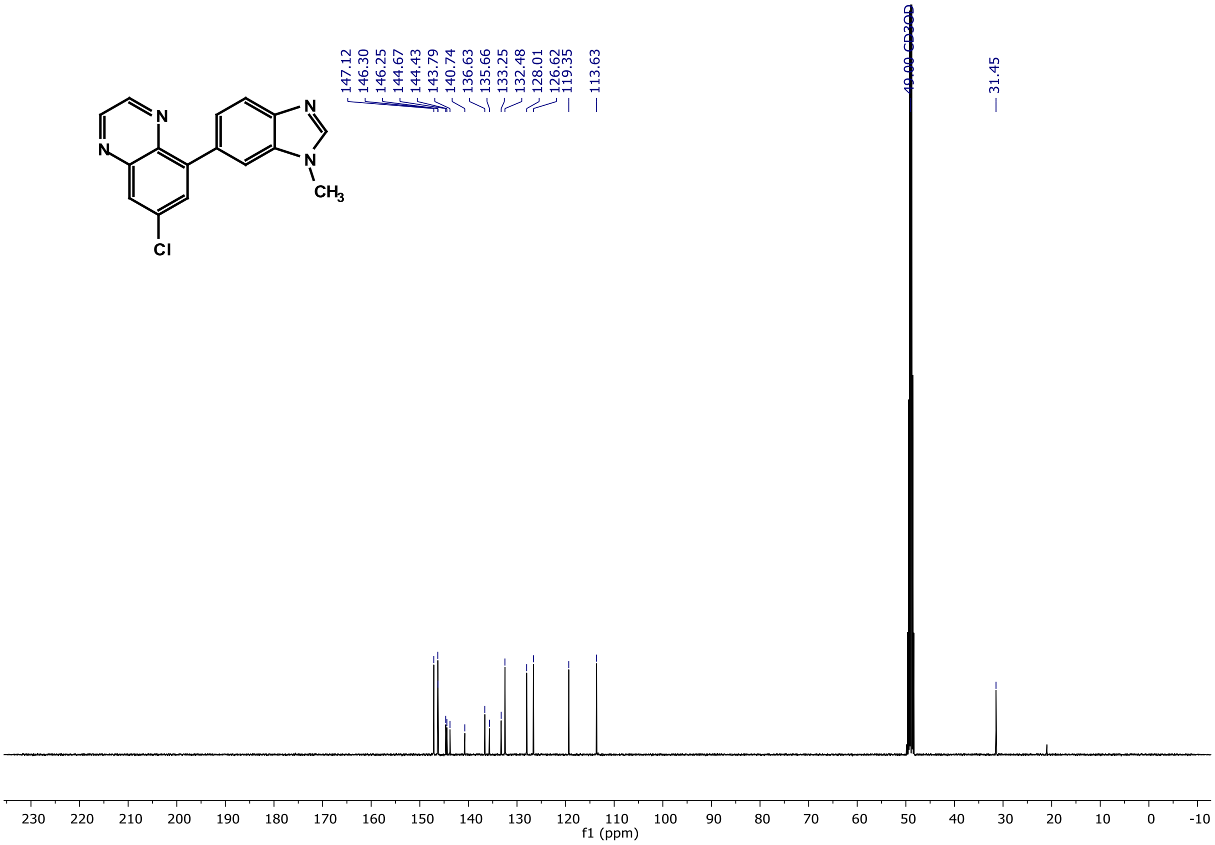

2-(1-Methyl-1*H*-benzo[*d*]imidazol-6-yl)isoindolin-1-one (**2ah**)

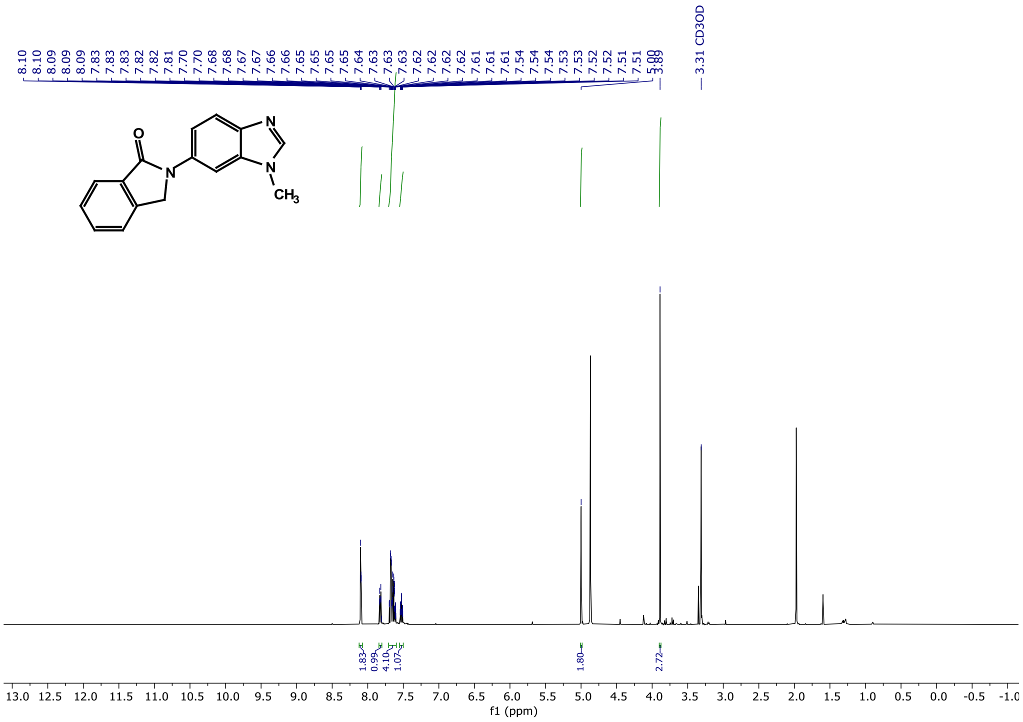

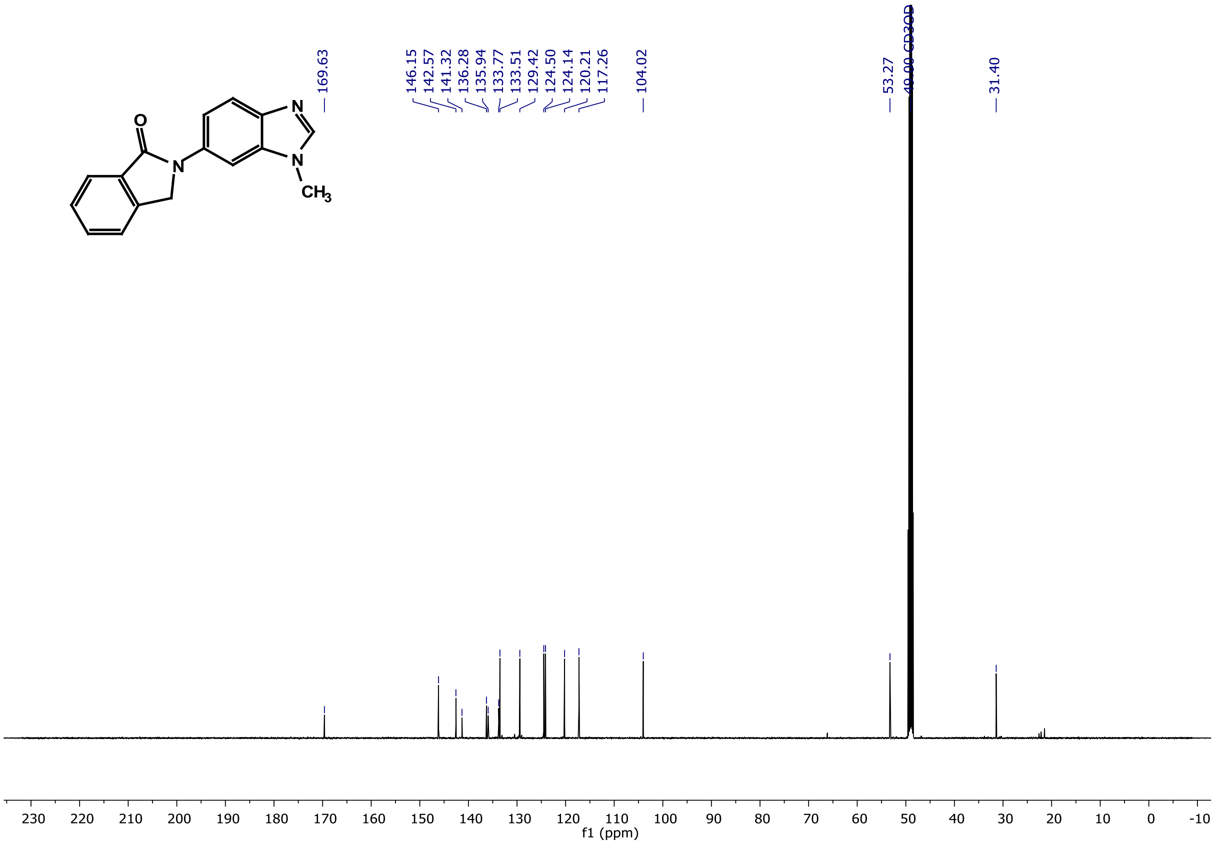

1-(1-Methyl-1*H*-benzo[*d*]imidazol-4-yl)-3-(pyridin-2-yl)urea (**2ai**)

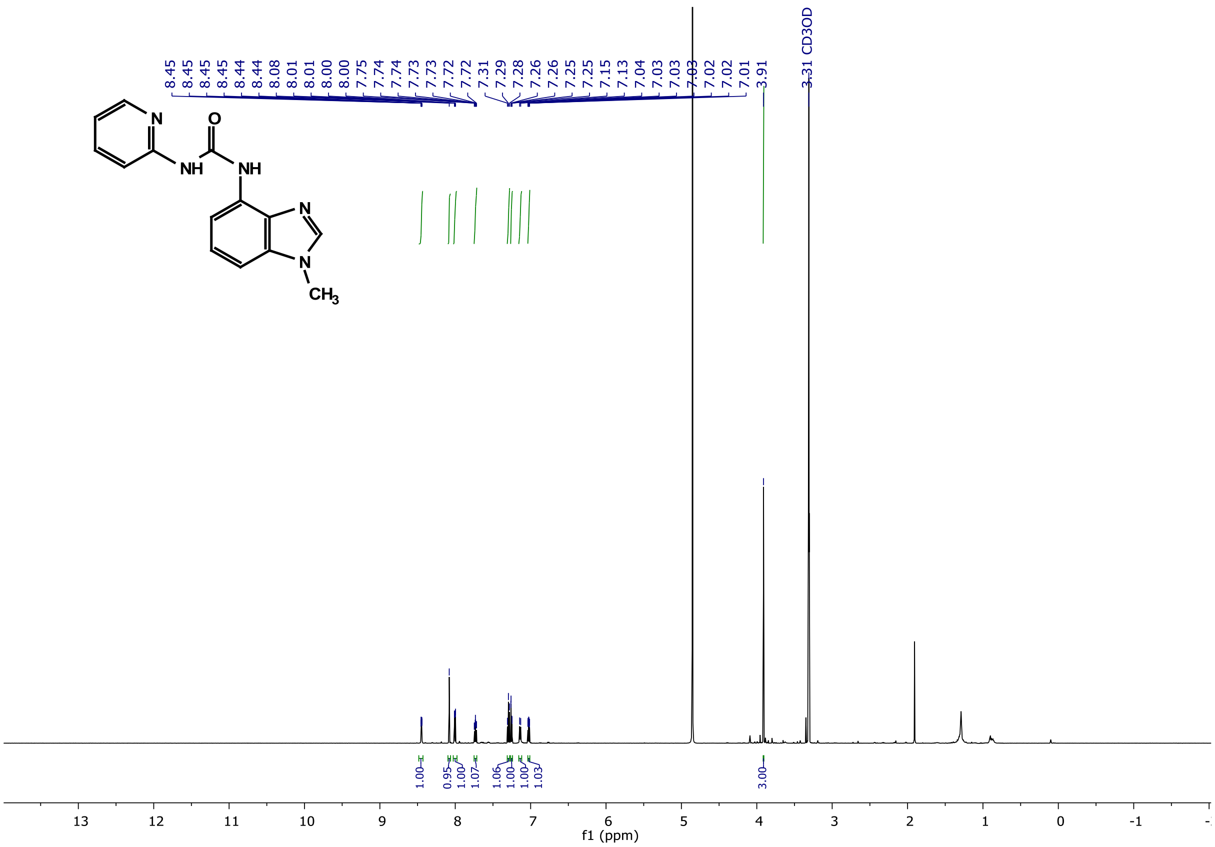

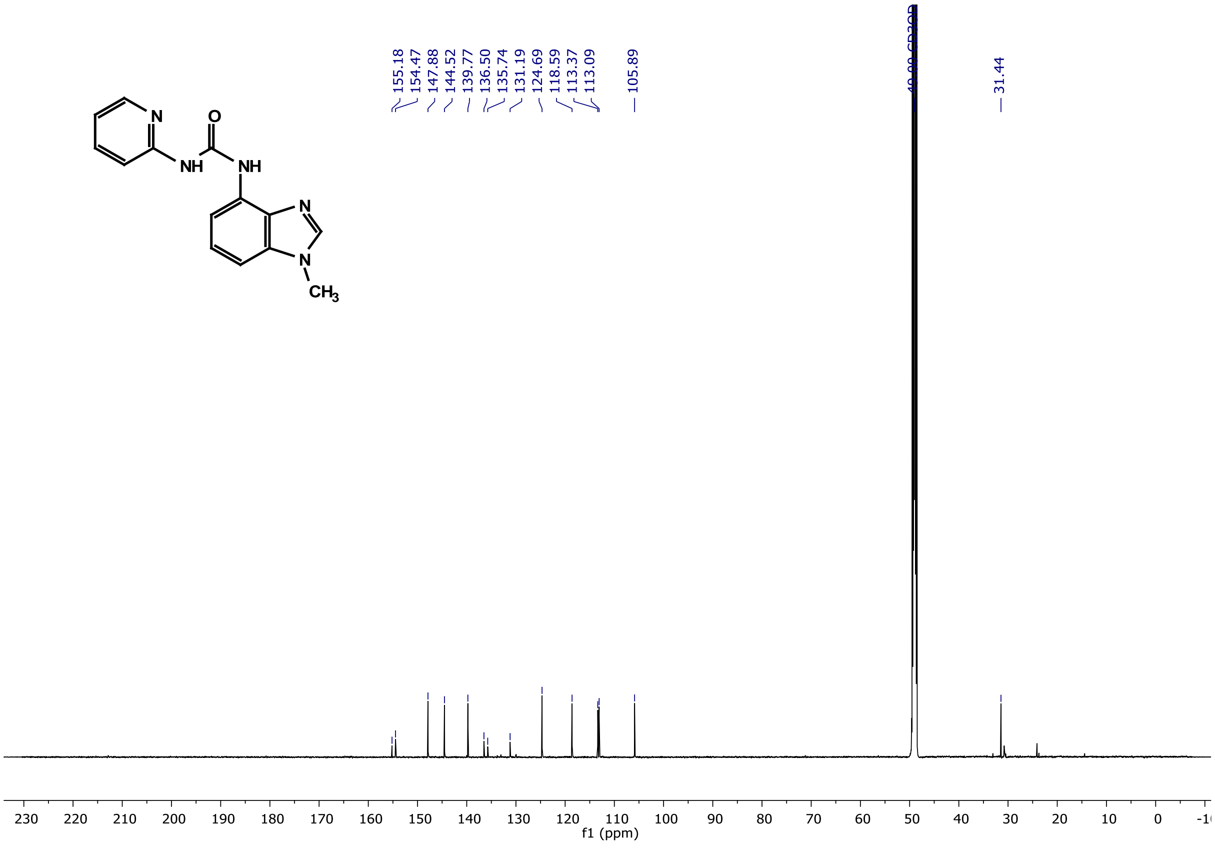

Methyl 4-(benzyloxy)-1-methyl-1*H*-benzo[*d*]imidazole-6-carboxylate (**2aj**)

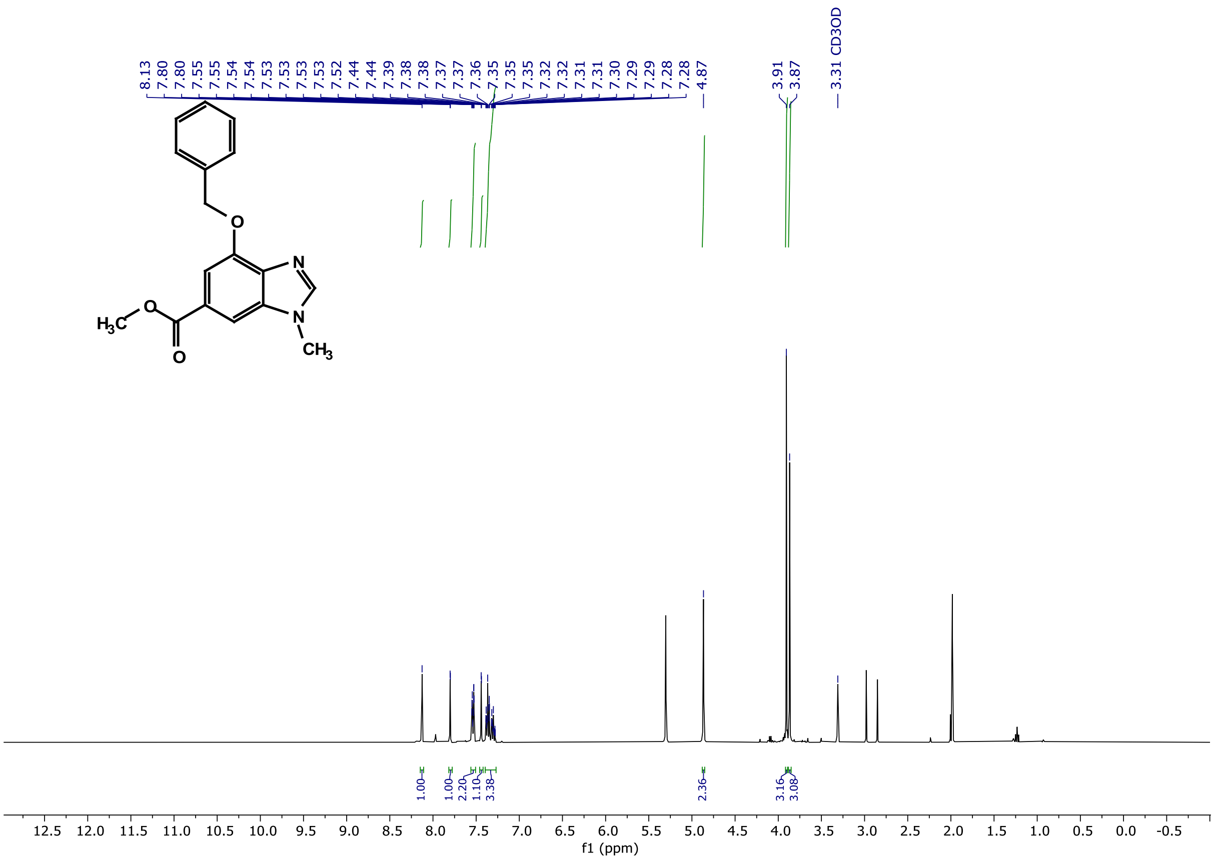

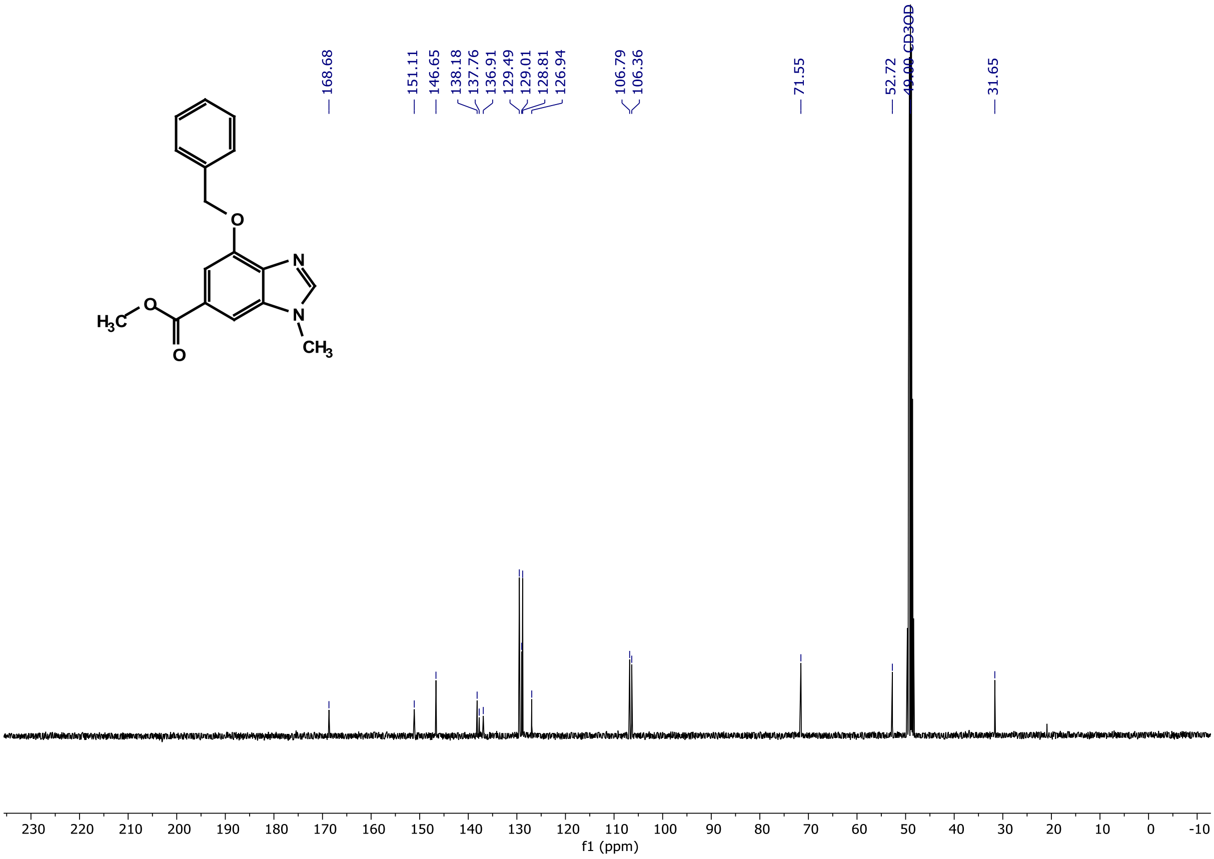

Methyl 4-(benzyloxy)-1-ethyl-1*H*-benzo[*d*]imidazole-6-carboxylate (**2ak**)

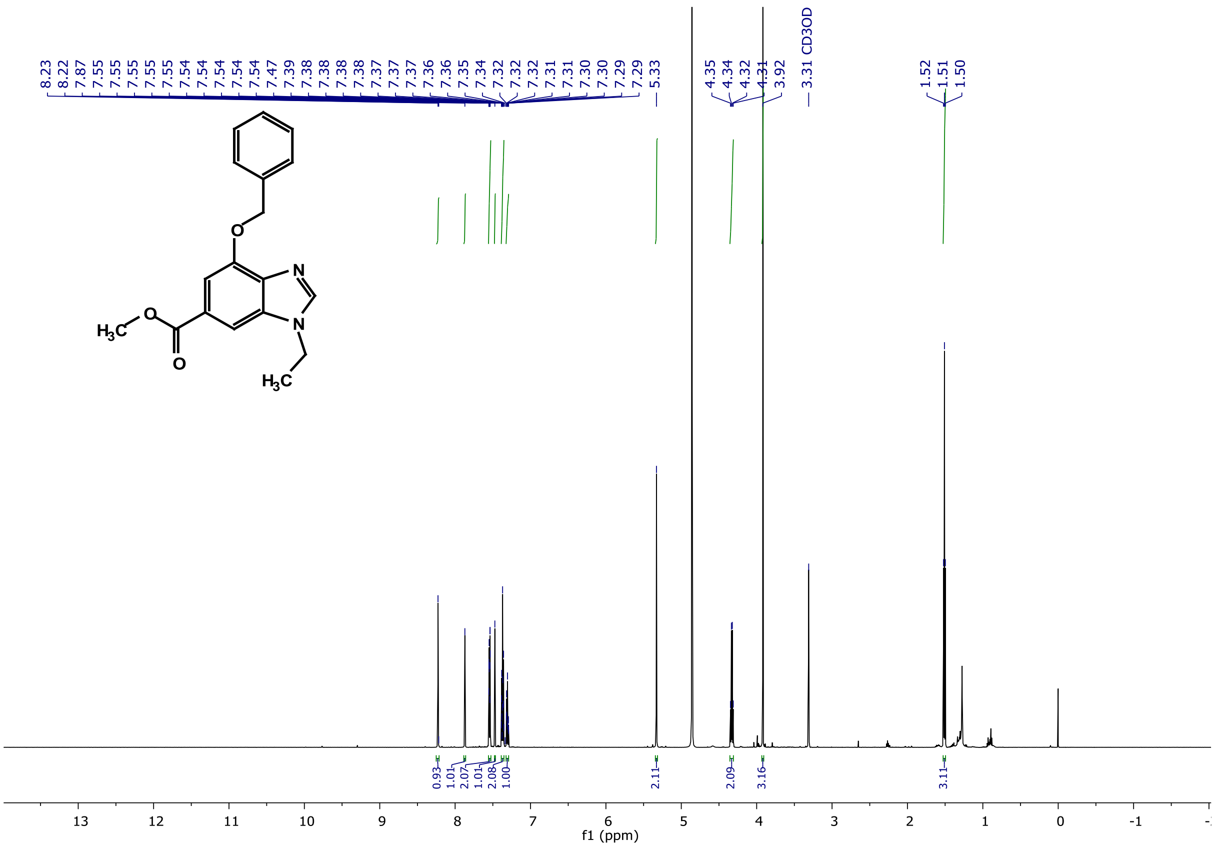

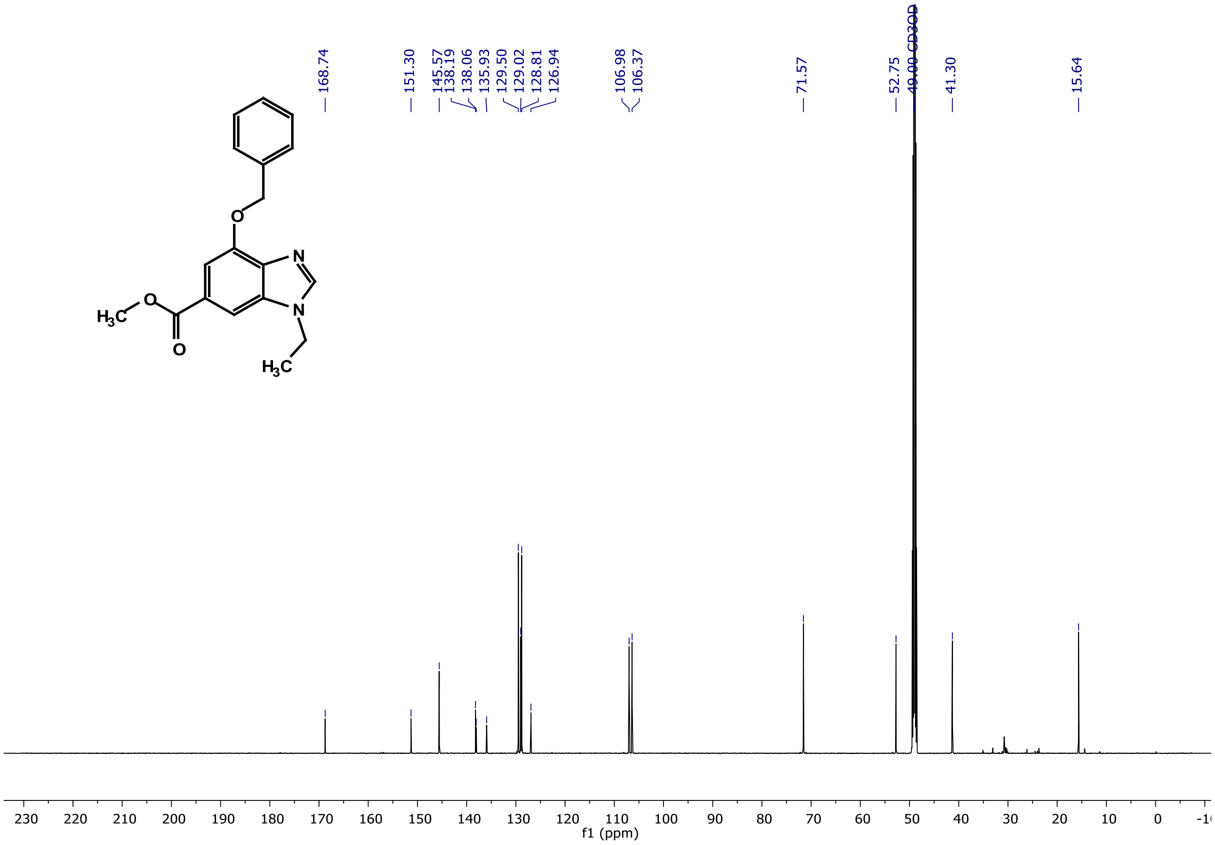

4-(Methyl(1-methyl-1*H*-benzo[*d*]imidazol-7-yl)carbamoyl)phenyl diethylcarbamate (**2al**)

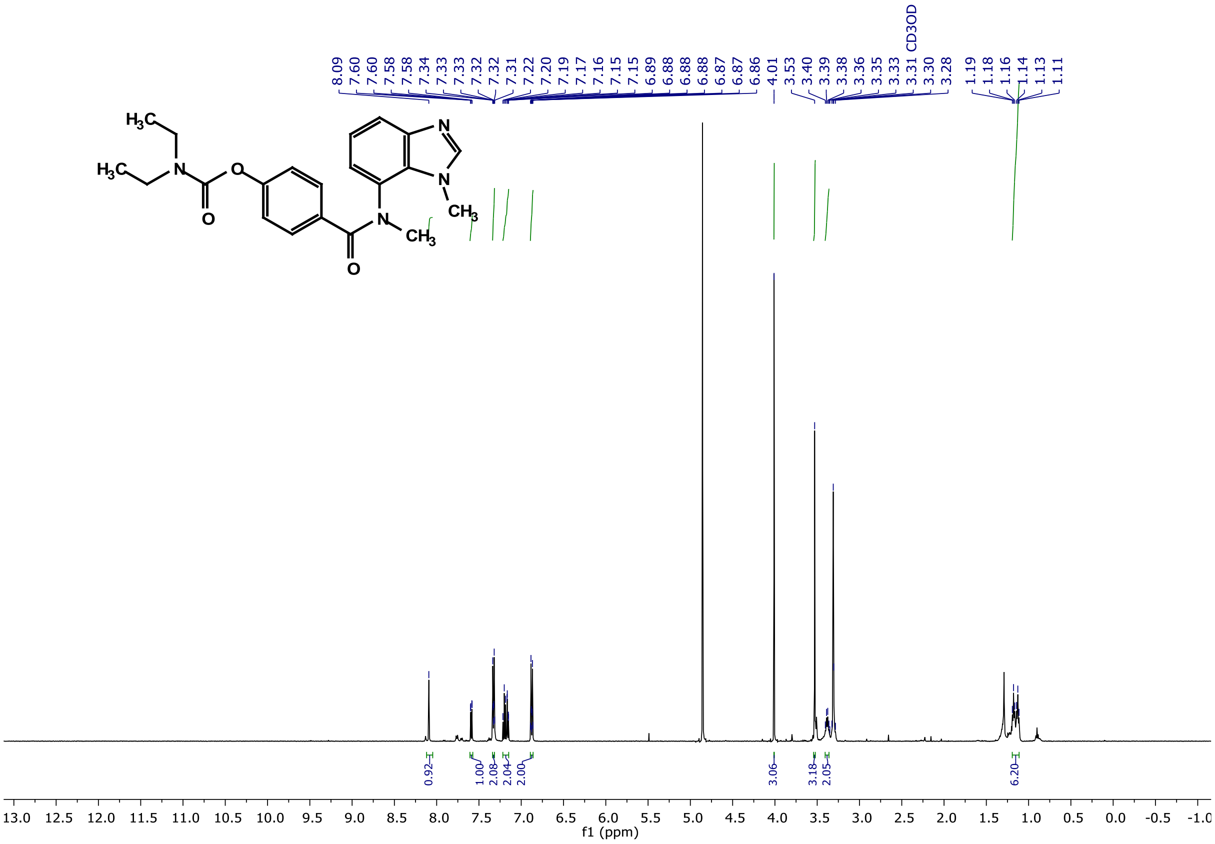

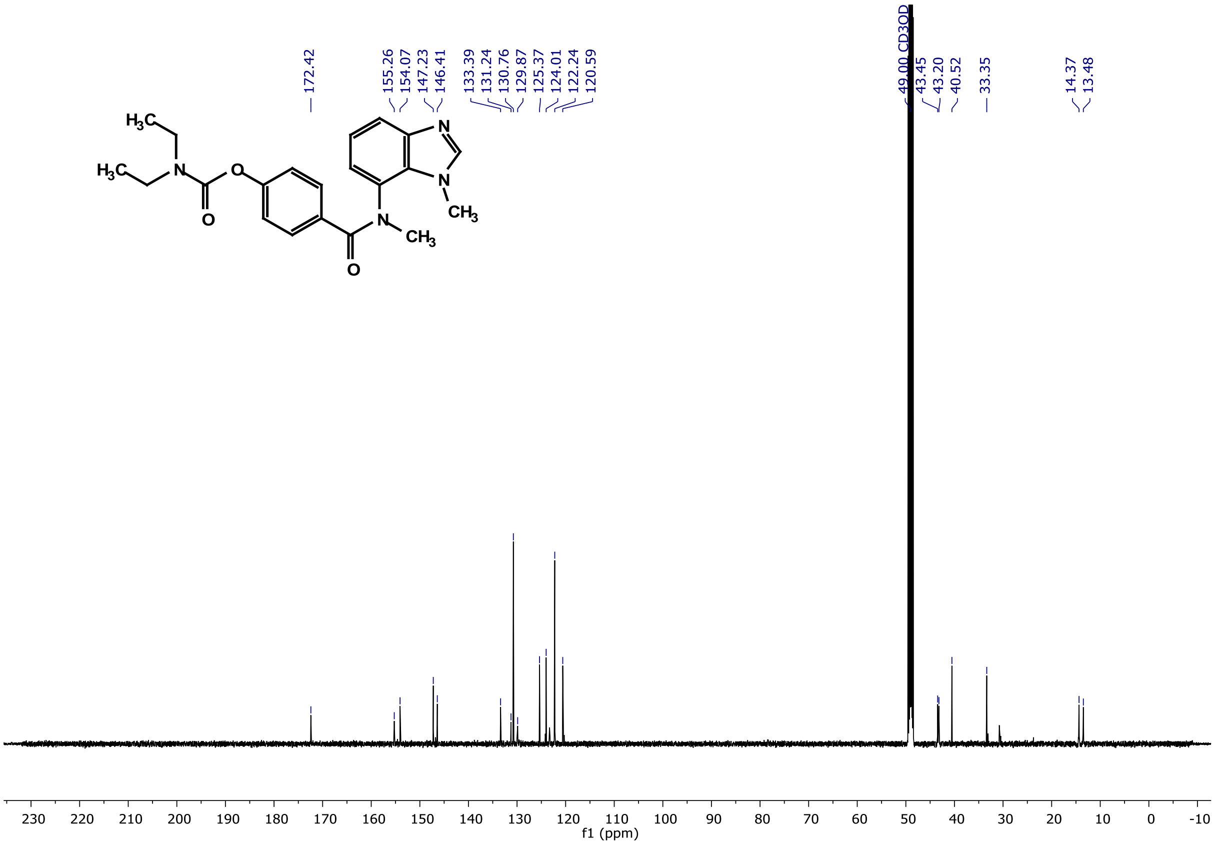

3-(1*H*-benzo[*d*]imidazol-1-yl)propanamide (**2am**)

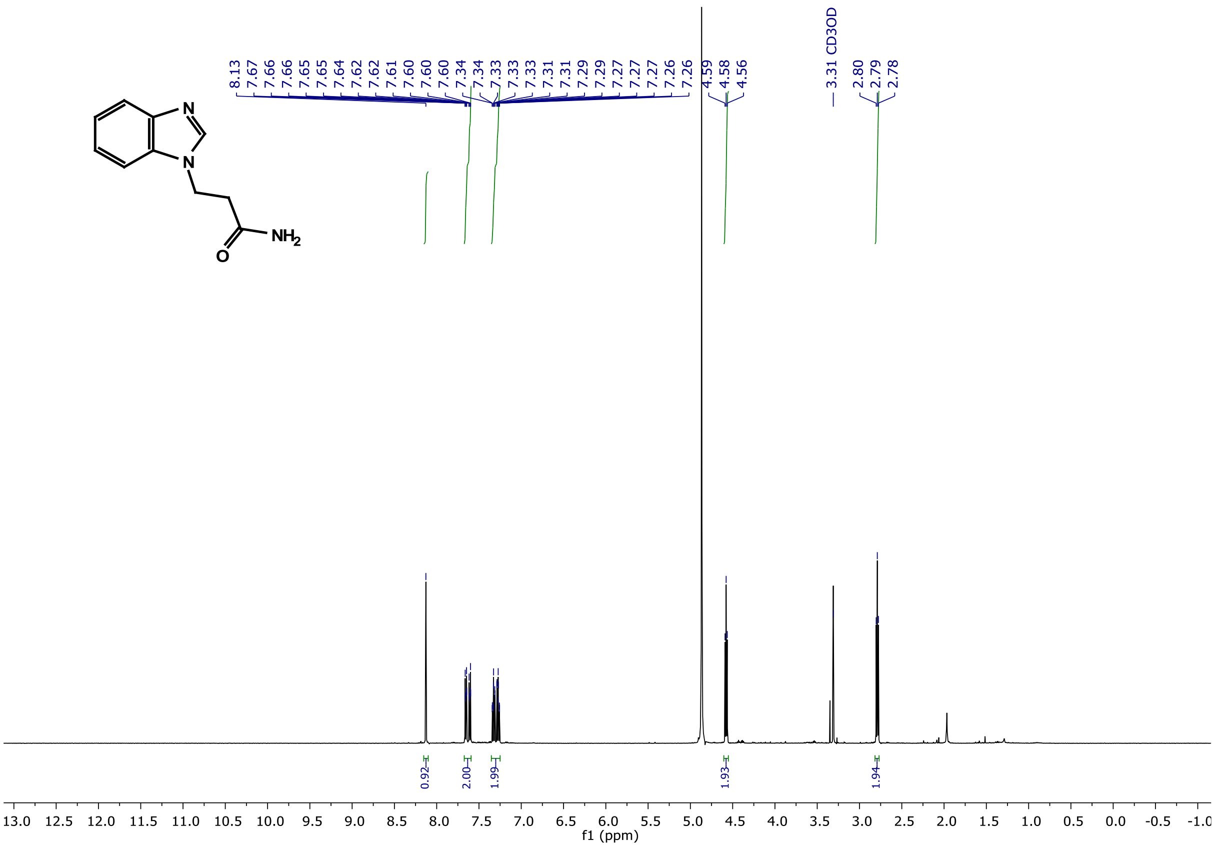

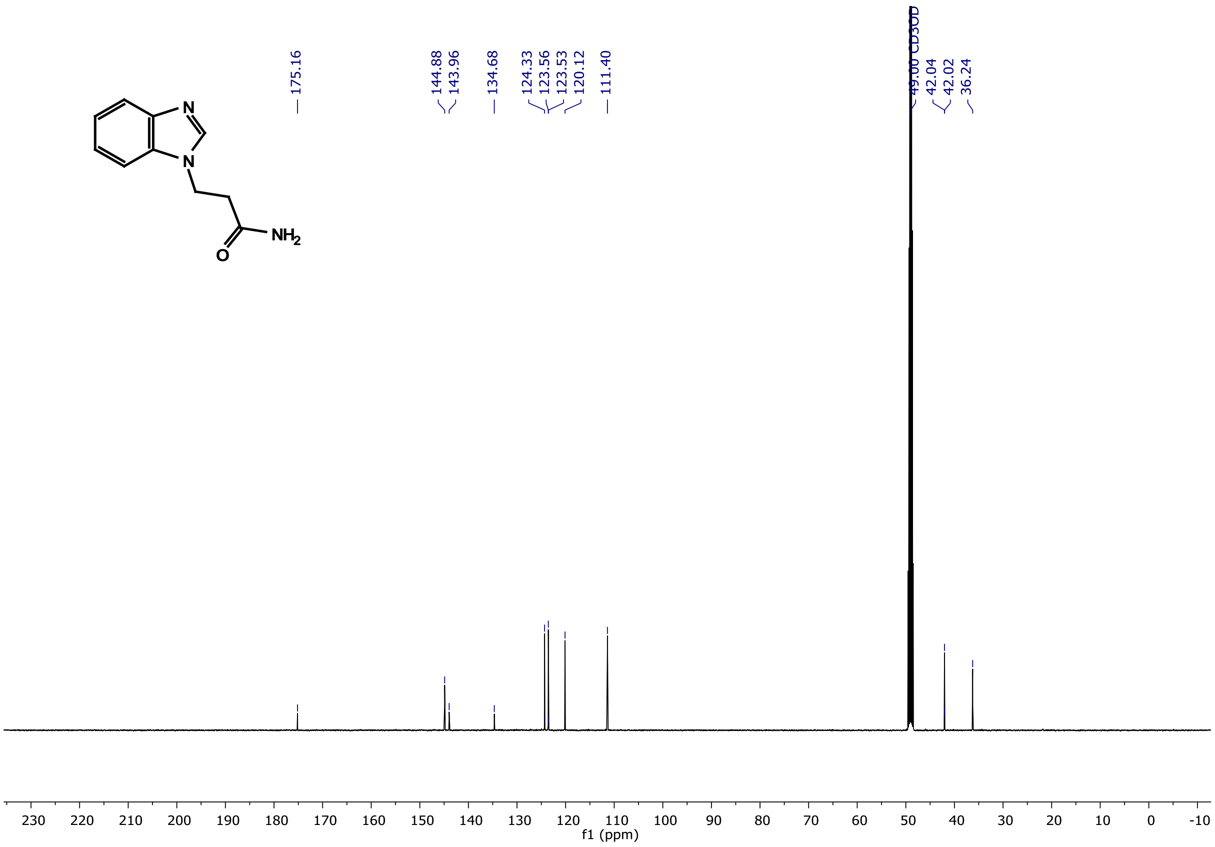

2-(6-(2-(4-Methyl-2-(4-(trifluoromethyl)phenyl)thiazol-5-yl)ethoxy)-1*H*-benzo[*d*]imidazol-1-yl)acetic acid (**2an**)

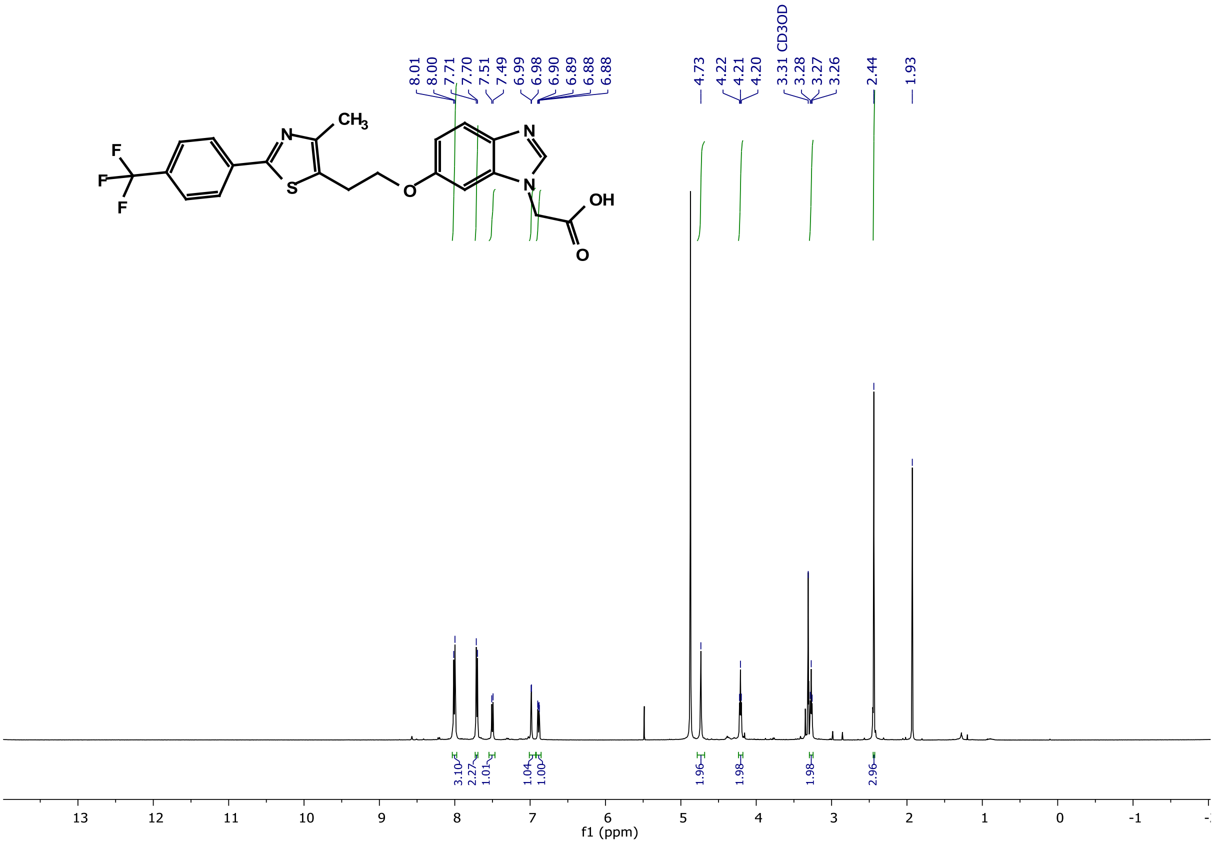

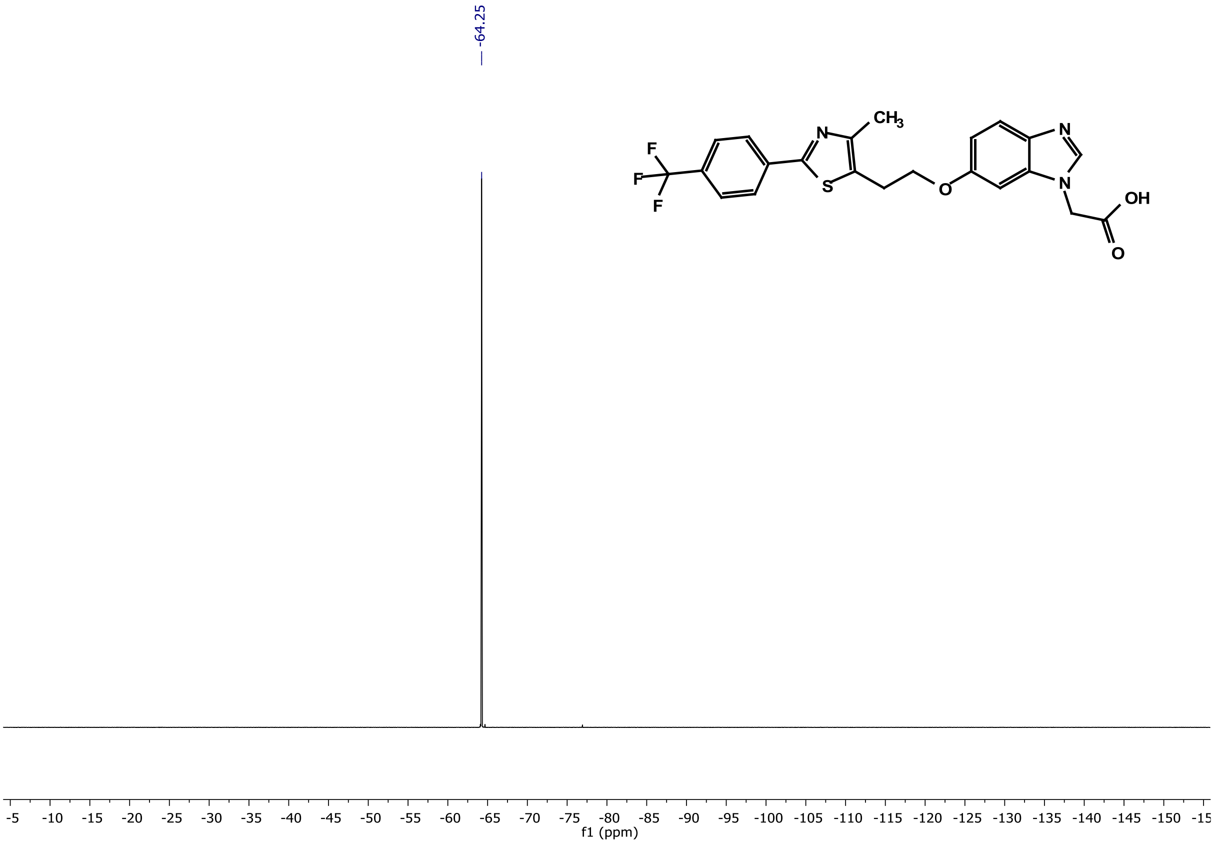

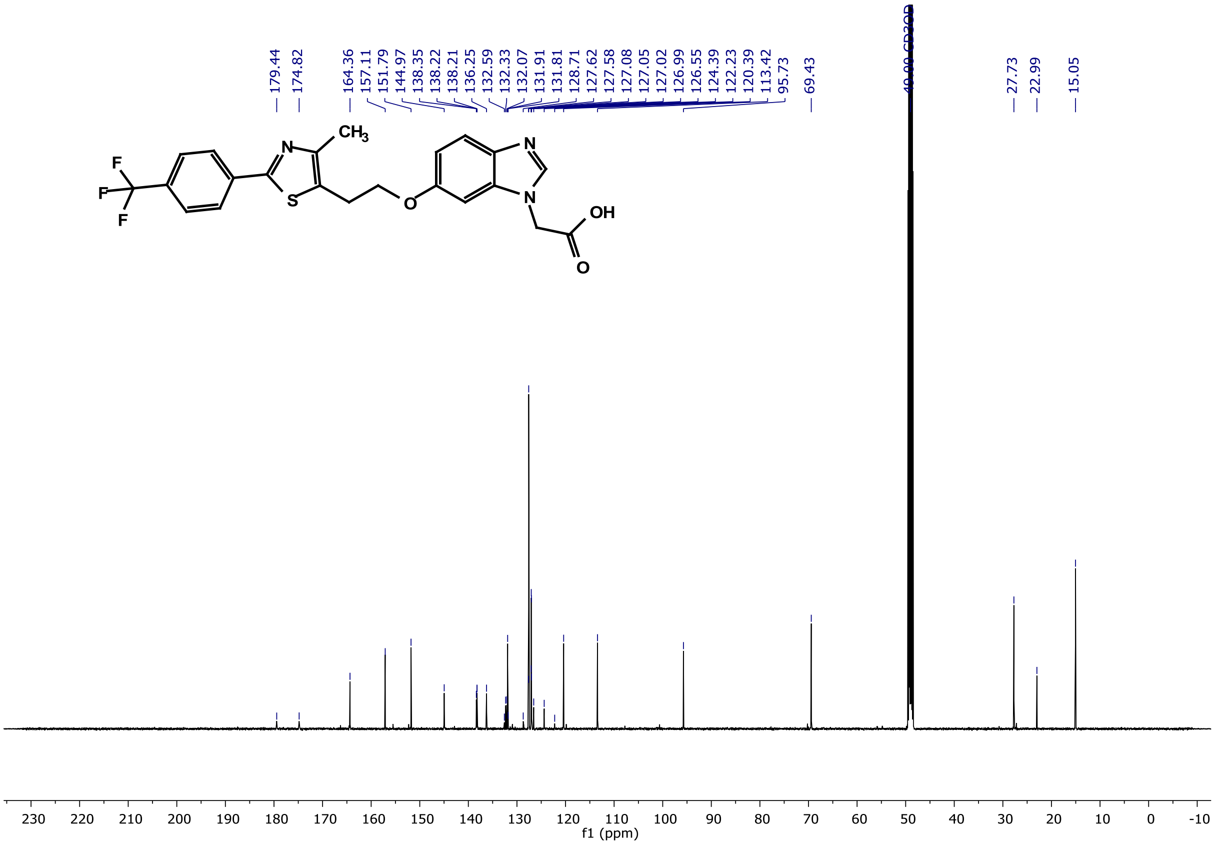
179.44
174.82
164.36
157.11
151.79
144.97
138.35
138.22
138.21
136.25
132.59
132.33
132.07
131.91
131.81
128.71
127.62
127.58
127.08
127.05
127.02
126.99
126.55
124.39
122.23
120.39
113.42
95.73
69.43
27.73
22.99
15.05
f1 (ppm)

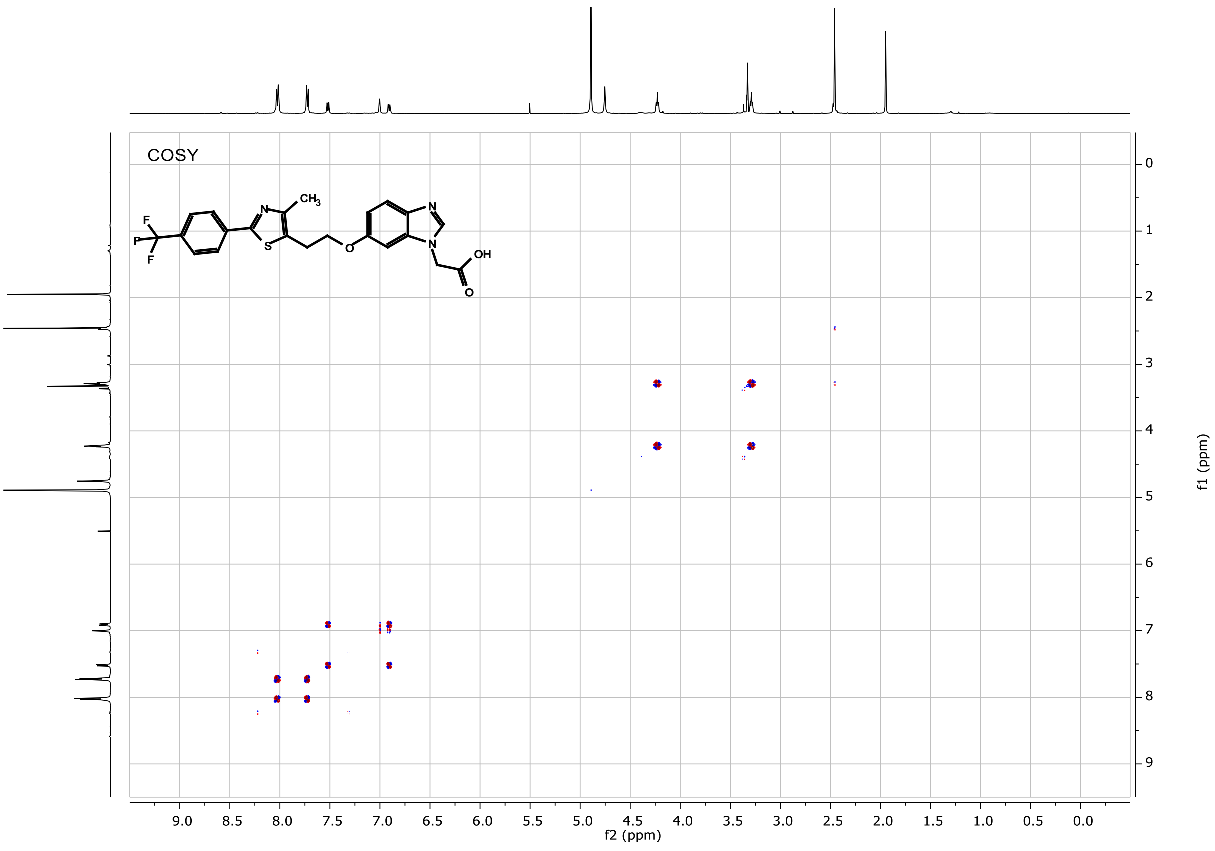
COSY
f2 (ppm)
f1 (ppm)

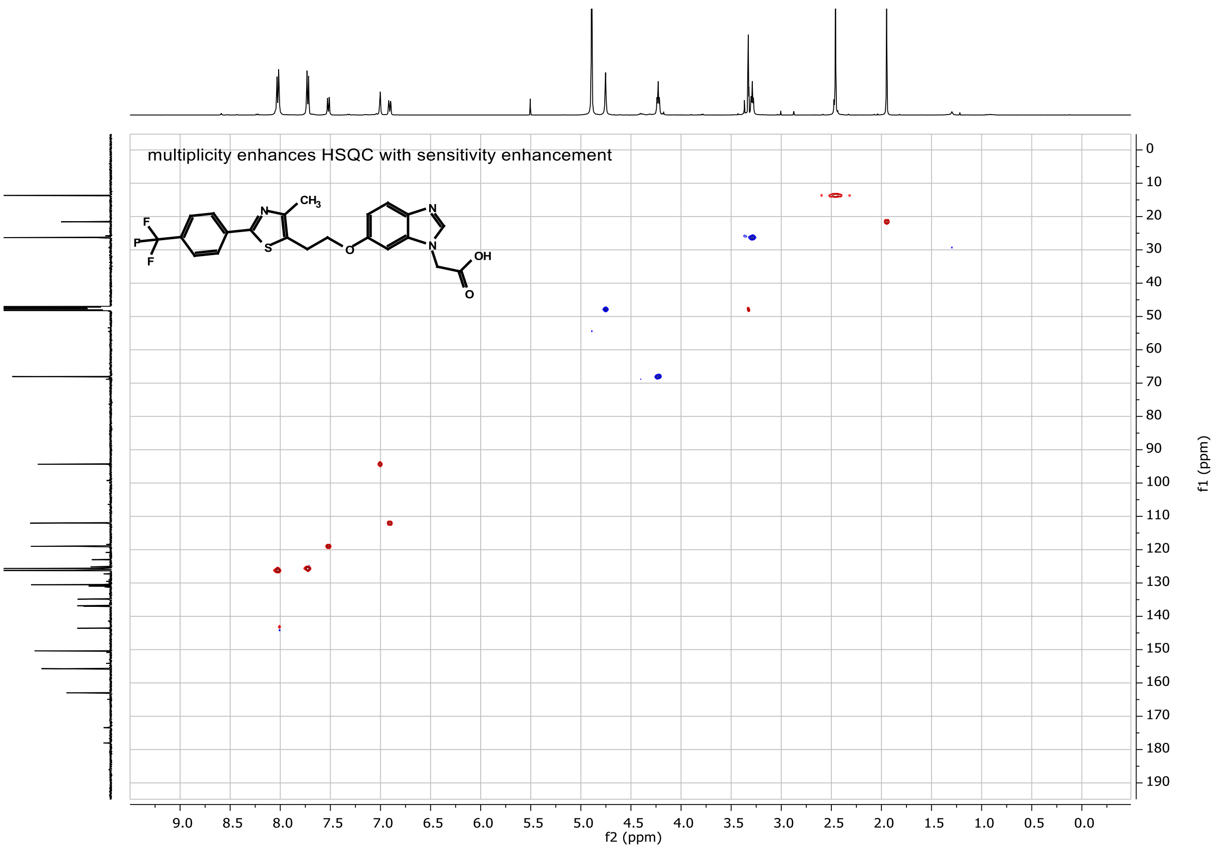
multiplicity enhances HSQC with sensitivity enhancement
f2 (ppm)
f1 (ppm)

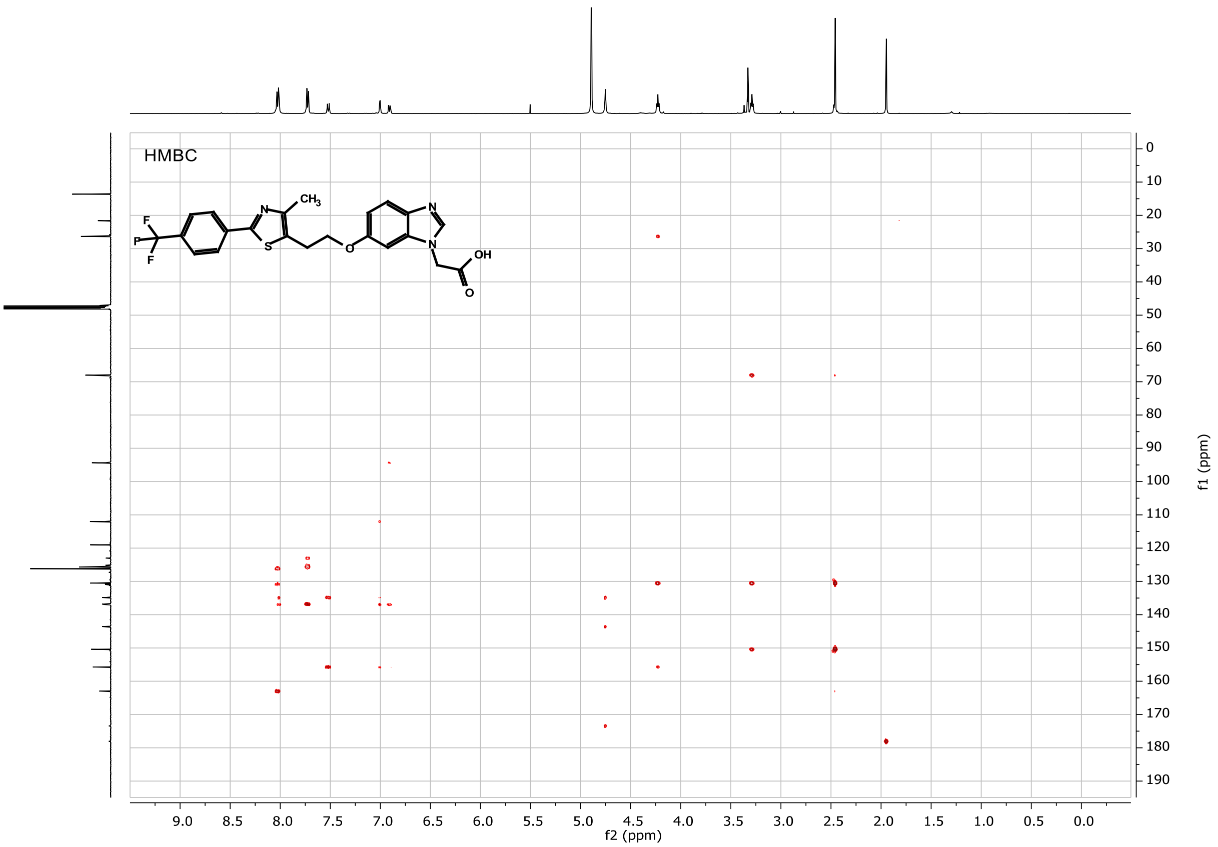
HMBC
f2 (ppm)
f1 (ppm)

N-(2-Chloro-5-(1-methyl-1*H*-benzo[*d*]imidazol-6-yl)pyridin-3-yl)benzenesulfonamide (**2ao**)

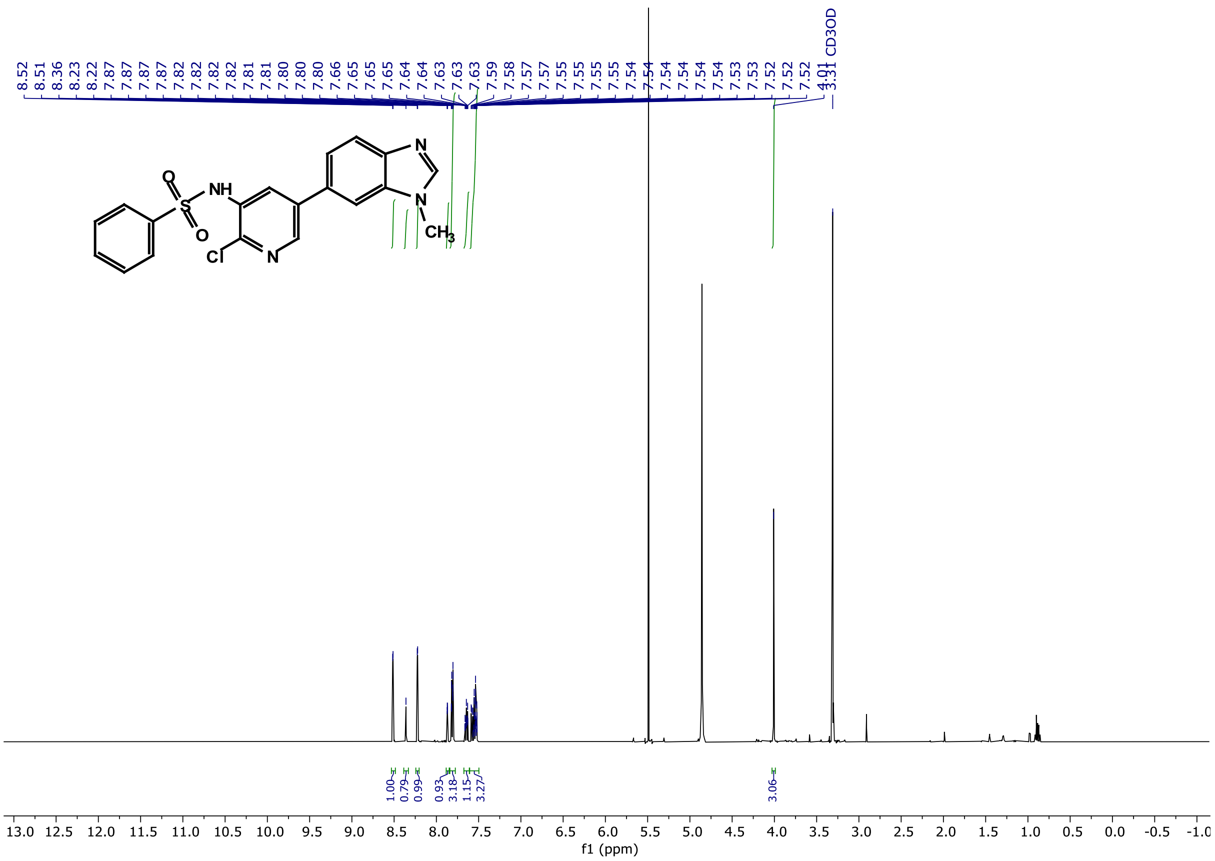

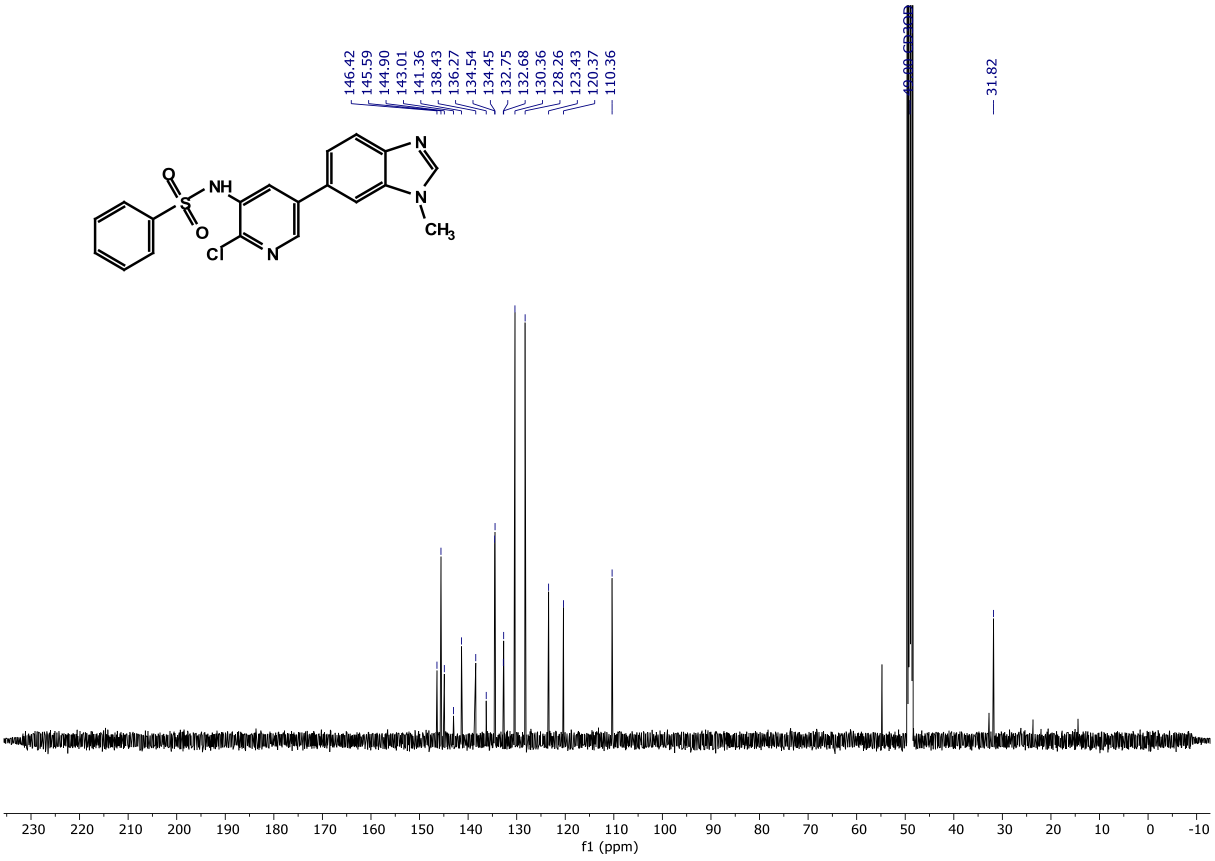

2-Ethoxy-3-(1-((2-(4-isopropylphenyl)-5-methyloxazol-4-yl)methyl)-4-methyl-1*H*-benzo[*d*]imidazol-5-yl)propanoic acid (**2ap**)

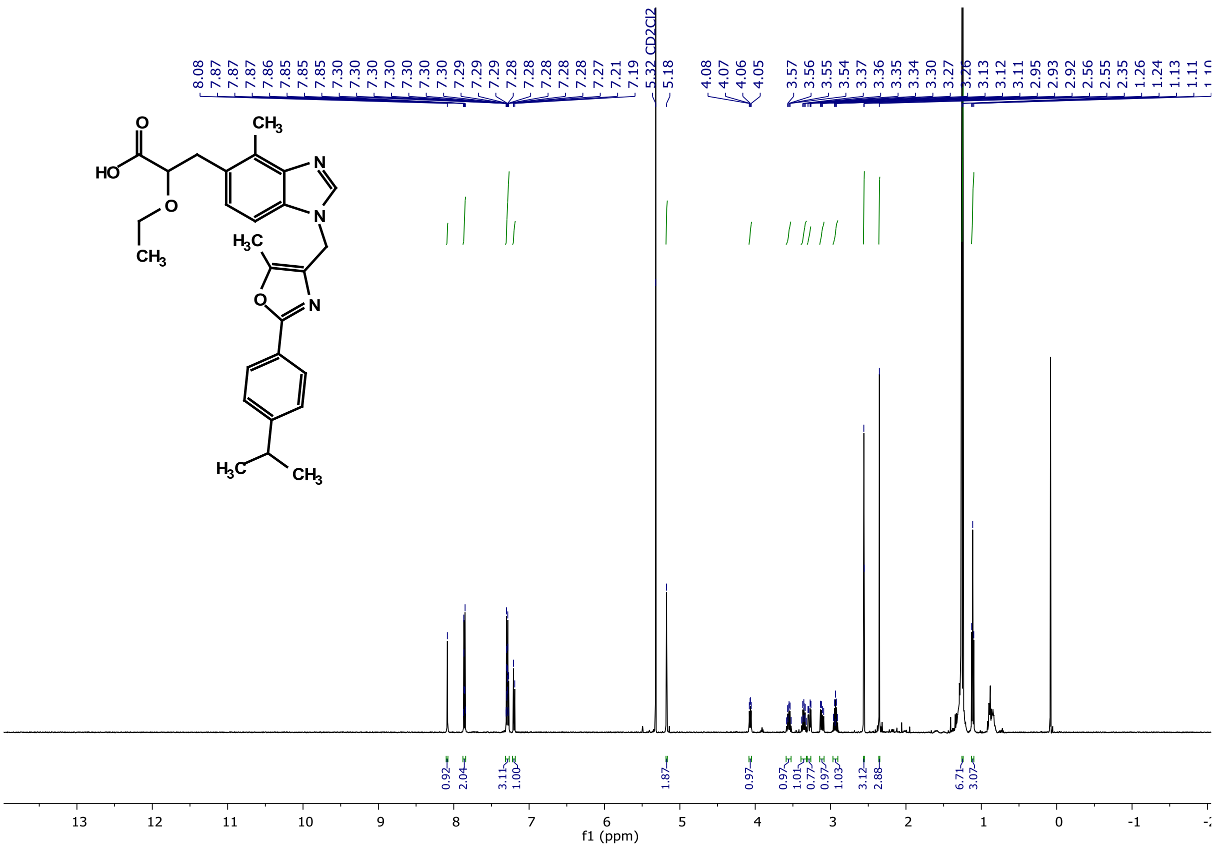

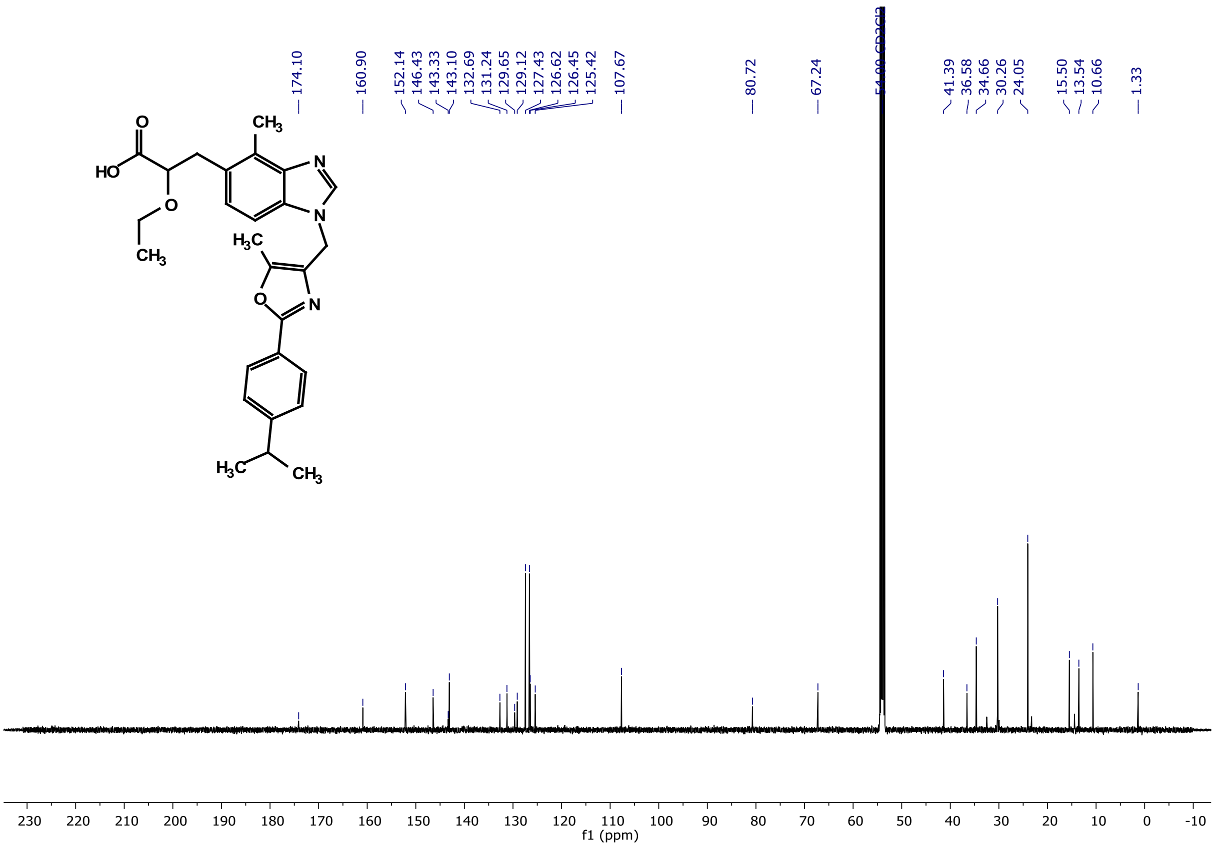

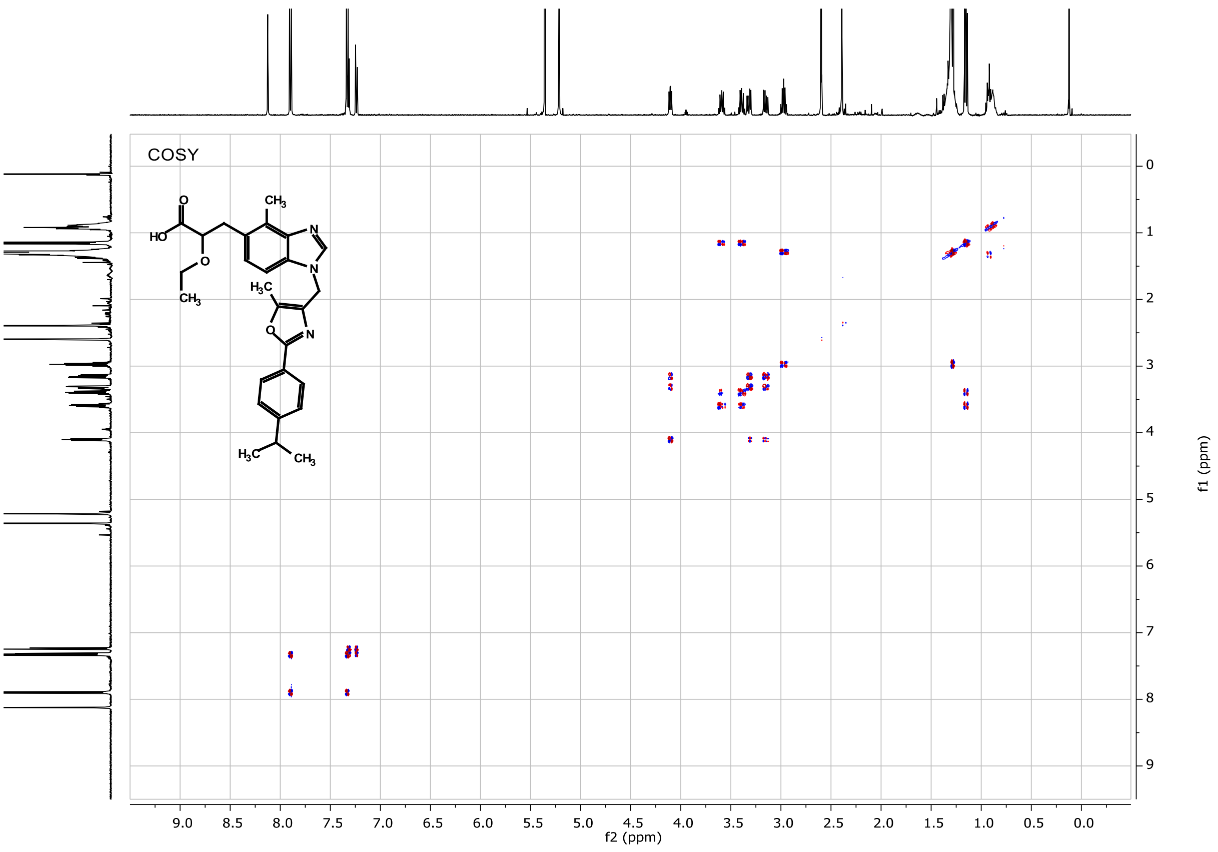
COSY
HO
O
CH3
N
H3C
O
N
f2 (ppm)
f1 (ppm)

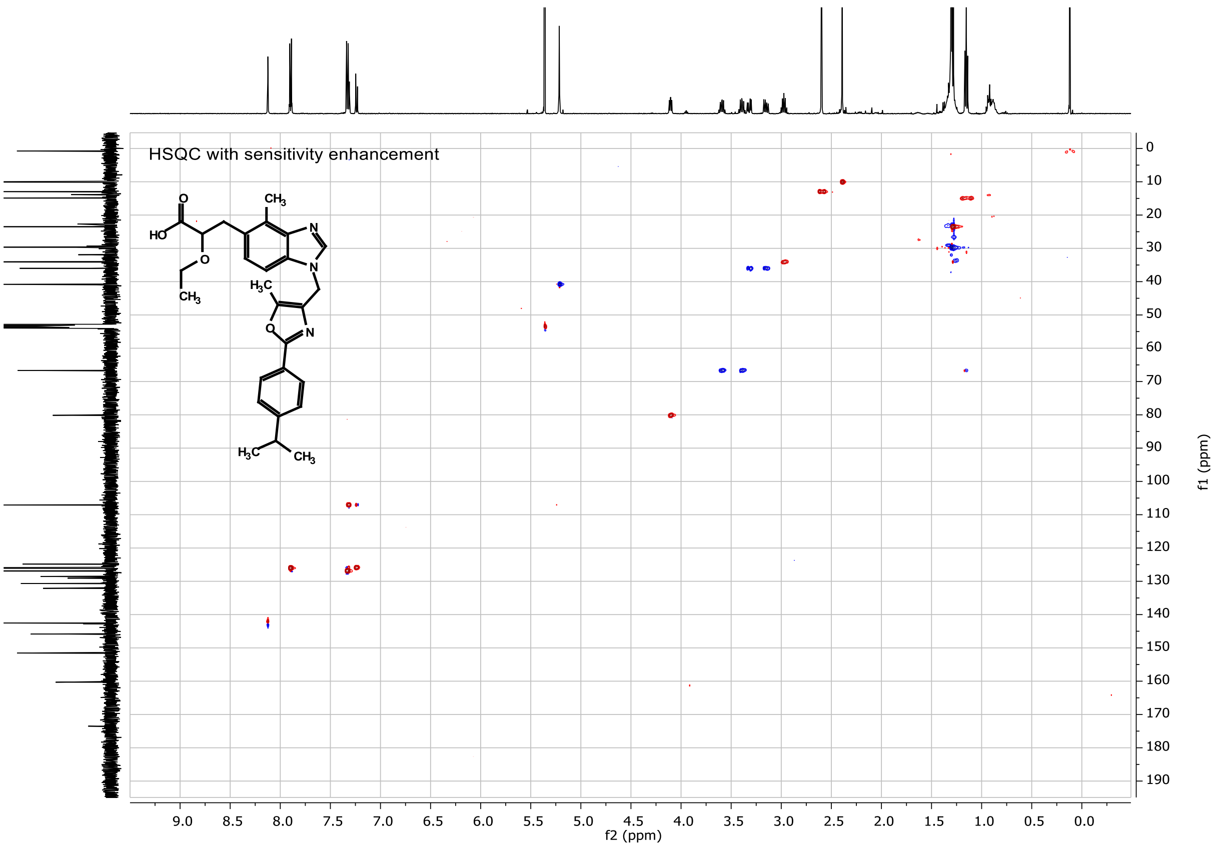
HSQC with sensitivity enhancement
HO
O
CH3
N
H3C
O
N
f2 (ppm)
f1 (ppm)

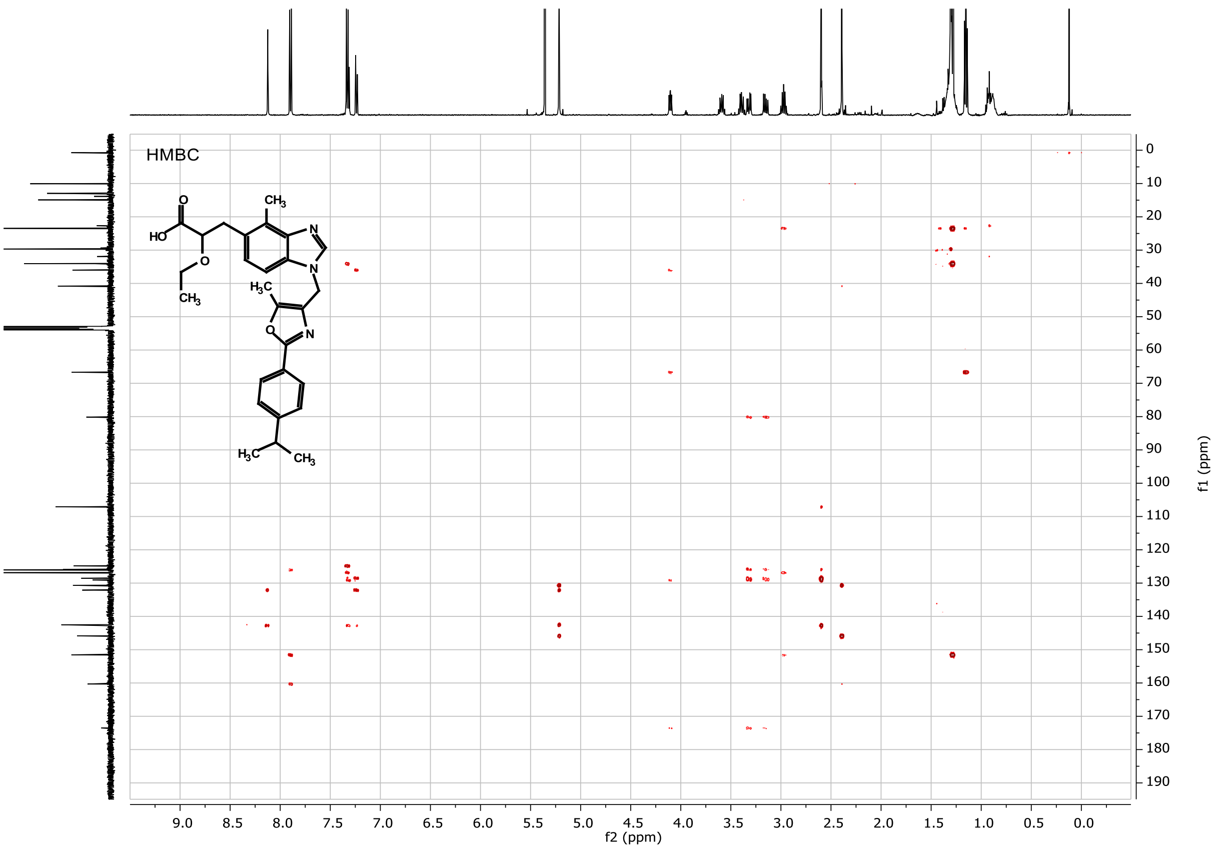
HMBC
f1 (ppm)
f2 (ppm)

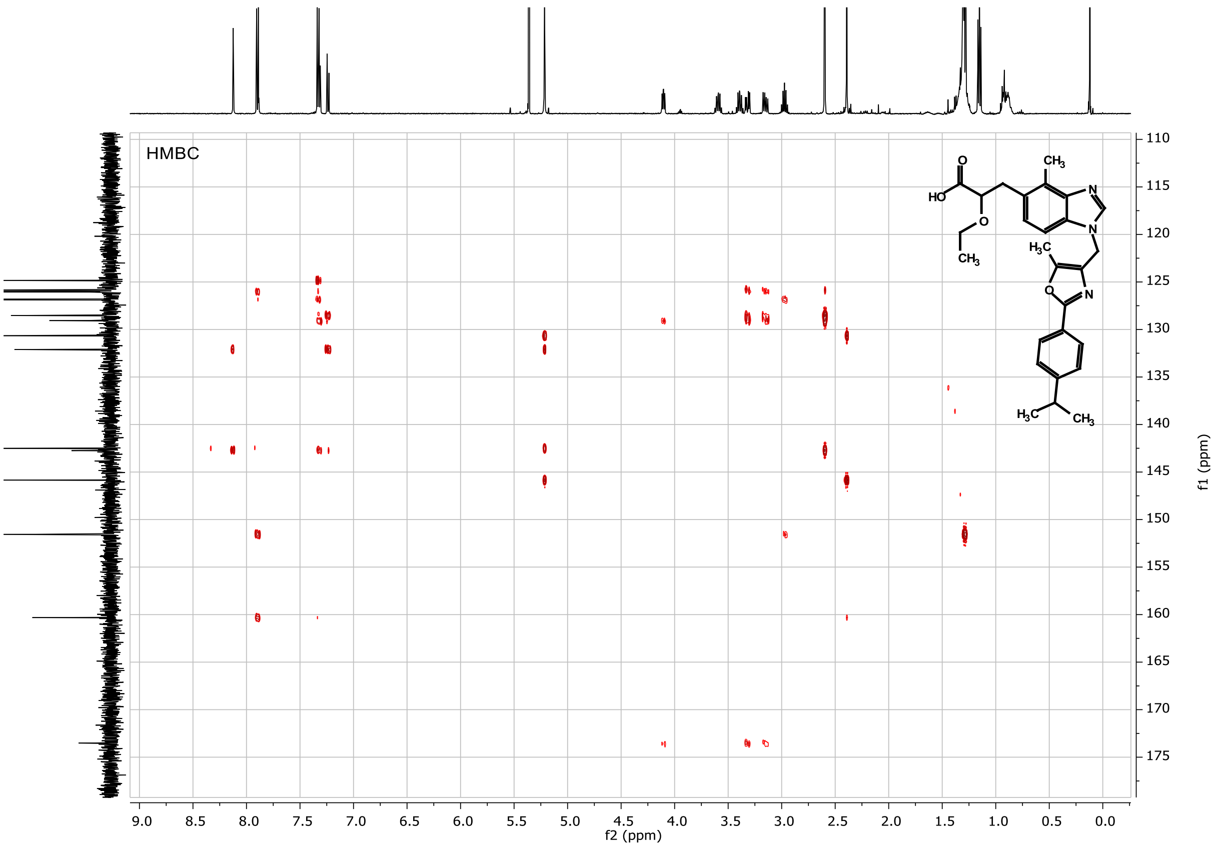
HMBC
f1 (ppm)
f2 (ppm)

3,3-Dimethyl-1-(1-methyl-1*H*-benzo[*d*]imidazol-6-yl)-6-(2-methylpyrimidin-5-yl)indolin-2-one (**2aq**)

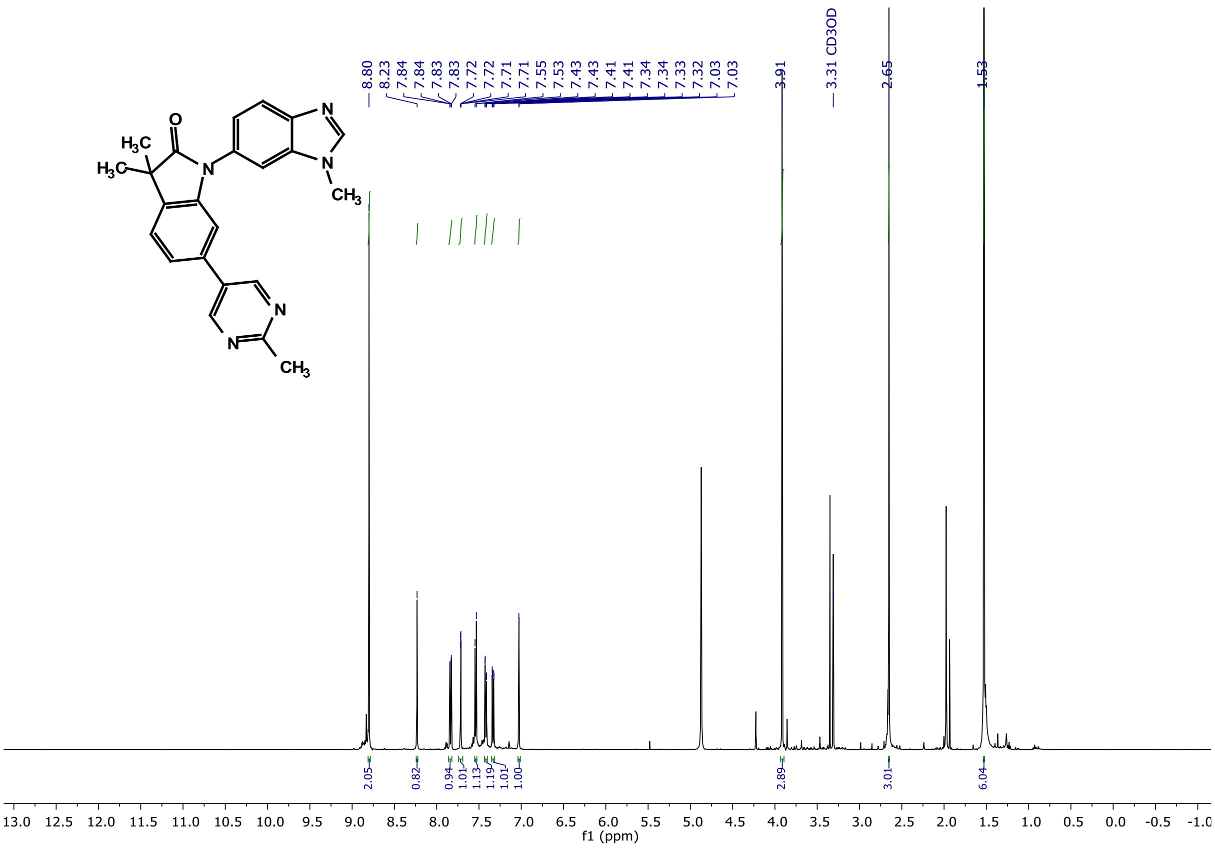

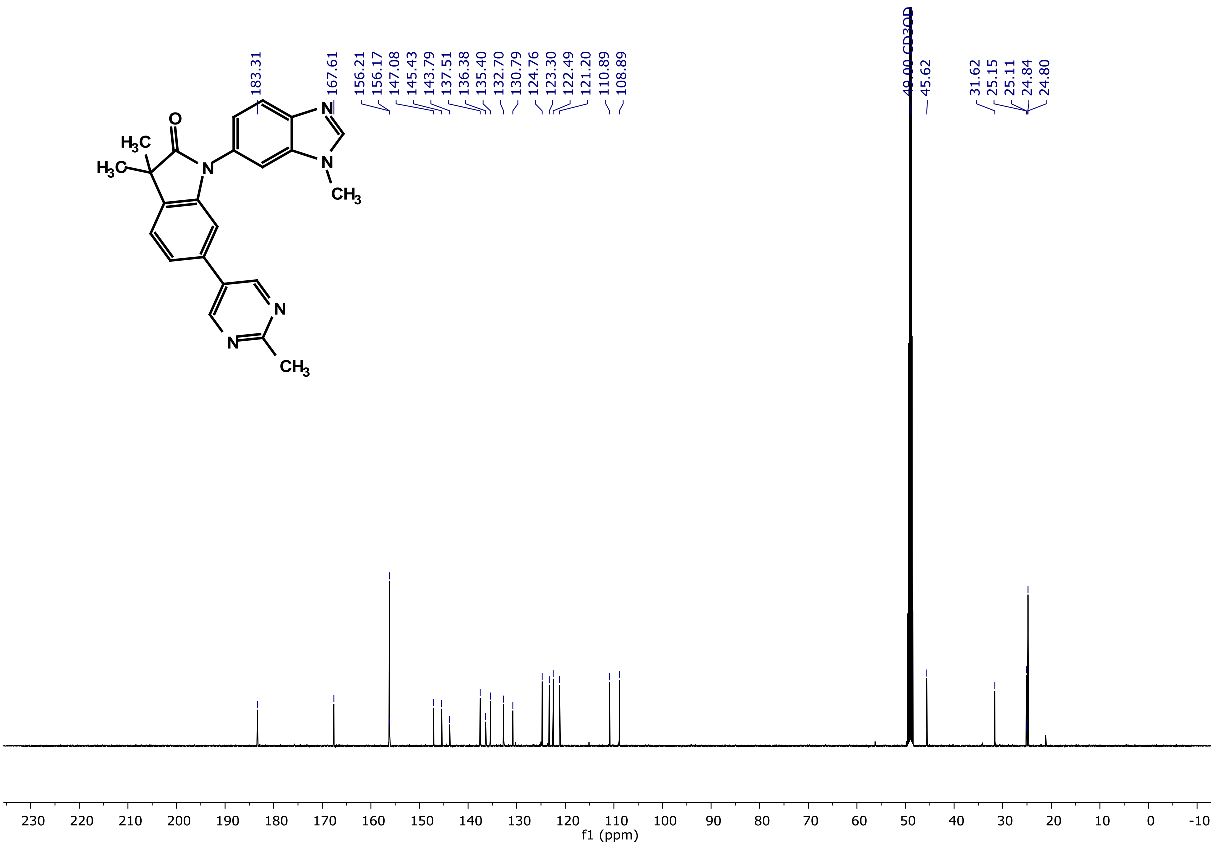

4-(6-Chloro-2-(1-methyl-1*H*-benzo[*d*]imidazol-4-yl)pyrimidin-4-yl)morpholine (**2ar**)

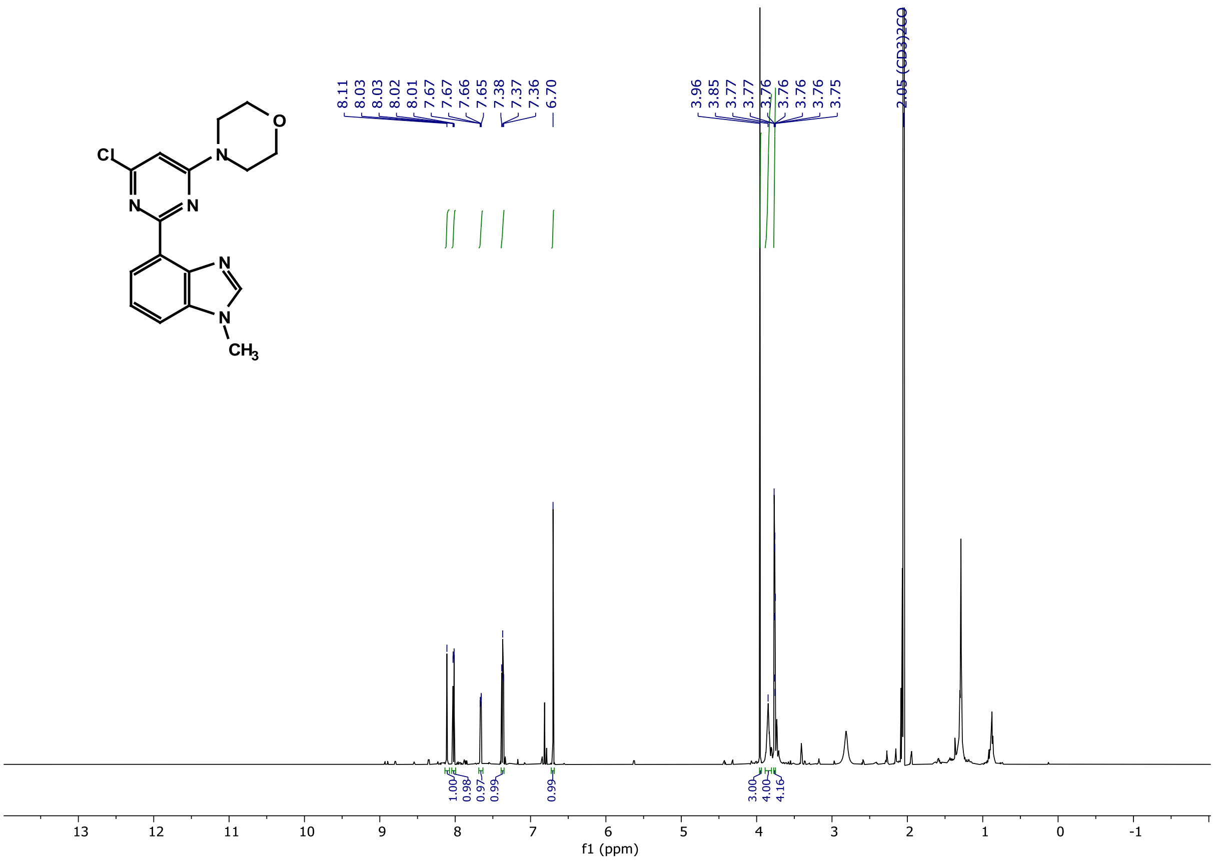

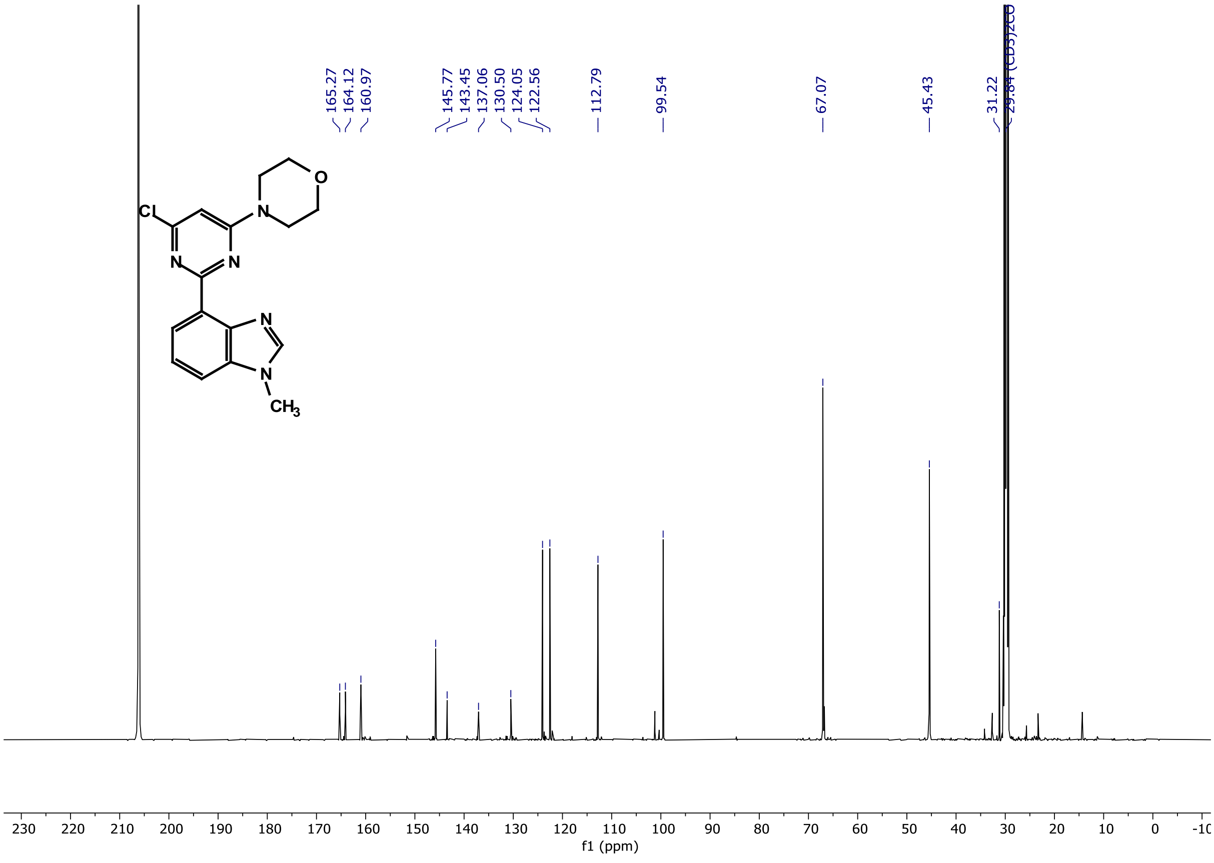

Methyl 1-ethyl-4-((2-(trimethylsilyl)ethoxy)methoxy)-1*H*-benzo[*d*]imidazole-6-carboxylate (**2as**)

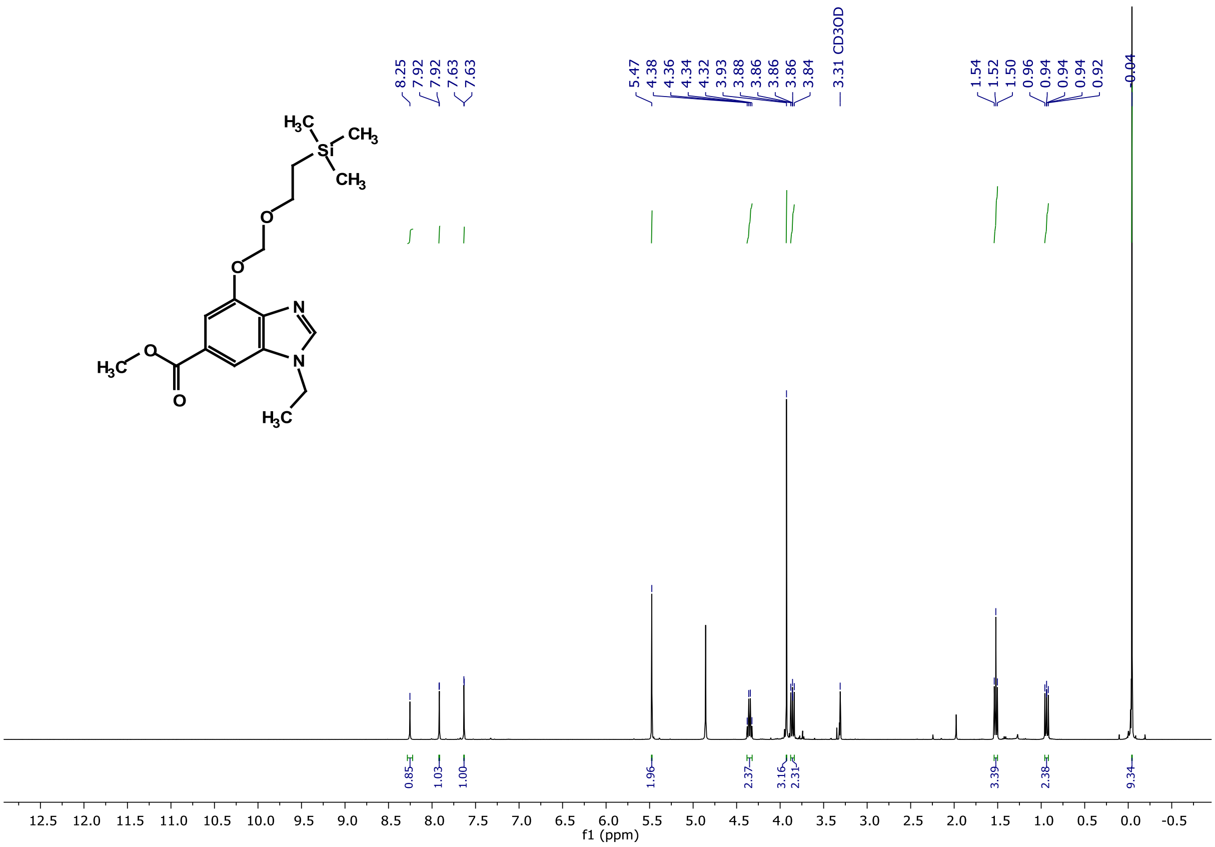

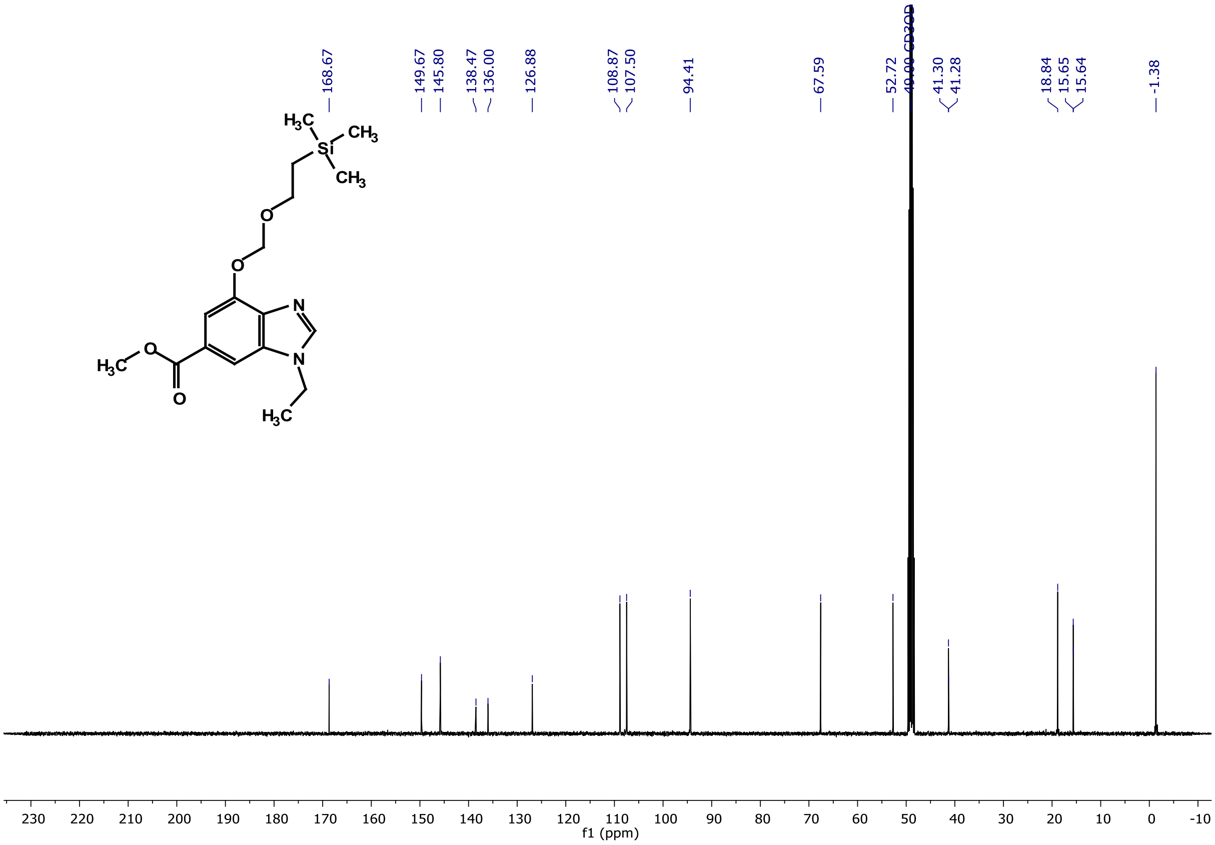

Ethyl 2-ethoxy-3-(1-(3-(5-methyl-2-phenyloxazol-4-yl)propyl)-1*H*-benzo[*d*]imidazol-4-yl)propanoate (**2at**)

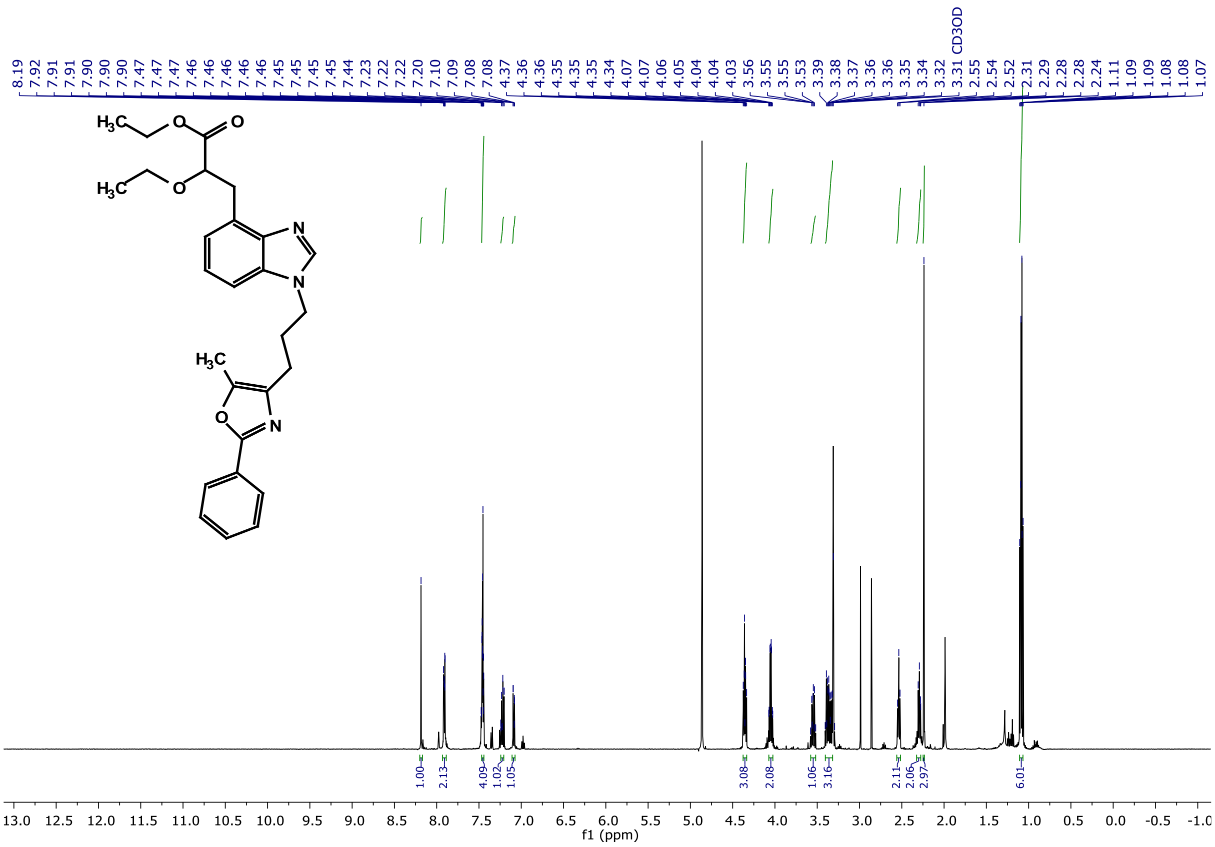

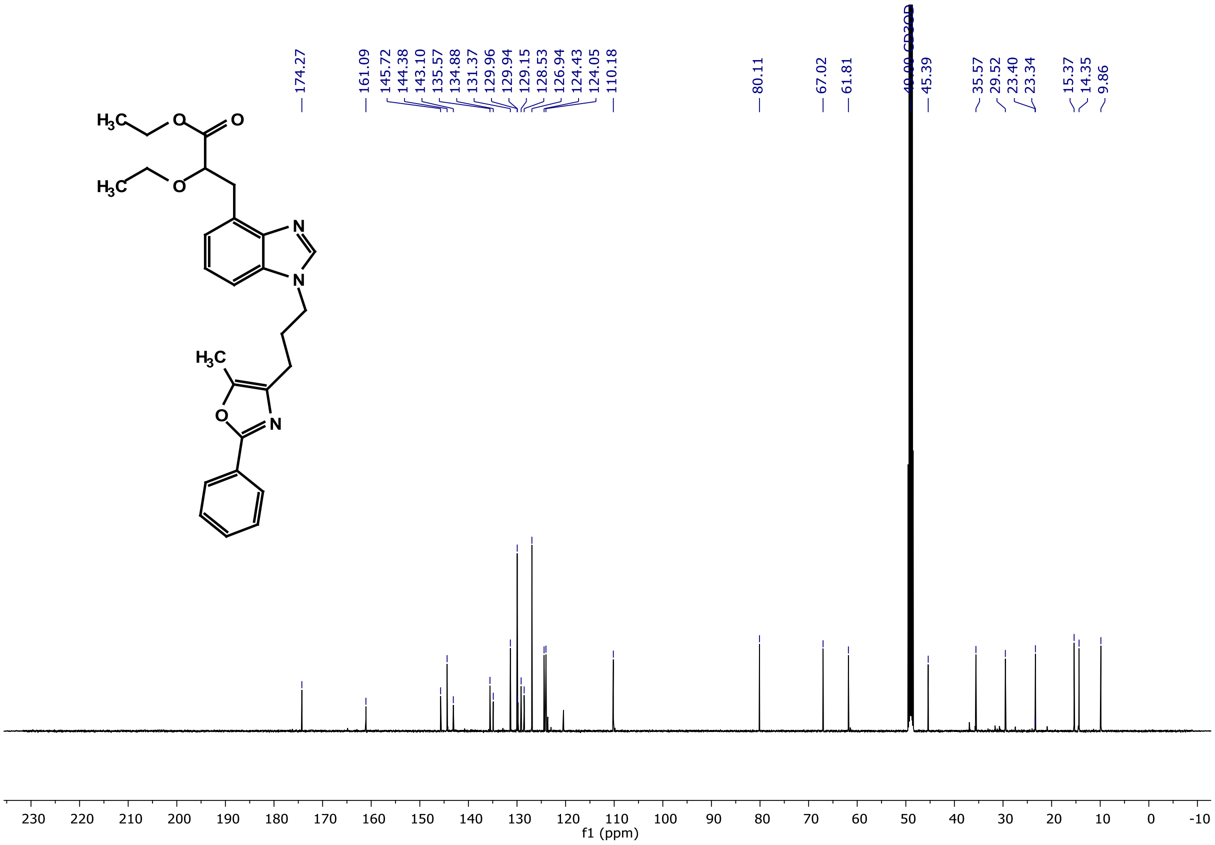

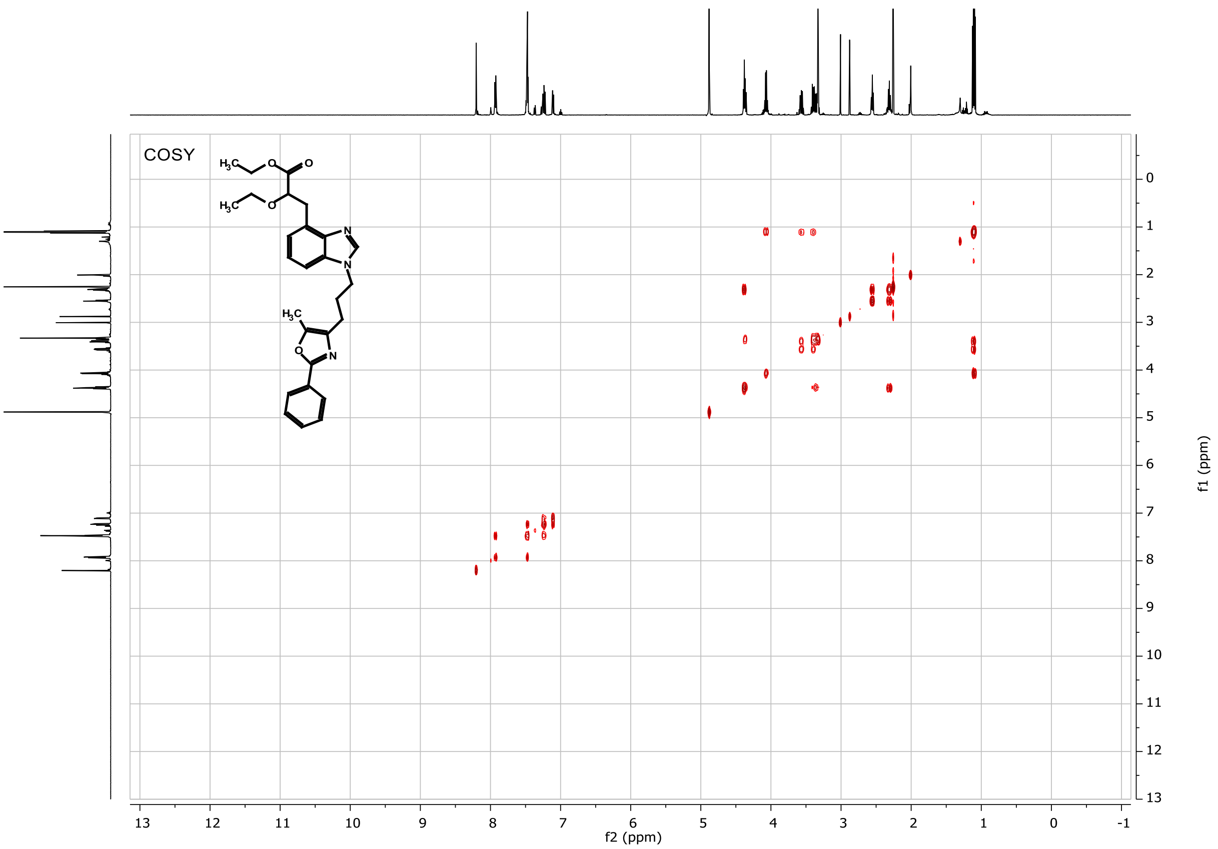
COSY
f2 (ppm)
f1 (ppm)

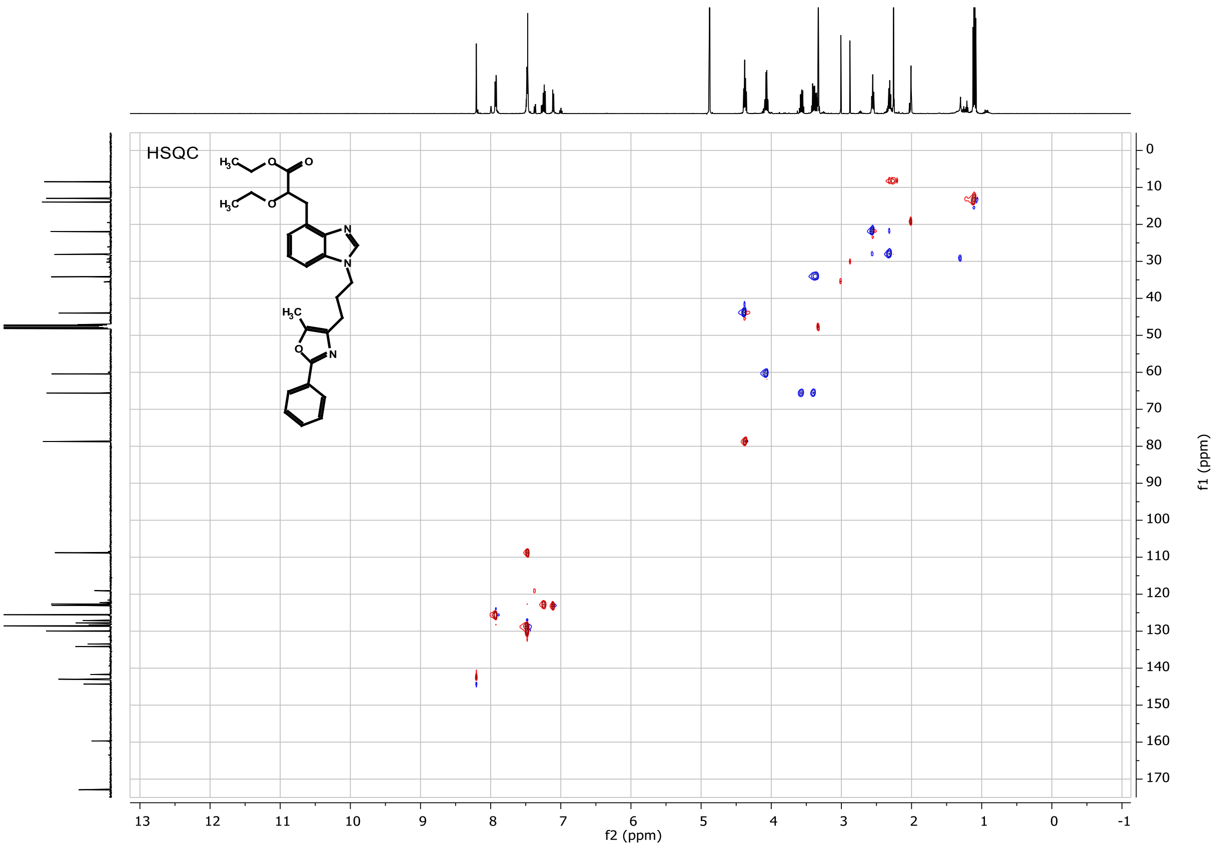
HSQC
f2 (ppm)
f1 (ppm)

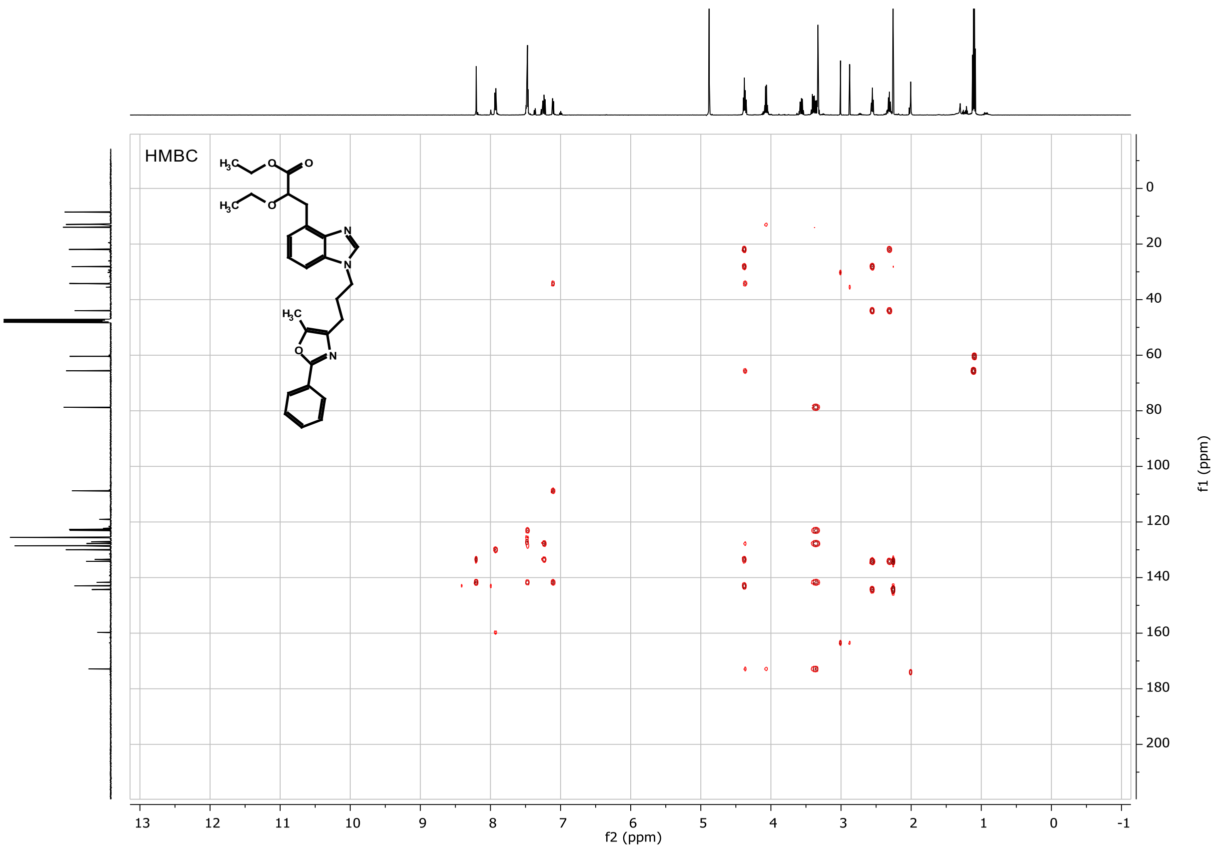

2-Ethoxy-3-(1-((5-methyl-2-(4-(trifluoromethyl)phenyl)oxazol-4-yl)methyl)-1*H*-benzo[*d*]imidazol-4-yl)propanoic acid (**2au**)

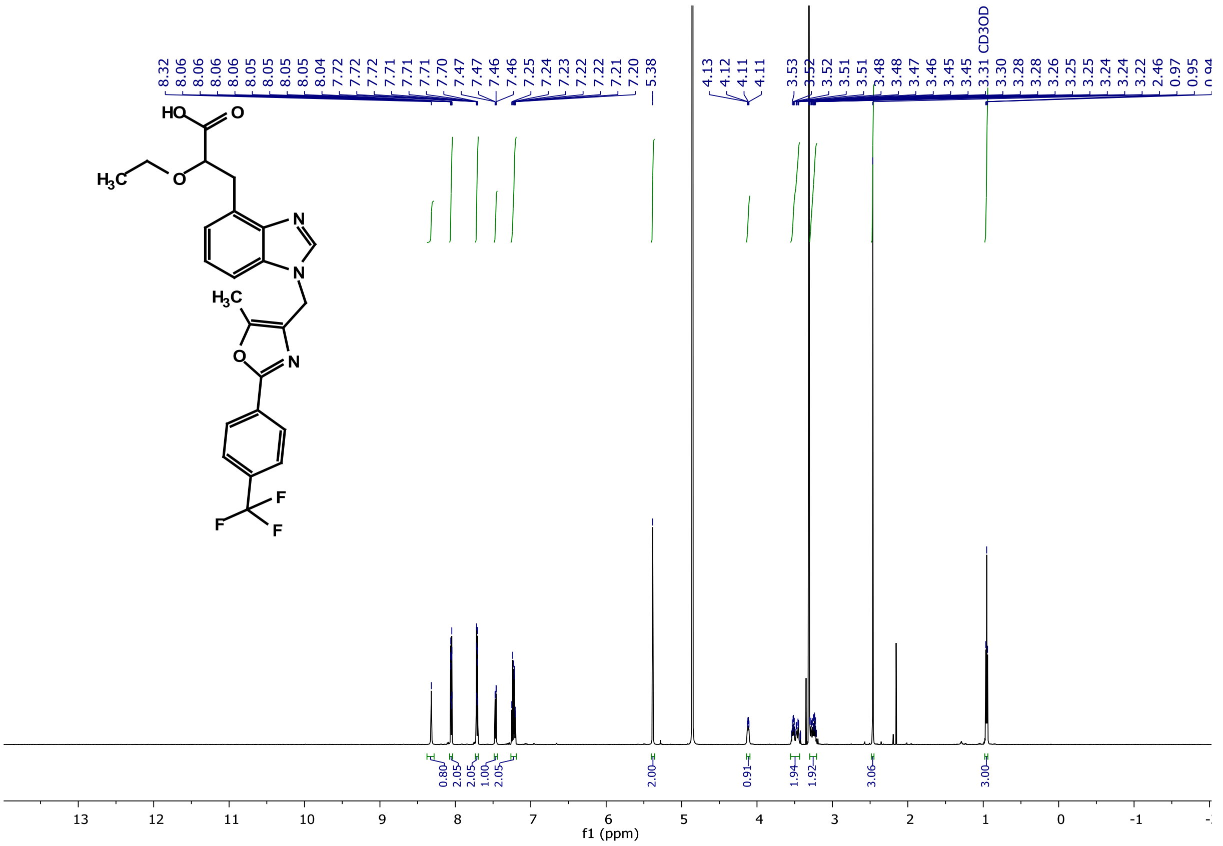

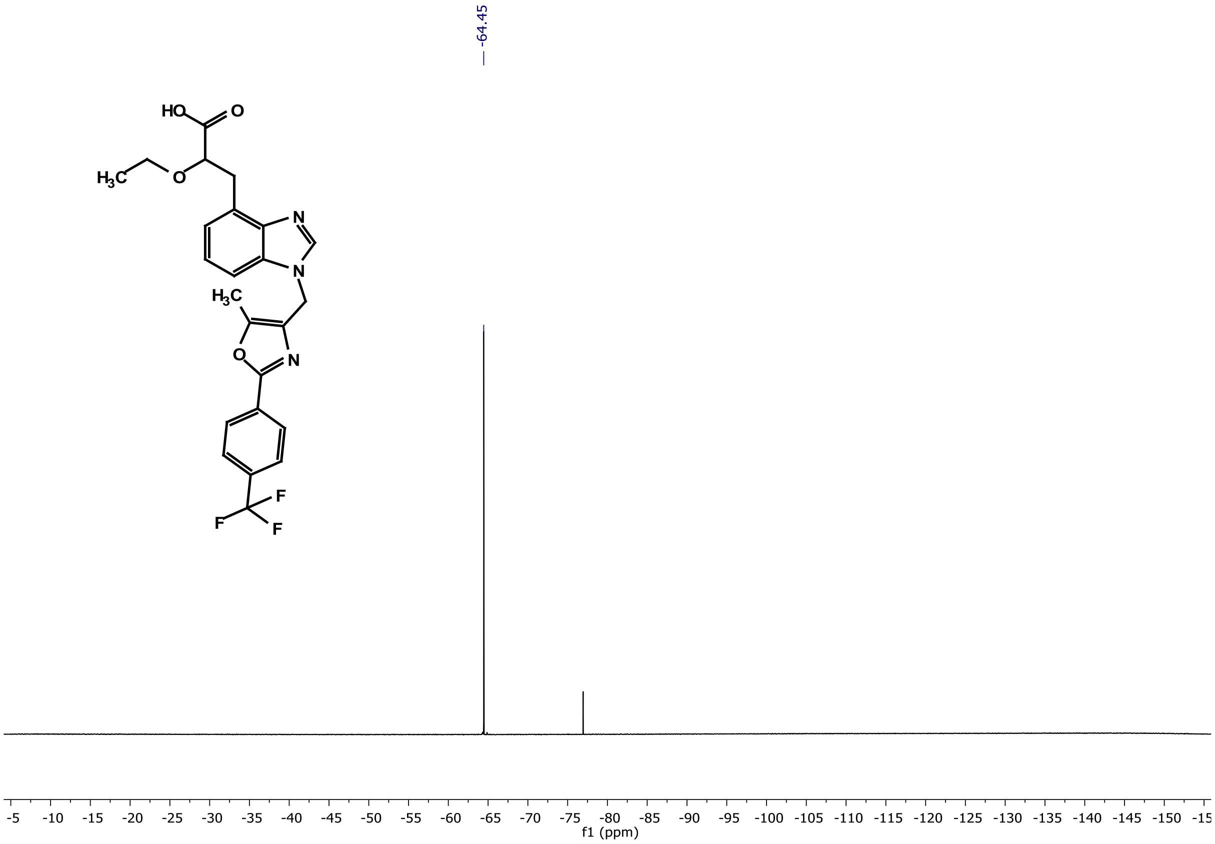
-64.45
HO
O
H3C
O
N
N
H3C
O
N
F
F
F
-5 -10 -15 -20 -25 -30 -35 -40 -45 -50 -55 -60 -65 -70 -75 -80 -85 -90 -95 -100 -105 -110 -115 -120 -125 -130 -135 -140 -145 -150
f1 (ppm)

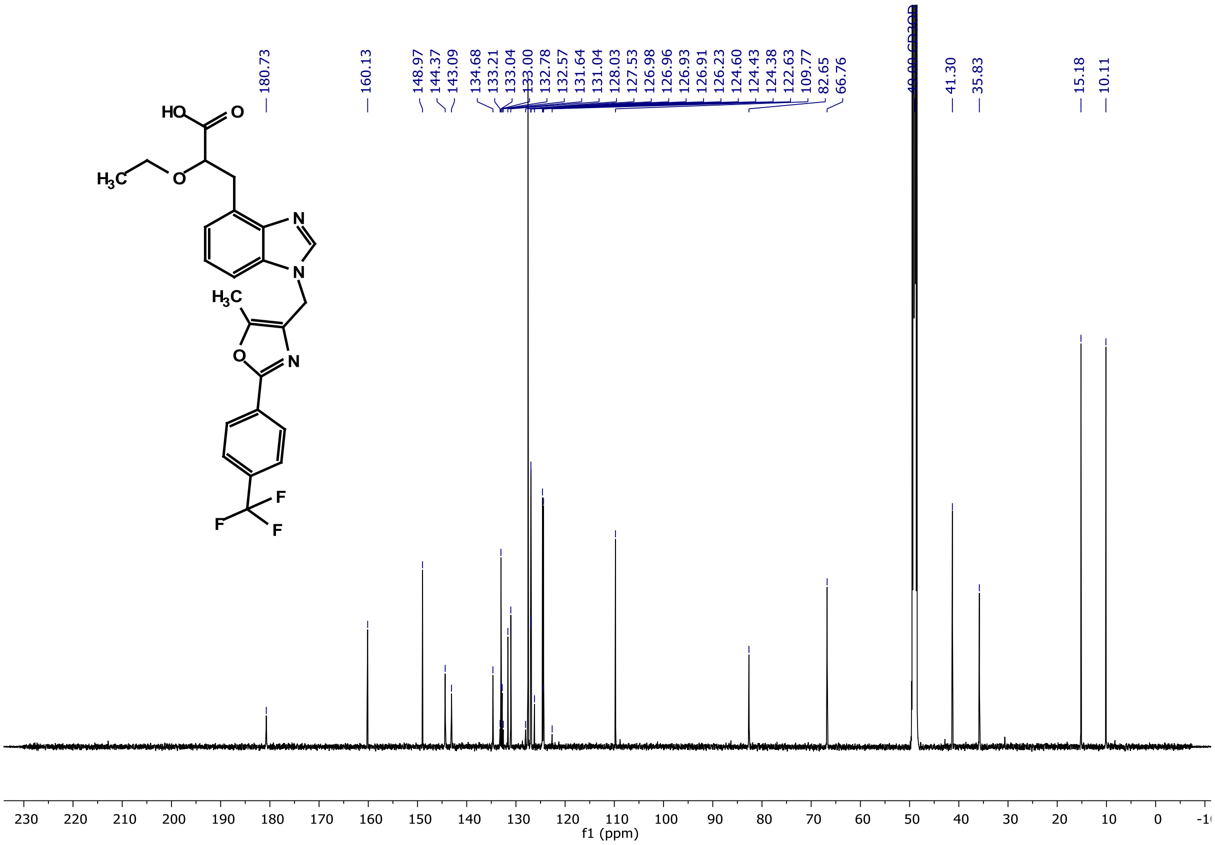
180.73
160.13
148.97
144.37
143.09
134.68
133.21
133.04
133.00
132.78
132.57
131.64
131.04
128.03
127.53
126.98
126.96
126.93
126.91
126.23
124.60
124.43
124.38
122.63
109.77
82.65
66.76
41.30
35.83
15.18
10.11
HO
O
H3C
O
N
N
H3C
O
N
F
F
F
230 220 210 200 190 180 170 160 150 140 130 120 110 100 90 80 70 60 50 40 30 20 10 0
f1 (ppm)

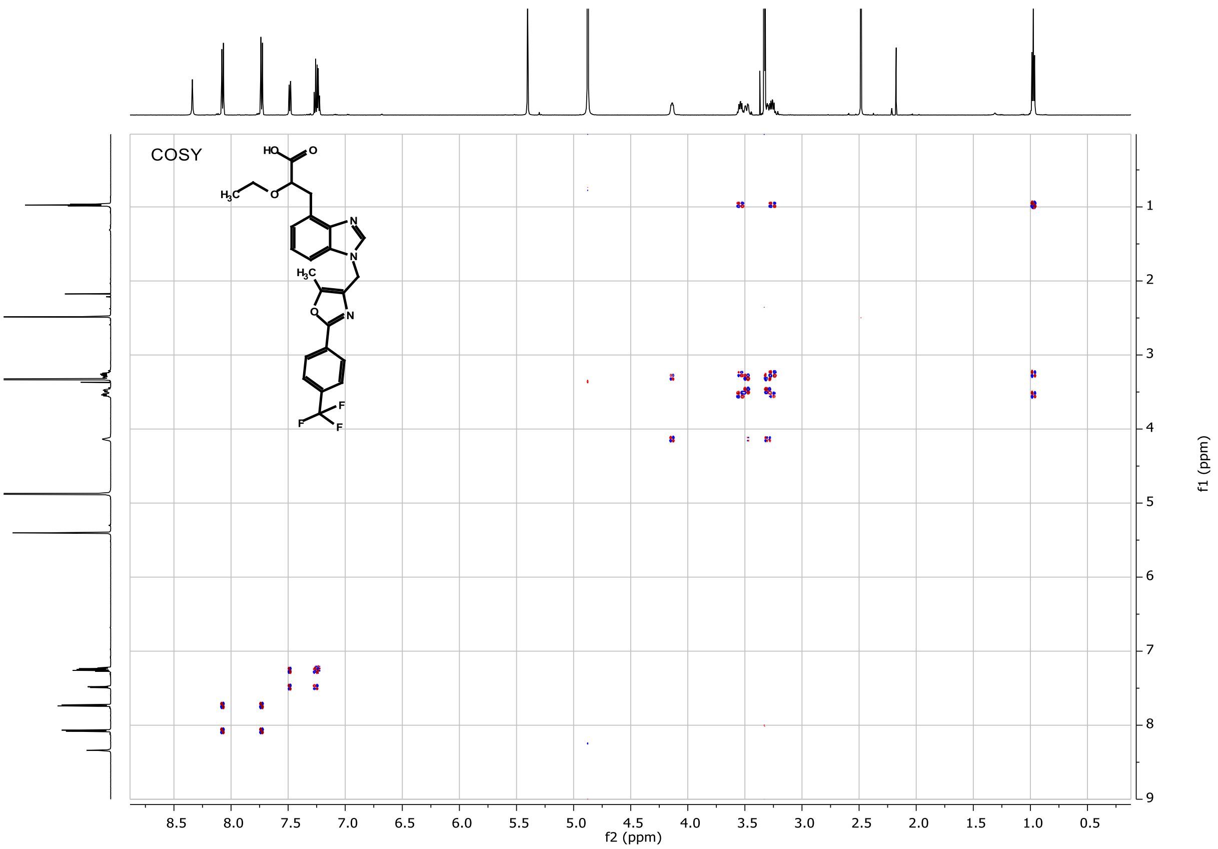
COSY
f2 (ppm)
f1 (ppm)

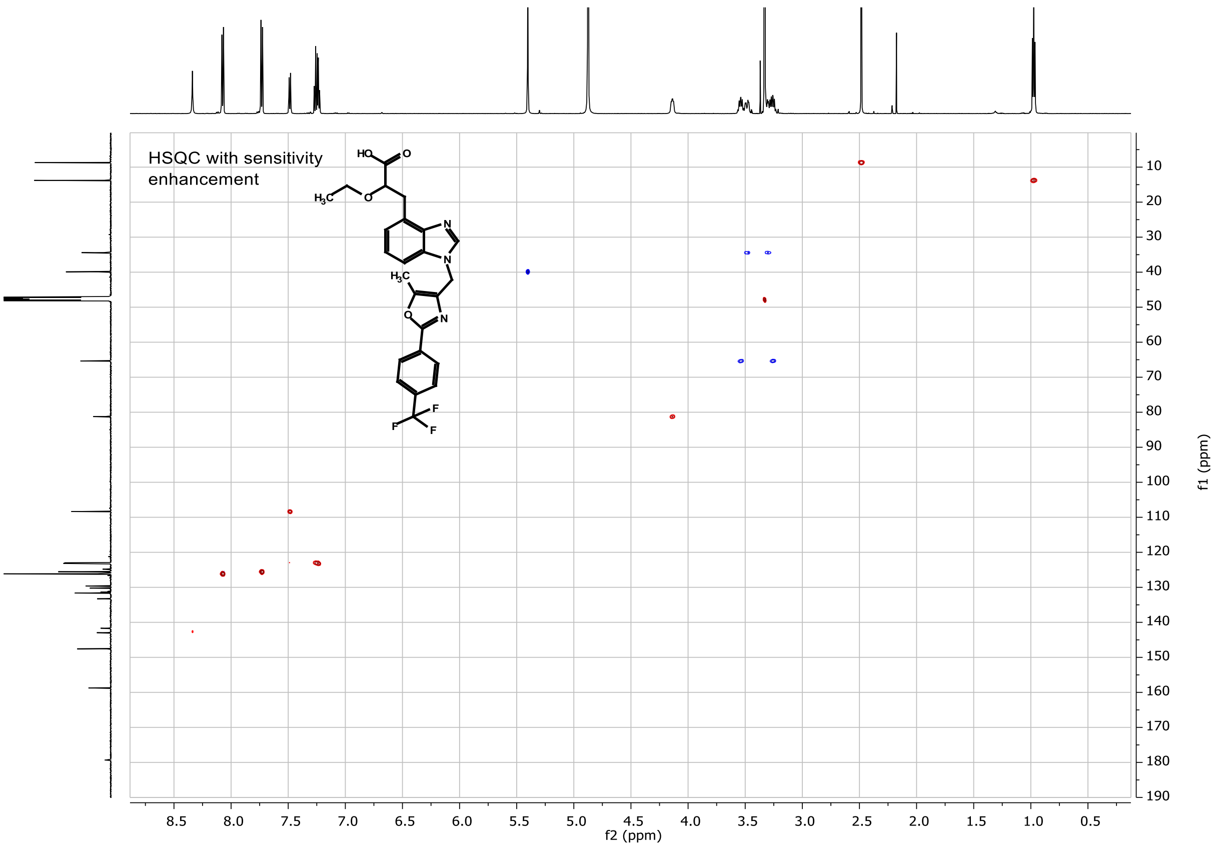
HSQC with sensitivity
enhancement
f2 (ppm)
f1 (ppm)

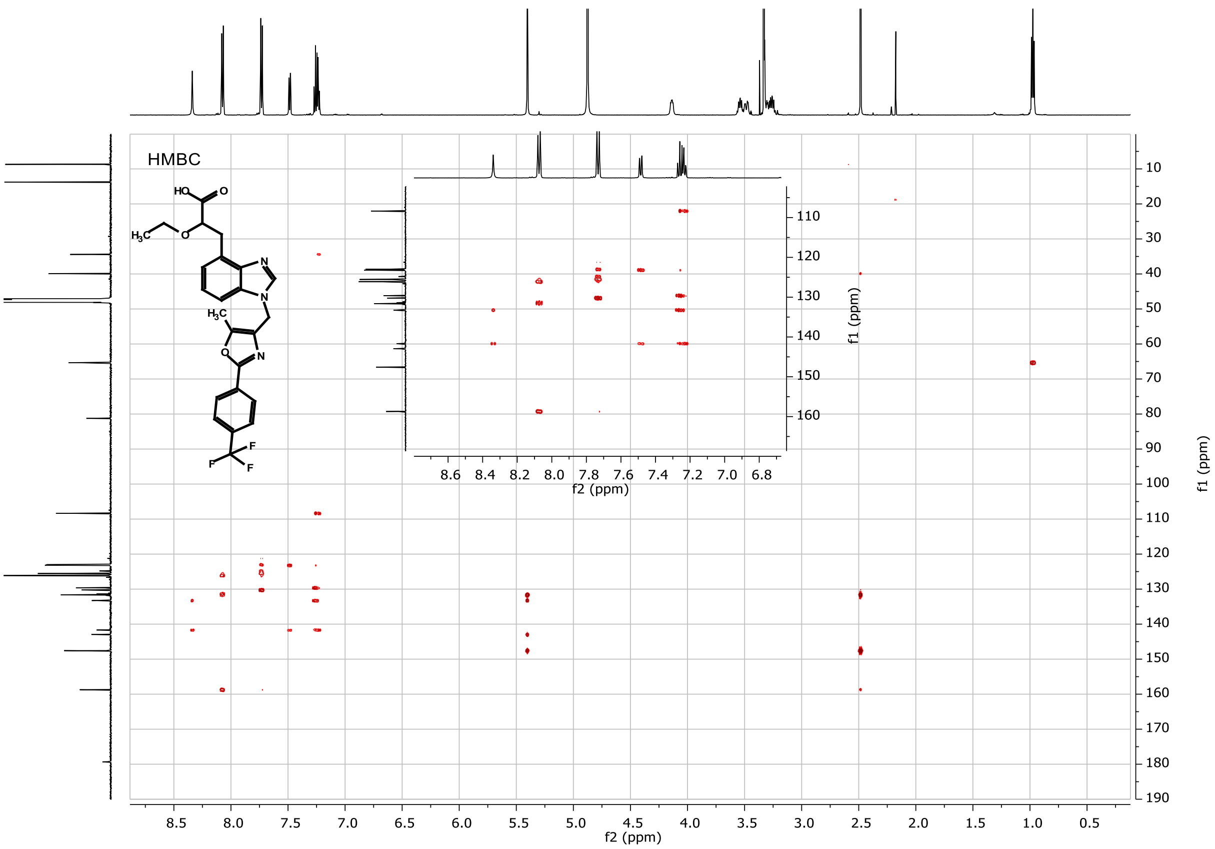
HMBC
f2 (ppm)
f1 (ppm)
